# Iridium‐Catalyzed Enantioselective Intermolecular Indole C2‐Allylation

**DOI:** 10.1002/anie.202001956

**Published:** 2020-03-11

**Authors:** James A. Rossi‐Ashton, Aimee K. Clarke, James R. Donald, Chao Zheng, Richard J. K. Taylor, William P. Unsworth, Shu‐Li You

**Affiliations:** ^1^ Department of Chemistry University of York York YO10 5DD UK; ^2^ State Key Laboratory of Organometallic Chemistry Shanghai Institute of Organic Chemistry Chinese Academy of Sciences 345 Lingling Lu Shanghai 200032 China

**Keywords:** allylic substitution, DFT calculations, enantioselective synthesis, indole, iridium

## Abstract

The enantioselective intermolecular C2‐allylation of 3‐substituted indoles is reported for the first time. This directing group‐free approach relies on a chiral Ir‐(P, olefin) complex and Mg(ClO_4_)_2_ Lewis acid catalyst system to promote allylic substitution, providing the C2‐allylated products in typically high yields (40–99 %) and enantioselectivities (83–99 % *ee*) with excellent regiocontrol. Experimental studies and DFT calculations suggest that the reaction proceeds via direct C2‐allylation, rather than C3‐allylation followed by in situ migration. Steric congestion at the indole‐C3 position and improved π–π stacking interactions have been identified as major contributors to the C2‐selectivity.

## Graphical abstract

**An enantioselective** C2‐allylation of 3‐substituted indoles is reported, providing C2‐allylated products with excellent regiocontrol. Experimental studies and DFT calculations suggest that the reaction proceeds via direct C2‐allylation with steric congestion at the indole‐C3 position and π–π stacking interactions identified as major contributors to the selectivity observed.

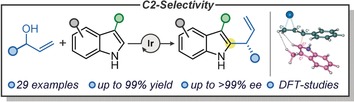

## Introduction

Indole is a core structural element in many natural and synthetic organic compounds that possess a wide diversity of important biological activities.^1^ Allylation of the indole core is a fundamental transformation, integral to numerous synthetic processes.^2^ Consistent with the innate reactivity of indoles, intermolecular asymmetric allylation of the C3‐position of indole is a well‐established process.^3^ The analogous N1‐allylation is relatively less well‐explored, although there exist various innovative methods for this recently developed by the groups of Hartwig,^4^ You,^5^ Krische^6^ and others.^7^ To the best of our knowledge, there are no methods capable of directly allylating indole at the C2‐position in an enantioselective, intermolecular fashion.

**Scheme 1:**
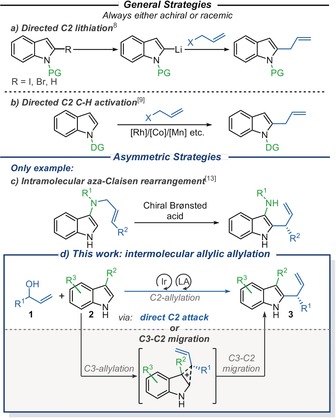
Intermolecular indole C2‐allylation.

Most intermolecular indole C2‐allylation strategies involve either directed lithiation^8^ (by lithium–halogen exchange or deprotonation) of the C2‐position (Scheme 1, a), or C−H activation orchestrated by a directing group (typically attached at the N1‐position) (Scheme 1, b).^9^, ^10^ Despite the numerous merits of these approaches, their dependency on directing groups is an obvious drawback, requiring additional installation and removal steps. Furthermore, these strategies typically result in the formation of achiral, linear allylated products, and the rare examples that form branched products generally require more forcing reaction conditions, are often low yielding, and are all racemic.9c–9g

Intramolecular reactions can overcome the regioselectivity issues associated with intermolecular indole reactivity.^11^ Several asymmetric indole C2‐allylation procedures have been developed following this approach.^12^ However, these strategies almost always lead to the formation of annulated C2‐allylated indoles. A search of the literature revealed that the sole exception to this generalization comes from Tambar et al.^13^ who developed an innovative intramolecular route to branched, highly enantioenriched C2‐allylated 3‐amino indoles employing an enantioselective aza‐Claisen rearrangement (Scheme 1, c).

In this study, we demonstrate the successful implementation of a strategy to access enantioenriched C2‐allylated 3‐substituted^14^, ^15^ indoles **3** via the intermolecular allylic substitution of branched allylic alcohols **1** and readily available indoles **2**, catalyzed by a chiral Ir‐(P, olefin) complex^16^ and a Lewis acid additive (Scheme 1 d). At the start of this study, gaining effective control over the regioselectivity of the allylic substitution step with respect to the indole (especially C2‐ versus C3‐substitution) was expected to be a key challenge. This motivated our decision to explore the use of Lewis acidic additives, as we postulated that under these reaction conditions, allylation of indole **2** at either its C2‐ or C3‐position^17^ would result in the convergent formation of the desired C2‐allylated product **3**; either, indole **2** could react with the π‐allyl iridium complex via the less sterically hindered C2‐position, to form allylated product **3** directly, or alternatively, it could react via the more electron‐rich C3‐position but then undergo an in situ stereospecific migration.^18^ In this latter scenario, the Lewis acid (that is essential for the activation of allylic alcohol **1**) would also help to promote the required stereospecific migration. Mechanistic and computational studies (see below) suggest that direct C2‐allylic substitution is the dominant pathway in the cases tested, but crucially, because both mechanistic pathways converge to the same product **3**, this means that effective C2‐allylation can be achieved even in cases in which C3‐allylic substitution competes.

## Results and Discussion

Our investigation started with the identification of suitable reaction conditions (see Supporting Information for full details), including finding a Lewis acid capable of performing up to three key roles within the allylation‐migration cascade: 1) to activate the allylic alcohol towards formation of the Ir–π‐allyl complex, 2) to facilitate the enantioselective C2‐ or C3‐allylation whilst avoiding competing N1‐allylation, and 3) to facilitate the stereospecific migration of the allyl group from the C3‐ to the C2‐position of indole if needed.

**Scheme 2:**
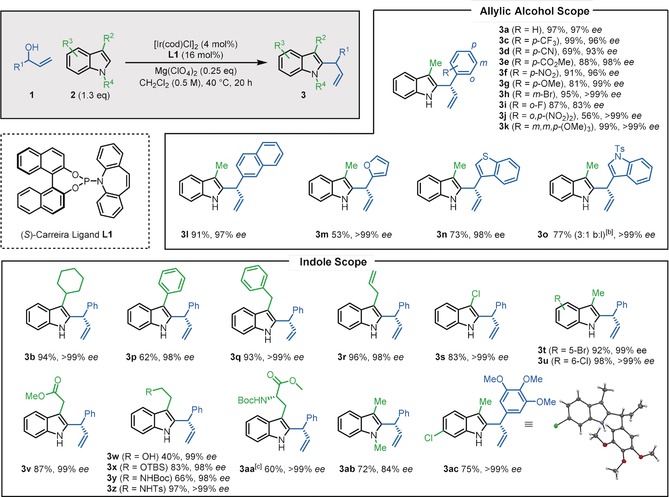
Allylic alcohol and indole substrate scope for C2‐allylation procedure.^[a]^ [a] Yields of isolated products after column chromatography are reported. Enantiomeric excess (*ee*) values were determined by HPLC analysis with a chiral stationary phase. The concentration 0.5 m is with respect to the allylic alcohol. [b] Branched:linear product ratio determined by analysis of ^1^H NMR spectrum of the crude reaction mixture. [c] As a 13:1 mixture of rotamers.

Remarkably, inexpensive Mg(ClO_4_)_2_ (which has never previously been used in iridium‐catalyzed allylic substitution) was identified as the best Lewis acid for the reaction between phenyl allylic alcohol **1 a** and 3‐methyl indole **2 a**, enabling the formation of C2‐allylated indole **3 a** in 99 % yield and 98 % *ee* (Table 1, entry 1) when used in combination with [Ir(cod)Cl]_2_ and the (*S*)‐Carreira ligand (**L1**; see Scheme 2 for its structure).^19^ When exploring the generality of this reaction we recognized that increasing the bulk at the C3‐position of the indole reaction partner (e.g. **2 b**) resulted in a lower yield due to the formation of a ketone side‐product **4** (entry 2), however, this problem was easily rectified by a minor increase in the indole equivalents (1.1→1.3 equiv, entry 3). We also found that in some cases, a small increase in the reaction temperature to 40 °C was needed to ensure complete conversion into the allylated products (entries 4 and 5), and for consistency, these conditions (entry 6) were taken forward into the substrate scoping phase of the work,^20^ which is summarized in Scheme 2.

**Table 1:** General reaction conditions optimization. 
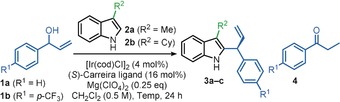

Entry	**1**	**2** (eq.)	Temp.	**3**		**1** [%]	**4** [%]
				%	*ee*		
1	**a**	**a** (1.1)	RT	99	98	0	0
2	**a**	**b** (1.1)	RT	63	99	0	19
3	**a**	**b** (1.3)	RT	96	>99	0	0
4	**b**	**a** (1.1)	RT	69	98	29	0
5	**b**	**a** (1.1)	40 °C	99	96	0	0
6	**a**	**a** (1.3)	40 °C	97	97	0	0

Substituents were tolerated at all positions around the phenyl ring of the allylic alcohol partner (Scheme 2) including electron‐deficient (di‐nitro **3 j**) and electron‐rich (trimethoxy **3 k**) aromatics. Enantioselectivity was universally high and the branched isomer product was formed exclusively in all of these examples. It was also possible to use heteroaryl allylic alcohols (**3 m**–**3 o**), although there was some erosion of the linear:branched regioselectivity when using indole allylic alcohol (**3 o**).

A wide variety of substituents were well tolerated at the C3‐position of indole including alkyl, aryl, benzyl and allyl as well as halide functionality^21^ at the C3‐, C5‐ and C6‐positions of indole, all providing their corresponding C2‐allylated products in excellent yields and enantioselectivities. The lower yield obtained for 3‐phenyl‐substituted allylated product **3 p** was due to the competitive formation of propiophenone side‐product **4**; the extent of side‐product formation was reduced as steric bulk decreased at the C3‐position (phenyl → benzyl → allyl) and could also be suppressed by using increased indole equivalents (see earlier optimization). Alkyl tethers incorporating a free alcohol (**3 w**), a protected alcohol (**3 x**) and amine functionality (**3 y**–**3 aa**) at the C3‐position of indole were all well tolerated. *N*‐Methyl indole was a suitable substrate, furnishing C2‐allylated product (**3 ab**) in 72 % yield and 84 % *ee*. Indole **3 ac** was also formed from a trimethoxybenzene‐based allylic alcohol in good yield and excellent enantioselectivity; its structural assignment is supported by X‐ray crystallographic data, from which the absolute stereochemistry of all other substrates was assigned by analogy.^19^ It should be noted that when carbonyl groups were directly attached to the C3‐position of indole, C2‐allylation was unsuccessful,^22^ which is not altogether surprising, given that the nucleophilicity of indole is significantly reduced upon substitution with such electron‐withdrawing groups.

**Scheme 3:**
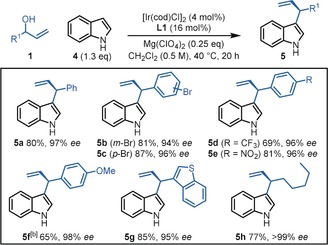
Substrate scope for C3‐allylation.^[a]^ [a] Yields of isolated products reported. Enantiomeric excess (*ee*) values were determined by HPLC analysis with a chiral stationary phase. [b] 0.25 equiv Zn(OTf)_2_ used instead of Mg(ClO_4_)_2_ and reaction performed at RT.

Predictably, a C3‐substituent is required on the indole to achieve selective C2‐allylation, and in the absence of this substituent, allylation takes places exclusively at the C3‐position; for example, the formation of products **5 a**–**h** from indole **4** (Scheme 3). Notably, these products were all formed in good to excellent yields and excellent enantioselectivities,^23^, ^24^ reflecting the mild nature of our reaction conditions relative to previous methods.3a–3c Indeed, this represents the most enantioselective method for the synthesis of these fundamental scaffolds to date.

### Mechanistic Studies

Next, a series of control reactions were conducted to help elucidate the roles of each reaction component (Table 2). Product **3 a** was not formed in the absence of Lewis acid (entry 2), clearly demonstrating its necessity for reactivity. Furthermore, the nature of the Lewis acid, both the cation and anion, plays a key role in C2‐ vs. N1‐selectivity, as well as the enantioselectivity of the reaction (see Lewis acid optimization screen in SI). In the absence of the iridium catalyst (entry 3) or the ligand (entry 4) a minor amount of product was formed, most probably via Lewis acidic activation of the allylic alcohol, as no asymmetric induction was observed.

**Table 2:** Control experiments. 
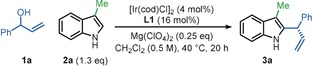

Entry	Deviation from standard conditions	Yield [%]	*ee* [%]
			
1	none	97	97
2	no Mg(ClO_4_)_2_	0	–
3	no [Ir(cod)Cl]_2_	18	0
4	no **L1**	18	0

**Scheme 4:**
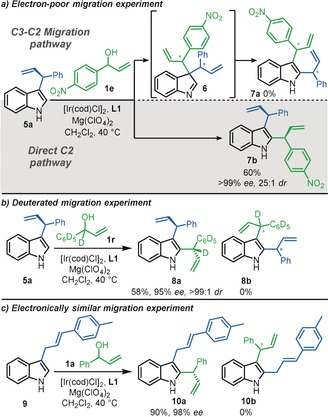
Mechanistic investigations^[a,b]^ [a] Reaction conditions: **5 a** or **9** (0.52 mmol), **1 a**, **1 e** or **1 r** (0.40 mmol), [Ir(cod)Cl]_2_ (4 mol %), **L1** (16 mol %), Mg(ClO_4_)_2_ (0.10 mmol) in CH_2_Cl_2_ (2.0 mL) at 40 °C, 20 h. [b] ^1^H NMR yields of products reported based on a trimethoxybenzene internal standard. Enantiomeric excess (*ee*) values were determined by HPLC analysis with a chiral stationary phase.

Next, to help understand whether the reaction proceeds via direct C2‐allylation or initial C3‐allylation followed by migration, a series of selective migration experiments were designed. First, C3‐allylated indole **5 a** was reacted with electron‐poor, *p*‐nitrophenyl allylic alcohol **1 e** (Scheme 4, a). The idea was that if the reaction proceeded via initial C3‐allylation, dearomatized intermediate **6** would first form, which would presumably be followed by migration of the more electron‐rich allyl substituent, in view of its higher migratory aptitude, thus furnishing C2‐allylated product **7 a**. However, if the reaction progressed via direct C2‐attack, C2‐allylated product **7 b** would instead be formed. The outcome of this reaction was clear; the sole isolation of indole **7 b** 25 (confirmed using NOE experiments), provided strong support that, in this case, the reaction proceeded via the direct C2‐pathway. Selectivity between electronically similar substituents was next investigated (Scheme 4, b), via the reaction of C3‐allylated indole **5 a** with deuterated allylic alcohol **1 r**. Due to the very similar electronic nature of the two allyl groups, a mixture of products was expected if the reaction proceeded via C3–C2 migration, but in fact, bis‐allylated product **8 a** was selectively formed (confirmed by NOE experiments), indicating again that the reaction proceeded via the direct C2‐pathway. The same direct C2‐allylation pathway was also observed in the reaction of linear C3‐allylated indole **9** with phenyl allylic alcohol **1 a** (Scheme 4, c).

**Figure 1:**
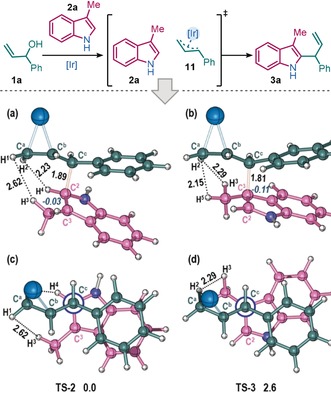
Optimized structures of **TS‐2** and **TS‐3** and their calculated relative Gibbs free energy (in kcal mol^−1^). (a) and (b) side views; (c) and (d) Newman projections along the forming C−C bond. The ligands associated to the Ir center are omitted for clarity.

With experimental evidence supporting a direct C2‐allylation pathway obtained, we then turned to DFT to gain a deeper understanding of the reaction mechanism (Figure 1). All possible transition states for intermolecular C2‐ or C3‐allylation between indole **2 a** and π‐allyl complex **11** were calculated. The most stable transition states for direct C2‐attack (**TS‐2**, 0.0 kcal mol^−1^) and direct C3‐attack (**TS‐3**, 2.6 kcal mol^−1^) are represented in Figure 1. The lower energy of **TS‐2** suggests a direct C2‐allylation pathway.

The innate electronic property of indole favors C3‐attack, which is exhibited by the more negative NPA (natural population analysis) charge at C3 of **TS‐3** (−0.11) compared with that at C2 of **TS‐2** (−0.03). This property is supported by the direct C3‐allylation of indoles not possessing a C3‐substituent (Scheme 3). However, the preference for direct C2‐attack, by virtue of the more stabilized **TS‐2** over **TS‐3**, stems from the compromise of several competitive effects: (1) the C3‐methyl substituent causes unfavorable steric congestion due to the close non‐bonding hydrogen atom pairs in **TS‐3** [B(H2⋅⋅⋅H5)=2.15 Å and B(H2⋅⋅⋅H3)=2.29 Å] and (2) **TS‐2** enables superior π–π stacking between the electron‐rich indole ring and the phenyl group of the electrophilic cinnamyl moiety, both of which contribute to the lower barrier of the direct C2‐allylation pathway (see the Supporting Information for more details).^26^

**Scheme 5:**
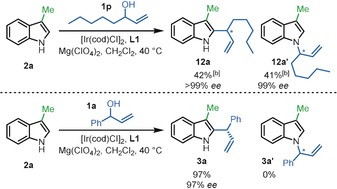
Aliphatic allylic alcohol experiment^[a]^ [a] Reaction conditions: **2 a** (0.52 mmol), **1 a** or **1 p** (0.40 mmol), [Ir(cod)Cl]_2_ (4 mol %), **L1** (16 mol %), Mg(ClO_4_)_2_ (0.10 mmol) in CH_2_Cl_2_ (2.0 mL) at 40 °C, 20 h. [b] ^1^H NMR yields reported based on trimethoxybenzene internal standard. Enantiomeric excess (*ee*) values were determined by HPLC analysis with chiral stationary phase.

Finally, to examine how π‐stacking, identified in the above DFT studies (illustrated in Figure 1 a,c), influences the regioselectivity of the reaction experimentally, alkyl allylic alcohol **1 p**, which is unable to undergo aryl π‐stacking, was reacted under the standard conditions. The reaction afforded a near equal mixture of the C2‐product **12 a** and N1‐product **12 a′** (42 % and 41 % yield, respectively, Scheme 5) suggesting that the aryl π‐stacking plays a key role in controlling the C2‐regioselectivity when reacting aryl allylic alcohols. Both products were formed with high enantioselectivity.^27^ Thus, at present we are only able to achieve high levels of C2‐regioselectivity using aryl substituted allylic alcohols. Nonetheless, we were pleased to observe that allylic substitution is still possible under our usual reaction conditions with aliphatic allylic alcohol **1 p**, given that π‐allyl complex formation is known to be more challenging for such alcohols compared with the more activated benzylic systems that are the main focus of this study.^28^

**Scheme 6:**
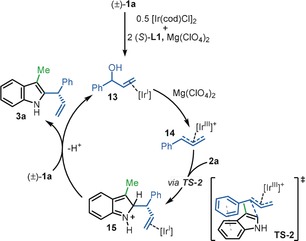
Proposed mechanism.

On the basis of the experimental data described above, in addition to previously described theoretical evidence,^29^ the following mechanism for the enantioselective direct C2‐allylation of indole is proposed (Scheme 6). The catalyst precursor [Ir(cod)Cl]_2_ coordinates to (*S*)‐**L1** and the racemic allylic alcohol [i.e., (±)‐**1 a**] to generate complex **13**. In the presence of Mg(ClO_4_)_2_, the carbon–oxygen bond of the secondary alcohol undergoes heterolytic cleavage which results in the formation of π‐allyl intermediate **14**. Nucleophilic attack on intermediate **14** by the indole (i.e., **2 a**) proceeds via transition state **TS‐2**, which is stabilized via the π‐π stacking interaction between the electron‐rich indole ring and the aryl group of the π‐allyl intermediate. Rearomatization of intermediate **15** and allylic alcohol exchange occurs to release the product (i.e., **3 a**) and regenerate active Ir^I^ complex **13**.^30^

## Conclusion

In summary, a highly enantioselective, directing group‐free intermolecular C2‐allylation procedure has been demonstrated for the first time, furnishing a wide range of C2‐allylated products in excellent yields with high regiocontrol. A combination of experimental migration studies and DFT calculations suggest the reaction proceeds via direct C2‐attack rather than C3‐allylation followed by in situ migration; this mode of reaction results in greater reaction predictability than might be expected via a C3–C2 migration pathway, in which isomeric products could form as a result of unselective migration. This unprecedented C2‐selectivity was achieved due to the combination of several factors: (1) steric congestion at the C3‐position of indole increases the relative reactivity of the C2‐ and N1‐positions; (2) π–π stacking interactions between the electron‐rich indole ring and the aryl group of the π‐allyl intermediate increases selectivity for C2‐allylation over N1‐allylation; (3) a suitable Lewis acid was identified able to activate the allylic alcohol and influence the N1 vs. C2 selectivity, without compromising enantioselectivity. The nature of both the cationic and anionic components of the Lewis acid were shown to be crucial for high selectivity and enantioselectivity. During the investigation, indole substrates without a C3‐substitutent were also explored using the optimized allylic substitution conditions affording C3‐allylated indoles in excellent enantioselectivities, further showcasing the mildness and broad suitability of the identified reaction conditions.

### General Procedure

To an oven‐dried Schlenk tube charged with a magnetic stirrer bar was added [Ir(cod)Cl]_2_ (0.016 mmol, 0.04 equiv) and (*S*)‐Carreira's Ligand **L1** (0.064 mmol, 0.16 equiv). The reaction vessel was purged by alternating vacuum and argon three times before dry CH_2_Cl_2_ (2 mL) was added. This mixture was stirred at RT for 15 min to form the active catalyst during which the solution turns from yellow to a deep red colour. Allylic alcohol (0.400 mmol, 1.0 equiv) was then added followed by the addition of indole derivative (0.520 mmol, 1.3 equiv) and Mg(ClO_4_)_2_ (0.100 mmol, 0.25 equiv) under a back pressure of argon. The reaction mixture was then heated to reflux and stirred for 15 h. The reaction mixture was directly concentrated on to silica and purified by column chromatography affording the desired allylated product.

## Conflict of interest

The authors declare no conflict of interest.

## Supporting information

As a service to our authors and readers, this journal provides supporting information supplied by the authors. Such materials are peer reviewed and may be re‐organized for online delivery, but are not copy‐edited or typeset. Technical support issues arising from supporting information (other than missing files) should be addressed to the authors.

Supplementary
