# Supplementary material for: Iridium‐Catalyzed Enantioselective Intermolecular Indole C2‐Allylation
Source: Angew Chem Int Ed Engl. 2020 Mar 11;59(19):7598–604. doi: 10.1002/anie.202001956 (PMC7217203; doi:10.1002/anie.202001956)
Supplement: Supplementary file 1 — Supplementary [file ANIE-59-7598-s001.pdf]

Angewandte Chemie
Eine Zeitschrift der Gesellschaft Deutscher Chemiker

Supporting Information

Iridium-Catalyzed Enantioselective Intermolecular Indole C2-Allylation

James A. Rossi-Ashton[+], Aimee K. Clarke[+], James R. Donald, Chao Zheng, Richard J. K. Taylor,* William P. Unsworth,* and Shu-Li You**

anie_202001956_sm_miscellaneous_information.pdf

Table of Contents

Table of Contents	Page
General information	3
List of starting materials	4
General procedures	5
Full optimization table	6
Compound characterization data and procedures	7–31
Kinetic isotope effect experiments and results	32
^{1}H and ^{13}C NMR spectra	33–78
HPLC chromatograms	79–120
X-ray crystal images	121–122
NOE spectra	123–125
Computational methods	126
The origin of regio- and enantioselectivity	126–129
Cartesian coordinates and energies	130–200
References	201–202

General Information

Except where stated, all reagents were purchased from commercial sources and used without further purification. Anhydrous CH_2Cl_2, toluene, acetonitrile and DMF were obtained from an Innovative Technology Inc. PureSolv® solvent purification system. Anhydrous THF was obtained by distillation over sodium benzophenone ketyl immediately before use. ^{1}H NMR and ^{13}C NMR spectra were recorded on a JEOL ECX400 or JEOL ECS400 spectrometer, operating at 400 MHz and 100 MHz. All spectral data was acquired at 295 K unless stated otherwise. Chemical shifts (δ) are quoted in parts per million (ppm). The residual solvent peaks, δ_H 7.26 and δ_c 77.16 for $CDCl_3$ were used as a reference. Coupling constants (*J*) are reported in Hertz (Hz) to the nearest 0.1 Hz. The multiplicity abbreviations used are: br s broad singlet, s singlet, d doublet, t triplet, q quartet, dt doublet of triplets, m multiplet. Signal assignment was achieved by analysis of DEPT, COSY, HMBC and HSQC experiments where required. Infrared (IR) spectra were recorded on a PerkinElmer UATR 2 spectrometer as a thin film dispersed from either CH_2Cl_2 or $CDCl_3$. Mass spectra (high-resolution) were obtained by the University of York Mass Spectrometry Service, using Electrospray Ionisation (ESI) on a Bruker Daltonics, Micro-tof spectrometer. Melting points were determined using Gallenkamp apparatus. Optical rotations were recorded at room temperature on a Bellingham + Stanley ADP450 series polarimeter. Chiral stationary phase HPLC was performed on an Agilent 1200 series chromatograph using a flow rate of 1.0 mL/min. Thin layer chromatography was carried out on Merck silica gel $60F_{254}$ pre-coated aluminium foil sheets and were visualised using UV light (254 nm) and stained with basic aqueous potassium permanganate. Flash column chromatography was carried out using slurry packed Fluka silica gel (SiO_2), 35–70 μm, 60 Å, under a light positive pressure, eluting with the specified solvent system.

Allylic alcohols were prepared by the reaction of the corresponding aldehyde with vinyl magnesium bromide.[1] Analytical data were in accordance with previously reported values.

List of starting materials

All the starting materials used in this publication are listed below. Commercially available starting materials (denoted with a *) were used as supplied, those with a reference number are known compounds prepared via the cited literature method, while for all others, preparative details and spectroscopic characterization data are provided.

Allylic alcohols

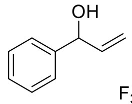
1a[2]

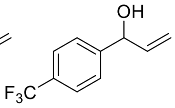
1b[2]

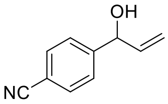
1c[3]

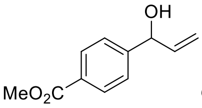
1d[2]

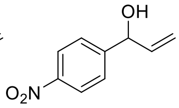
1e[2]

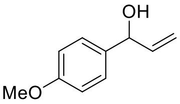
1f[4]

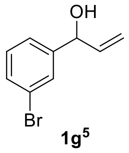
1g[5]

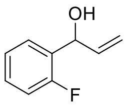
1h[4]

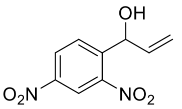
1i

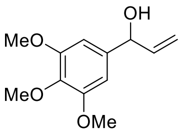
1j[3]

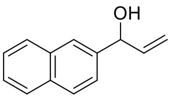
1k[2]

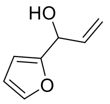
1l[3]

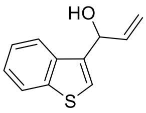
1m

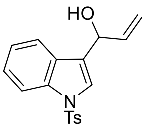
1n[6]

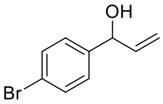
1o[4]

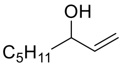
1p*

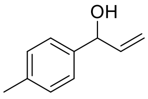
1q[3]

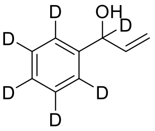
1r

3-Substituted indoles

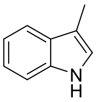
2a*

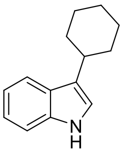
2b[7]

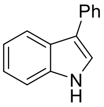
2c[8]

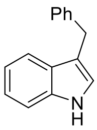
2d[9]

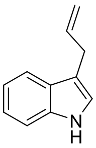
2e[10]

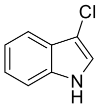
2f[11]

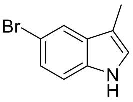
2g[12]

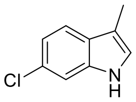
2h[13]

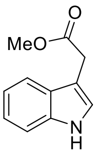
2i*

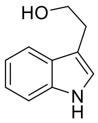
2j*

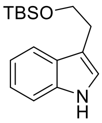
2k[14]

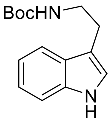
2l*

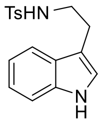
2m[15]

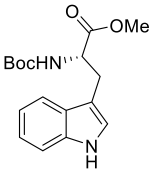
2n*

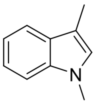
2o[16]

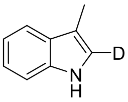
2a-*d*[17]

General procedures

General procedure A – Allylic Alcohol Synthesis

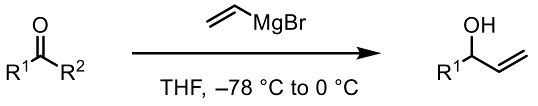

To a solution of aldehyde (10.0 mmol) in THF (20 mL) under argon was added vinyl magnesium bromide (11 mmol, 1.0 M in THF) dropwise at −78 °C. This reaction mixture was then stirred for 1 h at −78 °C then warmed to 0 °C and stirred until complete reaction was observed by TLC. The reaction was quenched by the addition of sat. aq. NH_4Cl (20 mL). The organics were separated and the aqueous layer extracted with Et_2O (3 x 20 mL). The organics were combined, washed with brine (20 mL), dried over $MgSO_4$, concentrated *in vacuo* and purified by column chromatography to afford the allylic alcohol product.

General procedure B – C2-Allylation Procedure

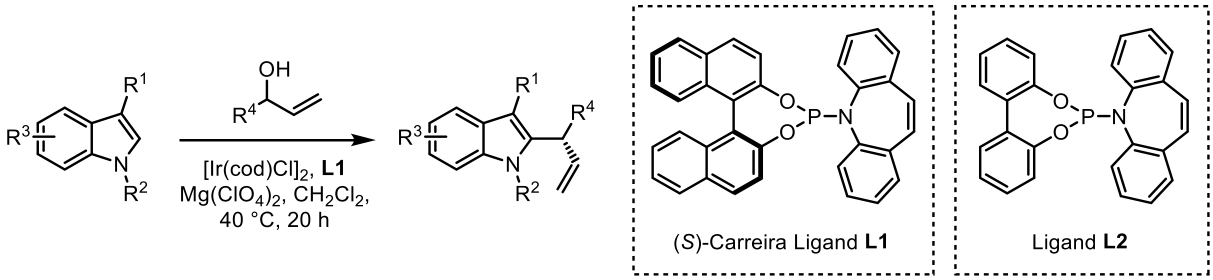

To an oven-dried Schlenk tube charged with a magnetic stirrer bar was added $[Ir(cod)Cl]_2$ (0.016 mmol, 0.04 equiv.) and (*S*)-Carreira's Ligand **L1** (0.064 mmol, 0.16 equiv.). The reaction vessel was purged by alternating vacuum and argon three times before dry CH_2Cl_2 (2 mL) was added. This mixture was stirred at RT for 15 min to form the active catalyst during which the solution turns from yellow to a deep red colour. Allylic alcohol (0.400 mmol, 1.0 equiv.) was then added followed by the addition of indole derivative (0.520 mmol, 1.3 equiv.) and $Mg(ClO_4)_2$ (0.100 mmol, 0.25 equiv.) under a back pressure of argon. The reaction mixture was then heated to reflux and stirred for 15 h. The reaction mixture was directly concentrated on to silica and purified by column chromatography affording the desired allylated product.

Racemic products were generated using the same procedure as described above using the ligand **L2**.

Table S1. C2-allylation optimization table

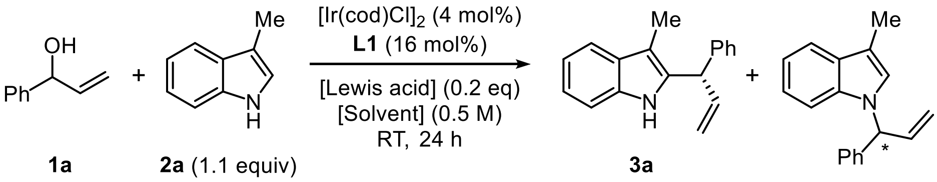

1a **2a** (1.1 equiv) **3a** **3a'**

Entry	Lewis Acid	Solvent	Yield /%			**3a** *ee* /%	Comments
			**3a**	**3a'**	**1a**		
1	$ZrCl_4$	CH_2Cl_2	21	35	0	>99	
2	AgOTf	CH_2Cl_2	45	15	0	61	
3	$Sn(OTf)_2$	CH_2Cl_2	52	0	0	84	
4	$BF_3{\cdot}OEt_2$	CH_2Cl_2	59	0	0	13	
5	$Eu(OTf)_3$	CH_2Cl_2	59	trace	24	>99	
6	$Yb(OTf)_3$	CH_2Cl_2	75	trace	7	95	
7	$Zn(OTf)_3$	CH_2Cl_2	72	9	trace	>99	
8	$InBr_3$	CH_2Cl_2	79	7	0	98	
9	$Y(OTf)_3$	CH_2Cl_2	79	0	15	99	
10	$Sc(OTf)_3$	CH_2Cl_2	81	0	0	92	
11	$Fe(OTf)_3$	CH_2Cl_2	90	0	0	96	
12	$Fe(OTf)_3$	DCE	88	0	3	96	
13	$Fe(OTf)_3$	$CHCl_3$	95	0	0	91	
14	$Fe(OTf)_3$	Toluene	71	3	13	77	
15	$Fe(OTf)_3$	THF	59	5	3	96	
16	$Fe(OTf)_3$	Et_2O	76	5	7	81	
17	$Fe(OTf)_3$	EA	54	3	23	85	
18	$Fe(OTf)_3$	Acetone	72	7	0	94	
19	$Fe(OTf)_3$	Dioxane	68	5	13	87	
20	$Mg(ClO_4)_2$	CH_2Cl_2	99	0	0	98	
21	$Mg(ClO_4)_2$	CH_2Cl_2	99	0	0	98	0.25 eq LA, 1.3 eq **2a**
22	$Mg(ClO_4)_2$	CH_2Cl_2	97	0	0	97	0.25 eq LA, 1.3 eq **2a**, 40 °C
23	$Mg(OTf)_2$	CH_2Cl_2	8	0	83	nd	
24	$MgCl_2$	CH_2Cl_2	20	24	43	nd	
25	MgI_2	CH_2Cl_2	37	6	41	nd	

Compound characterisation data and procedures

(*S*)-3-Methyl-2-(1-phenylallyl)-1*H*-indole (3a)

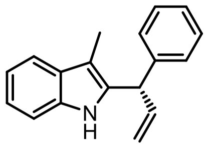

Prepared according to general procedure B using $[Ir(cod)Cl]_2$ (10.7 mg, 0.016 mmol), (*S*)-Carreira's Ligand **L1** (32.5 mg, 0.064 mmol), 1-phenylprop-2-en-1-ol **1a** (53.7 mg, 0.400 mmol), 3-methyl-1*H*-indole **2a** (68.2 mg, 0.52 mmol) and $Mg(ClO_4)_2$ (22.3 mg, 0.100 mmol) in dry CH_2Cl_2 (2 mL). Purification by flash chromatography on silica gel (8:2 hexane:Et_2O) afforded the title compound **3a** as a clear and colourless oil (96.0 mg, 97% yield, 97% *ee*); R_f 0.50 (7:3 hexane:Et_2O:); ν_{max} (thin film)/cm^{-1} 3407, 3057, 2918, 2857, 1637, 1491, 1460, 1450, 1260, 1096, 922, 741, 700; δ_H (400 MHz, $CDCl_3$) 7.60 (br s, 1H), 7.57–7.53 (m, 1H), 7.38–7.31 (m, 2H), 7.30–7.23 (m, 4H), 7.17–7.08 (m, 2H), 6.29 (ddd, *J* = 16.8, 10.2, 6.4 Hz, 1H), 5.32 (app. dt, *J* = 10.2, 1.4 Hz, 1H), 5.11–5.00 (m, 2H), 2.26 (s, 3H); δ_C (100 MHz, $CDCl_3$) 141.2, 138.0, 135.3, 134.3, 129.5, 128.9, 128.5, 127.0, 121.4, 119.2, 118.5, 117.6, 110.6, 108.2, 46.4, 8.7; HRMS (APCI$^+$): Found: 284.142486; $C_{18}H_{18}N^+$ (MH^+) Requires 248.143376 (3.6 ppm error); $[\alpha]_D^{20}$ −13.01 (c = 0.17, $CHCl_3$).

(*S*)-3-Cyclohexyl-2-(1-phenylallyl)-1*H*-indole (3b)

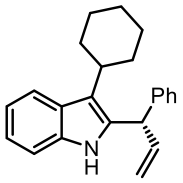

Prepared according to general procedure B using $[Ir(cod)Cl]_2$ (10.7 mg, 0.016 mmol), (*S*)-Carreira's Ligand **L1** (32.5 mg, 0.064 mmol), 1-phenylprop-2-en-1-ol **1a** (53.7 mg, 0.400 mmol), 3-cyclohexyl-1*H*-indole **2b** (104 mg, 0.520 mmol) and $Mg(ClO_4)_2$ (22.3 mg, 0.100 mmol) in dry CH_2Cl_2 (2 mL). Purification by flash chromatography on silica gel (1:1 CH_2Cl_2:hexane) afforded the title compound **3b** as a clear and colourless oil (119 mg, 94% yield, >99% *ee*); R_f 0.33 (7:3 hexane:toluene); ν_{max} (thin film)/cm^{-1} 3457, 3417, 2923, 2850, 1489, 1456, 1434, 1305, 921, 737; δ_H (400 MHz, $CDCl_3$) 7.76 (d, *J* = 7.7 Hz, 1H), 7.54 (br s, 1H), 7.36–7.30 (m, 2H), 7.28–7.21 (m, 4H), 7.13–7.01 (m, 2H), 6.30 (ddd, *J* = 17.1, 10.2, 6.1 Hz, 1H), 5.31 (dd, *J* = 10.2, 1.5 Hz, 1H), 5.14 (br d, *J* = 5.8 Hz, 1H), 5.03 (dd, *J* = 17.1, 1.5 Hz, 1H), 2.84–2.71 (m, 1H), 2.03–1.63 (m, 7H), 1.40–1.27 (m, 3H); δ_C (100 MHz, $CDCl_3$) 141.6, 138.5, 135.8, 133.3, 128.8, 128.4, 127.6, 127.0, 121.1, 120.5, 118.8, 118.4, 117.7, 110.9, 46.2, 36.7, 33.2, 33.1, 27.5 (2 x C), 26.5; HRMS (ESI$^+$): Found: 316.2057; $C_{23}H_{26}N^+$ (MH^+) Requires 316.2060 (0.8 ppm error); $[\alpha]_D^{20}$ –24.5 (c = 1.25, $CHCl_3$).

(*S*)-3-Methyl-2-(1-(4-(trifluoromethyl)phenyl)allyl)-1*H*-indole (3c)

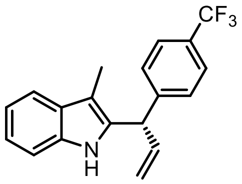

Prepared according to general procedure B using $[Ir(cod)Cl]_2$ (10.7 mg, 0.016 mmol), (*S*)-Carreira's Ligand **L1** (32.5 mg, 0.064 mmol), 1-(4-(trifluoromethyl)phenyl)prop-2-en-1-ol **1b** (53.7 mg, 0.400 mmol), 3-methyl-1*H*-indole **2a** (68.2 mg, 0.520 mmol) and $Mg(ClO_4)_2$ (22.3 mg, 0.100 mmol) in dry CH_2Cl_2 (2 mL). Purification by flash chromatography on silica gel (1:1 CH_2Cl_2:hexane) afforded the title compound **3c** as a clear and colourless oil (125 mg, 99% yield, 96% *ee*); R_f 0.51 (1:1 CH_2Cl_2:hexane); ν_{max} (thin film)/cm^{-1} 3411, 2920, 1618, 1460, 1323, 1163, 1120, 1110, 1067, 1017, 741; δ_H (400 MHz, $CDCl_3$) 7.64 (br s, 1H), 7.61–7.53 (m, 3H), 7.34 (d, *J* = 8.0 Hz, 2H), 7.30–7.27 (m, 1H), 7.19–7.10 (m, 2H), 6.28 (ddd, *J* = 17.0, 10.2, 6.4 Hz, 1H), 5.38 (ddd, *J* = 10.2, 1.3, 1.3 Hz, 1H), 5.13 (br d, *J* = 6.4 Hz, 1H), 5.07 (ddd, J = 17.0, 1.3, 1.3 Hz, 1H), 2.22 (s, 3H); δ_C (100 MHz, $CDCl_3$) 145.4, 137.3, 135.4, 133.3, 129.5, 129.3 (q, $^2J_{CF}$ = 32 Hz), 128.8, 125.7 (q, $^3J_{CF}$ = 4 Hz), 124.3 (q, $^1J_{CF}$ = 272 Hz), 121.8, 119.5, 118.7, 118.5, 110.8, 108.8, 46.3, 8.7; HRMS (APCI$^+$): Found: 316.129874; $C_{19}H_{17}F_3N^+$ (MH^+) Requires 316.130761 (2.8 ppm error); $[\alpha]_D^{20}$ +4.16 (c = 0.25, $CHCl_3$).

(*S*)-4-(1-(3-Methyl-1*H*-indol-2-yl)allyl)benzonitrile (3d)

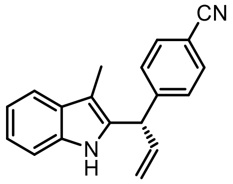

Prepared according to general procedure B using $[Ir(cod)Cl]_2$ (10.7 mg, 0.016 mmol), (*S*)-Carreira's Ligand **L1** (32.5 mg, 0.064 mmol), 4-(1-hydroxyallyl)benzonitrile **1c** (63.7 mg, 0.400 mmol), 3-methyl-1*H*-indole **2a** (68.2 mg, 0.520 mmol) and $Mg(ClO_4)_2$ (22.3 mg, 0.100 mmol) in dry CH_2Cl_2 (2 mL). Purification by flash chromatography on silica gel (6:4 CH_2Cl_2:hexane then 3:1 CH_2Cl_2:hexane) afforded the title compound **3d** as a yellow oil (75.0 mg, 69% yield, 93% *ee*); R_f 0.53 (100% CH_2Cl_2); ν_{max} (thin film)/cm^{-1} 3397, 2918, 2228, 1605, 1459, 1333, 926, 844, 744; δ_H (400 MHz, $CDCl_3$) 7.78 (br s, 1H), 7.64–7.55 (m, 3H), 7.38–7.30 (m, 3H), 7.23–7.12 (m, 2H), 6.29 (ddd, *J* = 16.9, 10.2, 6.5 Hz, 1H), 5.47–5.39 (m, 1H), 5.17–5.05 (m, 2H), 2.23 (s, 3H); δ_C (100 MHz, $CDCl_3$) 146.8, 136.8, 135.5, 132.7, 132.5, 129.3, 129.2, 121.9, 119.5, 118.9, 118.8, 118.6, 110.8, 110.7, 108.8, 46.4, 8.7; HRMS (APCI$^+$): Found: 273.137728; $C_{19}H_{17}N_2^+$ (MH^+) Requires 273.138625 (−3.3 ppm error); $[\alpha]_D^{20}$ +18.31 (c = 0.45, $CHCl_3$).

Methyl (*S*)-4-(1-(3-methyl-1*H*-indol-2-yl)allyl)benzoate (3e)

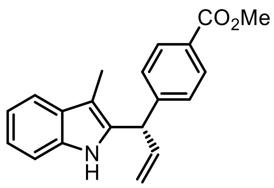

Prepared according to general procedure B using $[Ir(cod)Cl]_2$ (10.7 mg, 0.016 mmol), (*S*)-Carreira's Ligand **L1** (32.5 mg, 0.064 mmol), methyl 4-(1-hydroxyallyl)benzoate **1d** (76.9 mg, 0.400 mmol), 3-methyl-1*H*-indole **2a** (68.2 mg, 0.520 mmol) and $Mg(ClO_4)_2$ (22.3 mg, 0.100 mmol) in dry CH_2Cl_2 (2 mL). Purification by flash chromatography on silica gel (7:3 Et_2O:hexane) afforded the title compound **3e** as a clear and colourless oil (108 mg, 88% yield, 98% *ee*); R_f 0.34 (8:2 CH_2Cl_2:hexane); ν_{max} (thin film)/cm^{-1} 3389, 2951, 1709, 1609, 1459, 1436, 1281, 1180, 1113, 1019, 742; δ_H (400 MHz, $CDCl_3$) 8.06–8.00 (m, 2H), 7.84 (br s, 1H), 7.64–7.57 (m, 1H), 7.39–7.29 (m, 3H), 7.23–7.13 (m, 2H), 6.32 (ddd, *J* = 16.9, 10.2, 6.5 Hz, 1H), 5.46–5.35 (m, 1H), 5.20–5.05 (m, 2H), 3.95 (s, 3H), 2.28 (s, 3H); δ_C (100 MHz, $CDCl_3$) 167.0, 146.6, 137.3, 135.4, 133.5, 130.0, 129.4, 128.8, 128.4, 121.6, 119.3, 118.5, 118.2, 110.7, 108.5, 52.2, 46.5, 8.7; HRMS (ESI$^+$): Found: 328.1300; $C_{20}H_{19}NNaO_2^+$ (MNa^+) Requires 328.1308 (2.6 ppm error); $[\alpha]_D^{20}$ −11.41 (c = 0.97, $CHCl_3$).

(*S*)-3-Methyl-2-(1-(4-nitrophenyl)allyl)-1*H*-indole (3f)

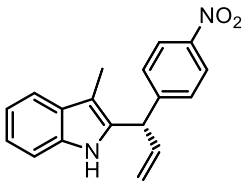

Prepared according to general procedure B using $[Ir(cod)Cl]_2$ (10.7 mg, 0.016 mmol), (*S*)-Carreira's Ligand **L1** (32.5 mg, 0.064 mmol), 1-(4-nitrophenyl)prop-2-en-1-ol **1e** (71.7 mg, 0.400 mmol), 3-methyl-1*H*-indole **2a** (68.2 mg, 0.520 mmol) and $Mg(ClO_4)_2$ (22.3 mg, 0.100 mmol) in dry CH_2Cl_2 (2 mL). Purification by flash chromatography on silica gel (1:1 CH_2Cl_2:hexane) afforded the title compound **3f** as a yellow oil (106 mg, 91% yield, 96% *ee*); R_f 0.17 (4:6 CH_2Cl_2:hexane); ν_{max} (thin film)/cm^{-1} 3412, 2922, 2856, 1596, 1516, 1459, 1345, 1109, 851, 743; δ_H (400 MHz, $CDCl_3$) 8.20–8.15 (m, 2H), 7.73 (br s, 1H), 7.57 (d, *J* = 7.7 Hz, 1H), 7.42–7.35 (m, 2H), 7.34–7.29 (m, 1H), 7.23–7.10 (m, 2H), 6.37–6.25 (m, 1H), 5.43 (d, *J* = 10.2 Hz, 1H), 5.17 (d, *J* = 6.4 Hz, 1H), 5.10 (d, *J* = 17.2 Hz, 1H), 2.22 (s, 3H); δ_C (100 MHz, $CDCl_3$) 148.9, 147.0, 136.8, 135.5, 132.6, 129.4, 129.3, 124.0, 122.0, 119.6, 119.1, 118.7, 110.8, 109.0, 46.3, 8.7; HRMS (APCI$^+$): Found: 293.128783; $C_{18}H_{17}N_2O_2^+$ (MH^+) Requires 293.128454 (1.1 ppm error); $[\alpha]_D^{20}$ +20.78 (c = 0.35, $CHCl_3$).

(*S*)-2-(1-(4-Methoxyphenyl)allyl)-3-methyl-1*H*-indole (3g)

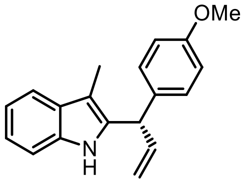

Prepared according to general procedure B using $[Ir(cod)Cl]_2$ (10.7 mg, 0.016 mmol), (*S*)-Carreira's Ligand **L1** (32.5 mg, 0.064 mmol), 1-(4-methoxyphenyl)prop-2-en-1-ol **1f** (65.7 mg, 0.400 mmol), 3-methyl-1*H*-indole **2a** (68.2 mg, 0.520 mmol) and $Mg(ClO_4)_2$ (22.3 mg, 0.100 mmol) in dry CH_2Cl_2 (2 mL). Purification by flash chromatography on silica gel (8:2 hexane:Et_2O) afforded the title compound **3g** as a clear and colourless oil (90.1 mg, 81% yield, 99% *ee*); R_f 0.39 (8:2 hexane:Et_2O); ν_{max} (thin film)/cm^{-1} 3409, 2919, 1609, 1509, 1460, 1300, 1246, 1177, 1033, 742; δ_H (400 MHz, $CDCl_3$) 7.59–7.51 (m, 2H), 7.25–7.22 (m, 1H), 7.18–7.07 (m, 4H), 6.90–6.84 (m, 2H), 6.31–6.20 (m, 1H), 5.32–5.25 (m, 1H), 5.07–4.97 (m, 2H), 3.80 (s, 3H), 2.23 (s, 3H); δ_C (100 MHz, $CDCl_3$) 158.6, 138.3, 135.3, 134.7, 133.2, 129.6, 129.5, 121.4, 119.2, 118.5, 117.2, 114.2, 110.6, 108.1, 55.4, 45.7, 8.7; HRMS (ESI$^+$): Found: 276.1380; $C_{19}H_{18}NO^+$ (MH^+) Requires 276.1383 (0.9 ppm error); $[\alpha]_D^{20}$ −19.27 (c = 0.35, $CHCl_3$).

(*S*)-2-(1-(3-Bromophenyl)allyl)-3-methyl-1*H*-indole (3h)

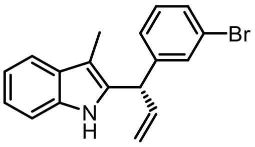

Prepared according to general procedure B using $[Ir(cod)Cl]_2$ (10.7 mg, 0.016 mmol), (*S*)-Carreira's Ligand **L1** (32.5 mg, 0.064 mmol), 1-(3-bromophenyl)prop-2-en-1-ol **1g** (85.2 mg, 0.400 mmol), 3-methyl-1*H*-indole **2a** (68.2 mg, 0.520 mmol) and $Mg(ClO_4)_2$ (22.3 mg, 0.100 mmol) in dry CH_2Cl_2 (2 mL). Purification by flash chromatography on silica gel (7:3 hexane:$CHCl_3$) afforded the title compound **3h** as a clear and colourless oil (124 mg, 95% yield, >99% *ee*); R_f 0.51 (8:2 hexane:Et_2O); ν_{max} (thin film)/cm^{-1} 3427, 3057, 2919, 1591, 1566, 1469, 1459, 1305, 1238, 924, 784, 742; δ_H (400 MHz, $CDCl_3$) 7.61 (br s, 1H), 7.58–7.52 (m, 1H), 7.42–7.38 (m, 2H), 7.29–7.25 (m, 1H), 7.22–7.08 (m, 4H), 6.25 (ddd, *J* = 16.7, 10.2, 6.6 Hz, 1H), 5.40–5.31 (m, 1H), 5.10–5.01 (m, 2H), 2.24 (s, 3H); δ_C (100 MHz, $CDCl_3$) 143.6, 137.4, 135.4, 133.4, 131.4, 130.4, 130.2, 129.4, 127.2, 123.0, 121.7, 119.4, 118.6, 118.3, 110.7, 108.6, 46.1, 8.7; HRMS (ESI$^+$): Found: 326.0537; $C_{18}H_{17}{}^{79}BrN^+$ (MH^+) Requires 326.0539 (0.7 ppm error); $[\alpha]_D^{20}$ −23.78 (c = 0.27, $CHCl_3$).

(*S*)-2-(1-(2-Fluorophenyl)allyl)-3-methyl-1*H*-indole (3i)

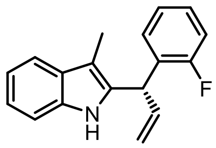

Prepared according to general procedure B using $[Ir(cod)Cl]_2$ (10.7 mg, 0.016 mmol), (*S*)-Carreira's Ligand **L1** (32.5 mg, 0.064 mmol), 1-(2-fluorophenyl)prop-2-en-1-ol **1h** (60.9 mg, 0.400 mmol), 3-methyl-1*H*-indole **2a** (68.2 mg, 0.520 mmol) and $Mg(ClO_4)_2$ (22.3 mg, 0.100 mmol) in dry CH_2Cl_2 (2 mL). Purification by flash chromatography on silica gel (1:1 $CHCl_3$:hexane) afforded the title compound **3i** as a yellow oil (92 mg, 87% yield, 83% *ee*); R_f 0.54 (1:1 CH_2Cl_2:hexane); ν_{max} (thin film)/cm^{-1} 3432, 3058, 2918, 1637, 1583, 1487, 1457, 1303, 1228, 1093, 925, 755, 741; δ_H (400 MHz, $CDCl_3$) 7.78 (br s, 1H), 7.56–7.50 (m, 1H), 7.30–7.22 (m, 2H), 7.21–7.03 (m, 5H), 6.34–6.24 (m, 1H), 5.38–5.32 (m, 2H), 5.06–4.98 (m, 1H), 2.23 (s, 3H); δ_C (100 MHz, $CDCl_3$) 160.6 (d, $^1J_{CF}$ = 245 Hz), 137, 135.3, 133.0, 129.9 (d, $^3J_{CF}$ = 4 Hz), 129.5, 128.8 (d, $^3J_{CF}$ = 8 Hz), 128.5 (d, $^2J_{CF}$ = 15 Hz), 124.5 (d, $^4J_{CF}$ = 4 Hz), 121.6, 119.3, 118.6, 117.7, 115.8 (d, $^2J_{CF}$ = 22 Hz), 110.7, 108.4, 40.0 (d, $^3J_{CF}$ = 3 Hz), 8.58; HRMS (ESI$^+$): Found: 266.1325; $C_{18}H_{17}FN^+$ (MH^+) Requires 266.1340 (5.6 ppm error); $[\alpha]_D^{20}$ +45.19 (c = 0.50, $CHCl_3$).

1-(2,4-Dinitrophenyl)prop-2-en-1-ol (1i)

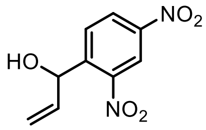

Prepared according to general procedure A using 2,4-dinitrobenzaldehyde (2.94 g, 15.0 mmol), vinyl magnesium bromide (16.5 mL, 16.5 mmol, 1.0 M. in THF), in dry THF (30 mL). Purification by flash chromatography on silica gel (95:05 toluene:Et_2O) afforded the title compound **1i** as a yellow oil (1.89 g, 56% yield); R_f 0.20 (95:05 toluene:Et_2O:); ν_{max} (thin film)/cm^{-1} 3543, 3109, 1606, 1527, 1343, 991, 911, 810, 727; δ_H (400 MHz, $CDCl_3$) 8.78 (d, *J* = 2.3 Hz, 1H), 8.48 (dd, *J* = 8.7, 2.3 Hz, 1H), 8.10 (d, *J* = 8.7 Hz, 1H), 6.03 (ddd, *J* = 16.9, 10.3, 5.7 Hz, 1H), 5.97–5.93 (m, 1H), 5.46 (app. dt, *J* = 16.9, 1.0 Hz, 1H), 5.32 (app. dt, *J* = 10.3, 1.0 Hz, 1H), 2.47 (d, *J* = 4.0 Hz, 1H); δ_C (100 MHz, $CDCl_3$) 148.0, 147.2, 144.3, 137.0, 130.3, 127.7, 120.2, 117.9, 69.8; HRMS (ESI$^-$): Found: 223.0354; $C_9H_7N_2O_5^-$ (MH^-) Requires 223.0360 (3.1 ppm error).

(*S*)-2-(1-(2,4-Dinitrophenyl)allyl)-3-methyl-1*H*-indole (3j)

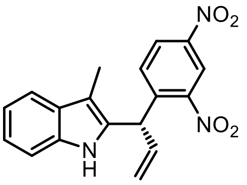

Prepared according to general procedure B using $[Ir(cod)Cl]_2$ (10.7 mg, 0.016 mmol), (*S*)-Carreira's Ligand **L1** (32.5 mg, 0.064 mmol), 1-(2,4-dinitrophenyl)prop-2-en-1-ol **1i** (89.7 mg, 0.400 mmol), 3-methyl-1*H*-indole **2a** (68.2 mg, 0.520 mmol) and $Mg(ClO_4)_2$ (22.3 mg, 0.100 mmol) in dry CH_2Cl_2 (2 mL). Purification by flash chromatography on silica gel (6:4 CH_2Cl_2:hexane) afforded the title compound **3j** as an orange oil (75.0 mg, 56% yield, >99% *ee*); R_f 0.44 (7:3 CH_2Cl_2:hexane); ν_{max} (thin film)/cm^{-1} 3426, 2919, 1604, 1529, 1458, 1346, 1305, 906, 835, 742; δ_H (400 MHz, $CDCl_3$) 8.75 (d, *J* = 2.2 Hz, 1H), 8.28 (dd, *J* = 8.6, 2.2 Hz, 1H), 7.90 (br s, 1H), 7.56 (d, *J* = 7.8 Hz, 1H), 7.41–7.32 (m, 2H), 7.22 (dd, *J* = 7.5, 7.5 Hz, 1H), 7.15 (dd, *J* = 7.5, 7.5 Hz, 1H), 6.32 (ddd, *J* = 17.0, 10.3, 6.2 Hz, 1H), 5.98 (d, *J* = 6.2 Hz, 1H), 5.45 (d, *J* = 10.3 Hz, 1H), 4.97 (d, *J* = 17.0 Hz, 1H), 2.15 (s, 3H); δ_C (100 MHz, $CDCl_3$) 149.1, 146.8, 142.7, 135.7, 135.5, 132.5, 130.3, 129.5, 127.0, 122.5, 120.5, 119.9, 119.3, 119.0, 110.0, 110.1, 41.8, 6.6; HRMS (ESI$^-$): Found: 336.0980; $C_{18}H_{14}N_3O_4^-$ (MH^-) Requires 336.0990 (3.0 ppm error); $[\alpha]_D^{20}$ +49.42 (c = 0.48, $CHCl_3$).

(*S*)-3-Methyl-2-(1-(3,4,5-trimethoxyphenyl)allyl)-1*H*-indole (3k)

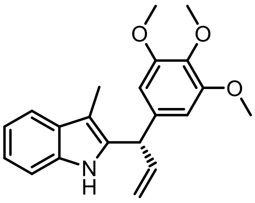

Prepared according to general procedure B using $[Ir(cod)Cl]_2$ (10.7 mg, 0.016 mmol), (*S*)-Carreira's Ligand **L1** (32.5 mg, 0.064 mmol), 1-(3,4,5-trimethoxyphenyl)prop-2-en-1-ol **1j** (97.7 mg, 0.400 mmol), 3-methyl-1*H*-indole **2a** (68.2 mg, 0.520 mmol) and $Mg(ClO_4)_2$ (22.3 mg, 0.100 mmol) in dry CH_2Cl_2 (2 mL). Purification by flash chromatography on silica gel (4:6 Et_2O:hexane) afforded the title compound **3k** as a white solid (133 mg, 99% yield, >99% *ee*); mp 120–122 °C; R_f 0.20 (4:6 Et_2O:hexane); ν_{max} (thin film)/cm^{-1} 3353, 2934, 1589, 1505, 1459, 1418, 1329, 1236, 1124, 1005, 831, 734; δ_H (400 MHz, $CDCl_3$) 7.60–7.51 (m, 2H), 7.28–7.24 (m, 1H), 7.16–7.08 (m, 2H), 6.49 (s, 2H), 6.23 (ddd, *J* = 17.0, 10.1, 6.3 Hz, 1H), 5.31 (ddd, *J* = 10.1, 1.5, 1.5 Hz, 1H), 5.05 (ddd, *J* = 17.0, 1.5, 1.5 Hz, 1H), 5.01–4.97 (m, 1H), 3.86 (s, 3H), 3.82 (s, 6H), 2.27 (s, 3H); δ_C (100 MHz, $CDCl_3$) 153.6, 137.7, 137.0, 136.8, 135.3, 134.1, 129.5, 121.5, 119.3, 118.5, 117.5, 110.7, 108.3, 105.5, 61.0, 56.3, 46.7, 8.8; HRMS (ESI$^+$): Found: 360.1570; $C_{21}H_{23}NNaO_3^+$ (MNa^+) Requires 360.1570 (0.1 ppm error); $[\alpha]_D^{20}$ −53.20 (c = 0.32, $CHCl_3$).

(*S*)-3-Methyl-2-(1-(naphthalen-2-yl)allyl)-1*H*-indole (3l)

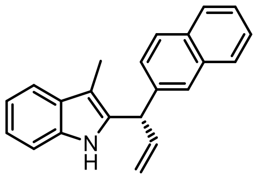

Prepared according to general procedure B using $[Ir(cod)Cl]_2$ (10.7 mg, 0.016 mmol), (*S*)-Carreira's Ligand **L1** (32.5 mg, 0.064 mmol), 1-(naphthalen-2-yl)prop-2-en-1-ol **1k** (73.7 mg, 0.400 mmol), 3-methyl-1*H*-indole **2a** (68.2 mg, 0.520 mmol) and $Mg(ClO_4)_2$ (22.3 mg, 0.100 mmol) in dry CH_2Cl_2 (2 mL). Purification by flash chromatography on silica gel (1:1 $CHCl_3$:hexane) afforded the title compound **3l** as an orange oil (109 mg, 91% yield, 97% *ee*); R_f 0.43 (8:2 hexane:Et_2O); ν_{max} (thin film)/cm^{-1} 3415, 3062, 2960, 2926, 2858, 1513, 1460, 1331, 1244, 823, 741; δ_H (400 MHz, $CDCl_3$) 7.85–7.77 (m, 3H), 7.70 (br s, 1H), 7.61 (br s, 1H), 7.57–7.53 (m, 1H), 7.51–7.45 (m, 2H), 7.35 (dd, *J* = 8.5, 1.8 Hz, 1H), 7.25–7.20 (m, 1H), 7.15–7.08 (m, 2H), 6.37 (ddd, *J* = 17.1, 10.2, 6.2 Hz, 1H), 5.37 (dd, *J* = 10.1, 1.3 Hz, 1H), 5.23 (d, *J* = 6.1 Hz, 1H), 5.08 (dd, *J* = 17.1, 1.3 Hz, 1H), 2.27 (s, 3H); δ_C (100 MHz, $CDCl_3$) 138.8, 137.9, 135.4, 134.2, 133.6, 132.6, 129.6, 128.5, 128.0, 127.8, 127.2, 126.6, 126.4, 126.0, 121.5, 119.3, 118.5, 117.8, 110.7, 108.4, 46.5, 8.8; HRMS (APCI$^+$): Found: 298.159017; $C_{22}H_{20}N^+$ (MH$^+$) Requires 298.159026 (0.0 ppm error); $[\alpha]_D^{20}$ −50.19 (c = 0.39, $CHCl_3$).

(*R*)-2-(1-(Furan-2-yl)allyl)-3-methyl-1*H*-indole (3m)

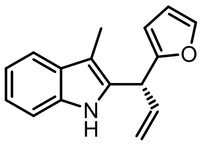

Prepared according to general procedure B using $[Ir(cod)Cl]_2$ (10.7 mg, 0.016 mmol), (*S*)-Carreira's Ligand **L1** (32.5 mg, 0.064 mmol), 1-(furan-2-yl)prop-2-en-1-ol **1l** (49.7 mg, 0.400 mmol), 3-methyl-1*H*-indole **2a** (68.2 mg, 0.520 mmol) and $Mg(ClO_4)_2$ (22.3 mg, 0.100 mmol) in dry CH_2Cl_2 (2 mL). Purification by flash chromatography on silica gel (8:2 hexane:Et_2O) afforded the title compound **3m** (51.2 mg, 53%, >99% *ee*) as clear and colourless oil; R_f 0.49 (8:2 hexane:Et_2O); ν_{max} (thin film)/cm^{-1} 3414, 2962, 2922, 2853, 1460, 1333, 1304, 1151, 1010, 923, 739; δ_H (400 MHz, $CDCl_3$) 7.87 (br s, 1H), 7.59–7.53 (m, 1H), 7.44–7.40 (m, 1H), 7.32–7.27 (m, 1H), 7.19–7.09 (m, 2H), 6.39–6.35 (m, 1H), 6.26–6.14 (m, 2H), 5.30–5.24 (m, 1H), 5.14–5.03 (m, 2H), 2.29 (s, 3H); δ_C (100 MHz, $CDCl_3$) 154.2, 142.3, 136.0, 135.4, 132.1, 129.3, 121.7, 119.2, 118.6, 117.2, 110.7, 110.5, 108.2, 107.1, 40.5, 8.5; HRMS (APCI$^+$): Found: 238.122122; $C_{16}H_{16}NO^+$ (MH$^+$) Requires 238.122641 (2.2 ppm error); $[\alpha]_D^{20}$ −25.51 (c = 0.29, $CHCl_3$).

1-(Benzo[*b*]thiophen-3-yl)prop-2-en-1-ol (1m)

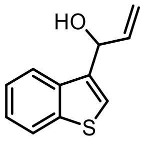

Prepared according to general procedure A using benzo[*b*]thiophene-3-carbaldehyde (2.43 g, 15.0 mmol), vinyl magnesium bromide (16.5 mL, 16.5 mmol, 1.0 M. in THF), in dry THF (30 mL). Purification by flash chromatography on silica gel (6:4 hexane:Et_2O) afforded the title compound **1m** as a yellow oil (2.55 g, 89% yield); R_f 0.37 (1:1 hexane:Et_2O:); ν_{max} (thin film)/cm^{-1} 3339, 3079, 1459, 1427, 1249, 1055, 986, 926, 781, 759, 732; δ_H (400 MHz, $CDCl_3$) 7.94–7.90 (m, 1H), 8.89–8.85 (m, 1H), 7.42–7.33 (m, 3H), 6.21 (ddd, *J* = 17.1, 10.3, 5.8 Hz, 1H), 5.60–5.55 (m, 1H), 5.48 (app. dt, *J* = 17.1, 1.4 Hz, 1H), 5.31 (app. dt, *J* = 10.3, 1.4 Hz, 1H), 2.09 (d, *J* = 4.4 Hz, 1H); δ_C (100 MHz, $CDCl_3$) 141.1, 138.8, 137.6, 137.4, 124.6, 124.2, 123.4, 123.0, 122.7, 116.3, 70.9; HRMS (ESI$^+$): Found: 213.0336; $C_{11}H_{10}NaOS^+$ (MNa^+) Requires 213.0345 (4.2 ppm error).

(*S*)-2-(1-(Benzo[*b*]thiophen-3-yl)allyl)-3-methyl-1*H*-indole (3n)

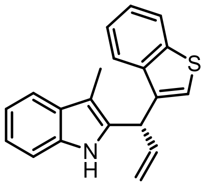

Prepared according to general procedure B using $[Ir(cod)Cl]_2$ (10.7 mg, 0.016 mmol), (*S*)-Carreira's Ligand **L1** (32.5 mg, 0.064 mmol), 1-(benzo[b]thiophen-3-yl)prop-2-en-1-ol **1m** (76.1 mg, 0.400 mmol), 3-methyl-1*H*-indole **2a** (68.2 mg, 0.520 mmol) and $Mg(ClO_4)_2$ (22.3 mg, 0.100 mmol) in dry CH_2Cl_2 (2 mL). Purification by flash chromatography on silica gel (6:4 hexane:CH_2Cl_2) afforded the title compound **3n** as a colourless foam (89.1 mg, 73% yield, 98% *ee*); R_f 0.33 (6:4 hexane:CH_2Cl_2); ν_{max} (thin film)/cm^{-1} 3427, 3056, 2915, 1459, 1427, 1333, 1307, 1239, 924, 766, 742, 732; δ_H (400 MHz, $CDCl_3$) 7.90–7.84 (m, 1H), 7.62–7.53 (m, 3H), 7.36–7.27 (m, 2H), 7.24 (s, 1H), 7.21–7.17 (m, 1H), 7.13–7.08 (m, 2H), 6.37 (ddd, *J* = 17.1, 10.0, 6.0 Hz, 1H), 5.38–5.31 (m, 2H), 5.08–4.99 (m, 1H), 2.36 (s, 3H); δ_C (100 MHz, $CDCl_3$) 140.8, 138.2, 136.8, 136.0, 135.3, 132.9, 129.6, 124.7, 124.4, 123.9, 123.0, 122.4, 121.5, 119.2, 118.5, 117.6, 110.8, 108.3, 40.8, 8.7; HRMS (APCI$^+$): Found: 304.116043; $C_{20}H_{18}NS^+$ (MH^+) Requires 304.115447 (−2.0 ppm error); $[\alpha]_D^{20}$ −25.08 (c = 1.43, $CHCl_3$).

(*S*)-3-Methyl-2-(1-(1-tosyl-1*H*-indol-3-yl)allyl)-1*H*-indole (3o) and (*E*)-3-Methyl-2-(3-(1-tosyl-1*H*-indol-3-yl)allyl)-1*H*-indole (3o')

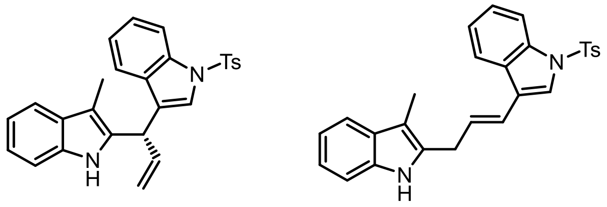

Prepared according to general procedure B using $[Ir(cod)Cl]_2$ (10.7 mg, 0.016 mmol), (*S*)-Carreira's Ligand **L1** (32.5 mg, 0.064 mmol), 1-(1-tosyl-1*H*-indol-3-yl)prop-2-en-1-ol **1n** (131 mg, 0.400 mmol), 3-methyl-1*H*-indole **2a** (68.2 mg, 0.520 mmol) and $Mg(ClO_4)_2$ (22.3 mg, 0.100 mmol) in dry CH_2Cl_2 (2 mL). Purification by flash chromatography on silica gel (7:3 hexane:Et_2O) afforded the title compounds **3o** (100 mg, 57%, >99% *ee*) and **3o'** (35.0 mg, 20%) as white foams (135 mg, 77% overall yield).

Data for branched isomer **3o**: R_f 0.33 (8:2 hexane:Et_2O); ν_{max} (thin film)/cm^{-1} 3411, 3055, 2918, 1459, 1447, 1366, 1187, 1171, 1122, 1093, 979, 908, 737; δ_H (400 MHz, $CDCl_3$) 8.01 (d, *J* = 8.3 Hz, 1H), 7.78 (d, *J* = 8.3 Hz, 2H), 7.63–7.54 (m, 2H), 7.41 (s, 1H), 7.34–7.28 (m, 1H), 7.28–7.23 (m, 3H), 7.22–7.18 (m, 1H), 7.19–7.09 (m, 3H), 6.34–6.22 (m, 1H), 6.26–6.14 (m, 2H), 5.32 (ddd, *J* = 10.2, 1.3, 1.3 Hz, 1H), 5.18–5.13 (m, 1H), 5.06 (d, *J* = 17.1 Hz, 1H) 2.38 (s, 3H), 2.28 (s, 3H); δ_C (100 MHz, $CDCl_3$) 145.2, 136.2, 135.7, 135.3, 135.3, 132.5, 130.04, 129.96, 129.5, 127.0, 125.2, 124.3, 123.5, 123.1, 121.6, 120.3, 119.3, 118.5, 117.7, 113.9, 110.8, 108.2, 38.0, 21.7, 8.6; HRMS (ESI$^+$): Found: 441.1623; $C_{27}H_{25}N_2O_2S^+$ (MH^+) Requires 441.1631 (2.0 ppm error); $[\alpha]_D^{20}$ −18.47 (c = 0.83, $CHCl_3$).

Data for linear isomer **3o'**: R_f 0.12 (8:2 hexane:Et_2O); ν_{max} (thin film)/cm^{-1} 3411, 2919, 1462, 1446, 1366, 1188, 1173, 1123, 1099, 979, 742, 674; δ_H (400 MHz, $CDCl_3$) 8.02−7.98 (m, 1H), 7.81 (br s, 1H), 7.78–7.73 (m, 1H), 7.71–7.66 (m, 1H), 7.56–7.50 (m, 2H), 7.36–7.26 (m, 3H), 7.23–7.19 (m, 2H), 7.17–7.07 (m, 2H), 6.56 (dd, *J* = 16.0, 0.7 Hz, 1H), 6.40 (dt, *J* = 16.0, 6.5 Hz, 1H), 3.69 (dd, *J* = 6.5, 0.7 Hz, 2H), 2.33 (s, 3H), 2.30 (s, 3H); δ_C (100 MHz, $CDCl_3$) 145.2, 135.6, 135.5, 135.2, 132.0, 130.0, 129.5, 129.1, 128.1, 127.0, 125.1, 123.7, 123.6, 122.6, 121.5, 120.5, 120.34, 119.3, 118.4, 113.9, 110.5, 107.8, 30.4, 21.7, 8.6; HRMS (ESI$^+$): Found: 463.1472; $C_{27}H_{24}N_2NaO_2S^+$ (MH^+) Requires 463.1451 (−4.6 ppm error).

(*S*)-3-Phenyl-2-(1-phenylallyl)-1*H*-indole (3p)

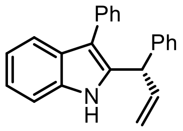

Prepared according to general procedure B using $[Ir(cod)Cl]_2$ (10.7 mg, 0.016 mmol), (*S*)-Carreira's Ligand **L1** (32.5 mg, 0.064 mmol), 1-phenylprop-2-en-1-ol **1a** (53.7 mg, 0.400 mmol), 3-phenyl-1*H*-indole **2c** (101 mg, 0.520 mmol) and $Mg(ClO_4)_2$ (22.3 mg, 0.100 mmol) in dry CH_2Cl_2 (2 mL). Purification by flash chromatography on silica gel (9:1 hexane:Et_2O) afforded the title compound **3p** as a clear and colourless oil (77.0 mg, 62% yield, 98% *ee*); R_f 0.31 (9:1 hexane:Et_2O); ν_{max} (thin film)/cm^{-1} 3426, 3057, 2926, 1601, 1492, 1457, 1430, 1017, 926, 784, 746; δ_H (400 MHz, $CDCl_3$) 7.86 (br s, 1H), 7.69 (d, *J* = 7.9 Hz, 1H), 7.56–7.50 (m, 2H), 7.48–7.42 (m, 2H), 7.37–7.30 (m, 4H), 7.29–7.22 (m, 3H), 7.22–7.16 (m, 1H), 7.16–7.11 (m, 1H), 6.39–6.27 (m, 1H), 5.41–5.35 (m, 1H), 5.19 (br d, *J* = 5.7 Hz, 1H), 5.10–5.02 (m, 1H); δ_C (100 MHz, $CDCl_3$) 141.5, 138.7, 135.7, 135.1, 135.0, 129.7, 128.9, 128.7, 128.4, 128.0, 127.1, 126.4, 122.1, 120.2, 119.5, 118.2, 115.8, 110.9, 45.9; HRMS (ESI$^+$): Found: 310.1593; $C_{23}H_{20}N^+$ (MH$^+$) Requires 310.1590 (−1.0 ppm error); $[\alpha]_D^{20}$ –89.34 (c = 0.20, $CHCl_3$).

(*S*)-3-Benzyl-2-(1-phenylallyl)-1*H*-indole (3q)

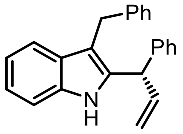

Prepared according to general procedure B using $[Ir(cod)Cl]_2$ (10.7 mg, 0.016 mmol), (*S*)-Carreira's Ligand **L1** (32.5 mg, 0.064 mmol), 1-phenylprop-2-en-1-ol **1a** (53.7 mg, 0.400 mmol), 3-benzyl-1*H*-indole **2d** (108 mg, 0.520 mmol) and $Mg(ClO_4)_2$ (22.3 mg, 0.100 mmol) in dry CH_2Cl_2 (2 mL). Purification by flash chromatography on silica gel (9:1 hexane:Et_2O) afforded the title compound **3q** as a clear and colourless oil (120 mg, 93% yield, <99% *ee*); R_f 0.31 (9:1 hexane:Et_2O); ν_{max} (thin film)/cm^{-1} 3422, 3058, 3026, 2923, 1601, 1493, 1458, 1433, 1307, 924, 742; δ_H (400 MHz, $CDCl_3$) 7.69 (br s, 1H), 7.41 (d, *J* = 7.9 Hz, 1H), 7.34–7.28 (m, 2H), 7.28–7.24 (m, 3H), 7.23–7.17 (m, 5H), 7.16–7.09 (m, 2H), 7.06–6.99 (m, 1H), 6.32–6.22 (m, 1H), 5.30 (ddd, *J* = 10.2, 1.4, 1.4 Hz, 1H), 5.05 (br d, *J* = 6.2 Hz, 1H), 5.01 (ddd, *J* = 17.1, 1.4, 1.4 Hz, 1H), 4.11 (s, 2H); δ_C (100 MHz, $CDCl_3$) 141.4, 141.1, 138.1, 135.6, 135.4, 129.0, 128.9, 128.6, 128.42, 128.37, 127.1, 125.9, 121.6, 119.5, 119.1, 117.9, 111.5, 110.7, 46.2, 30.2; HRMS (ESI$^+$): Found: 324.1740; $C_{24}H_{22}N^+$ (MH$^+$) Requires 324.1747 (2.1 ppm error); $[\alpha]_D^{20}$ –81.92 (c = 0.35, $CHCl_3$).

(*S*)-3-Allyl-2-(1-phenylallyl)-1*H*-indole (3r)

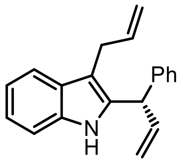

Prepared according to general procedure B using $[Ir(cod)Cl]_2$ (10.7 mg, 0.016 mmol), (*S*)-Carreira's Ligand **L1** (32.5 mg, 0.064 mmol), 1-phenylprop-2-en-1-ol **1a** (53.7 mg, 0.400 mmol), 3-allyl-1*H*-indole **2e** (81.8 mg, 0.520 mmol) and $Mg(ClO_4)_2$ (22.3 mg, 0.100 mmol) in dry CH_2Cl_2 (2 mL). Purification by flash chromatography on silica gel (9:1 hexane:Et_2O) afforded the title compound **3r** as a clear and colourless oil (106 mg, 96% yield, 98% *ee*); R_f 0.35 (9:1 hexane:Et_2O); ν_{max} (thin film)/cm^{-1} 3417, 3057, 2898, 1636, 1491, 1458, 1432, 1339, 1301, 994, 912, 739, 700; δ_H (400 MHz, $CDCl_3$) 7.65 (br s, 1H), 7.58–7.54 (m, 1H), 7.36–7.30 (m, 2H), 7.29–7.22 (m, 4H), 7.16–7.05 (m, 2H), 6.29 (ddd, *J* = 17.1, 10.2, 6.3 Hz, 1H), 6.00–5.88 (m, 1H), 5.32 (ddd, *J* = 10.2, 1.4, 1.4 Hz, 1H), 5.11–4.96 (m, 4H), 3.51–3.47 (m, 2H); δ_C (100 MHz, $CDCl_3$) 141.2, 138.2, 137.3, 135.5, 135.0, 128.85, 128.80, 128.5, 127.1, 121.5, 119.4, 118.9, 117.8, 114.8, 110.8, 110.3, 46.2, 28.7; HRMS (ESI$^+$): Found: 274.1585; $C_{20}H_{20}N^+$ (MH$^+$) Requires 274.1590 (2.1 ppm error); $[\alpha]_D^{20}$ –67.65 (c = 0.82, $CHCl_3$).

(*S*)-3-Chloro-2-(1-phenylallyl)-1*H*-indole (3s)

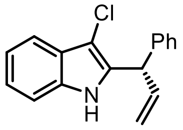

Prepared according to general procedure B using $[Ir(cod)Cl]_2$ (10.7 mg, 0.016 mmol), (*S*)-Carreira's Ligand **L1** (32.5 mg, 0.064 mmol), 1-phenylprop-2-en-1-ol **1a** (53.7 mg, 0.400 mmol), 3-chloro-1*H*-indole **2f** (78.8 mg, 0.520 mmol) and $Mg(ClO_4)_2$ (22.3 mg, 0.100 mmol) in dry CH_2Cl_2 (2 mL). Purification by flash chromatography on silica gel (9:1 hexane:Et_2O) afforded the title compound **3s** as a clear and colourless oil (89.1 mg, 83% yield, >99% *ee*); R_f 0.58 (1:1 hexane:Et_2O); ν_{max} (thin film)/cm^{-1} 3380, 2924, 1599, 1492, 1455, 1304, 1155, 1093, 925, 743; δ_H (400 MHz, $CDCl_3$) 7.72 (br s, 1H), 7.62–7.57 (m, 1H), 7.39–7.33 (m, 2H), 7.31–7.24 (m, 4H), 7.21–7.14 (m, 2H), 6.32–6.22 (m, 1H), 5.36 (ddd, *J* = 10.2, 1.9, 0.9 Hz, 1H), 5.22 (br d, *J* = 6.1 Hz, 1H), 5.08 (ddd, *J* = 17.1, 1.9, 0.9 Hz, 1H); δ_C (100 MHz, $CDCl_3$) 140.2, 136.8, 134.3, 134.1, 129.0, 128.4, 127.3, 126.3, 122.7, 120.5, 118.2, 118.0, 111.2, 104.1, 45.7; HRMS (APCI$^+$): Found: 268.089965; $C_{17}H_{15}ClN^+$ (MH$^+$) Requires 268.088754 (4.5 ppm error); $[\alpha]_D^{20}$ –69.7 (c = 0.15, $CHCl_3$).

(*S*)-5-Bromo-3-methyl-2-(1-phenylallyl)-1*H*-indole (3t)

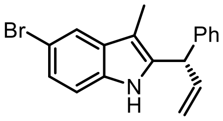

Prepared according to general procedure B using $[Ir(cod)Cl]_2$ (10.7 mg, 0.016 mmol), (*S*)-Carreira's Ligand **L1** (32.5 mg, 0.064 mmol), 1-phenylprop-2-en-1-ol **1a** (53.7 mg, 0.400 mmol), 5-bromo-3-methyl-1*H*-indole **2g** (109 mg, 0.520 mmol) and $Mg(ClO_4)_2$ (22.3 mg, 0.100 mmol) in dry CH_2Cl_2 (2 mL). Purification by flash chromatography on silica gel (7:3 hexane:CH_2Cl_2) afforded the title compound **3t** as a clear and colourless oil (120 mg, 92% yield, 99% *ee*); R_f 0.40 (6:4 hexane:CH_2Cl_2); ν_{max} (thin film)/cm^{-1} 3433, 3060, 2918, 1492, 1464, 1450, 1281, 1307, 1047, 995, 923, 903, 793, 701, 587; δ_H (400 MHz, $CDCl_3$) 7.67–7.56 (m, 2H), 7.38–7.32 (m, 2H), 7.30–7.17 (m, 4H), 7.10 (d, *J* = 8.5 Hz, 1H), 6.26 (ddd, *J* = 16.9, 10.2, 6.4 Hz, 1H), 5.33 (ddd, J = 10.3, 1.3, 1.3 Hz, 1H), 5.07–4.99 (m, 2H), 2.19 (s, 3H); δ_C (100 MHz, $CDCl_3$) 140.8, 137.7, 135.8, 133.9, 131.4, 129.0, 128.4, 127.2, 124.2, 121.2, 117.8, 112.5, 112.1, 108.0, 46.4, 8.6; HRMS (ESI$^+$): Found: 342.0476; $C_{18}H_{17}{}^{79}BrNO^+$ (MH^+) Requires 342.0488 (3.5 ppm error); $[\alpha]_D^{20}$ −62.21 (c = 1.15, $CHCl_3$).

(*S*)-6-Chloro-3-methyl-2-(1-phenylallyl)-1*H*-indole (3u)

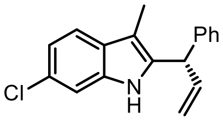

Prepared according to general procedure B using $[Ir(cod)Cl]_2$ (10.7 mg, 0.016 mmol), (*S*)-Carreira's Ligand **L1** (32.5 mg, 0.064 mmol), 1-phenylprop-2-en-1-ol **1a** (53.7 mg, 0.400 mmol), 6-chloro-3-methyl-1*H*-indole **2h** (86.1 mg, 0.520 mmol) and $Mg(ClO_4)_2$ (22.3 mg, 0.100 mmol) in dry CH_2Cl_2 (2 mL). Purification by flash chromatography on silica gel (9:1 hexane:Et_2O) afforded the title compound **3u** as a clear and colourless oil (111 mg, 98% yield, >99% *ee*); R_f 0.33 (9:1 hexane:Et_2O); ν_{max} (thin film)/cm^{-1} 3431, 2924, 2854, 1708, 1493, 1461, 1334, 1229, 1067, 920, 800, 702; δ_H (400 MHz, $CDCl_3$) 7.61 (br s, 1H), 7.48 (d, *J* = 8.4 Hz, 1H), 7.43–7.38 (m, 2H), 7.36–7.27 (m, 3H), 7.23 (d, *J* = 1.7 Hz, 1H), 7.12 (dd, *J* = 8.4, 1.7 Hz, 1H), 6.31 (ddd, J = 17.4, 10.2, 6.0 Hz, 1H), 5.42–5.34 (m, 1H), 5.14–5.04 (m, 2H), 2.28 (s, 3H); δ_C (100 MHz, $CDCl_3$) 140.9, 137.7, 135.6, 135.1, 128.9, 128.4, 128.1, 127.2, 127.1, 119.9, 119.3, 117.7, 110.6, 108.3, 46.4, 8.6; HRMS (APCI$^+$): Found: 282.103449; $C_{18}H_{17}ClN^+$ (MH^+) Requires 282.104404 (3.4 ppm error); $[\alpha]_D^{20}$ −18.40 (c = 0.34, $CHCl_3$).

Methyl (*S*)-2-(2-(1-phenylallyl)-1*H*-indol-3-yl)acetate (3v)

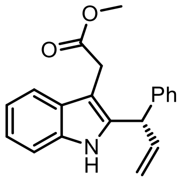

Prepared according to general procedure B using $[Ir(cod)Cl]_2$ (10.7 mg, 0.016 mmol), (*S*)-Carreira's Ligand **L1** (32.5 mg, 0.064 mmol), 1-phenylprop-2-en-1-ol **1a** (53.7 mg, 0.400 mmol), methyl 2-(1*H*-indol-3-yl)acetate **2i** (98.4 mg, 0.520 mmol) and $Mg(ClO_4)_2$ (22.3 mg, 0.100 mmol) in dry CH_2Cl_2 (2 mL). Purification by flash chromatography on silica gel (100% CH_2Cl_2) afforded the title compound **3v** as a clear and colourless oil (106 mg, 87% yield, 99% *ee*); R_f 0.27 (100% CH_2Cl_2); ν_{max} (thin film)/cm^{-1} 3383, 2958, 2923, 2855, 1729, 1493, 1460, 1435, 1305, 1264, 1162, 1025, 924, 742; δ_H (400 MHz, $CDCl_3$) 7.73 (br s, 1H), 7.61–7.56 (m, 1H), 7.38–7.31 (m, 2H), 7.30–7.24 (m, 4H), 7.17–7.09 (m, 2H), 6.28 (ddd, *J* = 17.1, 10.2, 6.3 Hz, 1H), 5.33 (ddd, *J* = 10.2, 1.4, 1.4 Hz, 1H), 5.17 (br d, *J* = 6.3 Hz, 1H), 5.05 (ddd, *J* = 17.1, 1.5, 1.5 Hz, 1H), 3.71 (d, *J* = 2.4 Hz, 2H), 3.61 (s, 3H); δ_C (100 MHz, $CDCl_3$) 172.4, 140.8, 137.8, 136.2, 135.2, 128.9, 128.6, 128.5, 127.2, 121.9, 119.9, 118.6, 117.8, 110.9, 105.5, 52.0, 46.3, 30.2; HRMS (ESI$^+$): Found: 306.1485; $C_{20}H_{20}NO_2^+$ (MH^+) Requires 306.1489 (1.3 ppm error); $[\alpha]_D^{20}$ –15.54 (c =0.21, $CHCl_3$).

(*S*)-2-(2-(1-Phenylallyl)-1*H*-indol-3-yl)ethan-1-ol (3w)

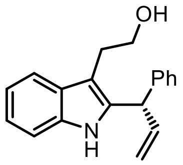

Prepared according to general procedure B using $[Ir(cod)Cl]_2$ (10.7 mg, 0.016 mmol), (*S*)-Carreira's Ligand **L1** (32.5 mg, 0.064 mmol), 1-phenylprop-2-en-1-ol **1a** (53.7 mg, 0.400 mmol), 2-(1*H*-indol-3-yl)ethan-1-ol **2j** (83.8 mg, 0.520 mmol) and $Mg(ClO_4)_2$ (22.3 mg, 0.100 mmol) in dry CH_2Cl_2 (2 mL). Purification by flash chromatography on silica gel (6:4 toluene:Et_2O) afforded the title compound **3w** as a clear and colourless oil (44.7 mg, 40% yield, 99% *ee*); R_f 0.49 (6:4 toluene:Et_2O); ν_{max} (thin film)/cm^{-1} 3559, 3410, 3057, 2936, 2880, 1492, 1459, 1220, 1041, 1009, 923, 744, 701; δ_H (400 MHz, $CDCl_3$) 7.74 (br s, 1H), 7.58 (d, *J* = 7.6 Hz, 1H), 7.37–7.30 (m, 2H), 7.29–7.23 (m, 4H), 7.18–7.07 (m, 2H), 6.31 (ddd, *J* = 17.1, 10.2, 6.2 Hz, 1H), 5.34 (dd, *J* = 10.2, 1.8, 1H), 5.14 (br d, *J* = 6.2 Hz, 1H), 5.04 (dd, *J* = 17.1, 1.8 Hz, 1H), 3.82 (app. q, *J* = 6.4 Hz, 2H), 3.03 (t, *J* = 6.4 Hz, 2H), 1.39 (t, *J* = 6.4 Hz, 1H); δ_C (100 MHz, $CDCl_3$) 141.2, 138.5, 136.1, 135.6, 129.0, 128.7, 128.3, 127.2, 121.8, 119.7, 118.7, 117.9, 110.9, 108.7, 62.9, 46.1, 27.9; HRMS (ESI$^+$): Found: 278.1533; $C_{19}H_{20}NO^+$ (MH^+) Requires 278.1539 (2.5 ppm error); $[\alpha]_D^{20}$ –7.50 (c = 0.36, $CHCl_3$).

(*S*)-3-(2-((*tert*-Butyldimethylsilyl)oxy)ethyl)-2-(1-phenylallyl)-1*H*-indole (3x)

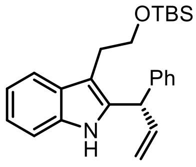

Prepared according to general procedure B using $[Ir(cod)Cl]_2$ (10.7 mg, 0.016 mmol), (*S*)-Carreira's Ligand **L1** (32.5 mg, 0.064 mmol), 1-phenylprop-2-en-1-ol **1a** (53.7 mg, 0.400 mmol), 3-(2-((*tert*-butyldimethylsilyl)oxy)ethyl)-1*H*-indole **2k** (143 mg, 0.520 mmol) and $Mg(ClO_4)_2$ (22.3 mg, 0.100 mmol) in dry CH_2Cl_2 (2 mL). Purification by flash chromatography on silica gel (8:2 hexane:Et_2O) afforded the title compound **3x** as a clear and colourless oil (130 mg, 83% yield, 98% *ee*); R_f 0.62 (7:3 hexane:Et_2O); v_{max} (thin film)/cm^{-1} 3414, 2953, 2928, 2856, 1459, 1253, 1088, 1066, 1005, 922, 833, 775, 738; δ_H (400 MHz, $CDCl_3$) 7.72 (br s, 1H), 7.66–7.62 (m, 1H), 7.40–7.35 (m, 2H), 7.33–7.26 (m, 4H), 7.20–7.11 (m, 2H), 6.35 (ddd, *J* = 17.1, 10.2, 6.2 Hz, 1H), 5.37 (app. dt, 10.2, 1.4 Hz, 1H), 5.18 (br d, *J* = 6.2 Hz, 1H), 5.08 (app. dt, *J* = 17.1, 1.4 Hz, 1H), 3.89–3.71 (m, 2H), 3.06 (t, *J* = 7.9 Hz, 2H), 0.95 (s, 9H), 0.09 (s, 6H); δ_C (100 MHz, $CDCl_3$) 141.3, 138.4, 135.5, 135.4, 129.0, 128.8, 128.4, 127.1, 121.5, 119.4, 118.7, 117.8, 110.8, 109.3, 63.8, 46.1, 28.4, 26.2, 18.6, −5.1; HRMS (ESI$^+$): Found: 392.2406; $C_{25}H_{34}NOSi^+$ (MH^+) Requires 392.2404 (−0.6 ppm error); $[\alpha]_D^{20}$ –30.82 (c = 1.38, $CHCl_3$).

***tert*-Butyl (*S*)-(2-(2-(1-phenylallyl)-1*H*-indol-3-yl)ethyl)carbamate (3y)**

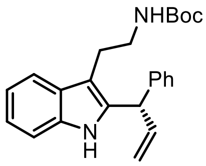

Prepared according to general procedure B using $[Ir(cod)Cl]_2$ (10.7 mg, 0.016 mmol), (*S*)-Carreira's Ligand **L1** (32.5 mg, 0.064 mmol), 1-phenylprop-2-en-1-ol **1a** (53.7 mg, 0.400 mmol), *tert*-butyl (2-(1*H*-indol-3-yl)ethyl)carbamate **2l** (135 mg, 0.520 mmol) and $Mg(ClO_4)_2$ (22.3 mg, 0.100 mmol) in dry CH_2Cl_2 (2 mL). Purification by flash chromatography on silica gel (95:05 toluene:Et_2O) afforded the title compound **3y** as a clear and colourless oil (99.5 mg, 66% yield, 98% *ee*); R_f 0.31 (7:3 hexane:Et_2O); v_{max} (thin film)/cm^{-1} 3418, 3329, 2976, 2930, 1690, 1505, 1459, 1365, 1247, 994, 909, 731, 700; δ_H (400 MHz, $CDCl_3$) 7.70 (br s, 1H), 7.58 (d, *J* = 7.6 Hz, 1H), 7.38–7.24 (m, 6H), 7.17–7.07 (m, 2H), 6.36–6.23 (m, 1H), 5.33 (d, *J* = 10.2, 1H), 5.12–5.00 (m, 2H), 4.57 (br s, 1H), 3.45–3.30 (m, 2H), 3.00–2.89 (m, 2H), 1.43 (s, 9H); δ_C (100 MHz, $CDCl_3$) 153.0, 141.1, 138.3, 135.7, 135.6, 129.0, 128.6, 128.4, 127.2, 121.8, 119.6, 118.7, 117.9, 110.8, 109.7, 79.1, 46.1, 40.9, 28.6, 24.8; HRMS (ESI$^+$): Found: 377.2222; $C_{24}H_{29}N_2O_2^+$ (MH^+) Requires 377.2224 (0.3 ppm error); $[\alpha]_D^{20}$ –62.83 (c = 0.35, $CHCl_3$).

(*S*)-4-Methyl-*N*-(2-(2-(1-phenylallyl)-1*H*-indol-3-yl)ethyl)benzenesulfonamide (3z)

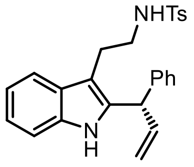

Prepared according to general procedure B using $[Ir(cod)Cl]_2$ (10.7 mg, 0.016 mmol), (*S*)-Carreira's Ligand **L1** (32.5 mg, 0.064 mmol), 1-phenylprop-2-en-1-ol **1a** (53.7 mg, 0.400 mmol), *N*-(2-(1*H*-indol-3-yl)ethyl)-4-methylbenzenesulfonamide **2m** (163 mg, 0.520 mmol) and $Mg(ClO_4)_2$ (22.3 mg, 0.100 mmol) in dry CH_2Cl_2 (2 mL). Purification by flash chromatography on silica gel (1:1 hexane:Et_2O) afforded the title compound **3z** as a white solid (168 mg, 97% yield, >99% *ee*); mp 52–54 °C; R_f 0.24 (1:1 hexane:Et_2O); ν_{max} (thin film)/cm^{-1} 3381, 3058, 1599, 1493, 1460, 1322, 1157, 1093, 815, 745; δ_H (400 MHz, $CDCl_3$) 7.72 (br s, 1H), 7.60 (d, *J* = 8.0 Hz, 2H), 7.38 (d, *J* = 7.9 Hz, 1H), 7.35–7.30 (m, 2H), 7.29–7.18 (m, 6H), 7.16–7.10 (m, 1H), 7.07–7.02 (m, 1H), 6.26 (ddd, *J* = 16.7, 10.2, 6.2 Hz, 1H), 5.35–5.28 (m, 1H), 5.04–4.96 (m, 2H), 4.31 (br s, 1H), 3.20–3.12 (m, 2H), 2.93 (t, *J* = 6.8 Hz, 2H) 2.39 (s, 3H); δ_C (100 MHz, $CDCl_3$) 143.3, 140.9, 138.2, 136.9, 136.0, 135.5, 129.7, 128.9, 128.3, 128.2, 127.2, 127.1, 121.8, 119.7, 118.3, 118.0, 111.0, 108.1, 46.1, 43.3, 24.8, 21.6; HRMS (ESI$^+$): Found: 453.1608; $C_{26}H_{26}N_2NaO_2S^+$ (MNa$^+$) Requires 453.1607 (−0.3 ppm error); $[\alpha]_D^{20}$ +26.93 (c = 0.27, $CHCl_3$).

Methyl (*S*)-2-((*tert*-butoxycarbonyl)amino)-3-(2-((*S*)-1-phenylallyl)-1*H*-indol-3-yl)propanoate (3aa)

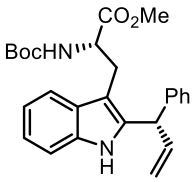

Prepared according to general procedure B using $[Ir(cod)Cl]_2$ (10.7 mg, 0.016 mmol), (*S*)-Carreira's Ligand **L1** (32.5 mg, 0.064 mmol), 1-phenylprop-2-en-1-ol **1a** (53.7 mg, 0.400 mmol), methyl (*tert*-butoxycarbonyl)-*L*-tryptophanate **2n** (166 mg, 0.520 mmol) and $Mg(ClO_4)_2$ (22.3 mg, 0.100 mmol) in dry CH_2Cl_2 (2 mL). Purification by flash chromatography on silica gel (7:3 hexane:Et_2O) afforded the title compound **3aa** as a white foam as a single diastereomer existing as a 13:1 mixture of rotamers (105 mg, 60% yield, >99% *ee*); R_f (both rotamers) 0.33 (7:3 hexane:Et_2O); ν_{max} (thin film)/cm^{-1} 3407, 2978, 2931, 1743, 1698, 1493, 1366, 1162, 907, 728; δ_H (400 MHz, $CDCl_3$) 7.99–7.73 (m, 1H, both rotamers), 7.63–7.50 (m, 1H, both), 7.41–7.22 (m, 6H, both), 7.19–7.08 (m, 2H, both), 6.44–6.09 (m, 1H, both), 5.46–5.30 (m, 1H, both), 5.17–4.75 (m, 3H, both), 4.73–4.46 (m, 1H, both), 3.72–3.54 (m, 3H, both), 3.38–3.09 (m, 2H, both), 1.49–1.27 (m, 9H, both). Only peaks for major rotamer listed δ_C (100 MHz, $CDCl_3$) 173.0, 155.3, 140.8, 138.2, 136.5, 135.5, 128.9, 128.7, 128.3, 127.2, 121.8, 119.7, 118.7, 118.0, 110.8,

107.0, 79.8, 54.1, 52.4, 46.0, 28.4, 27.3; HRMS (ESI$^+$): Found: 457.2104; $C_{26}H_{30}N_2NaO_4^+$ (MNa$^+$) Requires 457.2098 (–1.4 ppm error); $[\alpha]_D^{20}$ –3.29 (c = 0.40, $CHCl_3$)

(*S*)-1,3-Dimethyl-2-(1-phenylallyl)-1*H*-indole (3ab)

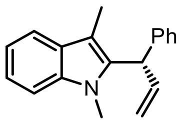

Prepared according to general procedure B using $[Ir(cod)Cl]_2$ (10.7 mg, 0.016 mmol), (*S*)-Carreira's Ligand **L1** (32.5 mg, 0.064 mmol), 1-phenylprop-2-en-1-ol **1a** (53.7 mg, 0.400 mmol), 1,3-dimethyl-1*H*-indole **2o** (75.5 mg, 0.520 mmol) and $Mg(ClO_4)_2$ (22.3 mg, 0.100 mmol) in dry CH_2Cl_2 (2 mL). Purification by flash chromatography on silica gel (8:2 cyclohexane:Et_2O) afforded the title compound **3ab** as a clear and colourless oil (75.2 mg, 72% yield, 84% *ee*); R_f 0.71 (8:2 cyclohexane:Et_2O); ν_{max} (thin film)/cm^{-1} 3358, 2924, 2853, 1709, 1673, 1630, 1518, 1455, 1237, 1129, 1026, 908, 850, 730, 696; δ_H (400 MHz, $CDCl_3$) 7.57 (d, *J* = 7.8 Hz, 1H), 7.33–7.25 (m, 3H), 7.24–7.18 (m, 4H), 7.15–7.09 (m, 1H), 6.45 (ddd, *J* = 16.9, 10.2, 6.7 Hz, 1H), 5.32 (d, *J* = 10.2 Hz, 1H), 5.20 (d, *J* = 6.7 Hz, 1H), 5.08 (d, *J* = 16.9 Hz, 1H), 3.45 (s, 3H), 2.27 (s, 3H); δ_C (100 MHz, $CDCl_3$) 141.2, 137.6, 137.0, 136.0, 128.64, 128.61, 128.0, 126.6, 121.3, 118.8, 118.5, 117.6, 108.8, 108.5, 45.7, 30.9, 9.4; $[\alpha]_D^{20}$ +145.61 (c = 0.20, $CHCl_3$). Note: HRMS could not be obtained.

(*S*)-6-Chloro-3-methyl-2-(1-(3,4,5-trimethoxyphenyl)allyl)-1*H*-indole (3ac)

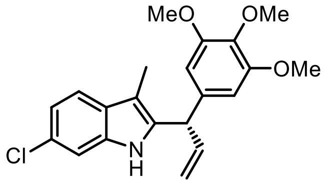

Prepared according to general procedure B using $[Ir(cod)Cl]_2$ (10.7 mg, 0.016 mmol), (*S*)-Carreira's Ligand **L1** (32.5 mg, 0.064 mmol), 1-(3,4,5-trimethoxyphenyl)prop-2-en-1-ol **1j** (89.7 mg, 0.400 mmol), 6-chloro-3-methyl-1*H*-indole **2h** (86.1 mg, 0.520 mmol) and $Mg(ClO_4)_2$ (22.3 mg, 0.100 mmol) in dry CH_2Cl_2 (2 mL). Purification by flash chromatography on silica gel (95:5 toluene:Et_2O) afforded the title compound **3ac** as a white solid (112 mg, 75% yield, >99% *ee*); mp 43–45 °C; R_f 0.32 (95:5 toluene:Et_2O); ν_{max} (thin film)/cm^{-1} 3352, 2937, 1589, 1506, 1461, 1416, 1233, 1128, 1003, 918; δ_H (400 MHz, $CDCl_3$) 7.71 (br s, 1H), 7.43 (d, *J* = 8.4 Hz, 1H), 7.24 (d, *J* = 1.8 Hz, 1H), 7.06 (dd, *J* = 8.4, 1.8 Hz, 1H), 6.47 (s, 2H), 6.22 (ddd, *J* = 16.8, 10.1, 6.4 Hz, 1H), 5.32 (app. dt, *J* = 10.1, 1.3 Hz, 1H), 5.05 (app. dt, *J* = 16.8, 1.5 Hz, 1H), 4.97 (br d, *J* = 6.4 Hz, 1H), 3.86 (s, 3H), 3.80 (s, 6H), 2.24 (s, 3H); δ_C (100 MHz, $CDCl_3$) 153.6, 137.5, 137.0, 136.5, 135.6, 134.9, 128.1, 127.2, 119.8, 119.3, 117.6, 110.7, 108.3, 105.4,

61.0, 56.2, 46.7, 8.7; HRMS (ESI$^+$): Found: 394.1183; $C_{21}H_{22}ClNNaO_3^+$ (MNa$^+$) Requires 394.1180 (−0.6 ppm error); $[\alpha]_D^{20}$ −30.47 (c = 0.75, $CHCl_3$).

(*S*)-3-(1-Phenylallyl)-1*H*-indole (5a)

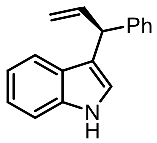

Prepared according to general procedure B using $[Ir(cod)Cl]_2$ (10.7 mg, 0.016 mmol), (*S*)-Carreira's Ligand **L1** (32.5 mg, 0.064 mmol), 1-phenylprop-2-en-1-ol **1a** (53.7 mg, 0.400 mmol), 1*H*-indole (60.9 mg, 0.520 mmol) and $Mg(ClO_4)_2$ (22.3 mg, 0.100 mmol) in dry CH_2Cl_2 (2 mL). Purification by flash chromatography on silica gel (100% toluene) afforded the title compound **5a** as a white foam (75.0 mg, 80% yield, 97% *ee*); R_f 0.51 (100% toluene); ν_{max} (thin film)/cm^{-1} 3418, 3057, 1490, 1456, 1337, 1219, 1094, 995, 919, 741, 700; δ_H (400 MHz, $CDCl_3$) 7.97 (br s, 1H), 7.41 (d, *J* = 8.0 Hz, 1H), 7.36 (d, *J* = 8.0 Hz, 1H), 7.32–7.28 (m, 4H), 7.25–7.16 (m, 2H), 7.07–7.01 (m, 1H), 6.90–6.87 (m, 1H), 6.42–6.30 (m, 1H), 5.21 (d, *J* = 10.1 Hz, 1H), 5.13–5.05 (m, 1H), 4.98 (d, *J* = 7.1 Hz, 1H); δ_C (100 MHz, $CDCl_3$) 143.3, 140.6, 136.8, 128.6, 128.5, 126.9, 126.4, 122.6, 122.2, 120.0, 119.5, 118.7, 115.6, 111.2, 47.1; HRMS (APCI$^+$): Found: 234.126622; $C_{17}H_{16}N^+$ (MH$^+$) Requires 234.127726 (−4.7 ppm error); $[\alpha]_D^{20}$ +3.14 (c = 0.38, $CHCl_3$).

(*S*)-3-(1-(3-Bromophenyl)allyl)-1*H*-indole (5b)

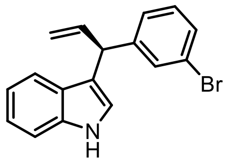

Prepared according to general procedure B using $[Ir(cod)Cl]_2$ (10.7 mg, 0.016 mmol), (*S*)-Carreira's Ligand **L1** (32.5 mg, 0.064 mmol), 1-(3-bromophenyl)prop-2-en-1-ol **1g** (85.2 mg, 0.400 mmol), 1*H*-indole (60.9 mg, 0.520 mmol) and $Mg(ClO_4)_2$ (22.3 mg, 0.100 mmol) in dry CH_2Cl_2 (2 mL). Purification by flash chromatography on silica gel (100% toluene) afforded the title compound **5b** as a clear and colourless oil (101 mg, 81% yield, 94% *ee*); R_f 0.70 (100% toluene); ν_{max} (thin film)/cm^{-1} 3414, 3056, 1590, 1566, 1472, 1456, 1417, 1337, 1219, 1094, 921, 741; δ_H (400 MHz, $CDCl_3$) 7.96 (br s, 1H), 7.52–7.48 (m, 1H), 7.44 (d, *J* = 7.9 Hz, 1H), 7.41–7.35 (m, 2H), 7.26–7.16 (m, 3H), 7.14–7.07 (m, 1H), 6.89 (d, *J* = 2.4 Hz, 1H), 6.35 (ddd, *J* = 17.1, 10.1, 7.1 Hz, 1H), 5.27 (app. dt, *J* = 10.1, 1.3 Hz, 1H), 5.12 (app. dt, *J* = 17.1, 1.3 Hz, 1H), 4.97 (d, *J* = 7.0 Hz, 1H); δ_C (100 MHz, $CDCl_3$) 145.7, 139.8, 136.7, 131.6, 130.1, 129.6, 127.3, 126.7, 122.7, 122.6, 122.4, 119.8, 119.6, 117.9, 116.3, 111.3, 46.7; HRMS (APCI$^+$): Found: 312.039131; $C_{17}H_{15}{}^{79}BrN^+$ (MH$^+$) Requires 312.038238 (−2.9 ppm error); $[\alpha]_D^{20}$ +4.87 (c = 0.27, $CHCl_3$).

(*S*)-3-(1-(4-Bromophenyl)allyl)-1*H*-indole (5c)

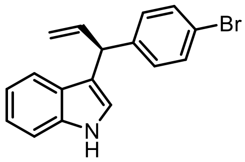

Prepared according to general procedure B using $[Ir(cod)Cl]_2$ (10.7 mg, 0.016 mmol), (*S*)-Carreira's Ligand **L1** (32.5 mg, 0.064 mmol), 1-(4-bromophenyl)prop-2-en-1-ol **1o** (85.2 mg, 0.400 mmol), 1*H*-indole (60.9 mg, 0.520 mmol) and $Mg(ClO_4)_2$ (22.3 mg, 0.100 mmol) in dry CH_2Cl_2 (2 mL). Purification by flash chromatography on silica gel (100% toluene) afforded the title compound **5c** as a clear and colourless oil (109 mg, 87% yield, 96% *ee*); R_f 0.47 (1:1 CH_2Cl_2:hexane); ν_{max} (thin film)/cm^{-1} 3413, 3056, 1636, 1485, 1456, 1417, 1403, 1337, 1219, 1095, 1071, 1010, 919, 813, 740, 580; δ_H (400 MHz, $CDCl_3$) 7.99 (br s, 1H), 7.46–7.40 (m, 2H), 7.39–7.35 (m, 2H), 7.23–7.14 (m, 3H), 7.09–7.02 (m, 1H), 6.89 (dd, *J* = 2.4, 0.9 Hz, 1H), 6.33 (ddd, *J* = 17.1, 10.1, 7.0 Hz, 1H), 5.26–5.20 (m, 1H), 5.11–5.03 (m, 1H), 4.94 (d, *J* = 7.0 Hz, 1H); δ_C (100 MHz, $CDCl_3$) 142.3, 140.0, 136.7, 131.5, 130.3, 126.7, 122.6, 122.3, 120.2, 119.8, 119.6, 118.0, 116.1, 111.3, 46.5; HRMS (APCI$^+$): Found: 312.036863; $C_{17}H_{15}{}^{79}BrN^+$ (MH^+) Requires 312.038238 (4.4 ppm error); $[\alpha]_D^{20}$ −2.26 (c = 1.44, $CHCl_3$). Spectroscopic data matched those reported in the literature.[17]

(*S*)-3-(1-(4-(Trifluoromethyl)phenyl)allyl)-1*H*-indole (5d)

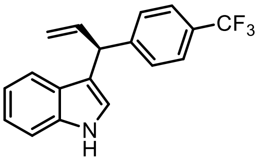

Prepared according to general procedure B using $[Ir(cod)Cl]_2$ (10.7 mg, 0.016 mmol), (*S*)-Carreira's Ligand **L1** (32.5 mg, 0.064 mmol), 1-(4-(trifluoromethyl)phenyl)prop-2-en-1-ol **1b** (80.9 mg, 0.400 mmol), 1*H*-indole (60.9 mg, 0.520 mmol) and $Mg(ClO_4)_2$ (22.3 mg, 0.100 mmol) in dry CH_2Cl_2 (2 mL). Purification by flash chromatography on silica gel (1:1 CH_2Cl_2:hexane) afforded the title compound **5d** as a clear and colourless oil (83.0 mg, 69% yield, 96% *ee*); R_f 0.59 (1:1 CH_2Cl_2:hexane); ν_{max} (thin film)/cm^{-1} 3415, 1617, 1457, 1418, 1324, 1163, 1120, 1109, 1067, 1018, 743; δ_H (400 MHz, $CDCl_3$) 7.99 (br s, 1H), 7.59 (d, *J* = 8.2 Hz, 2H), 7.48–7.35 (m, 4H), 7.29–7.20 (m, 1H), 7.14–7.05 (m, 1H), 6.92 (d, *J* = 2.0 Hz, 1H), 6.38 (ddd, *J* = 17.1, 10.1, 7.1 Hz, 1H), 5.29 (d, *J* = 10.1 Hz, 1H), 5.17–5.09 (m, 1H), 5.07 (d, *J* = 7.0 Hz, 1H); δ_C (100 MHz, $CDCl_3$) 147.4, 139.7, 136.8, 128.9, 128.7 (q, $^2J_{CF}$ = 32 Hz), 126.6, 125.4 (q, $^3J_{CF}$ = 4 Hz), 124.5 (q, $^1J_{CF}$ = 272 Hz), 122.7, 122.4, 119.71, 119.67, 117.7, 116.4, 111.4, 46.9; HRMS (APCI$^+$): Found: 302.113598; $C_{18}H_{15}F_3N^+$ (MH^+) Requires 302.115111 (5.0 ppm error); $[\alpha]_D^{20}$ +10.66 (c = 0.83, $CHCl_3$). Spectroscopic data matched those reported in the literature.[17]

(*S*)-3-(1-(4-Nitrophenyl)allyl)-1*H*-indole (5e)

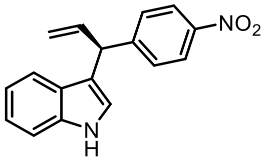

Prepared according to general procedure B using [Ir(cod)Cl]$_2$ (10.7 mg, 0.016 mmol), (*S*)-Carreira's Ligand **L1** (32.5 mg, 0.064 mmol), 1-(4-nitrophenyl)prop-2-en-1-ol **1e** (71.7 mg, 0.400 mmol), 1*H*-indole (60.9 mg, 0.520 mmol) and Mg(ClO$_4$)$_2$ (22.3 mg, 0.100 mmol) in dry CH_2Cl_2 (2 mL). Purification by flash chromatography on silica gel (1:1 CH_2Cl_2:hexane) afforded the title compound **5e** as a yellow oil (90.0 mg, 81% yield, 96% *ee*); R_f 0.48 (1:1 CH_2Cl_2:hexane); ν_{max} (thin film)/cm^{-1} 3415, 1596, 1516, 1457, 1344, 1108, 855, 835, 742; δ_H (400 MHz, $CDCl_3$) 8.20–8.10 (m, 3H), 7.48–7.42 (m, 2H), 7.39 (d, *J* = 8.2 Hz, 1H), 7.35–7.30 (m, 1H), 7.25–7.18 (m, 1H), 7.10–7.03 (m, 1H), 6.97 (d, *J* = 1.9 Hz, 1H), 6.36 (ddd, *J* = 17.1, 10.1, 7.2 Hz, 1H), 5.30 (app. dt, *J* = 10.1, 1.1 Hz, 1H), 5.16–5.05 (m, 2H); δ_C (100 MHz, $CDCl_3$) 151.1, 146.6, 139.0, 136.7, 129.4, 126.4, 123.7, 122.7, 122.5, 119.7, 119.4, 116.9 (2 x C), 111.5, 46.8; HRMS (APCI$^+$): Found: 297.112100; $C_{17}H_{15}N_2O_2^+$ (MH$^+$) Requires 279.112804 (−2.5 ppm error); $[\alpha]_D^{20}$ +5.67 (c = 0.33, $CHCl_3$).

(*S*)-3-(1-(4-Methoxyphenyl)allyl)-1*H*-indole (5f)

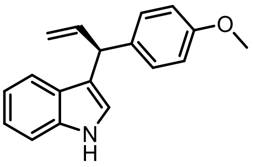

To an oven-dried Schlenk tube charged with a magnetic stirrer bar was added [Ir(cod)Cl]$_2$ (21.5 mg, 0.032 mmol) and (*S*)-Carreira's Ligand **L1** (65.0 mg, 0.128 mmol). The reaction vessel was purged by alternating vacuum and argon three times before dry CH_2Cl_2 (4 mL) was added. This mixture was stirred at RT for 15 min to form the active catalyst during which the solution turns from yellow to a deep red colour. 1-(4-Methoxyphenyl)prop-2-en-1-ol **1f** (131 mg, 0.800 mmol) was then added followed by the addition of 1*H*-indole (122 mg, 1.04 mmol) and Zn(OTf)$_2$ (72.7 mg, 0.200 mmol) under a back pressure of argon. The reaction mixture was then stirred at room temperature for 20 h. The reaction mixture was directly concentrated on to silica and purified by flash chromatography (95:05 toluene:$CHCl_3$) to afford the title compound **5f** as a yellow oil (137 mg, 65% yield, 98% *ee*); R_f 0.43 (95:05 toluene:$CHCl_3$); ν_{max} (thin film)/cm^{-1} 3416, 2954, 2835, 1609, 1509, 1456, 1244, 1176, 1034, 918, 823, 743; δ_H (400 MHz, $CDCl_3$) 7.98 (br s, 1H), 7.40 (d, *J* = 8.0 Hz, 1H), 7.35 (d, *J* = 8.0 Hz, 1H), 7.23–7.14 (m, 3H), 7.06–7.00 (m, 1H), 6.90–6.82 (m, 3H), 6.33 (ddd, *J* = 17.1, 10.1, 7.1 Hz, 1H), 5.20–5.15 (m, 1H), 5.08 (app. dt, *J* = 17.1, 1.6 Hz, 1H), 4.92 (d, J = 7.1 Hz, 1H), 3.79 (s, 3H); HRMS (ESI$^+$): Found: 286.1201; $C_{18}H_{17}NNaO^+$ (MNa$^+$) Requires

286.1202 (0.5 ppm error); $[\alpha]_D^{20}$ –3.52 (c = 0.31, $CHCl_3$). Spectroscopic data matched those reported in the literature.[17]

(*R*)-3-(1-(Benzo[b]thiophen-3-yl)allyl)-1*H*-indole (5g)

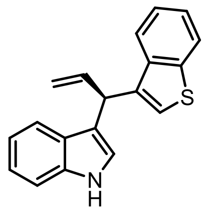

Prepared according to general procedure B using $[Ir(cod)Cl]_2$ (10.7 mg, 0.016 mmol), (*S*)-Carreira's Ligand **L1** (32.5 mg, 0.064 mmol), 1-(benzo[b]thiophen-3-yl)prop-2-en-1-ol **1m** (76.1 mg, 0.400 mmol), 1*H*-indole (60.9 mg, 0.520 mmol) and $Mg(ClO_4)_2$ (22.3 mg, 0.100 mmol) in dry CH_2Cl_2 (2 mL). Purification by flash chromatography on silica gel (100% toluene) afforded the title compound **5g** as a clear and colourless oil (97.8 mg, 85% yield, 95% *ee*); R_f 0.70 (100% toluene); ν_{max} (thin film)/cm^{-1} 3420, 1456, 1426, 1337, 1219, 1093, 994, 907, 761, 730; δ_H (400 MHz, $CDCl_3$) 7.97–7.87 (m, 2H), 7.75–7.70 (m, 1H), 7.53 (d, *J* = 8.0 Hz, 1H), 7.37 (d, *J* = 8.2 Hz, 1H), 7.35–7.27 (m, 2H), 7.23–7.18 (m, 1H), 7.12 (s, 1H), 7.11–7.05 (m, 1H), 6.84 (d, *J* = 1.8 Hz, 1H), 6.41 (ddd, *J* = 16.9, 10.1, 6.7 Hz, 1H), 5.34 (d, *J* = 6.67 Hz, 1H), 5.25 (app. dt, *J* = 10.1, 1.4 Hz, 1H), 5.12 (app. dt, *J* = 16.9, 1.4 Hz, 1H); δ_C (100 MHz, $CDCl_3$) 140.9, 138.9, 138.6, 137.9, 136.7, 126.9, 124.3, 123.9, 123.4, 122.9, 122.8, 122.8, 122.3, 119.8, 119.6, 117.1, 116.2, 111.3, 40.6; HRMS (APCI$^+$): Found: 290.099082; $C_{19}H_{16}NS^+$ (MH^+) Requires 290.099797 (2.5 ppm error); $[\alpha]_D^{20}$ −36.55 (c = 1.64, $CHCl_3$).

(*R*)-3-(Oct-1-en-3-yl)-1*H*-indole (5h)

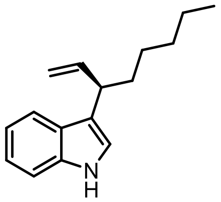

Prepared according to general procedure B using $[Ir(cod)Cl]_2$ (10.7 mg, 0.016 mmol), (*S*)-Carreira's Ligand **L1** (32.5 mg, 0.064 mmol), oct-1-en-3-ol **1p** (51.3 mg, 0.400 mmol), 1*H*-indole (60.9 mg, 0.520 mmol) and $Mg(ClO_4)_2$ (22.3 mg, 0.100 mmol) in dry CH_2Cl_2 (2 mL). Purification by flash chromatography on silica gel (6:4 hexane:CH_2Cl_2) afforded the title compound **5h** as a clear and colourless oil (70.0 mg, 77% yield, >99% *ee*); R_f 0.47 (6:4 hexane:CH_2Cl_2); ν_{max} (thin film)/cm^{-1} 3417, 2955, 2927, 2860, 1635, 1456, 1419, 1336, 1097, 911, 740; δ_H (400 MHz, $CDCl_3$) 7.91 (br s, 1H), 7.67 (d, *J* = 7.9 Hz, 1H), 7.36 (d, *J* = 8.1 Hz, 1H), 7.24–7.17 (m, 1H), 7.16–7.09 (m, 1H), 6.98 (d, *J* = 2.3 Hz, 1H), 6.12–5.92 (m, 1H), 5.20–5.10 (m, 1H), 5.09–5.02 (m, 1H), 3.59 (q, *J* = 7.5 Hz, 1H),

2.00–1.69 (m, 2H), 1.49–1.28 (m, 6H), 0.99–0.82 (m, 3H); δ_C (100 MHz, $CDCl_3$) 142.4, 136.6, 127.0, 122.0, 120.7, 119.7, 119.4, 119.2, 113.7, 111.2, 41.3, 34.8, 32.0, 27.5, 22.8, 14.3; HRMS (APCI$^+$): Found: 228.175010; $C_{16}H_{22}N^+$ (MH^+) Requires 228.174676 (−1.5 ppm error); $[\alpha]_D^{20}$ −31.86 (c = 0.40, $CHCl_3$).

2-((*S*)-1-(4-Nitrophenyl)allyl)-3-((*S*)-1-phenylallyl)-1*H*-indole (7b)

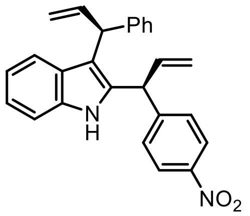

Prepared according to general procedure B using $[Ir(cod)Cl]_2$ (10.7 mg, 0.016 mmol), (*S*)-Carreira's Ligand **L1** (32.5 mg, 0.064 mmol), 1-(4-nitrophenyl)prop-2-en-1-ol **1e** (71.7 mg, 0.400 mmol), 3-(1-phenylallyl)-1*H*-indole **5a** (121 mg, 0.520 mmol) and $Mg(ClO_4)_2$ (22.3 mg, 0.100 mmol) in dry CH_2Cl_2 (2 mL). The crude reaction mixture was analysed by ^1H NMR spectroscopy using a trimethoxybenzene internal standard and a 60% yield (25:1 *dr*) of title compound **7b** was calculated. For the purposes of characterization, purification was achieved using preparative chromatography (100% $CHCl_3$) which afforded the title compound **7b** as a yellow oil in >99% enantiomeric excess. R_f 0.52 (100% $CHCl_3$); ν_{max} (thin film)/cm^{-1} 3415, 2924, 1597, 1516, 1457, 1434, 1345, 922, 854, 743, 700; δ_H (400 MHz, $CDCl_3$) 8.15–8.07 (m, 2H), 7.80 (br s, 1H), 7.40–7.35 (m, 1H), 7.33–7.30 (m, 1H), 7.28–7.20 (m, 6H), 7.19–7.13 (m, 2H), 7.04–6.99 (m, 1H), 6.41 (ddd, *J* = 17.1, 10.1, 7.2 Hz, 1H), 6.29 (ddd, *J* = 16.7, 10.3, 6.1 Hz, 1H), 5.46–5.40 (m, 1H), 5.19–5.11 (m, 2H), 5.04 (dd, *J* = 17.1, 1.1 Hz, 1H), 4.98 (br d, *J* = 7.2 Hz, 1H), 4.90 (ddd, *J* = 17.1, 1.5, 1.5 Hz, 1H); δ_C (100 MHz, $CDCl_3$) 148.6, 146.9, 142.7, 139.2, 137.0, 135.9, 133.4, 129.3, 128.34, 128.26, 127.7, 126.4, 123.9, 122.1, 120.4, 119.8, 119.5, 116.2, 114.6, 111.0, 45.9, 45.7; HRMS (APCI$^+$): Found: 395.177265; $C_{26}H_{23}N_2O_2^+$ (MH^+) Requires 395.175404 (−4.7 ppm error); $[\alpha]_D^{20}$ –59.36 (c = 0.14, $CHCl_3$).

1-(Phenyl-d$_5$)prop-2-en-1-*d*-1-ol (1r)

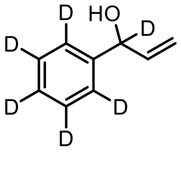

Prepared according to general procedure A using benzaldehyde-d_6^* (960 mg, 8.60 mmol), vinyl magnesium bromide (9.4 mL, 9.4 mmol, 1.0 M. in THF), in dry THF (17 mL). Purification by flash chromatography on silica gel (7:3 hexane:Et_2O) afforded the title compound **1r** as a yellow oil (1.10 g, 91% yield); R_f 0.19 (8:2 hexane:Et_2O:); ν_{max} (thin film)/cm^{-1} 3351, 2274, 1639, 1409, 1327, 1170, 1068, 989, 924, 840, 548; δ_H (400 MHz, $CDCl_3$) 6.06 (dd, *J* = 17.1, 10.3 Hz, 1H), 5.36 (dd, *J* = 17.1, 1.4 Hz, 1H), 5.21 (dd, *J* = 10.3, 1.4 Hz, 1H), 2.06 (s, 1H); δ_C (100 MHz,

$CDCl_3$) 142.5, 140.3, 128.2 (t, *J* = 24.4 Hz), 127.4 (t, *J* = 24.4 Hz), 126.0 (t, *J* = 24.1), 115.3, 75.0 (t, *J* = 22.0 Hz); HRMS ($APCI^+$): Found: 141.117182; $C_9H_5D_6O^+$ (MH^+) Requires 141.118102 (−6.5 ppm error).

*This chemical was purchased from Cambridge Isotope Laboratories; CAS no: 17901-93-8, Item no: DLM-396-PK.

2-((*S*)-1-(Phenyl-d$_5$)allyl-1-*d*)-3-((*S*)-1-phenylallyl)-1*H*-indole (8a)

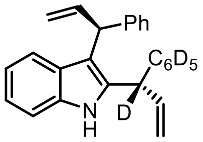

Prepared according to general procedure B using $[Ir(cod)Cl]_2$ (10.7 mg, 0.016 mmol), (*S*)-Carreira's Ligand **L1** (32.5 mg, 0.064 mmol), 1-(phenyl-d$_5$)prop-2-en-1-*d*-1-ol **1r** (56.1 mg, 0.400 mmol), 3-(1-phenylallyl)-1*H*-indole **5a** (121 mg, 0.520 mmol) and $Mg(ClO_4)_2$ (22.3 mg, 0.100 mmol) in dry CH_2Cl_2 (2 mL). The crude reaction mixture was analysed by ^{1}H NMR spectroscopy using a trimethoxybenzene internal standard and a 58% yield (>99:1 *dr*) of title compound **8a** was calculated. For the purposes of characterization, purification was achieved using preparative chromatography (1:1 cyclohexane:$CHCl_3$) which afforded the title compound **8a** as a clear and colourless oil in 95% enantiomeric excess. R_f 0.44 (6:4 $CHCl_3$:cyclohexane); ν_{max} (thin film)/cm^{-1} 3433, 3058, 2924, 1636, 1492, 1458, 1431, 1302, 994, 919, 742, 700; δ_H (400 MHz, $CDCl_3$) 7.70 (br s, 1H), 7.36–7.22 (m, 6H), 7.22–7.16 (m, 1H), 7.12–7.06 (m, 1H), 6.99–6.92 (m, 1H), 6.45 (ddd, *J* = 17.3, 10.2, 7.3 Hz, 1H), 6.28 (dd, *J* = 17.3, 10.2 Hz, 1H), 5.32 (dd, *J* = 10.2, 1.4 Hz, 1H), 5.18–5.13 (m, 1H), 5.06–4.93 (m, 3H); δ_C (100 MHz, $CDCl_3$) 143.1, 141.0, 139.7, 138.2, 135.8, 135.3, 128.4, 128.3 (2 x C), 128.1, 127.8, 126.2, 124.0, 121.5, 120.4, 119.4, 118.1, 115.9, 113.8, 110.8, 45.8, 29.9; HRMS ($APCI^+$): Found: 356.2277; $C_{26}H_{18}D_6N^+$ (MH^+) Requires 356.2280 (0.8 ppm error); $[\alpha]_D^{20}$ –106.12 (c = 0.37, $CHCl_3$). Note: enantiomeric excess could not be determined for this compound.

(*E*)-3-(3-(*p*-Tolyl)allyl)-1*H*-indole (9)

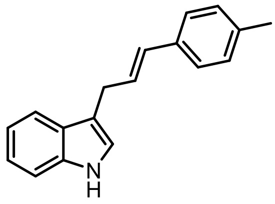

To an oven-dried Schlenk tube charged with a magnetic stirrer bar was added 1*H*-indole (234 mg, 2.00 mmol) and $Pd(PPh_3)_4$ (116 mg, 0.100 mmol). The reaction vessel was purged by alternating vacuum and argon three times before dry toluene (10 mL) was added. 1-(*p*-Tolyl)prop-2-en-1-ol **1q** (356 mg, 2.40 mmol) was then added followed by the addition of BEt_3 (0.60 mL, 0.600 mmol, 1.0 M in THF). The reaction mixture was heated to 60 °C and stirred for 24 h. The reaction mixture was then cooled and directly concentrated on to silica and purified by flash chromatography (100% toluene) to afford the title compound **9** as a white solid (180 mg, 36% yield); mp 103–105 °C; R_f 0.54 (100% toluene); ν_{max} (thin film)/cm^{-1} 3424, 3021, 2880, 1457, 1225, 1089, 969, 804, 736; δ_H (400 MHz, $CDCl_3$) 7.96 (br s, 1H), 7.66 (d, *J* = 7.8 Hz, 1H), 7.39–7.35 (m, 1H), 7.30–7.25 (m, 2H), 7.24–7.18 (m, 1H), 7.14–7.09 (m, 3H), 7.05–7.02 (m, 1H), 6.52 (d, *J* = 15.8 Hz, 1H), 5.47–5.38 (m, 1H), 3.69 (d, *J* = 6.4 Hz, 2H), 2.33 (s, 3H); δ_C (100 MHz, $CDCl_3$) 136.8, 136.5, 135.0, 130.4, 129.3, 128.3, 127.6, 126.1, 122.2, 121.9, 119.4, 119.3, 114.9, 111.2, 29.1, 21.3; HRMS (ESI$^+$): Found: 248.1434; $C_{18}H_{18}N^+$ (MH^+) Requires 248.1434 (−0.1 ppm error).

(*S*,*E*)-2-(1-Phenylallyl)-3-(3-(*p*-tolyl)allyl)-1*H*-indole (10a)

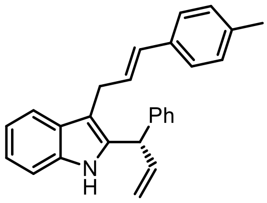

Prepared according to general procedure B using $[Ir(cod)Cl]_2$ (10.7 mg, 0.016 mmol), (*S*)-Carreira's Ligand **L1** (32.5 mg, 0.064 mmol), 1-phenylprop-2-en-1-ol **1a** (53.7 mg, 0.400 mmol), (*E*)-3-(3-(*p*-tolyl)allyl)-1*H*-indole **9** (129 mg, 0.520 mmol) and $Mg(ClO_4)_2$ (22.3 mg, 0.100 mmol) in dry CH_2Cl_2 (2 mL). The crude reaction mixture was analysed by ^{1}H NMR spectroscopy using a trimethoxybenzene internal standard and a 90% yield of title compound **10a** was calculated. For the purposes of characterization, purification of the crude material was achieved using preparative chromatography (1:1 CH_2Cl_2:hexane) which afforded the title compound **10a** as a clear and colourless oil in 98% enantiomeric excess; R_f 0.57 (1:1 CH_2Cl_2:hexane); ν_{max} (thin film)/cm^{-1} 3418, 3024, 2962, 2922, 1512, 1458, 1303, 1260, 1093, 1014, 964, 922, 798; δ_H (400 MHz, $CDCl_3$) 7.69 (br s, 1H), 7.60 (d, *J* = 7.8 Hz, 1H), 7.38–7.22 (m, 6H), 7.20–7.05 (m, 6H), 6.39 (d, *J* = 15.8 Hz, 1H), 6.35–6.22 (m, 1H), 5.37–5.28 (m, 1H), 5.13 (d, *J* = 6.2 Hz, 1H), 5.06 (dd, *J* = 17.1, 1.5 Hz, 1H), 3.63 (d, *J* = 5.9 Hz, 2H), 2.31 (s, 3H); δ_C (100 MHz, $CDCl_3$) 141.2, 138.2, 136.6, 135.5,

135.09, 135.06, 129.9, 129.2, 128.9 (2 x C), 128.5, 128.4, 127.1, 126.1, 121.6, 119.5, 119.0, 117.9, 110.8, 110.5, 46.3, 27.9, 21.3; HRMS (ESI$^+$): Found: 364.2062; $C_{27}H_{26}N^+$ (MH^+) Requires 364.2060 (−0.7 ppm error); $[\alpha]_D^{20}$ –37.74 (c = 0.20, $CHCl_3$).

(*R*)-3-Methyl-2-(oct-1-en-3-yl)-1*H*-indole (12a)

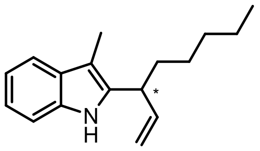

Prepared according to general procedure B using $[Ir(cod)Cl]_2$ (10.7 mg, 0.016 mmol), (*S*)-Carreira's Ligand **L1** (32.5 mg, 0.064 mmol), oct-1-en-3-ol **1p** (51.3 mg, 0.400 mmol), 3-methyl-1*H*-indole **2a** (68.2 mg, 0.520 mmol and $Mg(ClO_4)_2$ (22.3 mg, 0.100 mmol) in dry CH_2Cl_2 (2 mL). The crude reaction mixture was analysed by ^{1}H NMR spectroscopy using a trimethoxybenzene internal standard and a 42% yield of title compound **12a** was calculated. For the purposes of characterization, purification of the crude material was achieved using preparative chromatography (6:4 hexane:CH_2Cl_2) which afforded the title compound **12a** as a clear and colourless oil in >99% enantiomeric excess; R_f 0.60 (7:3 hexane:Et_2O); ν_{max} (thin film)/cm^{-1} 3420, 2955, 2925, 2856, 1462, 1333, 1240, 915, 739; δ_H (400 MHz, $CDCl_3$) 7.69 (br s, 1H), 7.54–7.49 (m, 1H), 7.31–7.27 (m, 1H), 7.16–7.06 (m, 2H), 5.99 (ddd, *J* = 16.8, 10.4, 6.1 Hz, 1H), 5.14–5.06 (m, 2H), 3.68–3.58 (m, 1H), 2.26 (s, 3H), 1.87–1.66 (m, 2H), 1.34–1.21 (m, 6H), 0.90–0.82 (m, 3H); δ_C (100 MHz, $CDCl_3$) 140.1, 135.9, 135.4, 129.5, 121.2, 119.1, 118.3, 114.9, 110.4, 107.6, 40.4, 34.0, 31.9, 27.3, 22.7, 14.2, 8.7; HRMS (ESI$^+$): Found: 242.1898; $C_{17}H_{24}N^+$ (MH^+) Requires 242.1903 (2.3 ppm error); $[\alpha]_D^{20}$ +14.61 (c = 0.28, $CHCl_3$).

3-Methyl-1-(oct-1-en-3-yl)-1*H*-indole (12a')

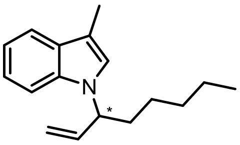

Prepared according to general procedure B using $[Ir(cod)Cl]_2$ (10.7 mg, 0.016 mmol), (*S*)-Carreira's Ligand **L1** (32.5 mg, 0.064 mmol), oct-1-en-3-ol **1p** (51.3 mg, 0.400 mmol), 3-methyl-1*H*-indole **2a** (68.2 mg, 0.520 mmol and $Mg(ClO_4)_2$ (22.3 mg, 0.100 mmol) in dry CH_2Cl_2 (2 mL). The crude reaction mixture was analysed by ^{1}H NMR spectroscopy using a trimethoxybenzene internal standard and a 41% yield of title compound **12a'** was calculated. For the purposes of characterization, purification of the crude material was achieved using preparative chromatography (6:4 hexane:CH_2Cl_2) which afforded the title compound **12a'** as a clear and colourless oil in 99% enantiomeric excess; R_f 0.72 (7:3 hexane:Et_2O); ν_{max} (thin film)/cm^{-1} 2955, 2927, 2858, 1460, 1356, 1186, 1015,

986, 921, 736; δ_H (400 MHz, $CDCl_3$) 7.58 (d, *J* = 7.8 Hz, 1H), 7.32 (d, *J* = 8.2 Hz, 1H), 7.22–7.16 (m, 1H), 7.14–7.07 (m, 1H), 6.94 (s, 1H), 6.06–5.94 (m, 1H), 5.13 (d, *J* = 10.5 Hz, 1H), 5.03 (d, *J* = 17.3 Hz, 1H), 4.85–4.76 (m, 1H), 2.35 (s, 3H), 2.04–1.91 (m, 2H), 1.35–1.22 (m, 6H), 0.89–0.82 (m, 3H); δ_C (100 MHz, $CDCl_3$) 138.4, 136.6, 128.8, 122.6, 121.3, 119.1, 118.7, 115.8, 110.7, 109.7, 58.1, 34.4, 31.7, 26.1, 22.6, 14.2, 9.9; HRMS ($APCI^+$): Found: 242.189868; $C_{17}H_{24}N^+$ (MH^+) Requires 242.190326 (1.9 ppm error); $[\alpha]_D^{20}$ −17.38 (c = 0.28, $CHCl_3$).

Kinetic isotope effect experiments

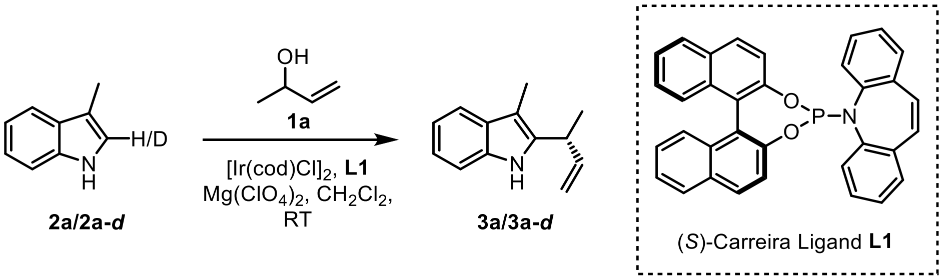

To an oven-dried Schlenk tube charged with a magnetic stirrer bar was added $[Ir(cod)Cl]_2$ (5.35 mg, 0.008 mmol) and (*S*)-Carreira Ligand **L1** (16.3 mg, 0.032 mmol). The reaction vessel was purged by alternating vacuum and argon three times before CD_2Cl_2 (1 mL) was added. This mixture was stirred at RT for 15 min to form the active catalyst during which the solution turns from yellow to a deep red colour. Allylic alcohol **1a** (26.9 mg, 0.200 mmol) was then added followed by the addition of indole **2a** (34.1 mg, 0.260 mmol) *or* indole **2a-*d*** (34.4 mg, 0.260 mmol) and $Mg(ClO_4)_2$ (11.2 mg, 0.05 mmol) under a back pressure of argon. The reaction mixture was then stirred for 5 min (800 rpm) at RT to ensure dissolution of reagents. Following this, the reaction mixture was transferred under an inert atmosphere to an NMR tube fitted with a Young's tap. At which point ^{1}H NMR spectra were recorded to monitor progress of the reaction. Results are displayed below.

Note: These experiments were conducted at RT to enable more accurate reaction monitoring. After transferring to an NMR tube there was no agitation of the reaction mixture.

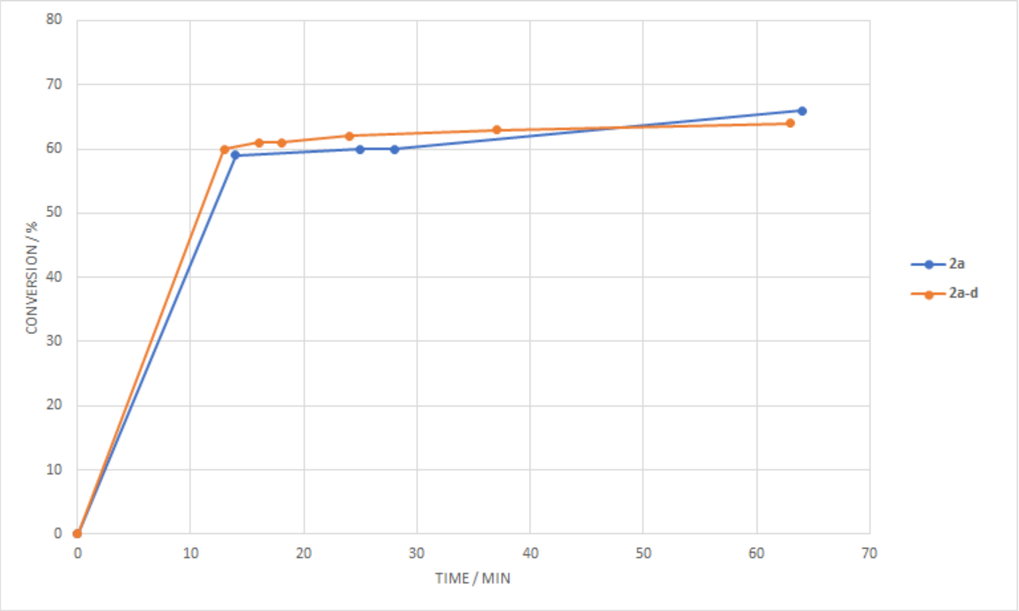

Figure S1. Progress of C2-allylation reaction using indole **2a** and C2-deuterated analogue **2a-*d***.

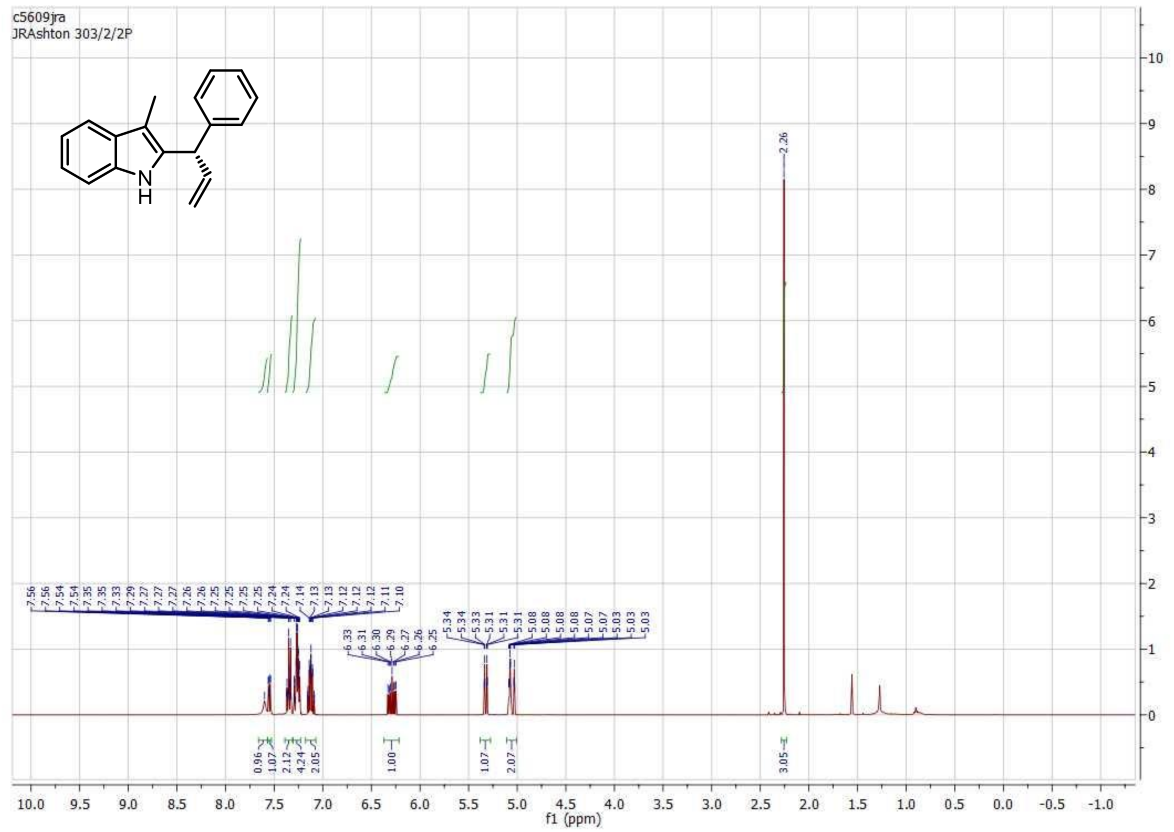
c5609jra
JRAshton 303/2/2P
f1 (ppm)

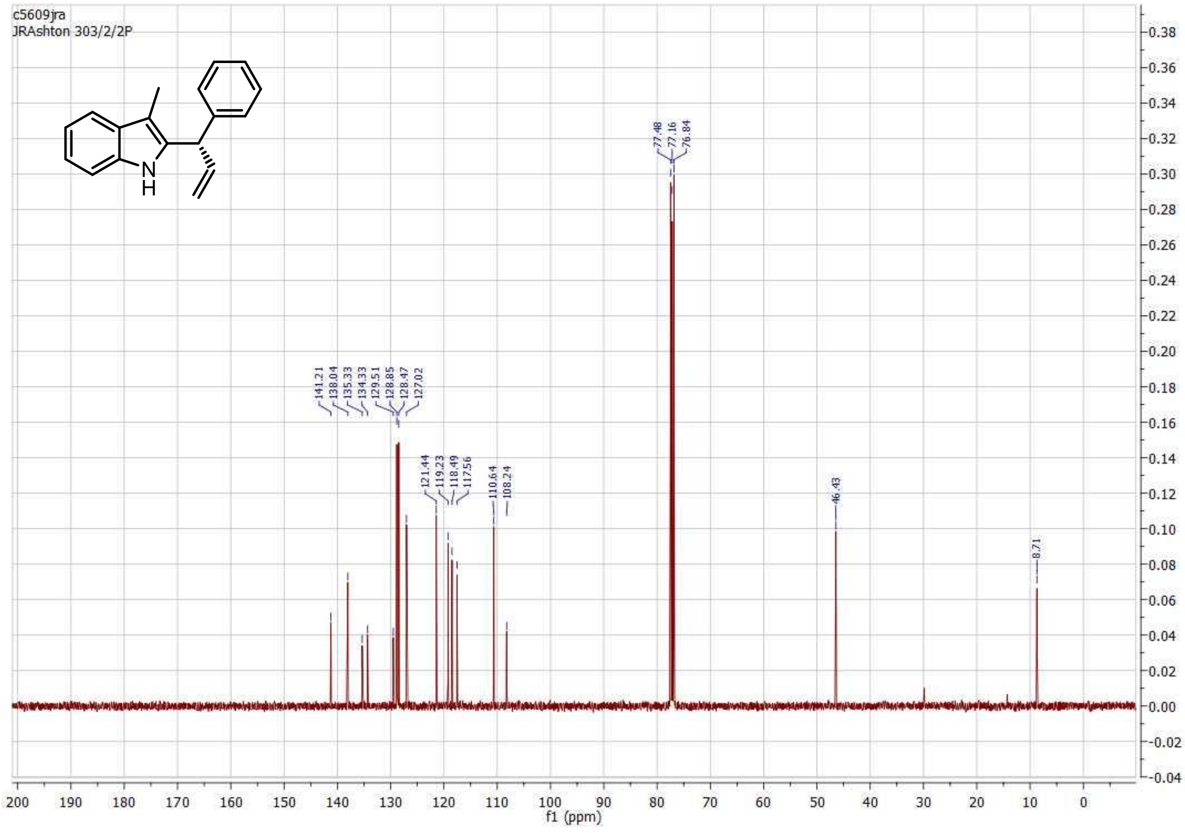
c5609jra
JRAshton 303/2/2P
f1 (ppm)

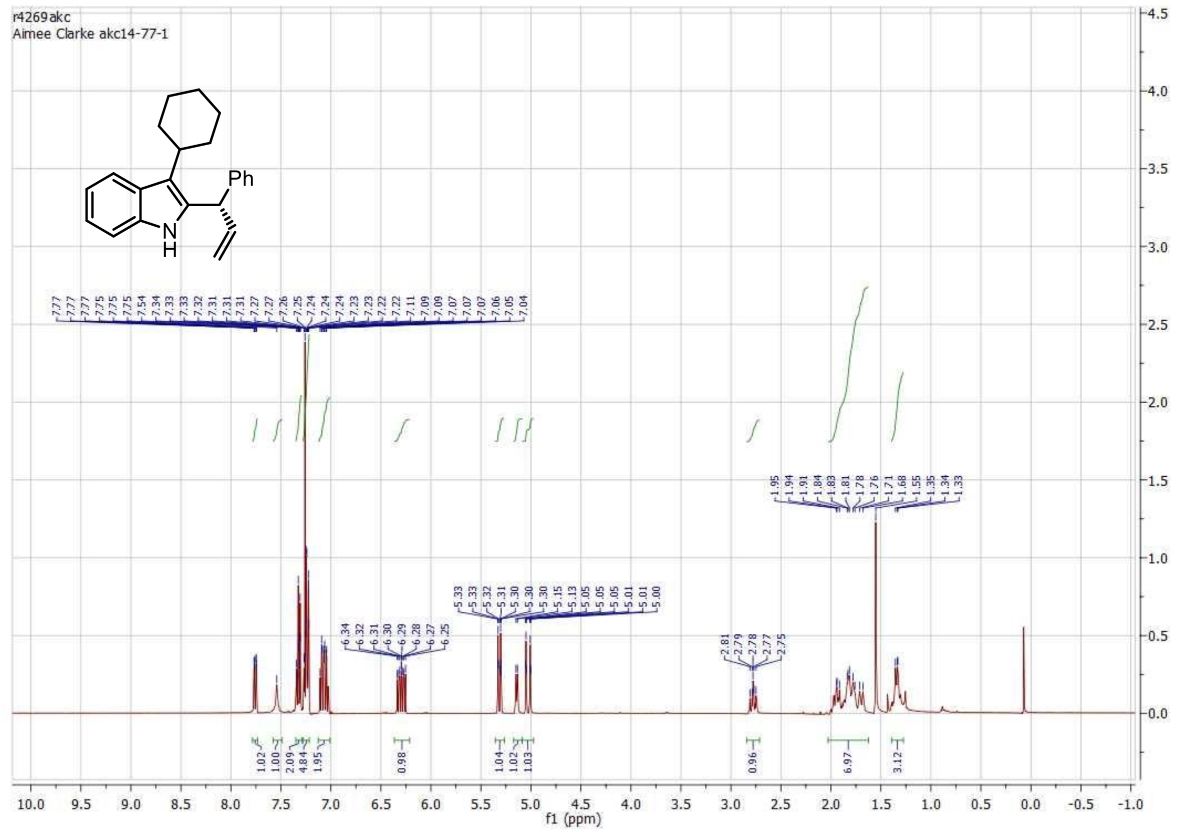
r4269akc
Aimee Clarke akc14-77-1
Ph
N
H
f1 (ppm)

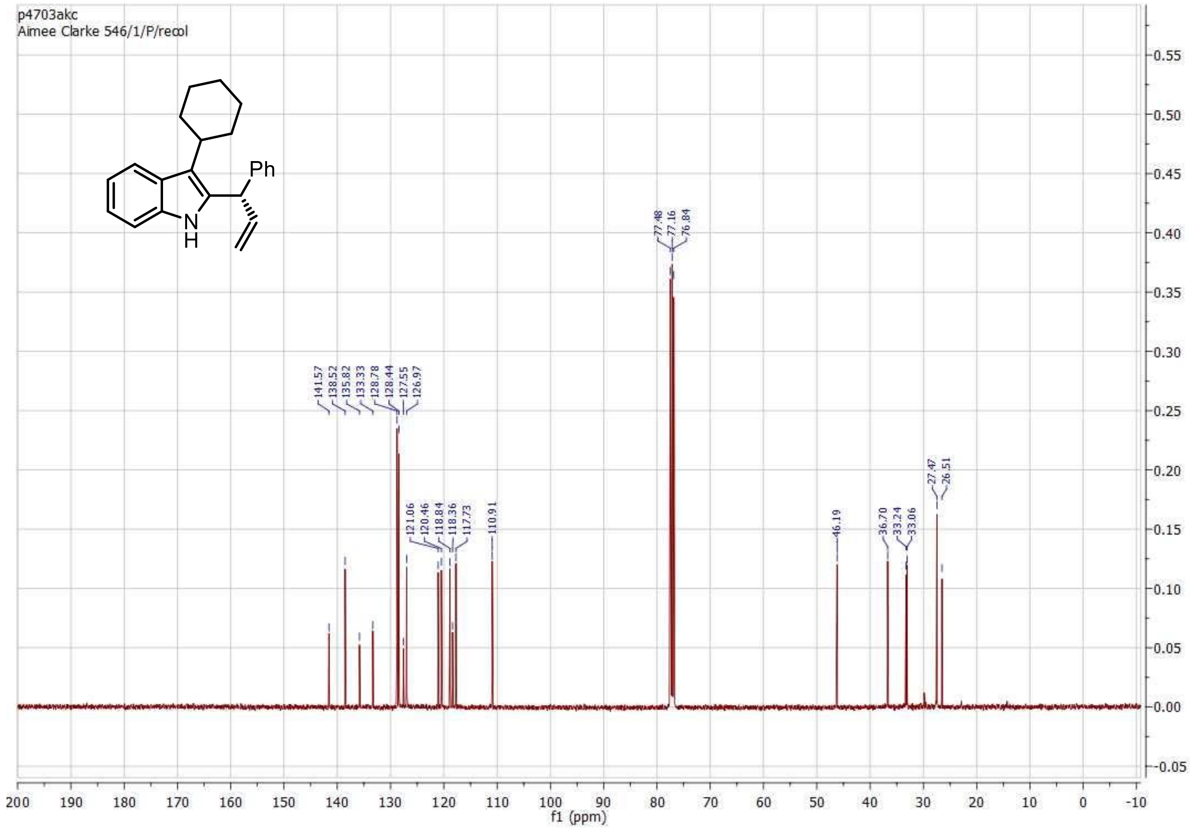
p4703akc
Aimee Clarke 546/1/P/recol
Ph
N
H
141.57
138.52
135.82
133.33
128.78
128.44
127.55
126.97
121.06
120.46
118.84
118.36
117.73
110.91
77.48
77.16
76.84
46.19
36.70
33.24
33.06
27.47
26.51
f1 (ppm)

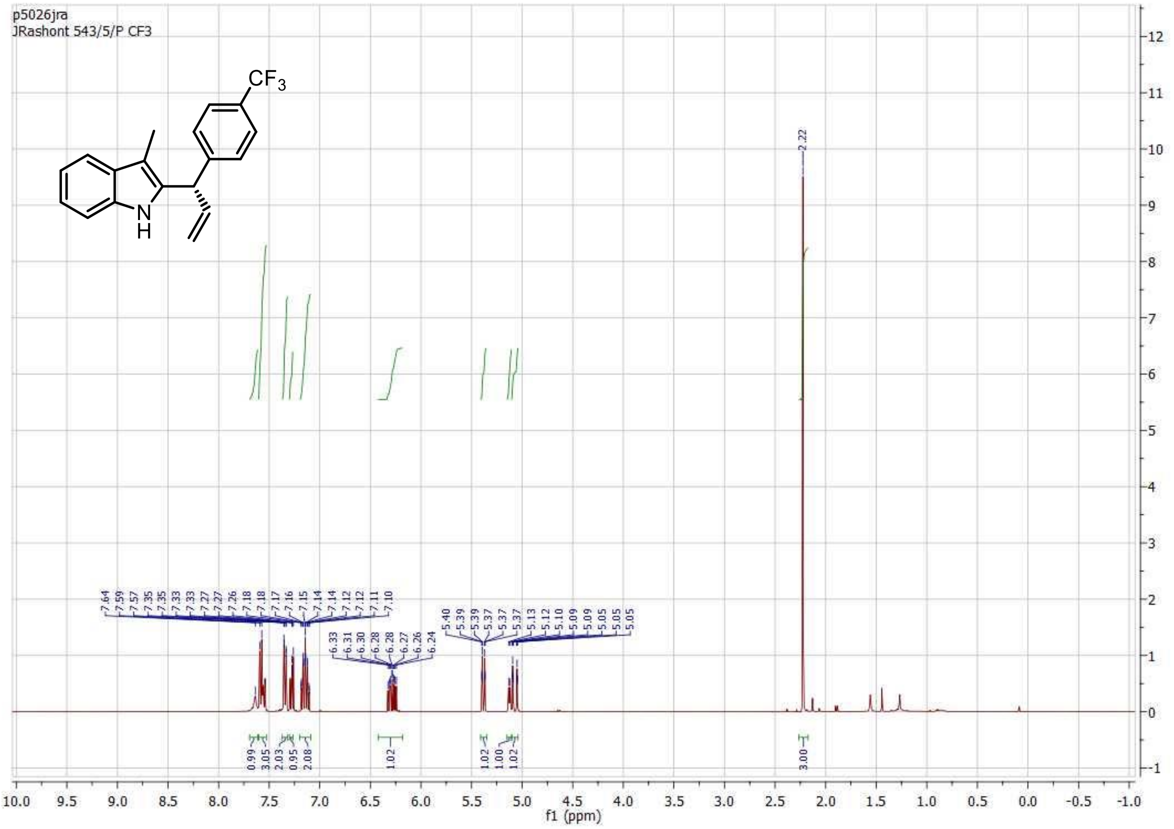
p5026jra
JRashont 543/5/P CF3
CF3
N
H
f1 (ppm)

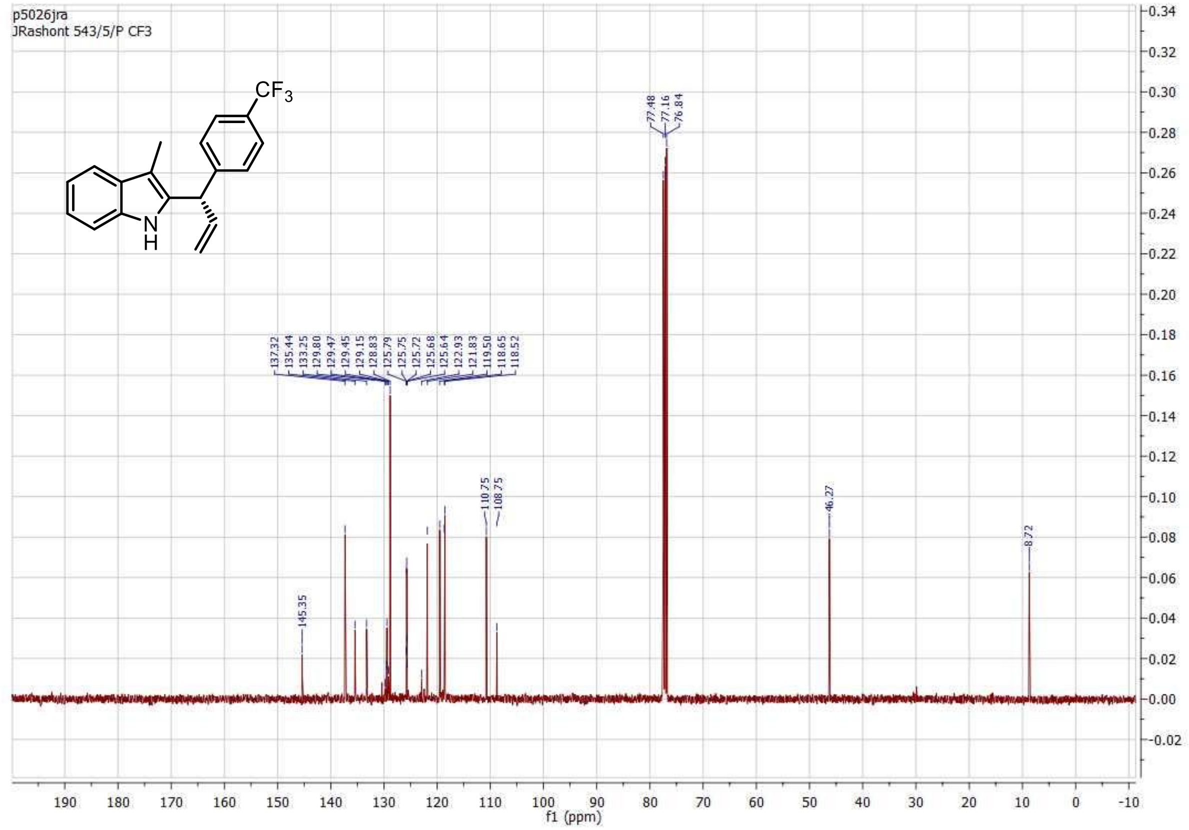
p5026jra
JRashont 543/5/P CF3
CF3
N
H
f1 (ppm)

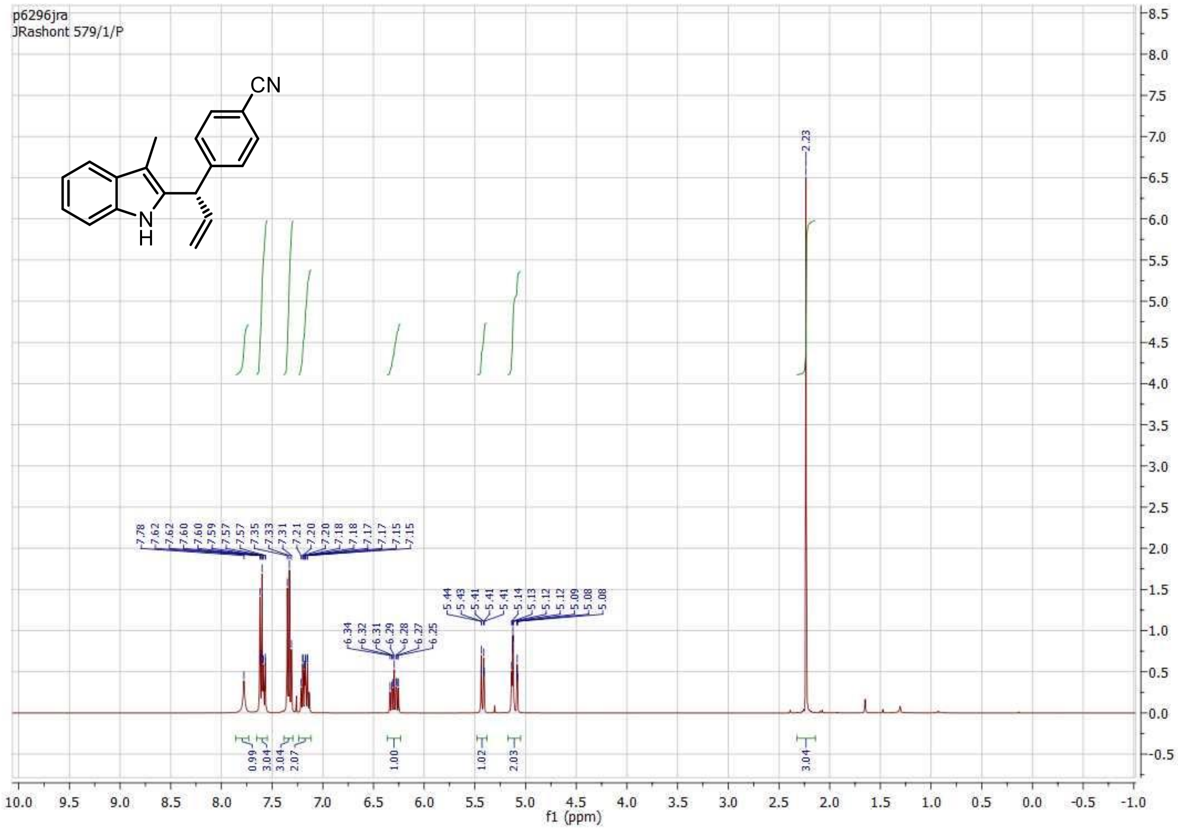
p6296jra
JRashont 579/1/P
CN
N
H
7.78 7.62 7.62 7.60 7.60 7.59 7.57 7.57 7.35 7.33 7.31 7.21 7.20 7.20 7.18 7.18 7.17 7.17 7.15 7.15
6.34 6.32 6.31 6.29 6.28 6.27 6.25
5.44 5.43 5.41 5.41 5.41 5.14 5.13 5.12 5.12 5.09 5.08 5.08
2.23
0.99 3.04 3.04 2.07 1.00 1.02 2.03 3.04
f1 (ppm)

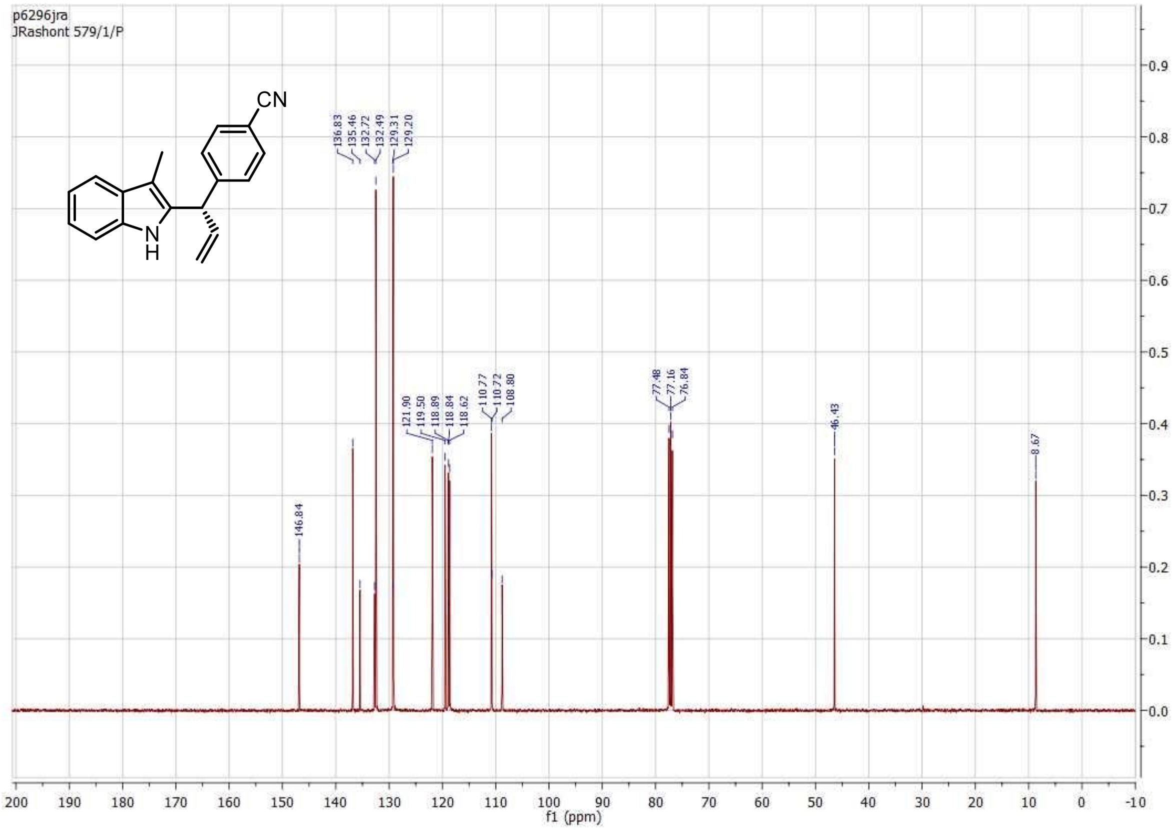
p6296jra
JRashont 579/1/P
CN
N
H
136.83 135.46 132.72 132.49 129.31 129.20
146.84
121.90 119.50 118.89 118.84 118.62 110.77 110.72 108.80
77.48 77.16 76.84
46.43
8.67
f1 (ppm)

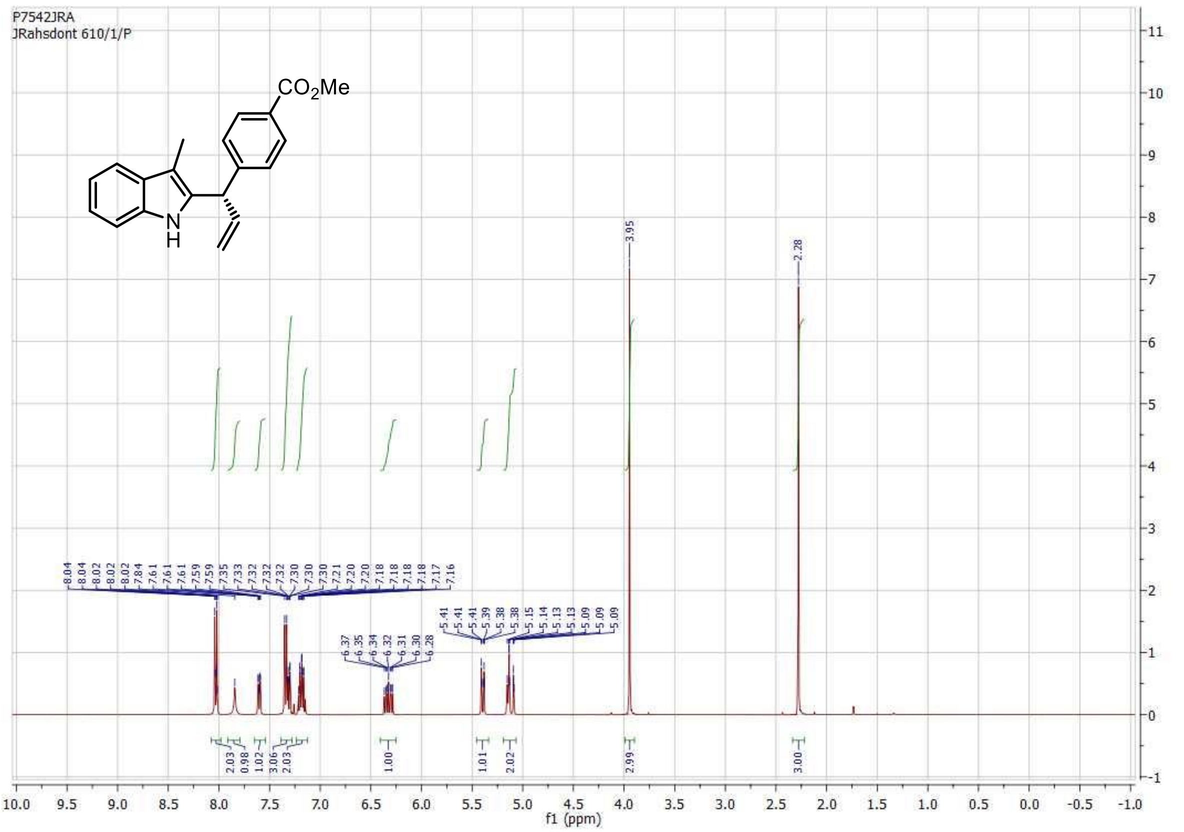

P7542JRA
JRahsdont 610/1/P
CO2Me
N
H
f1 (ppm)

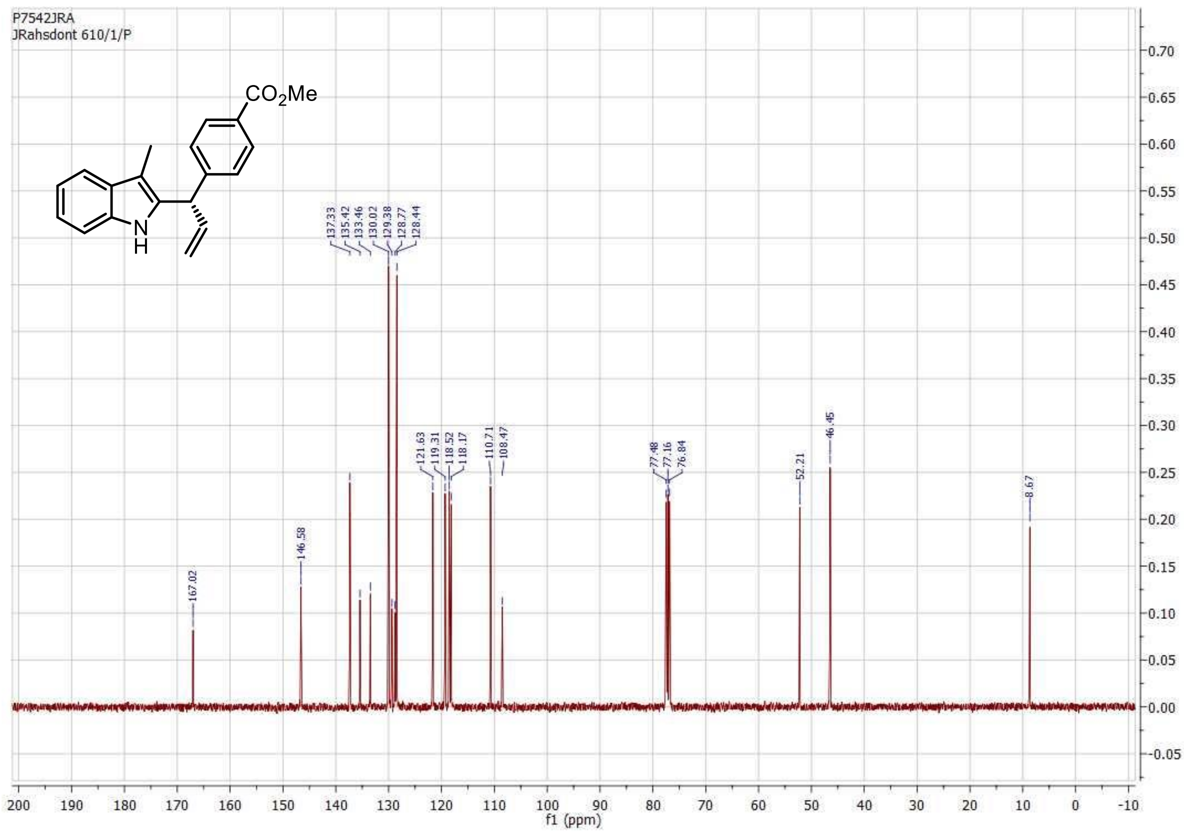

P7542JRA
JRahsdont 610/1/P
CO2Me
N
H
f1 (ppm)

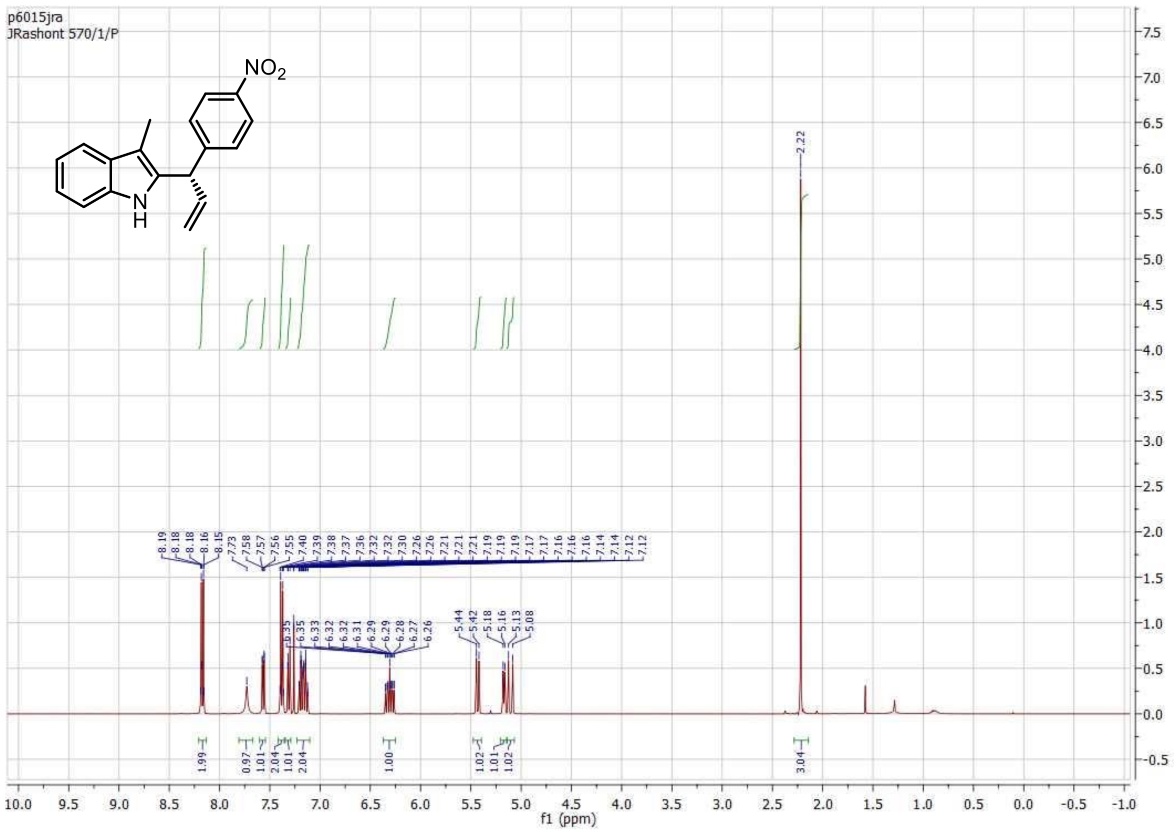
p6015jra
JRashont 570/1/P
f1 (ppm)

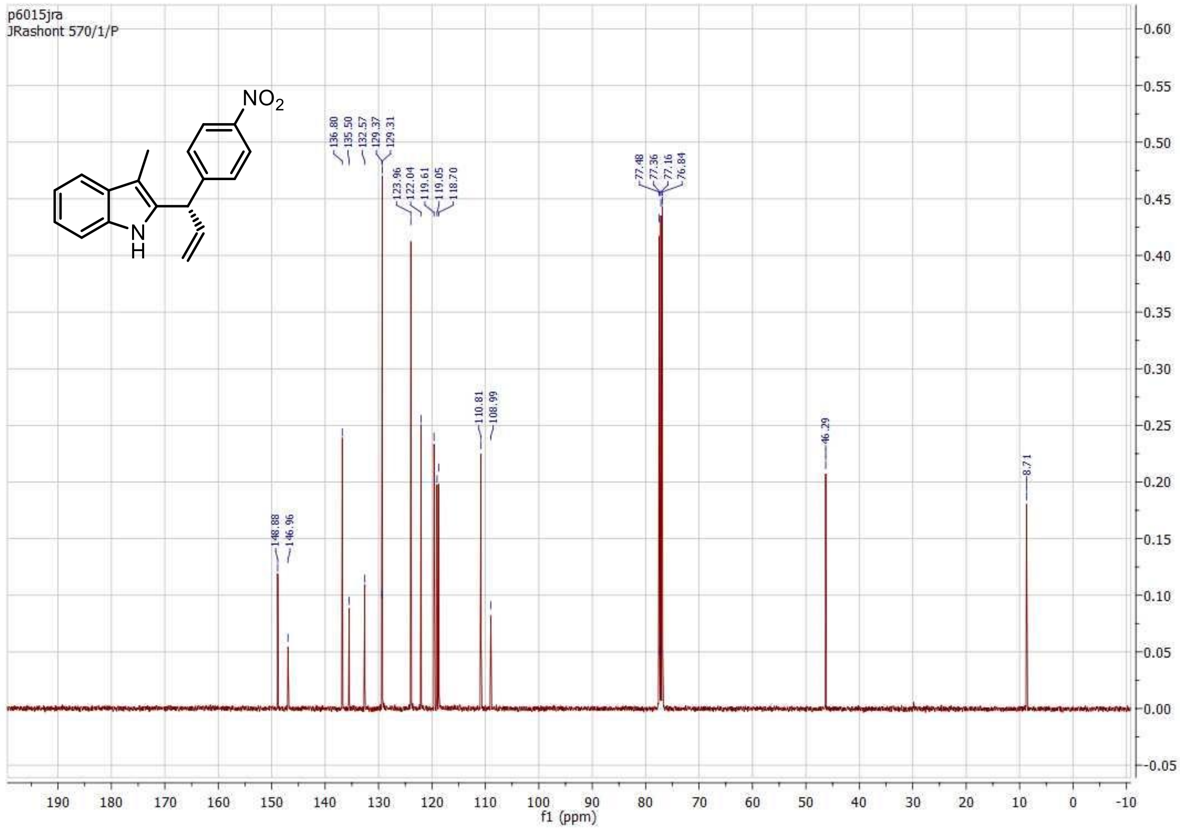
p6015jra
JRashont 570/1/P
f1 (ppm)

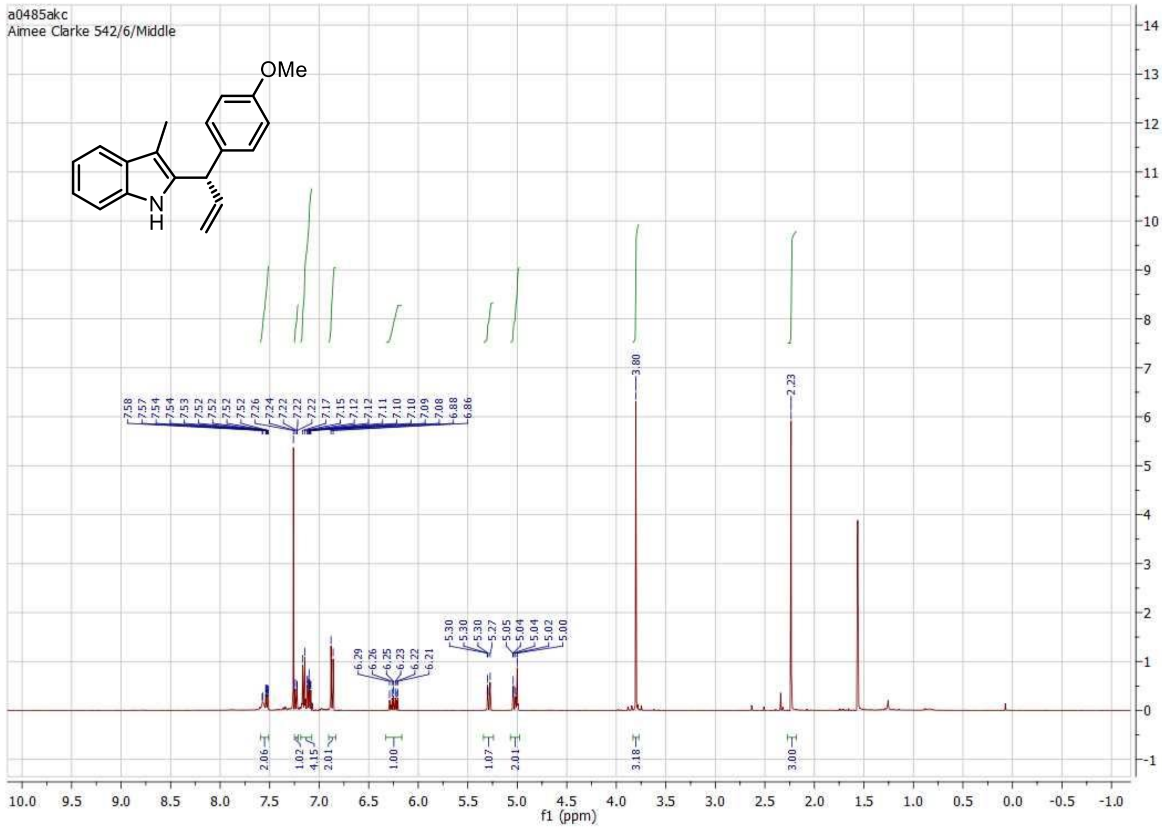
a0485akc
Aimee Clarke 542/6/Middle
OMe
N
H
f1 (ppm)

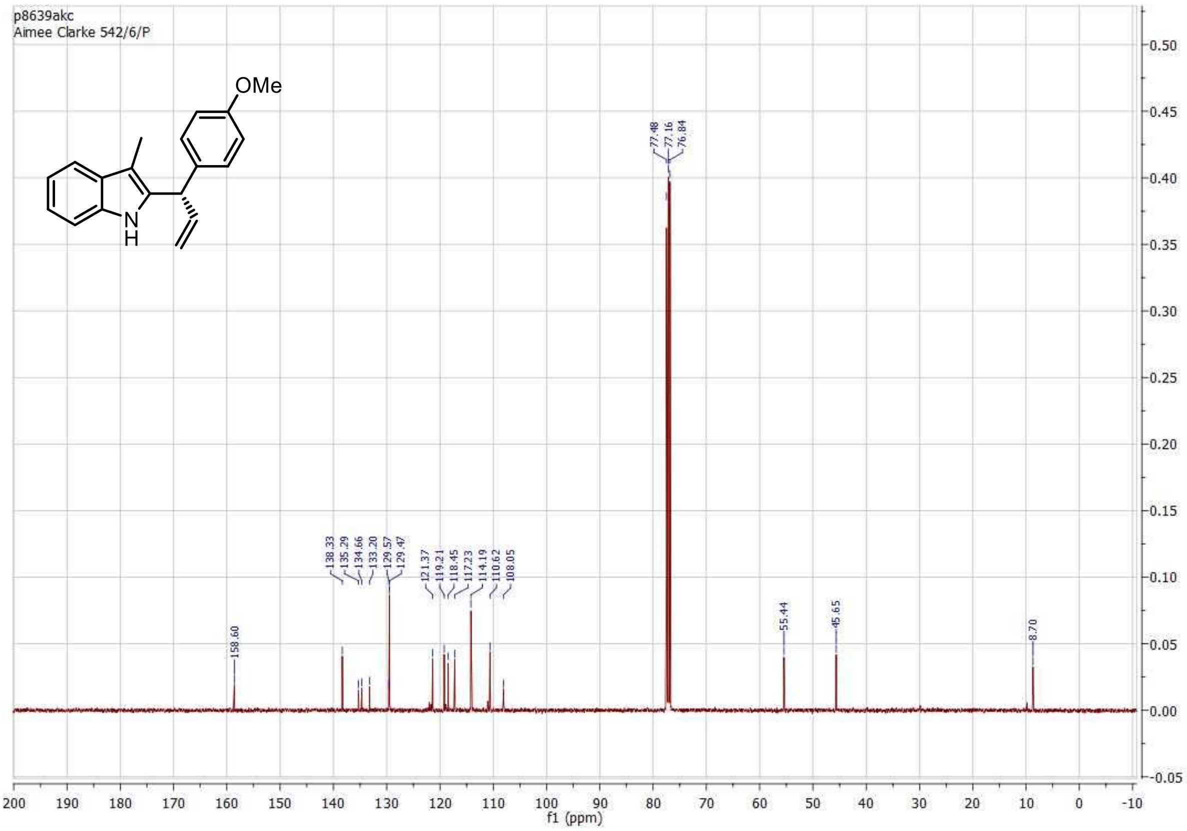
p8639akc
Aimee Clarke 542/6/P
OMe
N
H
f1 (ppm)

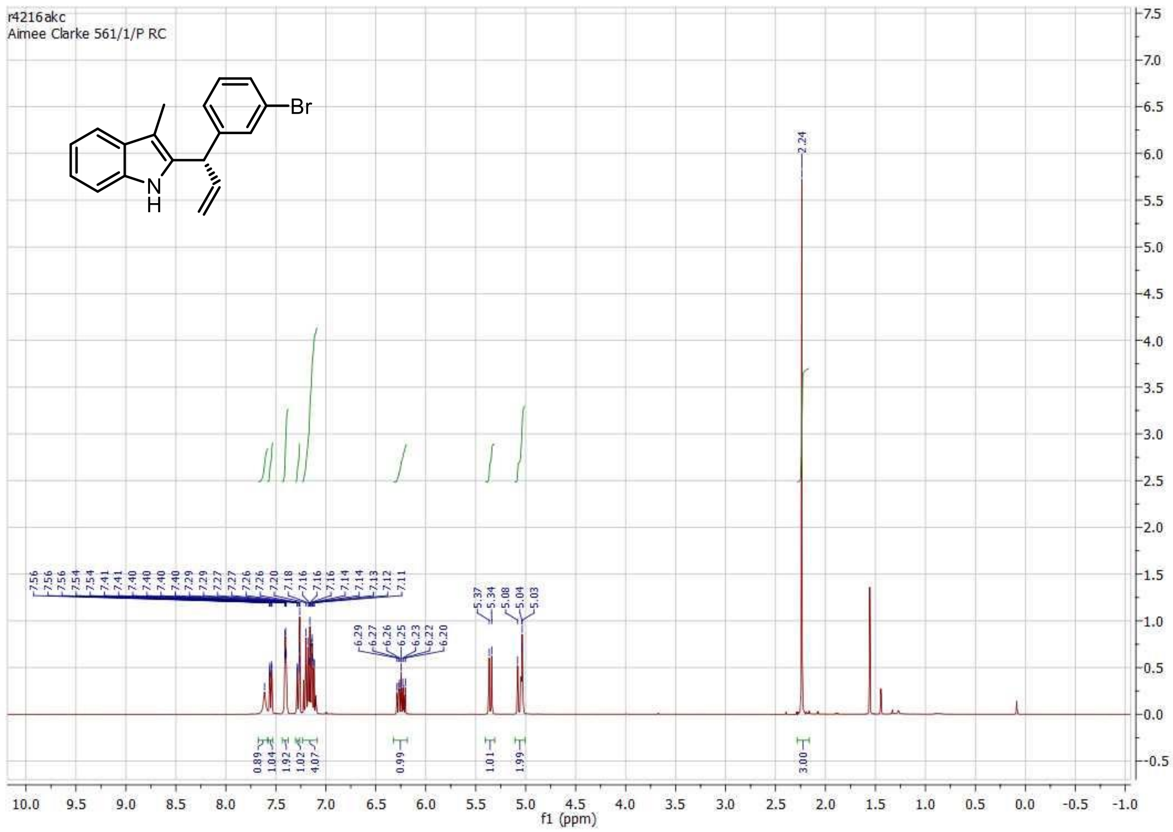
r4216akc
Aimee Clarke 561/1/P RC
Br
N
H
f1 (ppm)

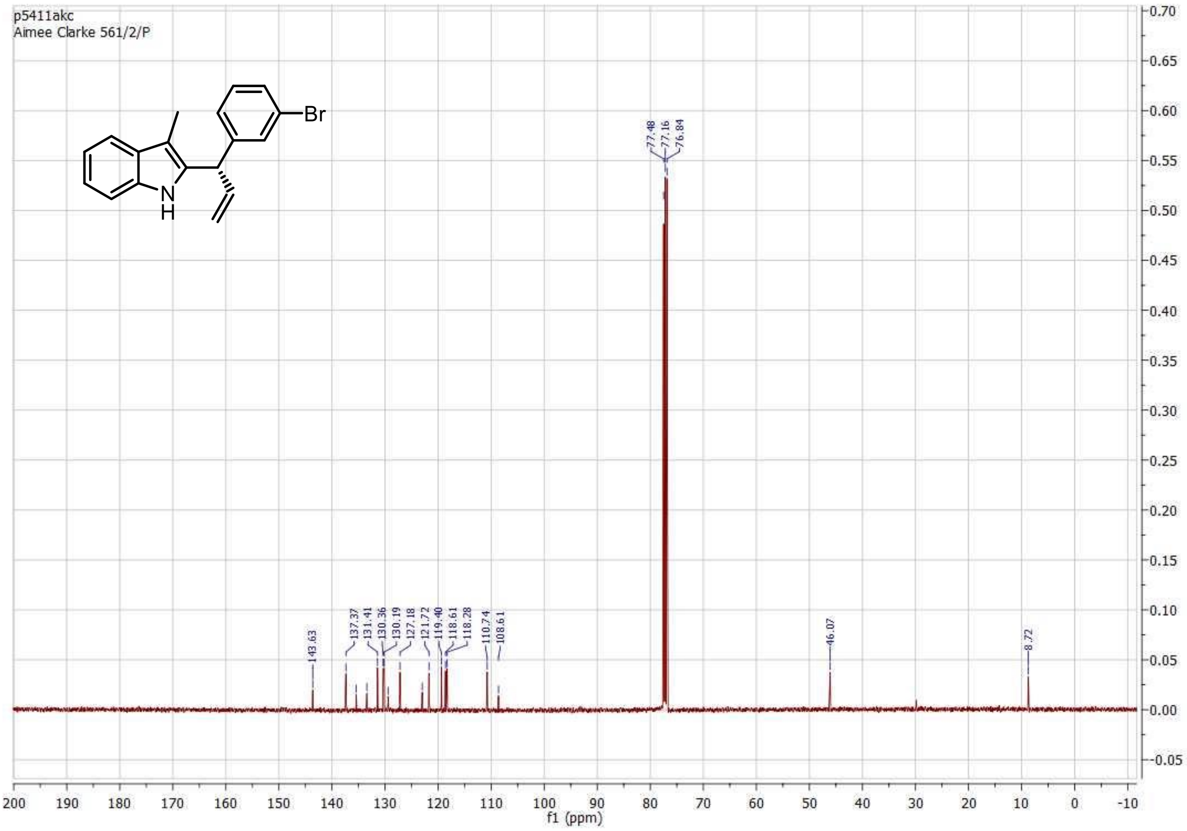
p5411akc
Aimee Clarke 561/2/P
Br
N
H
f1 (ppm)

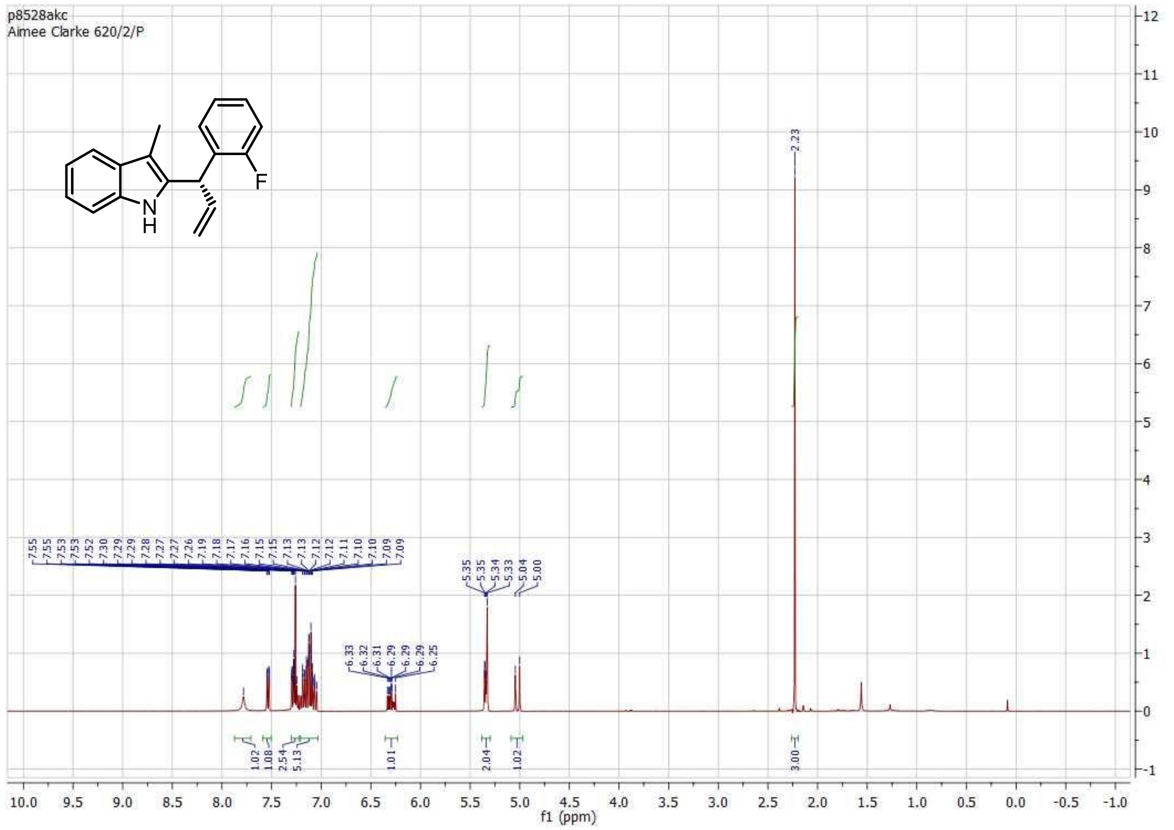
p8528akc
Aimee Clarke 620/2/P
F
N
H
7.55 7.55 7.53 7.53 7.52 7.30 7.29 7.29 7.28 7.27 7.27 7.26 7.19 7.18 7.17 7.16 7.15 7.15 7.13 7.13 7.12 7.12 7.11 7.10 7.09 7.09
6.33 6.32 6.31 6.29 6.29 6.29 6.25
5.35 5.35 5.34 5.33 5.04 5.00
2.23
1.02 1.08 2.54 5.13 1.01 2.04 1.02 3.00
f1 (ppm)

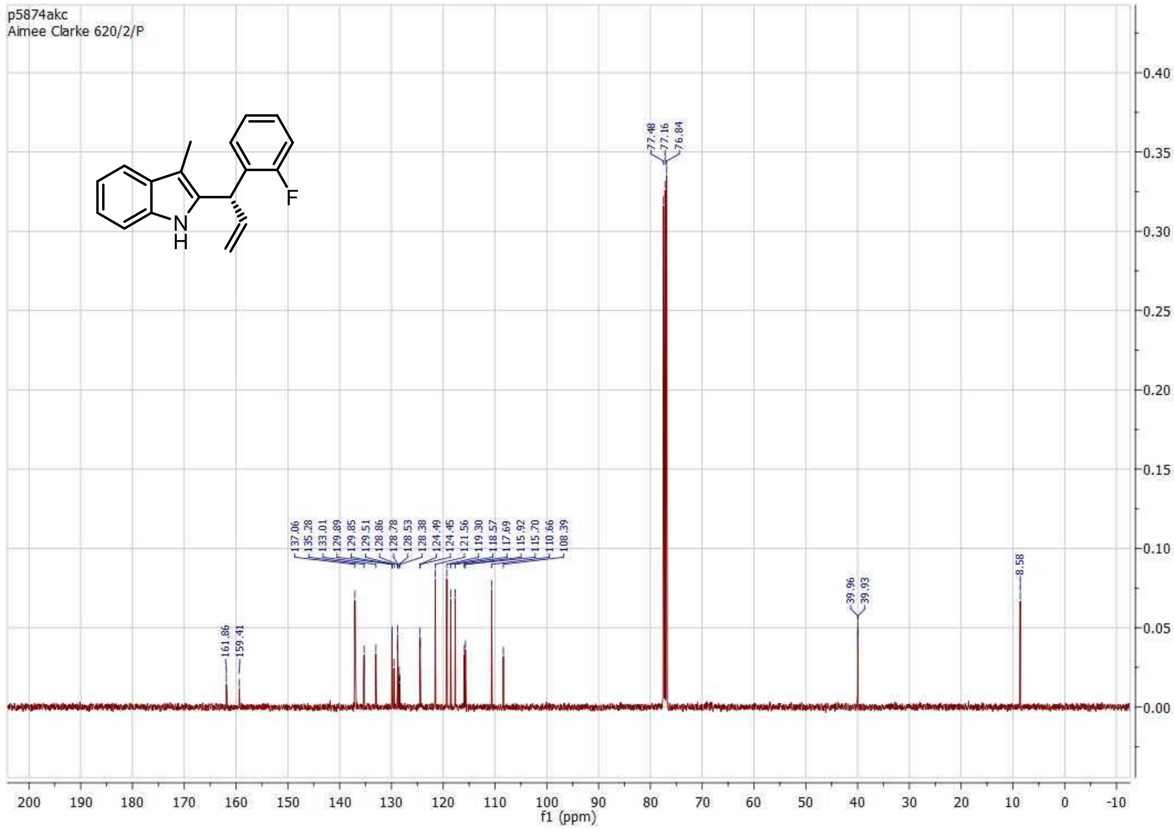
p5874akc
Aimee Clarke 620/2/P
F
N
H
161.86 159.41
137.06 135.28 133.01 129.89 129.85 129.51 128.86 128.78 128.53 128.38 124.49 124.45 121.56 119.30 118.57 117.69 115.92 115.70 110.66 108.39
77.48 77.16 76.84
39.96 39.93
8.58
f1 (ppm)

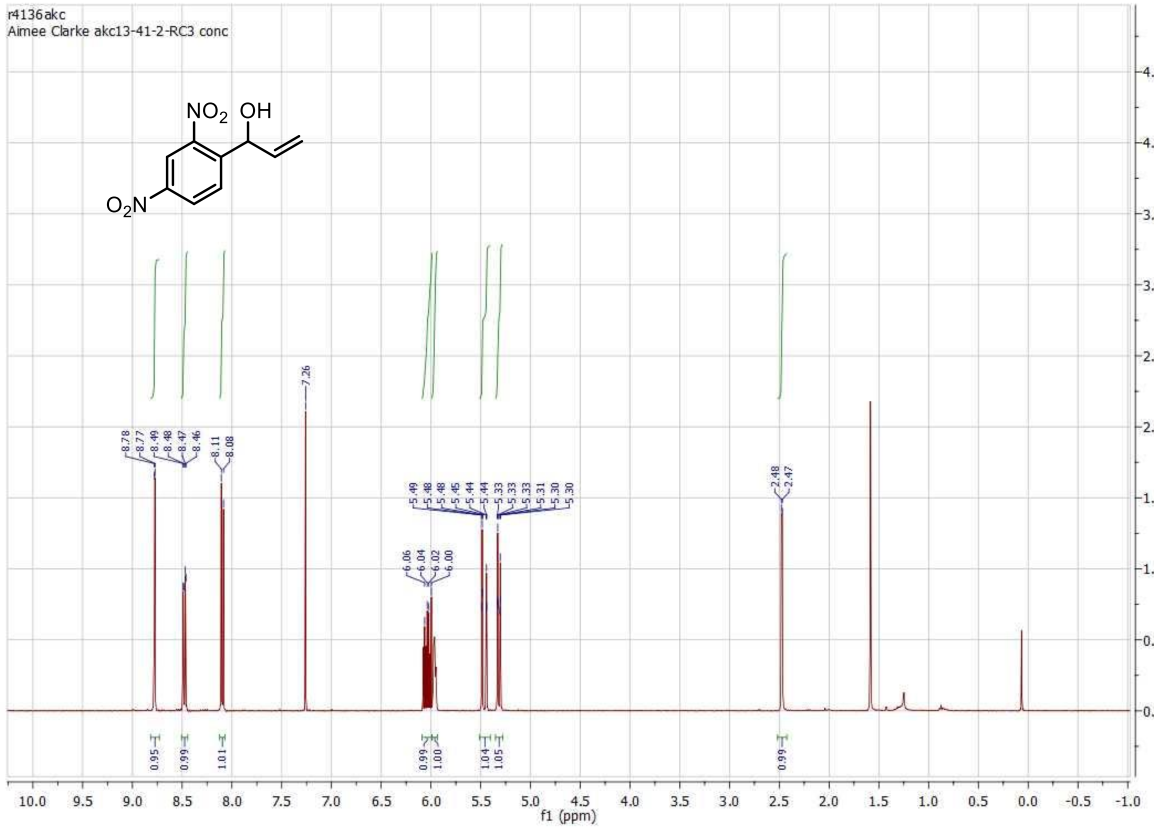
r4136akc
Aimee Clarke akc13-41-2-RC3 conc
NO2 OH
O2N
8.78 8.77 8.49 8.48 8.47 8.46 8.11 8.08
7.26
6.06 6.04 6.02 6.00
5.49 5.48 5.48 5.45 5.44 5.44 5.33 5.33 5.33 5.31 5.30 5.30
2.48 2.47
0.95 0.99 1.01 0.99 1.00 1.04 1.05 0.99
f1 (ppm)

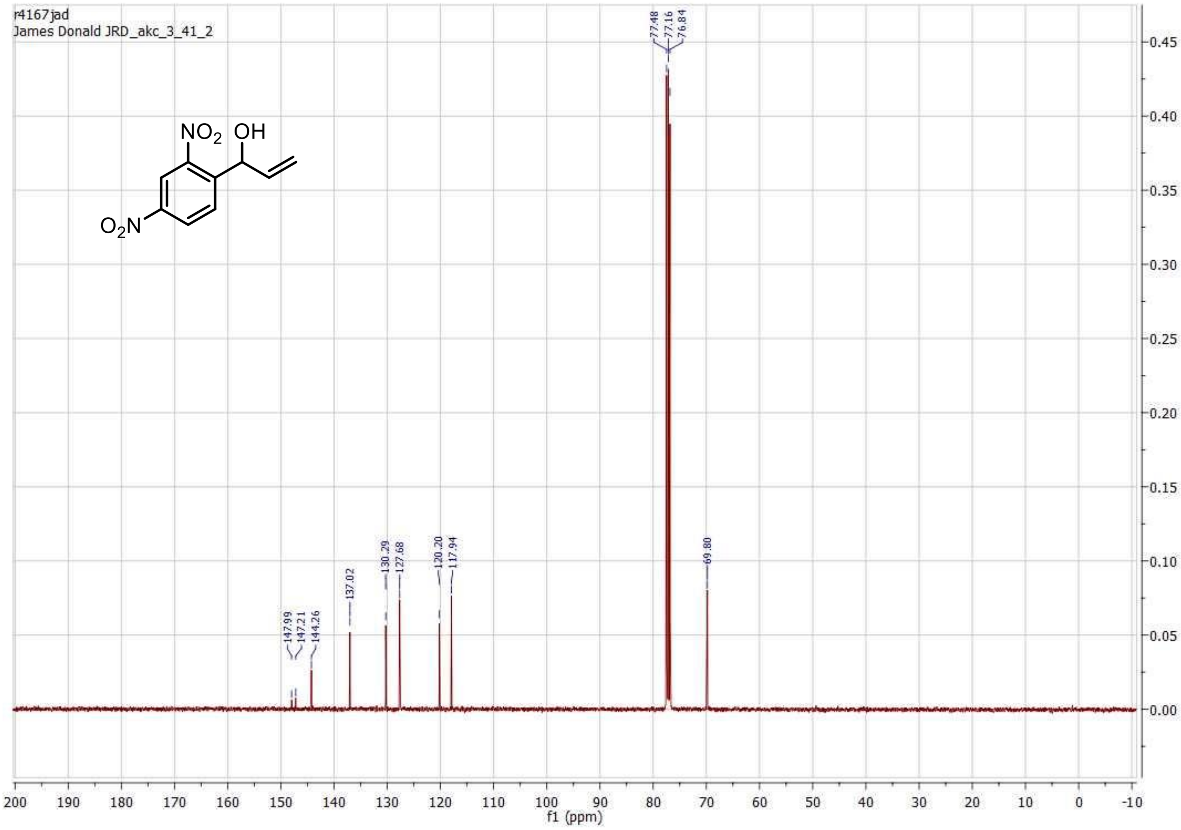
r4167jad
James Donald JRD_akc_3_41_2
NO2 OH
O2N
77.48 77.16 76.84
147.99 147.21 144.26
137.02
130.29 127.68
120.20 117.94
69.80
f1 (ppm)

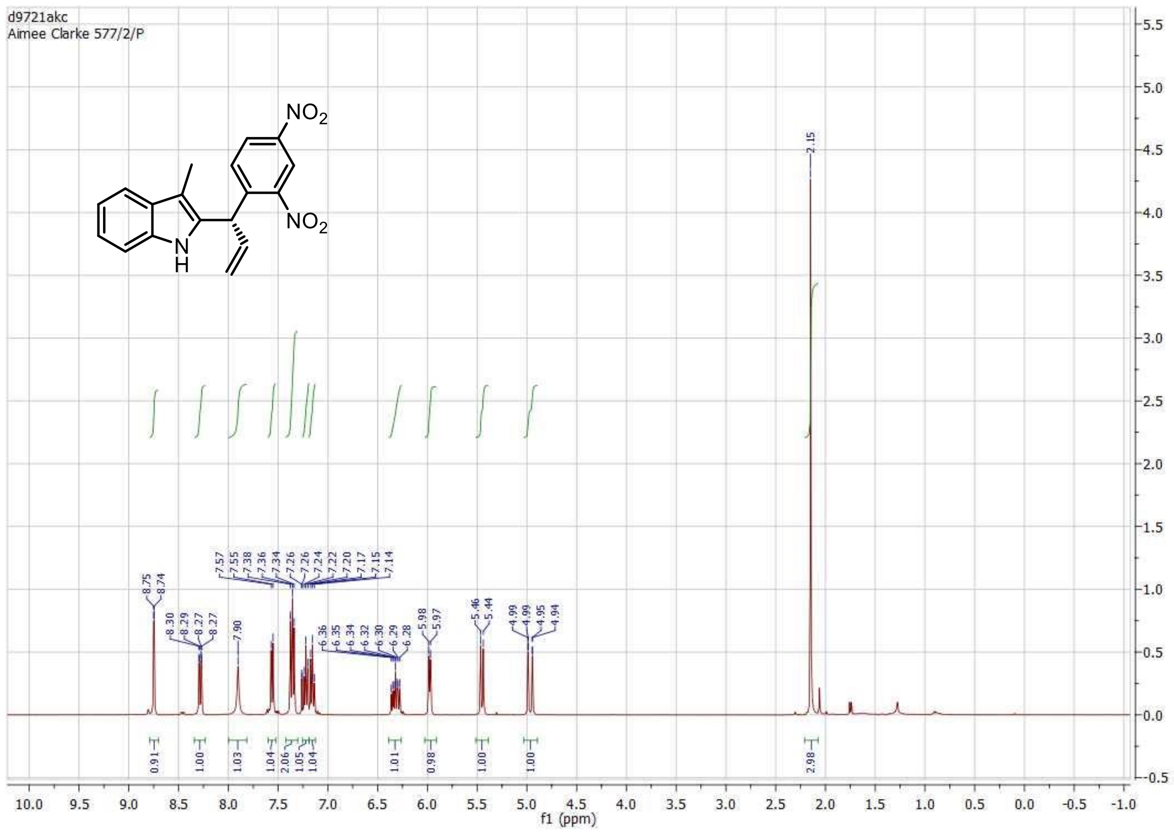
d9721akc
Aimee Clarke 577/2/P
f1 (ppm)

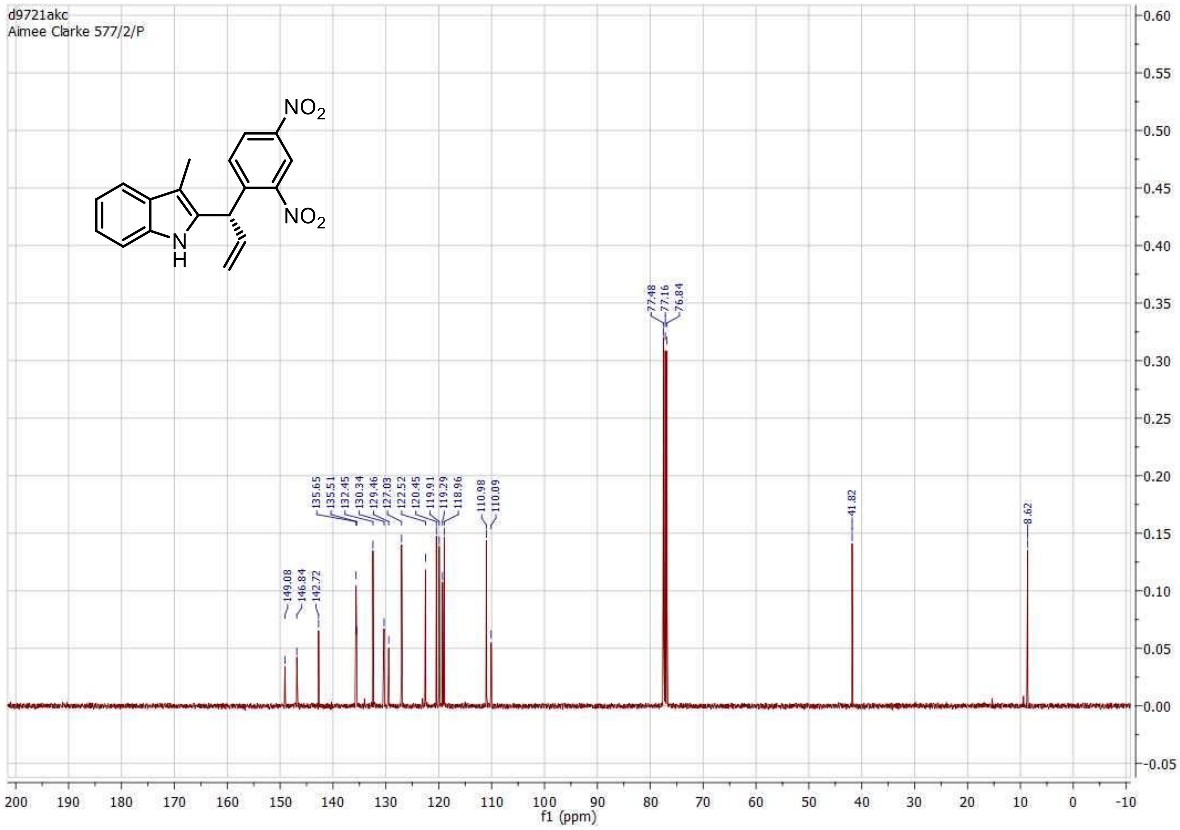
d9721akc
Aimee Clarke 577/2/P
f1 (ppm)

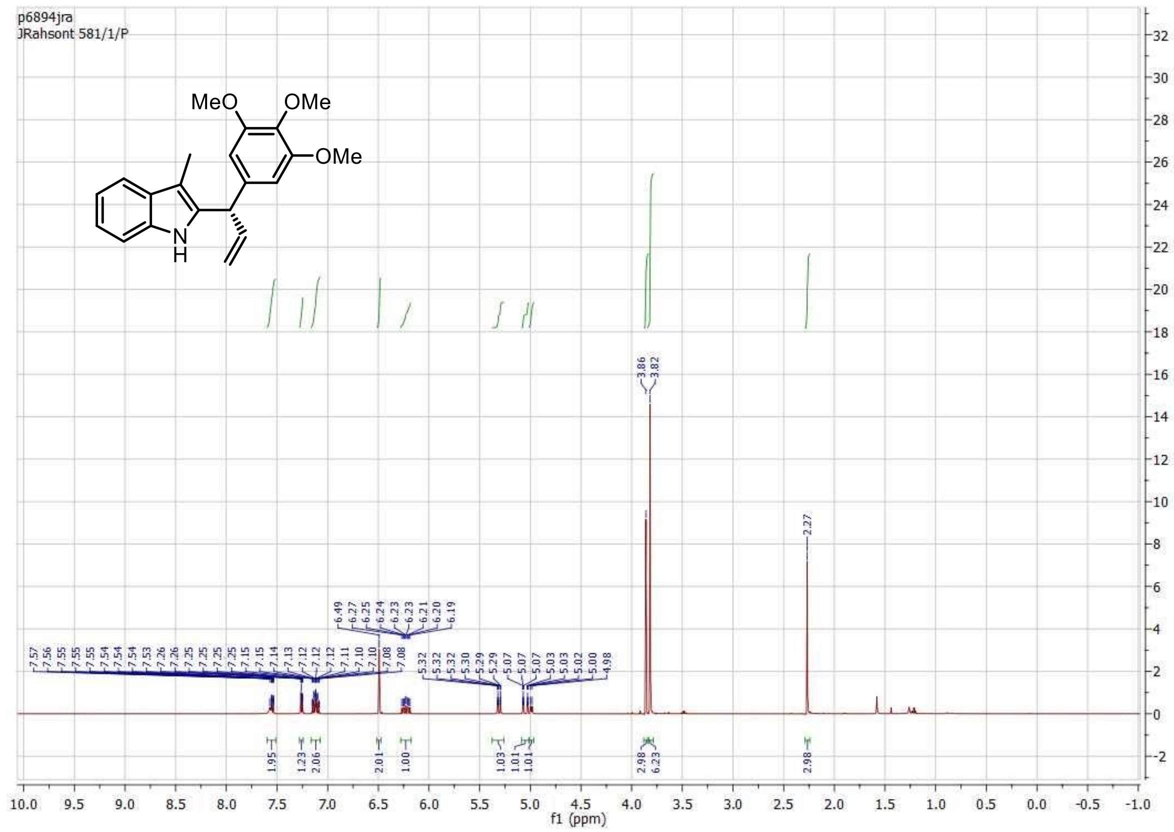
p6894jra
JRahsont 581/1/P
MeO
OMe
OMe
N
H
f1 (ppm)

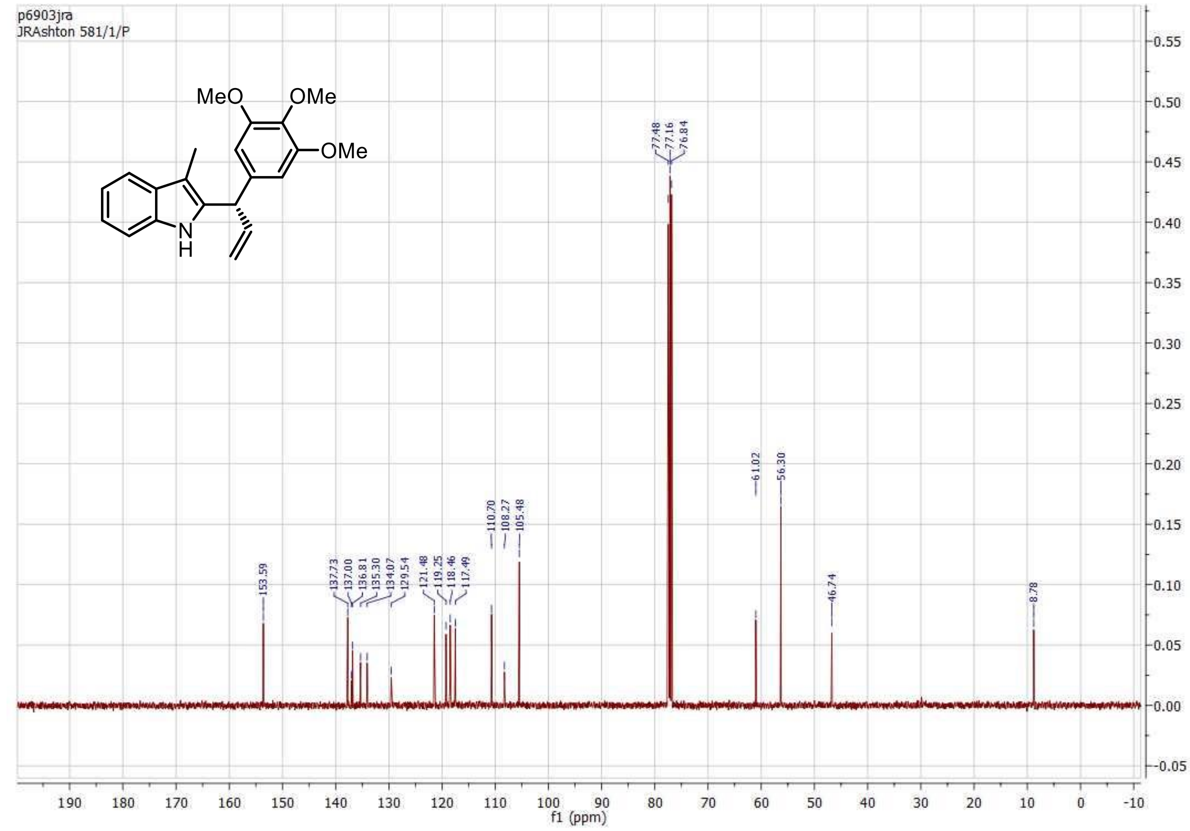
p6903jra
JRAshton 581/1/P
MeO
OMe
OMe
N
H
f1 (ppm)

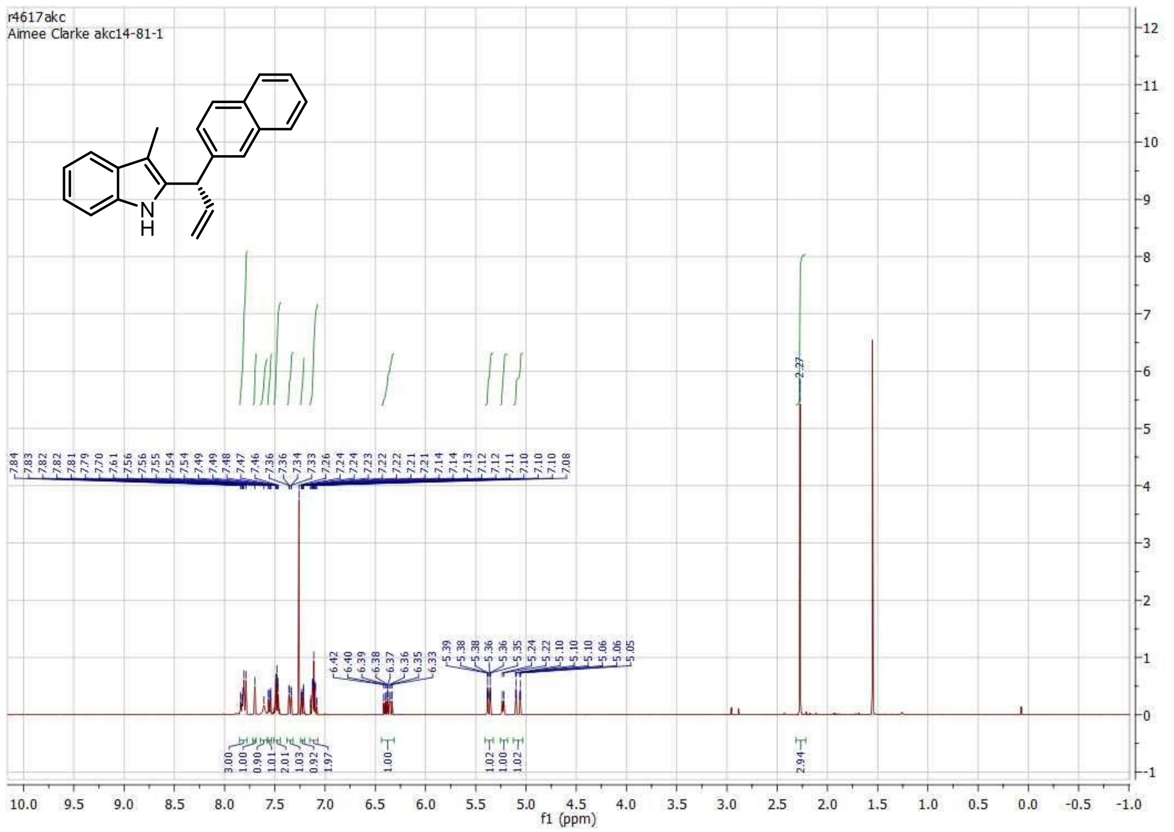

r4617akc
Aimee Clarke akc14-81-1
f1 (ppm)

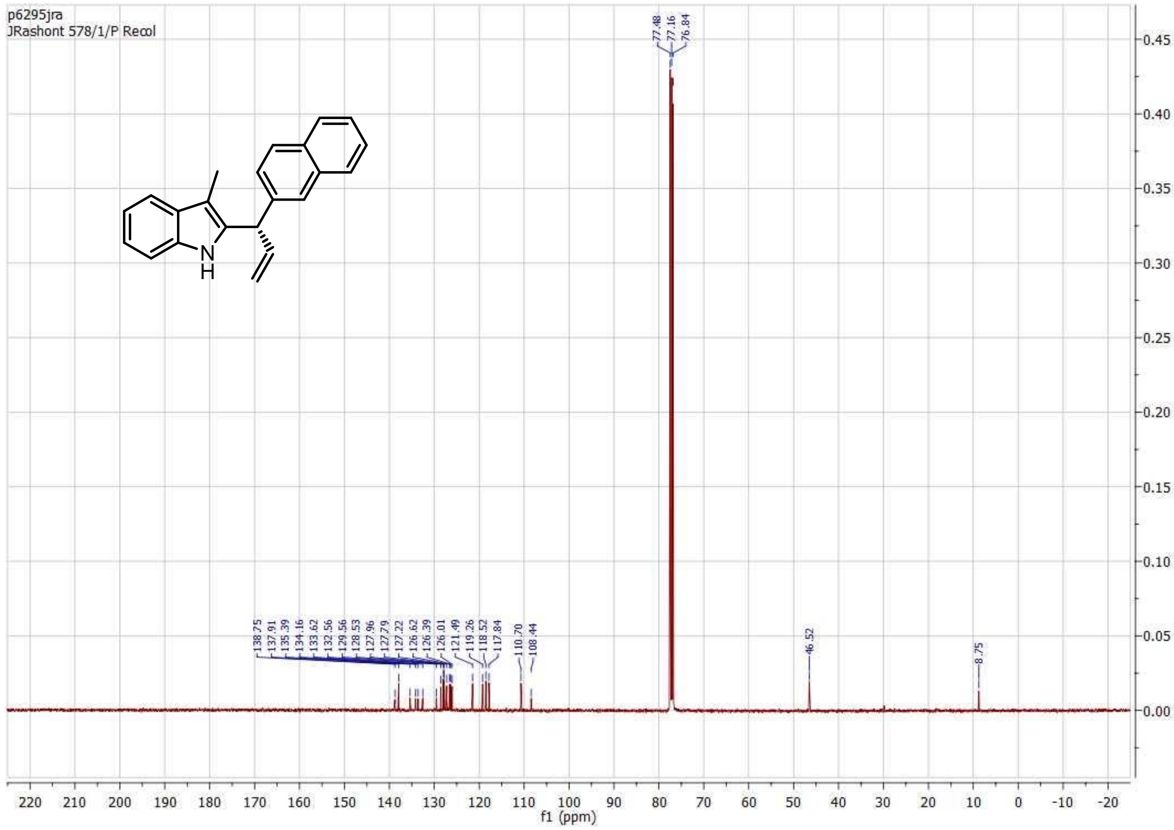

p6295jra
JRashont 578/1/P Recol
f1 (ppm)

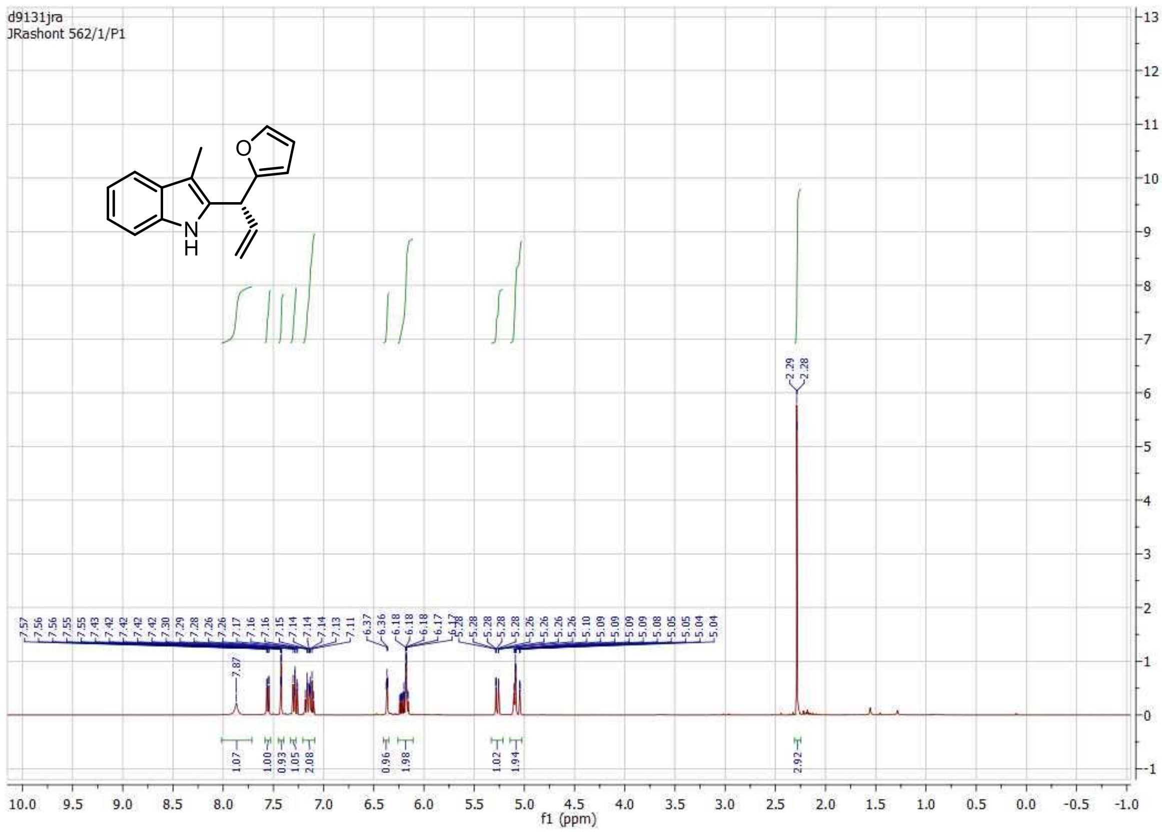
d9131jra
JRashont 562/1/P1
f1 (ppm)

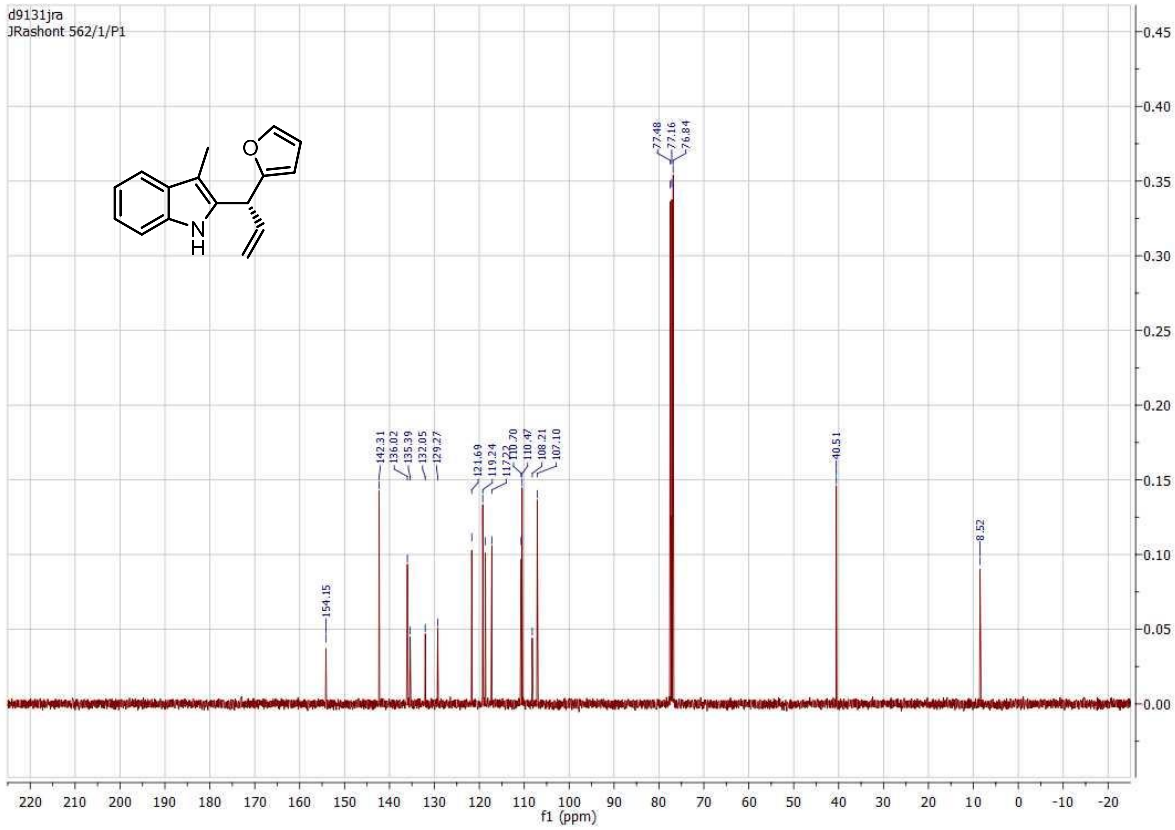
d9131jra
JRashont 562/1/P1
154.15
142.31
136.02
135.39
132.05
129.27
121.69
119.24
110.70
110.47
108.21
107.10
77.48
77.16
76.84
40.51
8.52
f1 (ppm)

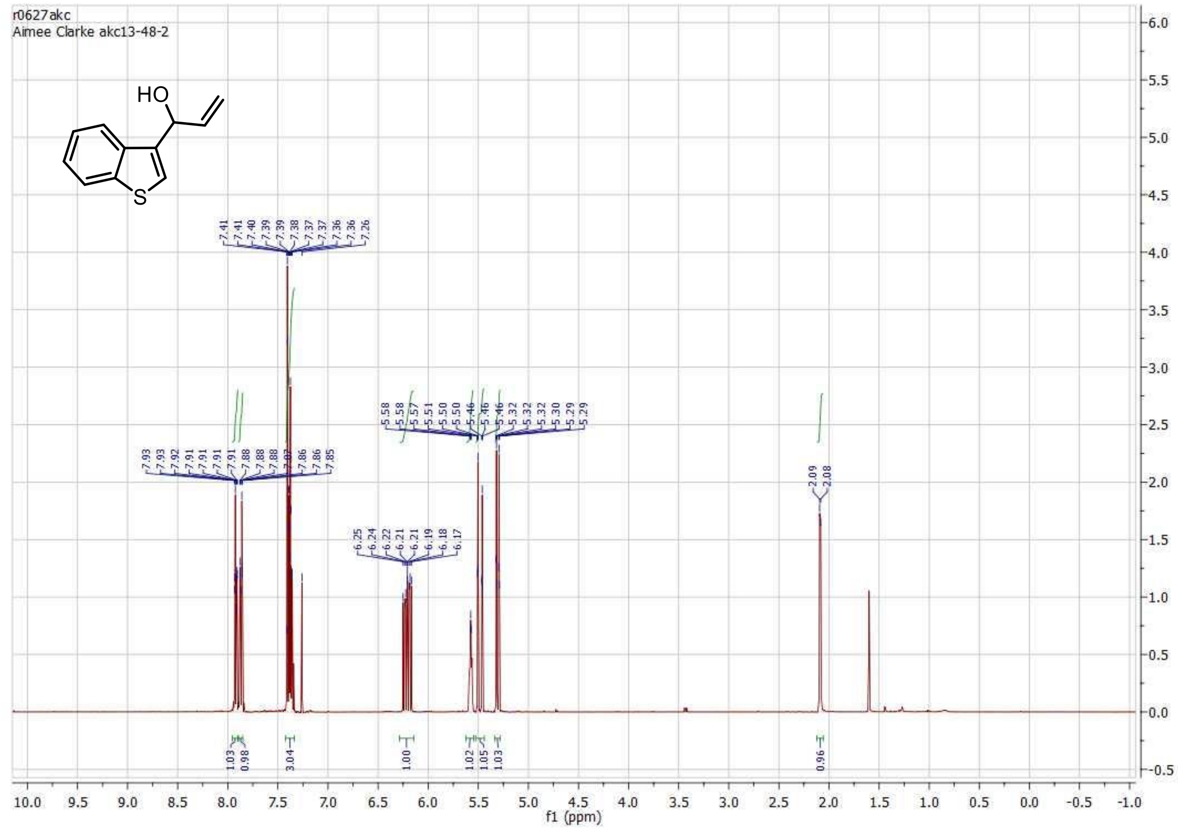
r0627akc
Aimee Clarke akc13-48-2
HO
S
7.41 7.41 7.40 7.39 7.39 7.38 7.37 7.37 7.36 7.36 7.26
7.93 7.93 7.92 7.91 7.91 7.91 7.91 7.88 7.88 7.88 7.86 7.86 7.85
5.58 5.58 5.57 5.51 5.50 5.50 5.46 5.46 5.46 5.32 5.32 5.32 5.30 5.29 5.29
6.25 6.24 6.22 6.21 6.21 6.19 6.18 6.17
2.09 2.08
1.03 0.98 3.04 1.00 1.02 1.05 1.03 0.96
f1 (ppm)

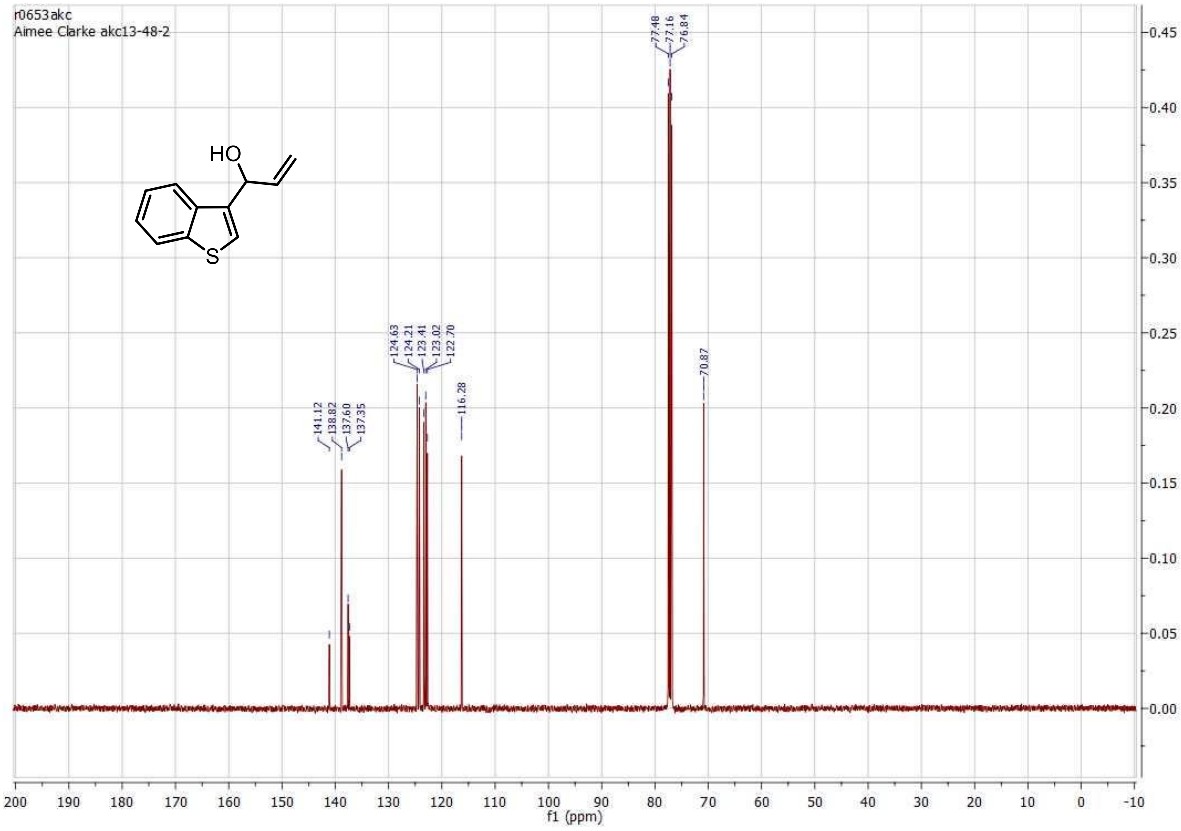
r0653akc
Aimee Clarke akc13-48-2
HO
S
77.48 77.16 76.84
124.63 124.21 123.41 123.02 122.70
70.87
116.28
141.12 138.82 137.60 137.35
f1 (ppm)

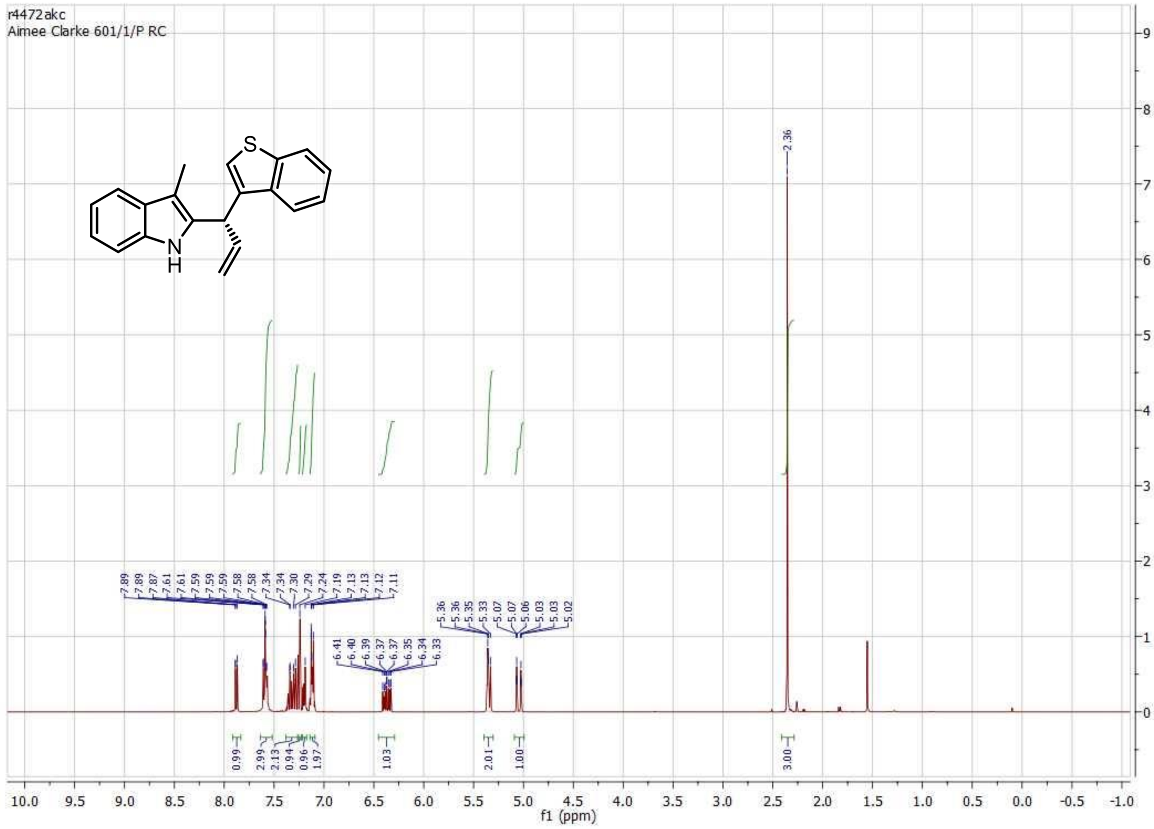
r4472akc
Aimee Clarke 601/1/P RC
S
N
H
7.89
7.89
7.87
7.61
7.59
7.59
7.59
7.58
7.58
7.34
7.34
7.30
7.29
7.24
7.19
7.13
7.13
7.12
7.11
6.41
6.40
6.39
6.37
6.37
6.35
6.34
6.33
5.36
5.36
5.35
5.33
5.07
5.07
5.06
5.03
5.02
2.36
0.99
2.99
2.13
0.94
0.96
1.97
1.03
2.01
1.00
3.00
f1 (ppm)

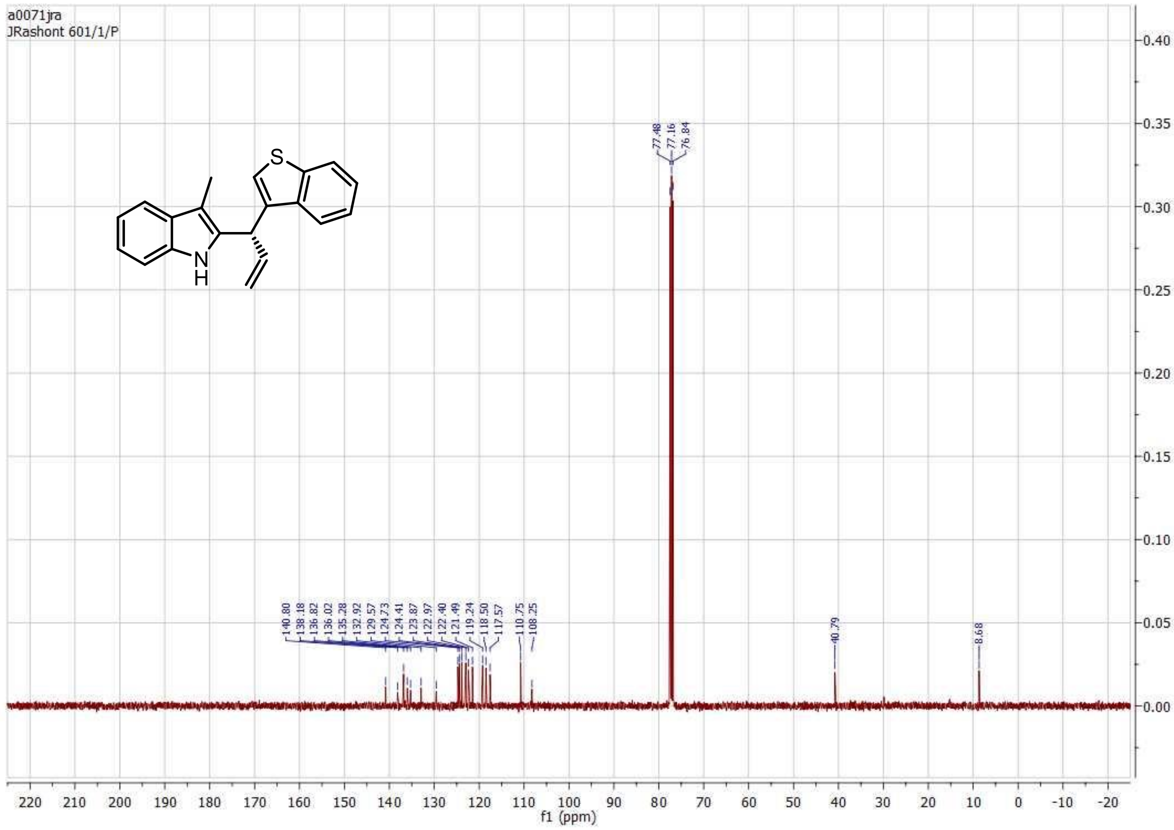
a0071jra
JRashont 601/1/P
S
N
H
77.48
77.16
76.84
140.80
138.18
136.82
136.02
135.28
132.92
129.57
124.73
124.41
123.87
122.97
122.40
121.49
119.24
118.50
117.57
110.75
108.25
40.79
8.68
f1 (ppm)

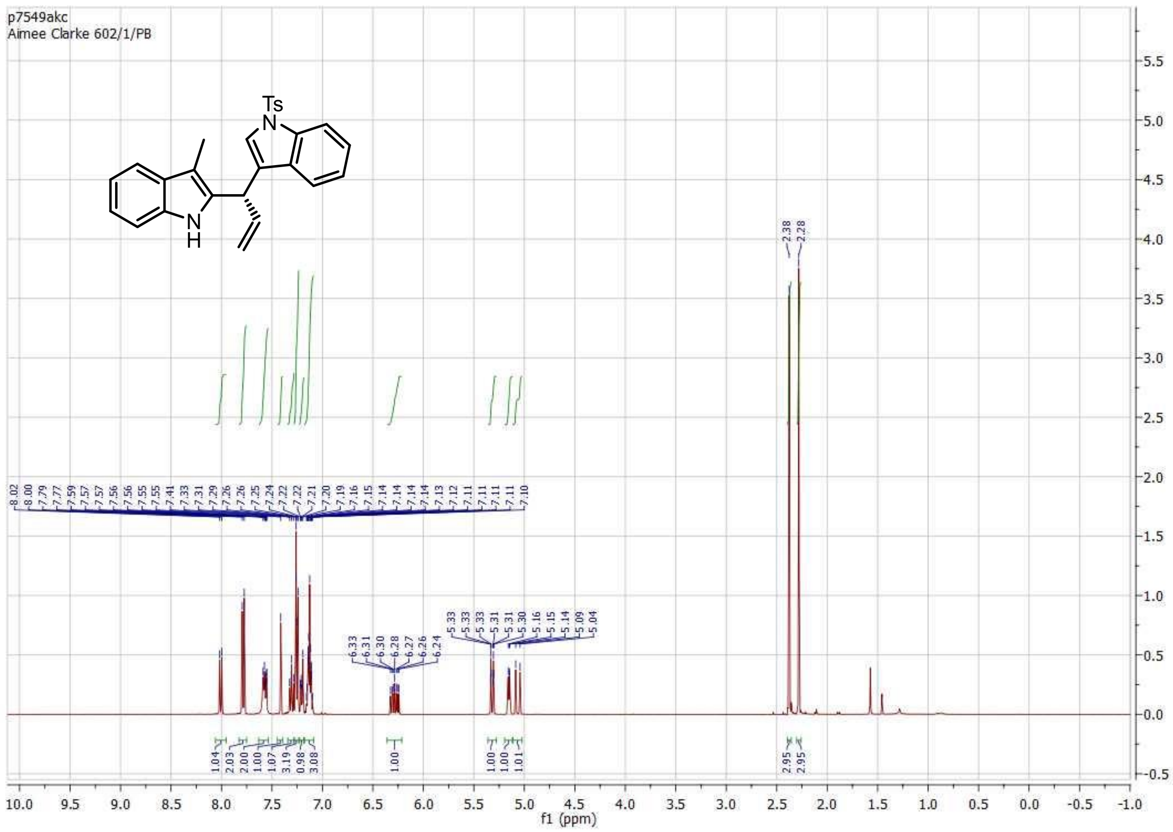
p7549akc
Aimee Clarke 602/1/PB
Ts
N
N
H
f1 (ppm)

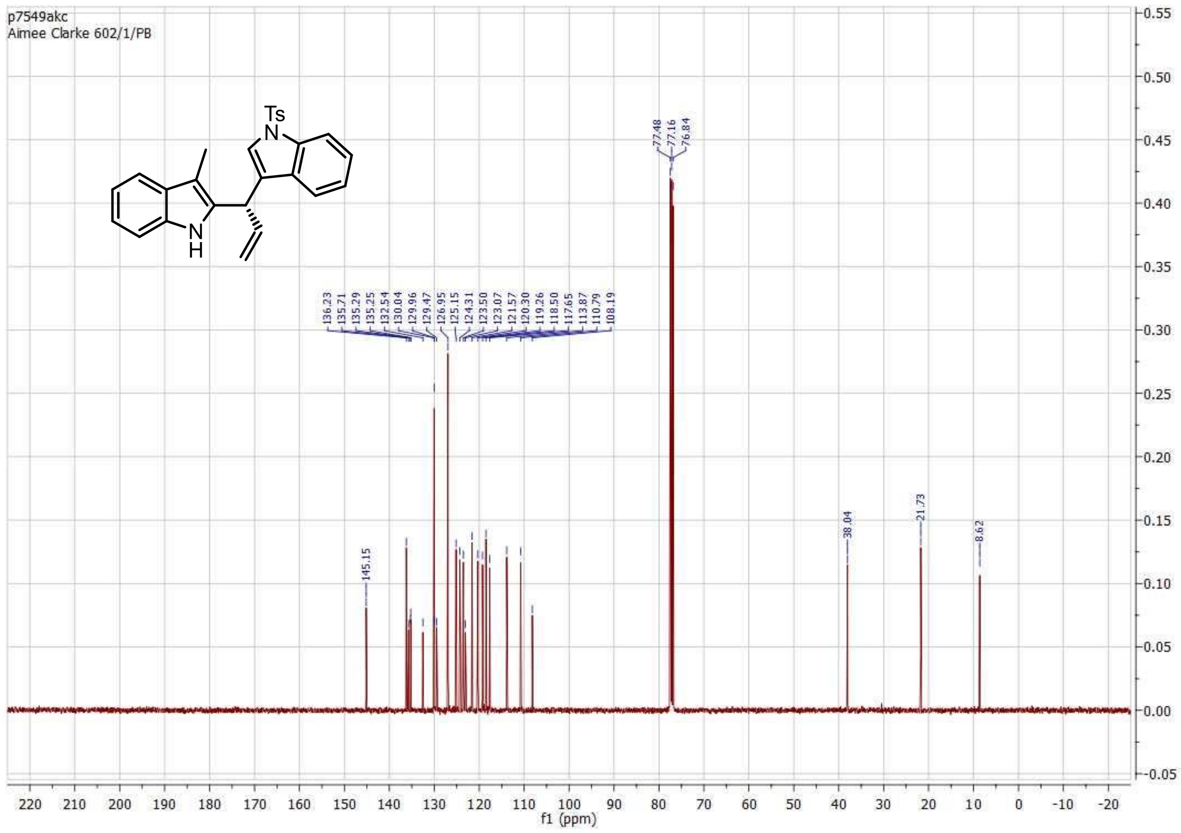
p7549akc
Aimee Clarke 602/1/PB
Ts
N
N
H
f1 (ppm)

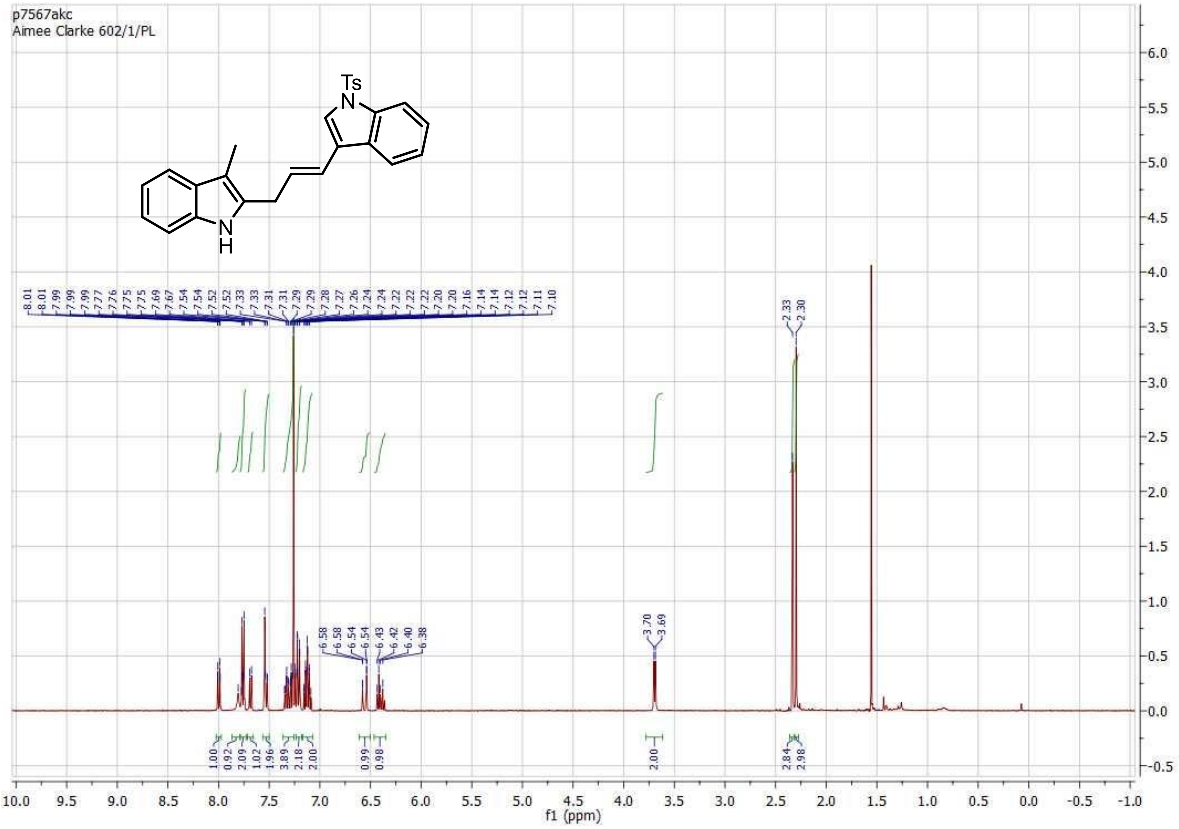
p7567akc
Aimee Clarke 602/1/PL
Ts
N
H
8.01 8.01 7.99 7.99 7.99 7.77 7.76 7.75 7.75 7.69 7.67 7.54 7.54 7.54 7.52 7.52 7.33 7.33 7.31 7.31 7.29 7.29 7.28 7.27 7.26 7.24 7.24 7.22 7.22 7.22 7.22 7.20 7.20 7.16 7.14 7.14 7.12 7.12 7.11 7.10
6.58 6.58 6.54 6.54 6.43 6.42 6.40 6.38
3.70 3.69
2.33 2.30
1.00 0.92 2.09 1.02 1.96 3.89 2.18 2.00 0.99 0.98 2.00 2.84 2.98
f1 (ppm)

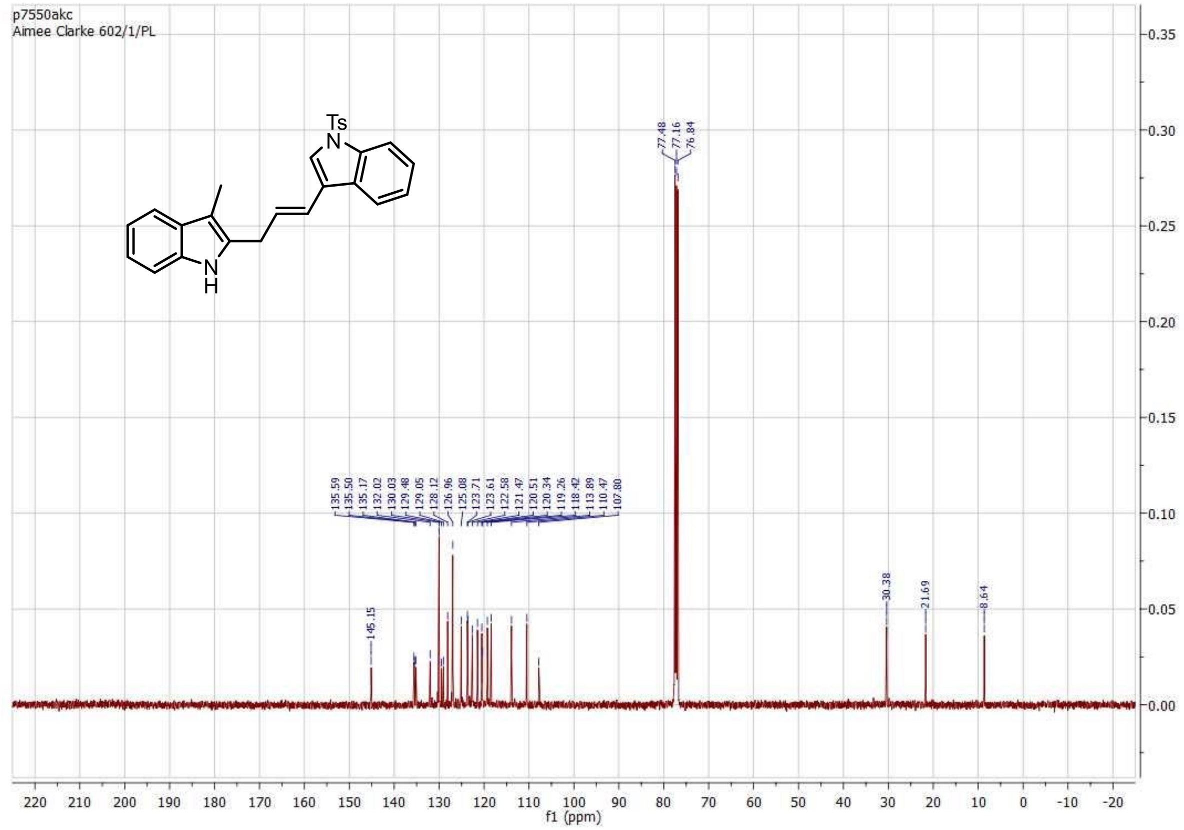
p7550akc
Aimee Clarke 602/1/PL
Ts
N
H
77.48 77.16 76.84
135.59 135.50 135.17 132.02 130.03 129.48 129.05 128.12 126.96 125.08 123.71 123.61 122.58 121.47 120.51 120.34 119.26 118.42 113.89 110.47 107.80
145.15
30.38
21.69
8.64
f1 (ppm)

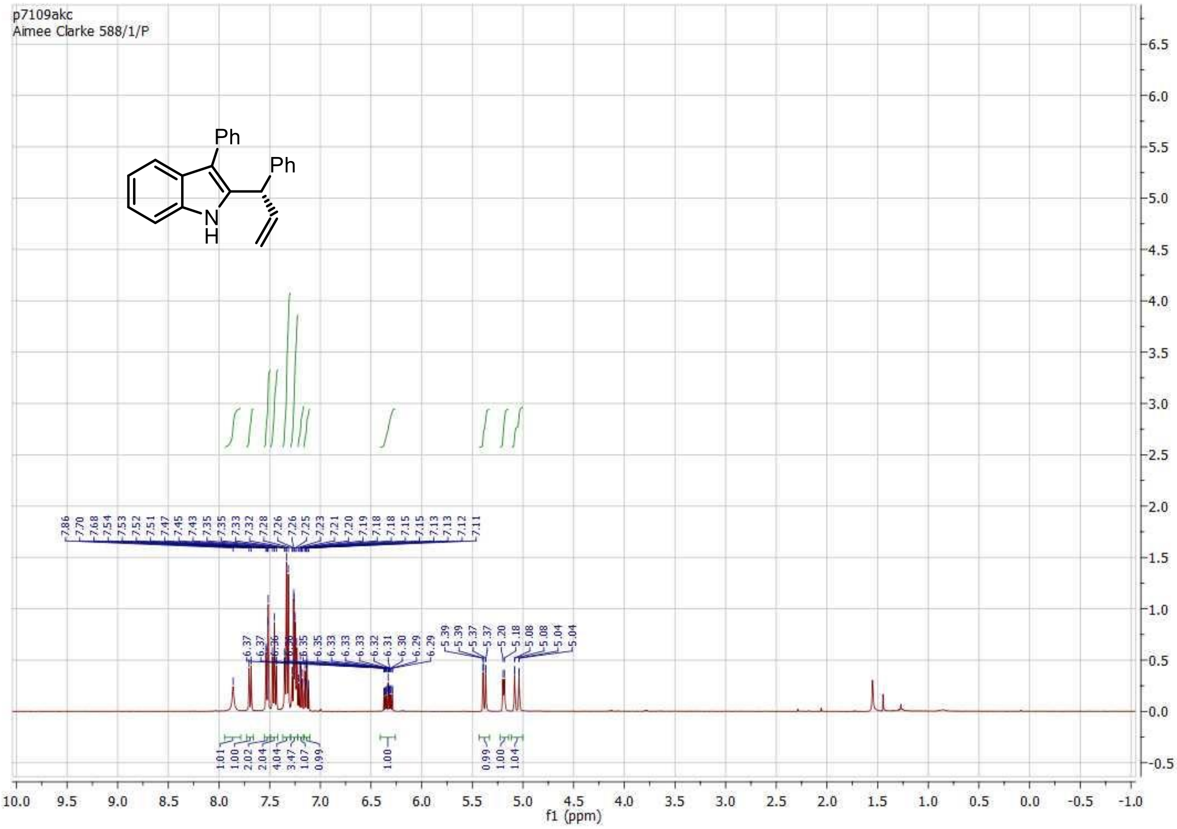
p7109akc
Aimee Clarke 588/1/P
Ph
Ph
N
H
7.86 7.70 7.68 7.54 7.53 7.52 7.51 7.47 7.45 7.43 7.35 7.35 7.33 7.32 7.28 7.26 7.26 7.25 7.23 7.21 7.20 7.19 7.18 7.18 7.15 7.15 7.13 7.13 7.12 7.11
6.37 6.37 6.36 6.35 6.35 6.33 6.33 6.33 6.32 6.31 6.30 6.29 6.29
5.39 5.39 5.37 5.37 5.20 5.18 5.08 5.08 5.04 5.04
1.01 1.00 2.02 2.04 4.04 3.47 1.07 0.99
1.00
0.99 1.00 1.04
f1 (ppm)

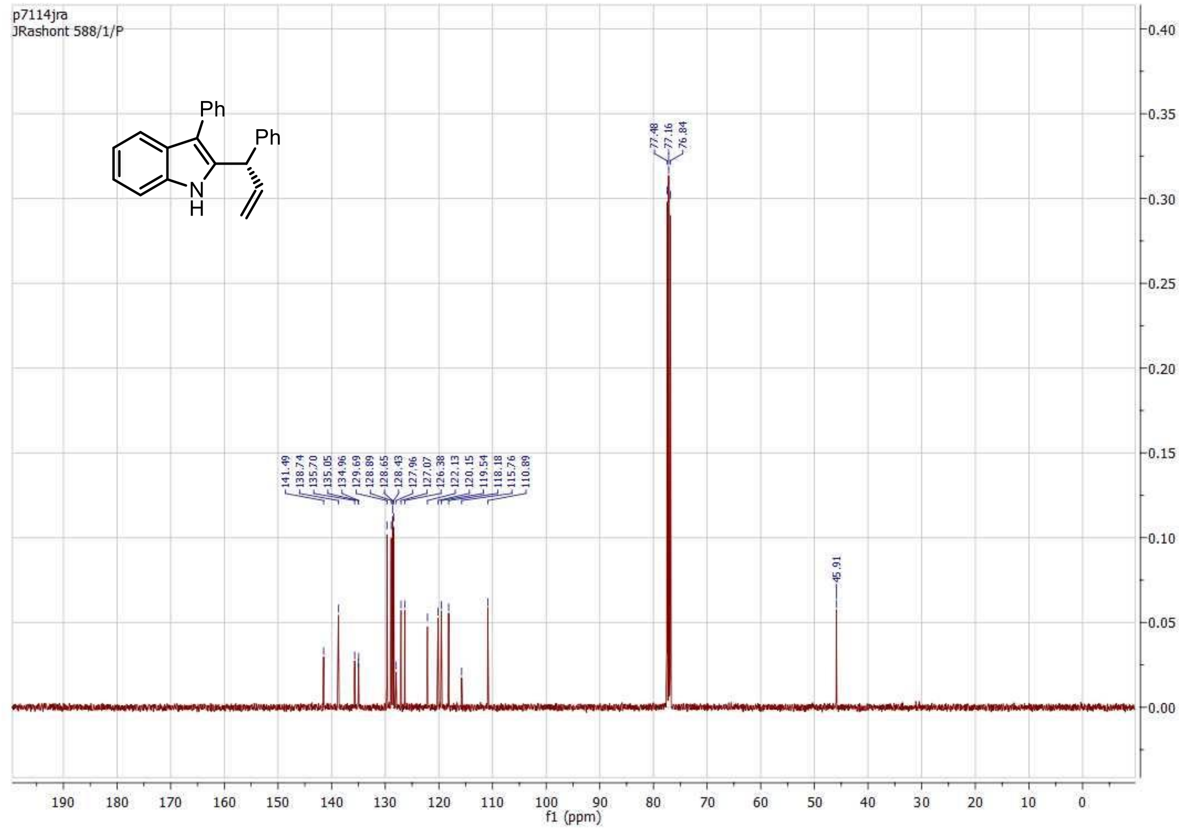
p7114jra
JRashont 588/1/P
Ph
Ph
N
H
141.49 138.74 135.70 135.05 134.96 129.69 128.89 128.65 128.43 127.96 127.07 126.38 122.13 120.15 119.54 118.18 115.76 110.89
77.48 77.16 76.84
45.91
f1 (ppm)

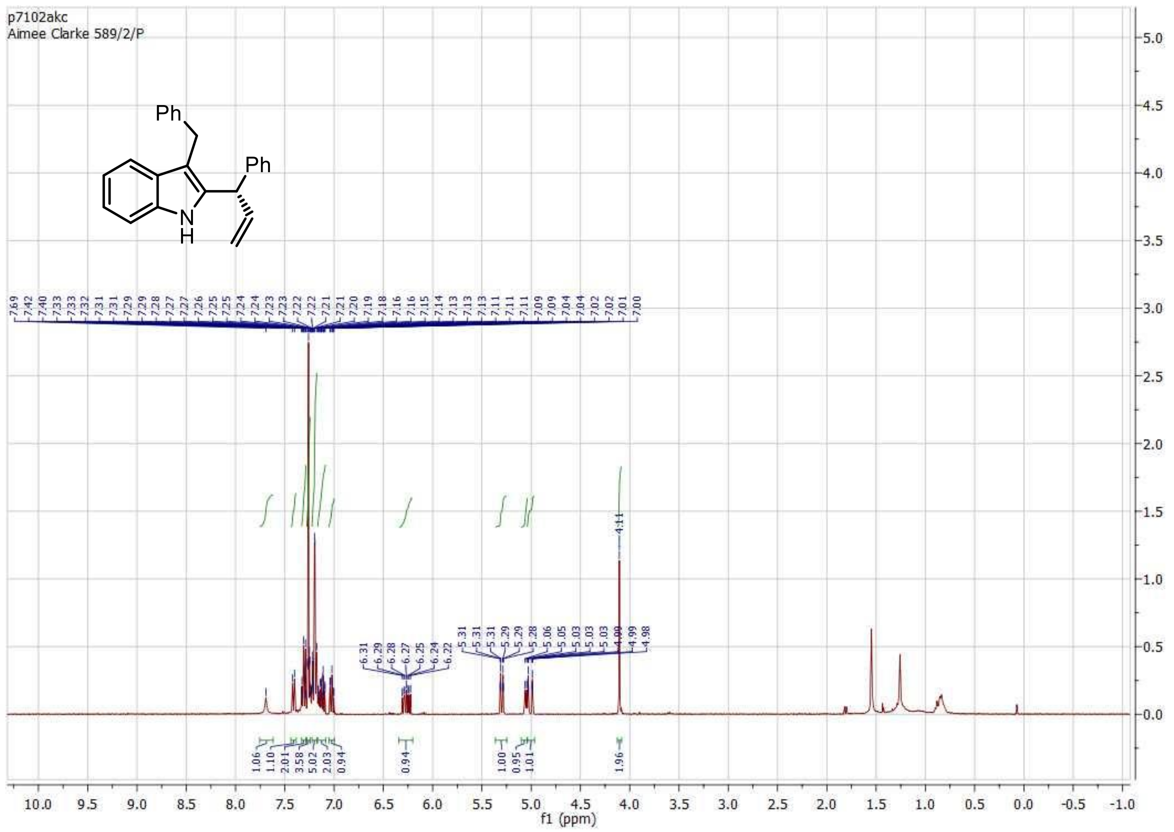
p7102akc
Aimee Clarke 589/2/P
Ph
Ph
N
H
f1 (ppm)

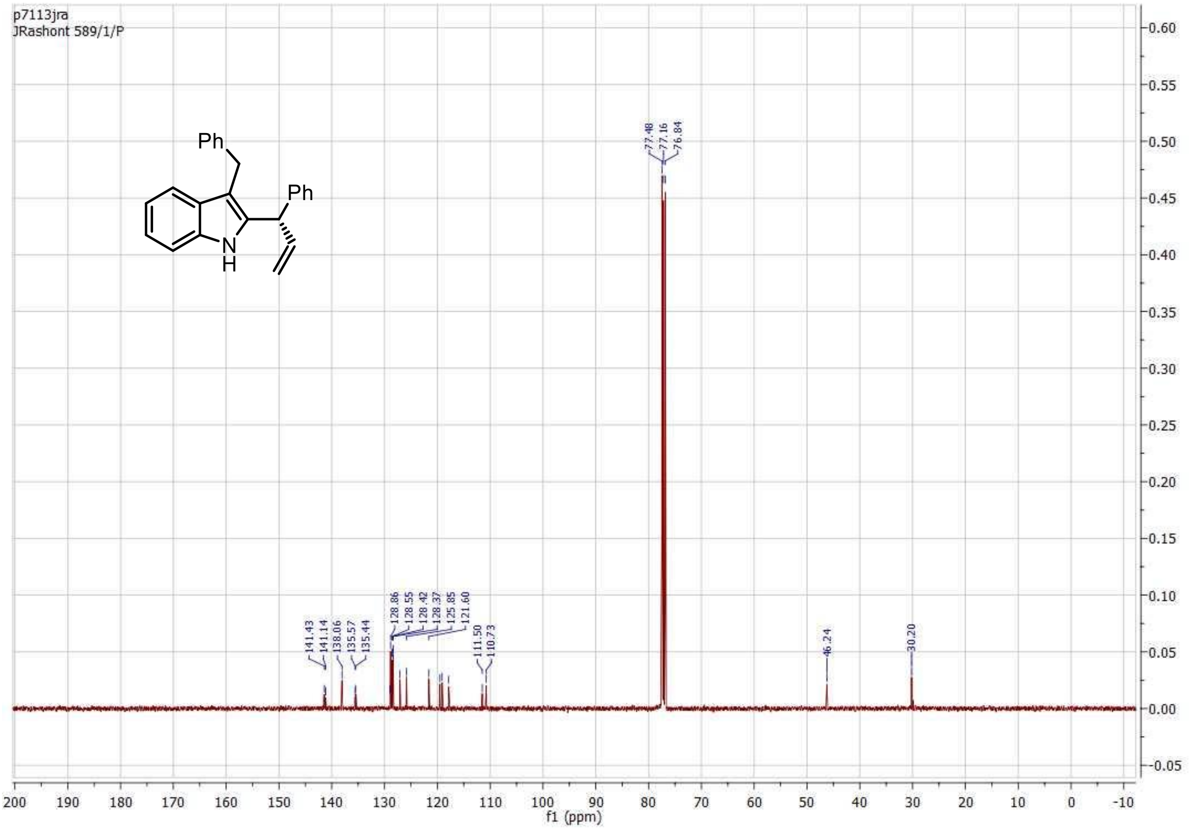
p7113jra
JRashont 589/1/P
Ph
Ph
N
H
141.43
141.14
138.06
135.57
135.44
128.86
128.55
128.42
128.37
125.85
121.60
111.50
110.73
77.48
77.16
76.84
46.24
30.20
f1 (ppm)

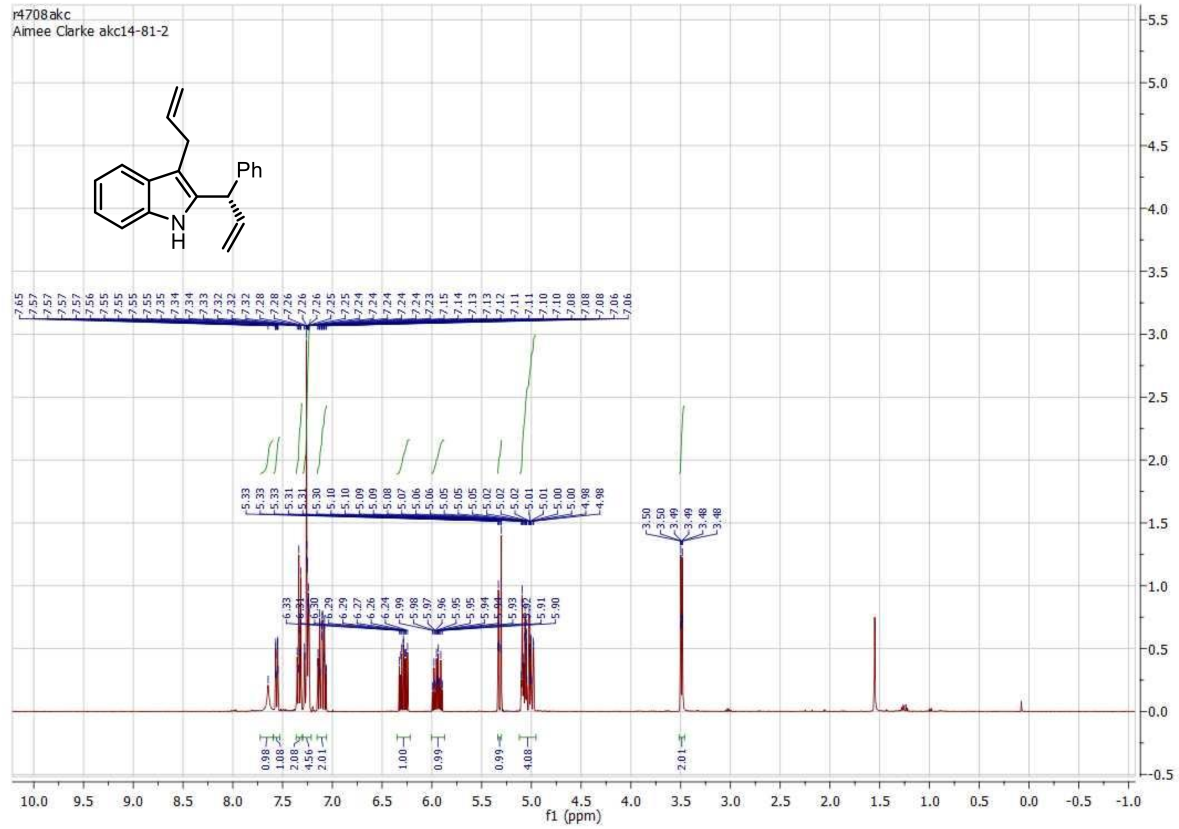
r4708akc
Aimee Clarke akc14-81-2
Ph
N
H
f1 (ppm)

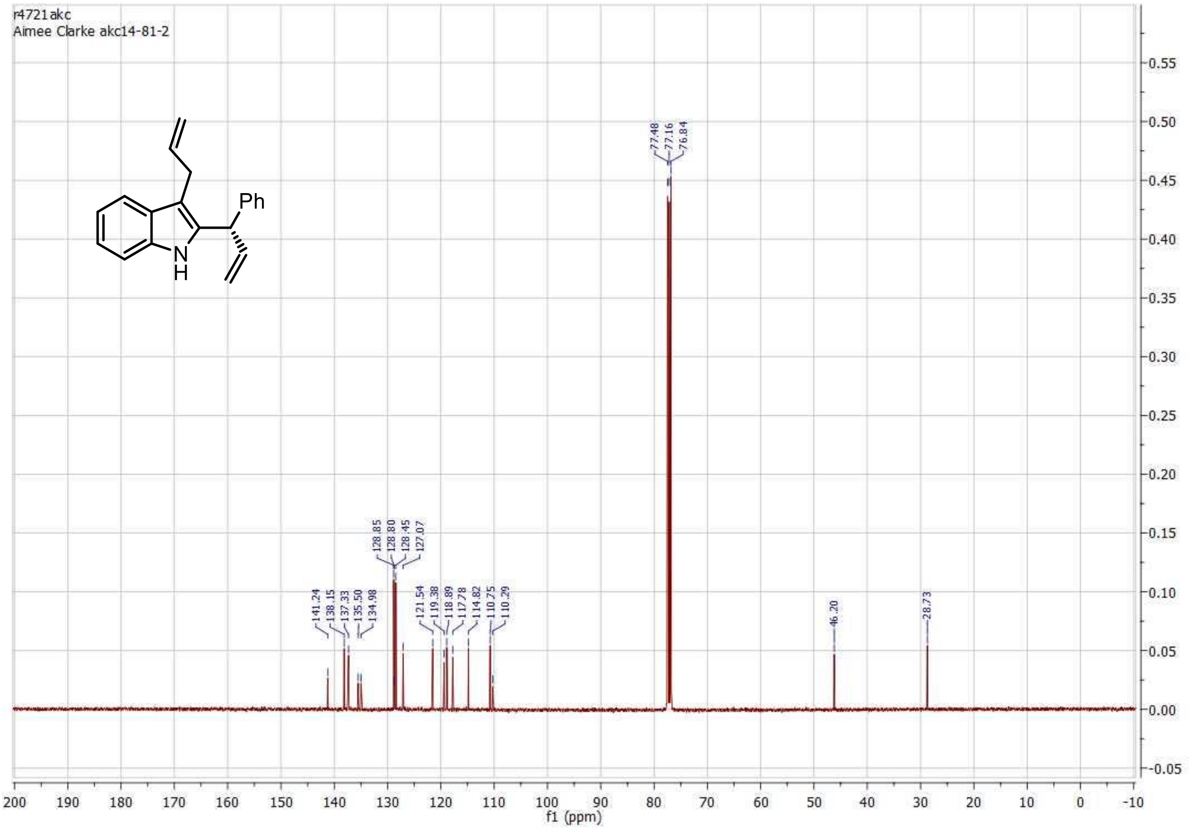
r4721akc
Aimee Clarke akc14-81-2
Ph
N
H
f1 (ppm)

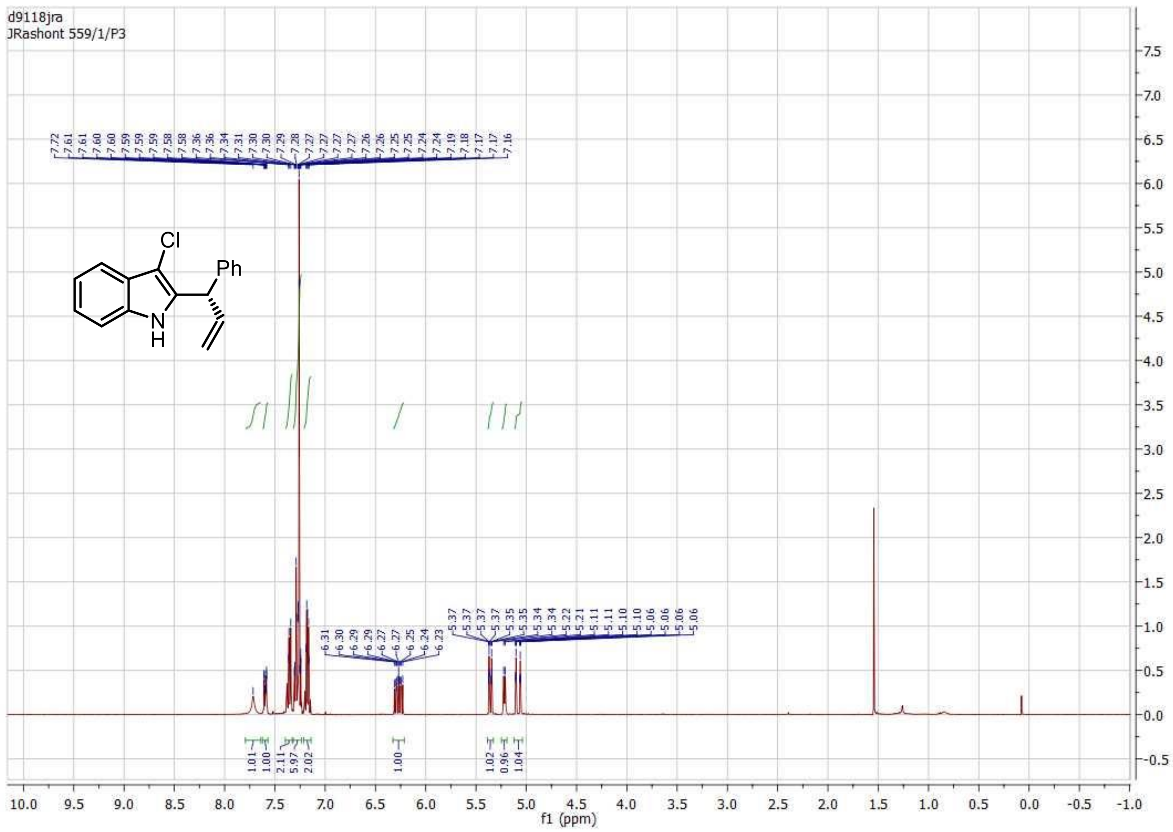
d9118jra
JRashont 559/1/P3
Cl
Ph
N
H
7.72
7.61
7.60
7.59
7.59
7.58
7.58
7.36
7.36
7.34
7.31
7.30
7.30
7.29
7.28
7.27
7.27
7.27
7.27
7.26
7.26
7.25
7.25
7.24
7.24
7.19
7.18
7.17
7.17
7.16
6.31
6.30
6.29
6.29
6.27
6.27
6.25
6.24
6.23
5.37
5.37
5.37
5.37
5.35
5.35
5.34
5.34
5.22
5.21
5.11
5.11
5.10
5.06
5.06
5.06
5.06
1.01
1.00
2.11
5.97
2.02
1.00
1.02
0.96
1.04
f1 (ppm)

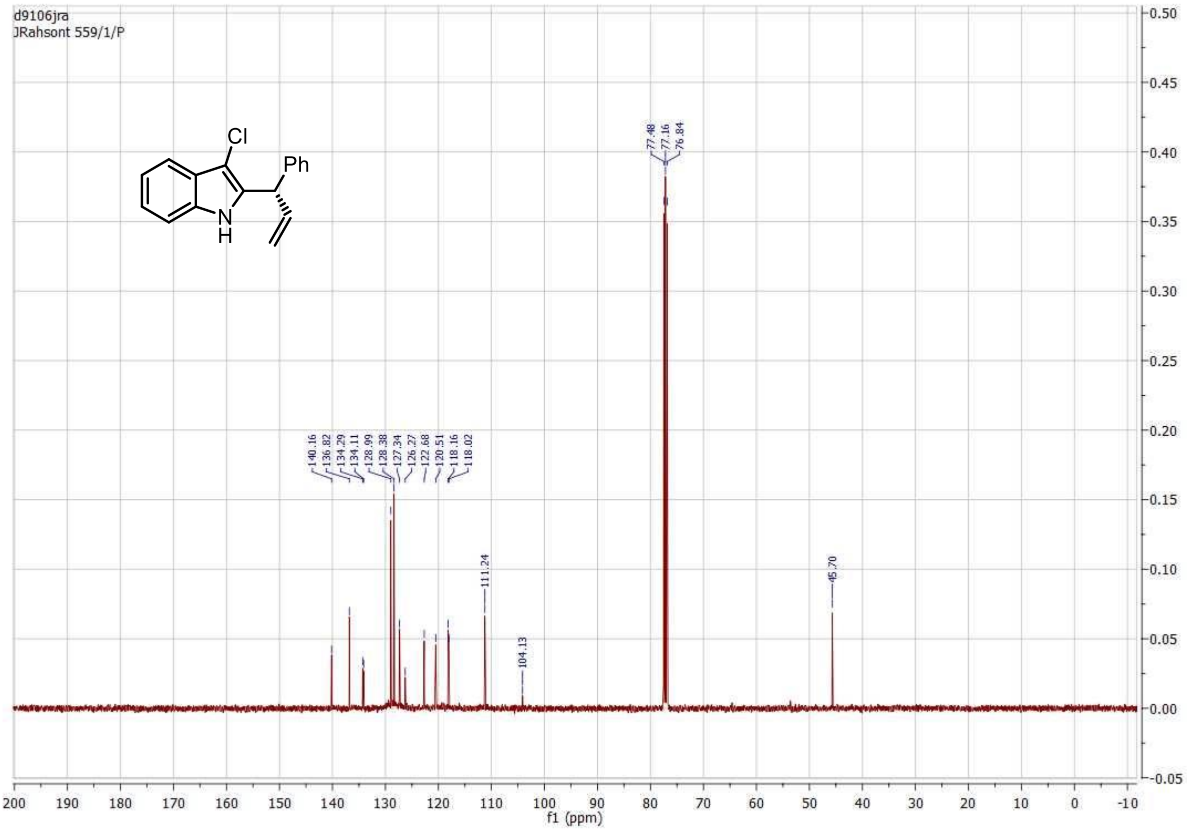
d9106jra
JRahsont 559/1/P
Cl
Ph
N
H
77.48
77.16
76.84
140.16
136.82
134.29
134.11
128.99
128.38
127.34
126.27
122.68
120.51
118.16
118.02
111.24
104.13
45.70
f1 (ppm)

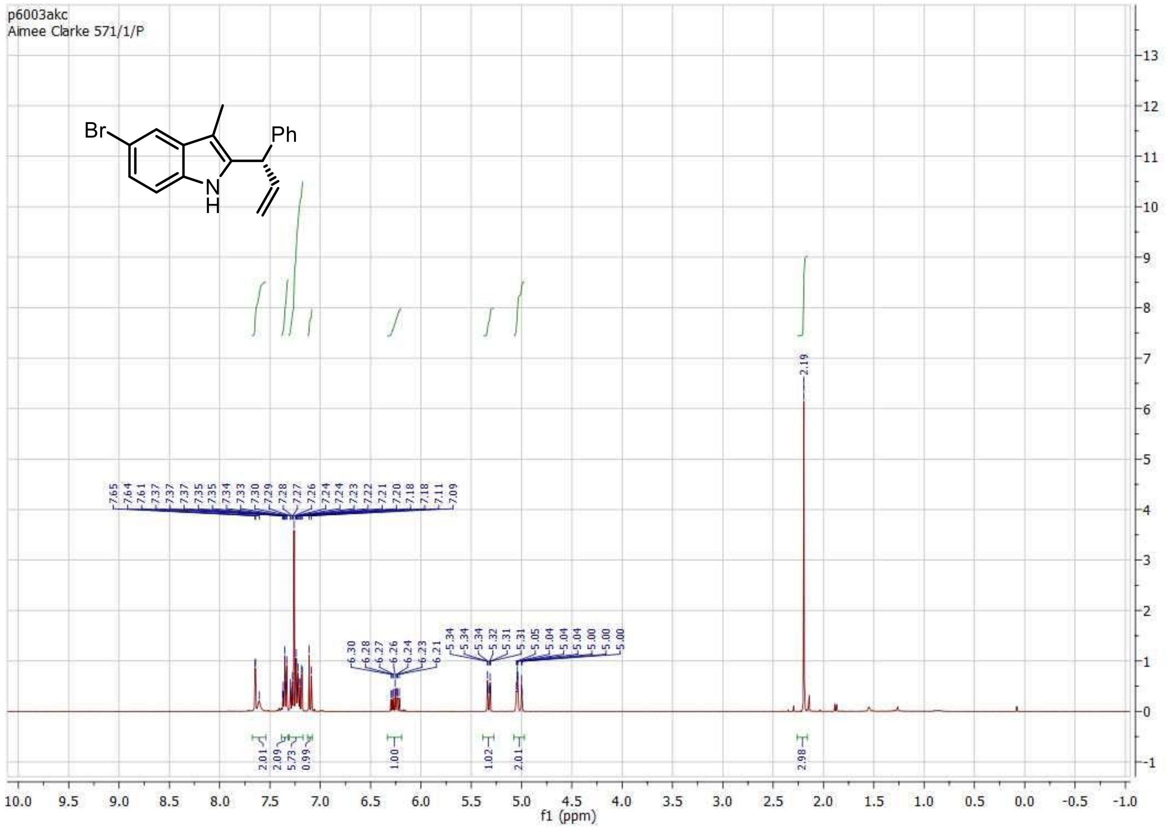
p6003akc
Aimee Clarke 571/1/P
Br
Ph
N
H
f1 (ppm)

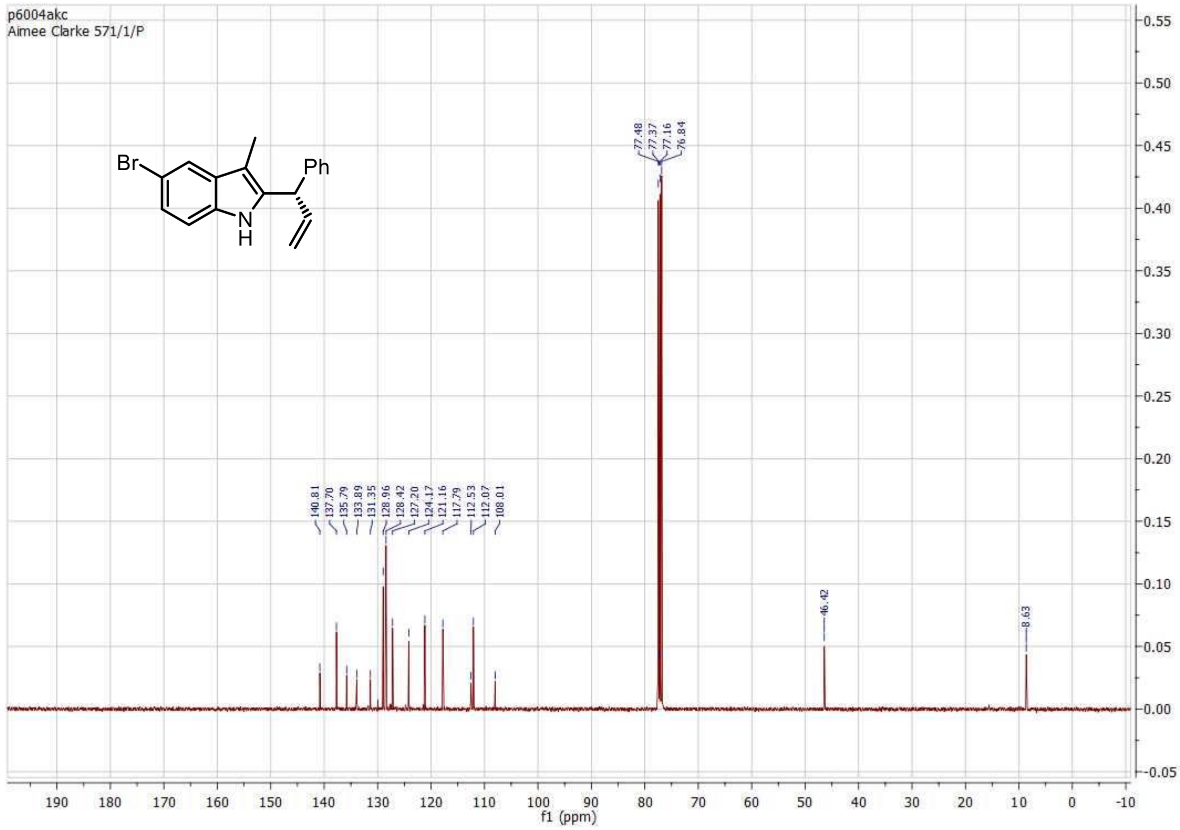
p6004akc
Aimee Clarke 571/1/P
Br
Ph
N
H
f1 (ppm)

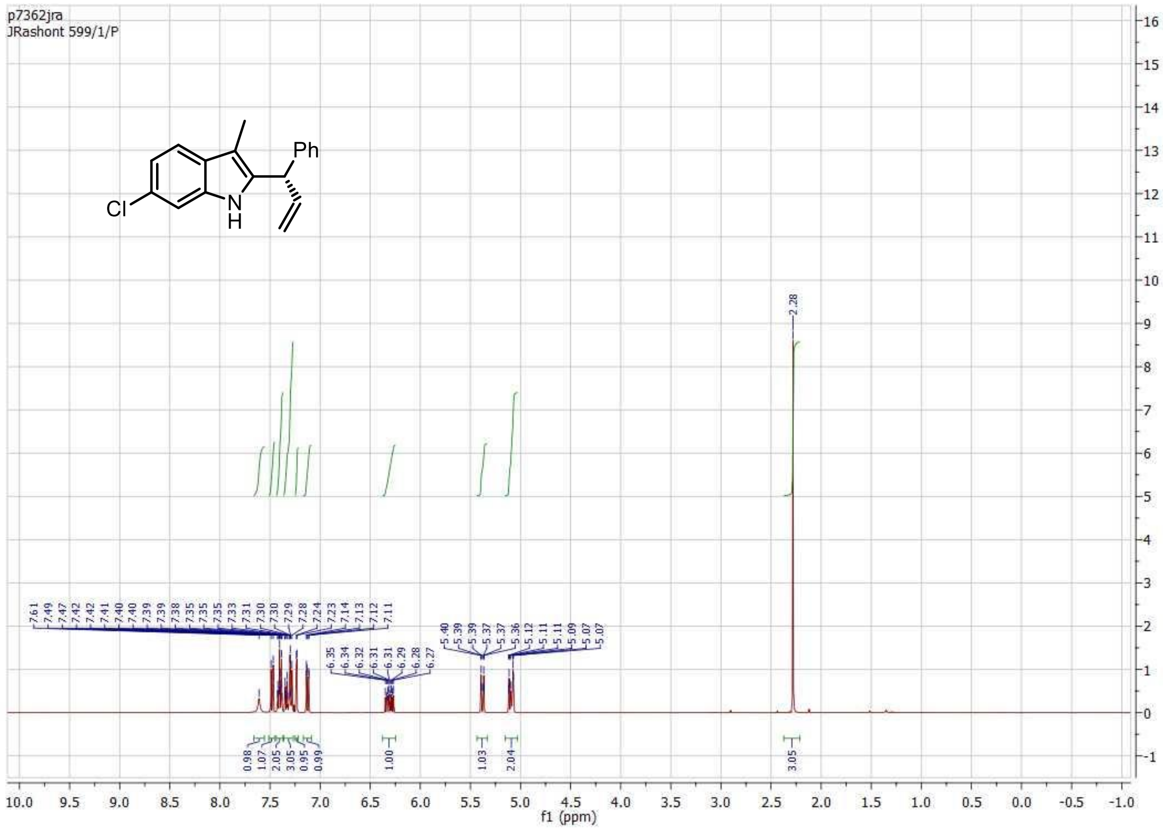
p7362jra
JRashont 599/1/P
Ph
Cl
N
H
2.28
0.98
1.07
2.05
3.05
0.95
0.99
1.00
1.03
2.04
3.05
f1 (ppm)

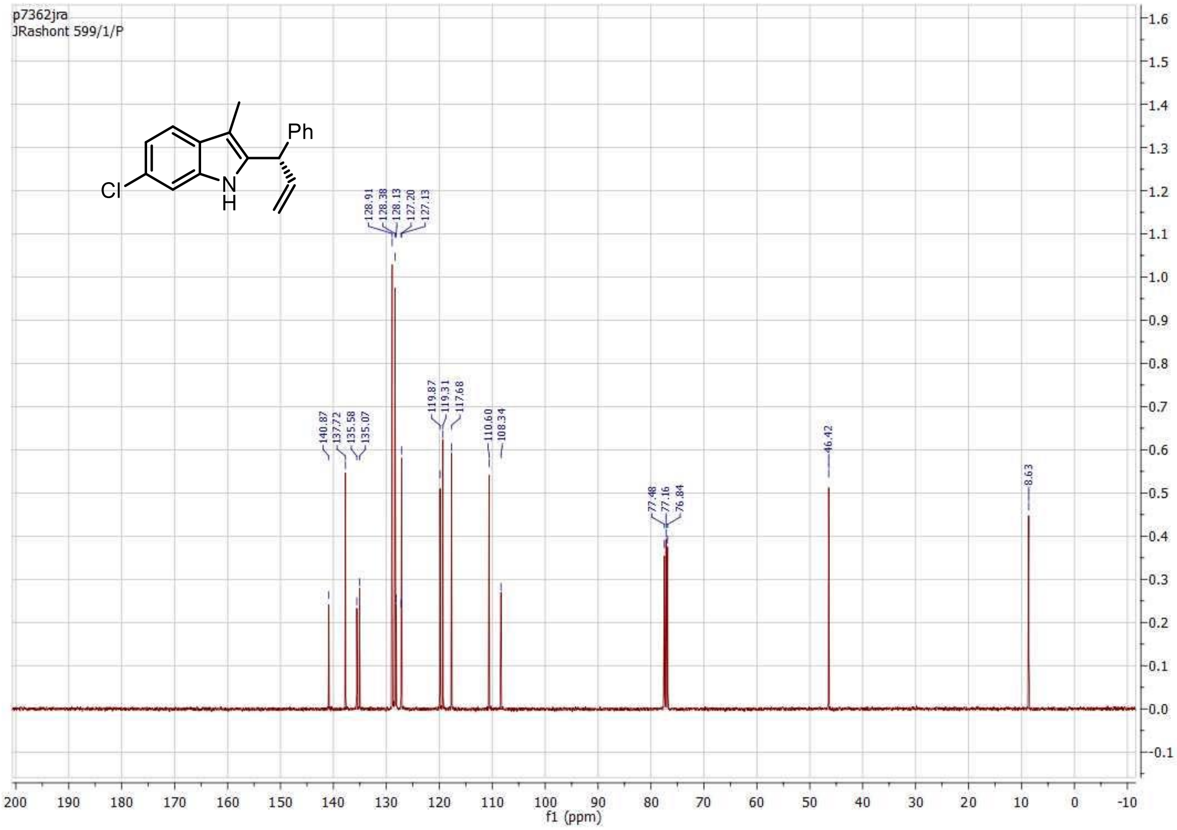
p7362jra
JRashont 599/1/P
Ph
Cl
N
H
128.91
128.38
128.13
127.20
127.13
140.87
137.72
135.58
135.07
119.87
119.31
117.68
110.60
108.34
77.48
77.16
76.84
46.42
8.63
f1 (ppm)

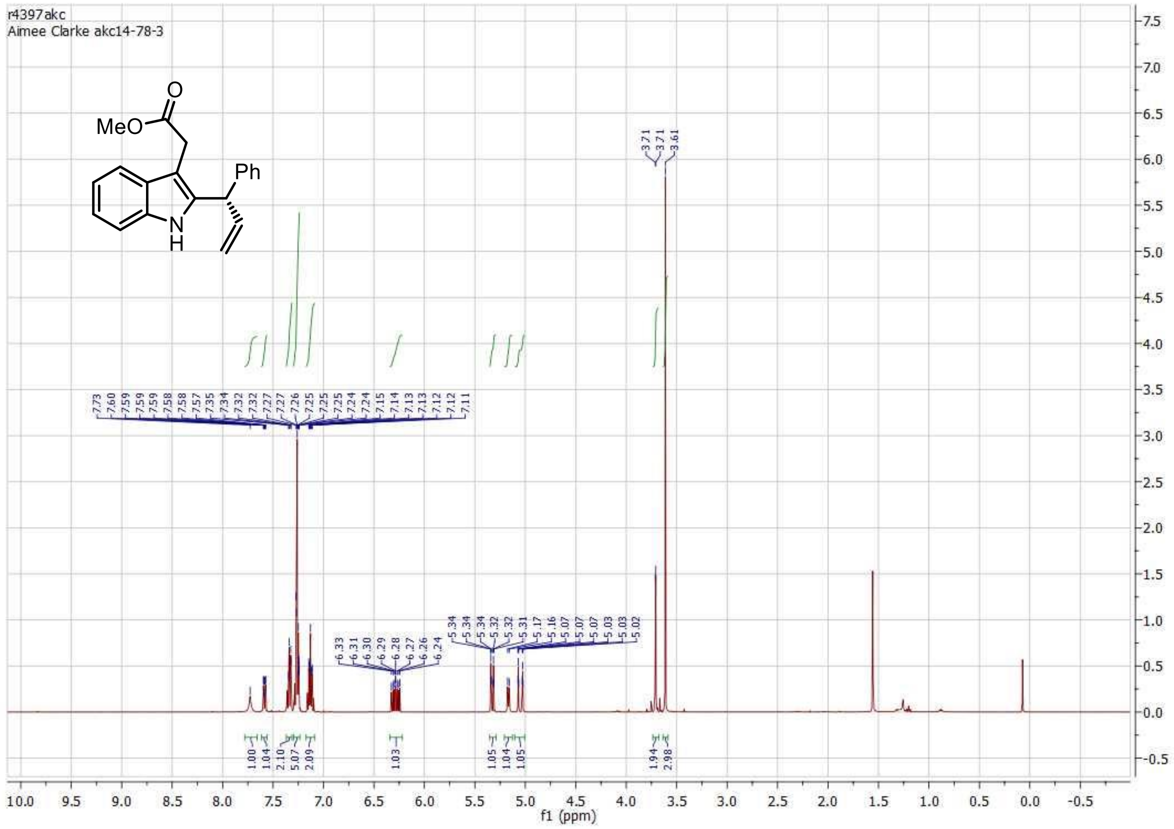
r4397akc
Aimee Clarke akc14-78-3
MeO
O
Ph
N
H
7.73
7.60
7.59
7.59
7.59
7.58
7.58
7.57
7.35
7.34
7.32
7.32
7.27
7.27
7.26
7.25
7.25
7.25
7.24
7.24
7.15
7.14
7.13
7.13
7.12
7.12
7.11
6.33
6.31
6.30
6.29
6.28
6.27
6.26
6.24
5.34
5.34
5.34
5.32
5.32
5.31
5.17
5.16
5.07
5.07
5.07
5.03
5.03
5.02
3.71
3.71
3.61
1.00
1.04
2.10
5.07
2.09
1.03
1.05
1.04
1.05
1.94
2.98
f1 (ppm)

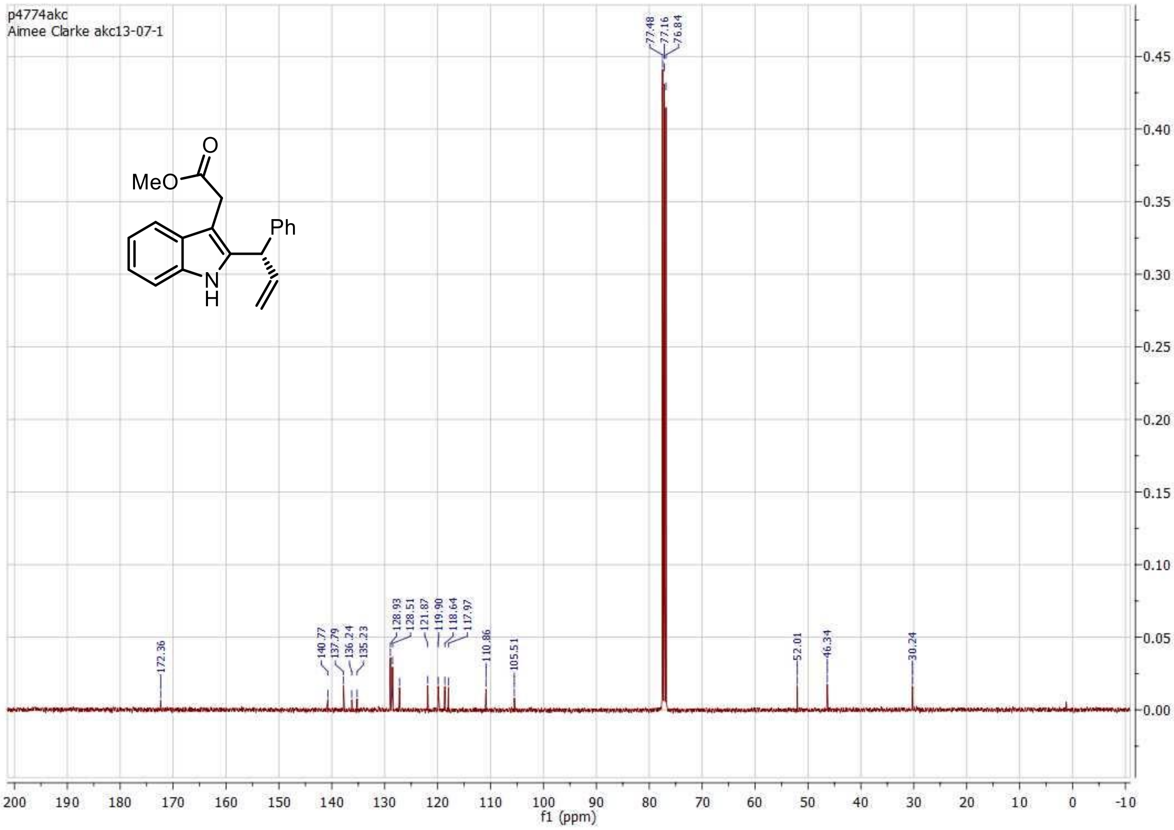
p4774akc
Aimee Clarke akc13-07-1
MeO
O
Ph
N
H
77.48
77.16
76.84
172.36
140.77
137.79
136.24
135.23
128.93
128.51
121.87
119.90
118.64
117.97
110.86
105.51
52.01
46.34
30.24
f1 (ppm)

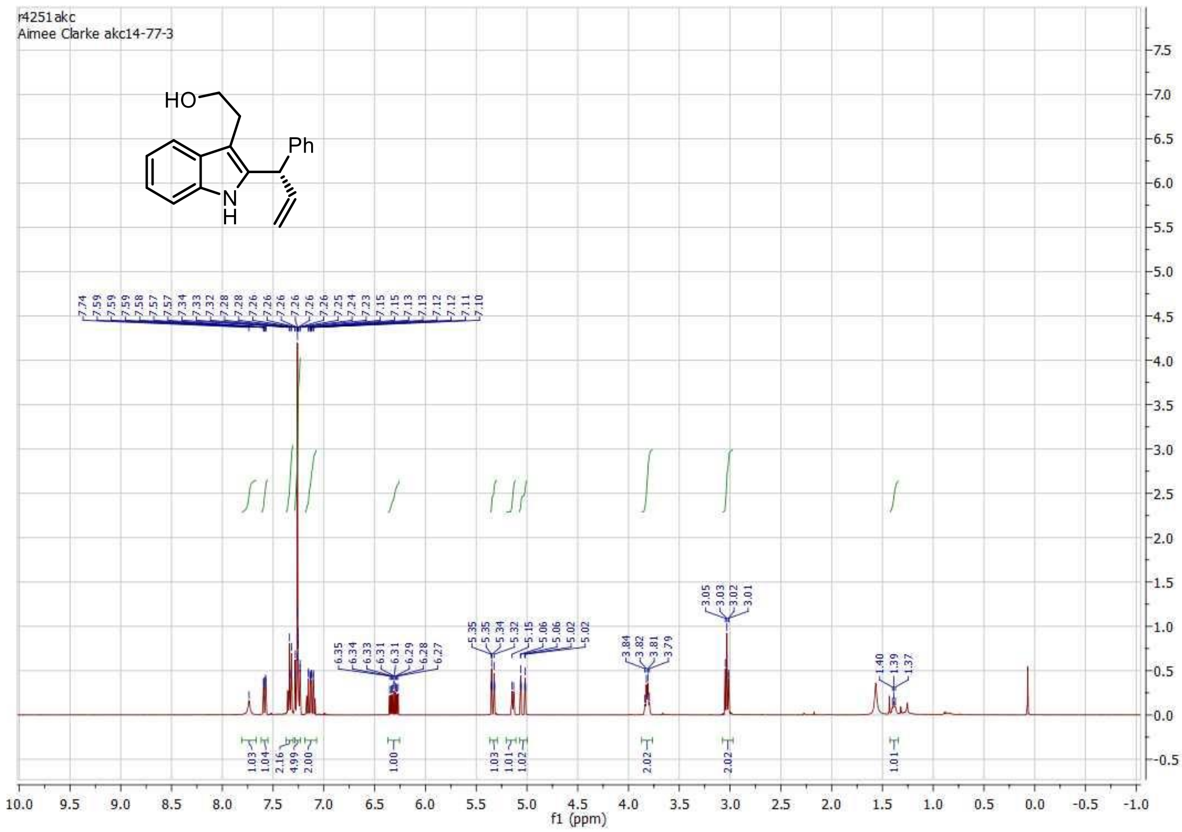
r4251akc
Aimee Clarke akc14-77-3
HO
Ph
N
H
f1 (ppm)

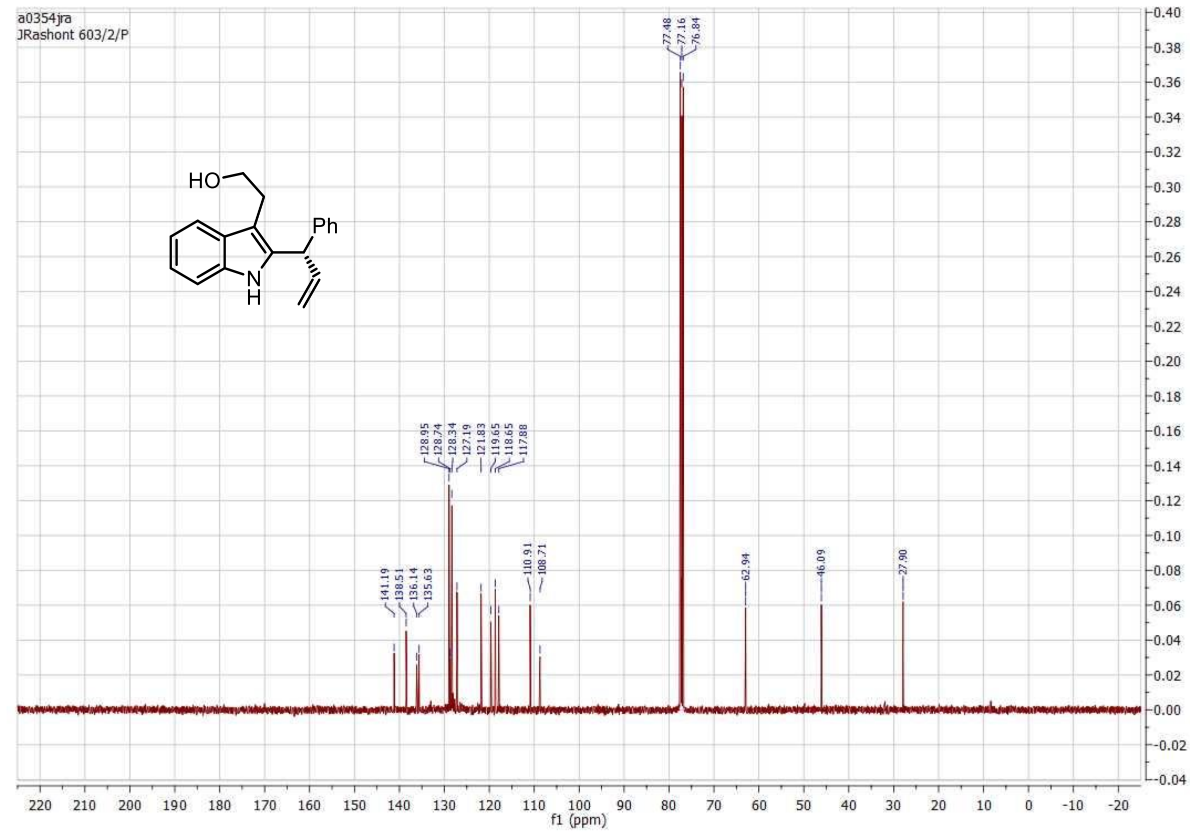
a0354jra
JRashont 603/2/P
HO
Ph
N
H
f1 (ppm)

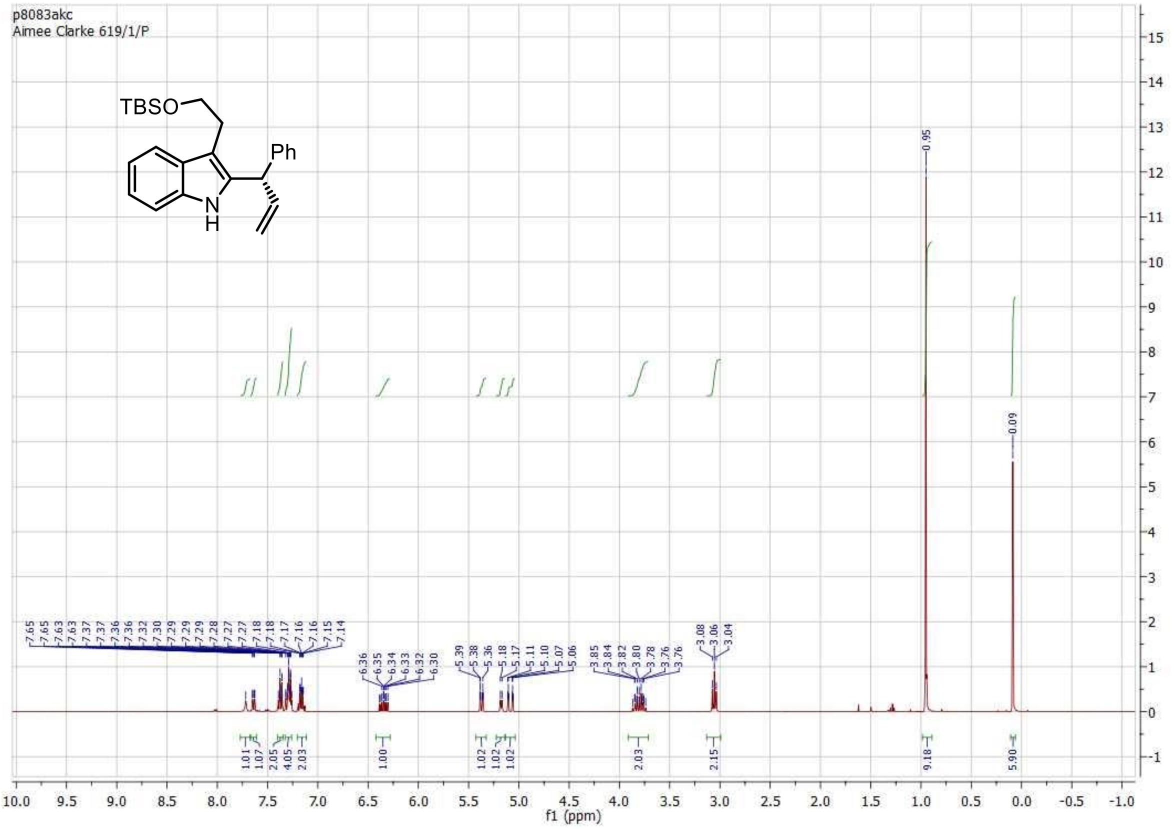
p8083akc
Aimee Clarke 619/1/P
TBSO
Ph
N
H
f1 (ppm)

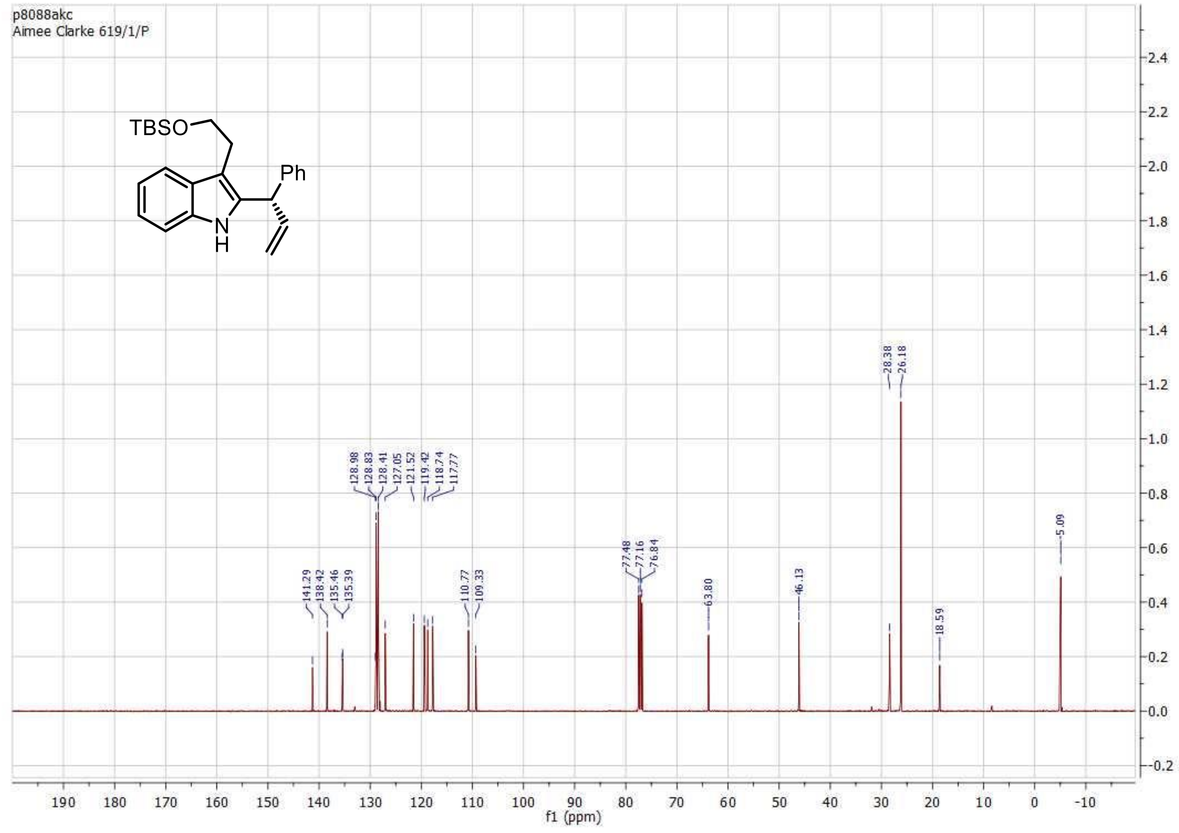
p8088akc
Aimee Clarke 619/1/P
TBSO
Ph
N
H
f1 (ppm)

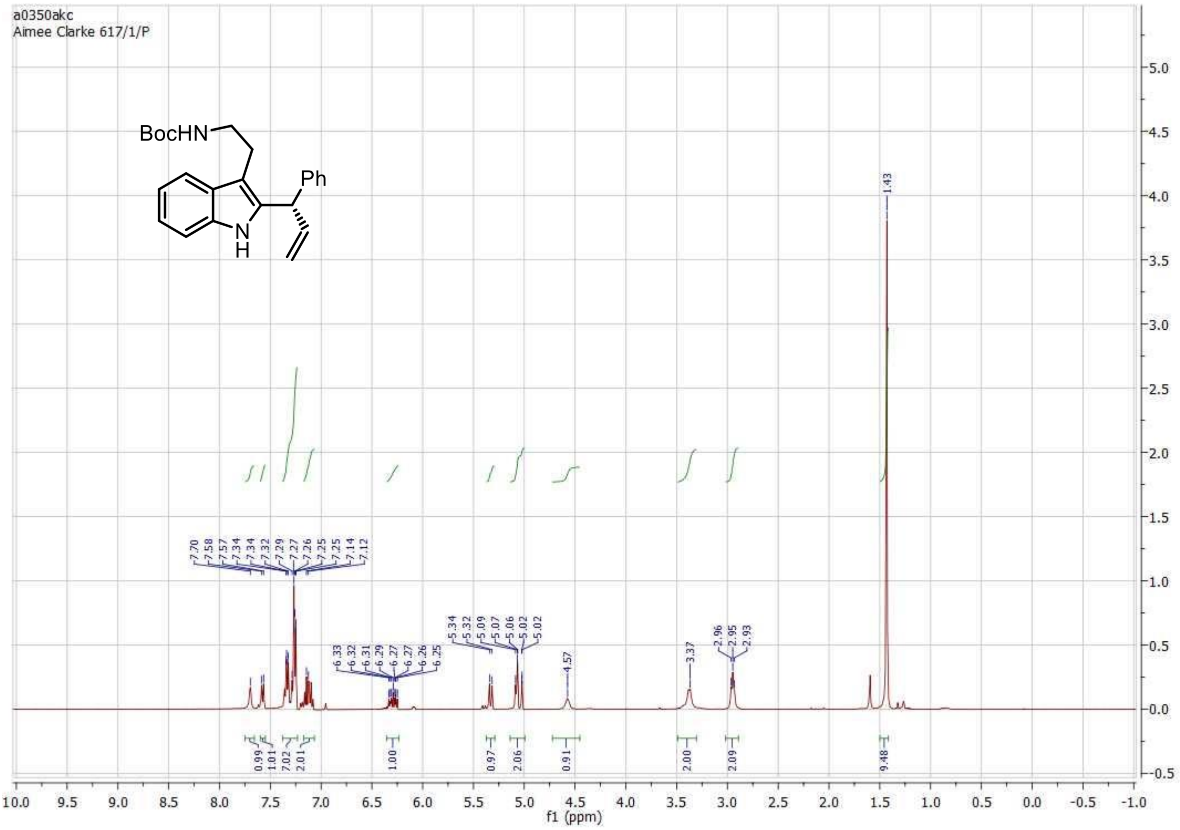
a0350akc
Aimee Clarke 617/1/P
BocHN
Ph
N
H
7.70 7.58 7.57 7.34 7.34 7.32 7.29 7.27 7.27 7.26 7.25 7.25 7.14 7.12
6.33 6.32 6.31 6.29 6.27 6.27 6.26 6.25
5.34 5.32 5.09 5.07 5.06 5.02 5.02
4.57
3.37
2.96 2.95 2.93
1.43
0.99 1.01 7.02 2.01 1.00 0.97 2.06 0.91 2.00 2.09 9.48
f1 (ppm)

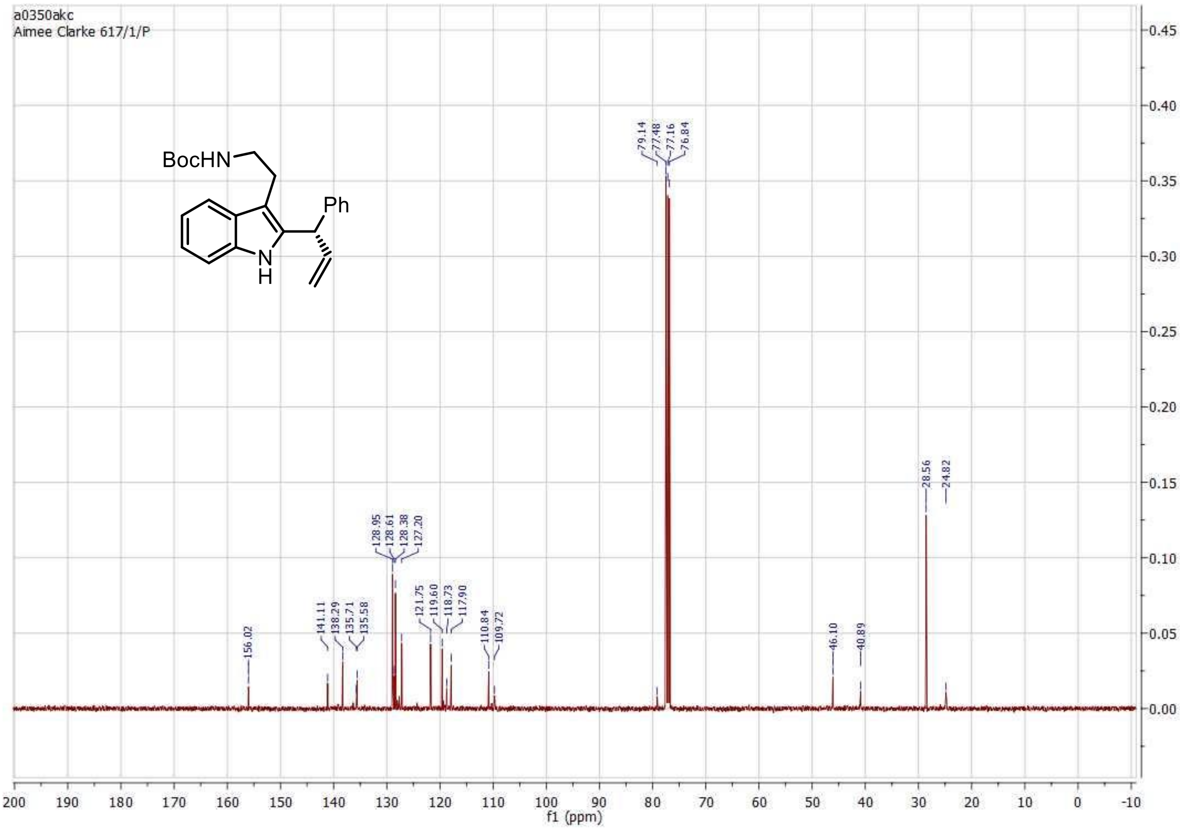
a0350akc
Aimee Clarke 617/1/P
BocHN
Ph
N
H
156.02
141.11 138.29 135.71 135.58
128.95 128.61 128.38 127.20
121.75 119.60 118.73 117.90
110.84 109.72
79.14 77.48 77.16 76.84
46.10
40.89
28.56
24.82
f1 (ppm)

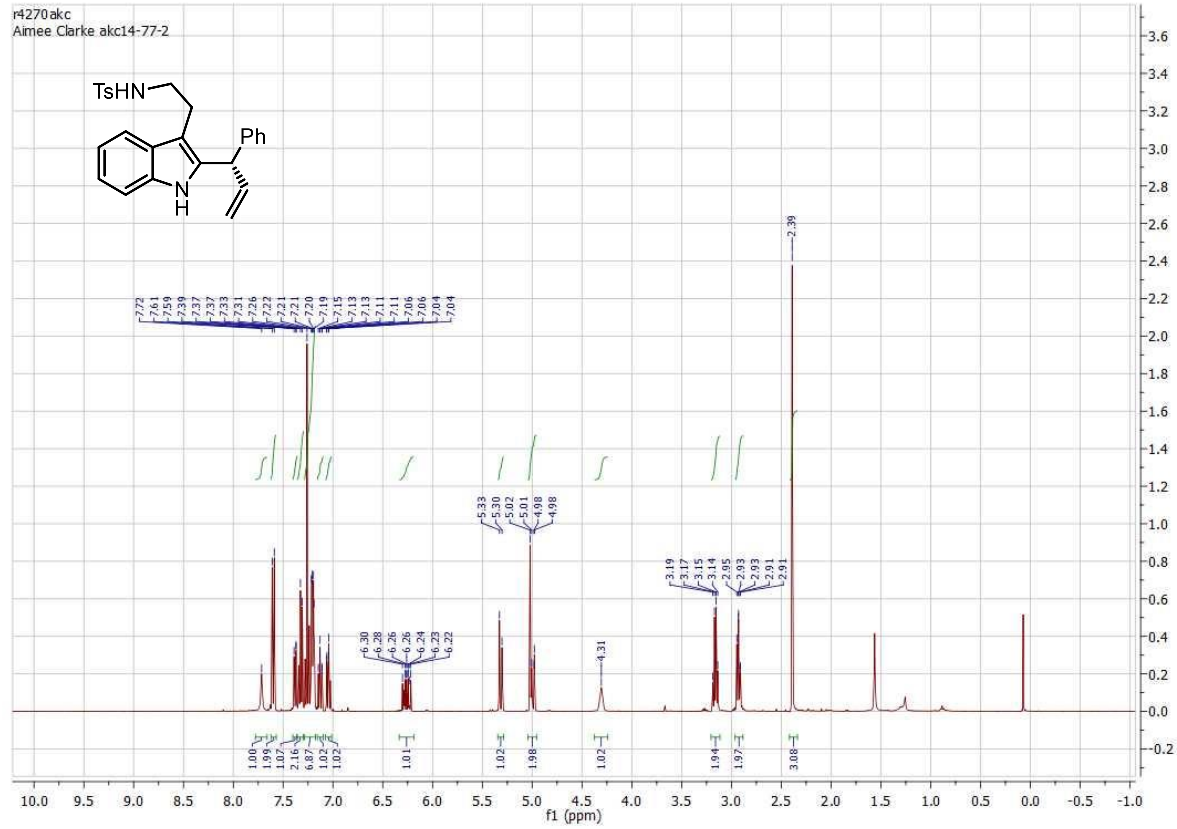
r4270akc
Aimee Clarke akc14-77-2
TsHN
Ph
N
H
f1 (ppm)

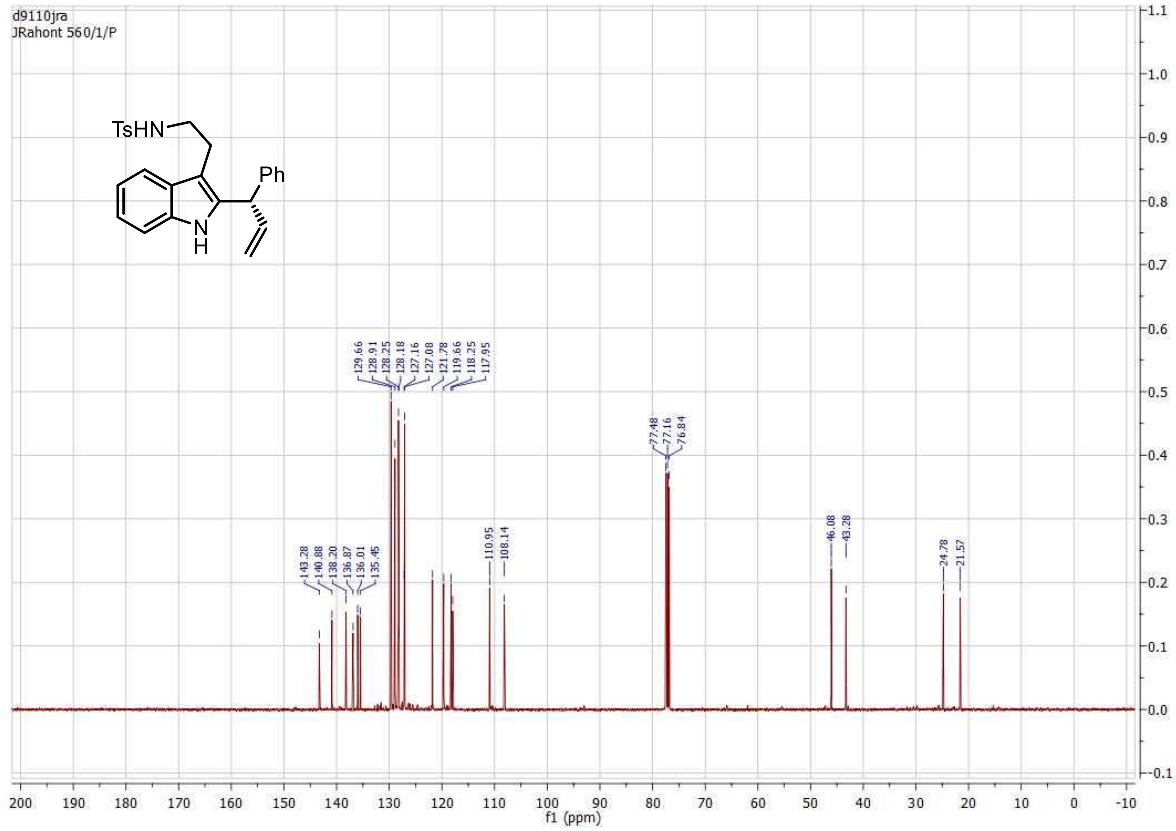
d9110jra
JRahont 560/1/P
TsHN
Ph
N
H
f1 (ppm)

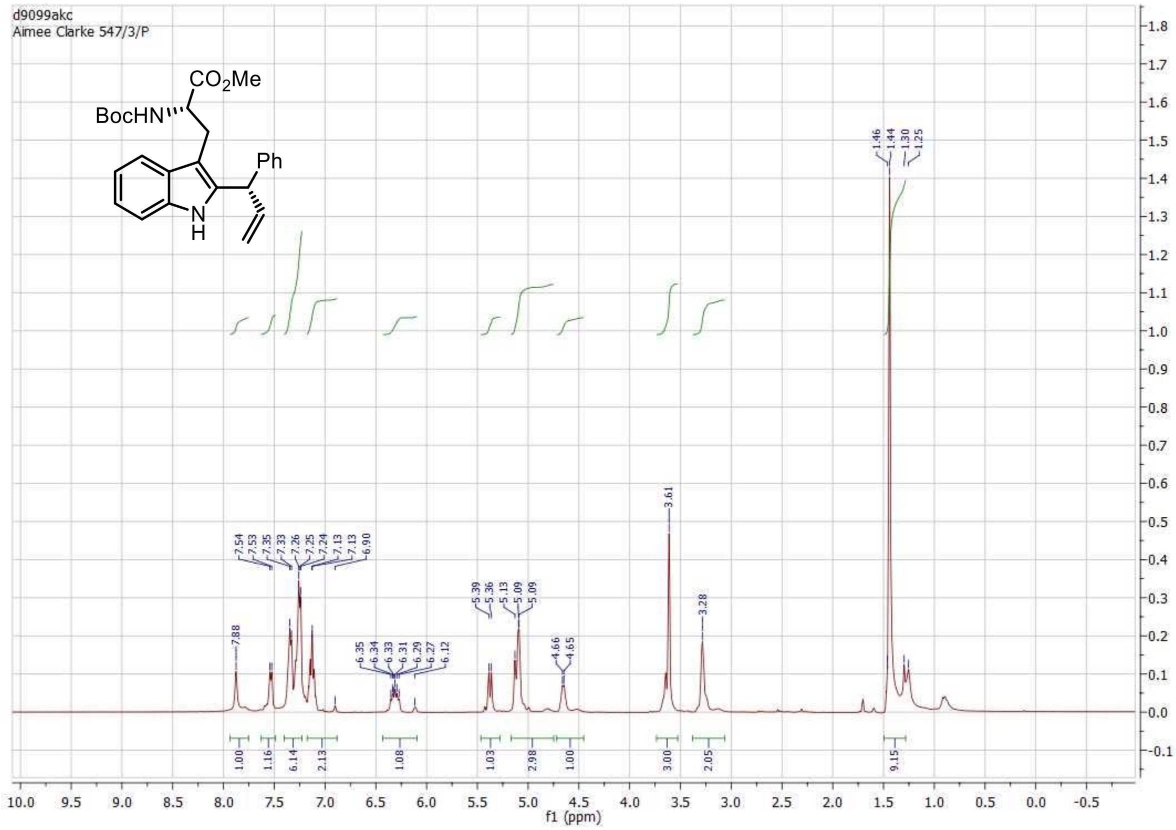
d9099akc
Aimee Clarke 547/3/P
CO2Me
BocHN
Ph
N
H
7.88
7.54 7.53 7.35 7.33 7.26 7.25 7.24 7.13 7.13 6.90
6.35 6.34 6.33 6.31 6.29 6.27 6.12
5.39 5.36 5.13 5.09 5.09
4.66 4.65
3.61
3.28
1.46 1.44 1.30 1.25
1.00 1.16 6.14 2.13 1.08 1.03 2.98 1.00 3.00 2.05 9.15
f1 (ppm)

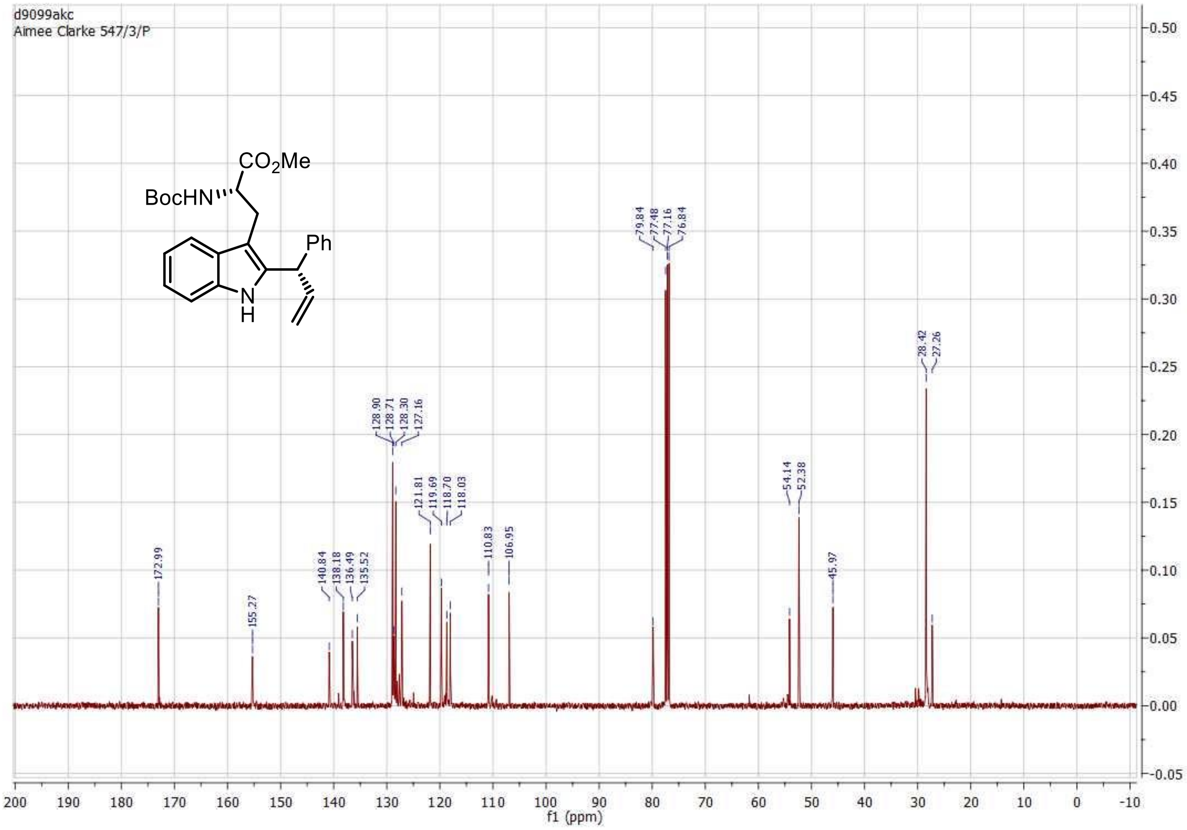
d9099akc
Aimee Clarke 547/3/P
CO2Me
BocHN
Ph
N
H
172.99
155.27
140.84 138.18 136.49 135.52
128.90 128.71 128.30 127.16
121.81 119.69 118.70 118.03
110.83 106.95
79.84 77.48 77.16 76.84
54.14 52.38
45.97
28.42 27.26
f1 (ppm)

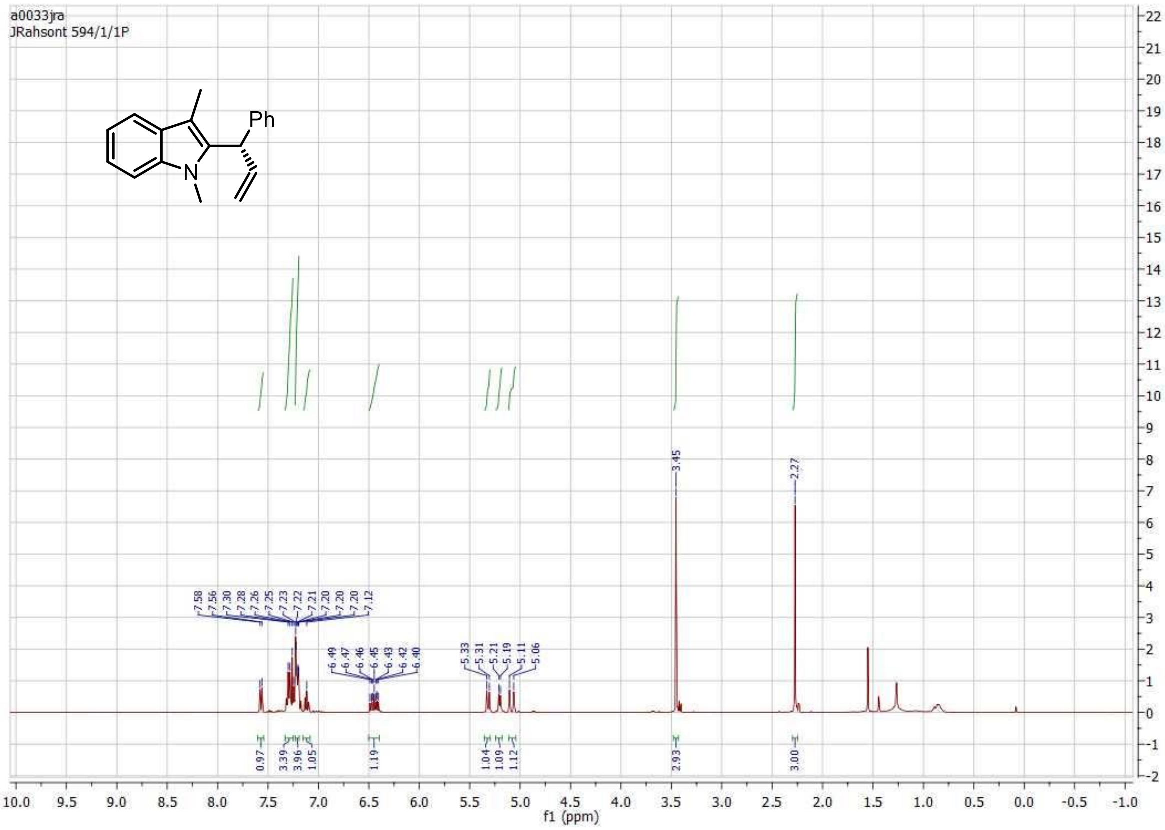
a0033jra
JRahsont 594/1/1P
Ph
N
f1 (ppm)

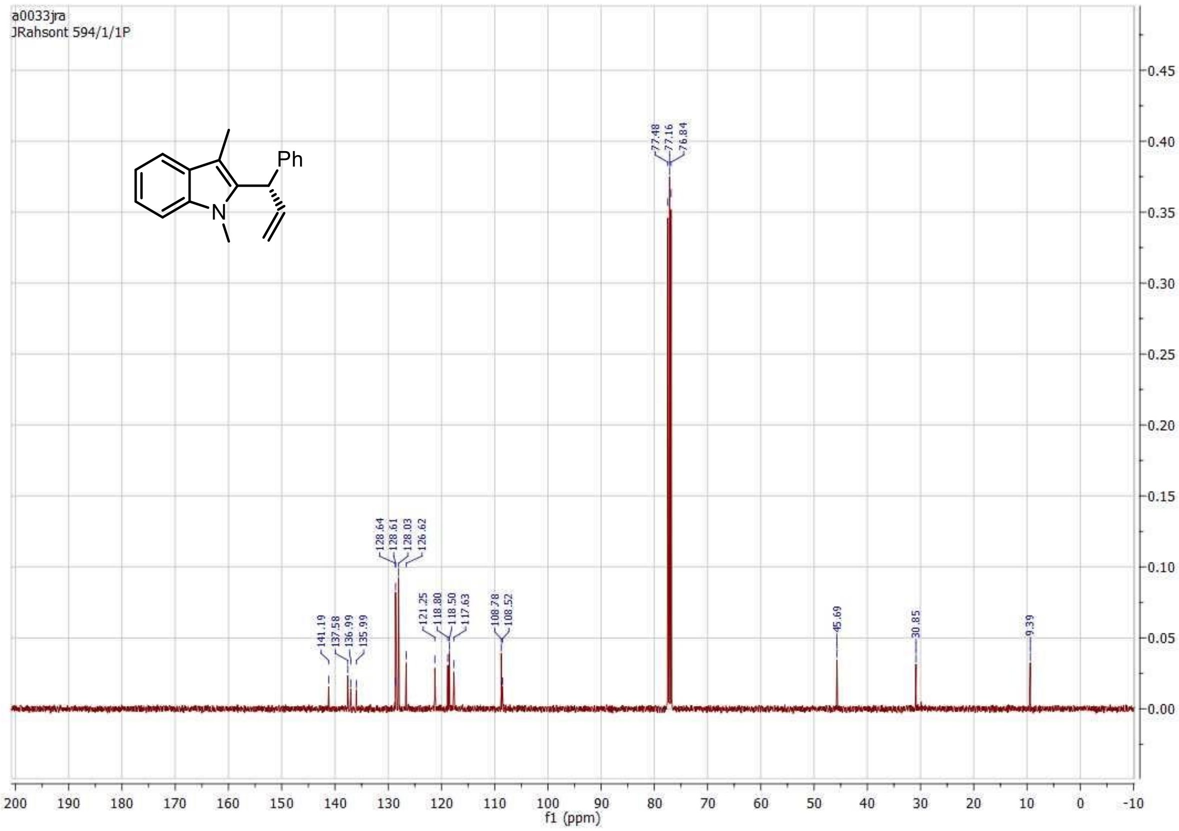
a0033jra
JRahsont 594/1/1P
Ph
N
f1 (ppm)

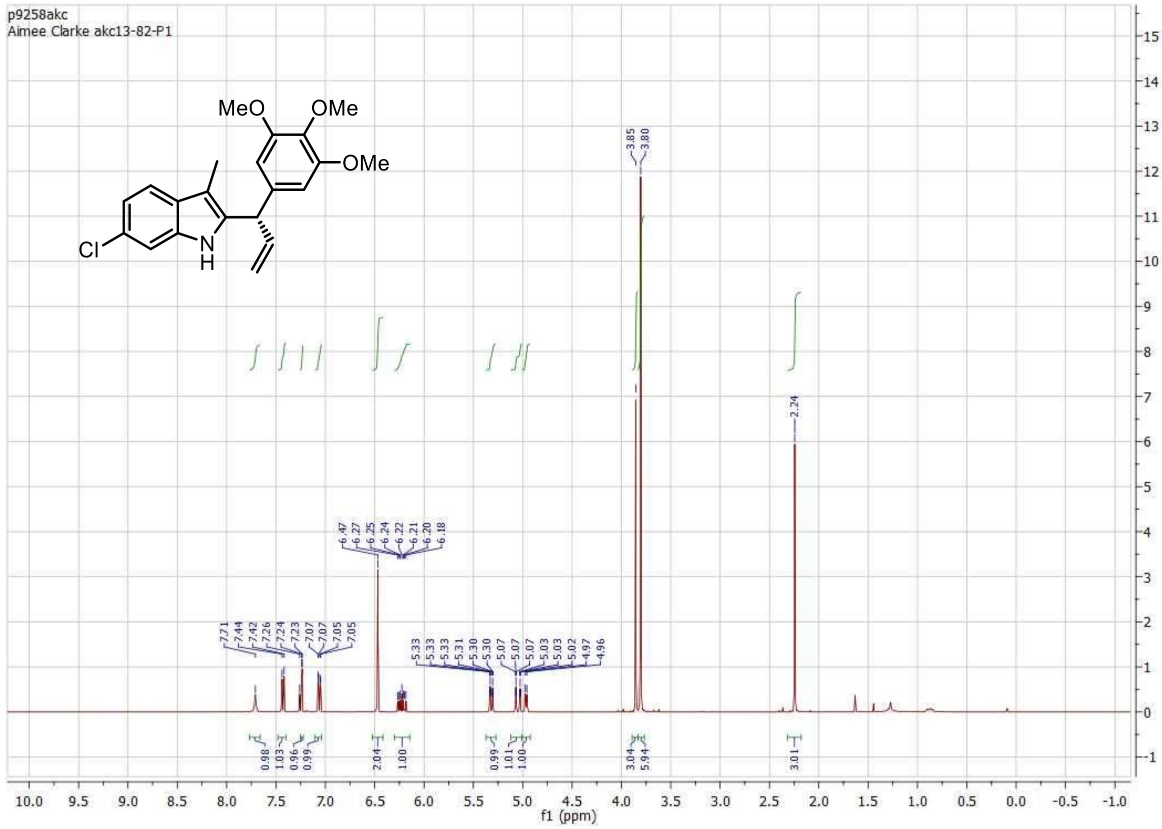
p9258akc
Aimee Clarke akc13-82-P1
MeO
OMe
OMe
Cl
N
H
7.71
7.44
7.42
7.26
7.24
7.23
7.07
7.07
7.05
7.05
6.47
6.27
6.25
6.24
6.22
6.21
6.20
6.18
5.33
5.33
5.33
5.31
5.30
5.30
5.07
5.07
5.07
5.03
5.03
5.02
4.97
4.96
3.85
3.80
2.24
0.98
1.03
0.96
0.99
2.04
1.00
0.99
1.01
1.00
3.04
5.94
3.01
f1 (ppm)

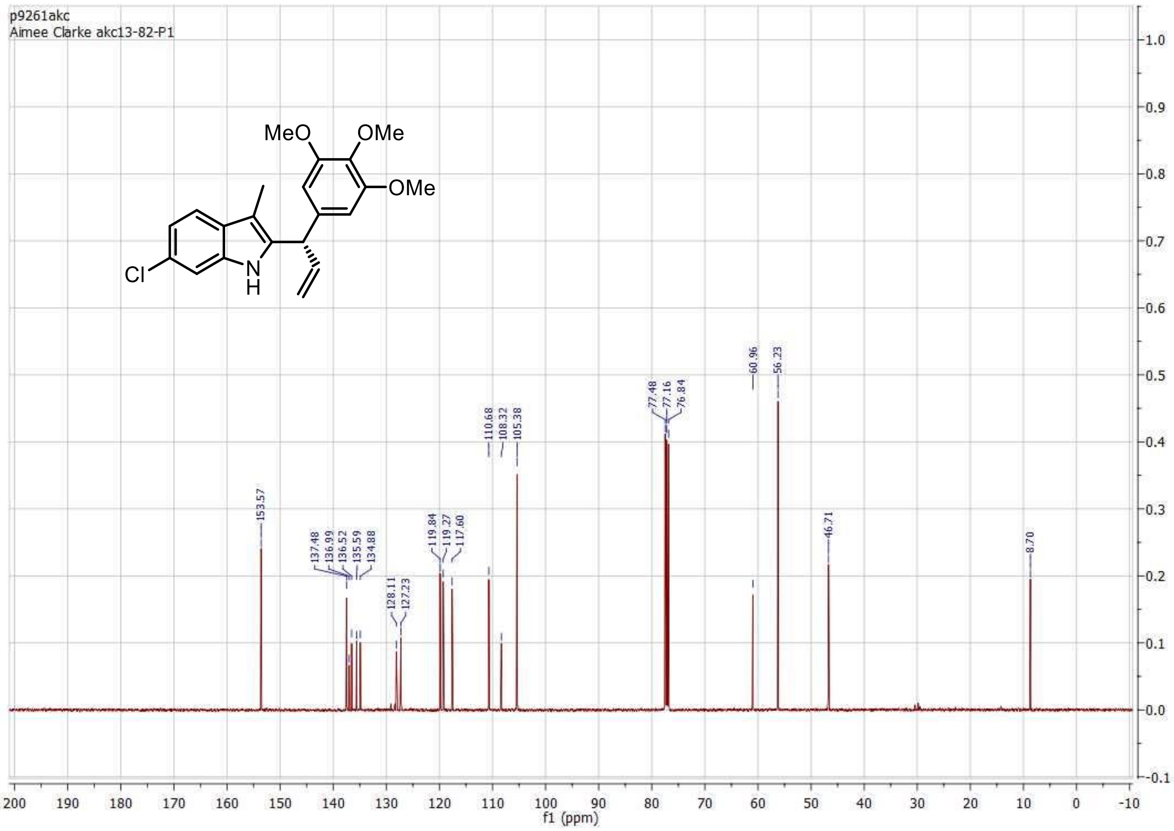
p9261akc
Aimee Clarke akc13-82-P1
MeO
OMe
OMe
Cl
N
H
153.57
137.48
136.99
136.52
135.59
134.88
128.11
127.23
119.84
119.27
117.60
110.68
108.32
105.38
77.48
77.16
76.84
60.96
56.23
46.71
8.70
f1 (ppm)

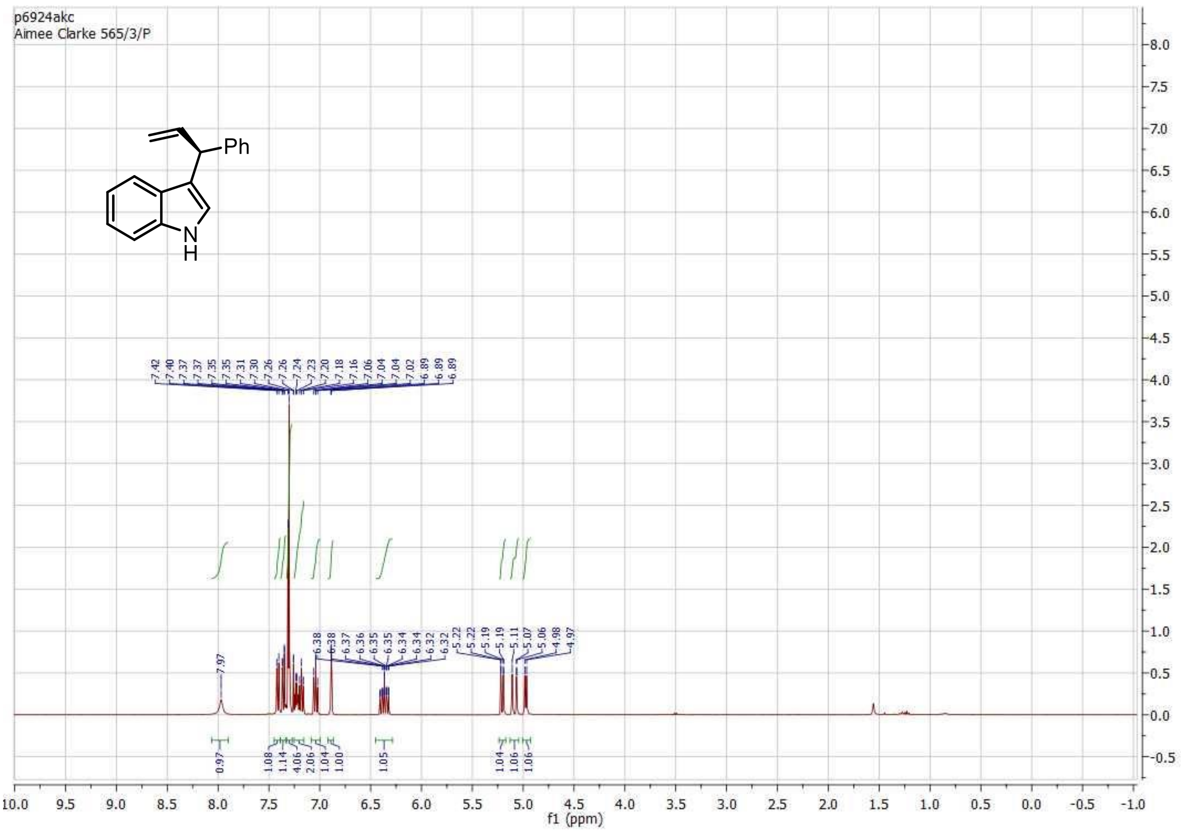
p6924akc
Aimee Clarke 565/3/P
Ph
N
H
f1 (ppm)

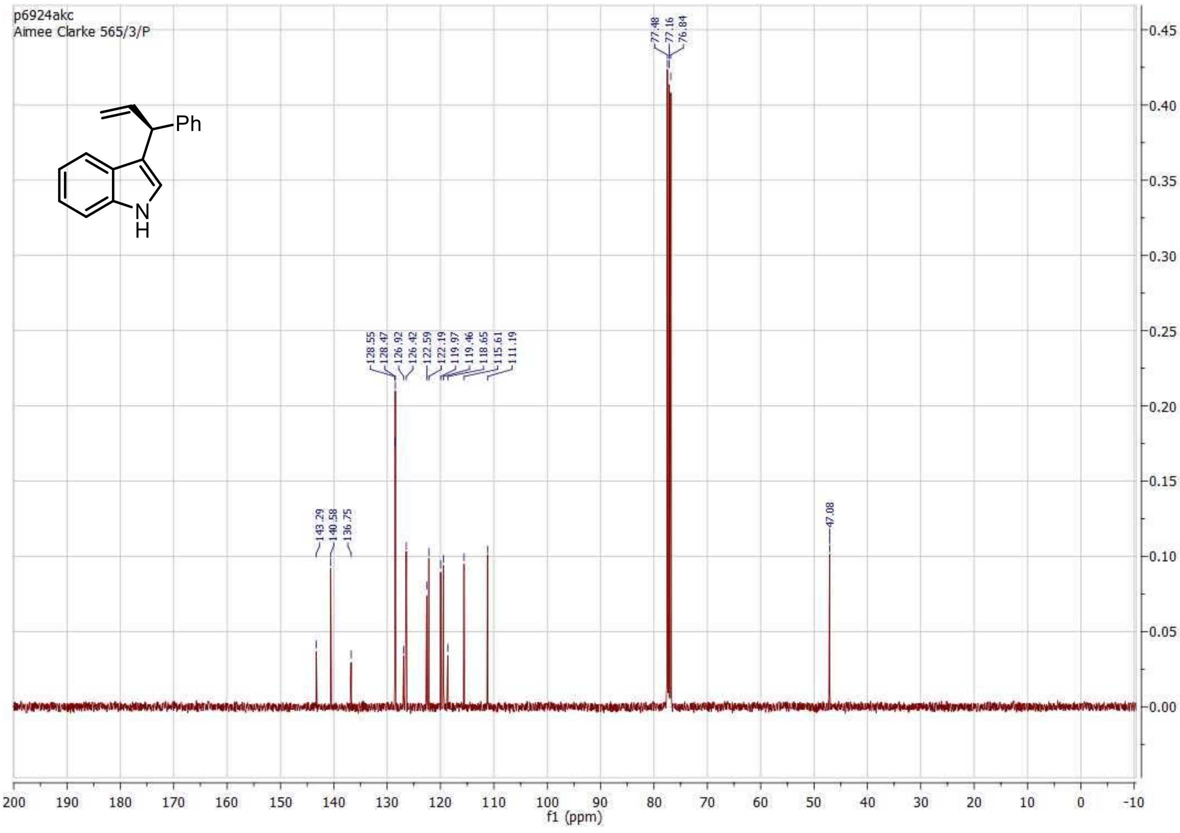
p6924akc
Aimee Clarke 565/3/P
Ph
N
H
f1 (ppm)

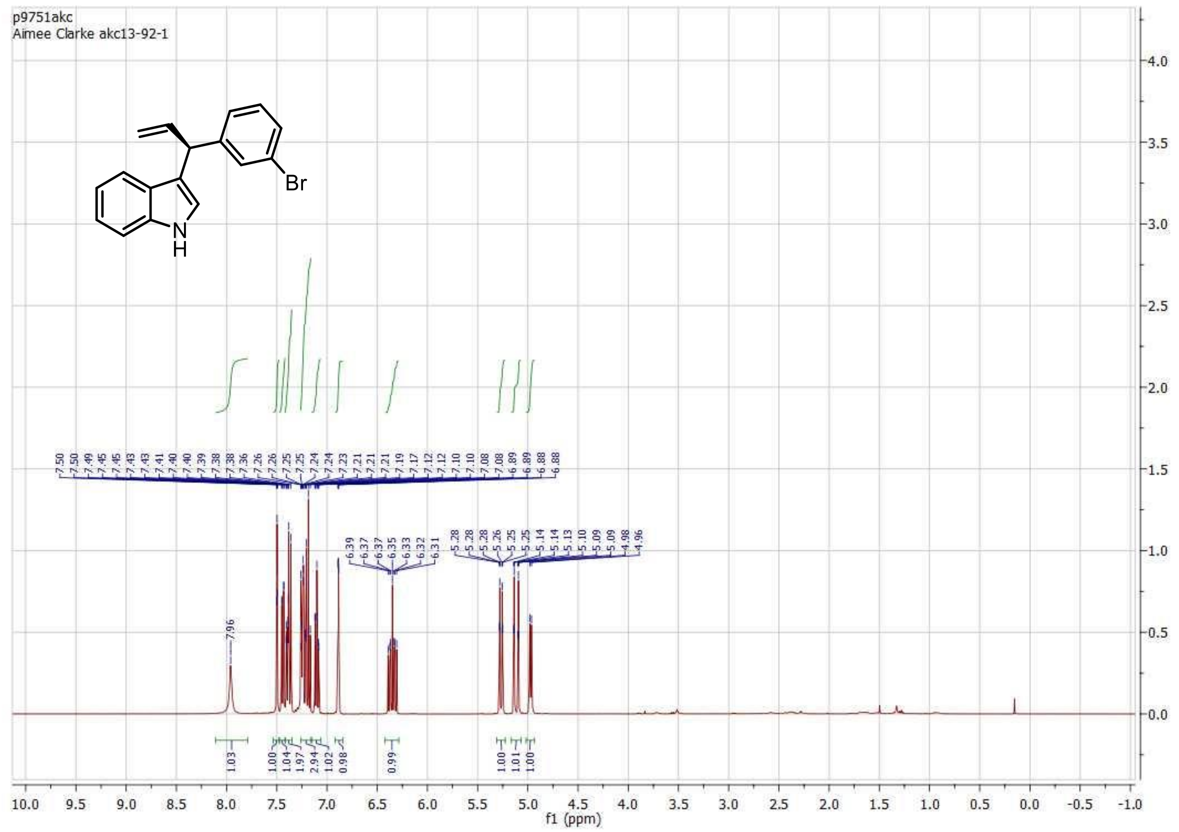
p9751akc
Aimee Clarke akc13-92-1
Br
N
H
7.96
1.03
1.00
1.04
1.97
2.94
1.02
0.98
0.99
1.00
1.01
1.00
f1 (ppm)

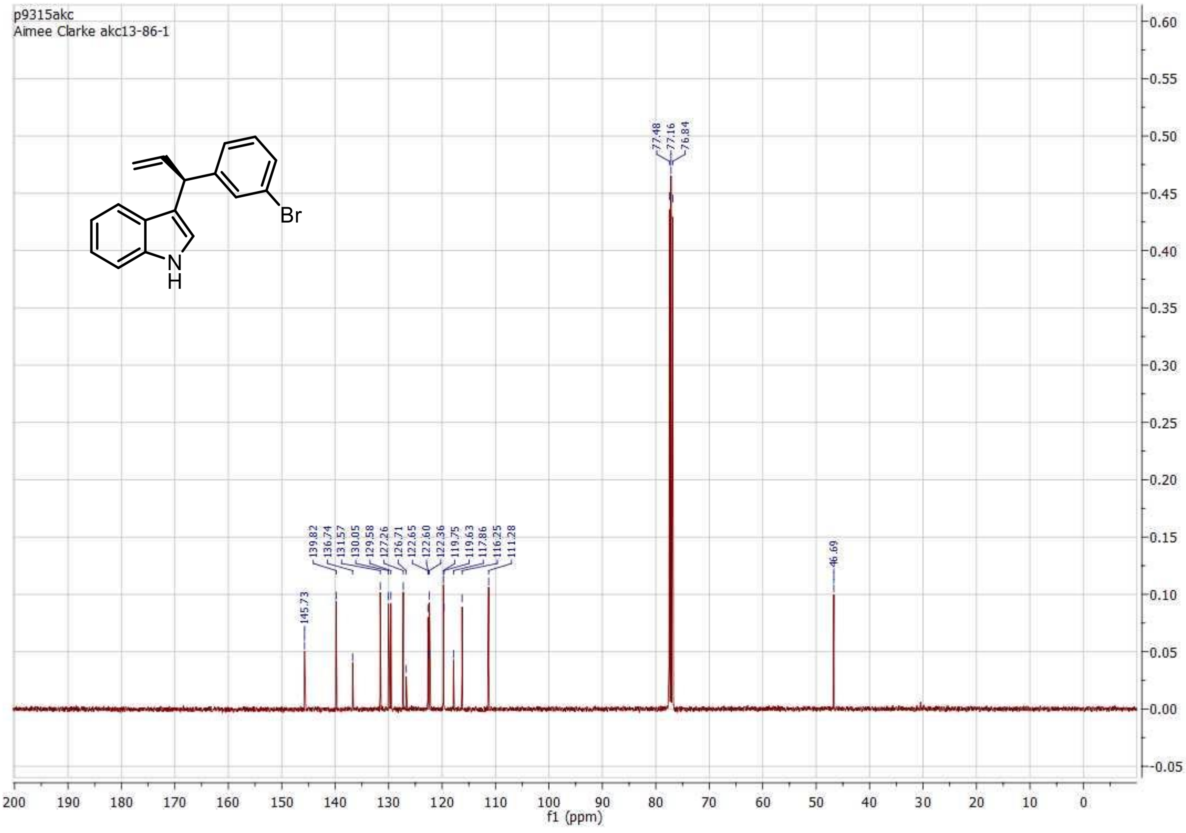
p9315akc
Aimee Clarke akc13-86-1
Br
N
H
77.48
77.16
76.84
145.73
139.82
136.74
131.57
130.05
129.58
127.26
126.71
122.65
122.60
122.36
119.75
119.63
117.86
116.25
111.28
46.69
f1 (ppm)

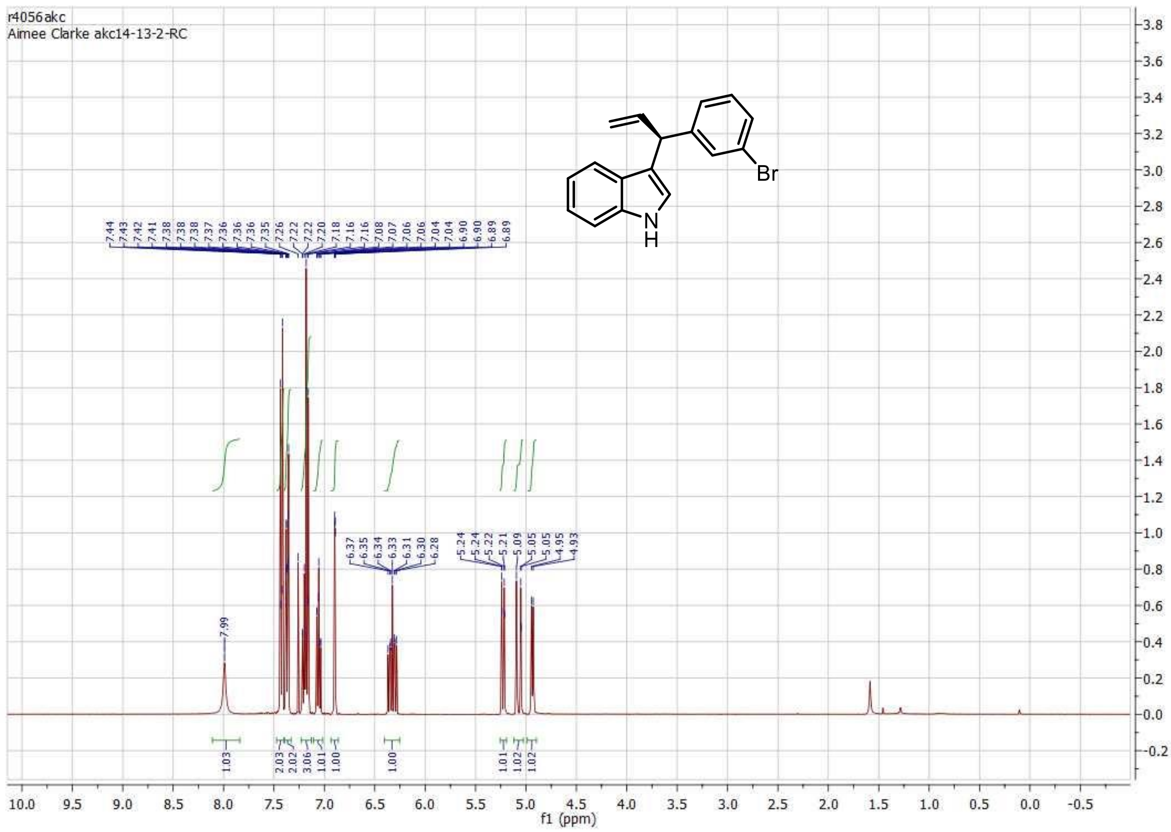
r4056akc
Aimee Clarke akc14-13-2-RC
Br
N
H
f1 (ppm)

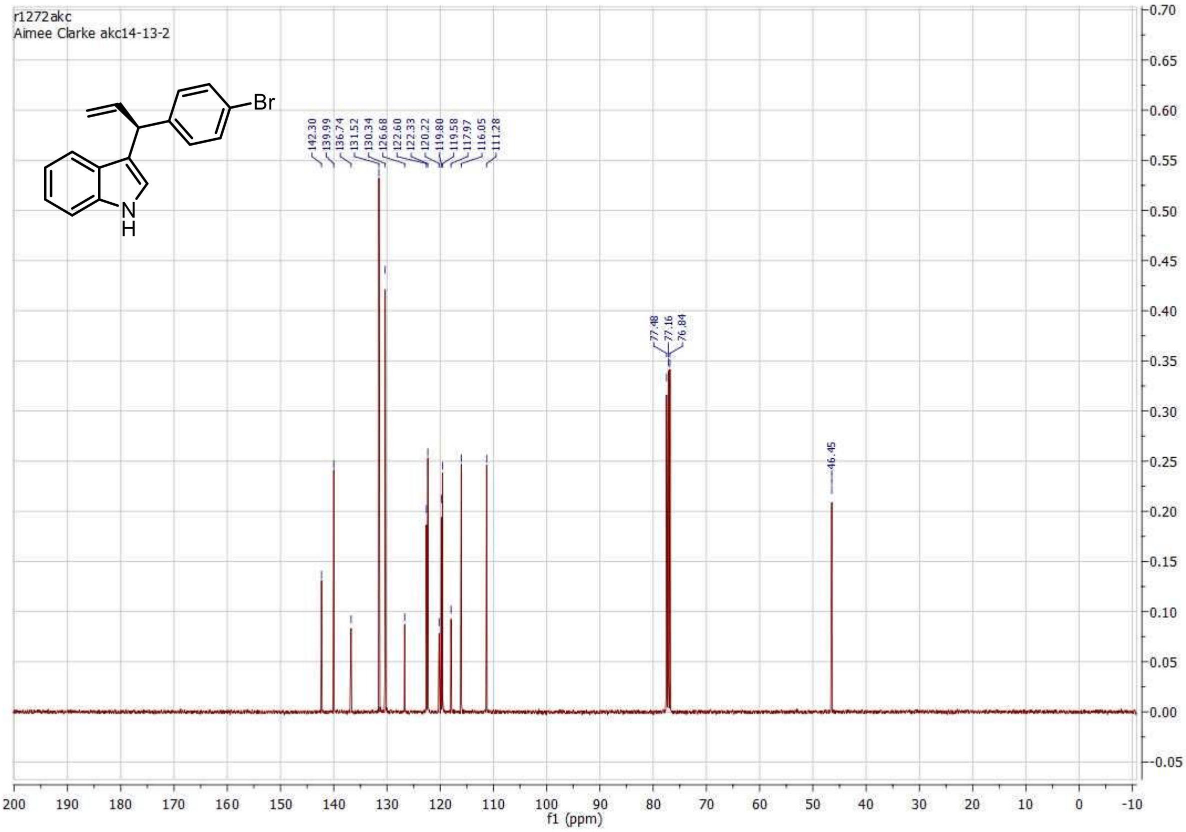
r1272akc
Aimee Clarke akc14-13-2
Br
N
H
f1 (ppm)

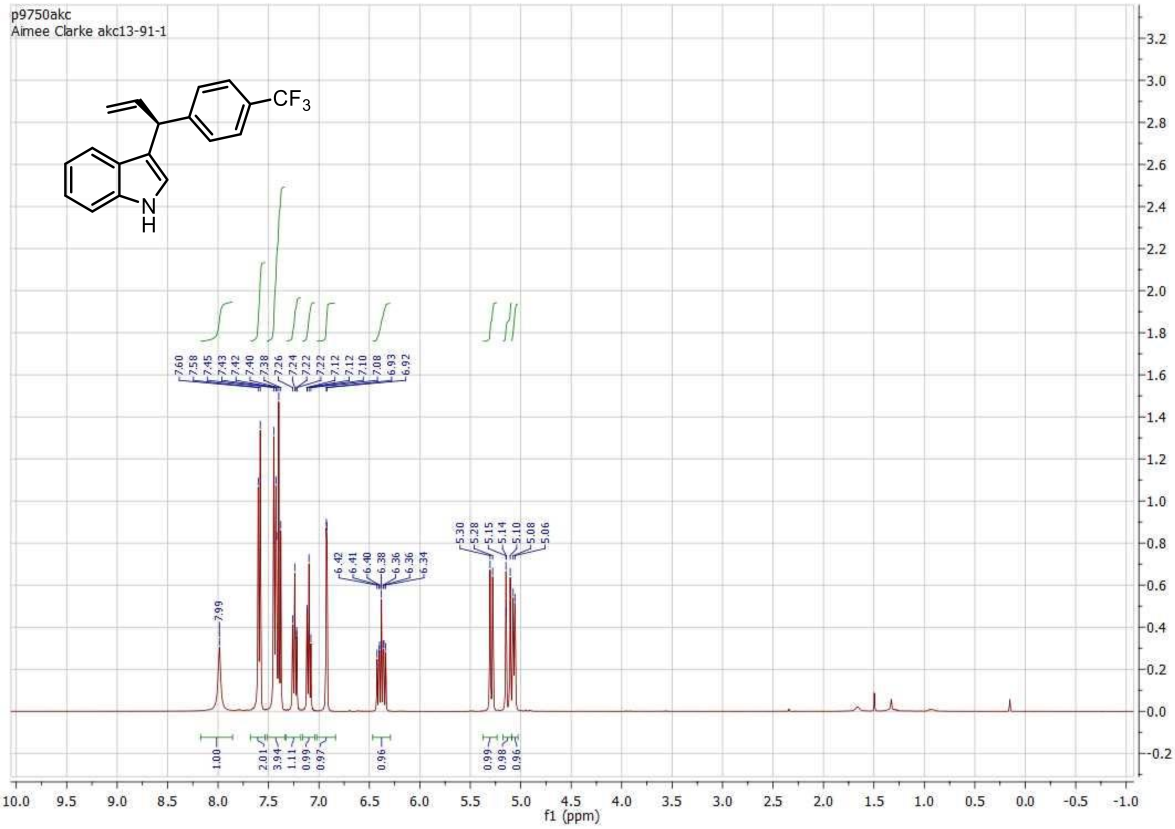
p9750akc
Aimee Clarke akc13-91-1
CF3
N
H
f1 (ppm)

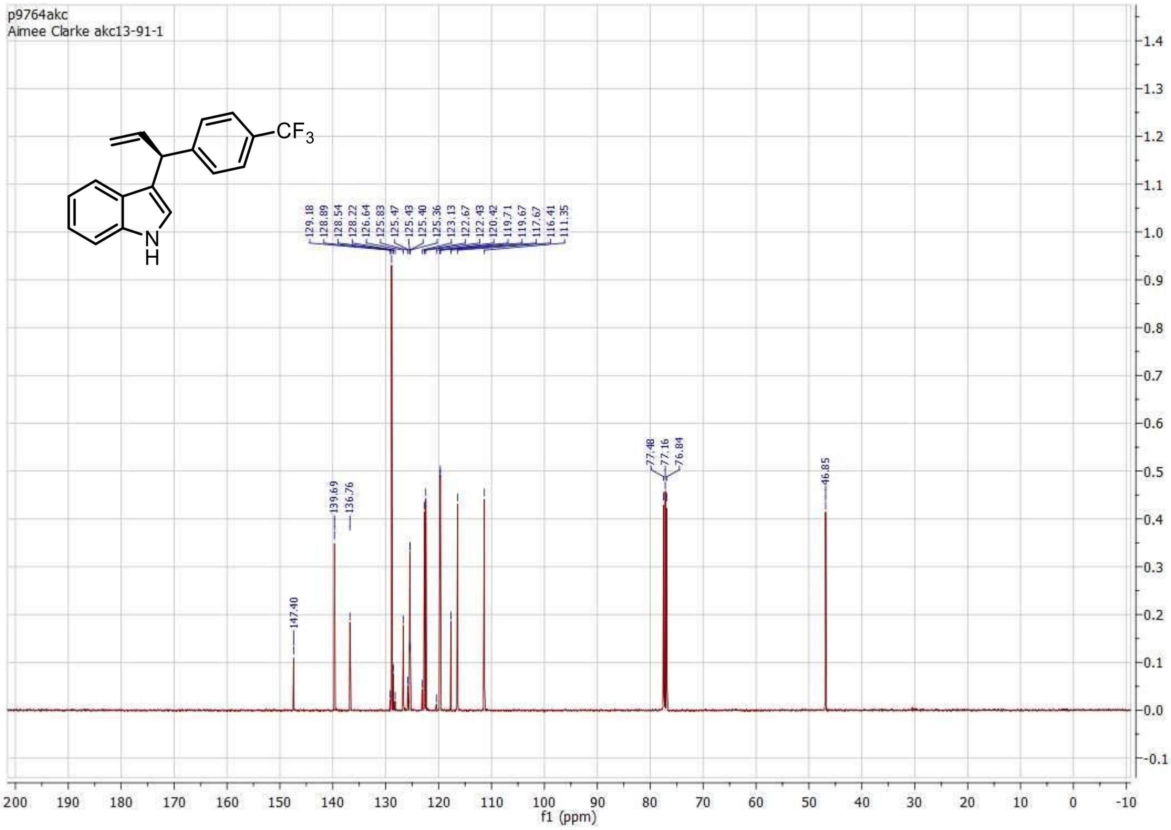
p9764akc
Aimee Clarke akc13-91-1
CF3
N
H
f1 (ppm)

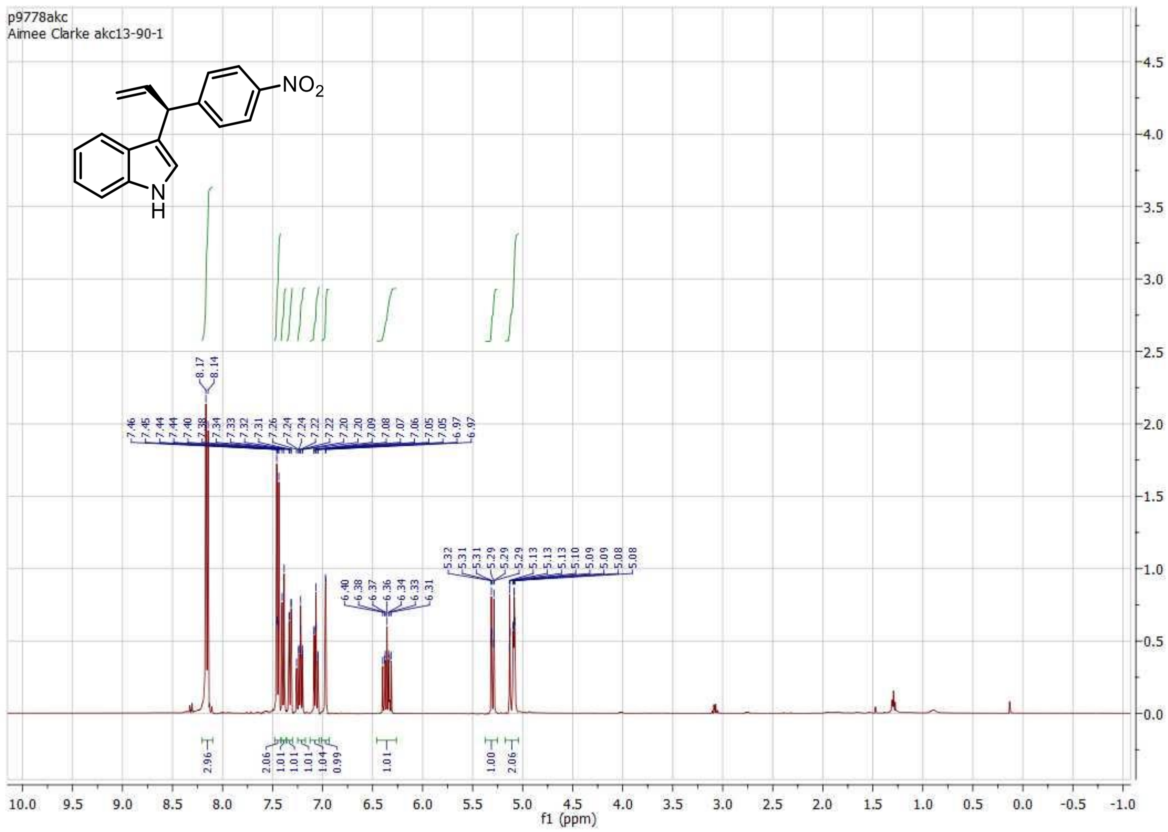
p9778akc
Aimee Clarke akc13-90-1
NO2
N
H
f1 (ppm)

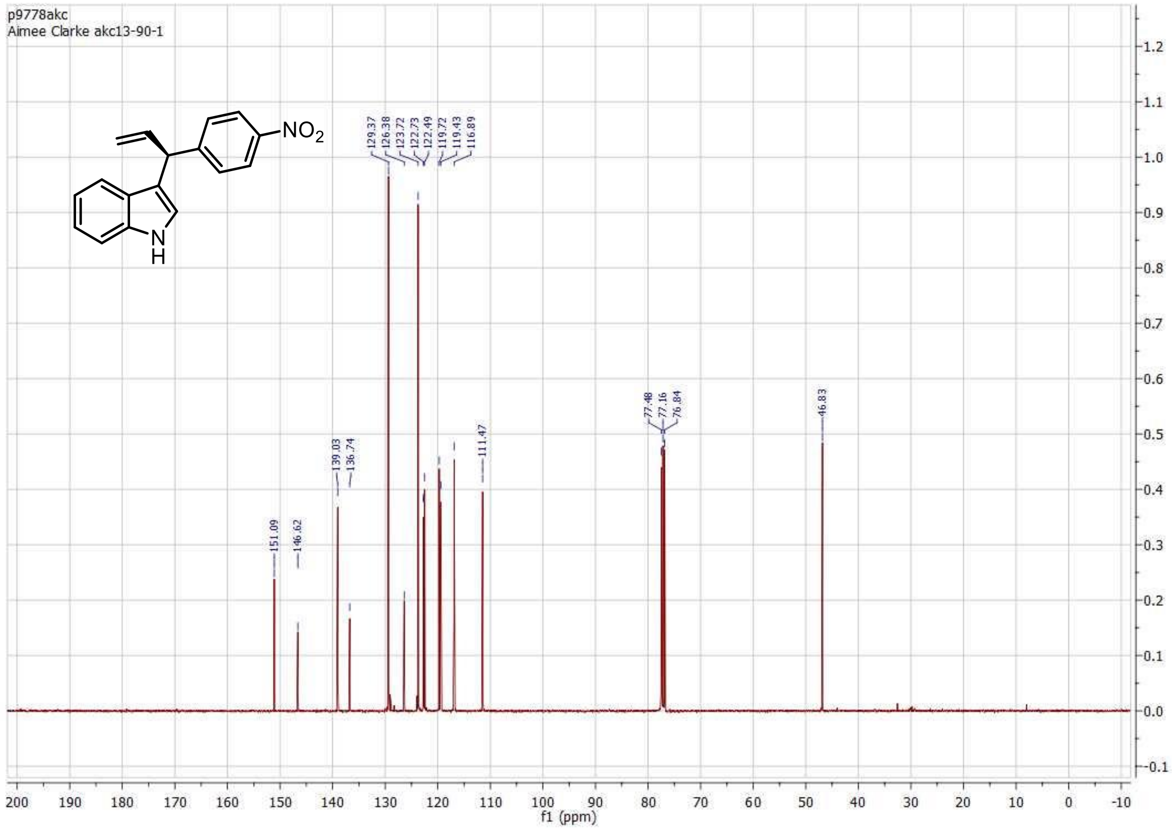
p9778akc
Aimee Clarke akc13-90-1
NO2
N
H
151.09
146.62
139.03
136.74
129.37
126.38
123.72
122.73
122.49
119.72
119.43
116.89
111.47
77.48
77.16
76.84
46.83
f1 (ppm)

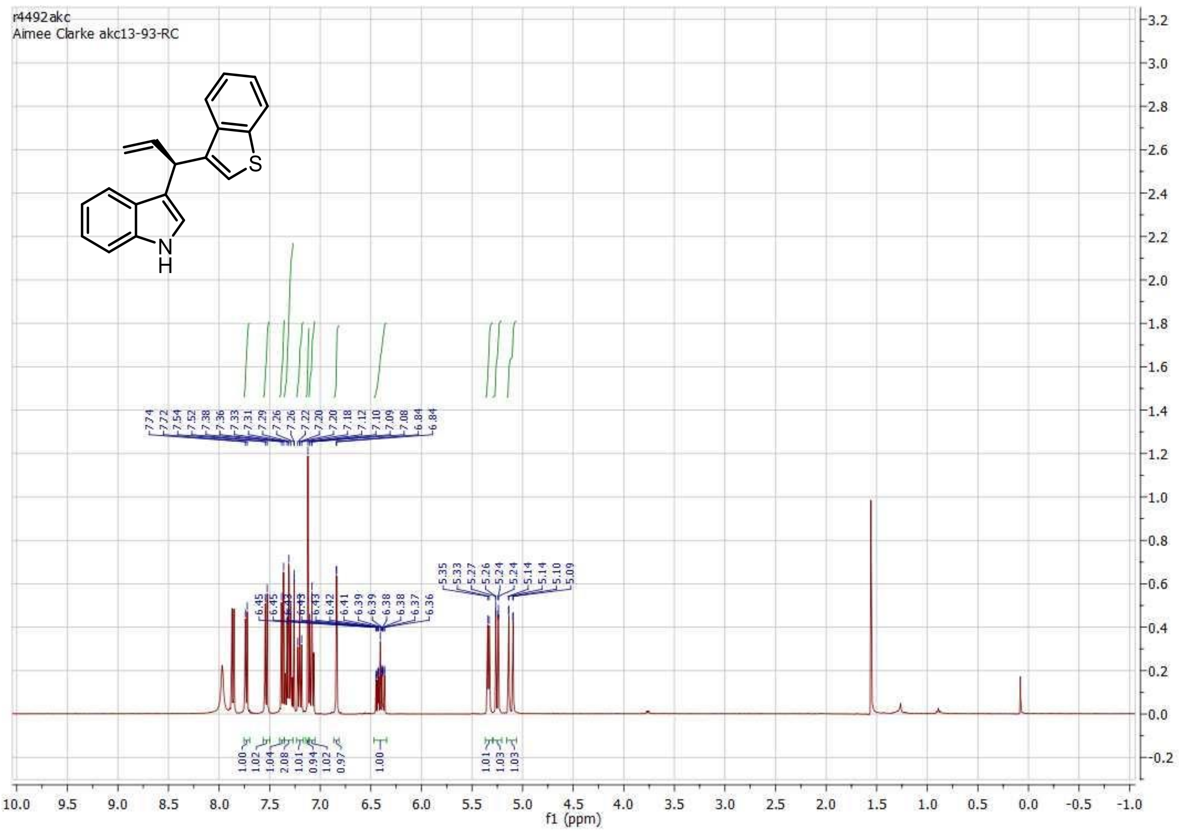
r4492akc
Aimee Clarke akc13-93-RC
S
N
H
f1 (ppm)

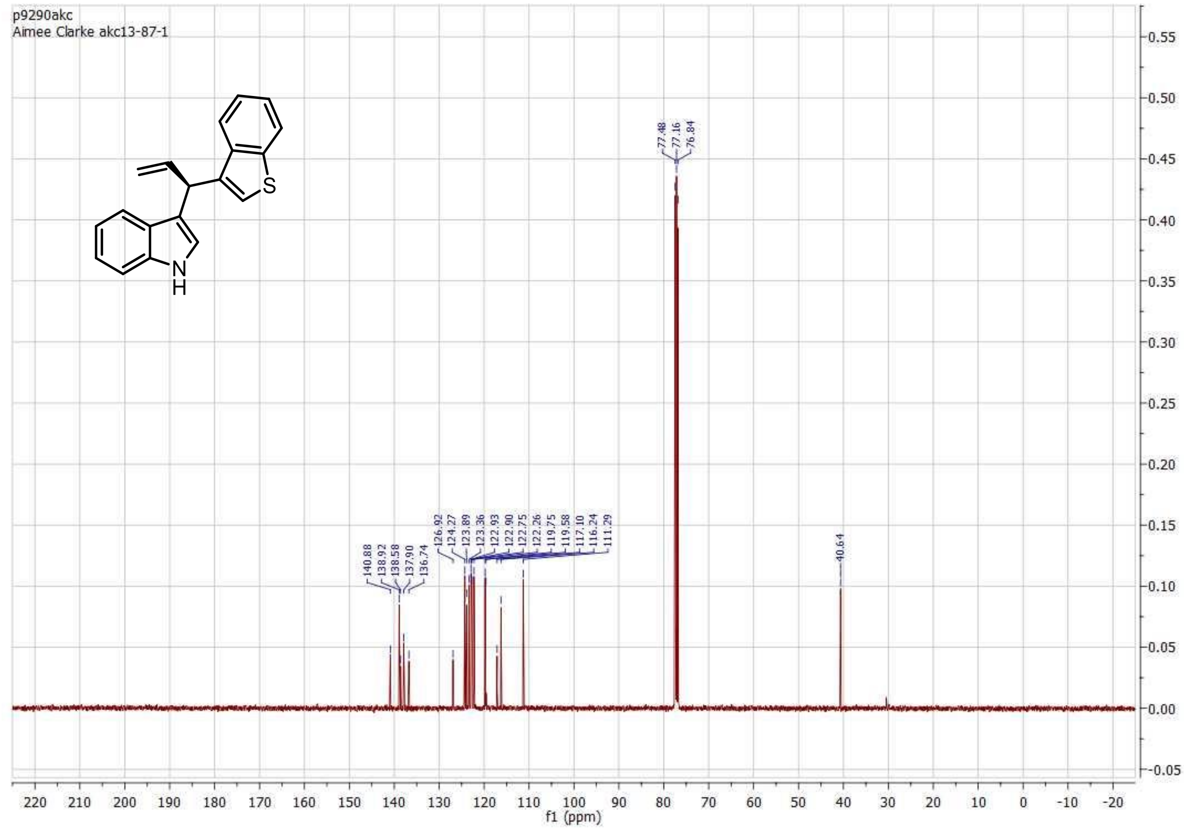
p9290akc
Aimee Clarke akc13-87-1
S
N
H
f1 (ppm)

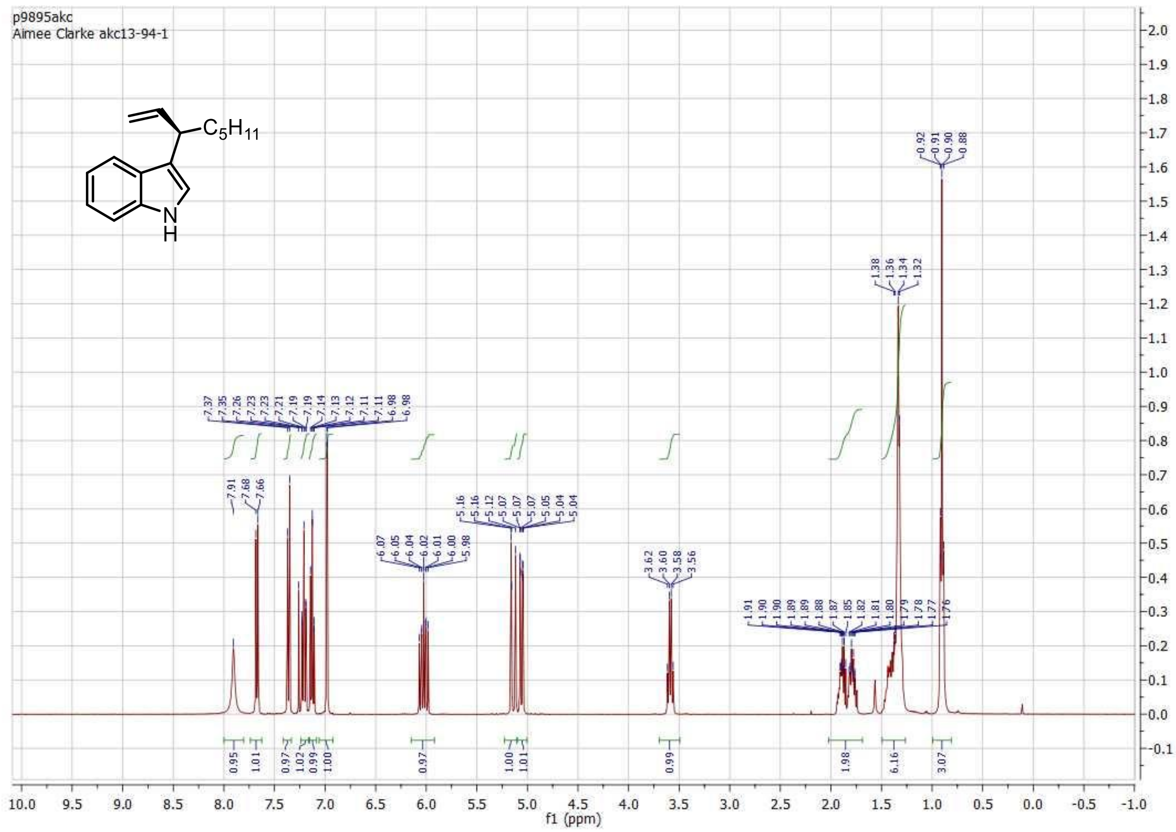
p9895akc
Aimee Clarke akc13-94-1
C5H11
N
H
f1 (ppm)

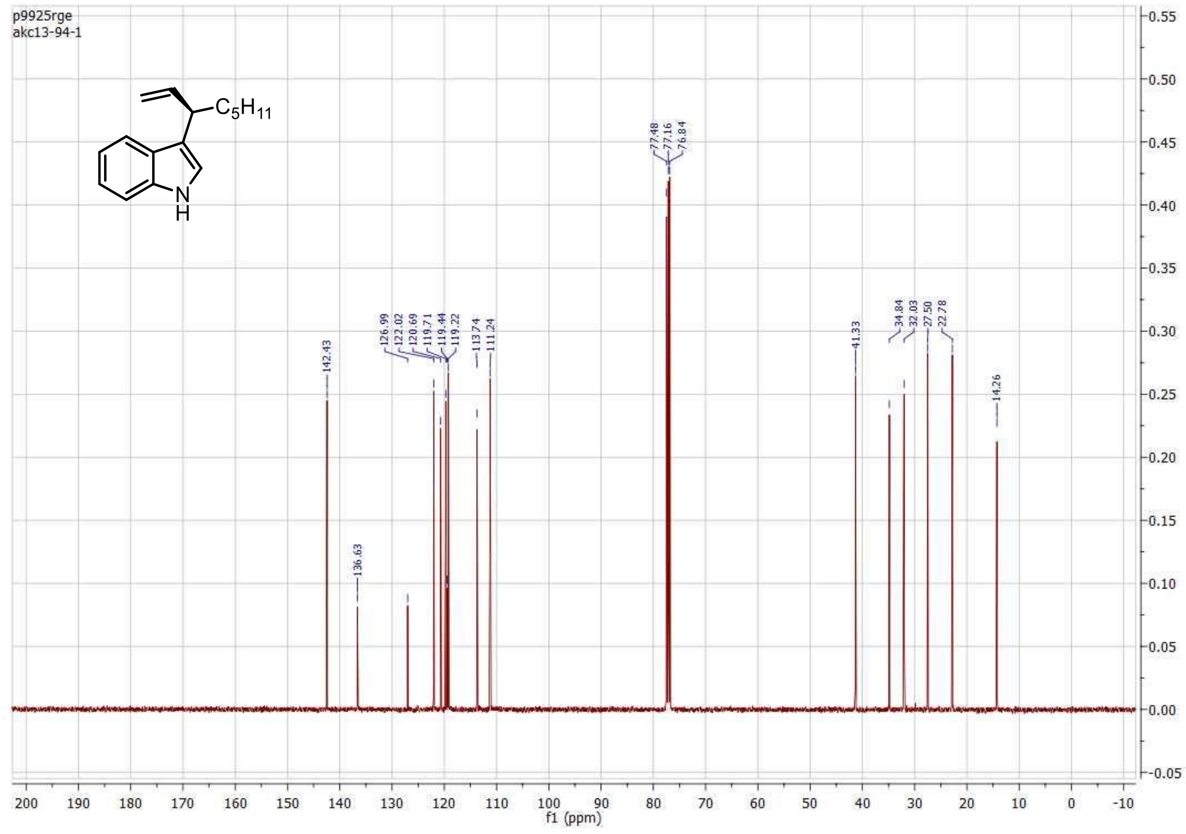
p9925rge
akc13-94-1
C5H11
N
H
f1 (ppm)

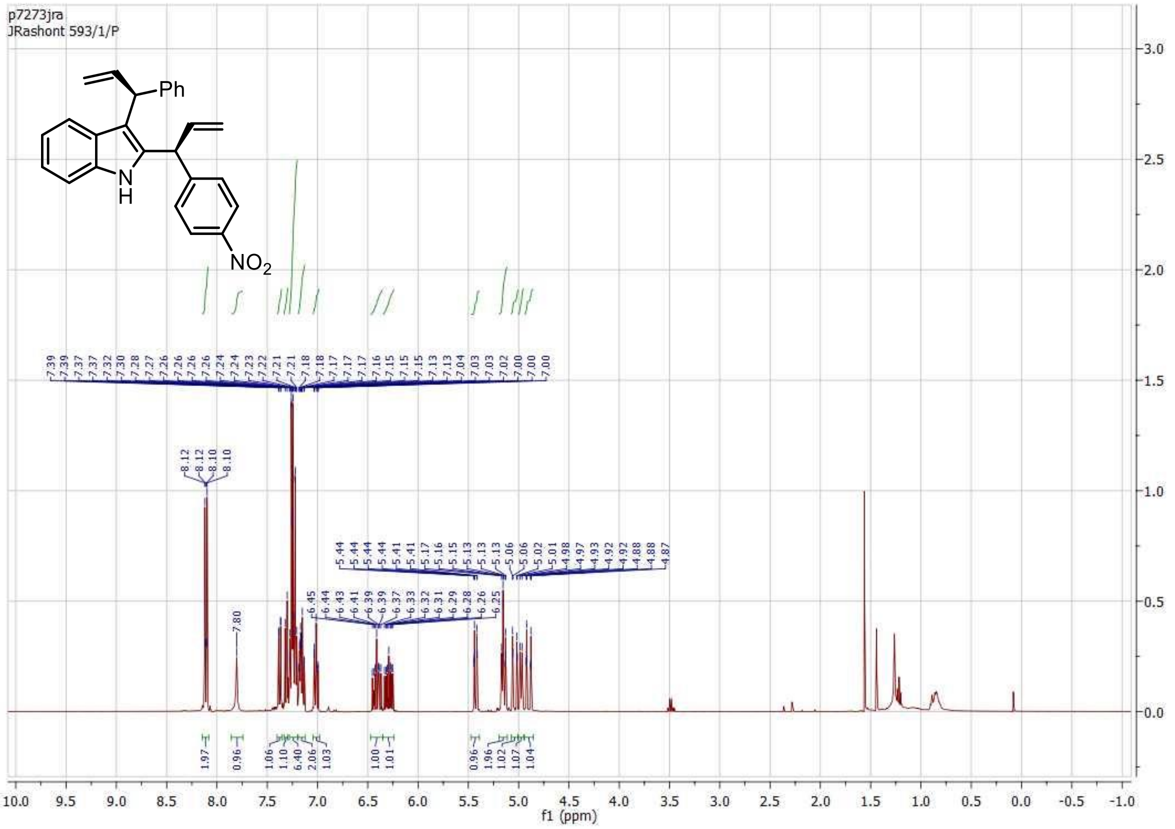
p7273jra
JRashont 593/1/P
Ph
N
H
NO2
f1 (ppm)

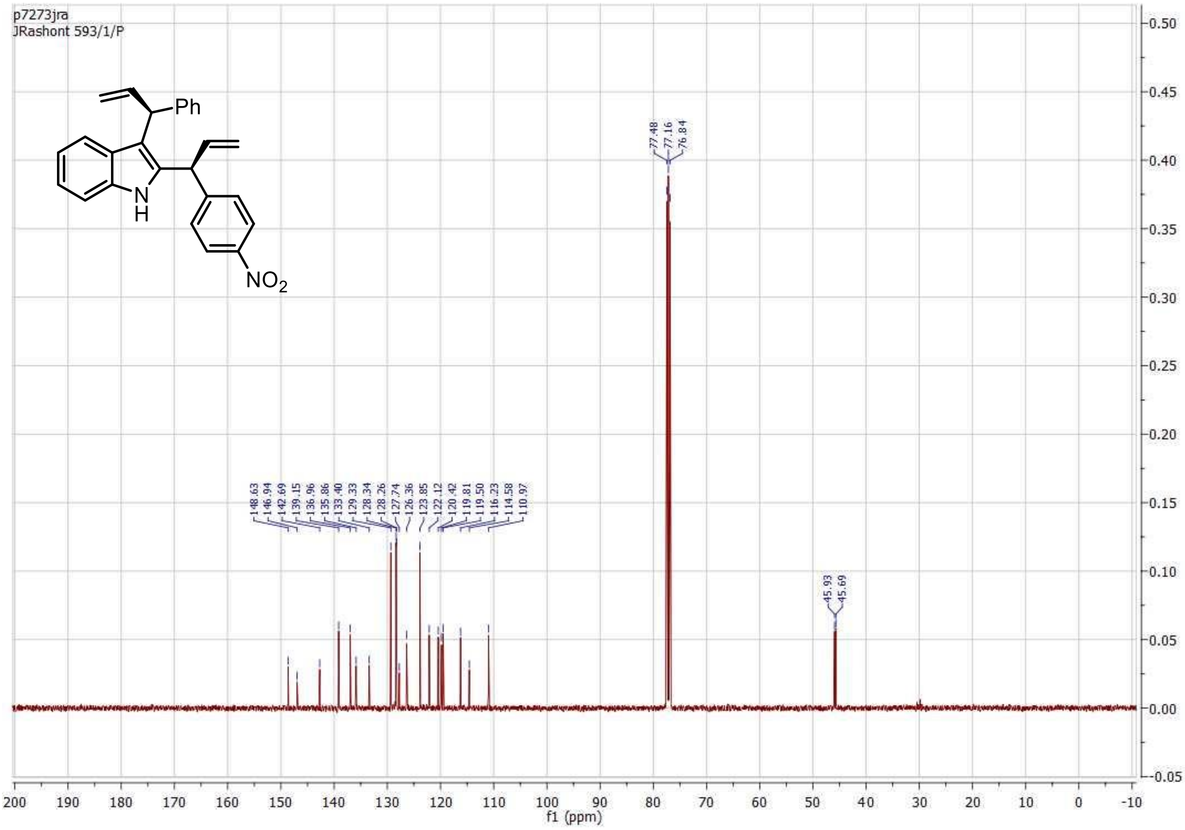
p7273jra
JRashont 593/1/P
Ph
N
H
NO2
148.63
146.94
142.69
139.15
136.96
135.86
133.40
129.33
128.34
128.26
127.74
126.36
123.85
122.12
120.42
119.81
119.50
116.23
114.58
110.97
77.48
77.16
76.84
45.93
45.69
f1 (ppm)

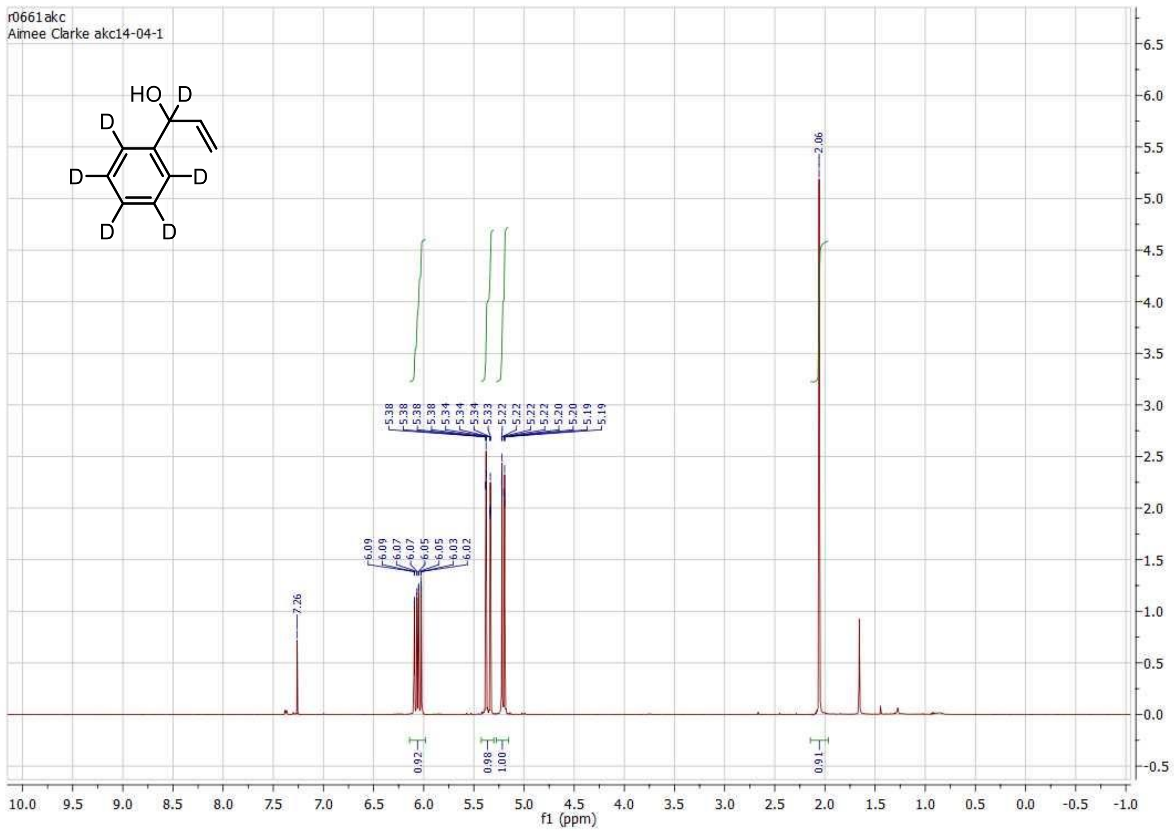
r0661akc
Aimee Clarke akc14-04-1
HO
D
D
D
D
D
D
7.26
6.09
6.09
6.07
6.07
6.05
6.05
6.03
6.02
5.38
5.38
5.38
5.38
5.34
5.34
5.34
5.33
5.22
5.22
5.22
5.22
5.22
5.20
5.20
5.19
5.19
2.06
0.92
0.98
1.00
0.91
f1 (ppm)

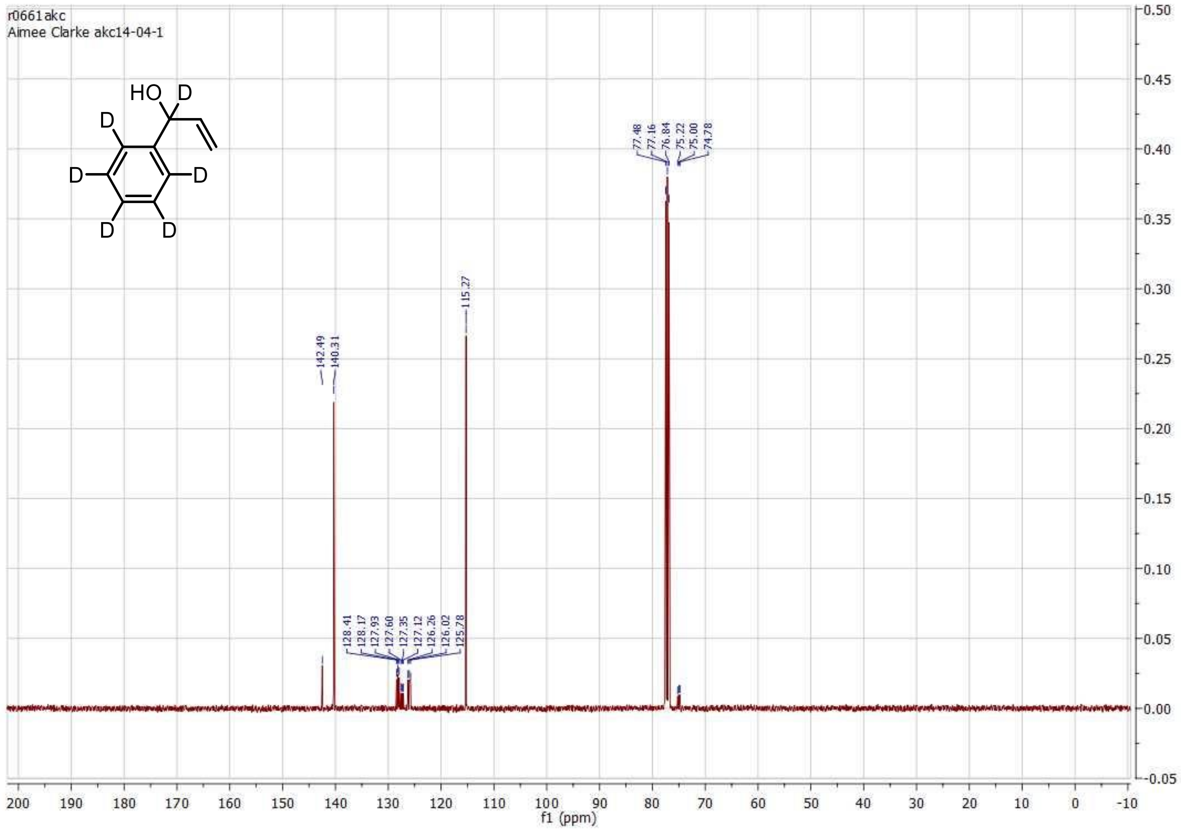
r0661akc
Aimee Clarke akc14-04-1
HO
D
D
D
D
D
D
142.49
140.31
128.41
128.17
127.93
127.60
127.35
127.12
126.26
126.02
125.78
115.27
77.48
77.16
76.84
75.22
75.00
74.78
f1 (ppm)

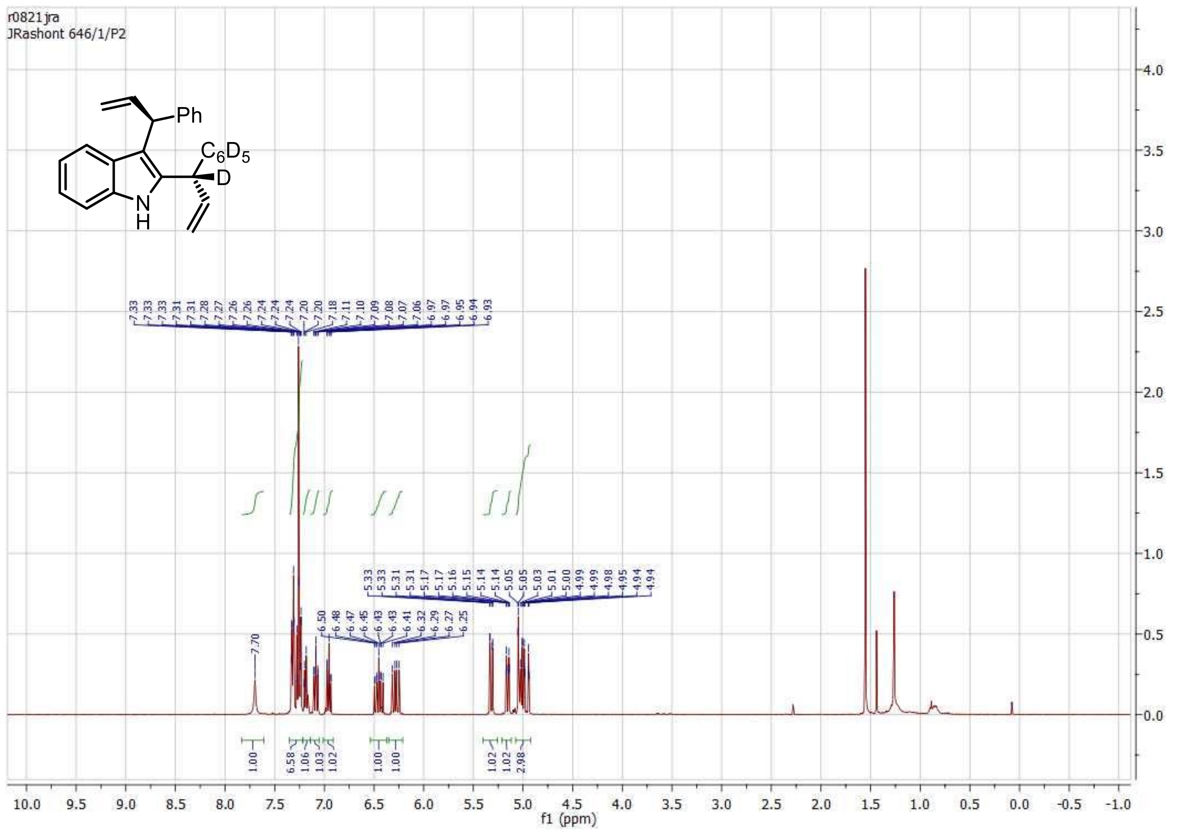
r0821jra
JRashont 646/1/P2
Ph
C_6D_5
D
N
H
f1 (ppm)

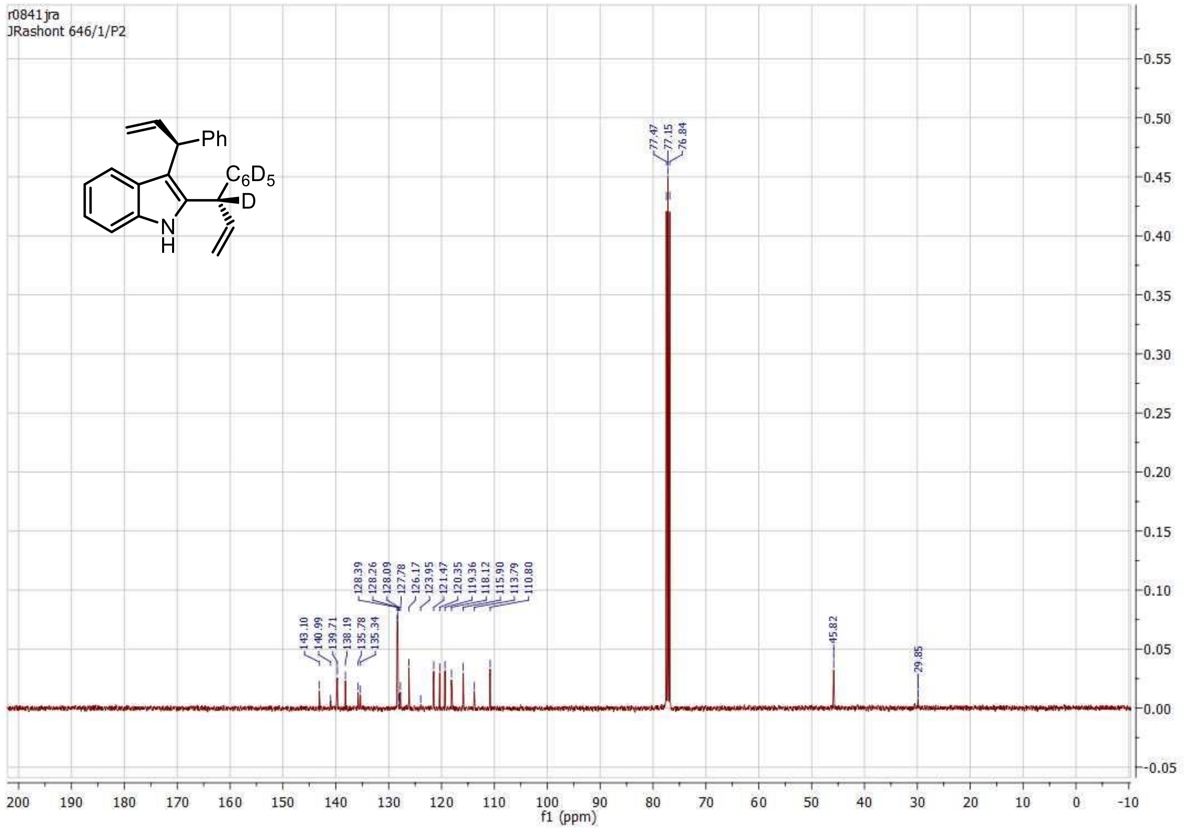
r0841jra
JRashont 646/1/P2
Ph
C_6D_5
D
N
H
f1 (ppm)

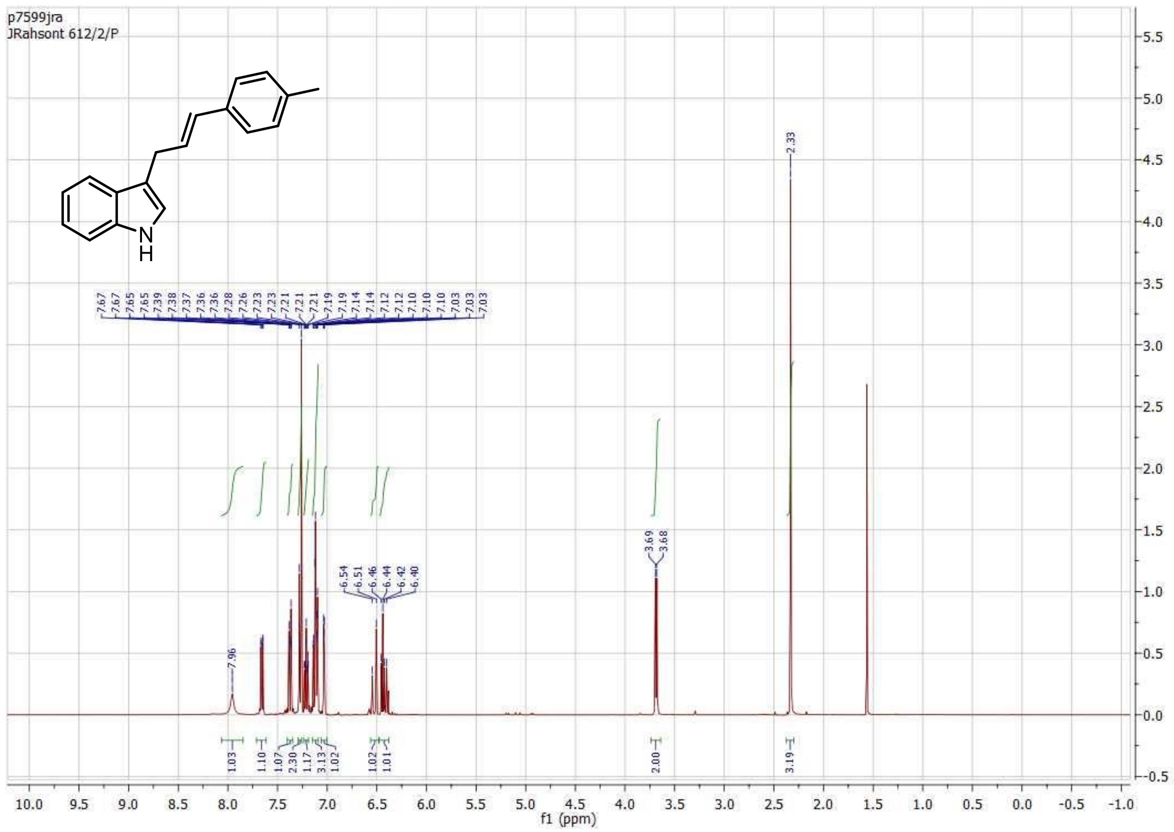
p7599jra
JRahsont 612/2/P
N
H
7.96
3.69
3.68
2.33
6.54
6.51
6.46
6.44
6.42
6.40
1.03
1.10
1.07
2.30
1.17
3.13
1.02
1.02
1.01
2.00
3.19
f1 (ppm)

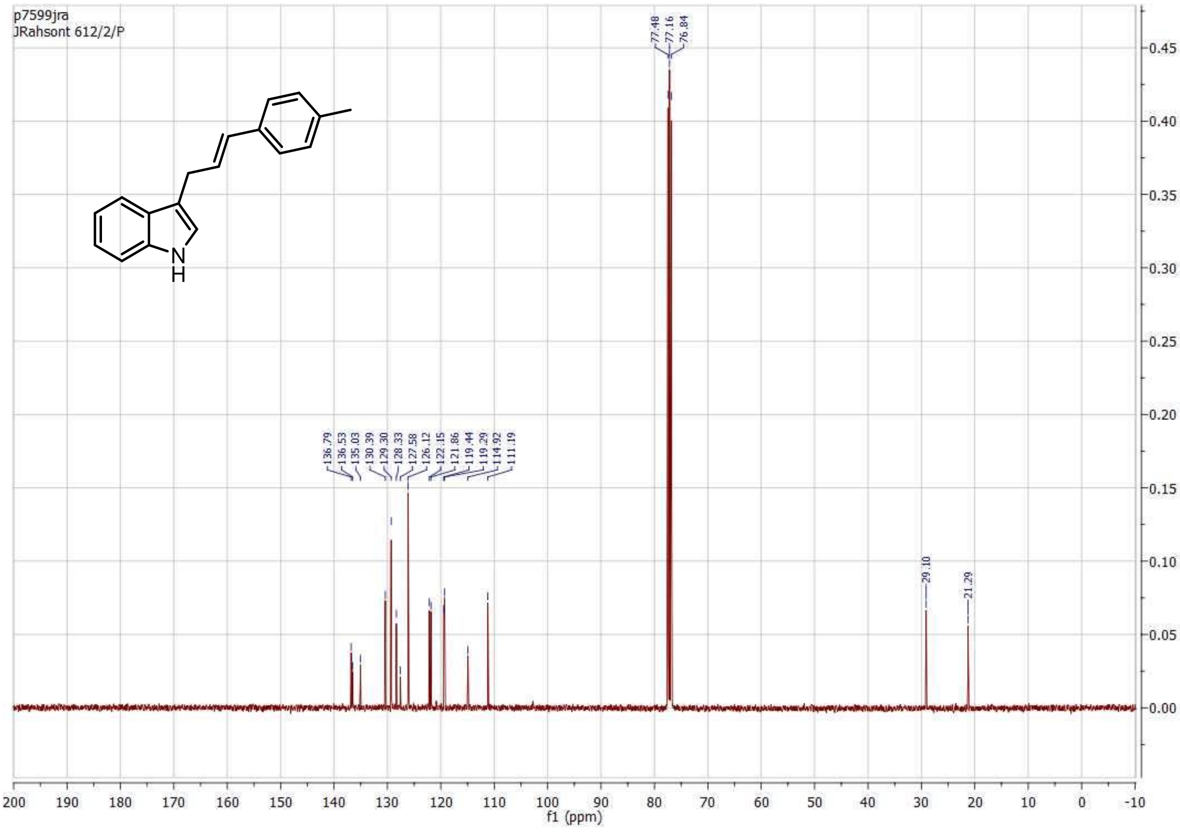
p7599jra
JRahsont 612/2/P
N
H
77.48
77.16
76.84
136.79
136.53
135.03
130.39
129.30
128.33
127.58
126.12
122.15
121.86
119.44
119.29
114.92
111.19
29.10
21.29
f1 (ppm)

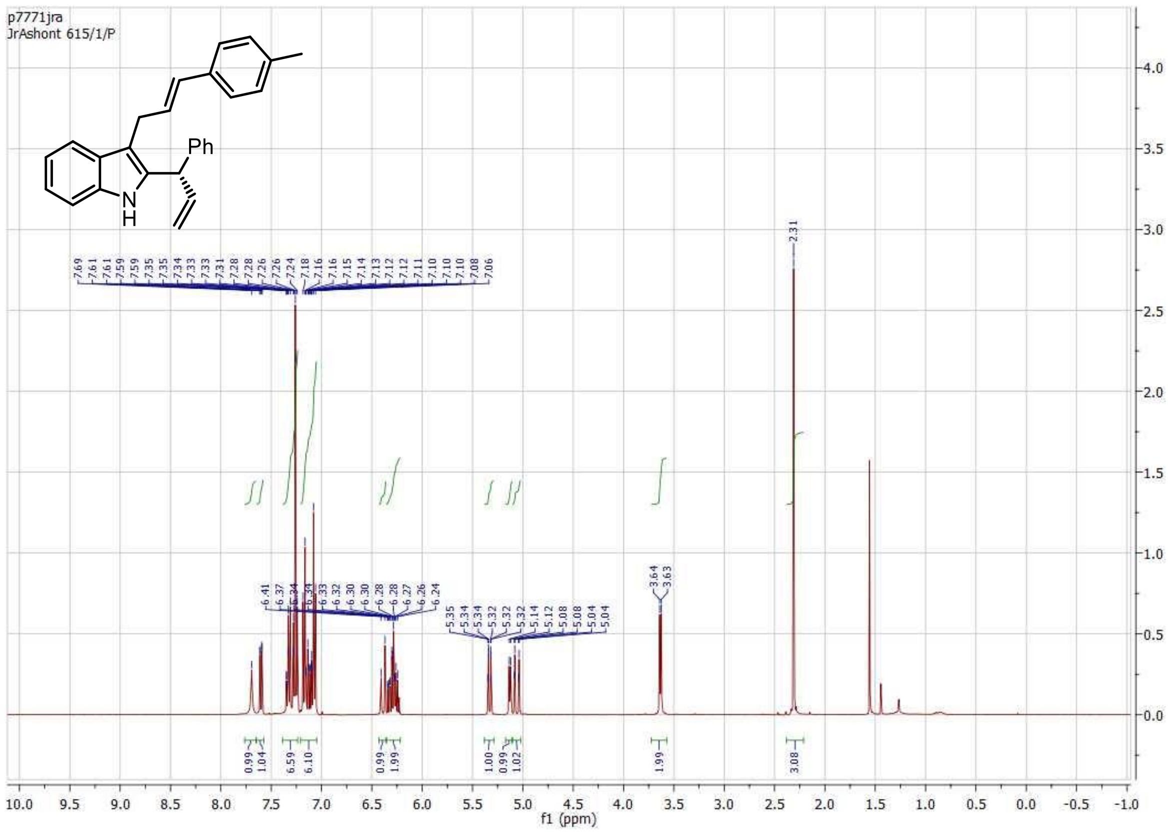
p7771jra
JrAshont 615/1/P
Ph
N
H
f1 (ppm)

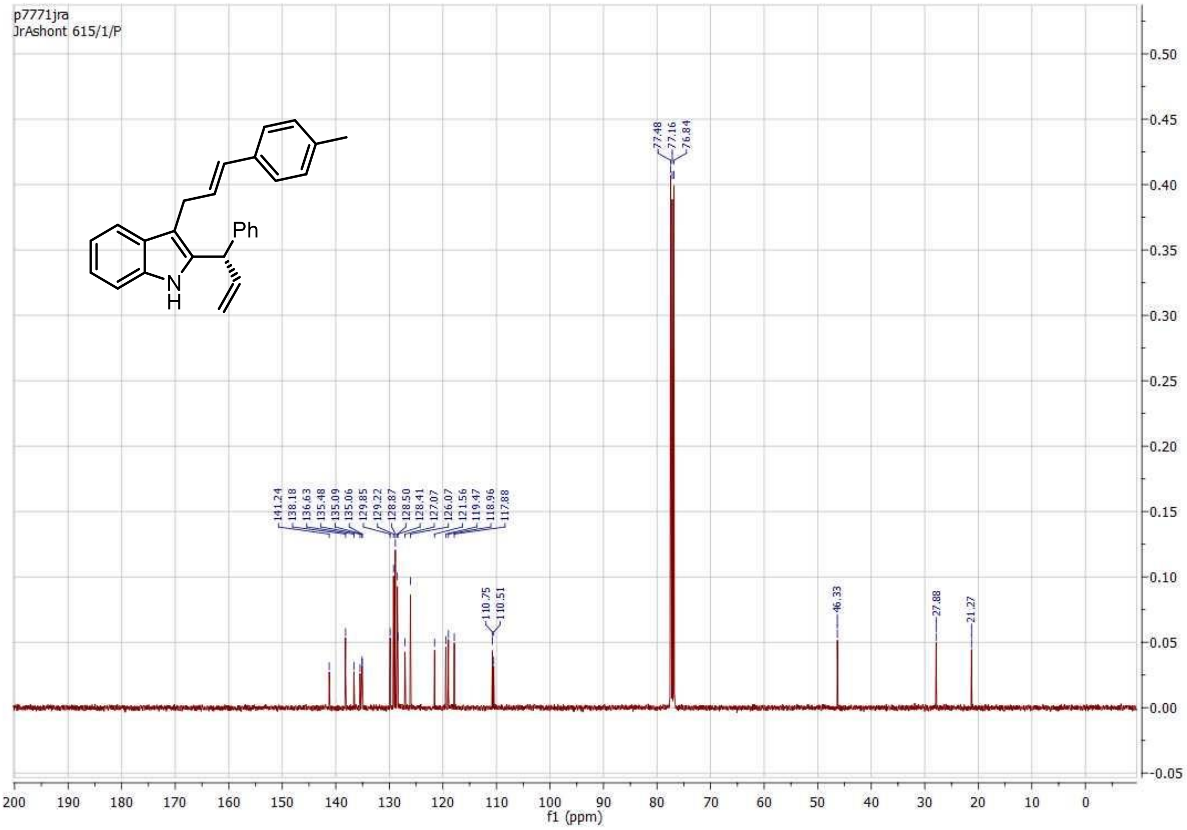
p7771jra
JrAshont 615/1/P
Ph
N
H
f1 (ppm)

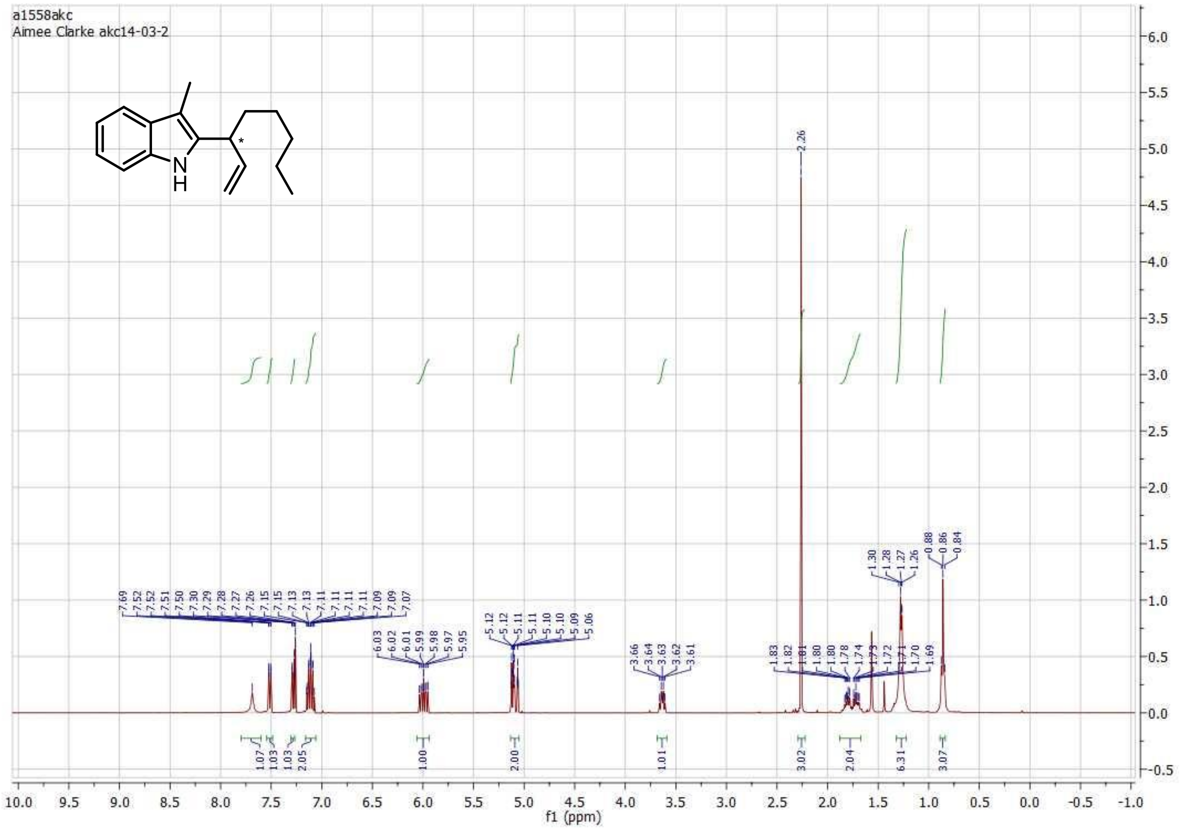
a1558akc
Aimee Clarke akc14-03-2
N
H
*
7.69 7.52 7.52 7.51 7.50 7.30 7.29 7.28 7.27 7.26 7.15 7.15 7.13 7.13 7.11 7.11 7.11 7.11 7.09 7.09 7.07
6.03 6.02 6.01 5.99 5.98 5.97 5.95
5.12 5.12 5.11 5.11 5.10 5.10 5.09 5.06
3.66 3.64 3.63 3.62 3.61
2.26
1.83 1.82 1.81 1.80 1.78 1.74 1.73 1.72 1.71 1.70 1.69
1.30 1.28 1.27 1.26
0.88 0.86 0.84
1.07 1.03 1.03 2.05 1.00 2.00 1.01 3.02 2.04 6.31 3.07
f1 (ppm)

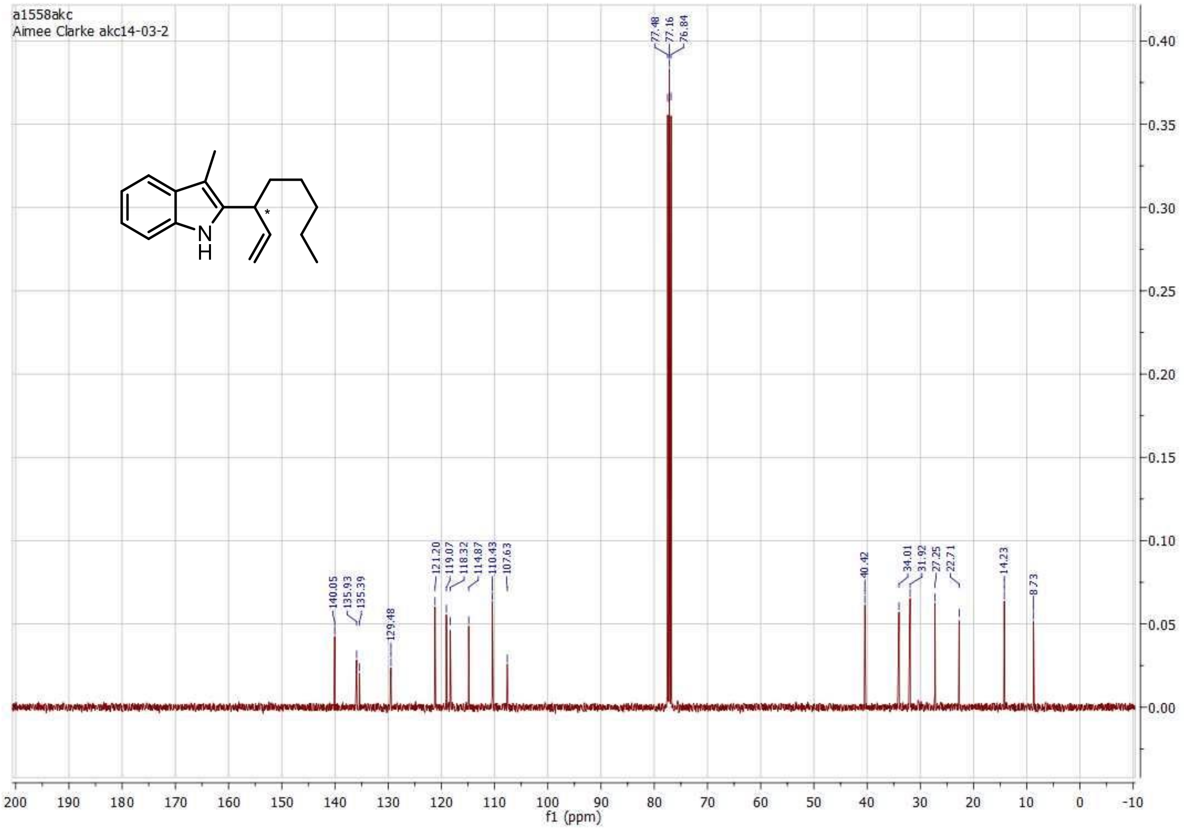
a1558akc
Aimee Clarke akc14-03-2
N
H
*
77.48 77.16 76.84
140.05 135.93 135.39 129.48 121.20 119.07 118.32 114.87 110.43 107.63
40.42 34.01 31.92 27.25 22.71 14.23 8.73
f1 (ppm)

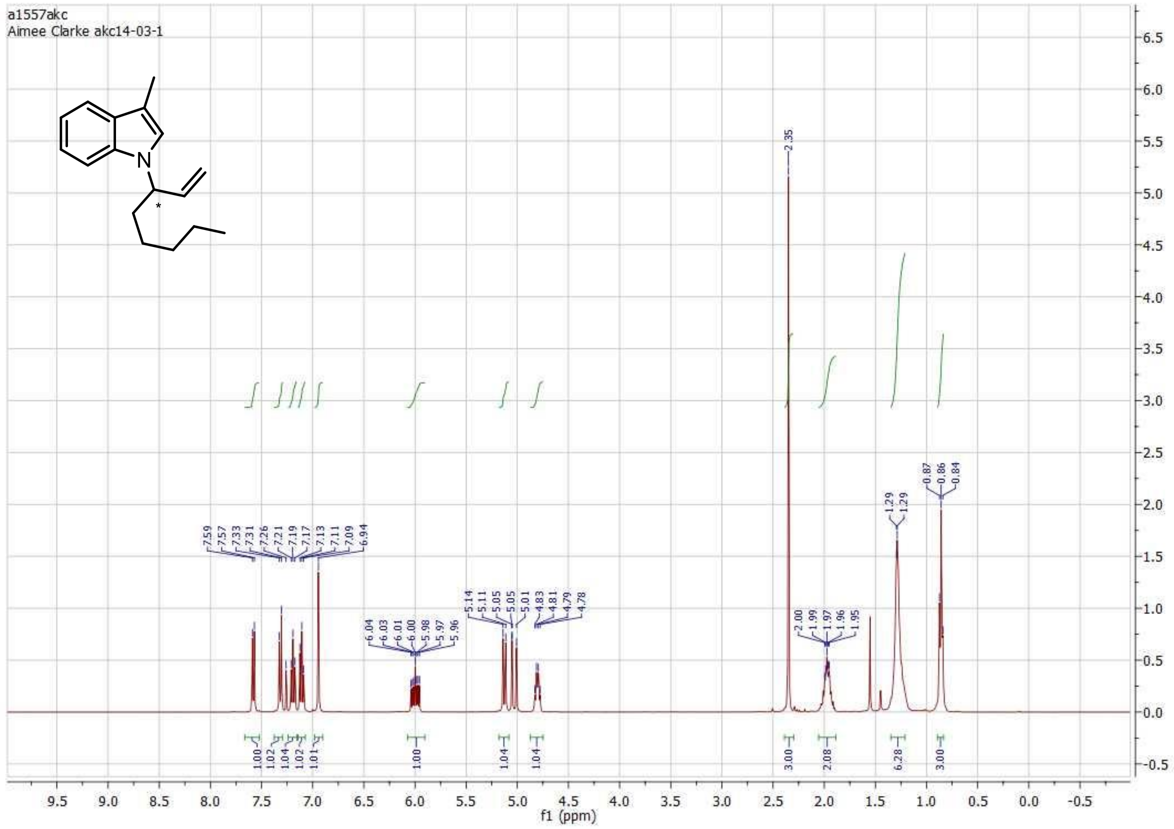
a1557akc
Aimee Clarke akc14-03-1
N
f1 (ppm)

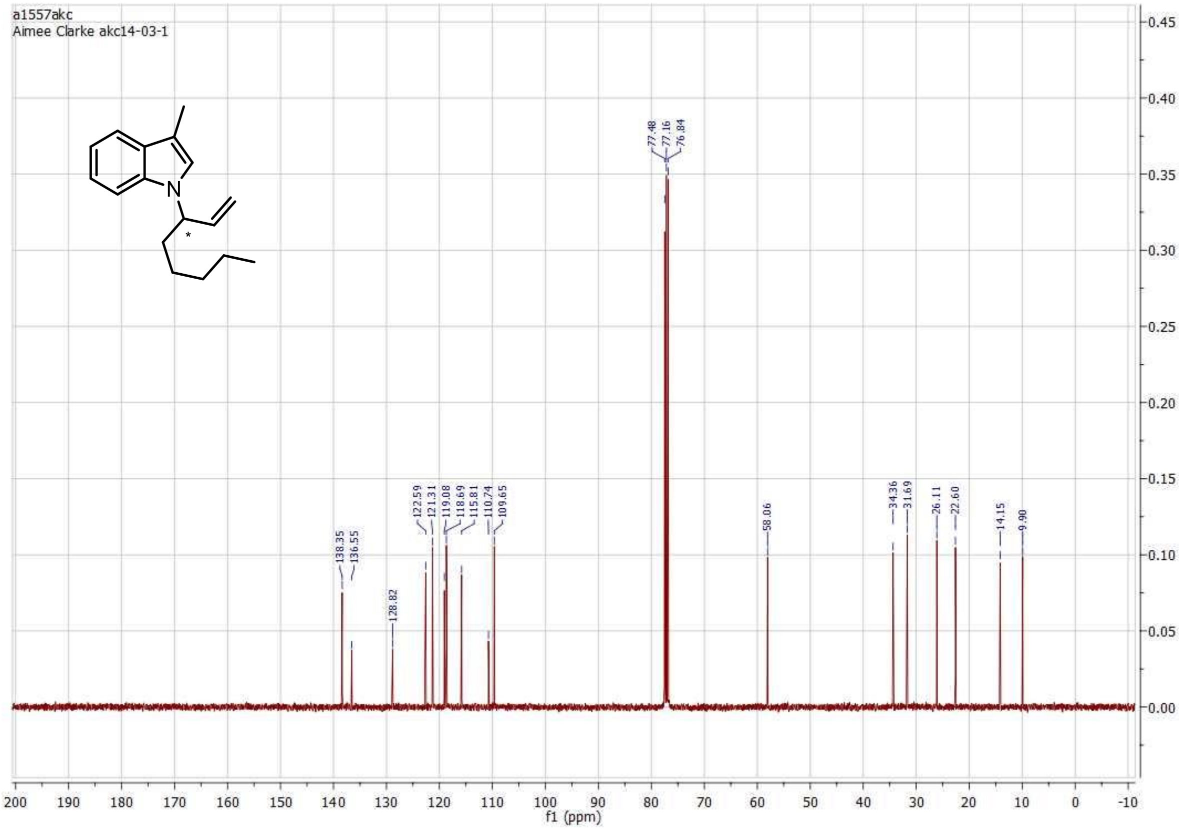
a1557akc
Aimee Clarke akc14-03-1
N
f1 (ppm)

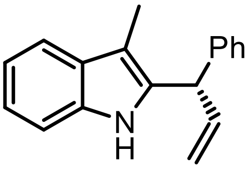

3a

CSP-HPLC of **3a** using Chiralpak OD-H column, eluting with 5% IPA in hexanes.

Racemic **3a** recorded at 254 nm

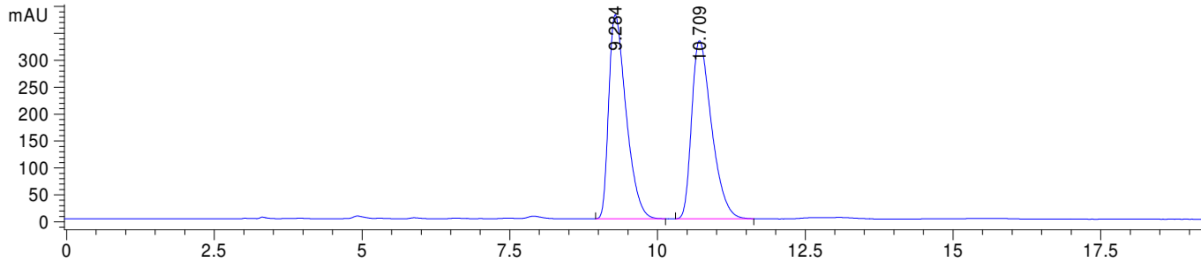

Peak No.	Retention Time / min	Peak Area	Peak Area / %
1	9.284	7756	49.9
2	10.709	7777	50.1

(−)3a recorded at 254 nm

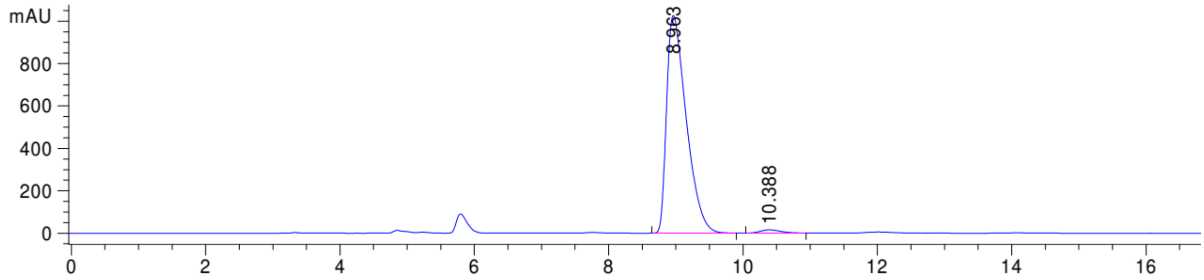

Peak No.	Retention Time / min	Peak Area	Peak Area / %
1	8.963	20854	98.5
2	10.388	313	1.5

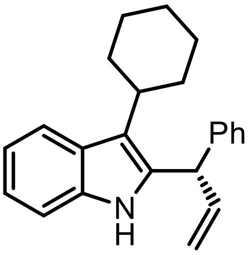

3b

CSP-HPLC of **3b** using Chiralpak OD-H column, eluting with 3% IPA in hexanes.

Racemic **3b** recorded at 280 nm

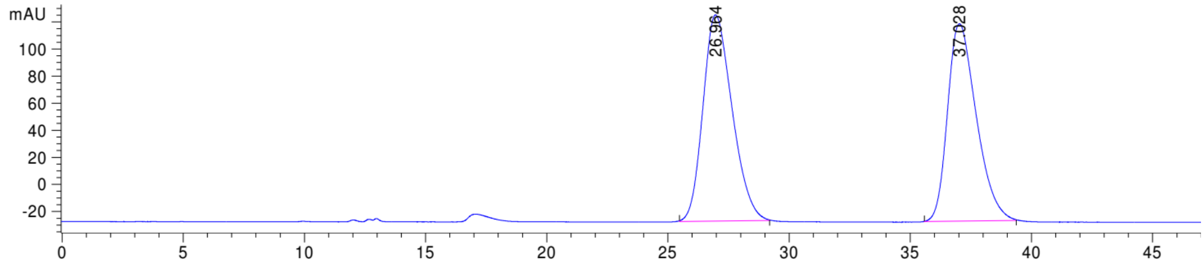

Peak No.	Retention Time / min	Peak Area	Peak Area / %
1	26.963	36904	51.3
2	37.026	34978	48.7

(−)3b recorded at 280 nm

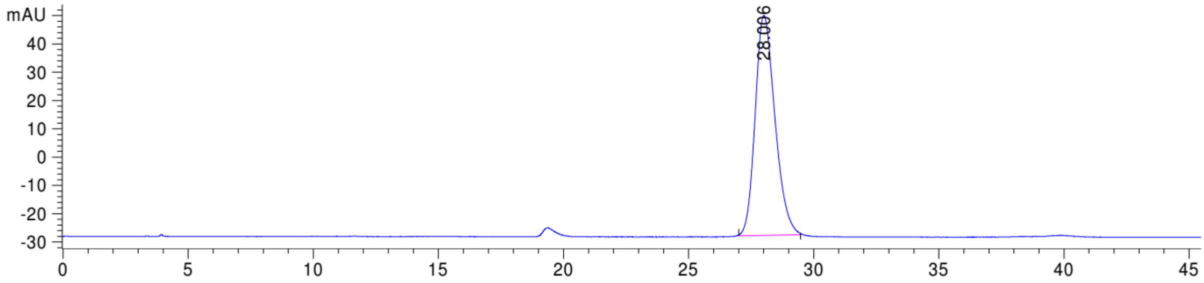

Peak No.	Retention Time / min	Peak Area	Peak Area / %
1	28.006	4270	100.00
2	-	-	-

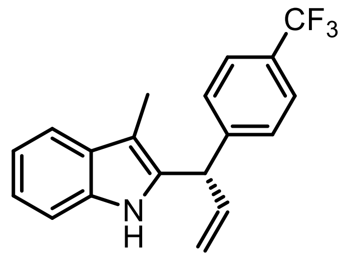

3c

CSP-HPLC of **3c** using Chiralpak OD-H column, eluting with 5% IPA in hexanes.

Racemic **3c** recorded at 254 nm

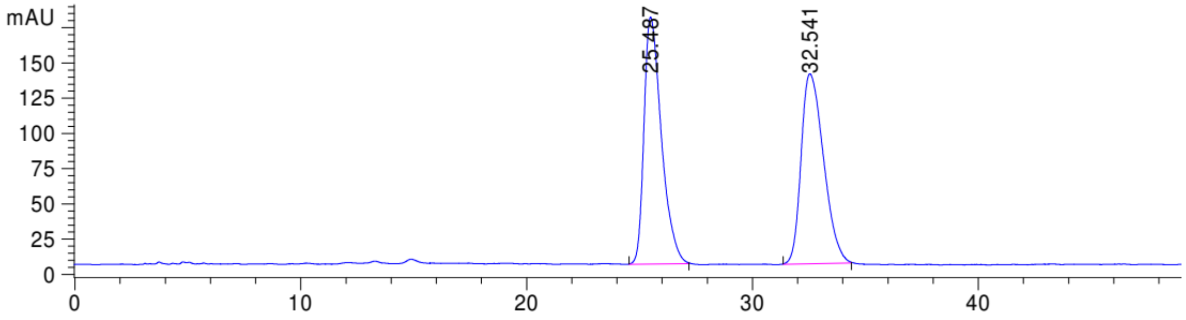

Peak No.	Retention Time / min	Peak Area	Peak Area / %
1	25.487	9371	50.1
2	32.541	9327	49.9

(+)3c recorded at 254 nm

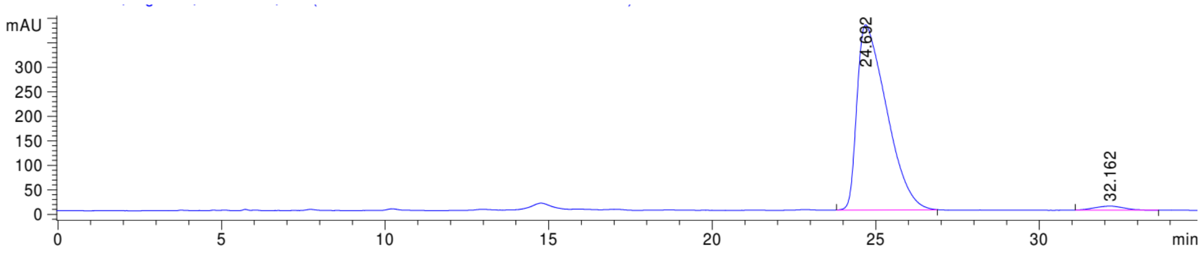

Peak No.	Retention Time / min	Peak Area	Peak Area / %
1	24.692	23740	98.0
2	32.162	496	2.0

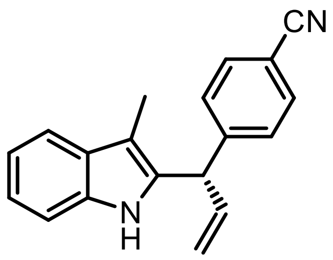

3d

CSP-HPLC of **3d** using Chiralpak OD column, eluting with 10% IPA in hexanes.

Racemic **3d** recorded at 254 nm

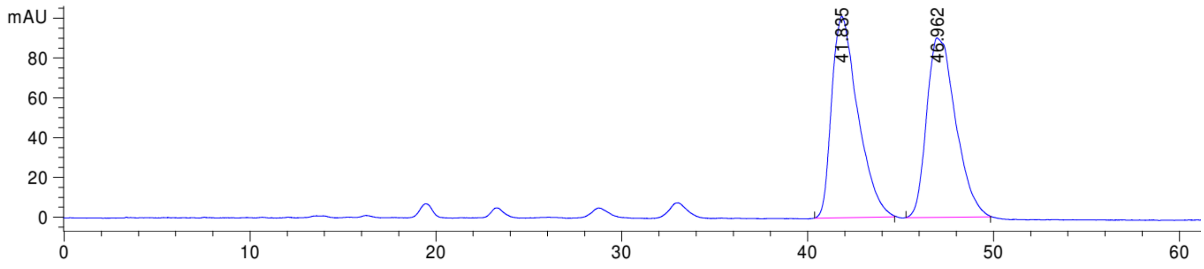

Peak No.	Retention Time / min	Peak Area	Peak Area / %
1	41.835	9904	50.2
2	46.962	9821	49.8

(+)3d recorded at 254 nm

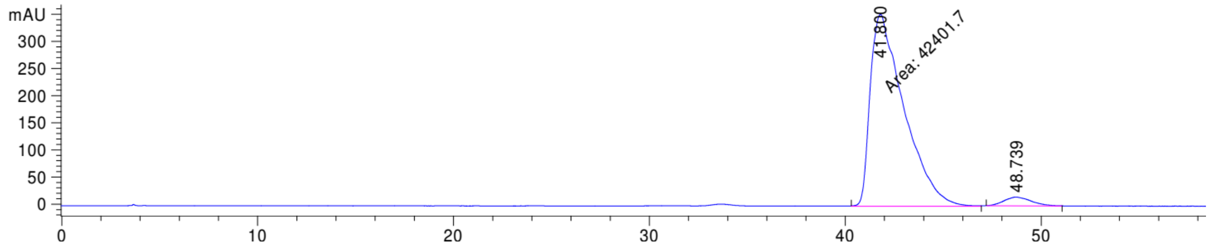

Peak No.	Retention Time / min	Peak Area	Peak Area / %
1	41.800	42402	96.5
2	48.739	1531	3.5

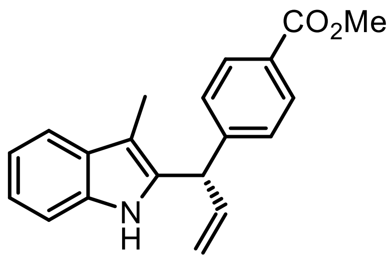

3e

CSP-HPLC of **3e** using Chiralpak AD-H column, eluting with 5% IPA in hexanes.

Racemic **3e** recorded at 254 nm

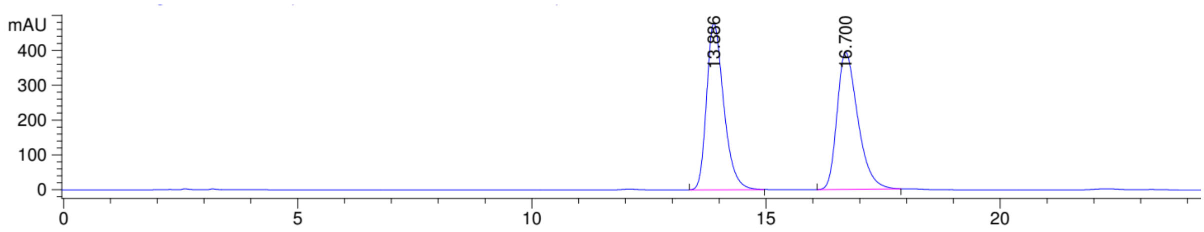

Peak No.	Retention Time / min	Peak Area	Peak Area / %
1	13.886	12171	50.1
2	16.700	12121	49.9

(−)3e recorded at 254 nm

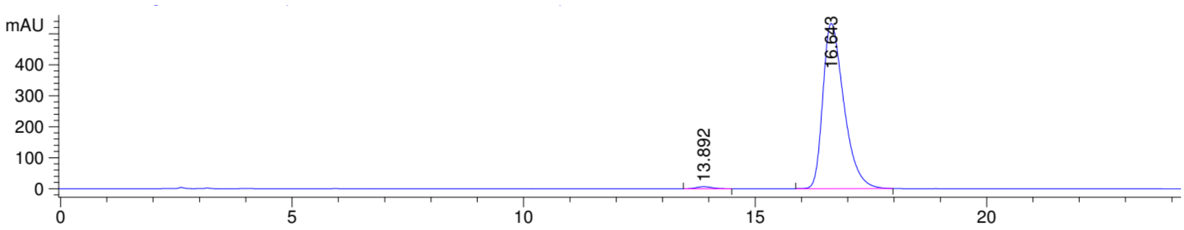

Peak No.	Retention Time / min	Peak Area	Peak Area / %
1	13.892	164	1.0
2	16.643	16863	99.0

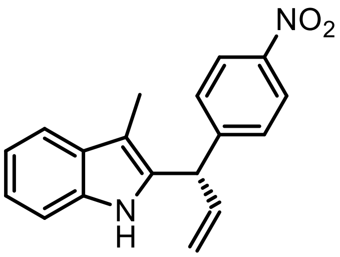

3f

CSP-HPLC of **3f** using Chiralpak IB column, eluting with 15% IPA in hexanes.

Racemic **3f** recorded at 254 nm

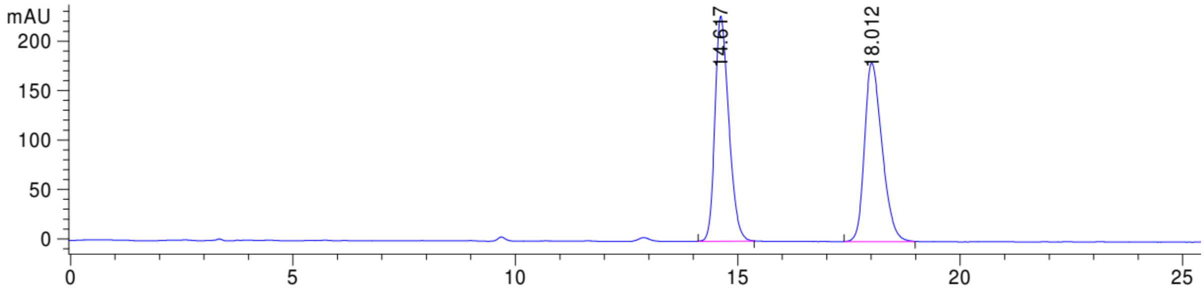

Peak No.	Retention Time / min	Peak Area	Peak Area / %
1	14.617	4937	49.9
2	18.012	4965	50.1

(+)3f recorded at 254 nm

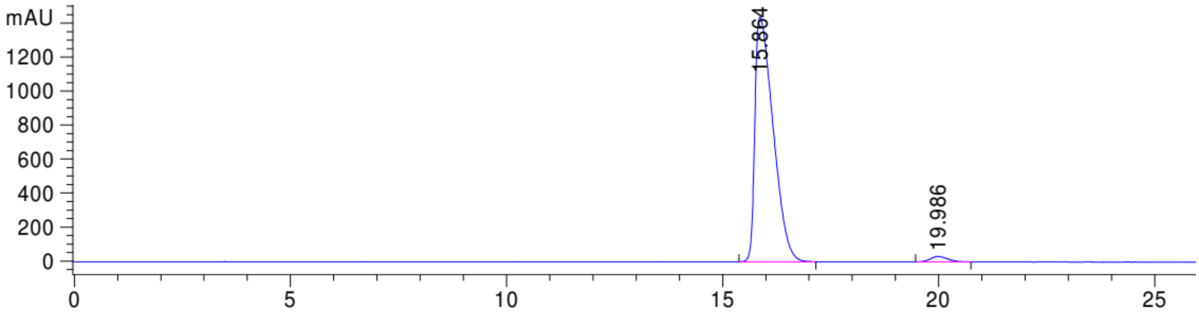

Peak No.	Retention Time / min	Peak Area	Peak Area / %
1	15.864	42257	97.9
2	19.986	917	2.1

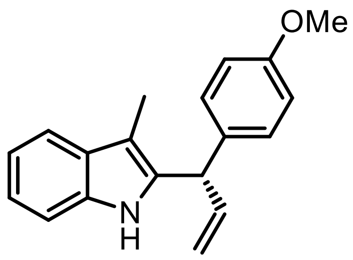

3g

CSP-HPLC of **3g** using Chiralpak OD-H column, eluting with 5% IPA in hexanes.

Racemic **3g** recorded at 254 nm

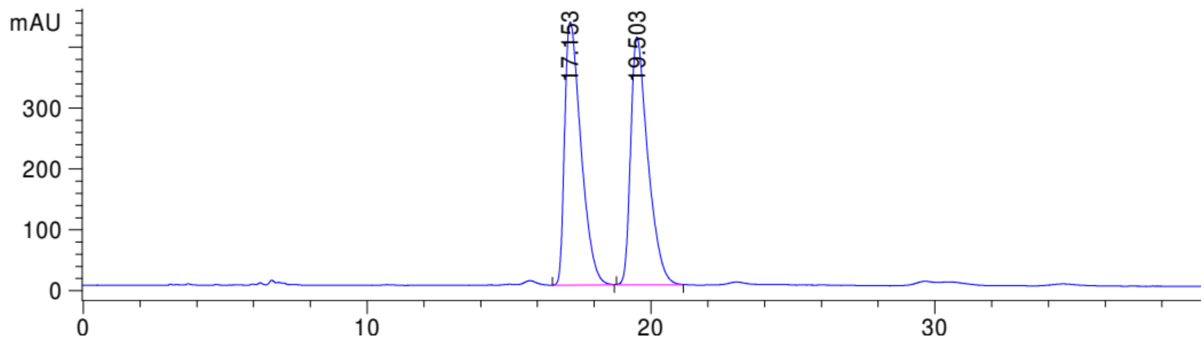

Peak No.	Retention Time / min	Peak Area	Peak Area / %
1	17.153	17334	50.2
2	19.503	17194	49.8

(−)3g recorded at 254 nm

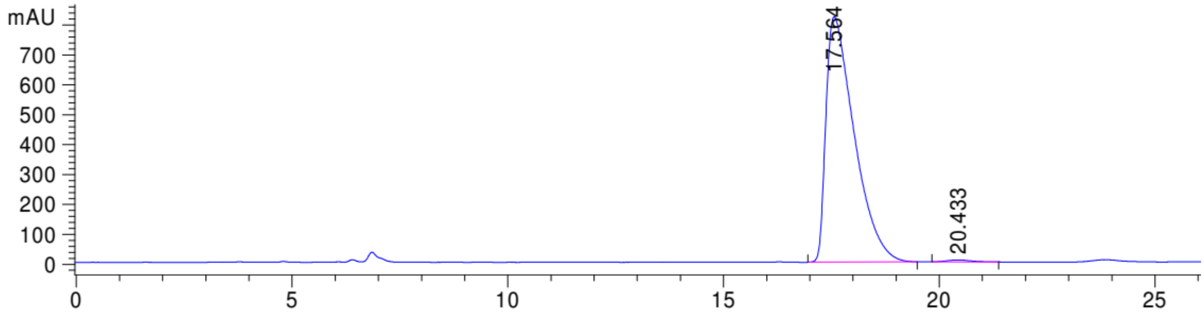

Peak No.	Retention Time / min	Peak Area	Peak Area / %
1	17.564	37458	99.3
2	20.433	265	0.7

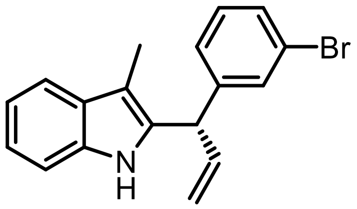

3h

CSP-HPLC of **3h** using Chiralpak OD column, eluting with 5% IPA in hexanes.

Racemic **3h** recorded at 254 nm

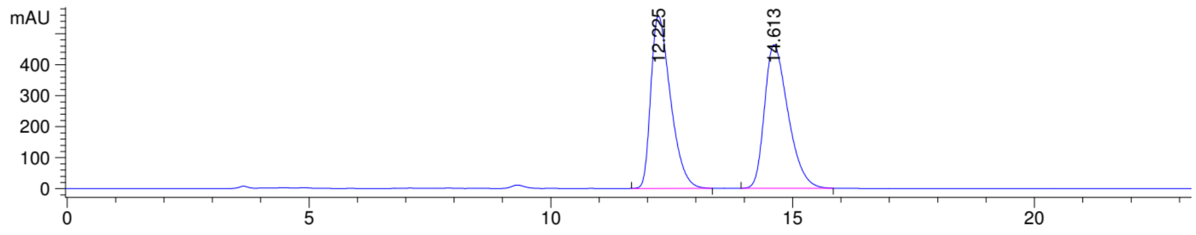

Peak No.	Retention Time / min	Peak Area	Peak Area / %
1	12.225	15735	50.1
2	14.613	15692	49.9

(−)3h recorded at 254 nm

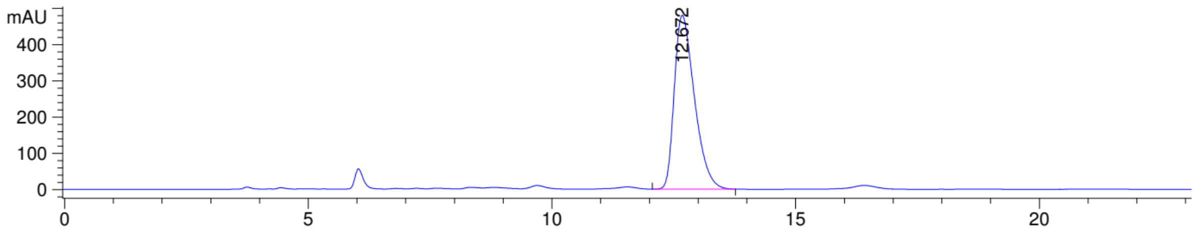

Peak No.	Retention Time / min	Peak Area	Peak Area / %
1	12.672	13823	100.0
2	-	-	-

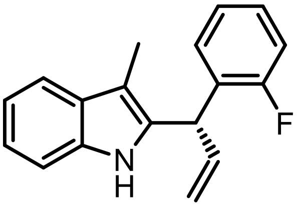

3i

CSP-HPLC of **3i** using Chiralpak AD-H column, eluting with 3% IPA in hexanes.

Racemic **3i** recorded at 280 nm

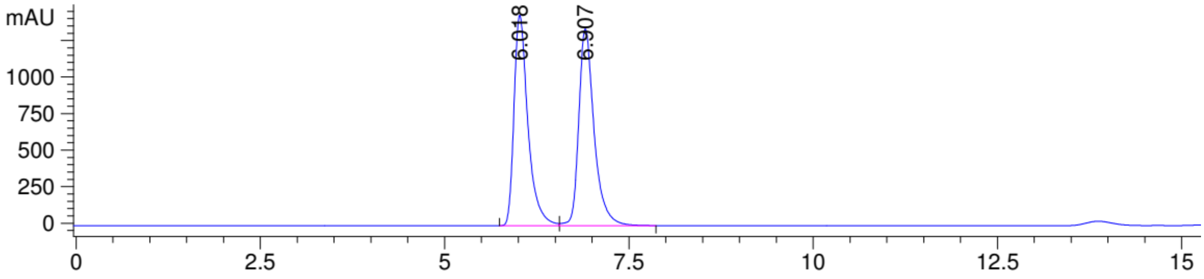

Peak No.	Retention Time / min	Peak Area	Peak Area / %
1	6.018	19011	49.3
2	6.907	19549	50.7

(+)3i recorded at 280 nm

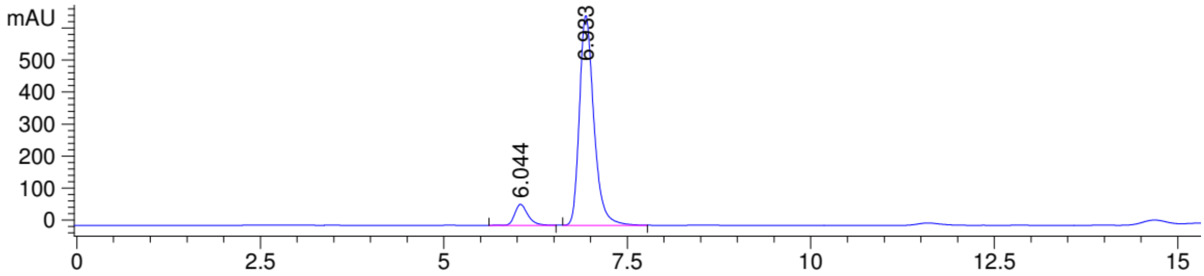

Peak No.	Retention Time / min	Peak Area	Peak Area / %
1	6.044	830	8.4
2	6.933	9008	91.6

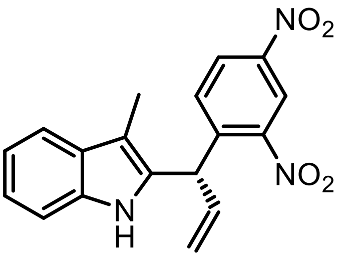

3j

CSP-HPLC of **3j** using Chiralpak IB column, eluting with 10% IPA in hexanes.

Racemic **3j** recorded at 254 nm

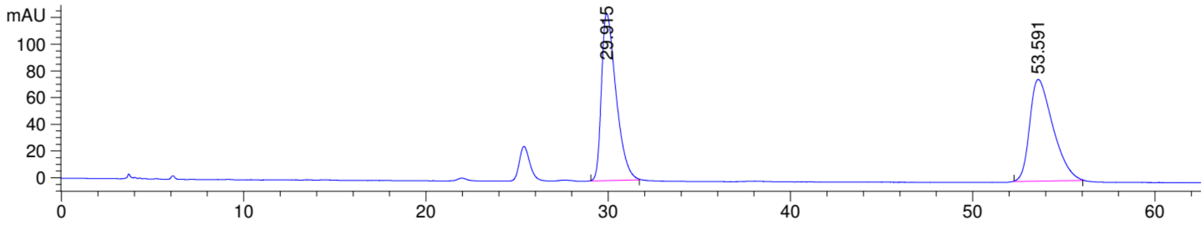

Peak No.	Retention Time / min	Peak Area	Peak Area / %
1	29.915	6943	50.6
2	53.591	6781	49.4

(+)3j recorded at 254 nm

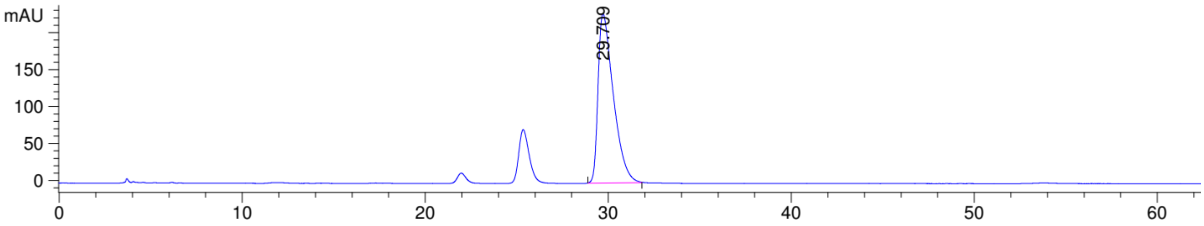

Peak No.	Retention Time / min	Peak Area	Peak Area / %
1	29.709	13259	100.0
2	-	-	-

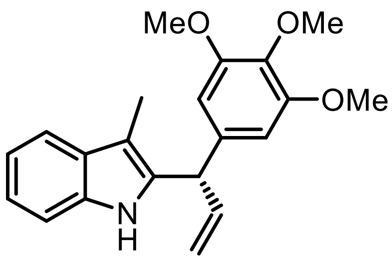

3k

CSP-HPLC of **3k** using Chiralpak OD column, eluting with 10% IPA in hexanes.

Racemic **3k** recorded at 280 nm

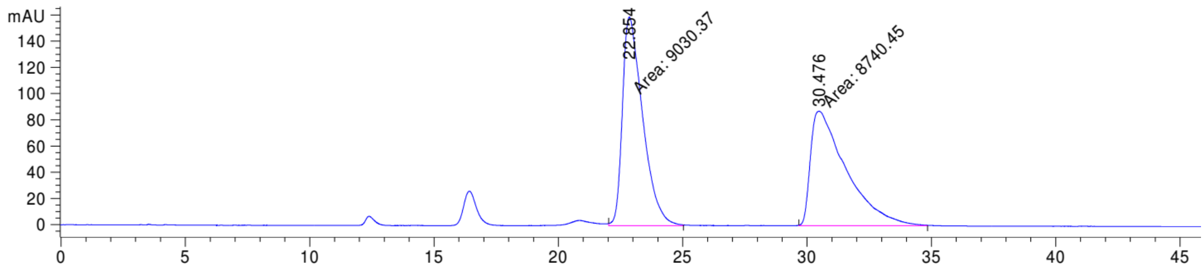

Peak No.	Retention Time / min	Peak Area	Peak Area / %
1	22.854	9030	50.8
2	30.476	8740	49.2

(−)3k recorded at 280 nm

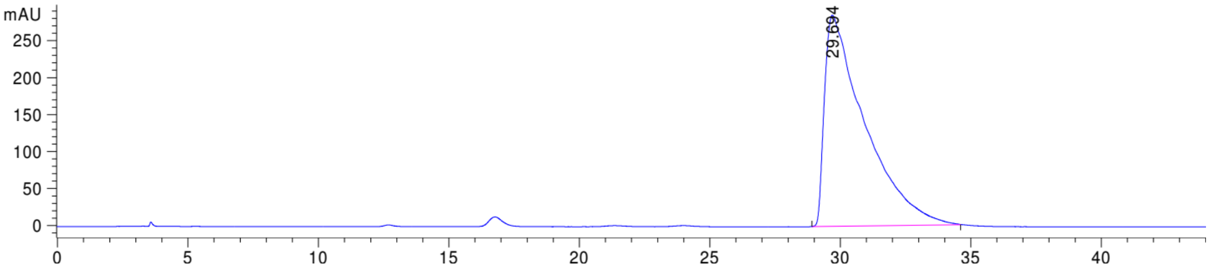

Peak No.	Retention Time / min	Peak Area	Peak Area / %
1	29.694	30735	100.0
2	-	-	-

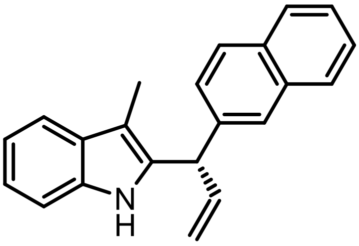

3l

CSP-HPLC of **3l** using Chiralpak AD-H column, eluting with 3% IPA in hexanes.

Racemic **3l** recorded at 254 nm

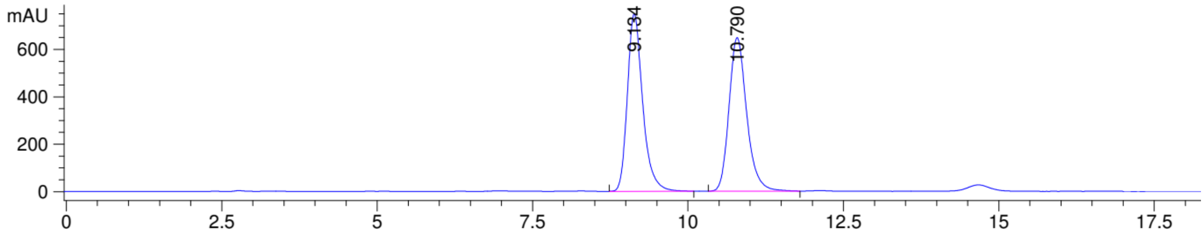

Peak No.	Retention Time / min	Peak Area	Peak Area / %
1	9.134	12863	50.0
2	10.790	12845	50.0

(−)3l recorded at 254 nm

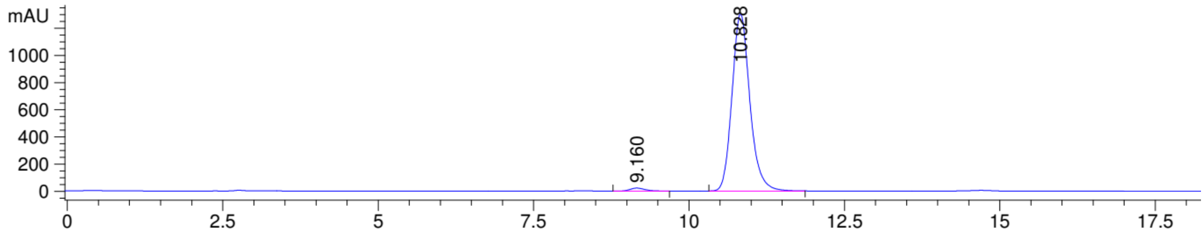

Peak No.	Retention Time / min	Peak Area	Peak Area / %
1	9.160	381	1.4
2	10.828	26033	98.6

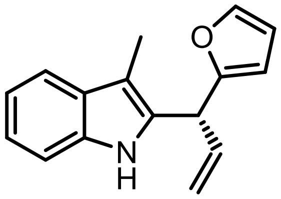

3m

CSP-HPLC of **3m** using Chiralpak OD column, eluting with 1% IPA in hexanes (0.4 mL/min flow rate).

Racemic **3m** recorded at 254 nm

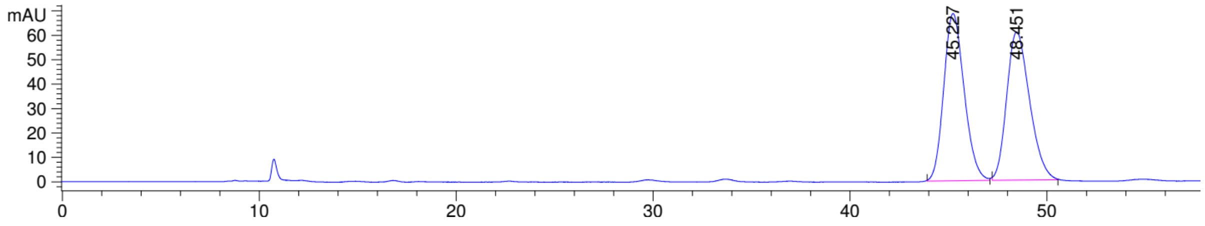

Peak No.	Retention Time / min	Peak Area	Peak Area / %
1	45.227	4949	50.2
2	48.451	4904	49.8

(−)3m recorded at 254 nm

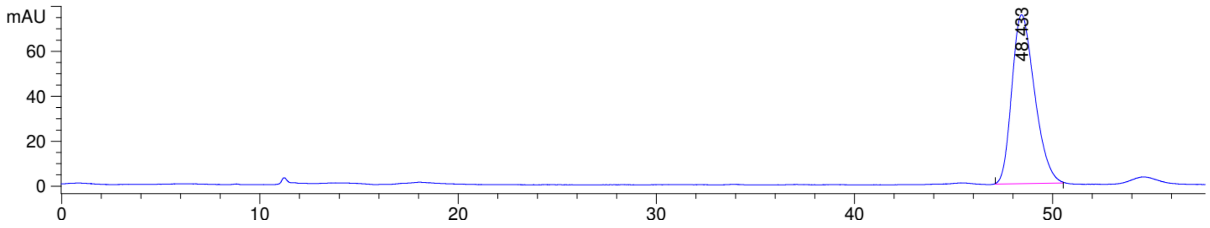

Peak No.	Retention Time / min	Peak Area	Peak Area / %
1	48.433	5995	100.0
2	-	-	-

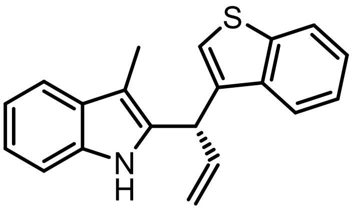

3n

CSP-HPLC of **3n** using Chiralpak AD-H column, eluting with 5% IPA in hexanes.

Racemic **3n** recorded at 254 nm

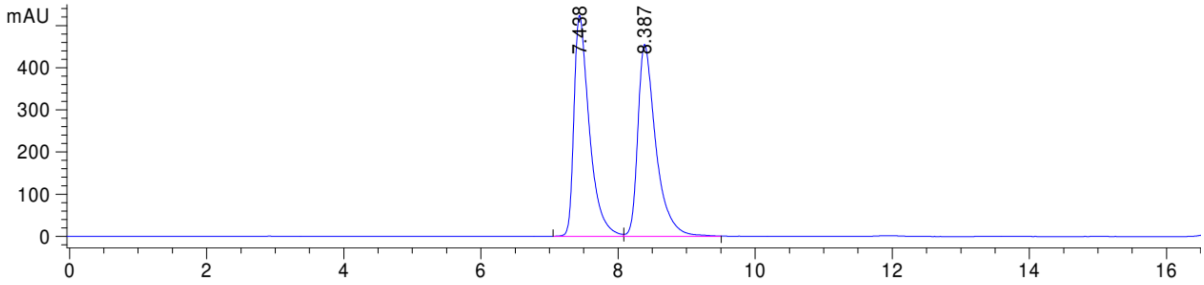

Peak No.	Retention Time / min	Peak Area	Peak Area / %
1	7.438	8184	49.6
2	8.387	8306	50.4

(−)3n recorded at 254 nm

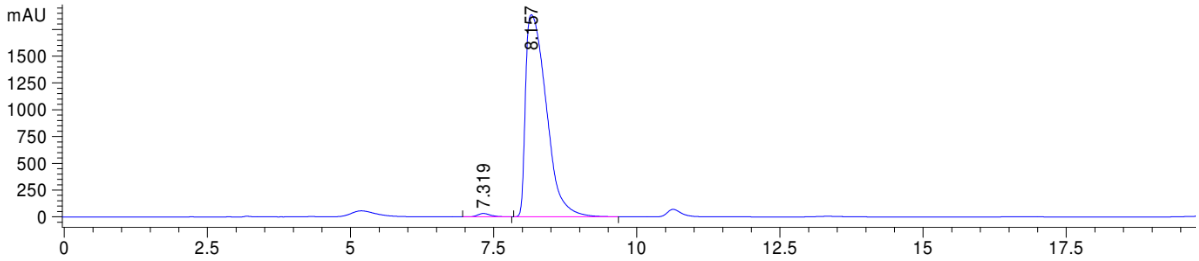

Peak No.	Retention Time / min	Peak Area	Peak Area / %
1	7.319	501	1.0
2	8.157	47499	99.0

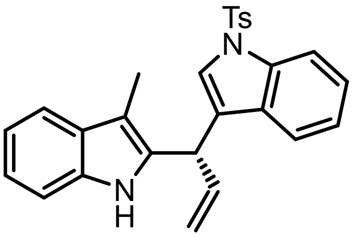

3o

CSP-HPLC of **3o** using Chiralpak IB column, eluting with 10% IPA in hexanes.

Racemic **3o** recorded at 280 nm

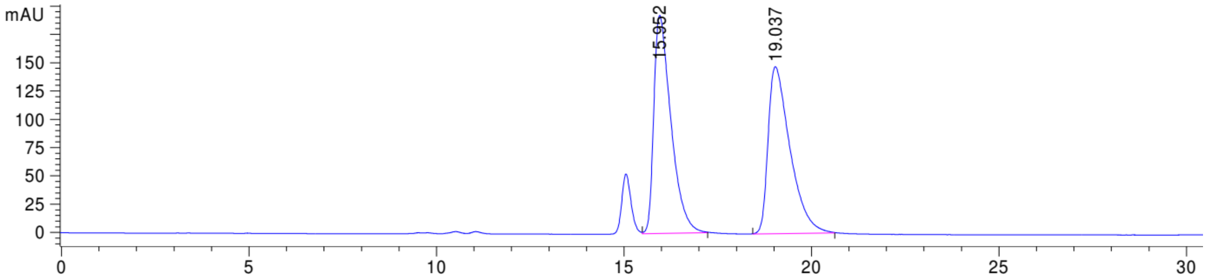

Peak No.	Retention Time / min	Peak Area	Peak Area / %
1	15.952	6112	50.5
2	19.037	6002	49.5

(−)3o recorded at 280 nm

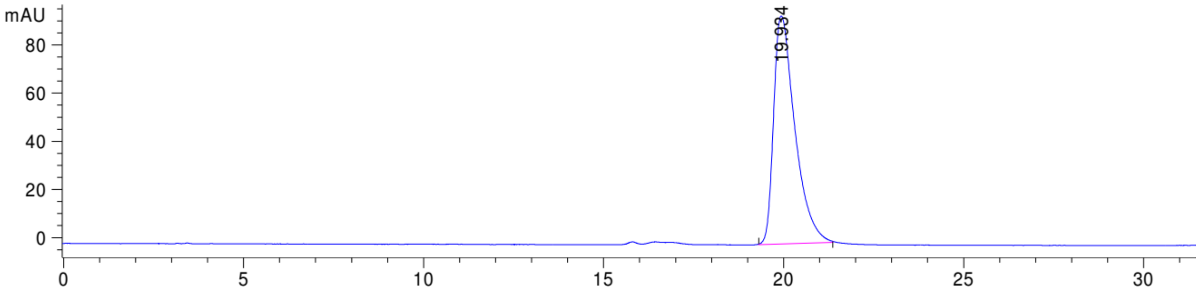

Peak No.	Retention Time / min	Peak Area	Peak Area / %
1	19.934	3891	100.0
2	-	-	-

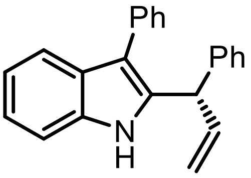

3p

CSP-HPLC of **3p** using Chiralpak AD-H column, eluting with 5% IPA in hexanes.

Racemic **3p** recorded at 280 nm

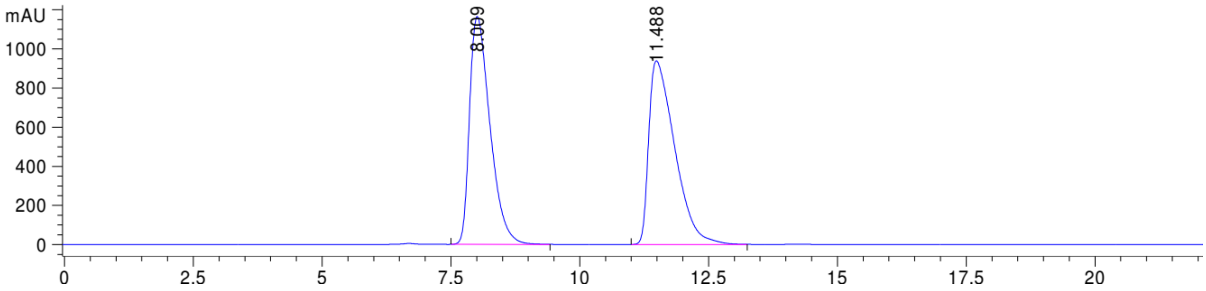

Peak No.	Retention Time / min	Peak Area	Peak Area / %
1	8.009	32148	49.5
2	11.488	32843	50.5

(−)3p recorded at 280 nm

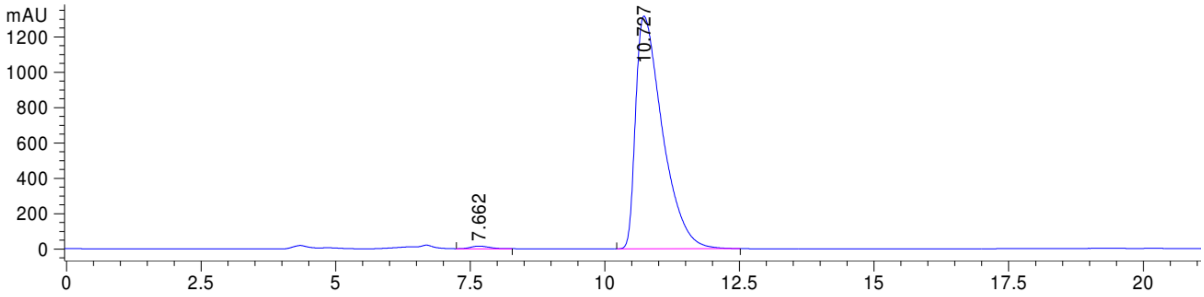

Peak No.	Retention Time / min	Peak Area	Peak Area / %
1	7.662	362	0.8
2	10.727	45585	99.2

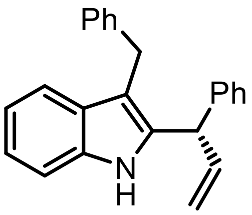

3q

CSP-HPLC of **3q** using Chiralpak AD-H column, eluting with 5% IPA in hexanes.

Racemic **3q** recorded at 254 nm

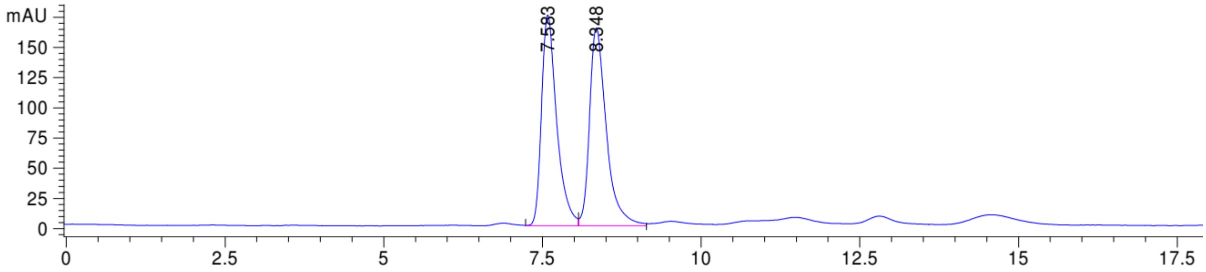

Peak No.	Retention Time / min	Peak Area	Peak Area / %
1	7.583	2904	49.0
2	8.348	3019	51.0

(−)3q recorded at 254 nm

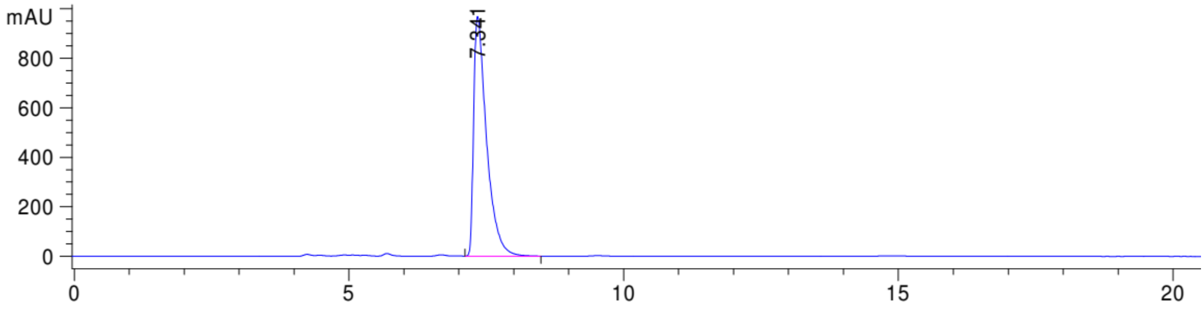

Peak No.	Retention Time / min	Peak Area	Peak Area / %
1	7.341	15836	100.0
2	-	-	-

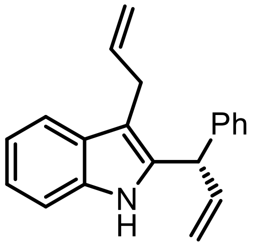

3r

CSP-HPLC of **3r** using Chiralpak IB column, eluting with 5% IPA in hexanes.

Racemic **3r** recorded at 280 nm

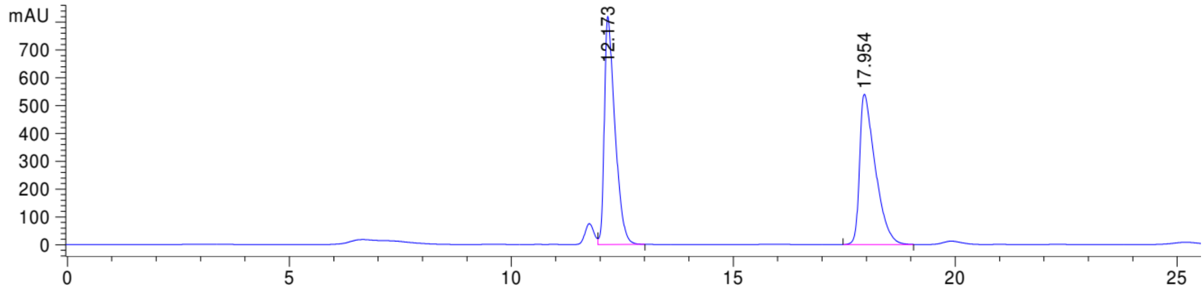

Peak No.	Retention Time / min	Peak Area	Peak Area / %
1	12.173	13599	50.6
2	17.954	13264	49.4

(−)3r recorded at 280 nm

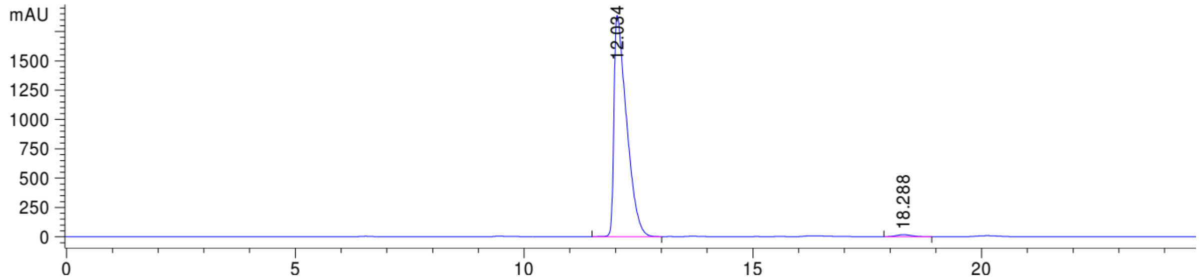

Peak No.	Retention Time / min	Peak Area	Peak Area / %
1	12.034	36035	98.9
2	18.288	403	1.1

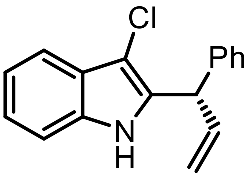

3s

CSP-HPLC of **3s** using Chiralpak OD column, eluting with 5% IPA in hexanes.

Racemic **3s** recorded at 254 nm

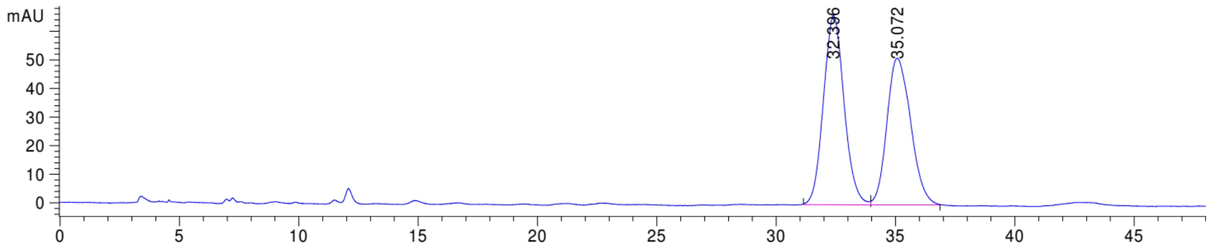

Peak No.	Retention Time / min	Peak Area	Peak Area / %
1	32.396	3989	52.9
2	35.072	3553	47.1

(−)3s recorded at 254 nm

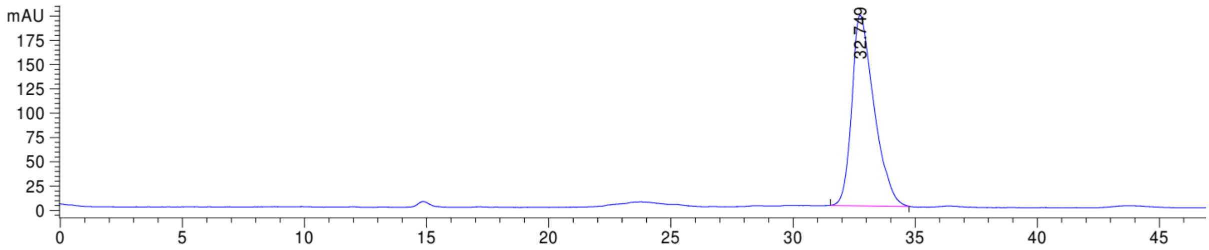

Peak No.	Retention Time / min	Peak Area	Peak Area / %
1	32.749	12478	100.0
2	-	-	-

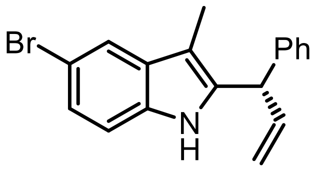

3t

CSP-HPLC of **3t** using Chiralpak IB column, eluting with 5% IPA in hexanes.

Racemic **3t** recorded at 280 nm

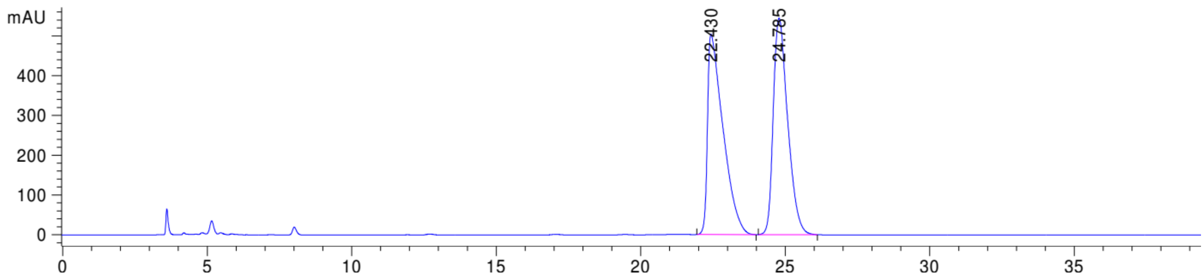

Peak No.	Retention Time / min	Peak Area	Peak Area / %
1	22.430	18831	49.9
2	24.785	18941	50.1

(−)3t recorded at 280 nm

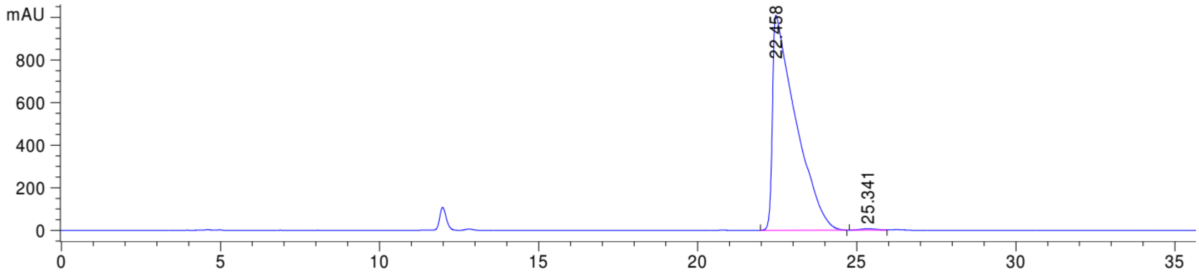

Peak No.	Retention Time / min	Peak Area	Peak Area / %
1	22.458	51253	99.6
2	25.341	208	0.4

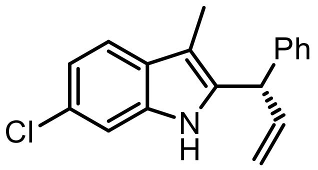

3u

CSP-HPLC of **3u** using Chiralpak OD column, eluting with 5% IPA in hexanes.

Racemic **3u** recorded at 254 nm

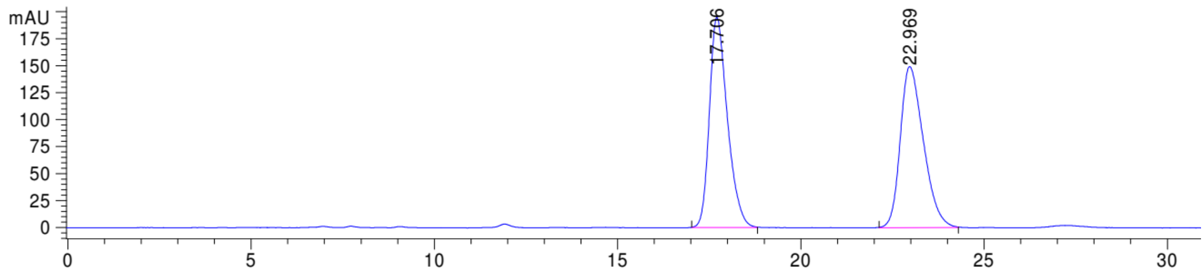

Peak No.	Retention Time / min	Peak Area	Peak Area / %
1	17.706	6651	50.4
2	22.969	6537	49.6

(−)3u recorded at 254 nm

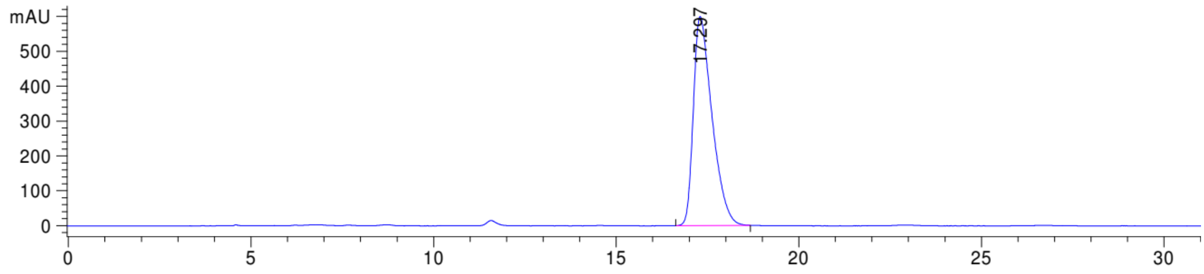

Peak No.	Retention Time / min	Peak Area	Peak Area / %
1	17.297	21330	100.0
2	-	-	-

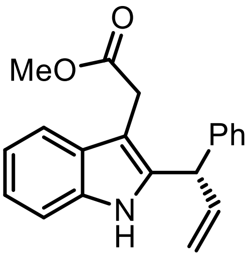

3v

CSP-HPLC of **3v** using Chiralpak OD column, eluting with 10% IPA in hexanes.

Racemic **3v** recorded at 280 nm

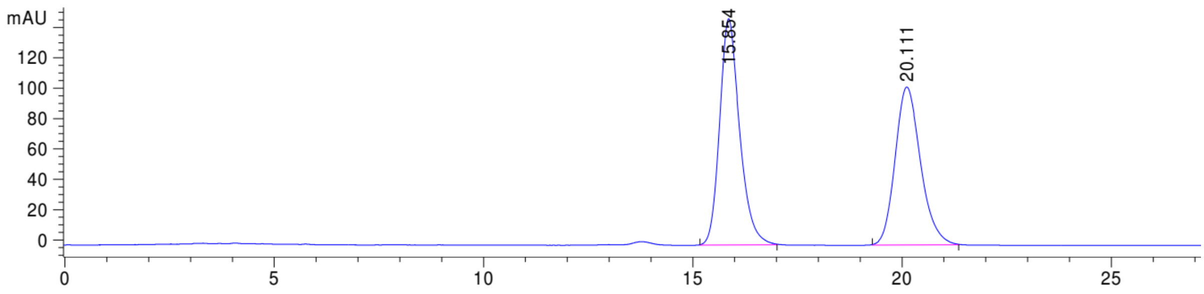

Peak No.	Retention Time / min	Peak Area	Peak Area / %
1	15.854	4938	52.9
2	20.111	4400	47.1

(−)3v recorded at 280 nm

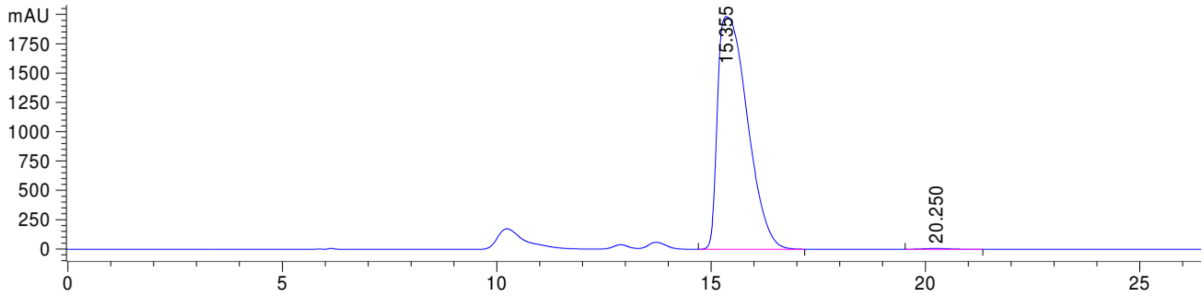

Peak No.	Retention Time / min	Peak Area	Peak Area / %
1	15.355	95698	99.7
2	20.250	326	0.3

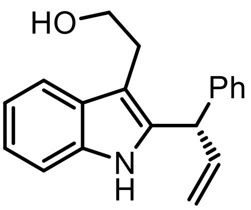

3w

CSP-HPLC of **3v** using Chiralpak AD-H column, eluting with 15% IPA in hexanes.

Racemic **3w** recorded at 280 nm

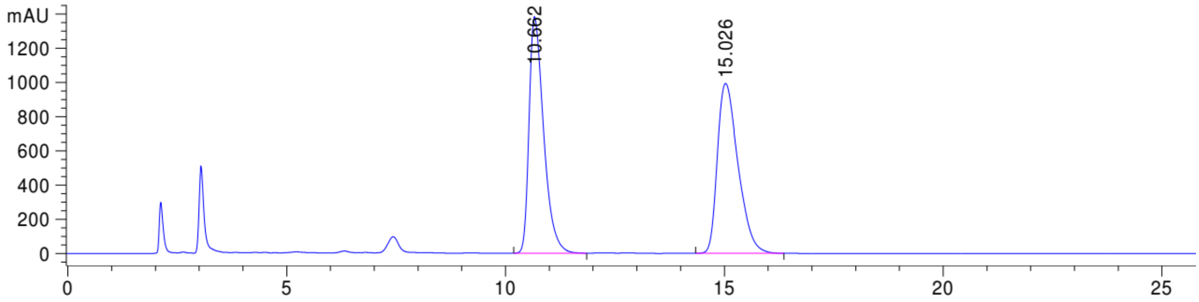

Peak No.	Retention Time / min	Peak Area	Peak Area / %
1	10.662	32291	49.8
2	15.026	32536	50.2

(−)3w recorded at 280 nm

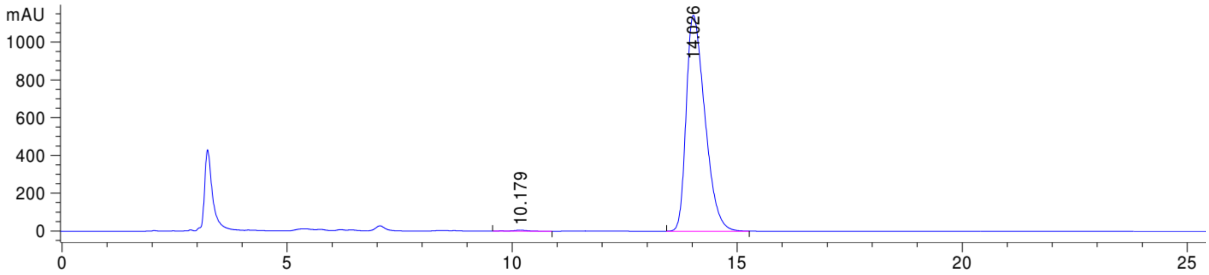

Peak No.	Retention Time / min	Peak Area	Peak Area / %
1	10.179	129	0.4
2	14.026	32973	99.6

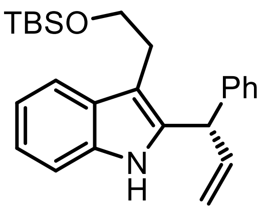

3x

CSP-HPLC of **3x** using Chiralpak OD-H column, eluting with 4% IPA in hexanes.

Racemic **3x** recorded at 280 nm

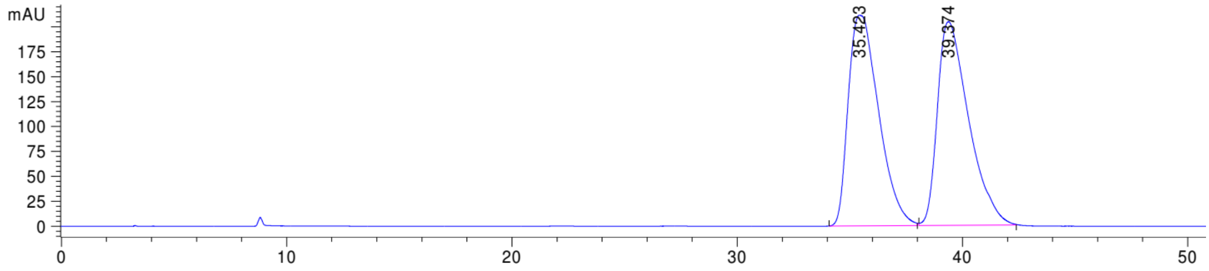

Peak No.	Retention Time / min	Peak Area	Peak Area / %
1	35.423	19797	50.2
2	39.374	19672	49.8

(−)3x recorded at 280 nm

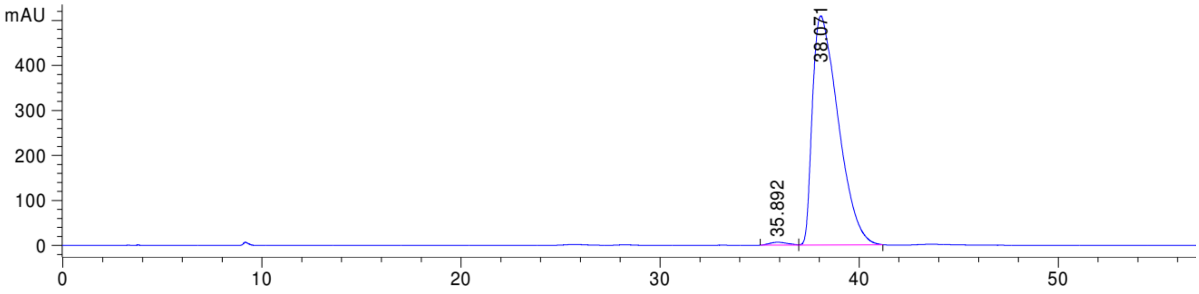

Peak No.	Retention Time / min	Peak Area	Peak Area / %
1	35.892	407	0.9
2	38.071	45837	99.1

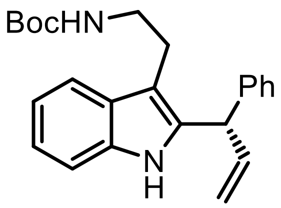

3y

CSP-HPLC of **3y** using Chiralpak AD-H column, eluting with 4% IPA in hexanes.

Racemic **3y** recorded at 280 nm

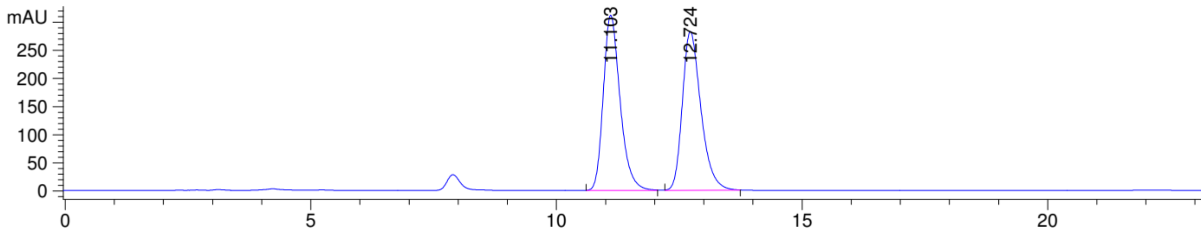

Peak No.	Retention Time / min	Peak Area	Peak Area / %
1	11.103	7406	50.0
2	12.724	7413	50.0

(−)3y recorded at 280 nm

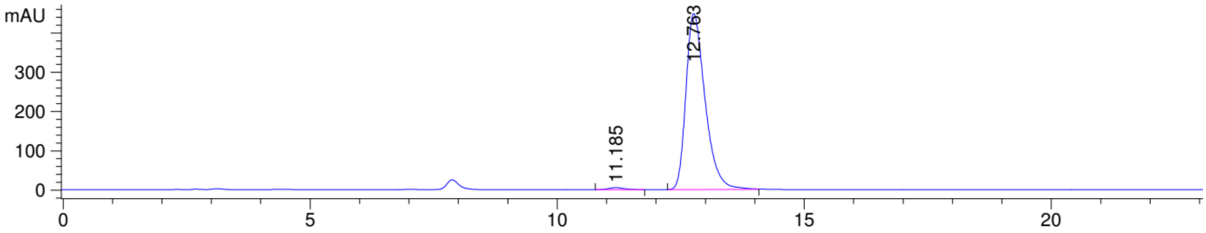

Peak No.	Retention Time / min	Peak Area	Peak Area / %
1	11.185	120	1.0
2	12.763	12078	99.0

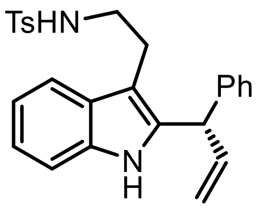

3z

CSP-HPLC of **3z** using Chiralpak AD-H column, eluting with 20% IPA in hexanes.

Racemic **3z** recorded at 280 nm

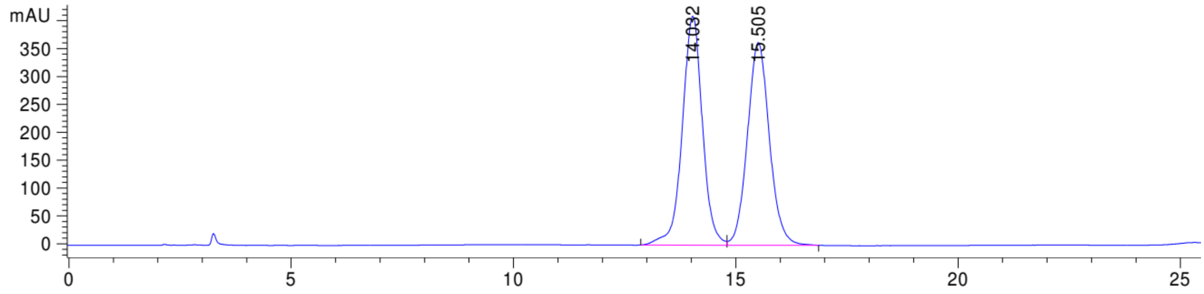

Peak No.	Retention Time / min	Peak Area	Peak Area / %
1	14.032	12980	50.8
2	15.505	12547	49.2

(+)3z recorded at 280 nm

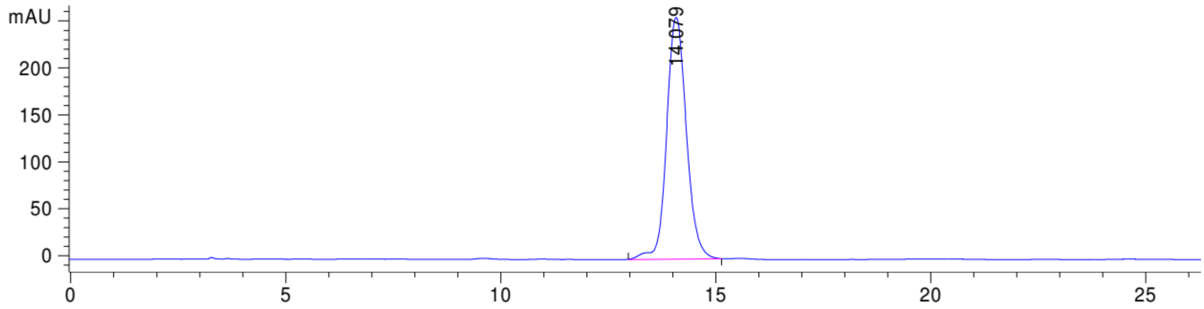

Peak No.	Retention Time / min	Peak Area	Peak Area / %
1	14.079	8231	100.0
2	-	-	-

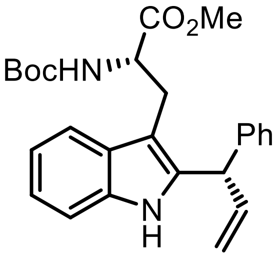

3aa

CSP-HPLC of **3aa** using Chiralpak ID column, eluting with 7% IPA in hexanes.

3aa recorded at 280 nm from enantiopure **2n** using racemic ligand **L2**. **3aa** was formed as a ~1:1 mixture of diastereoisomers with each existing as a ~10:1 mixture of rotamers.

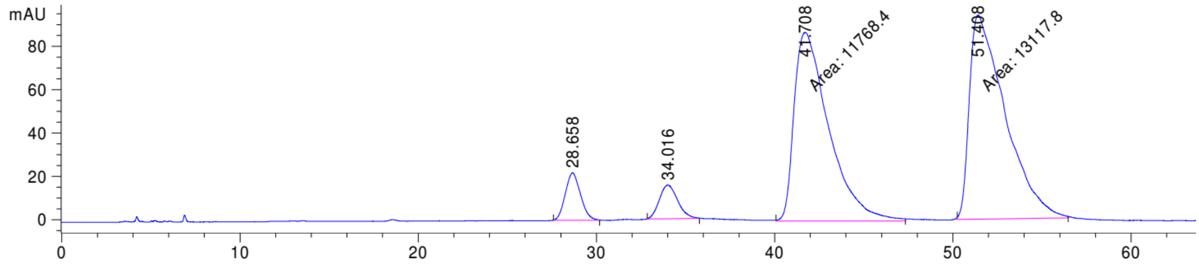

Peak No.	Retention Time / min	Peak Area	Peak Area / %
1	28.658	1291	4.7
2	34.016	1109	4.1
3	41.708	11768	43.1
4	51.408	13118	48.1

(−)3aa recorded at 280 nm (13:1 mixture of rotamers)

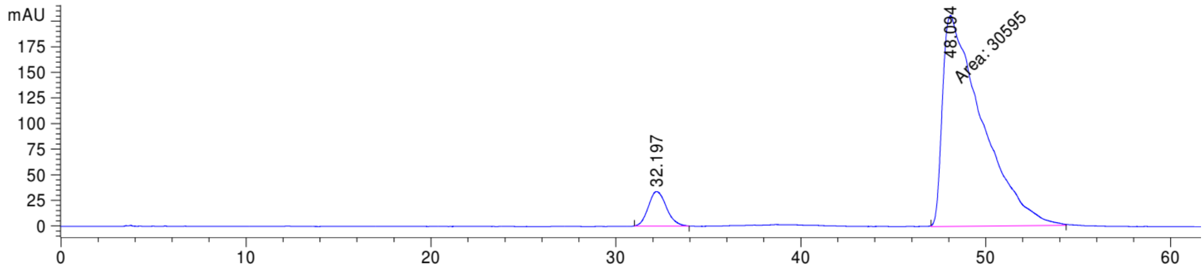

Peak No.	Retention Time / min	Peak Area	Peak Area / %
1	32.197	2303	7.0
2	48.094	30595	93.0
3	-	-	-
4	-	-	-

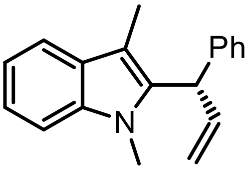

3ab

CSP-HPLC of **3ab** using Chiralpak OD column, eluting with 5% IPA in hexanes.

Racemic **3ab** recorded at 280 nm

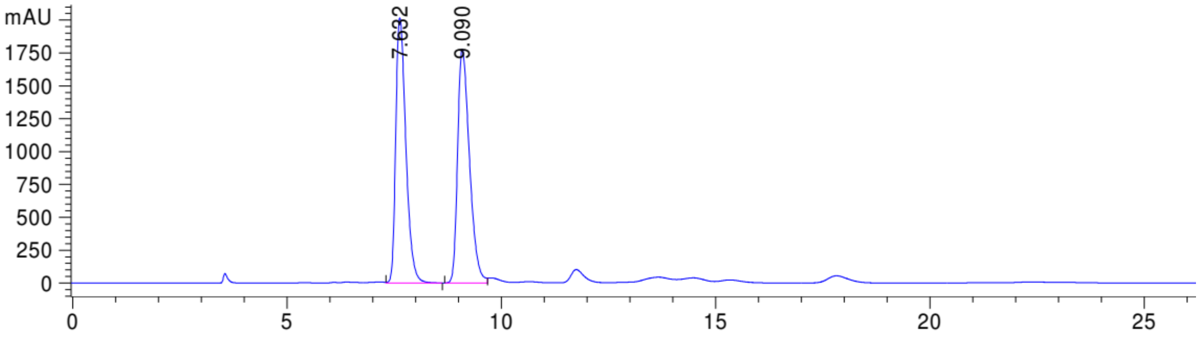

Peak No.	Retention Time / min	Peak Area	Peak Area / %
1	7.632	2017	49.2
2	9.090	1777	50.8

(+)3ab recorded at 280 nm

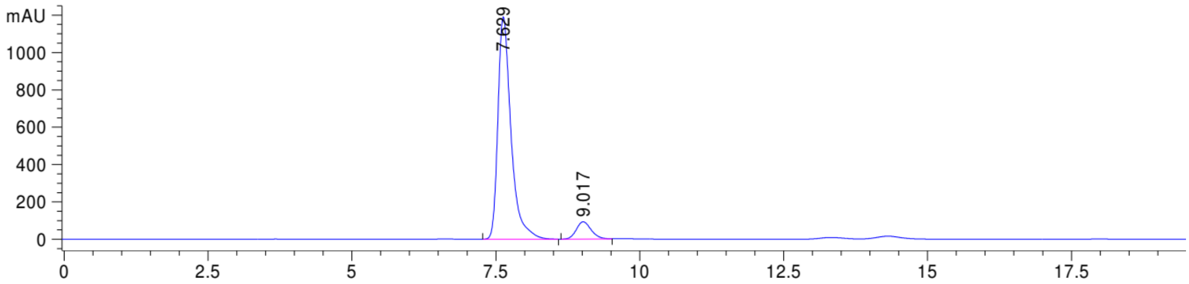

Peak No.	Retention Time / min	Peak Area	Peak Area / %
1	7.629	18853	91.9
2	9.017	1666	8.1

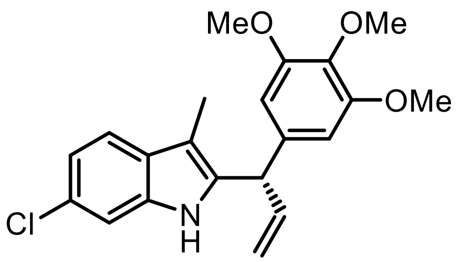

3ac

CSP-HPLC of **3ac** using Chiralpak AD-H column, eluting with 10% IPA in hexanes.

Racemic **3ac** recorded at 254 nm

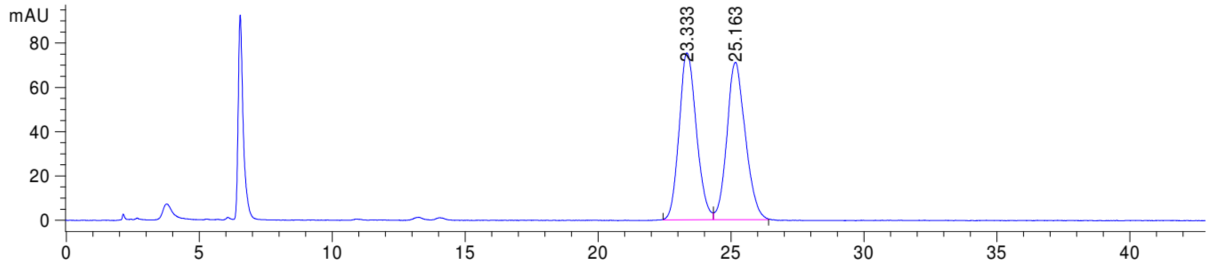

Peak No.	Retention Time / min	Peak Area	Peak Area / %
1	23.333	3416	49.8
2	25.163	3439	50.2

(−)3ac recorded at 254 nm

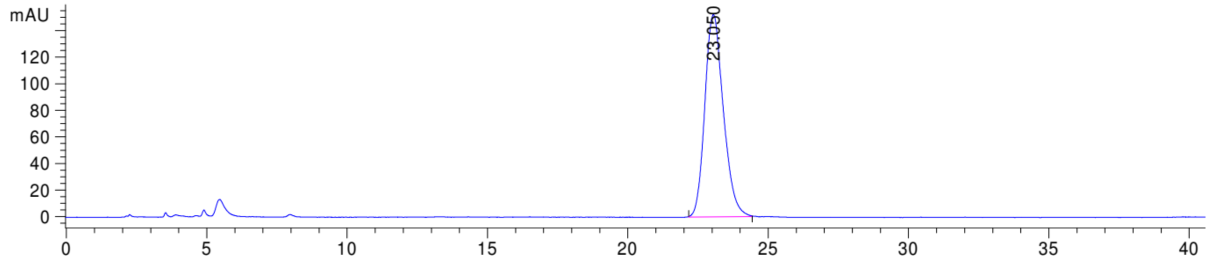

Peak No.	Retention Time / min	Peak Area	Peak Area / %
1	23.050	6974	100.0
2	-	-	-

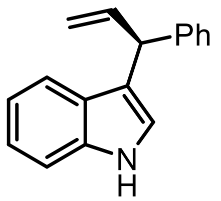

5a

CSP-HPLC of **5a** using Chiralpak IB column, eluting with 2% IPA in hexanes (0.5 mL/min flow rate).

Racemic **5a** recorded at 254 nm

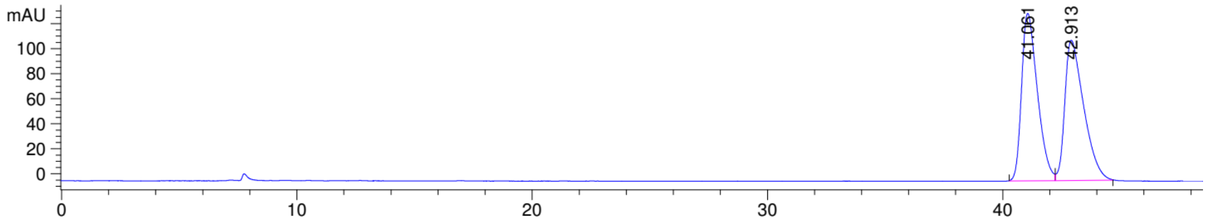

Peak No.	Retention Time / min	Peak Area	Peak Area / %
1	41.061	6267	50.0
2	42.913	6263	50.0

(+)5a recorded at 254 nm

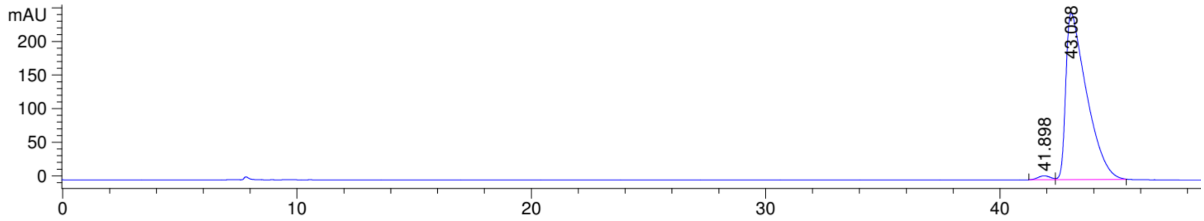

Peak No.	Retention Time / min	Peak Area	Peak Area / %
1	41.898	213	1.4
2	43.038	15261	98.6

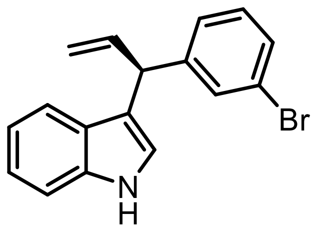

5b

CSP-HPLC of **5b** using Chiralpak IB column, eluting with 2% IPA in hexanes (0.4mL/min flow rate).

Racemic **5b** recorded at 254 nm

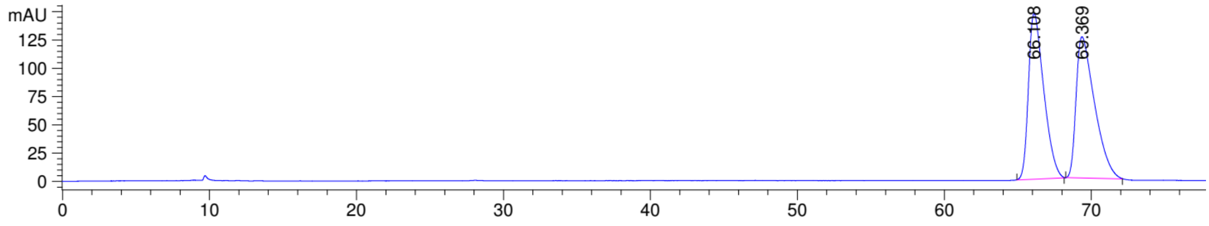

Peak No.	Retention Time / min	Peak Area	Peak Area / %
1	66.108	10608	49.3
2	69.369	10919	50.7

(+)5b recorded at 254 nm

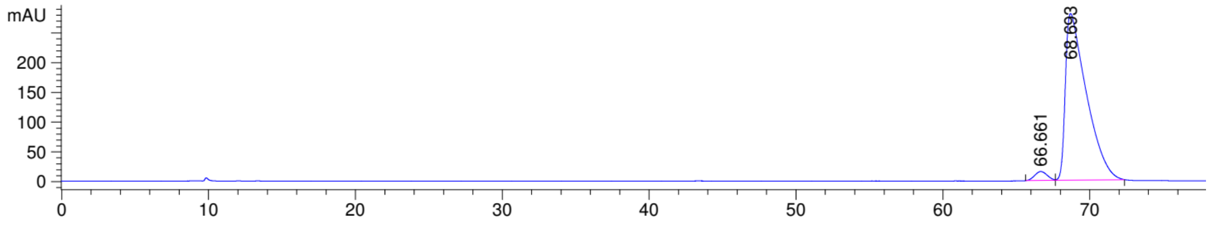

Peak No.	Retention Time / min	Peak Area	Peak Area / %
1	66.661	906	3.1
2	68.693	27886	96.9

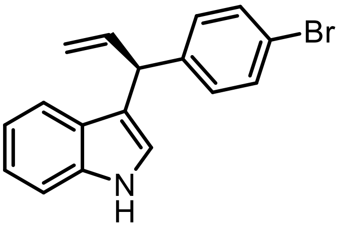

5c

CSP-HPLC of **5c** using Chiralpak AD-H column, eluting with 5% IPA in hexanes.

Racemic **5c** recorded at 254 nm

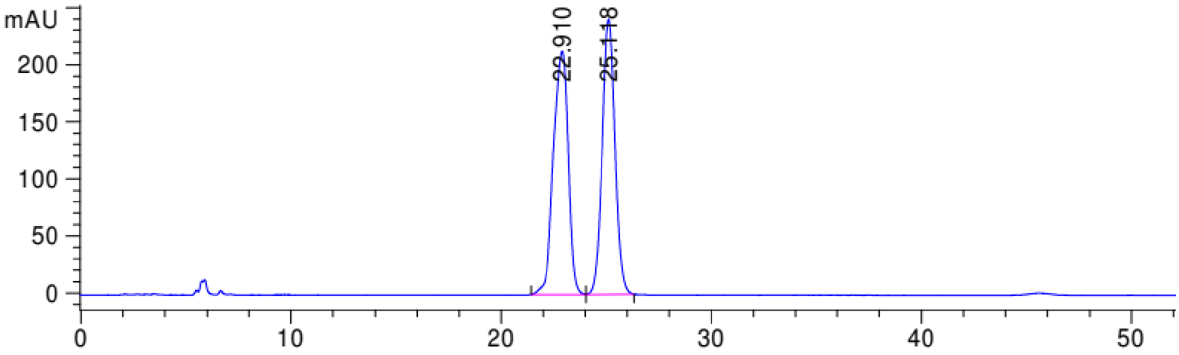

Peak No.	Retention Time / min	Peak Area	Peak Area / %
1	22.910	10451	50.5
2	25.118	10247	49.5

(−)5c recorded at 254 nm

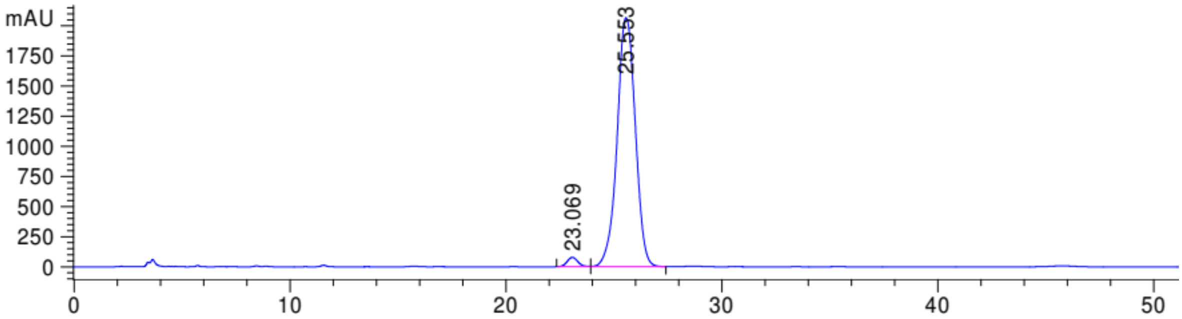

Peak No.	Retention Time / min	Peak Area	Peak Area / %
1	23.069	2728	2.2
2	25.553	121242	97.8

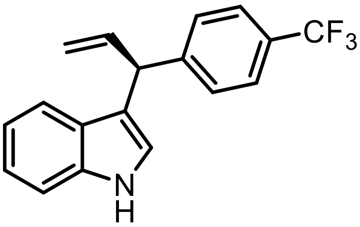

5d

CSP-HPLC of **5d** using Chiralpak IB column, eluting with 2% IPA in hexanes (0.5 mL/min flow rate).

Racemic **5d** recorded at 254 nm

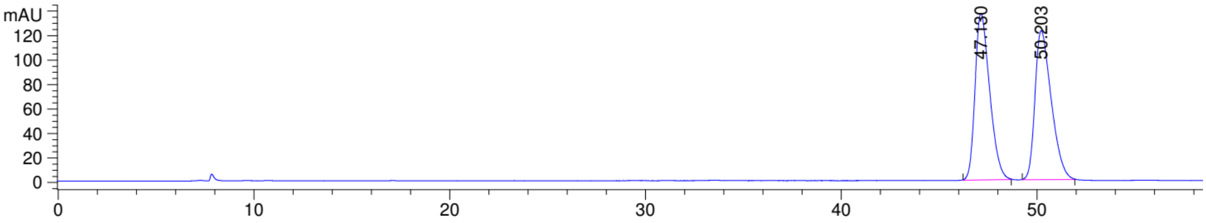

Peak No.	Retention Time / min	Peak Area	Peak Area / %
1	47.130	7064	50.1
2	50.203	7049	49.9

(+)5d recorded at 254 nm

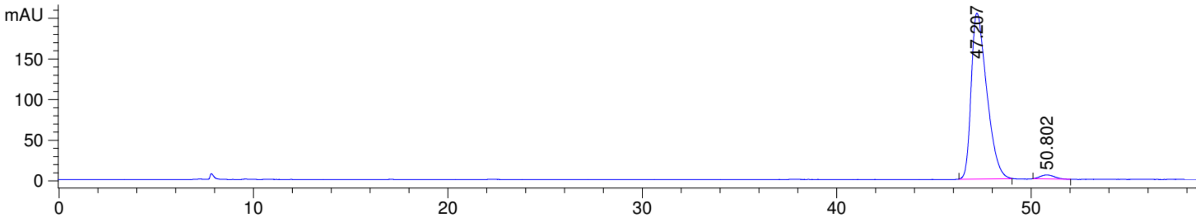

Peak No.	Retention Time / min	Peak Area	Peak Area / %
1	47.207	11331	97.8
2	50.802	256	2.2

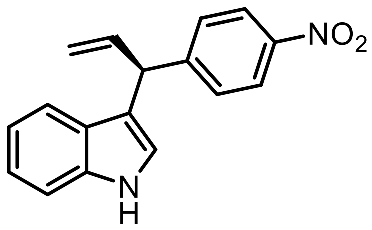

5e

CSP-HPLC of **5e** using Chiralpak AD-H column, eluting with 3% IPA in hexanes.

Racemic **5e** recorded at 280 nm

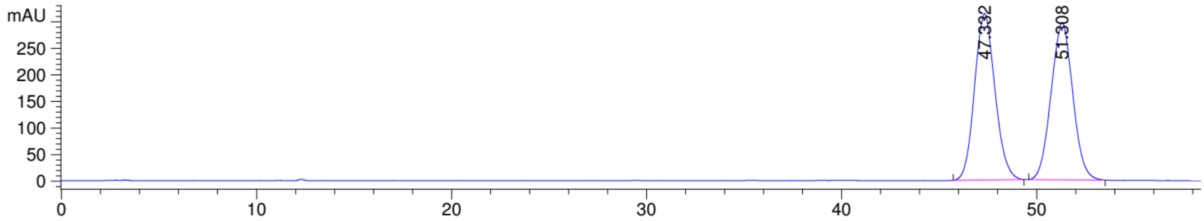

Peak No.	Retention Time / min	Peak Area	Peak Area / %
1	47.332	22900	50.0
2	51.308	22882	50.0

(+)5e recorded at 280 nm

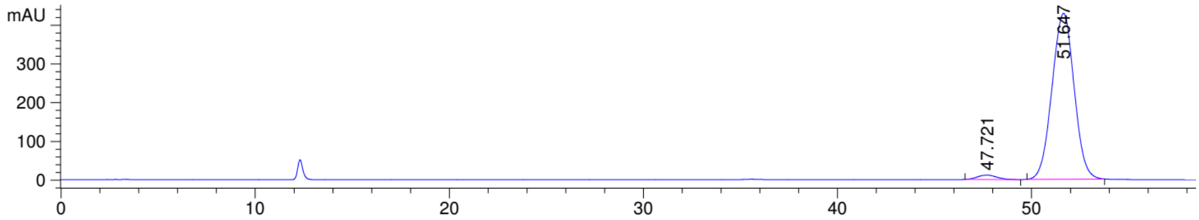

Peak No.	Retention Time / min	Peak Area	Peak Area / %
1	47.721	776	2.2
2	51.647	33885	97.8

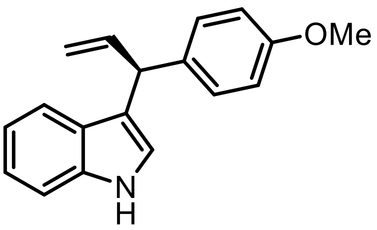

5f

CSP-HPLC of **5f** using Chiralpak OD-H column, eluting with 10% IPA in hexanes.

Racemic **5f** recorded at 254 nm

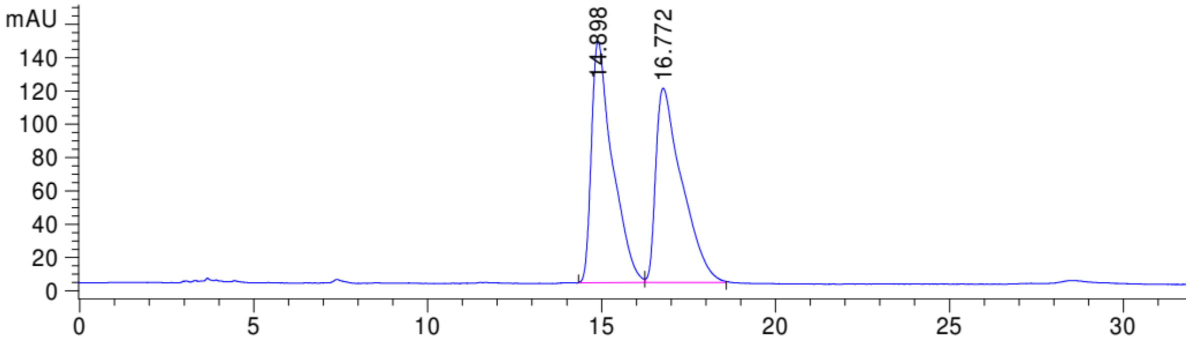

Peak No.	Retention Time / min	Peak Area	Peak Area / %
1	14.898	6083	49.9
2	16.772	6102	50.1

(−)5f recorded at 254 nm

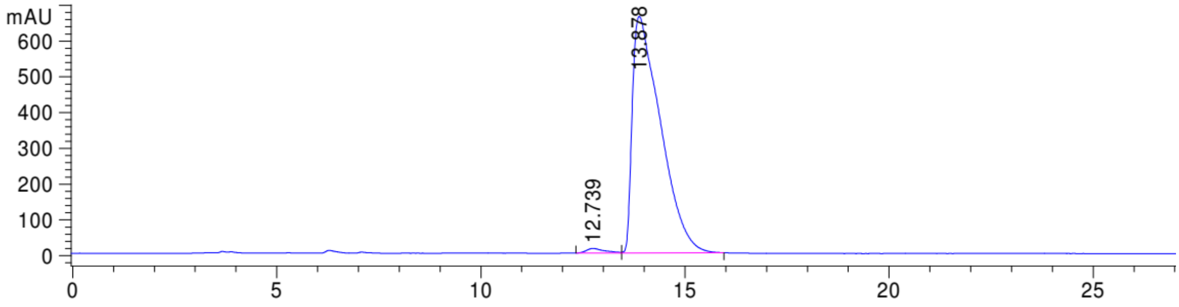

Peak No.	Retention Time / min	Peak Area	Peak Area / %
1	12.739	397	1.2
2	13.878	31814	98.8

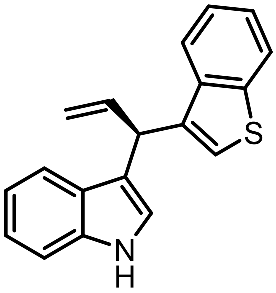

5g

CSP-HPLC of **5g** using Chiralpak AD-H column, eluting with 3% IPA in hexanes.

Racemic **5g** recorded at 280 nm

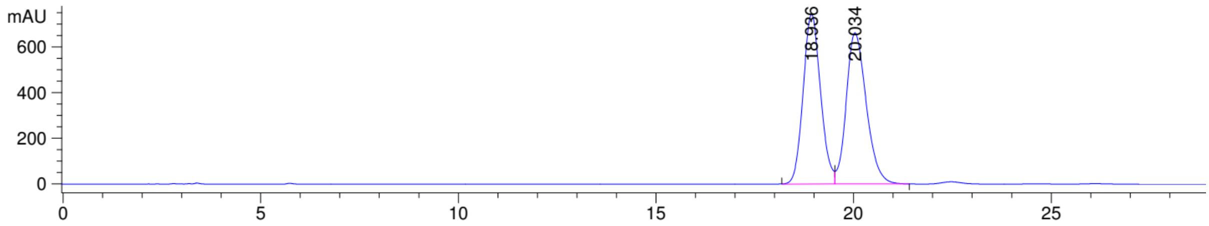

Peak No.	Retention Time / min	Peak Area	Peak Area / %
1	18.936	22860	49.4
2	20.034	23406	50.6

(−)5g recorded at 280 nm

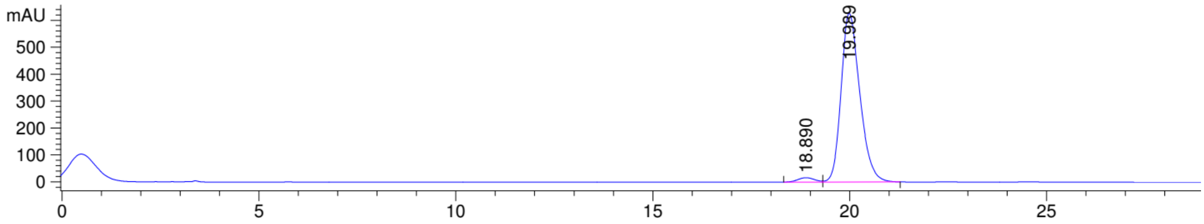

Peak No.	Retention Time / min	Peak Area	Peak Area / %
1	18.890	486	2.3
2	19.989	20711	97.7

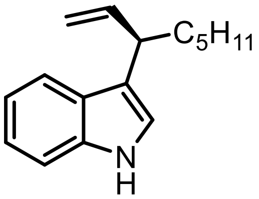

5h

CSP-HPLC of **5h** using Chiralpak OD column, eluting with 2% IPA in hexanes (0.5 mL/min flow rate)

Racemic **5h** recorded at 254 nm

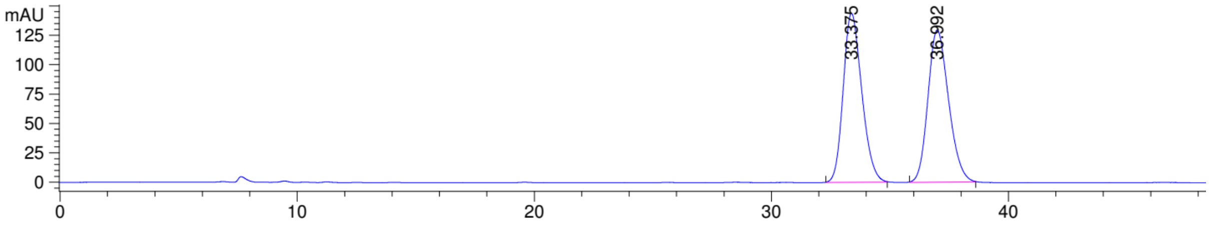

Peak No.	Retention Time / min	Peak Area	Peak Area / %
1	33.375	7831	50.0
2	36.992	7835	50.0

(−)5h recorded at 254 nm

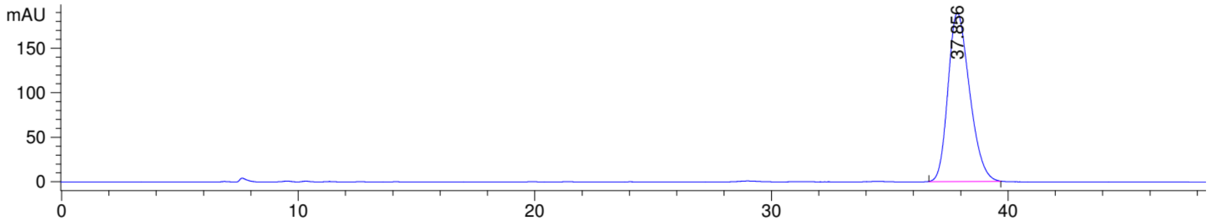

Peak No.	Retention Time / min	Peak Area	Peak Area / %
1	37.856	12030	100.0
2	-	-	-

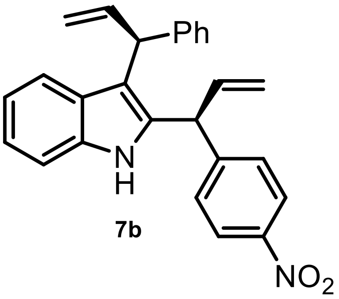

7b

CSP-HPLC of **7b** using Chiralpak OD column, eluting with 10% IPA in hexanes.

7b recorded at 254 nm, made in 2:1 *dr* from compound **5a** (97% *ee*) using racemic ligand **L2**

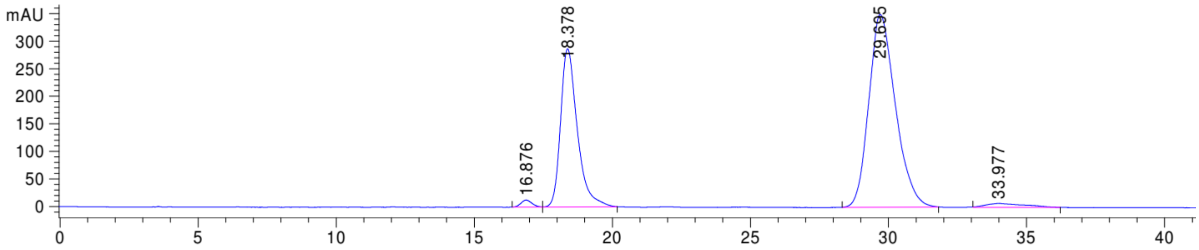

Peak No.	Retention Time / min	Peak Area	Peak Area / %
1	16.876	355	0.97
2	18.378	12097	33.05
3	29.695	23443	64.05
4	33.977	704	1.92

(−)7b recorded at 254 nm

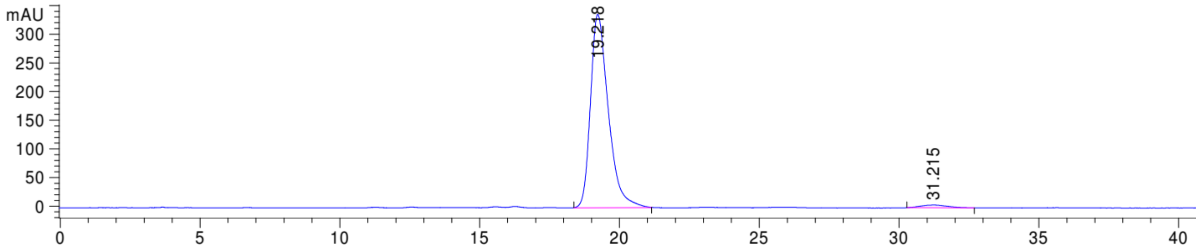

Peak No.	Retention Time / min	Peak Area	Peak Area / %
1	19.218	14956	97.9
2	31.215	316	2.1
3	-	-	-
4	-	-	-

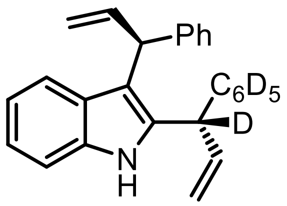

8a

CSP-HPLC of **8a** using Chiralpak OD-H column, eluting with 2% IPA in hexanes.

8a recorded at 280 nm, made in 3:1 *dr* from compound **5a** (97% *ee*) using racemic ligand **L2**

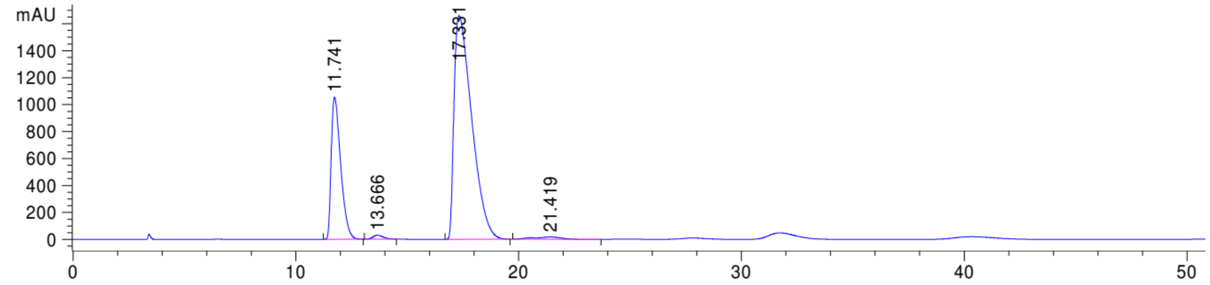

Peak No.	Retention Time / min	Peak Area	Peak Area / %
1	11.741	31124	24.88
2	13.666	965	0.77
3	17.331	91536	73.18
4	21.419	1459	1.17

(−)8a recorded at 280 nm

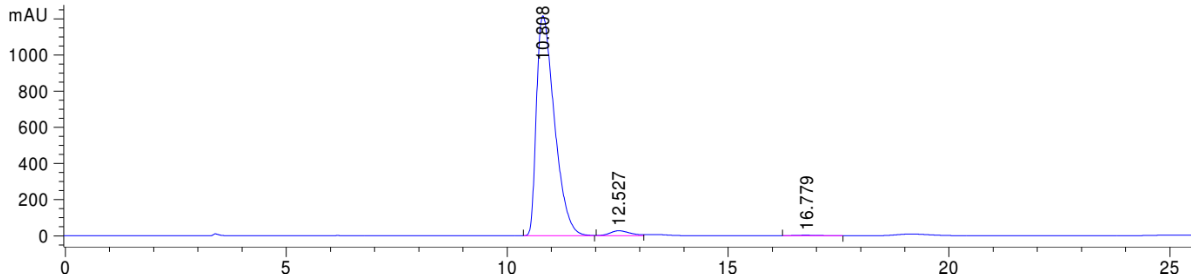

Peak No.	Retention Time / min	Peak Area	Peak Area / %
1	10.808	34345	97.2
2	12.527	881	2.5
3	16.779	103	0.3
4	-	-	-

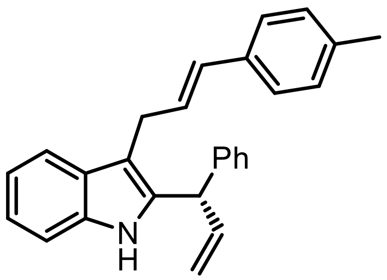

10a

CSP-HPLC of **10a** using Chiralpak OD column, eluting with 8% IPA in hexanes.

Racemic **10a** recorded at 280 nm

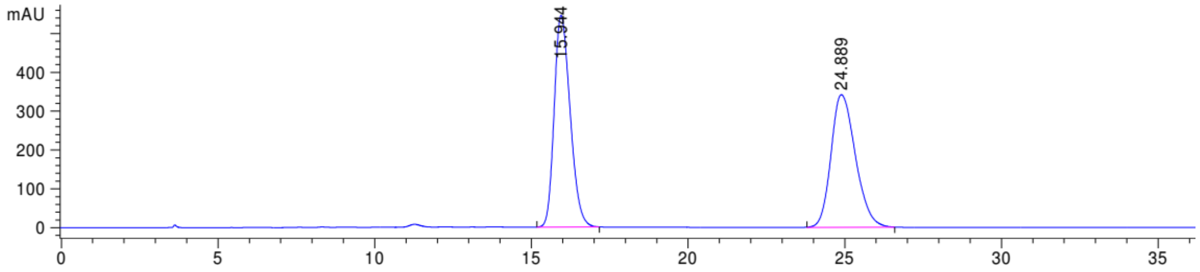

Peak No.	Retention Time / min	Peak Area	Peak Area / %
1	15.944	13919	51.1
2	24.889	18519	48.9

(−)10a recorded at 280 nm

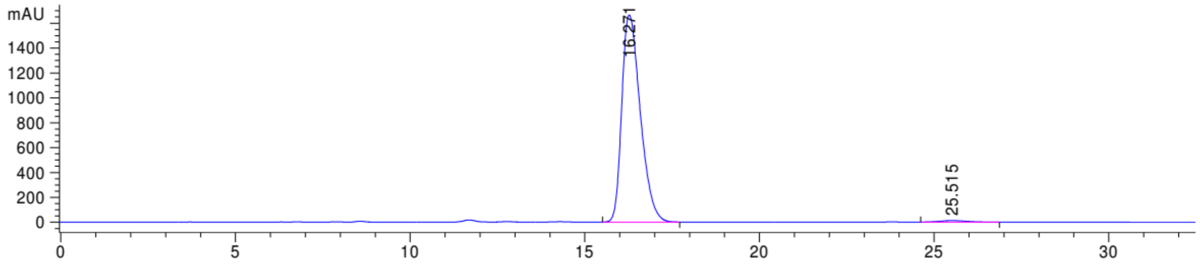

Peak No.	Retention Time / min	Peak Area	Peak Area / %
1	16.271	61286	98.9
2	25.515	670	1.1

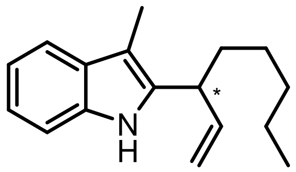

12a

CSP-HPLC of **12a** using Chiralpak OD column, eluting with 5% IPA in hexanes.

Racemic **12a** recorded at 254 nm

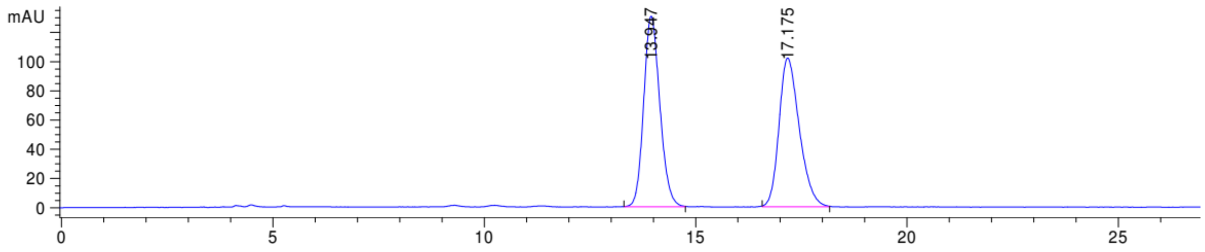

Peak No.	Retention Time / min	Peak Area	Peak Area / %
1	13.947	3466	50.5
2	17.175	3403	49.5

(+)12a recorded at 254 nm

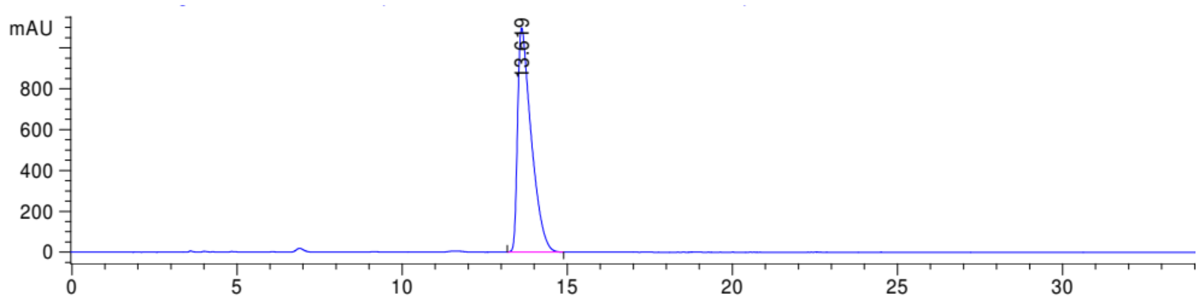

Peak No.	Retention Time / min	Peak Area	Peak Area / %
1	13.619	31510	100.0
2	-	-	-

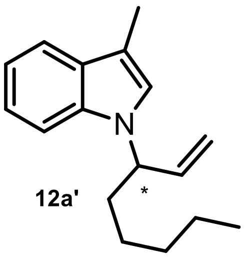

12a'

CSP-HPLC of **12a’** using Chiralpak OD column, eluting with 5% IPA in hexanes.

Racemic **12a’** recorded at 280 nm

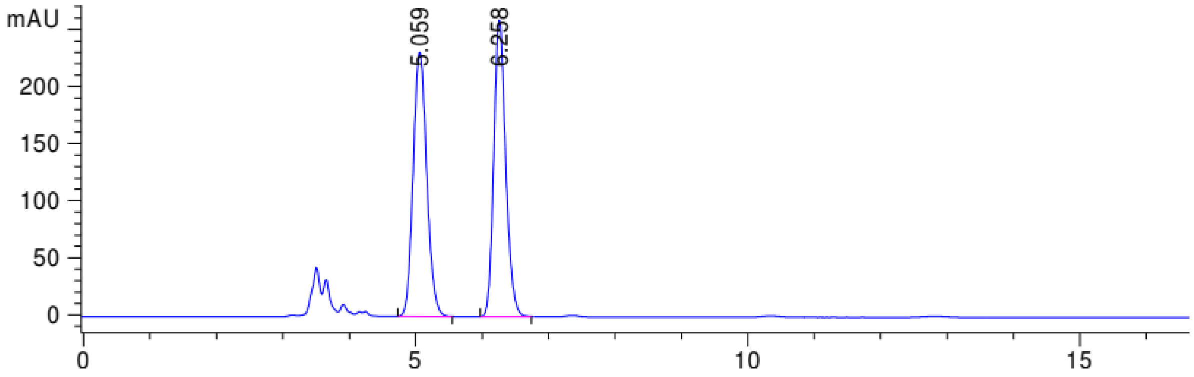

Peak No.	Retention Time / min	Peak Area	Peak Area / %
1	5.059	3281	50.3
2	6.258	3238	49.7

(−)12a’ recorded at 280 nm

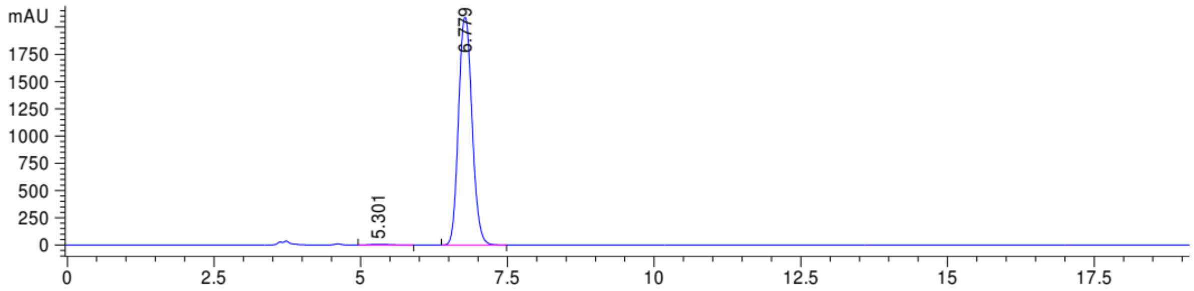

Peak No.	Retention Time / min	Peak Area	Peak Area / %
1	5.301	157	0.5
2	6.779	33596	99.5

X-ray crystal images

CCDC 1942593 and 1942594 contain the supplementary crystallographic data for this paper. This data can be obtained free of charge from The Cambridge Crystallographic Data Centre via www.ccdc.cam.ac.uk/data_request/cif

Compound (*S*)-3k

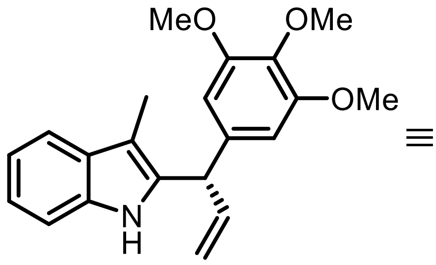

(*S*)-3k

≡

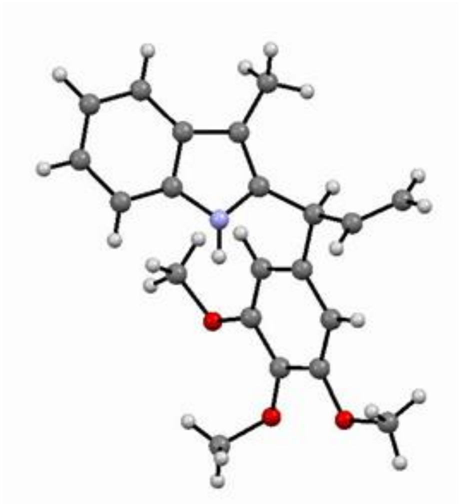

Table S2. Crystal data and structure refinement for 3k.

Identification code	wpu1904
Empirical formula	$C_{21}H_{23}NO_3$
Formula weight	337.40
Temperature/K	110.05(10)
Crystal system	orthorhombic
Space group	$P2_12_12_1$
a/Å	7.73777(12)
b/Å	15.2923(3)
c/Å	15.4203(3)
α/°	90
β/°	90
γ/°	90
Volume/Å^3	1824.66(5)
Z	4
ρ_{calc}g/cm^3	1.228
μ/mm^{-1}	0.655
F(000)	720.0
Crystal size/mm^3	0.326 × 0.086 × 0.046
Radiation	CuKα (λ = 1.54184)
2Θ range for data collection/°	8.142 to 134.09
Index ranges	-9 ≤ h ≤ 9, -18 ≤ k ≤ 18, -18 ≤ l ≤ 18
Reflections collected	13105
Independent reflections	3250 [R_{int} = 0.0253, R_{sigma} = 0.0200]
Data/restraints/parameters	3250/0/319
Goodness-of-fit on F^2	1.064
Final R indexes [I>=2σ (I)]	R_1 = 0.0256, wR_2 = 0.0628
Final R indexes [all data]	R_1 = 0.0275, wR_2 = 0.0642
Largest diff. peak/hole / e Å^{-3}	0.16/-0.13
Flack parameter	-0.06(7)

Compound (*S*)-3ac

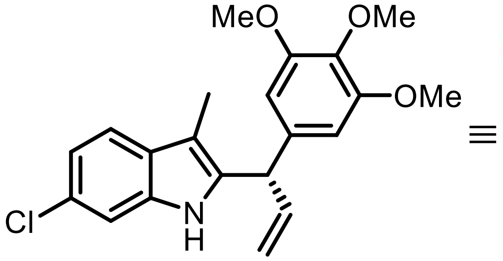

(*S*)-3ac

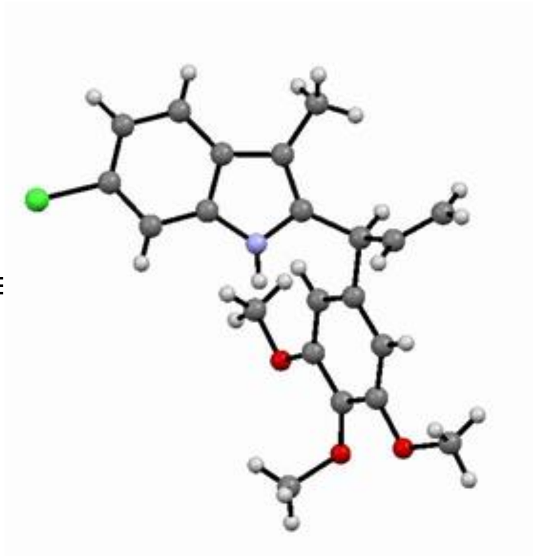

Table S3. Crystal data and structure refinement for 3ac.

Identification code	wpu1905
Empirical formula	$C_{21}H_{22}ClNO_3$
Formula weight	371.84
Temperature/K	110.00(10)
Crystal system	orthorhombic
Space group	$P2_12_12_1$
a/Å	7.41330(10)
b/Å	15.1310(3)
c/Å	17.1744(3)
α/°	90
β/°	90
γ/°	90
Volume/Å^3	1926.46(6)
Z	4
ρ_{calc}g/cm^3	1.282
μ/mm^{-1}	1.916
F(000)	784.0
Crystal size/mm^3	0.128 × 0.074 × 0.042
Radiation	CuKα (λ = 1.54184)
2 Θ range for data collection/°	7.788 to 142.534
Index ranges	-8 ≤ h ≤ 9, -15 ≤ k ≤ 18, -19 ≤ l ≤ 21
Reflections collected	17468
Independent reflections	3689 [R_{int} = 0.0289, R_{sigma} = 0.0213]
Data/restraints/parameters	3689/0/243
Goodness-of-fit on F^2	1.040
Final R indexes [I>=2σ (I)]	R_1 = 0.0261, wR_2 = 0.0684
Final R indexes [all data]	R_1 = 0.0273, wR_2 = 0.0697
Largest diff. peak/hole / e Å^{-3}	0.22/-0.18
Flack parameter	-0.008(5)

NOE spectrum recorded at 500 MHz for compound 7b

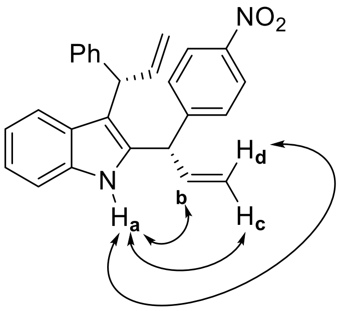

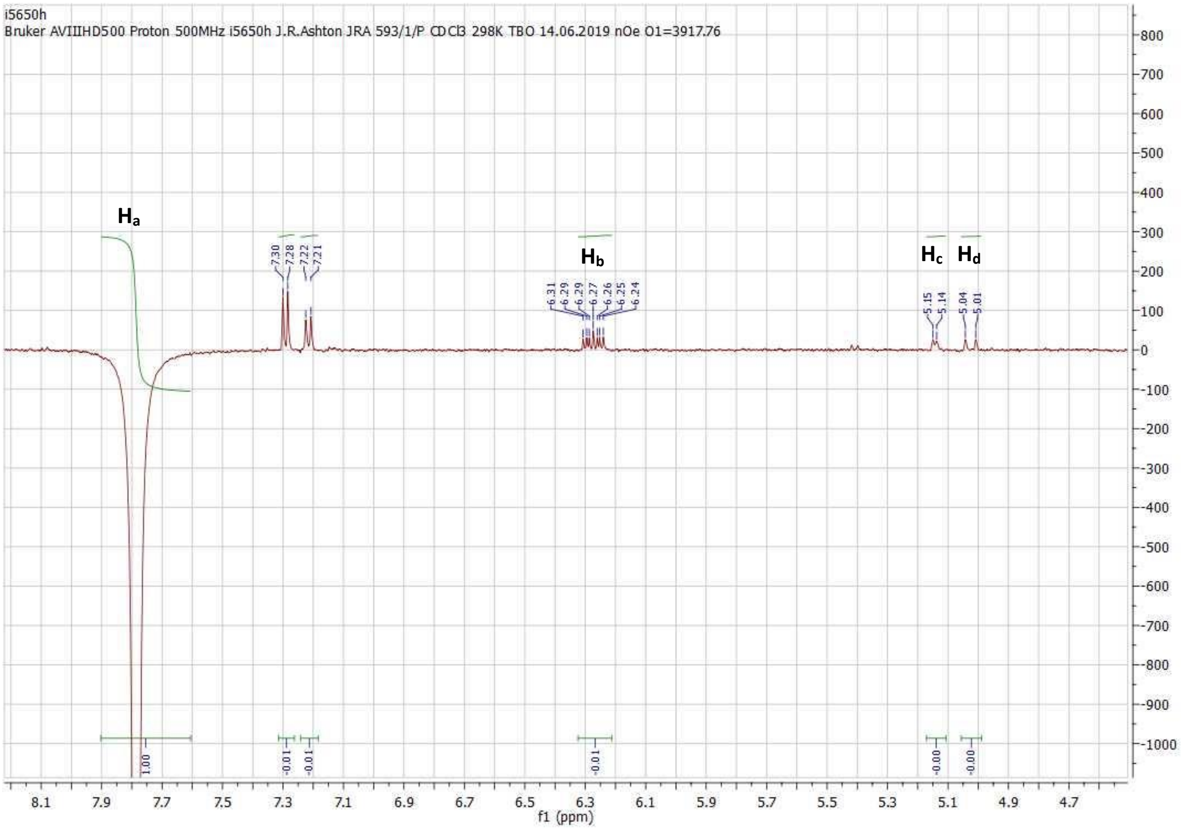

NOE spectrum recorded at 500 MHz for compound 8a

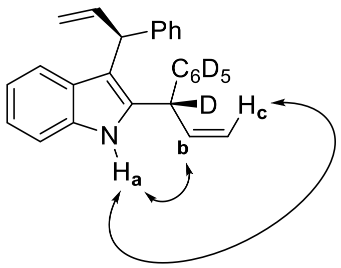

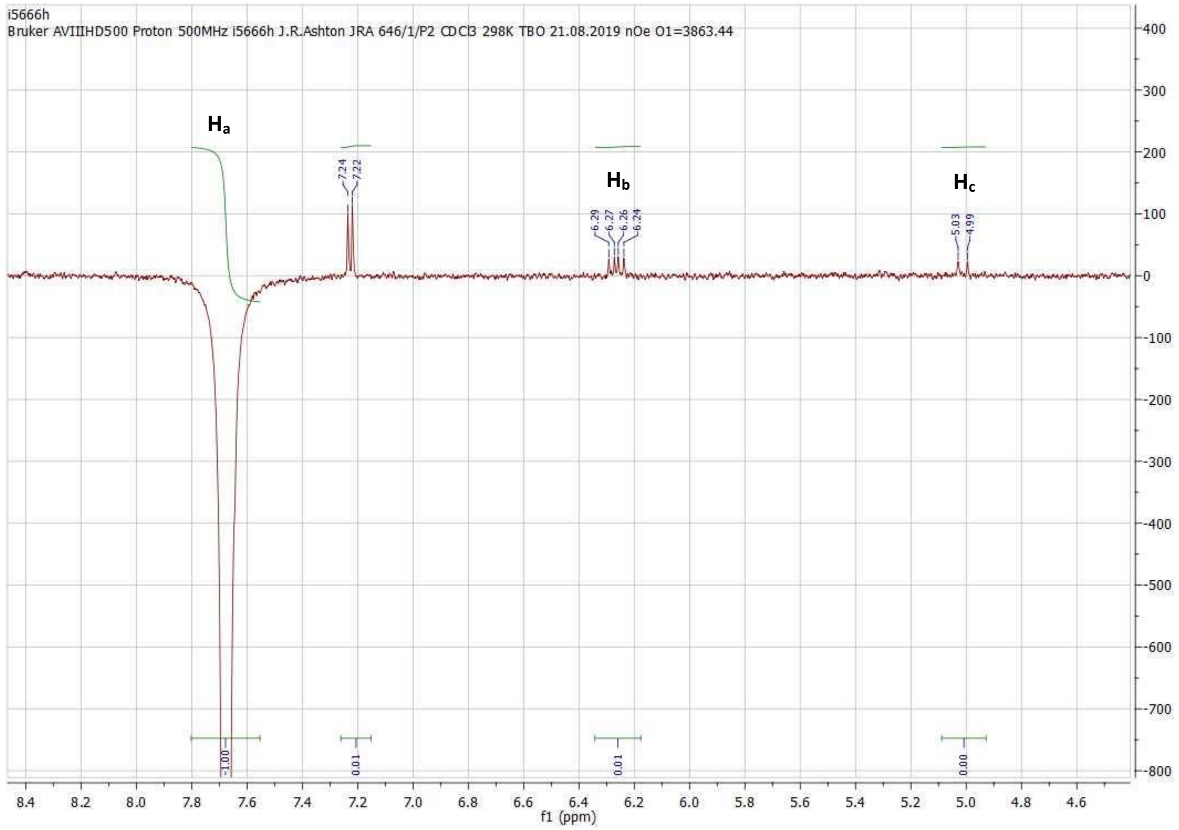

NOE spectrum recorded at 500 MHz for compound 10a

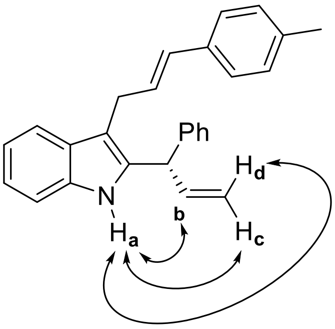

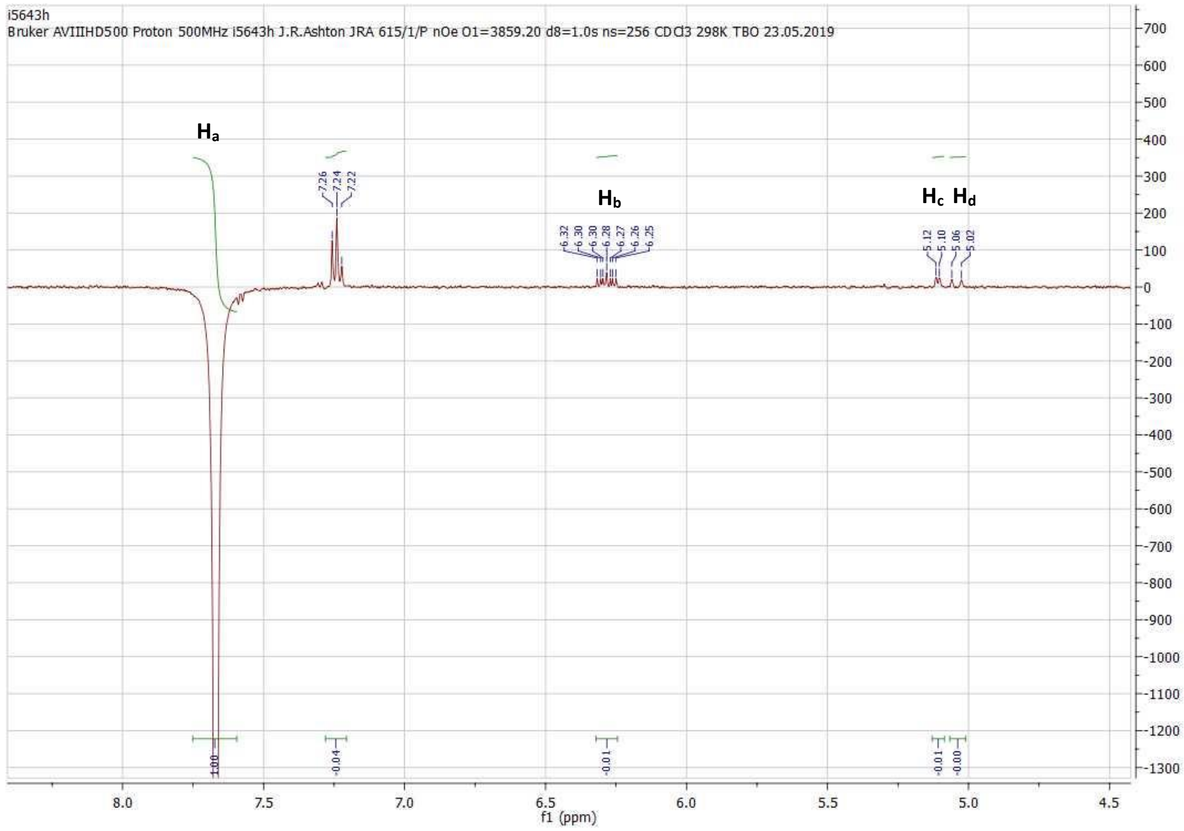

Computational Methods

All the calculations were performed with Gaussian16[19] or ORCA 4.1[20] packages. B3LYP functional[21] with Grimme's empirical dispersion correction (D3-BJ)[22] was employed in the TS optimization. The SDD basis set with the associated effective core potential was used for Ir, and def2-SVP basis set of Weigend and Ahlrichs for other atoms.[23] The energies were further estimated by single-point calculations using RI-B3LYP-D3(BJ)/def2-TZVPP[23,24] in DCM (ε = 8.93) with SMD model.[25] The 3D images of the calculated structures and the orbital diagrams were prepared using CYLView[26] or VMD.[27]

The Origin of Regio- and Enantioselectivity

The origin of enantioselectivity of the Ir-catalyzed asymmetric allylic substitution reactions with the Carreira (P, olefin) ligand has been explored both computationally[28] and experimentally.[29] According to Carreira's mechanistic work,[29] the *exo* π-allyl iridium species was characterized by X-ray crystallographic analysis, while the preferred allylic substitution products were generated from the *endo* ones, which suggested a fast *π-σ-π* flip of the *π*-allyl iridium intermediate was in operation. Herein, when (*S*)-Carreira (P, olefin) ligand was used, the nucleophilic attacks of indole (C2 or C3 position) to *endo π*-cinnamyl iridium species will afford the (*S*)-product, while the corresponding attacks to *exo π*-cinnamyl iridium species will afford the (*R*)-product.

Our calculations confirmed that the attacks to *endo π*-cinnamyl iridium species in the current system are significantly preferred compared with the *exo* ones, which is in agreement with the experimentally observed absolute configuration of the product. The general energy difference between *endo* and *exo* attacks likely stems from the less steric congestion between the cinnamyl moiety (together with the incoming nucleophile) and the chiral ligand during *endo* attacks (Figure S1).

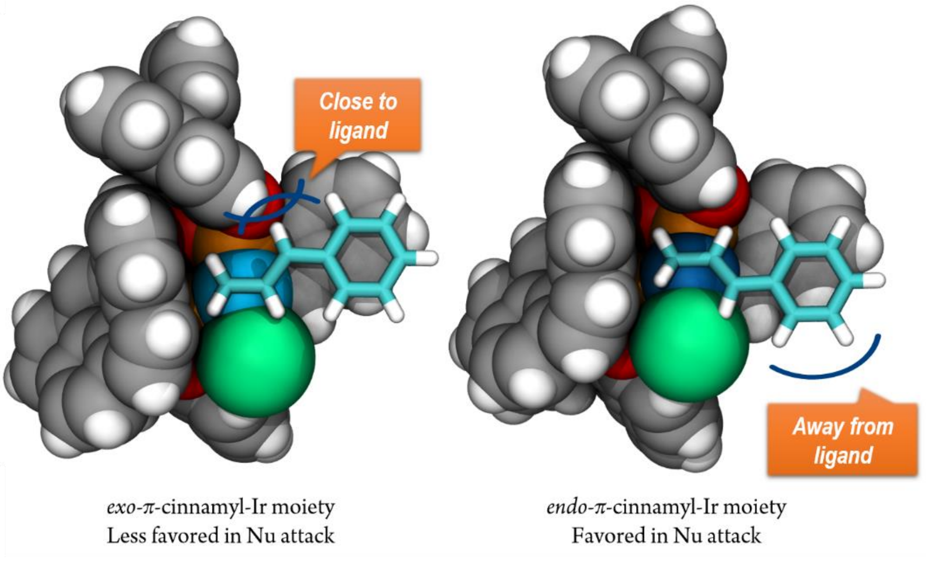

Figure S2. The 3D structure of *exo* or *endo* π-cinnamyl iridium species with the incoming nucleophiles omitted.

For the regioselectivity between C2 and C3 attacks, a total of 24 possible transition states were calculated. The schematic structures (Figure S3) and relative energies (Table S4) are listed below. For each reaction site (C3 or C2), skatole can attack the benzylic carbon *via* its *Re* or *Si* face. Besides, skatole can approach through different trajectories, leading to different conformation of the transition states. These transition states can be distinguished by the relative position (+*sc*, –*sc*, or *ap*) of the benzylic C–H bond and the C3–Me bond (C3 attack) or C2–H bond (C2 attack). In each series, the transition states with the best overlap of phenyl group of the π-cinnamyl iridium moiety and the indole ring is the most stable (highlighted with a gray rectangle). The globally favorite transition states leading to C2 or C3 allylation products are highlighted with a pink rectangle. The calculated Gibbs free energy of **TS-2-*endo*-*Re*-(-*sc*)** [labelled in the main text as **TS-2**] is 2.6 kcal/mol lower than that of **TS-3-*endo*-*Si*-(-*sc*)** [labelled in the main text as **TS-3**], suggesting a direct C2 attack mechanism with the dominant formation of the (*S*) product.

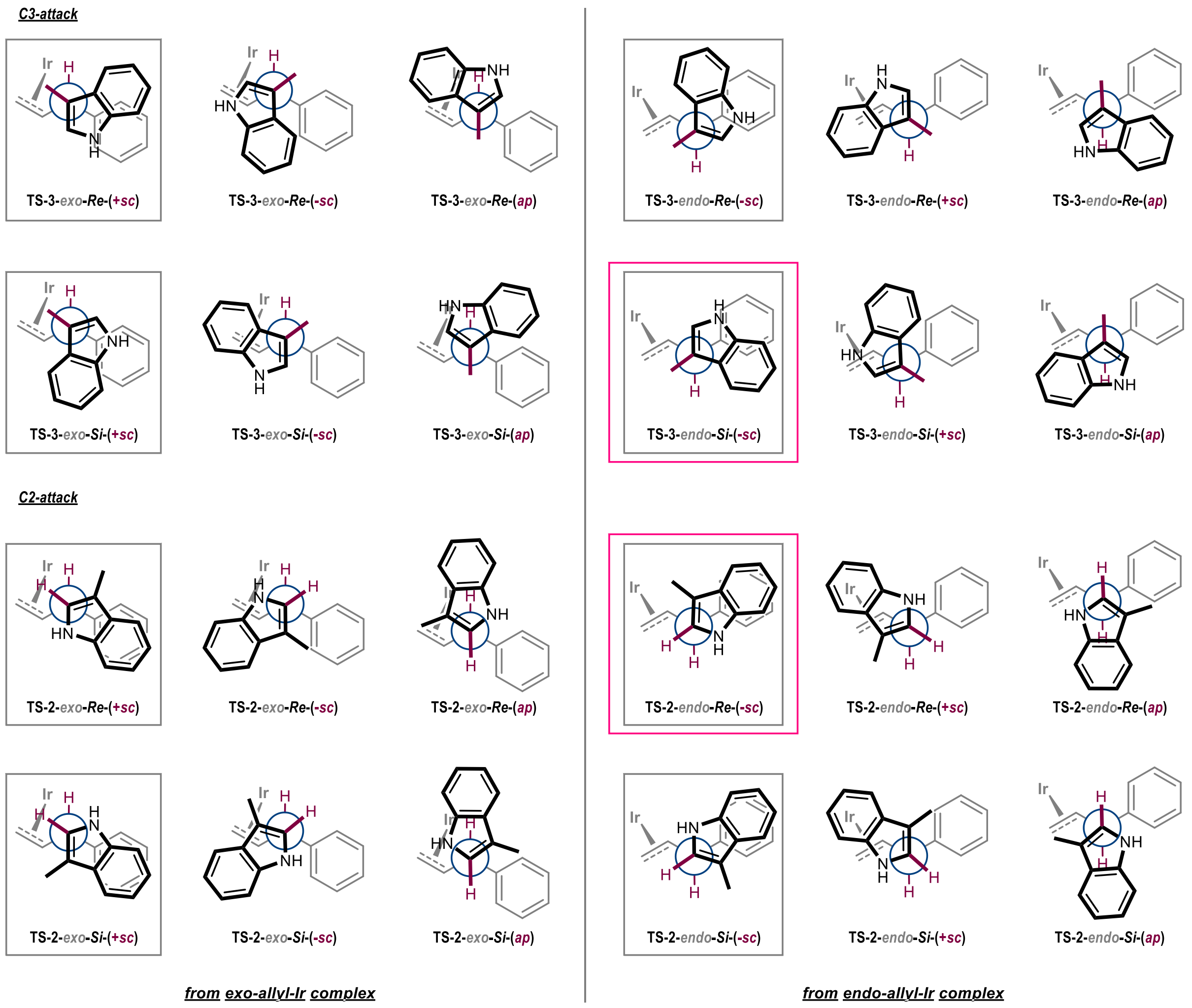

Figure S3. All possible transition states considered.

Table S4. The calculated Gibbs free energies (Δ*G*, in kcal/mol) of transition states.

Transition states	Calculated Gibbs free energies (Δ*G*)
**TS-2-*endo*-*Re*-(-*sc*) [TS-2 in main text]**	0.0
**TS-2-*endo*-*Re*-(+*sc*)**	1.9
**TS-2-*endo*-*Re*-(*ap*)**	4.7
**TS-2-*endo*-*Si*-(-*sc*)**	0.4
**TS-2-*endo*-*Si*-(+*sc*)**	4.9
**TS-2-*endo*-*Si*-(*ap*)**	4.5
**TS-2-*exo*-*Re*-(+*sc*)**	7.9
**TS-2-*exo*-*Re*-(-*sc*)**	10.0
**TS-2-*exo*-*Re*-(*ap*)**	11.8
**TS-2-*exo*-*Si*-(+*sc*)**	6.7

**TS-2-*exo*-*Si*-(-*sc*)**	10.5
**TS-2-*exo*-*Si*-(*ap*)**	11.0
**TS-3-*endo*-*Si*-(-*sc*) [TS-3 in main text]**	2.6
**TS-3-*endo*-*Si*-(+*sc*)**	4.7
**TS-3-*endo*-*Si*-(*ap*)**	5.9
**TS-3-*endo*-*Re*-(-*sc*)**	3.3
**TS-3-*endo*-*Re*-(+*sc*)**	3.5
**TS-3-*endo*-*Re*-(*ap*)**	4.0
**TS-3-*exo*-*Re*-(+*sc*)**	9.9
**TS-3-*exo*-*Re*-(-*sc*)**	12.9
**TS-3-*exo*-*Re*-(*ap*)**	12.1
**TS-3-*exo*-*Si*-(+*sc*)**	9.8
**TS-3-*exo*-*Si*-(-*sc*)**	11.5
**TS-3-*exo*-*Si*-(ap)**	12.3

Cartesian Coordinates and Energies for All Optimized Structures

TS-2-*endo*-*Re*-(-*sc*) [TS-2 in main text]

```
Opt @ B3LYP-D3(BJ)/SDD/def2-SVP [Gaussian16]
SCF Done: E(RB3LYP) = -5024.50617664 a.u.
Zero-point correction = 1.254237 Hartree/Particle
Thermal correction to Gibbs Free Energy = 1.142498 Hartree/Particle
Imaginary Frequency = -326.8208 cm-1
SP @ RI-B3LYP-D3(BJ)/def2-TZVPP in DCM (SMD) [ORCA 4.1]
FINAL SINGLE POINT ENERGY = -5026.539308855050 a.u.
---------------------------------------------------------------------
Ir,0,2.5251178888,5.1057217269,-14.1768847206
Cl,0,4.7999582441,5.253479168,-15.1935216748
C,0,1.6933971053,4.9882301023,-16.1444903061
H,0,2.3859149139,5.4283820393,-16.8640559077
H,0,0.6675621375,5.3460165798,-16.2454439976
C,0,1.9096532725,3.6268088126,-15.7294367491
H,0,1.0454720914,3.056876268,-15.3870207698
C,0,3.0397265695,2.8216659538,-16.2032781123
C,0,3.2114940637,1.4286908699,-15.7358583268
C,0,2.1323781221,0.5423663634,-15.584441777
H,0,1.1135694297,0.8932777772,-15.7555204471
C,0,2.3467853596,-0.7858548524,-15.2226312326
H,0,1.4965620235,-1.4613094616,-15.109681098
C,0,3.6464426927,-1.2523639693,-15.001101877
H,0,3.8133736378,-2.2947755352,-14.7208595487
C,0,4.7304815556,-0.3789475711,-15.1299111581
H,0,5.7464488796,-0.7336544615,-14.9434014376
C,0,4.5120716172,0.9486278303,-15.4992787063
P,0,2.7398684922,7.4089939957,-13.8510349438
O,0,4.230827434,8.0832887215,-13.7150593134
O,0,2.1206033706,7.7739165419,-12.3507368805
N,0,2.1652191057,8.5159291514,-14.9989713735
C,0,4.9895783082,8.100172132,-12.563935039
C,0,6.1974087099,7.3655109376,-12.5935405008
H,0,6.4338591193,6.8124749838,-13.5026260717
C,0,7.0186077135,7.3672475967,-11.4918403465
H,0,7.9603174843,6.8137087186,-11.5059969776
C,0,6.6449579035,8.0607153192,-10.3075927206
C,0,7.449767452,8.0021511644,-9.1368343552
H,0,8.3908371841,7.4483295914,-9.1788906459
C,0,7.0497263545,8.6139258102,-7.9692613764
H,0,7.6744447862,8.5586235239,-7.075234462
C,0,5.8137408727,9.3027251868,-7.9266080407
H,0,5.484240801,9.7656831461,-6.9936425827
C,0,5.0176356238,9.3905501057,-9.0495070157
H,0,4.0633691917,9.9111357023,-8.9912743356
C,0,5.4114117922,8.7947592613,-10.2806876207
C,0,4.5952490124,8.8539787895,-11.4664940226
C,0,3.3416191294,9.6535044622,-11.4935145094
C,0,3.3275958065,11.0562304752,-11.1750183451
C,0,4.5233114801,11.8088758842,-11.0078284714
H,0,5.4843959934,11.3082248531,-11.1171842285
C,0,4.4810635299,13.1594187352,-10.7342431692
H,0,5.4132566326,13.7175422533,-10.622138406
C,0,3.2414488869,13.8320655146,-10.6039619398
H,0,3.2229975459,14.9004007815,-10.3780642594
```

C,0,2.0642535166,13.1385565094,-10.7794153781
H,0,1.101675354,13.6500810044,-10.7026573987
C,0,2.0732340244,11.7494320791,-11.0848246138
C,0,0.8661388973,11.0450029343,-11.3467478754
H,0,-0.0858971636,11.5738704362,-11.2624429244
C,0,0.8992011255,9.7329282734,-11.7580137677
H,0,-0.0071391601,9.1905482375,-12.0263482891
C,0,2.1392933891,9.0616481864,-11.8548860359
C,0,1.1014490465,8.1796616724,-15.894836007
C,0,-0.2149783482,8.1439314789,-15.4264926256
H,0,-0.404543944,8.3950590251,-14.3839709451
C,0,-1.2574755742,7.7757591002,-16.2753773524
H,0,-2.280955942,7.7508241864,-15.9040721732
C,0,-0.9805618224,7.4397593848,-17.6055105281
H,0,-1.7949506986,7.145535763,-18.2707320513
C,0,0.3285470322,7.4873786361,-18.0776104797
H,0,0.5430335273,7.227582851,-19.1174609913
C,0,1.3984922888,7.8669365001,-17.241826251
C,0,2.7596097883,7.8697815412,-17.7728888738
H,0,2.9198408605,7.2171198155,-18.6373264873
C,0,3.8017122413,8.6458207048,-17.3912040731
H,0,4.7292678401,8.5538705664,-17.9638679255
C,0,3.7677028333,9.7284283996,-16.4141503519
C,0,4.5859934195,10.8589413792,-16.6052522662
H,0,5.297451994,10.8591799195,-17.4345915771
C,0,4.4822218205,11.9732822339,-15.7757283112
H,0,5.1293090283,12.8373762157,-15.9414253764
C,0,3.536612422,11.9891035171,-14.7453038219
H,0,3.4343719698,12.8607567037,-14.0956301268
C,0,2.7346711848,10.8690638393,-14.5250224366
H,0,2.0045156725,10.864464473,-13.7216361697
C,0,2.8793414456,9.7310961215,-15.3177914347
P,0,0.5955707887,4.9180025915,-13.0971551194
O,0,-0.2481940851,6.2871974085,-12.8289222406
O,0,-0.5646040912,3.8984753109,-13.6844369107
N,0,0.8145968866,4.186752255,-11.5688721699
C,0,-1.4239946335,6.2654049567,-12.0801638783
C,0,-1.3953722437,6.8956348969,-10.8184268998
H,0,-0.472733505,7.3739016449,-10.49413747
C,0,-2.5251285085,6.8785113472,-10.0349257378
H,0,-2.5250668237,7.3707014787,-9.0596861523
C,0,-3.6946707946,6.190950115,-10.4606814546
C,0,-4.8415035186,6.0987684097,-9.6250001109
H,0,-4.8174385252,6.5902756277,-8.6493850896
C,0,-5.9552478073,5.3945533348,-10.0263902507
H,0,-6.8282988145,5.3263121217,-9.3739113028
C,0,-5.9619867243,4.7433649979,-11.2835928139
H,0,-6.8372325072,4.165194096,-11.5880558522
C,0,-4.8726204341,4.8271836349,-12.1248513708
H,0,-4.8909414122,4.3143616615,-13.085973213
C,0,-3.7140714093,5.5635279352,-11.7521045672
C,0,-2.5599842719,5.6727779173,-12.6058710075
C,0,-2.5751940634,5.1976123709,-14.0143766459
C,0,-3.5669128362,5.6763949923,-14.9452086752
C,0,-4.4803280568,6.7172409643,-14.6158387859
H,0,-4.4473333332,7.1618057936,-13.6226918011
C,0,-5.388231023,7.1892625494,-15.5408763952
H,0,-6.0686376719,7.9977796929,-15.2649120877

```
C,0,-5.4428729937,6.6419205731,-16.8450120895
H,0,-6.1716354057,7.0203877719,-17.5647727943
C,0,-4.5652822847,5.6419321933,-17.2023266773
H,0,-4.5850801565,5.2205457797,-18.210494977
C,0,-3.6037280323,5.1488321207,-16.279064846
C,0,-2.6488292579,4.1707240834,-16.6648988647
H,0,-2.69433603,3.7527958725,-17.6733028888
C,0,-1.6608211654,3.7818795486,-15.7955096268
H,0,-0.9037379079,3.0560908872,-16.0813665911
C,0,-1.6096896121,4.3223461151,-14.4910743866
C,0,1.6202274247,4.9287473235,-10.6223052561
C,0,1.0460125167,5.3294108071,-9.4151964632
H,0,0.0073268581,5.0635797631,-9.2197834447
C,0,1.7984698412,6.0555603663,-8.4903886309
H,0,1.3469195703,6.3706641615,-7.5471917917
C,0,3.1307010654,6.3723522126,-8.7770215818
H,0,3.7295910825,6.9440305264,-8.0659649997
C,0,3.7056613471,5.9551964387,-9.9754340049
H,0,4.7481303036,6.1875499205,-10.1877796721
C,0,2.9661367259,5.2241112112,-10.9182772201
C,0,3.6216670169,4.7757955024,-12.1588463707
H,0,4.60880329,5.2066755308,-12.3291066047
C,0,3.3881222487,3.5180129784,-12.7729675814
H,0,4.2199917948,3.1195509035,-13.3567949448
C,0,2.4613878857,2.4859332544,-12.2618604086
C,0,2.8199759038,1.1294724094,-12.3432041665
H,0,3.7776780291,0.8602950239,-12.7896922826
C,0,1.9736699171,0.1317454355,-11.8625447356
H,0,2.2802316473,-0.9137203934,-11.9352411879
C,0,0.7367724577,0.4636522965,-11.3005376773
H,0,0.0688372449,-0.3182926595,-10.9331572604
C,0,0.3582309761,1.8044448159,-11.2112162814
H,0,-0.6015586719,2.1019489432,-10.7866386719
C,0,1.2169045107,2.8013777431,-11.6716487699
H,0,5.3568194381,1.6360565532,-15.5944270486
H,0,3.9765433574,3.3848370554,-16.1674576455
C,0,3.8082225464,0.7355270949,-18.7127105965
C,0,2.3713688261,0.7089260018,-18.7892060438
C,0,1.7047540634,-0.5086031945,-19.0900167471
C,0,2.462330524,-1.6434214053,-19.277905469
C,0,3.8842451065,-1.5992196562,-19.1789281035
C,0,4.5719731048,-0.4310797344,-18.9013364633
C,0,3.0567702756,2.7761888681,-18.0917606465
C,0,1.9108559638,2.000634775,-18.4855869919
H,0,0.6156745909,-0.5395060598,-19.158705281
H,0,1.9783943114,-2.5953991266,-19.5029826911
H,0,4.4479632528,-2.5228530569,-19.3286954748
H,0,5.6599262529,-0.4154845881,-18.8236179415
H,0,5.13869084,2.2715816481,-18.1771799967
H,0,3.1122627376,3.8523930086,-18.2662052006
N,0,4.1935122876,2.0032030564,-18.4234452638
C,0,0.5040351432,2.4772701285,-18.4806675017
H,0,-0.1439499636,1.7985807888,-17.9017948093
H,0,0.414410801,3.4909642881,-18.0729989108
H,0,0.1077665088,2.4879704799,-19.5104037782
```

TS-2-*endo*-*Re*-(+*sc*)

Opt @ B3LYP-D3(BJ)/SDD/def2-SVP [Gaussian16]
SCF Done: E(RB3LYP) = -5024.50958136 a.u.
Zero-point correction = 1.254551 Hartree/Particle
Thermal correction to Gibbs Free Energy = 1.145316 Hartree/Particle
Imaginary Frequency = -437.2027 cm^{-1}
SP @ RI-B3LYP-D3(BJ)/def2-TZVPP in DCM (SMD) [ORCA 4.1]
FINAL SINGLE POINT ENERGY = -5026.539063648155 a.u.

Ir,0,2.5116000942,5.1909651394,-14.2352639009
Cl,0,4.7642355797,5.3656795987,-15.2809838165
C,0,1.6819765588,5.1221673081,-16.207165761
H,0,2.3824554212,5.5707377502,-16.9090699488
H,0,0.6613780607,5.492340497,-16.3097973283
C,0,1.8621829945,3.7458925494,-15.8254805321
H,0,0.9778328886,3.2129211252,-15.4820802208
C,0,2.9680468789,2.8941592329,-16.2286677029
C,0,3.0536997432,1.5115514352,-15.6934938066
C,0,1.9126347665,0.7487220003,-15.3862892743
H,0,0.9202868942,1.2008905544,-15.4303476901
C,0,2.0331438002,-0.5859016345,-14.9993731703
H,0,1.1375814089,-1.1613989773,-14.7569178153
C,0,3.2948991949,-1.1795495882,-14.9011242784
H,0,3.3870267233,-2.2254196835,-14.6003945739
C,0,4.4396324959,-0.4246728237,-15.1766946521
H,0,5.4295350565,-0.8772481724,-15.086495983
C,0,4.3181838826,0.9071734925,-15.573792902
P,0,2.7153974044,7.4815194018,-13.8620234376
O,0,4.2067452833,8.153867971,-13.7397456405
O,0,2.1185531095,7.8089640138,-12.3450398355
N,0,2.1173912599,8.6155237192,-14.9683611183
C,0,4.9881945726,8.1488527229,-12.6031070349
C,0,6.1982249345,7.420525617,-12.67275622
H,0,6.4203479634,6.8901619348,-13.5987103077
C,0,7.0386800043,7.3984099014,-11.5859224747
H,0,7.9818452221,6.849054291,-11.6306039122
C,0,6.6837397293,8.0611657685,-10.3785459004
C,0,7.5095276223,7.9770751442,-9.2240357397
H,0,8.4518654958,7.4286362137,-9.2964871634
C,0,7.127644521,8.5576558174,-8.0346801091
H,0,7.7682104555,8.4827374843,-7.1534142817
C,0,5.889757685,9.2394486913,-7.9529551551
H,0,5.5748765113,9.6771545809,-7.0029140494
C,0,5.0738369199,9.3524772618,-9.0593047588
H,0,4.1187943612,9.8674782301,-8.9714503897
C,0,5.4485415275,8.7898249947,-10.3118065002
C,0,4.6108393237,8.8744069212,-11.4811156465
C,0,3.3552090653,9.6706271533,-11.4666644634
C,0,3.3463817064,11.0658026708,-11.1151073741
C,0,4.5446361729,11.8147566755,-10.9487570554
H,0,5.5040846727,11.317568404,-11.0846473206
C,0,4.506645984,13.1583130271,-10.6419521228
H,0,5.4405388056,13.7137159532,-10.5305842117
C,0,3.2693013323,13.8272998404,-10.4762258879
H,0,3.2544893924,14.8897628279,-10.2239894121
C,0,2.0895273301,13.1380966819,-10.6508855272
H,0,1.1282926416,13.6473723876,-10.5473517889
C,0,2.0938169571,11.756794474,-10.9899808434
C,0,0.8828124943,11.0587713156,-11.2505639002

H,0,-0.0677405409,11.585308096,-11.1395301161
C,0,0.9091681782,9.7559684842,-11.690571587
H,0,-0.002123152,9.2190387807,-11.9528728888
C,0,2.1470265436,9.0866025831,-11.8215782189
C,0,0.9933995506,8.327881048,-15.8043079278
C,0,-0.2920400299,8.3035715616,-15.2566250176
H,0,-0.4115238377,8.5203515658,-14.1968130196
C,0,-1.3937201146,7.989881602,-16.0508508441
H,0,-2.3923047689,7.9682934074,-15.616388577
C,0,-1.2082911395,7.7076533108,-17.4084273944
H,0,-2.0677300318,7.4582338483,-18.033595389
C,0,0.0694787377,7.7494241114,-17.9587154031
H,0,0.2091182041,7.5360835355,-19.0184904236
C,0,1.1996020037,8.0606299329,-17.1773350236
C,0,2.5264371903,8.0459946633,-17.7893147884
H,0,2.6164511448,7.4149483496,-18.6781336965
C,0,3.6131442599,8.7775791833,-17.4464442874
H,0,4.5033866654,8.6771751876,-18.0744526131
C,0,3.6650095039,9.8315330156,-16.4388620542
C,0,4.4968956122,10.9489512632,-16.6469113599
H,0,5.162532751,10.9559134881,-17.5134378209
C,0,4.4611973762,12.0442084074,-15.7866987171
H,0,5.1171484104,12.898775554,-15.9662297769
C,0,3.5705750631,12.0546310729,-14.7081919709
H,0,3.5198433506,12.9129502899,-14.0351320907
C,0,2.7577278496,10.9459567685,-14.4711979436
H,0,2.0696150619,10.9373697719,-13.631689448
C,0,2.837872208,9.8244777288,-15.2959853335
P,0,0.5890261532,4.9637037836,-13.1509724153
O,0,-0.2663047849,6.3163064369,-12.83360307
O,0,-0.5610068265,3.9542653891,-13.7745430938
N,0,0.8235864674,4.2064185235,-11.638395172
C,0,-1.4297031783,6.2537698796,-12.066710208
C,0,-1.396771575,6.8636183218,-10.7952513496
H,0,-0.4837845416,7.3658640524,-10.480209672
C,0,-2.5091046456,6.7953350448,-9.9895360127
H,0,-2.505135906,7.2707342466,-9.0060084371
C,0,-3.6628464519,6.0744589354,-10.4029970322
C,0,-4.7877315528,5.9252339337,-9.5458884045
H,0,-4.7594779738,6.39593268,-8.5601819585
C,0,-5.8857135961,5.1927834842,-9.9402133653
H,0,-6.7416934983,5.0806717067,-9.2713910208
C,0,-5.8987528764,4.5703389574,-11.2120317016
H,0,-6.7613091718,3.9708263667,-11.5113552884
C,0,-4.831308136,4.7095018302,-12.0736865733
H,0,-4.8529817632,4.2198831154,-13.0469940495
C,0,-3.6895034363,5.474391876,-11.7070558782
C,0,-2.5595646429,5.640593393,-12.5825709636
C,0,-2.5991599477,5.2262852358,-14.0096800522
C,0,-3.6224944064,5.733675234,-14.889898824
C,0,-4.5621625146,6.7224069686,-14.4816446243
H,0,-4.5254171205,7.1073121616,-13.4642969356
C,0,-5.5026632988,7.2192439465,-15.3598041457
H,0,-6.2036013887,7.9857808618,-15.0222898249
C,0,-5.5651499007,6.7515062812,-16.6943863104
H,0,-6.3203094298,7.1481345632,-17.376174457
C,0,-4.6612591622,5.8069062903,-17.1277473085
H,0,-4.6851980427,5.4481732796,-18.1598695313

C,0,-3.6666137072,5.2910242351,-16.2530363399
C,0,-2.6824785826,4.3809186838,-16.7187032269
H,0,-2.7188820023,4.0440199668,-17.7550814508
C,0,-1.6668415212,3.9635918714,-15.8954226981
H,0,-0.8861031833,3.29985065,-16.2624655182
C,0,-1.6198769757,4.4064155096,-14.5531763839
C,0,1.6133048528,4.9503289724,-10.6771516002
C,0,1.0335455997,5.3059770089,-9.4586201442
H,0,0.002147398,5.0112642214,-9.267498933
C,0,1.7717740934,6.0237809065,-8.5161968422
H,0,1.3149956594,6.3037253363,-7.5644742554
C,0,3.0969355882,6.375548565,-8.7949419898
H,0,3.6855448778,6.9393202846,-8.0690753727
C,0,3.6794228435,5.9999481653,-10.0032225578
H,0,4.7179656128,6.2546485304,-10.2078292553
C,0,2.9538027942,5.2789114564,-10.9643247507
C,0,3.6239149685,4.8629616555,-12.2083409921
H,0,4.5965274218,5.3252248917,-12.3791703807
C,0,3.422168424,3.6048561892,-12.8246267301
H,0,4.256676507,3.2311135292,-13.4214181293
C,0,2.5297793732,2.5519956006,-12.2961886003
C,0,2.9581384918,1.2142703589,-12.3042683697
H,0,3.9367127253,0.9737960828,-12.7215174761
C,0,2.1549812822,0.1992864496,-11.7877205566
H,0,2.5168927414,-0.830643155,-11.7983735901
C,0,0.8882916965,0.4926613101,-11.2730578407
H,0,0.252422101,-0.3044265988,-10.8820715583
C,0,0.442523417,1.8155926212,-11.2514080634
H,0,-0.5353489036,2.0845724047,-10.8496106334
C,0,1.263903117,2.8330203033,-11.7349192066
H,0,5.2124327282,1.4945328467,-15.7995053199
H,0,3.9345034433,3.3984434392,-16.2870807525
C,0,0.8018302145,2.8687677637,-18.9922428594
C,0,1.6282816626,3.9967544604,-19.3354605505
C,0,1.0652001407,5.0738824308,-20.0671520049
C,0,-0.2679458002,5.0168498558,-20.4143102441
C,0,-1.0717884507,3.9018707171,-20.0446717076
C,0,-0.5631226974,2.8271378265,-19.3345597411
C,0,2.8605413106,2.4896114235,-18.1408426766
C,0,2.9252688505,3.7365362933,-18.8487724377
H,0,1.6841841874,5.9248329227,-20.3556571042
H,0,-0.7215787838,5.8304673888,-20.9830026386
H,0,-2.1224728,3.8912594724,-20.344032713
H,0,-1.1882922368,1.9728073595,-19.0692804513
H,0,1.2515710104,1.0924693467,-17.9371158274
H,0,3.701174003,1.796221197,-18.1441367383
N,0,1.5765913379,1.9526329043,-18.3622288707
C,0,4.1382725292,4.5884207983,-18.9479300944
H,0,4.0062450366,5.3978940548,-19.6780173684
H,0,4.3714184282,5.0459594176,-17.9674747
H,0,5.0202593704,3.9959957871,-19.2371124008

TS-2-*endo*-*Re*-(*ap*)
Opt @ B3LYP-D3(BJ)/SDD/def2-SVP [Gaussian16]
SCF Done: E(RB3LYP) = -5024.50307045 a.u.
Zero-point correction = 1.254148 Hartree/Particle
Thermal correction to Gibbs Free Energy = 1.143879 Hartree/Particle

Imaginary Frequency = -424.0817 cm^{-1}
SP @ RI-B3LYP-D3(BJ)/def2-TZVPP in DCM (SMD) [ORCA 4.1]
FINAL SINGLE POINT ENERGY = -5026.533233556192 a.u.

Ir,0,2.6365236688,5.1484971736,-14.1286059868
Cl,0,4.9298810617,5.2755602365,-15.0712805402
C,0,1.8124063006,5.0943480425,-16.0927783461
H,0,2.5156264567,5.5440538517,-16.7935395357
H,0,0.7904112437,5.4679787146,-16.183348949
C,0,2.0354314647,3.7137509627,-15.7363988976
H,0,1.2017340281,3.11653313,-15.3648171143
C,0,3.142268774,2.9581871883,-16.3059954697
C,0,3.5336841988,1.6537539327,-15.7243664477
C,0,2.6001280664,0.6294119728,-15.4961502661
H,0,1.5382045225,0.8040326874,-15.6849137223
C,0,3.0167944931,-0.6228743508,-15.0478493543
H,0,2.2818163189,-1.4146520775,-14.8897684702
C,0,4.3708107429,-0.8596325809,-14.7856393813
H,0,4.6967762575,-1.8408539944,-14.4336942749
C,0,5.3027140928,0.1675246037,-14.962308822
H,0,6.3580122605,-0.0058861677,-14.740415744
C,0,4.8893697747,1.4130814939,-15.4405839412
P,0,2.8461607929,7.4386201405,-13.7517670852
O,0,4.333587504,8.1021257505,-13.5575271357
O,0,2.1809203427,7.7759517831,-12.2667328005
N,0,2.3112297745,8.5738564651,-14.8923963651
C,0,5.0579085081,8.1054035838,-12.3844764138
C,0,6.2654840363,7.3699144549,-12.3896170925
H,0,6.5257143736,6.8267340718,-13.2983798291
C,0,7.0554997918,7.3585614466,-11.2655119389
H,0,7.9968692235,6.8043413165,-11.2594815596
C,0,6.6491079067,8.0392650902,-10.0845459557
C,0,7.4220722242,7.9675423926,-8.8932207505
H,0,8.3643418719,7.4146899808,-8.9163471032
C,0,6.9901311531,8.5653120049,-7.7297922339
H,0,7.5905186939,8.4999058418,-6.8199373051
C,0,5.7525897104,9.252235249,-7.7124730849
H,0,5.3971667879,9.7034669676,-6.783293423
C,0,4.9871358667,9.3529610083,-8.8554790662
H,0,4.0309147458,9.8715899256,-8.8164280339
C,0,5.4148655136,8.7728698508,-10.0828124781
C,0,4.6311384212,8.8456536476,-11.2900747112
C,0,3.3754531722,9.6412964186,-11.3397831696
C,0,3.3469003815,11.0379046184,-10.9957215908
C,0,4.5349834867,11.7927416286,-10.7893209112
H,0,5.5006588347,11.2985759286,-10.8872363419
C,0,4.4803480698,13.1379466101,-10.4927005923
H,0,5.4071929445,13.6982351574,-10.3510511662
C,0,3.2349596811,13.8024091441,-10.3764575907
H,0,3.2064317377,14.8663475122,-10.1316716601
C,0,2.0651256418,13.1066456045,-10.58873157
H,0,1.0986049223,13.6121714826,-10.5224891139
C,0,2.0875174529,11.7234547314,-10.9193142365
C,0,0.8898038478,11.0187035375,-11.220531394
H,0,-0.0666291129,11.541015572,-11.1455657033
C,0,0.9390388121,9.7162136139,-11.6595624156
H,0,0.0423887208,9.1757552682,-11.9619789038
C,0,2.1844690981,9.0531930633,-11.742865936

C,0,1.3197354676,8.2522444125,-15.8714710209
C,0,-0.0274503024,8.187398364,-15.5039536168
H,0,-0.2964536589,8.3980784741,-14.4696309719
C,0,-0.9999105619,7.8397449986,-16.4403834374
H,0,-2.0475656524,7.7904485906,-16.1474041004
C,0,-0.6215793312,7.5611995385,-17.7588589322
H,0,-1.3818544928,7.2935931778,-18.4957682431
C,0,0.7190222686,7.6376716551,-18.1289366297
H,0,1.007655433,7.4384693552,-19.1639431676
C,0,1.7222548433,7.9860976066,-17.2012888442
C,0,3.1233957179,7.9983662508,-17.6184539544
H,0,3.3651454862,7.3476791865,-18.4633542523
C,0,4.1261441605,8.7713405979,-17.1392351993
H,0,5.1011931996,8.6844869838,-17.627432737
C,0,4.0003553179,9.8387950981,-16.1537157624
C,0,4.8138461171,10.9832870449,-16.2589833725
H,0,5.5901888265,11.0086000146,-17.027492907
C,0,4.6251134756,12.0807296719,-15.4215432221
H,0,5.2696460581,12.9570356166,-15.5188550543
C,0,3.5993924138,12.0638100672,-14.4710931902
H,0,3.4321718634,12.9215526467,-13.8164917867
C,0,2.800956323,10.9277265594,-14.3342887872
H,0,2.0108205559,10.8963742665,-13.5901322331
C,0,3.0276062567,9.8074005043,-15.1322247166
P,0,0.6688177637,4.9501854757,-13.1150273113
O,0,-0.1882793311,6.317093309,-12.8756264706
O,0,-0.4629468299,3.9305118671,-13.7604296293
N,0,0.8314743979,4.2134711353,-11.5843421694
C,0,-1.4054174392,6.2847085072,-12.1954689599
C,0,-1.4496926021,6.8910566614,-10.9225716895
H,0,-0.5466809377,7.3591949523,-10.5345202485
C,0,-2.6245437545,6.8649563176,-10.2086724725
H,0,-2.6810994788,7.3387401419,-9.2259846407
C,0,-3.7697704306,6.1929162132,-10.7172334126
C,0,-4.9658281337,6.0940798535,-9.9545519855
H,0,-4.9984461044,6.5683536592,-8.9707039443
C,0,-6.0570712898,5.4046338421,-10.4354635071
H,0,-6.9686558387,5.330993499,-9.8386696328
C,0,-5.99083231,4.7753397789,-11.7019936822
H,0,-6.8491240966,4.2083875404,-12.0692791902
C,0,-4.8516598915,4.8659166083,-12.4737592105
H,0,-4.8145952475,4.3692132378,-13.4427115616
C,0,-3.7138383566,5.5878631154,-12.0182785658
C,0,-2.5087806289,5.7042474864,-12.7976230027
C,0,-2.4391964882,5.2421260781,-14.2091264949
C,0,-3.3690555147,5.7357529598,-15.1936811065
C,0,-4.285483128,6.7882114152,-14.913836944
H,0,-4.3035066368,7.2293099257,-13.918637499
C,0,-5.13183883,7.2745395194,-15.8885676645
H,0,-5.8160651201,8.0917998526,-15.6504243634
C,0,-5.1190671057,6.7295259465,-17.1948524623
H,0,-5.8004984699,7.1187536613,-17.954159914
C,0,-4.2374182619,5.7167596875,-17.5034702099
H,0,-4.2072513074,5.2954409299,-18.5113966185
C,0,-3.3373114158,5.2092430982,-16.5275909276
C,0,-2.3792203266,4.2136762093,-16.8574201024
H,0,-2.3811561336,3.7888772065,-17.8639793929
C,0,-1.4448711189,3.8114435593,-15.9354270029

H,0,-0.6899274196,3.0662602642,-16.1814709585
C,0,-1.4565261127,4.3569213725,-14.6299572422
C,0,1.600759409,4.9530382471,-10.605198112
C,0,0.979442497,5.3494610147,-9.4202577253
H,0,-0.0670525598,5.085629727,-9.2684473776
C,0,1.6965245098,6.0661355256,-8.4607129048
H,0,1.208028837,6.3774382384,-7.5348428521
C,0,3.0414501286,6.3763123891,-8.6891703914
H,0,3.6140201868,6.9381793151,-7.9492771909
C,0,3.6627774232,5.9649416939,-9.8660851135
H,0,4.7143798657,6.1924215712,-10.0333201463
C,0,2.9590128603,5.24552349,-10.8446699869
C,0,3.6654856702,4.8053934289,-12.0599440563
H,0,4.653739162,5.2453261939,-12.1973585412
C,0,3.4570411844,3.5549615527,-12.6887714091
H,0,4.3022017602,3.1633418754,-13.25729521
C,0,2.5140628568,2.5223097131,-12.2116095485
C,0,2.8907857859,1.1703729829,-12.265954169
H,0,3.8691252402,0.9114160749,-12.671363353
C,0,2.0381025397,0.1677055673,-11.8074537829
H,0,2.3586808485,-0.8751388401,-11.8558055459
C,0,0.7765027701,0.491048365,-11.2974605234
H,0,0.103601164,-0.2950348344,-10.9485094718
C,0,0.3799455613,1.8282545307,-11.2351429889
H,0,-0.5980219627,2.1185595251,-10.8488471349
C,0,1.2454687139,2.8301038959,-11.6713906399
H,0,5.612049045,2.2183847963,-15.5905905132
H,0,3.9922220346,3.5867797183,-16.5766872259
C,0,3.0718314741,4.1349151516,-19.6022142515
C,0,4.1000033299,3.1360034001,-19.6689209094
C,0,5.2209986039,3.3348669223,-20.5157064893
C,0,5.2942203347,4.5001268554,-21.2511085222
C,0,4.2656641093,5.480155412,-21.168323415
C,0,3.1541804285,5.3191196074,-20.3558083382
C,0,2.528895126,2.5048175754,-18.1255231373
C,0,3.7228436186,2.0828753027,-18.8082755791
H,0,6.0062278342,2.5790296123,-20.5788953988
H,0,6.145907041,4.6804867394,-21.9091872974
H,0,4.3544346043,6.3872951129,-21.7704860128
H,0,2.3672970302,6.0716419154,-20.3134490078
H,0,1.3595037904,4.2796931899,-18.3879990709
H,0,1.7551498769,1.7952734885,-17.830286288
N,0,2.1124379624,3.7014760485,-18.7425219376
C,0,4.4424496705,0.797578476,-18.6168955165
H,0,4.7177228401,0.3678855459,-19.593209685
H,0,5.380515299,0.956740676,-18.0585002939
H,0,3.8479999784,0.0646652886,-18.059137689

TS-2-*endo*-*Si*-(-*sc*)
Opt @ B3LYP-D3(BJ)/SDD/def2-SVP [Gaussian16]
SCF Done: E(RB3LYP) = -5024.50483203 a.u.
Zero-point correction = 1.253870 Hartree/Particle
Thermal correction to Gibbs Free Energy = 1.141459 Hartree/Particle
Imaginary Frequency = -399.7581 cm^{-1}
SP @ RI-B3LYP-D3(BJ)/def2-TZVPP in DCM (SMD) [ORCA 4.1]
FINAL SINGLE POINT ENERGY = -5026.537633272284 a.u.

Ir,0,0.5439382289,-1.0071749162,-0.048010008
Cl,0,0.396531682,-3.3431810643,0.7970372624
C,0,1.8545237344,-0.5329325139,1.5719047393
H,0,1.7049310415,-1.2439629439,2.3868894636
H,0,1.8578202582,0.5103440258,1.891428562
C,0,2.7028211685,-0.9250287884,0.4752760524
H,0,3.1895028302,-0.1390295268,-0.1012654618
C,0,3.3503879996,-2.2331277428,0.3967995582
C,0,4.1969186946,-2.5648041035,-0.765805524
C,0,5.0543617667,-1.6264526908,-1.3617151342
H,0,5.0525361474,-0.5906421982,-1.0219987924
C,0,5.9148938285,-2.0026496322,-2.3942619561
H,0,6.5773512969,-1.260372141,-2.8435706777
C,0,5.9190753309,-3.3184178184,-2.8590928061
H,0,6.592087329,-3.6124215016,-3.6675525507
C,0,5.0477302974,-4.2586367674,-2.294618462
H,0,5.0324860375,-5.2846863615,-2.6688222387
C,0,4.2006987457,-3.8859382913,-1.253526054
P,0,-1.5689123332,-0.7304898041,0.9016716013
O,0,-2.560126049,-2.0244040432,1.0885669527
O,0,-2.4819551378,0.1882411496,-0.1416421655
N,0,-1.7488237255,-0.1558470847,2.4853041486
C,0,-3.3530495593,-2.5650837878,0.0986022605
C,0,-3.0317429183,-3.8769033415,-0.3199150097
H,0,-2.1678349769,-4.35930871,0.1376849804
C,0,-3.8025391027,-4.4893571097,-1.2784809228
H,0,-3.5728858422,-5.5070802942,-1.6023433912
C,0,-4.8895535837,-3.8018635973,-1.8858452474
C,0,-5.6429375002,-4.3991845709,-2.9334365568
H,0,-5.3988709453,-5.421319408,-3.2330105188
C,0,-6.647613608,-3.7045765743,-3.5703281321
H,0,-7.2167550353,-4.1725579027,-4.3763471945
C,0,-6.9298730435,-2.3714055203,-3.1871290713
H,0,-7.708966244,-1.811666883,-3.7096507725
C,0,-6.2274225411,-1.7691194456,-2.1642488918
H,0,-6.4494191422,-0.7378486991,-1.8955688168
C,0,-5.2018149024,-2.4652399712,-1.4647075832
C,0,-4.4384864386,-1.8595839456,-0.4032626313
C,0,-4.7697904996,-0.4991767049,0.0981277986
C,0,-6.0824858828,-0.1679381355,0.5846710817
C,0,-7.0825555718,-1.1559256251,0.8036342928
H,0,-6.852829952,-2.1990898412,0.5907413163
C,0,-8.3223253185,-0.8148405953,1.3008340363
H,0,-9.069010899,-1.5935332208,1.4715363151
C,0,-8.6354028036,0.5338716613,1.5991701066
H,0,-9.6247265803,0.7907273298,1.9838282654
C,0,-7.6842203754,1.5137274234,1.4185221068
H,0,-7.9058266742,2.5549650933,1.6651157682
C,0,-6.3881120482,1.1922748333,0.9288255562
C,0,-5.3760973722,2.1847934954,0.8166931217
H,0,-5.6184268194,3.2203364455,1.0658859467
C,0,-4.0949209441,1.8370547077,0.4570244722
H,0,-3.2869697595,2.5678021352,0.4260927624
C,0,-3.8003399517,0.493754466,0.1308257762
C,0,-0.7521662917,0.6628005817,3.1044954025
C,0,-0.639214135,2.0091123313,2.7465068479
H,0,-1.3324696364,2.4126155585,2.009983328
C,0,0.3536353552,2.8090164543,3.3099503153

```
H,0,0.4360652718,3.8576468855,3.027530121
C,0,1.2411113449,2.2569458746,4.24088558
H,0,2.0225876632,2.88036474,4.6801883387
C,0,1.1220841752,0.919108743,4.6081615107
H,0,1.814375442,0.4901672366,5.3371888806
C,0,0.1207012039,0.0921914256,4.0599127778
C,0,0.0560473905,-1.3146786825,4.449286133
H,0,0.9969028822,-1.7365503145,4.817430005
C,0,-1.0345041302,-2.1158132708,4.5001138159
H,0,-0.8936485432,-3.127662294,4.8912640132
C,0,-2.4173240965,-1.717071126,4.2617975275
C,0,-3.4514141105,-2.3293613077,4.9965097463
H,0,-3.202836938,-3.1570631643,5.6651359162
C,0,-4.7669038119,-1.8807469293,4.9018034441
H,0,-5.5526411292,-2.3728728978,5.4790904852
C,0,-5.0738629567,-0.789590432,4.0823959425
H,0,-6.0980550748,-0.4182397608,4.0087787639
C,0,-4.0674367774,-0.1864713827,3.3277526113
H,0,-4.2944320639,0.6541293058,2.6792739398
C,0,-2.7616763584,-0.6730658592,3.3773011005
P,0,0.6292457781,1.00540558,-0.9808298296
O,0,-0.4114715647,2.1332579888,-0.4312645856
O,0,2.0671527604,1.8245340594,-0.988281858
N,0,0.3856809832,0.9136729863,-2.6683257625
C,0,-0.4701338106,3.39667561,-1.0175081588
C,0,-1.6387586056,3.717539861,-1.7395523298
H,0,-2.4371384398,2.9795234094,-1.7964031856
C,0,-1.730616069,4.9434947869,-2.3552426982
H,0,-2.6322682734,5.2139520048,-2.9094998519
C,0,-0.6450197938,5.8608897197,-2.3139429034
C,0,-0.6979787332,7.0979424491,-3.0131710092
H,0,-1.6054189079,7.3447076539,-3.5695995966
C,0,0.3739567011,7.962989458,-3.003704024
H,0,0.3237782689,8.9080250478,-3.5486465604
C,0,1.5514964904,7.6180643264,-2.2973257997
H,0,2.4085610235,8.2949963628,-2.3124214341
C,0,1.629125047,6.4337484689,-1.5952709047
H,0,2.5452952298,6.180105878,-1.0629572035
C,0,0.5339095732,5.5265673565,-1.5654899628
C,0,0.579945785,4.2823717191,-0.8423602351
C,0,1.6939947375,3.9542880894,0.086171424
C,0,2.0336976108,4.8397292779,1.1719787639
C,0,1.24583428,5.9788622341,1.5000983645
H,0,0.3626172659,6.2060703152,0.9056562265
C,0,1.5676890526,6.7836782303,2.573088112
H,0,0.9379804921,7.6439109991,2.8102387045
C,0,2.7008207334,6.5030423868,3.3735345609
H,0,2.9476601918,7.1530033573,4.21553501
C,0,3.4792574944,5.4019230697,3.0919251448
H,0,4.3482836408,5.1641525733,3.7104505956
C,0,3.1616568137,4.5411765654,2.0063551819
C,0,3.9197150755,3.3669844697,1.7537847481
H,0,4.7954991988,3.1602936552,2.373415605
C,0,3.5383303925,2.4898619038,0.7692864374
H,0,4.0916763431,1.5719725145,0.5757417502
C,0,2.4074583422,2.7698937561,-0.0332612268
C,0,-0.9153492878,0.4399641688,-3.0924863088
C,0,-1.7210776561,1.280406252,-3.861747372
```

H,0,-1.3414175496,2.2677536031,-4.1239519627
C,0,-2.9824920778,0.850911757,-4.2777529372
H,0,-3.6127907855,1.5107210876,-4.8778879875
C,0,-3.4299145785,-0.427262651,-3.9265976718
H,0,-4.4152046385,-0.7753260338,-4.2414314396
C,0,-2.6158744532,-1.2697111412,-3.1727712498
H,0,-2.9596950367,-2.2705447973,-2.9162703449
C,0,-1.34491274,-0.8564975367,-2.7435294598
C,0,-0.5059953359,-1.7911917152,-1.9737316873
H,0,-1.0218065597,-2.6898910163,-1.6341475825
C,0,0.8973623048,-1.9114813718,-2.1291270707
H,0,1.3165604159,-2.8886376083,-1.8815321713
C,0,1.711159314,-1.1263996071,-3.080839515
C,0,2.763287429,-1.7515994807,-3.7714943138
H,0,2.9708907164,-2.8050124253,-3.580155066
C,0,3.5379058487,-1.047221856,-4.6916132089
H,0,4.3453359874,-1.560037274,-5.2179808439
C,0,3.2932546393,0.3086710752,-4.9308473207
H,0,3.9071600615,0.8637202144,-5.6433075687
C,0,2.2565691161,0.9522418589,-4.2523644004
H,0,2.0397253821,2.0100107342,-4.4075920433
C,0,1.4664760595,0.2385399523,-3.3527072081
H,0,3.5203567033,-4.6165207357,-0.8101440563
H,0,2.7060698799,-3.0542157529,0.7174090613
C,0,6.6281372575,-1.8262131858,1.3303313542
C,0,6.4738212097,-3.2557210226,1.3677992125
C,0,7.5391342987,-4.0868068667,0.9350194675
C,0,8.7022370012,-3.4926761694,0.4935060346
C,0,8.836812771,-2.0748029822,0.4719399298
C,0,7.8202477464,-1.2287860178,0.8812421803
C,0,4.5017063344,-2.2700324913,1.9574969368
C,0,5.1746333188,-3.5283026081,1.8377087938
H,0,7.4323048593,-5.1729062134,0.9517656805
H,0,9.5370121405,-4.1078982995,0.1533798583
H,0,9.7752842808,-1.6427496213,0.116890698
H,0,7.935866186,-0.1440789248,0.8508451203
H,0,5.2624877984,-0.2753734424,1.7778617799
H,0,3.7114944543,-2.0905523693,2.6869046294
N,0,5.471083671,-1.2655088834,1.7691163144
C,0,4.5415782228,-4.854207026,2.0591538093
H,0,4.8660469004,-5.276985857,3.0259772969
H,0,4.8340112722,-5.5720609989,1.2775987851
H,0,3.4444521413,-4.7817230171,2.082200628

TS-2-*endo*-*Si*-(+*sc*)
Opt @ B3LYP-D3(BJ)/SDD/def2-SVP [Gaussian16]
SCF Done: E(RB3LYP) = -5024.50478307 a.u.
Zero-point correction = 1.254863 Hartree/Particle
Thermal correction to Gibbs Free Energy = 1.145317 Hartree/Particle
Imaginary Frequency = -359.9480 cm^{-1}
SP @ RI-B3LYP-D3(BJ)/def2-TZVPP in DCM (SMD) [ORCA 4.1]
FINAL SINGLE POINT ENERGY = -5026.534285094167 a.u.

Ir,0,2.1453172884,5.6119041353,-14.6065867504
Cl,0,4.4711142234,5.7282601628,-15.4993242023
C,0,1.533024754,5.4178946561,-16.648216575
H,0,2.3111172114,5.846668588,-17.2766957753

H,0,0.5292753874,5.7723465625,-16.8867296336
C,0,1.6562306748,4.073239112,-16.1449983823
H,0,0.7180434905,3.589152189,-15.8935880044
C,0,2.763332396,3.138148275,-16.3327568176
C,0,2.7194026684,1.826526739,-15.6293021504
C,0,1.5234214761,1.2375320874,-15.1851047045
H,0,0.583044369,1.7858335289,-15.2377984587
C,0,1.5200554059,-0.0526383413,-14.6568292555
H,0,0.5813007475,-0.4903208866,-14.3127970456
C,0,2.7105297337,-0.7752226827,-14.5503319218
H,0,2.7040339975,-1.7881696686,-14.1420301749
C,0,3.9156770615,-0.1844354618,-14.944643835
H,0,4.8554527327,-0.7309452076,-14.8395478539
C,0,3.9168803922,1.1052060631,-15.4761771874
P,0,2.2710157459,7.9408812842,-14.3922024175
O,0,3.7353433316,8.6571188073,-14.1972751695
O,0,1.5485012012,8.3463834099,-12.9507894223
N,0,1.744441514,8.9961440496,-15.6091889233
C,0,4.4251348654,8.7478725714,-13.0068649523
C,0,5.6526515823,8.049125466,-12.9381102224
H,0,5.9543760173,7.464629349,-13.8074877313
C,0,6.4095880169,8.1206879753,-11.7938359993
H,0,7.365070765,7.5948153746,-11.7318150863
C,0,5.950494023,8.8515831075,-10.6633113037
C,0,6.6899112322,8.8656399848,-9.4488645379
H,0,7.6474391503,8.340181382,-9.4161529619
C,0,6.2073581221,9.510702793,-8.3315447209
H,0,6.7821141267,9.510871087,-7.4029888894
C,0,4.9511127453,10.1606404513,-8.3856875243
H,0,4.5557331184,10.6492717196,-7.4922411186
C,0,4.2174491497,10.1790361256,-9.5535913189
H,0,3.246620421,10.6709806254,-9.5689929978
C,0,4.6972377411,9.5482466273,-10.735879678
C,0,3.94686641,9.533403057,-11.9666249761
C,0,2.6716180481,10.288464183,-12.0914137462
C,0,2.5980460284,11.7002429691,-11.8233890284
C,0,3.7604103297,12.4965961453,-11.6258886135
H,0,4.7406681412,12.0240815894,-11.6711195652
C,0,3.6635785551,13.8536542783,-11.4030889619
H,0,4.5717204481,14.4449484591,-11.2663946377
C,0,2.3991556604,14.490107357,-11.3564476618
H,0,2.3369527338,15.5643112455,-11.1696301091
C,0,1.2538611483,13.753360675,-11.5638448752
H,0,0.2734630249,14.2358191497,-11.5519105538
C,0,1.3204991252,12.3557282177,-11.8192306487
C,0,0.1500613179,11.6046158177,-12.1154155786
H,0,-0.820912784,12.1045600766,-12.0951844151
C,0,0.2445125992,10.2819823288,-12.480319611
H,0,-0.6300940803,9.7017141715,-12.7738608992
C,0,1.507725929,9.647903174,-12.4945145792
C,0,0.7245911534,8.6286304799,-16.5399402778
C,0,-0.6085034951,8.5674742279,-16.1247687047
H,0,-0.8434669924,8.8060529827,-15.08885916
C,0,-1.6109652966,8.1956396117,-17.0189269215
H,0,-2.647771742,8.1486318448,-16.6892230729
C,0,-1.2780606976,7.8943919132,-18.3439886601
H,0,-2.0615246128,7.6073016974,-19.0480725484
C,0,0.0477269121,7.9685478198,-18.7612921645

H,0,0.3007587432,7.7515554944,-19.799471832
C,0,1.0815633408,8.3318895675,-17.8757845306
C,0,2.4656505938,8.3497278728,-18.3455332161
H,0,2.6728379065,7.699681525,-19.2007956397
C,0,3.4843600004,9.1350269226,-17.9216506496
H,0,4.4377325493,9.0498005585,-18.4514865275
C,0,3.3880473972,10.2265523998,-16.9596993265
C,0,4.1900545076,11.3718055975,-17.128348134
H,0,4.9472804314,11.3734930389,-17.9161254567
C,0,4.0102995882,12.5001470868,-16.3311775439
H,0,4.6451250769,13.3767476624,-16.4772111721
C,0,3.0021594242,12.5144010025,-15.361976336
H,0,2.8375076396,13.39784774,-14.7418160105
C,0,2.2170314446,11.3783916871,-15.162476106
H,0,1.4378735865,11.3745160335,-14.4069121164
C,0,2.4403419749,10.2260937278,-15.91479983
P,0,0.145068158,5.3967007375,-13.6773505177
O,0,-0.7704895392,6.7415453039,-13.5417623197
O,0,-0.9227625184,4.3056162403,-14.3171891672
N,0,0.2652600216,4.7704776313,-12.0946524043
C,0,-2.008784348,6.6984808855,-12.902387274
C,0,-2.126625625,7.3880321874,-11.6774394886
H,0,-1.2616757468,7.9288866541,-11.2974516147
C,0,-3.3208721077,7.3478156965,-10.996865819
H,0,-3.4332109272,7.8844022665,-10.0519792678
C,0,-4.412184108,6.5801602908,-11.4883808577
C,0,-5.6244897339,6.4646954663,-10.7541081794
H,0,-5.7128525004,6.998131093,-9.8045742089
C,0,-6.662432395,5.6875832256,-11.2192317974
H,0,-7.5868860099,5.6022929663,-10.6441635635
C,0,-6.5247498148,4.9846350207,-12.4406719697
H,0,-7.3409482305,4.3507215459,-12.7942304433
C,0,-5.3686694865,5.0883375272,-13.1850046946
H,0,-5.2750104891,4.5364106966,-14.1199219877
C,0,-4.2845546254,5.8971326,-12.7447804883
C,0,-3.0649641979,6.0284235586,-13.4973531
C,0,-2.935138286,5.5106986464,-14.8848663328
C,0,-3.8543821496,5.9305726688,-15.9131666101
C,0,-4.8443844788,6.9295893594,-15.691346952
H,0,-4.9314628739,7.3863955703,-14.7072836376
C,0,-5.6773207235,7.3473916973,-16.7082594625
H,0,-6.4195211438,8.1243484879,-16.5126281347
C,0,-5.5763383844,6.7850805527,-18.0035199012
H,0,-6.2475680727,7.1203510757,-18.796932279
C,0,-4.6217502351,5.8249189007,-18.2567255811
H,0,-4.5213118471,5.3913593658,-19.2549393449
C,0,-3.73513481,5.3878266681,-17.2352779306
C,0,-2.7025176955,4.4540005104,-17.5106873676
H,0,-2.6170412391,4.0329485546,-18.5138869392
C,0,-1.7901377803,4.1133986502,-16.5435374748
H,0,-0.9798552026,3.4220321269,-16.7659108765
C,0,-1.89335696,4.6674038491,-15.245809392
C,0,0.9494182569,5.6237885721,-11.1406062953
C,0,0.2601748954,6.0539197823,-10.0061260101
H,0,-0.7762693765,5.7426865334,-9.8781726379
C,0,0.9000313843,6.8602093218,-9.0634128598
H,0,0.358431326,7.1980483802,-8.1772372372
C,0,2.2375141769,7.222606081,-9.2557377021

H,0,2.7518677196,7.8511380954,-8.5266739641
C,0,2.9278171392,6.7749532045,-10.3799534642
H,0,3.9748854889,7.040301308,-10.5149715738
C,0,2.3013573464,5.9689452426,-11.3428212237
C,0,3.0812547269,5.4875950235,-12.498471424
H,0,4.0551945296,5.963101913,-12.6181407255
C,0,2.9671612378,4.1718713976,-13.0190581435
H,0,3.8614643275,3.7752058201,-13.5039761423
C,0,2.0855420392,3.1409728721,-12.4313050996
C,0,2.5866625938,1.8480112817,-12.2107397746
H,0,3.6079709887,1.6159197019,-12.517452294
C,0,1.8005330412,0.8658953201,-11.6113446178
H,0,2.2181043591,-0.1284522214,-11.4417236787
C,0,0.4784693679,1.1421747094,-11.2492231027
H,0,-0.1437764288,0.3674752278,-10.7961718205
C,0,-0.0399323075,2.4222536134,-11.455053579
H,0,-1.0597304301,2.680687953,-11.1661808985
C,0,0.765209858,3.4155044565,-12.0113975658
H,0,4.8605313598,1.5618292148,-15.7884814319
H,0,3.744917486,3.6178969555,-16.2710909692
C,0,1.856059032,4.0606429452,-19.5297619327
C,0,1.0677370647,2.8688271209,-19.3696804493
C,0,-0.2081743745,2.7746063531,-19.9878426532
C,0,-0.6619434604,3.8462960072,-20.7253403408
C,0,0.134309982,5.0194678753,-20.8722926488
C,0,1.3815240514,5.1465147582,-20.2888152943
C,0,2.9942156982,2.6762081286,-18.1510216432
C,0,1.8171966042,1.9694010743,-18.5929359693
H,0,-0.8079262203,1.8688714653,-19.8783673296
H,0,-1.6360714605,3.8035492003,-21.2158341238
H,0,-0.2564494759,5.8440028006,-21.4728454988
H,0,1.9813754806,6.0463164737,-20.4190297847
H,0,3.6947757771,4.6301283154,-18.6782815824
H,0,3.9415101622,2.1453032109,-18.0384296389
N,0,3.0319535391,3.8883298447,-18.8788041267
C,0,1.4597869557,0.5657519275,-18.2674117407
H,0,1.1910165421,0.0239839351,-19.1890506622
H,0,0.5796672261,0.5278162764,-17.6040821308
H,0,2.2754026676,0.0312975185,-17.7672621913

TS-2-*endo-Si*-(*ap*)
Opt @ B3LYP-D3(BJ)/SDD/def2-SVP [Gaussian16]
SCF Done: E(RB3LYP) = -5024.50283521 a.u.
Zero-point correction = 1.254299 Hartree/Particle
Thermal correction to Gibbs Free Energy = 1.144260 Hartree/Particle
Imaginary Frequency = -404.8492 cm^{-1}
SP @ RI-B3LYP-D3(BJ)/def2-TZVPP in DCM (SMD) [ORCA 4.1]
FINAL SINGLE POINT ENERGY = -5026.533898150989 a.u.

Ir,0,2.1772555486,5.4690078012,-14.4797519736
Cl,0,4.5379356183,5.5731022794,-15.2594965762
C,0,1.4840263377,5.1914764173,-16.4784662887
H,0,2.2191761396,5.5687732633,-17.1904247439
H,0,0.4665421295,5.5323965392,-16.6726661893
C,0,1.6999549709,3.8584428815,-15.9772636643
H,0,0.8558903017,3.2752907417,-15.6072750796
C,0,2.8877386598,3.112422512,-16.3577869524

C,0,3.2652946555,1.8731691881,-15.6302106816
C,0,2.342367671,0.8446866588,-15.3789923879
H,0,1.2946040668,0.9723106271,-15.6618309324
C,0,2.7533882948,-0.3485878705,-14.7872272666
H,0,2.0260204489,-1.1424165618,-14.6058814948
C,0,4.0908293324,-0.5248421129,-14.416162955
H,0,4.4120552401,-1.4604305988,-13.9532241956
C,0,5.0130668944,0.5040104038,-14.6315042591
H,0,6.054285528,0.3793504457,-14.325744794
C,0,4.6056739152,1.6929402796,-15.242937225
P,0,2.3420460021,7.7864632217,-14.3114142976
O,0,3.8050827986,8.4889835,-14.0694667121
O,0,1.5637337649,8.2596039778,-12.9212528735
N,0,1.8892802732,8.7974018387,-15.5943443445
C,0,4.4351339591,8.6312432003,-12.8523381616
C,0,5.6498518383,7.9260387535,-12.6885575966
H,0,5.9868824588,7.2975123174,-13.5131588951
C,0,6.3514719186,8.0493786419,-11.5136999473
H,0,7.2973350532,7.5197798243,-11.3785949797
C,0,5.8459372247,8.8402191796,-10.4449720609
C,0,6.5256642168,8.9097793089,-9.1979974106
H,0,7.4745353991,8.3783380182,-9.091761991
C,0,5.997039774,9.615717626,-8.1398520258
H,0,6.5263163587,9.6585849107,-7.1855941298
C,0,4.7522638498,10.2731557219,-8.2882037442
H,0,4.3197588148,10.8104330912,-7.4411008723
C,0,4.0759804575,10.2376058652,-9.4898162915
H,0,3.1123307005,10.7361094694,-9.5775535197
C,0,4.6052670207,9.5428631636,-10.6136704193
C,0,3.9162167222,9.4731210537,-11.8775614522
C,0,2.6590601107,10.2342987237,-12.1058724295
C,0,2.587418038,11.658143897,-11.9125502291
C,0,3.7466208184,12.4536638274,-11.6949466779
H,0,4.7226908049,11.9712723397,-11.6658083898
C,0,3.6531677165,13.8209597288,-11.5456484256
H,0,4.559399637,14.4110667612,-11.3921820416
C,0,2.3949079245,14.4691809936,-11.596203719
H,0,2.3347842139,15.5519469619,-11.4669391615
C,0,1.2537069256,13.7322422155,-11.8247139326
H,0,0.2792023144,14.2227162523,-11.8870337004
C,0,1.3182009251,12.3230258157,-12.0062615136
C,0,0.1565346908,11.5677695165,-12.3256370616
H,0,-0.8086220008,12.0762850096,-12.3811608577
C,0,0.2554438104,10.2282742862,-12.6215593938
H,0,-0.6079226627,9.6425282406,-12.9358916495
C,0,1.5107310861,9.5843046537,-12.5366210248
C,0,0.982377667,8.3687372781,-16.6137780118
C,0,-0.3905296541,8.3234549712,-16.3548438459
H,0,-0.7460568322,8.6218095741,-15.3692503806
C,0,-1.2804426213,7.888311784,-17.3363035517
H,0,-2.3490395659,7.8543233096,-17.1284681301
C,0,-0.7930586811,7.5058108907,-18.591220381
H,0,-1.4884577125,7.1715193097,-19.3640993413
C,0,0.5741972049,7.5569700759,-18.8519930454
H,0,0.9521764017,7.2716732684,-19.8370513339
C,0,1.4954856428,7.985589241,-17.8749443934
C,0,2.9271653302,7.9627145692,-18.1701618432
H,0,3.2375522103,7.2295055363,-18.9192205288

C,0,3.8864548938,8.7868826894,-17.6883410867
H,0,4.9001739023,8.6572933714,-18.0785173647
C,0,3.6739847434,9.9488621662,-16.8329117928
C,0,4.4878428074,11.0873870739,-16.986981583
H,0,5.3273914282,11.0440167061,-17.6850413219
C,0,4.221578952,12.263028305,-16.2880702804
H,0,4.8674554038,13.1336606259,-16.4210761136
C,0,3.1172888623,12.3305135646,-15.4325909774
H,0,2.8894454386,13.2492594845,-14.8883829577
C,0,2.3161687626,11.2034267187,-15.2461268191
H,0,1.4645601751,11.2380839626,-14.5732815089
C,0,2.6170277108,10.0097672595,-15.9001544406
P,0,0.1473813192,5.339883132,-13.5852791493
O,0,-0.726561523,6.7156271524,-13.5443688799
O,0,-0.9390471264,4.2465862023,-14.1858225989
N,0,0.2140769596,4.7637834497,-11.9796259343
C,0,-1.9897218657,6.7462979279,-12.9550369751
C,0,-2.1258353453,7.4766218257,-11.7558178243
H,0,-1.2517010442,7.9818339456,-11.3488476736
C,0,-3.3517644187,7.5216062185,-11.1348058739
H,0,-3.4793671813,8.0910339604,-10.2113219992
C,0,-4.4603480683,6.8029587762,-11.6605857717
C,0,-5.712205514,6.7826719684,-10.9864803955
H,0,-5.8160423592,7.3538655124,-10.0607783853
C,0,-6.769372323,6.0493553267,-11.4784791313
H,0,-7.7247102883,6.0372671929,-10.949552007
C,0,-6.6115594717,5.2953715346,-12.6663403382
H,0,-7.4440560967,4.6951184173,-13.0400431404
C,0,-5.4156854753,5.3062949847,-13.3528907349
H,0,-5.3089252061,4.714085144,-14.2610586326
C,0,-4.309901164,6.0695830771,-12.8859688135
C,0,-3.0474186761,6.1059825352,-13.5773628502
C,0,-2.871264677,5.4950852403,-14.9212047375
C,0,-3.7175494085,5.8773812547,-16.0233214027
C,0,-4.6395915163,6.9579229629,-15.9338839838
H,0,-4.7285474756,7.5068054171,-14.9977558936
C,0,-5.4011286511,7.3367591649,-17.0199059648
H,0,-6.0910558901,8.1784165075,-16.9282052679
C,0,-5.2935717326,6.6500393995,-18.253090161
H,0,-5.9082420302,6.9559628147,-19.1022120818
C,0,-4.404992258,5.6044291522,-18.3773123941
H,0,-4.3026994016,5.0729472001,-19.3267203152
C,0,-3.5907456409,5.2038538766,-17.2834381485
C,0,-2.6273643863,4.1692276994,-17.4224868694
H,0,-2.5593986139,3.6309990188,-18.3706764424
C,0,-1.771034906,3.8709769821,-16.3919692701
H,0,-1.0121055338,3.0963450563,-16.4891955392
C,0,-1.8684535847,4.5668016273,-15.1647806607
C,0,0.9022871718,5.6104602304,-11.0282086435
C,0,0.1929293241,6.1119843165,-9.9361511612
H,0,-0.8591615608,5.8466097183,-9.8333778275
C,0,0.8315686466,6.9327623324,-9.0050375781
H,0,0.2744381454,7.3259995909,-8.1519565026
C,0,2.1862679016,7.2420142699,-9.1677990221
H,0,2.6981768591,7.8844304478,-8.4493111016
C,0,2.8948618045,6.7274019242,-10.2510404441
H,0,3.9532690784,6.955837104,-10.3656039588
C,0,2.270819069,5.9020449117,-11.1997229034

C,0,3.0668496629,5.3559007523,-12.3117894937
H,0,4.0556941218,5.8005257844,-12.4269478017
C,0,2.9186405891,4.0469860187,-12.825546029
H,0,3.8063499821,3.61813353,-13.2933184208
C,0,1.9554404769,3.0490590309,-12.3175015178
C,0,2.3487358685,1.7045201727,-12.2149639118
H,0,3.3561045317,1.42450556,-12.5241592704
C,0,1.4759803506,0.7361838233,-11.7216626074
H,0,1.8093806425,-0.3010701246,-11.6466035858
C,0,0.1786583214,1.0860763663,-11.3333012333
H,0,-0.5092179406,0.3259465691,-10.9571276778
C,0,-0.2349083155,2.4162180759,-11.4286206037
H,0,-1.2407722604,2.7253591629,-11.1413085501
C,0,0.6485516556,3.3862231927,-11.8995088174
H,0,5.3171634983,2.5067616929,-15.4046387888
H,0,3.7386892926,3.7673414308,-16.5521664615
C,0,4.6124987878,3.239887165,-19.2637207089
C,0,3.4751310635,4.0229174236,-19.6671292922
C,0,3.6550976229,5.1224135201,-20.5472835546
C,0,4.9323962613,5.4316403826,-20.9634615218
C,0,6.050263598,4.6637422531,-20.5248020552
C,0,5.9146675816,3.5733503255,-19.6825468174
C,0,2.8038937491,2.4197919636,-18.1785256175
C,0,2.3419082729,3.4683511962,-19.0429345502
H,0,2.7959061855,5.7049839836,-20.8838026569
H,0,5.1008852361,6.2739844321,-21.6366886995
H,0,7.0472788774,4.9447031579,-20.8715370948
H,0,6.7803807569,2.9920699523,-19.3613006422
H,0,4.774203414,1.6265395754,-17.9194392203
H,0,2.1950414796,1.533353388,-17.9962787534
N,0,4.1736589853,2.2261049303,-18.4749042825
C,0,0.9175853132,3.8382174898,-19.2413053511
H,0,0.7807357476,4.9257461077,-19.3093011192
H,0,0.2873483647,3.4663394737,-18.4247991543
H,0,0.5463588388,3.3946089688,-20.1825058241

TS-2-*exo*-*Re*-(+*sc*)
Opt @ B3LYP-D3(BJ)/SDD/def2-SVP [Gaussian16]
SCF Done: E(RB3LYP) = -5024.49535258 a.u.
Zero-point correction = 1.254566 Hartree/Particle
Thermal correction to Gibbs Free Energy = 1.144334 Hartree/Particle
Imaginary Frequency = -367.5866 cm^{-1}
SP @ RI-B3LYP-D3(BJ)/def2-TZVPP in DCM (SMD) [ORCA 4.1]
FINAL SINGLE POINT ENERGY = -5026.528502500561 a.u.

Ir,0,3.5216325463,5.8992313526,-14.5899357519
Cl,0,5.5443795707,5.7153016586,-16.0067388021
C,0,2.3070427144,5.4898620016,-16.3187106493
H,0,2.7433512051,5.9693123483,-17.1938922993
H,0,1.237541921,5.6758047299,-16.1940559423
C,0,2.8913064642,4.2435242426,-15.9102097167
H,0,3.8179406364,4.0048292721,-16.4319569531
C,0,2.1925755843,3.0650562962,-15.3903237499
H,0,1.3426582381,3.2765053798,-14.7361576362
C,0,3.0020990338,1.9039535493,-14.9379900557
C,0,4.3366352808,1.7157566213,-15.3406816588
H,0,4.8544701158,2.4852210009,-15.9140270324

C,0,5.0281471935,0.5536592462,-14.9950777147
H,0,6.0648088923,0.4282932368,-15.3151457701
C,0,4.399648419,-0.4447419358,-14.2464496729
H,0,4.9390324691,-1.358130078,-13.9862267144
C,0,3.0824403784,-0.2590220047,-13.8168854409
H,0,2.5905322454,-1.0205451287,-13.2085513144
C,0,2.3988632218,0.9057456546,-14.1540824746
P,0,3.7921489124,8.22299496,-14.7688557885
O,0,5.2835173158,8.8256910966,-15.0907229516
O,0,3.5219291079,8.895184518,-13.2705117459
N,0,2.989238817,9.1266393952,-15.9565485996
C,0,6.2823544556,8.9948622685,-14.1531206754
C,0,7.4383533646,8.1952466007,-14.307213147
H,0,7.4571939708,7.4816990357,-15.1305963197
C,0,8.4785727621,8.3308847565,-13.4197516766
H,0,9.3810820715,7.7260853358,-13.534092609
C,0,8.3871493199,9.2319054659,-12.3230866664
C,0,9.4232624063,9.3181910113,-11.3530890868
H,0,10.3192332735,8.7083600231,-11.4924099391
C,0,9.2986065827,10.1372537279,-10.2525404301
H,0,10.0991767935,10.1915558055,-9.5117567817
C,0,8.1183521309,10.8985040322,-10.0748410276
H,0,8.008902622,11.5282442988,-9.1889449744
C,0,7.102478886,10.8498174195,-11.006582305
H,0,6.1966189332,11.4316078924,-10.8446046431
C,0,7.2078414219,10.0357023888,-12.1693191805
C,0,6.1562223741,9.9464333593,-13.1498710071
C,0,4.9506049477,10.8112033378,-13.0584531977
C,0,5.0478155203,12.2456733161,-13.0105294904
C,0,6.2747037594,12.9319102954,-13.2305709134
H,0,7.1755282769,12.3552714235,-13.4360713499
C,0,6.3327480224,14.3091714697,-13.2088412747
H,0,7.2845339291,14.8128008559,-13.3918784779
C,0,5.169820855,15.0779395301,-12.9583986887
H,0,5.2325452609,16.1680025319,-12.9354043289
C,0,3.9611923613,14.4472364818,-12.7615891035
H,0,3.0526722366,15.029264599,-12.5886125211
C,0,3.8628175503,13.0288270109,-12.7993046796
C,0,2.6070784817,12.3738953305,-12.6743395919
H,0,1.7109274918,12.9734603931,-12.4995143254
C,0,2.5126151615,11.0102028556,-12.8262115547
H,0,1.5534993565,10.494282563,-12.7912911913
C,0,3.6824519808,10.2477022409,-13.0456913756
C,0,1.7010356605,8.7447294665,-16.4501127265
C,0,0.5704544913,8.9242030189,-15.6482200534
H,0,0.6953565961,9.370617621,-14.6635996994
C,0,-0.6871992299,8.5268397556,-16.0982186781
H,0,-1.5635713079,8.672856476,-15.4680791536
C,0,-0.81536311,7.9412187778,-17.3622610048
H,0,-1.7982148476,7.6235006415,-17.7157370972
C,0,0.3071279912,7.7743701097,-18.1691428172
H,0,0.2047315853,7.3239797902,-19.1597494152
C,0,1.5866717932,8.1849684774,-17.7438926397
C,0,2.7357512316,7.9785299237,-18.6230631448
H,0,2.61108113,7.1859806197,-19.3678571878
C,0,3.8770902282,8.7050790551,-18.6891223285
H,0,4.5936806784,8.4375835927,-19.4712574034
C,0,4.1743700463,9.9293710598,-17.9539951488

C,0,4.9528978975,10.9304271787,-18.5680149844
H,0,5.3983814179,10.7248671101,-19.5443305338
C,0,5.1369456148,12.1726753054,-17.9657096406
H,0,5.7467897903,12.9321893058,-18.4597127779
C,0,4.5228597428,12.4500216815,-16.7398388648
H,0,4.643792473,13.4249876411,-16.2631601208
C,0,3.7696256394,11.462969794,-16.104589007
H,0,3.29564897,11.6633622794,-15.1491759301
C,0,3.6357472343,10.1985063467,-16.6780254784
P,0,1.8330127611,6.0030632781,-13.1526601076
O,0,1.1191681443,7.4576176033,-12.9406057503
O,0,0.5192588712,5.0060117269,-13.3362964081
N,0,2.3337443819,5.527275585,-11.5880913543
C,0,0.181185799,7.6554032744,-11.9302552083
C,0,0.5439768957,8.5109217372,-10.8691332038
H,0,1.520920886,8.9893663222,-10.8945467961
C,0,-0.3362902096,8.7016874964,-9.8299495678
H,0,-0.0747368494,9.3679742669,-9.0047424283
C,0,-1.574503297,8.0040956792,-9.7882688329
C,0,-2.453466759,8.1216621941,-8.6764740953
H,0,-2.1676687999,8.779298415,-7.8519685056
C,0,-3.6339938082,7.4133173823,-8.6294810998
H,0,-4.2984639266,7.5074606647,-7.7679660922
C,0,-3.9811247216,6.5488661905,-9.696080206
H,0,-4.9070744866,5.9718177817,-9.6443331735
C,0,-3.159454024,6.425584301,-10.7964756809
H,0,-3.4369944542,5.7532172423,-11.6081075421
C,0,-1.9418773423,7.1552709756,-10.8861973809
C,0,-1.0620913309,7.0508595321,-12.0196920237
C,0,-1.4730065161,6.388694307,-13.2841222515
C,0,-2.6719205427,6.810373809,-13.9699019909
C,0,-3.4461407517,7.9249053708,-13.5391813692
H,0,-3.137394955,8.4820219729,-12.6570894357
C,0,-4.5675523311,8.3279736977,-14.2340087927
H,0,-5.1343775064,9.194074973,-13.8856340067
C,0,-4.9869466519,7.6376584703,-15.3960278879
H,0,-5.881274618,7.9633152149,-15.9312198906
C,0,-4.2507148278,6.5678433109,-15.854581903
H,0,-4.5477726467,6.0379080308,-16.7630056293
C,0,-3.0779076478,6.1429079138,-15.1733370658
C,0,-2.2652037871,5.1031306857,-15.6945288012
H,0,-2.5739010787,4.5962624428,-16.6118601694
C,0,-1.083821312,4.7722860303,-15.080924351
H,0,-0.4361428524,4.0057718642,-15.4893227033
C,0,-0.6849320878,5.4208634097,-13.890397597
C,0,3.3011853647,6.4016068389,-10.9507188358
C,0,2.9808078874,6.9819443273,-9.7226653973
H,0,2.0113657112,6.7590387353,-9.278471127
C,0,3.8907587685,7.8324292168,-9.0927063894
H,0,3.6357433953,8.2891575693,-8.133958027
C,0,5.126892379,8.09330246,-9.6942241956
H,0,5.8460506037,8.7619931081,-9.2174882951
C,0,5.4553960674,7.4881898339,-10.9052484235
H,0,6.4295658554,7.6693538454,-11.3562326661
C,0,4.5560215963,6.6261083215,-11.5519478698
C,0,4.9706897501,5.9529936629,-12.7954408146
H,0,5.8989270713,6.328741501,-13.2267550228
C,0,4.6587773951,4.599082806,-13.0899896283

H,0,5.3719081282,4.0779127396,-13.7315980006
C,0,3.8914658149,3.7131667067,-12.1885557076
C,0,4.3312738086,2.3967461685,-11.9733501656
H,0,5.2222915794,2.0439781765,-12.4945081829
C,0,3.6472018498,1.5421374096,-11.110516533
H,0,4.0170712306,0.5269673947,-10.9552051905
C,0,2.4855576855,1.9725160571,-10.4622572489
H,0,1.9406596599,1.2979274911,-9.7983896609
C,0,2.0331689678,3.2794398581,-10.6552167094
H,0,1.1427830485,3.6584951701,-10.1513094396
C,0,2.7444688904,4.1450722834,-11.485706917
H,0,1.3776506837,1.0516944105,-13.7976235809
C,0,2.3413295546,0.6285254016,-17.7289504925
C,0,1.1931981988,0.1633159397,-16.9902056679
C,0,1.0033554042,-1.2324924183,-16.7954465444
C,0,1.9421777798,-2.1007348079,-17.3024811926
C,0,3.0813073432,-1.6167361072,-18.0145140592
C,0,3.2977203458,-0.270098547,-18.2396157845
C,0,1.2640506663,2.4516616941,-16.929237639
C,0,0.4819166011,1.2929952004,-16.5634022786
H,0,0.1365429979,-1.6008390595,-16.2441165877
H,0,1.8271550272,-3.1767944416,-17.1615281518
H,0,3.8058220795,-2.3405096003,-18.3947914339
H,0,4.1755182732,0.0844710391,-18.7814781668
H,0,3.0452745508,2.5680820025,-18.1147896599
H,0,0.7820218191,3.3892166574,-17.2124592705
N,0,2.2891712922,1.9783452938,-17.7866932077
C,0,-0.7860591263,1.313748379,-15.7864670276
H,0,-1.1977465586,0.3034294957,-15.6653672562
H,0,-0.63441813,1.7398360807,-14.780567885
H,0,-1.5477514893,1.9406824545,-16.2759045565

TS-2-*exo*-*Re*-(-*sc*)
Opt @ B3LYP-D3(BJ)/SDD/def2-SVP [Gaussian16]
SCF Done: E(RB3LYP) = -5024.49691964 a.u.
Zero-point correction = 1.254741 Hartree/Particle
Thermal correction to Gibbs Free Energy = 1.145166 Hartree/Particle
Imaginary Frequency = -376.7481 cm^{-1}
SP @ RI-B3LYP-D3(BJ)/def2-TZVPP in DCM (SMD) [ORCA 4.1]
FINAL SINGLE POINT ENERGY = -5026.525993983302 a.u.

Ir,0,3.5703152684,5.8934503684,-14.4392808716
Cl,0,5.6166098215,5.6863613752,-15.8129547448
C,0,2.332717938,5.5870776051,-16.1811783289
H,0,2.73524004,6.1246012596,-17.0377771833
H,0,1.2689292535,5.764766462,-16.0036075456
C,0,2.9257089457,4.317944265,-15.8852129175
H,0,3.8242425291,4.1023160508,-16.4622632549
C,0,2.2711953545,3.142829779,-15.3347632015
H,0,1.4398154909,3.3640283081,-14.6623955976
C,0,3.1034661602,2.0101697908,-14.8402990678
C,0,4.4837122218,1.9284075032,-15.0928842244
H,0,5.0016154961,2.752653135,-15.5852313857
C,0,5.2088933434,0.803123177,-14.6955663188
H,0,6.2814696415,0.7559581167,-14.8967987403
C,0,4.5709856178,-0.2562872595,-14.0444827346
H,0,5.1396302831,-1.1390680975,-13.7441587961

C,0,3.2039980751,-0.1703692208,-13.760098363
H,0,2.7027898103,-0.9791057774,-13.2242461107
C,0,2.4832021105,0.9575900789,-14.1471523822
P,0,3.8547212225,8.2250113586,-14.6490773516
O,0,5.3584594736,8.7922769789,-14.9730048072
O,0,3.5775825618,8.94096655,-13.1682865354
N,0,3.0852415465,9.1266985122,-15.8675887809
C,0,6.3616121859,8.9600982256,-14.039040626
C,0,7.506600454,8.1429411518,-14.1808802778
H,0,7.5143600927,7.4132267843,-14.989670408
C,0,8.5519975316,8.2836056543,-13.3002569412
H,0,9.4462381519,7.6651551406,-13.4061801541
C,0,8.4779886854,9.2093239306,-12.223185012
C,0,9.5214028236,9.3053753652,-11.2619312721
H,0,10.4082194704,8.6802666153,-11.3916503931
C,0,9.4151341578,10.153586039,-10.181784045
H,0,10.2212645325,10.2158189669,-9.4477082997
C,0,8.2464071857,10.9350498456,-10.0161063419
H,0,8.1513444926,11.5884937859,-9.145871259
C,0,7.223458397,10.8758869096,-10.9394028064
H,0,6.3263392616,11.473244407,-10.7857689059
C,0,7.3100636862,10.0314288803,-12.0818302881
C,0,6.2526264452,9.9334844239,-13.0553416619
C,0,5.0648981221,10.8234793556,-12.9854275745
C,0,5.2001447847,12.2566014758,-12.9722188248
C,0,6.44517152,12.9044016153,-13.2081000342
H,0,7.3303358294,12.2996491195,-13.3992878802
C,0,6.5406749477,14.2797068583,-13.2196099945
H,0,7.5060323171,14.7523270177,-13.4138883774
C,0,5.3993413632,15.0860003285,-12.9887421938
H,0,5.4919575824,16.1741568869,-12.9911438521
C,0,4.1737499054,14.4932061505,-12.780293727
H,0,3.2808185038,15.1033593307,-12.6239659579
C,0,4.0372008826,13.0774623966,-12.7852655659
C,0,2.7639513272,12.4598739282,-12.6534539723
H,0,1.8829598309,13.0865358271,-12.4978156868
C,0,2.6339683575,11.0963657837,-12.7752294678
H,0,1.6592476693,10.6121437743,-12.7325120977
C,0,3.7816158441,10.2937082298,-12.9693929662
C,0,1.7532701534,8.8372372945,-16.2977503834
C,0,0.6805107206,9.0621742791,-15.4307373321
H,0,0.8872151461,9.4649481188,-14.4426790073
C,0,-0.6235158341,8.7640215519,-15.8190966248
H,0,-1.4511375801,8.9317797599,-15.1313235752
C,0,-0.8655658672,8.2622881686,-17.1021296923
H,0,-1.8881410776,8.0412552831,-17.4166176195
C,0,0.1995234244,8.0633426456,-17.9778874183
H,0,0.0109894073,7.7049225244,-18.991344407
C,0,1.5305712096,8.3350783317,-17.6016080636
C,0,2.6167944604,8.0431392229,-18.5361168771
H,0,2.3942720627,7.2648731658,-19.2718302127
C,0,3.8161769461,8.6635296594,-18.6399668039
H,0,4.4785942254,8.3393243806,-19.4480954791
C,0,4.2374469326,9.8560258545,-17.9130067633
C,0,5.0546128935,10.804280316,-18.5593944302
H,0,5.4498160156,10.5692948507,-19.5506284502
C,0,5.3354112867,12.0348667432,-17.9705547752
H,0,5.9733814285,12.7538951937,-18.4889657208

C,0,4.7781227818,12.3543574579,-16.7279914256
H,0,4.9697583697,13.3234174408,-16.262612105
C,0,3.9898979502,11.4165810871,-16.0613661952
H,0,3.5583774756,11.6517823195,-15.094643354
C,0,3.7655886595,10.1575873214,-16.6183278382
P,0,1.8691679617,6.0020754017,-13.0116587367
O,0,1.120122064,7.4461742896,-12.937108444
O,0,0.5863706256,4.9394379089,-13.0756717415
N,0,2.3633142693,5.6420890705,-11.4187617
C,0,0.1451756007,7.7285421098,-11.9889477162
C,0,0.4748987191,8.6600728693,-10.9821828482
H,0,1.4675987101,9.1055029796,-10.9883677728
C,0,-0.4577706782,8.9625039635,-10.0188869949
H,0,-0.2249334612,9.6898855989,-9.2378847773
C,0,-1.7188298988,8.3060169474,-9.9964470197
C,0,-2.6572794038,8.5534263775,-8.9572190512
H,0,-2.4017514091,9.2851573958,-8.1870471858
C,0,-3.8559255015,7.8765753049,-8.9091761636
H,0,-4.5666625893,8.0707134885,-8.1031787936
C,0,-4.1583087424,6.9114172353,-9.8993464603
H,0,-5.0964980223,6.354971509,-9.8430290281
C,0,-3.277701439,6.6609044121,-10.9307607985
H,0,-3.5252115597,5.9070521271,-11.6768605401
C,0,-2.0423666488,7.3596175868,-11.0271680454
C,0,-1.0972864126,7.1294762318,-12.0914214592
C,0,-1.4387125247,6.2747094225,-13.2586593572
C,0,-2.6280065039,6.5370526661,-14.0339443033
C,0,-3.3872525237,7.7297843105,-13.872944348
H,0,-3.0726905909,8.4655186581,-13.1342371594
C,0,-4.4985716115,7.9796559308,-14.6506893729
H,0,-5.0531624585,8.9107013592,-14.5153693659
C,0,-4.926730479,7.0444067497,-15.623176572
H,0,-5.8144177999,7.2510883516,-16.2245027247
C,0,-4.2162887167,5.8784908994,-15.8071018684
H,0,-4.5318845191,5.147962219,-16.5560763867
C,0,-3.0545207266,5.6040767833,-15.0358028206
C,0,-2.306088514,4.411489119,-15.2256610601
H,0,-2.6812562175,3.6496495003,-15.9139639322
C,0,-1.123339904,4.2228857711,-14.5513026808
H,0,-0.5528622932,3.3009718197,-14.6532005677
C,0,-0.6501989272,5.1924515322,-13.6314692695
C,0,3.3405586716,6.5370753325,-10.8351317426
C,0,3.0136583819,7.2239148002,-9.6653803198
H,0,2.0263703633,7.0710515105,-9.2298721587
C,0,3.9440746718,8.0827679083,-9.0777660659
H,0,3.6875950136,8.6211782857,-8.1628075252
C,0,5.2055319079,8.2416890963,-9.661592936
H,0,5.9435217104,8.9088657823,-9.2129501587
C,0,5.5322239237,7.5425610531,-10.8216720463
H,0,6.5210996865,7.6544455931,-11.2631896305
C,0,4.6092002109,6.6785552048,-11.4306698584
C,0,5.001033937,5.9383713665,-12.6436007592
H,0,5.9438851922,6.2654008326,-13.0832976844
C,0,4.6455470081,4.5841686647,-12.8945380173
H,0,5.3486973213,4.019875351,-13.5085228169
C,0,3.8541858424,3.7551163961,-11.9581180237
C,0,4.2320802976,2.4235797139,-11.7186866877
H,0,5.0987089648,2.0179801473,-12.2405206618

C,0,3.5181167808,1.6202179848,-10.8314616172
H,0,3.8368997446,0.589286971,-10.6647109421
C,0,2.3953207104,2.1239164932,-10.1671494097
H,0,1.8286823648,1.4912713329,-9.4806741216
C,0,2.007671287,3.4481366881,-10.3790993566
H,0,1.1435788918,3.8806304898,-9.8726621405
C,0,2.7414748504,4.2556500615,-11.2469559299
H,0,1.4196473769,1.0252702605,-13.9015831099
C,0,0.9021968537,3.7915458987,-18.4398265034
C,0,1.97991413,2.9114186135,-18.7989629915
C,0,2.6713497924,3.1097487246,-20.0256923744
C,0,2.2819735855,4.1516804717,-20.8381979286
C,0,1.2017653408,5.0065615072,-20.4657842899
C,0,0.5054796159,4.8465443716,-19.2814966877
C,0,1.2097020414,2.3670614933,-16.7108974278
C,0,2.1097884384,1.9641506593,-17.770574173
H,0,3.4933857733,2.4500150279,-20.3093682737
H,0,2.7962252226,4.3315627596,-21.7837761666
H,0,0.9160828776,5.8132873621,-21.1451403579
H,0,-0.3164055669,5.5039693164,-19.0027621653
H,0,-0.2608252024,3.9343899246,-16.6982812814
H,0,0.7057712349,1.610217247,-16.105611667
N,0,0.3957077653,3.3904541447,-17.2507015566
C,0,3.0437379615,0.811649048,-17.7425125516
H,0,3.0329278497,0.2877218605,-18.7112431772
H,0,4.0775630179,1.1598275656,-17.5747401477
H,0,2.8065708049,0.0980876283,-16.944853368

TS-2-*exo*-*Re*-(*ap*)

Opt @ B3LYP-D3(BJ)/SDD/def2-SVP [Gaussian16]
SCF Done: E(RB3LYP) = -5024.49247119 a.u.
Zero-point correction = 1.254632 Hartree/Particle
Thermal correction to Gibbs Free Energy = 1.145122 Hartree/Particle
Imaginary Frequency = -408.2353 cm^{-1}
SP @ RI-B3LYP-D3(BJ)/def2-TZVPP in DCM (SMD) [ORCA 4.1]
FINAL SINGLE POINT ENERGY = -5026.523081924413 a.u.

Ir,0,3.5713139935,5.8363165439,-14.4394976865
Cl,0,5.6048042038,5.6613714133,-15.8350458562
C,0,2.4630290447,5.4340539128,-16.222057353
H,0,2.9625843376,5.9137677993,-17.0613989835
H,0,1.3873466041,5.6205979167,-16.1653834857
C,0,2.9630255865,4.1609101063,-15.7893839606
H,0,3.9145215246,3.8342533671,-16.2131137682
C,0,2.0923968198,3.1124691749,-15.2882339652
H,0,1.1724124291,3.4877512017,-14.847273686
C,0,2.6336327966,1.9465775926,-14.5402468419
C,0,3.870482814,1.3645730204,-14.8644092645
H,0,4.5206958356,1.838816783,-15.6027774403
C,0,4.2671874166,0.1694963257,-14.2646600514
H,0,5.2269754672,-0.2780088975,-14.532079272
C,0,3.436849338,-0.46026542,-13.3315558078
H,0,3.7445239692,-1.402438762,-12.8729063408
C,0,2.224159597,0.1326477994,-12.970560509
H,0,1.5912629928,-0.3314157317,-12.2116668643
C,0,1.8274296146,1.3300628027,-13.567559028
P,0,3.7818606614,8.1572093483,-14.7217229953

O,0,5.2541213182,8.7908359564,-15.0687715204
O,0,3.4897476895,8.8889143178,-13.2572917297
N,0,2.9583974323,8.9745706682,-15.963484049
C,0,6.2495394467,9.022635596,-14.1394322877
C,0,7.428010428,8.2519532506,-14.267174701
H,0,7.46728934,7.5098438535,-15.0639657533
C,0,8.4652307764,8.45047104,-13.3880337329
H,0,9.384747363,7.8683958563,-13.4829012896
C,0,8.3491066829,9.3875486142,-12.3244675501
C,0,9.3829218206,9.5378204555,-11.3598578563
H,0,10.2954936015,8.9485925232,-11.4783388464
C,0,9.2357643555,10.3923350737,-10.2894350468
H,0,10.0348311967,10.4957568632,-9.5522778427
C,0,8.034774819,11.1263022152,-10.1377187468
H,0,7.9082174461,11.7844514742,-9.2750638618
C,0,7.0200484417,11.0152108463,-11.0653464958
H,0,6.0981913246,11.5766163907,-10.9227171865
C,0,7.1474857962,10.1623529035,-12.1976036043
C,0,6.0979986729,10.0074095661,-13.1723468844
C,0,4.8717411171,10.8451525351,-13.1136481186
C,0,4.9343237001,12.2824149483,-13.1193073459
C,0,6.1445722365,12.989231977,-13.364805947
H,0,7.0591357279,12.4272227409,-13.5488734263
C,0,6.1694692962,14.3673206296,-13.3943416058
H,0,7.1090237959,14.886504427,-13.596088452
C,0,4.9882574475,15.1167593601,-13.1723457995
H,0,5.0249125803,16.2081110476,-13.1895280297
C,0,3.7948798569,14.4650132528,-12.9524222007
H,0,2.87251347,15.0310610276,-12.8011048878
C,0,3.7306165313,13.0441881899,-12.9379227498
C,0,2.4905534579,12.3645266037,-12.7913184022
H,0,1.5797402794,12.9483543916,-12.6407918989
C,0,2.428681938,10.9944285758,-12.8948557284
H,0,1.4808342487,10.4593257145,-12.847342628
C,0,3.6173880228,10.2526788809,-13.0829875345
C,0,1.6857266086,8.5309417224,-16.4427344207
C,0,0.5640039322,8.6188931239,-15.6132737238
H,0,0.6814062256,9.0507857433,-14.6217041087
C,0,-0.6748518563,8.1394610725,-16.0370462066
H,0,-1.5383594785,8.2026087874,-15.3760696487
C,0,-0.8006071763,7.5856037521,-17.3149081151
H,0,-1.7700301149,7.2136962306,-17.6536086278
C,0,0.3094968272,7.5195533765,-18.154131262
H,0,0.2072339289,7.1065857108,-19.1610709694
C,0,1.5764675743,7.9841284419,-17.7443544889
C,0,2.7216673112,7.8134488357,-18.6358980438
H,0,2.612017906,7.0178268104,-19.3796263579
C,0,3.8500452258,8.5592003263,-18.7037611259
H,0,4.5698346718,8.3046908831,-19.4872343419
C,0,4.1189419112,9.7921717668,-17.9739506149
C,0,4.8697207671,10.8081916173,-18.5974880188
H,0,5.3279167651,10.60244696,-19.5678286362
C,0,5.0080830696,12.0650173899,-18.0135141625
H,0,5.5976710588,12.8363500355,-18.5136592885
C,0,4.3707254137,12.3407580894,-16.7996820747
H,0,4.4515667439,13.3270415943,-16.3381152261
C,0,3.6461103322,11.3382566907,-16.1549347364
H,0,3.1545585342,11.5404703056,-15.2094553139

C,0,3.5641910462,10.0590080648,-16.7044649184
P,0,1.8872488443,5.9532627286,-12.9813528072
O,0,1.1800147836,7.4158590917,-12.80910458
O,0,0.5730218947,4.9425682575,-13.0753390691
N,0,2.4257542181,5.5502968319,-11.40597612
C,0,0.306441598,7.6777067161,-11.7576764358
C,0,0.7256698154,8.5980856311,-10.7746803651
H,0,1.6979152838,9.0734216737,-10.882590161
C,0,-0.0966205216,8.8532251037,-9.7023416505
H,0,0.2084140218,9.5695885136,-8.9361545914
C,0,-1.326927833,8.1576955529,-9.5471009332
C,0,-2.1428699752,8.3439477062,-8.3972492158
H,0,-1.8141531077,9.0520108247,-7.6326582742
C,0,-3.3161218412,7.6393831203,-8.2402023025
H,0,-3.9316905626,7.7867159312,-7.3502688371
C,0,-3.7192030759,6.7103036263,-9.2300178123
H,0,-4.6388026721,6.1375904634,-9.0907517696
C,0,-2.9598560972,6.518854161,-10.3649246256
H,0,-3.2799990103,5.7974887164,-11.1165537591
C,0,-1.7514365258,7.2410126221,-10.5672194748
C,0,-0.9352320484,7.0678100013,-11.7394478546
C,0,-1.4193209016,6.3326858533,-12.9352358242
C,0,-2.6617068123,6.7258484961,-13.5587902326
C,0,-3.3808260242,7.8895168852,-13.1630089047
H,0,-2.9943662803,8.5094034439,-12.3564426832
C,0,-4.5471312386,8.2599179242,-13.7998686727
H,0,-5.0698950536,9.1646340628,-13.4820060441
C,0,-5.0697610117,7.4862636877,-14.8635797466
H,0,-5.998814666,7.7876130268,-15.3519224043
C,0,-4.3899640927,6.3657535391,-15.2868558189
H,0,-4.7660998973,5.7677908293,-16.120714411
C,0,-3.1735430451,5.9721458695,-14.6658725297
C,0,-2.4281367918,4.867641565,-15.1507335083
H,0,-2.8224770938,4.2792938656,-15.9780525575
C,0,-1.2021809454,4.5664477581,-14.6146357676
H,0,-0.6227310649,3.7386500935,-15.0093072408
C,0,-0.6843192134,5.3166123785,-13.5331455731
C,0,3.3867360189,6.4613536691,-10.813642306
C,0,3.0826037613,7.0682724585,-9.5943946055
H,0,2.1263941137,6.8424545594,-9.1240285037
C,0,3.9915936166,7.9493116356,-9.0066871825
H,0,3.7493533286,8.4266504886,-8.054691543
C,0,5.2096241552,8.2158590018,-9.6420445374
H,0,5.9265558729,8.9095241933,-9.1989490379
C,0,5.5234990462,7.5831759859,-10.8429010873
H,0,6.485565669,7.7659921625,-11.3185428035
C,0,4.6263464235,6.6865861021,-11.4444610693
C,0,5.0299327784,5.9742034594,-12.6682700839
H,0,5.9462307448,6.3466576753,-13.1271061548
C,0,4.7333779203,4.6047014087,-12.9112707346
H,0,5.4476204656,4.07291779,-13.5435041354
C,0,4.0043099178,3.7426054398,-11.9576402906
C,0,4.4884409793,2.452769254,-11.683142929
H,0,5.374951712,2.0970291995,-12.2106555918
C,0,3.8665857676,1.6393437117,-10.7383264161
H,0,4.2742210865,0.6484696996,-10.5295879293
C,0,2.7191995264,2.0796146295,-10.0702101933
H,0,2.224245393,1.4356389527,-9.340135271

C,0,2.2155115005,3.3541252129,-10.3327435563
H,0,1.3334505867,3.739473761,-9.8190256487
C,0,2.8681146286,4.1837910317,-11.2449454329
H,0,0.889537738,1.806161397,-13.2711204876
C,0,-0.8605451652,1.7051589109,-16.7838521638
C,0,-0.6609036432,2.772513707,-17.7275864888
C,0,-1.7724278121,3.3090017991,-18.4307225364
C,0,-3.0247362354,2.7915914767,-18.1757760008
C,0,-3.2056583971,1.754197304,-17.2139805104
C,0,-2.1492130832,1.2095885071,-16.5035511345
C,0,1.3642605714,2.1856820557,-16.8075716679
C,0,0.7195100135,3.0370245365,-17.7803940726
H,0,-1.6289268092,4.110841358,-19.1573011594
H,0,-3.8964644188,3.1772521387,-18.7072994952
H,0,-4.2146746672,1.3759618752,-17.0349579308
H,0,-2.3049168634,0.4128032677,-15.774673668
H,0,0.5104994994,0.6660318615,-15.5560026289
H,0,2.3028543324,1.7031600271,-17.0894027392
N,0,0.3489536862,1.3335827265,-16.302573039
C,0,1.407617997,3.9500049696,-18.7283636784
H,0,1.192573441,3.6388962462,-19.7650192499
H,0,1.0370094592,4.9802426436,-18.6163269472
H,0,2.4938012449,3.954461342,-18.5864896369

TS-2-*exo*-*Si*-(+*sc*)
Opt @ B3LYP-D3(BJ)/SDD/def2-SVP [Gaussian16]
SCF Done: E(RB3LYP) = -5024.49579720 a.u.
Zero-point correction = 1.254309 Hartree/Particle
Thermal correction to Gibbs Free Energy = 1.142925 Hartree/Particle
Imaginary Frequency = -341.9511 cm^{-1}
SP @ RI-B3LYP-D3(BJ)/def2-TZVPP in DCM (SMD) [ORCA 4.1]
FINAL SINGLE POINT ENERGY = -5026.529029026448 a.u.

Ir,0,3.7348811482,5.9451140628,-14.5516450369
Cl,0,5.8190496792,5.8398954859,-15.8797048275
C,0,2.6621510047,5.492529653,-16.3542182643
H,0,3.1535210315,5.9746121094,-17.1987281219
H,0,1.580938689,5.651490672,-16.3132383068
C,0,3.2282688371,4.2582911163,-15.8860578324
H,0,4.1943826783,4.0029036825,-16.3175675424
C,0,2.4448007786,3.1184575206,-15.4148814136
H,0,1.5777641241,3.4006143287,-14.8142147695
C,0,3.1160639753,1.9123185018,-14.876338394
C,0,4.4350073803,1.5645405163,-15.2215613865
H,0,5.0419043233,2.245339224,-15.8194694251
C,0,4.9843183926,0.3576909563,-14.7940538905
H,0,6.0101588894,0.1030131311,-15.0692290896
C,0,4.2273141118,-0.527569385,-14.0174093958
H,0,4.6581195961,-1.4772719354,-13.6927679377
C,0,2.9294525461,-0.1799703064,-13.6411444744
H,0,2.3433673669,-0.8478813544,-13.0068661927
C,0,2.3847806457,1.0330506708,-14.0620556679
P,0,3.9219749707,8.2790551368,-14.7336073523
O,0,5.3985752439,8.9438635942,-14.9990088832
O,0,3.563303665,8.9510102886,-13.2529842441
N,0,3.1311033032,9.1380213764,-15.9637756545
C,0,6.3512067566,9.1503322702,-14.0211731181

C,0,7.5414586049,8.3939766792,-14.1238658337
H,0,7.6224675564,7.6812355755,-14.944123569
C,0,8.5366280135,8.5682273801,-13.1924715102
H,0,9.4647879261,7.9969096011,-13.2665975545
C,0,8.3646907395,9.4652395335,-12.1021624436
C,0,9.3529701987,9.5887253043,-11.0873111698
H,0,10.2755857285,9.0115280375,-11.1857568914
C,0,9.1500701818,10.4025819892,-9.9945380465
H,0,9.9144508532,10.4854393287,-9.218979715
C,0,7.9366074741,11.1211460843,-9.8705343172
H,0,7.7655415424,11.7465369367,-8.9913325488
C,0,6.9655097278,11.0360882861,-10.846362624
H,0,6.0328430561,11.5847653533,-10.7259198686
C,0,7.1515455388,10.2259136286,-12.0016508424
C,0,6.1479271255,10.0979159817,-13.0269192191
C,0,4.9095113943,10.9192457241,-12.9916557043
C,0,4.9527543257,12.3563562048,-12.9472757127
C,0,6.1629625525,13.0857258767,-13.1146381224
H,0,7.092362722,12.5411921571,-13.2756248645
C,0,6.170320539,14.464247397,-13.0974083907
H,0,7.1107399555,15.0013736109,-13.2393541593
C,0,4.9701977671,15.191273806,-12.9042408965
H,0,4.99253725,16.2829705532,-12.884061645
C,0,3.7773326402,14.5179583837,-12.7592457665
H,0,2.8415456579,15.0673594423,-12.6305538583
C,0,3.7319060446,13.0967810014,-12.7939316644
C,0,2.496322746,12.3971475404,-12.7218975453
H,0,1.5720543742,12.9644658346,-12.5914625885
C,0,2.4581266056,11.0302132879,-12.8693714872
H,0,1.5173680939,10.4804979401,-12.8749042011
C,0,3.6635253246,10.3096939267,-13.0314346243
C,0,1.881157324,8.6933297356,-16.5008650322
C,0,0.7177267832,8.8124460164,-15.7353548459
H,0,0.7875427856,9.2673770037,-14.7492930287
C,0,-0.5015642391,8.3424458954,-16.2200406104
H,0,-1.4026476034,8.4394320342,-15.6157412709
C,0,-0.558787358,7.7477474165,-17.4850218632
H,0,-1.5110640565,7.3739868662,-17.8668228901
C,0,0.5952093837,7.6442296157,-18.2577744083
H,0,0.5482160604,7.1867425487,-19.249345381
C,0,1.8370997801,8.1246758195,-17.7953846504
C,0,3.0223734109,7.972322745,-18.6362824155
H,0,2.958447832,7.1749695515,-19.3835999926
C,0,4.1322794488,8.7481203682,-18.6646924925
H,0,4.8855974318,8.5119877255,-19.4220314028
C,0,4.3522147615,9.9841389203,-17.9222065425
C,0,5.1105682052,11.0149083919,-18.5119155947
H,0,5.5983217444,10.8254115305,-19.4711242885
C,0,5.2230553315,12.2648357273,-17.9079989394
H,0,5.8189148909,13.0473424452,-18.3827942801
C,0,4.555358379,12.5192279854,-16.7055251432
H,0,4.6198396447,13.4990951881,-16.2278956693
C,0,3.8204933755,11.5036902723,-16.09409261
H,0,3.3057387396,11.6871479318,-15.1566502019
C,0,3.7578844144,10.2333816416,-16.6669943665
P,0,1.9873438896,5.9877007697,-13.1783276219
O,0,1.2267644524,7.4208467513,-12.9902528306
O,0,0.7034503791,4.9519082509,-13.3926049842

N,0,2.4479957271,5.5219453729,-11.59689035
C,0,0.2694493108,7.6063518682,-11.9971236591
C,0,0.5918935117,8.4858326977,-10.9425129074
H,0,1.5558905834,8.9899338129,-10.9583341191
C,0,-0.3105743343,8.6671166681,-9.920971019
H,0,-0.0805460172,9.3513848944,-9.1011345175
C,0,-1.5297045509,7.936215047,-9.8890681325
C,0,-2.429361607,8.0449767993,-8.7930396255
H,0,-2.1747703324,8.7215139718,-7.973619553
C,0,-3.5908441128,7.3053441068,-8.7544513952
H,0,-4.271412331,7.3931346109,-7.9049214373
C,0,-3.8970082592,6.4171204337,-9.813967605
H,0,-4.807601649,5.8156081722,-9.7686094061
C,0,-3.0546333867,6.301264374,-10.8994549091
H,0,-3.3010661048,5.6102364292,-11.7053937925
C,0,-1.8559419432,7.0625033435,-10.9804903805
C,0,-0.9550746591,6.9676169443,-12.0985375913
C,0,-1.3308584361,6.2782759345,-13.359357978
C,0,-2.5351978382,6.6591230849,-14.0605194199
C,0,-3.3323788844,7.7682928116,-13.6590912877
H,0,-3.0378443588,8.3517248973,-12.7892211489
C,0,-4.4587069559,8.1329292744,-14.367185568
H,0,-5.0430153753,8.9962747061,-14.041601647
C,0,-4.8609997812,7.407055736,-15.5133707654
H,0,-5.7594683449,7.7026583755,-16.0589395109
C,0,-4.1029495498,6.3408032282,-15.9437938601
H,0,-4.3867556754,5.7832002763,-16.8397704252
C,0,-2.9246275896,5.9554836032,-15.248640631
C,0,-2.0924549292,4.9162161689,-15.7388508625
H,0,-2.3904295082,4.374701395,-16.6396187843
C,0,-0.9053073666,4.6281399024,-15.1146991555
H,0,-0.2386970889,3.8684344953,-15.5063644864
C,0,-0.5146619364,5.3202548562,-13.9445427678
C,0,3.3591414456,6.4264347776,-10.920932384
C,0,2.9751895677,6.9863290023,-9.7016583314
H,0,2.0003620837,6.7240920349,-9.292084629
C,0,3.8292225111,7.8683270793,-9.037712011
H,0,3.5244082017,8.3092297367,-8.0861168728
C,0,5.0728123026,8.1824180504,-9.5971120761
H,0,5.7478772693,8.8776306059,-9.09449626
C,0,5.4658116816,7.5968865493,-10.7985970132
H,0,6.4463695856,7.8193101752,-11.2161376362
C,0,4.6241071963,6.7019245151,-11.4776623201
C,0,5.1068954136,6.0487421882,-12.7067557686
H,0,6.0382115886,6.4573559505,-13.0997543259
C,0,4.8541846082,4.6849717209,-13.0196414317
H,0,5.6122801943,4.1941695272,-13.6332609836
C,0,4.0862516584,3.7656984216,-12.1534732323
C,0,4.562238913,2.4619095057,-11.9354810131
H,0,5.4820266853,2.1446750925,-12.4291147861
C,0,3.8819309372,1.5794233745,-11.0985910821
H,0,4.2804954008,0.5763484003,-10.93700348
C,0,2.6878286126,1.9674934873,-10.4824979543
H,0,2.1469115177,1.2709804481,-9.8382777086
C,0,2.1964902181,3.2586030826,-10.6835278609
H,0,1.2785018774,3.6036924945,-10.2053128095
C,0,2.9031002342,4.1538141006,-11.4869419639
H,0,1.3804210659,1.3151148164,-13.7386747379

C,0,1.1383281572,0.2856240979,-16.8317695598
C,0,2.2595445301,0.5351727383,-17.6992182405
C,0,2.9935756223,-0.5589445718,-18.2325959763
C,0,2.6127510478,-1.8360334904,-17.8875729412
C,0,1.5028307916,-2.0603863743,-17.0196001846
C,0,0.7584266884,-1.0243164599,-16.4847757388
C,0,1.4517514277,2.5295154934,-16.9115199992
C,0,2.4009839875,1.9256478029,-17.8184985295
H,0,3.8450925044,-0.3821413348,-18.8920986266
H,0,3.1609393542,-2.6960639416,-18.2759251201
H,0,1.2336485033,-3.0894608281,-16.7706570896
H,0,-0.084435498,-1.2133478142,-15.818373761
H,0,-0.1248431379,1.5949408759,-15.782487286
H,0,0.9732381783,3.4776132954,-17.164855915
N,0,0.6067340501,1.4800923157,-16.4728909771
C,0,3.4066478598,2.6610201208,-18.6246469925
H,0,3.1241877114,3.7084174161,-18.7848573148
H,0,4.3854124629,2.6596615942,-18.1126049385
H,0,3.5562524127,2.1732262623,-19.5995171838

TS-2-*exo*-*Si*-(-*sc*)
Opt @ B3LYP-D3(BJ)/SDD/def2-SVP [Gaussian16]
SCF Done: E(RB3LYP) = -5024.49390236 a.u.
Zero-point correction = 1.254524 Hartree/Particle
Thermal correction to Gibbs Free Energy = 1.145214 Hartree/Particle
Imaginary Frequency = -404.4285 cm^{-1}
SP @ RI-B3LYP-D3(BJ)/def2-TZVPP in DCM (SMD) [ORCA 4.1]
FINAL SINGLE POINT ENERGY = -5026.525329778239 a.u.

Ir,0,3.6924329553,5.9276599775,-14.5158294983
Cl,0,5.788501516,5.8053491878,-15.8284662814
C,0,2.6647819546,5.5545183995,-16.3605922283
H,0,3.1921674097,6.0495597247,-17.1738884756
H,0,1.5879987014,5.7344614455,-16.3399576602
C,0,3.164420118,4.2859429989,-15.9206706473
H,0,4.1421888967,4.0008978064,-16.3100623825
C,0,2.3004105901,3.1842777394,-15.5318364114
H,0,1.3290506665,3.4915853218,-15.1449619327
C,0,2.8578059781,2.0076935829,-14.8113315854
C,0,4.2005634129,1.6128670614,-14.9597497524
H,0,4.9037364964,2.2574887995,-15.4912263645
C,0,4.6481873478,0.4075083357,-14.4164951534
H,0,5.6914871908,0.1101512106,-14.5449756962
C,0,3.7710621676,-0.4092873461,-13.6968186323
H,0,4.1222110319,-1.3535948003,-13.2753684953
C,0,2.4497560186,0.0005091046,-13.4975667623
H,0,1.7682882554,-0.6151890197,-12.9071197509
C,0,1.9977867464,1.1964167223,-14.053979872
P,0,3.8746797191,8.2636464494,-14.7224832177
O,0,5.3495943585,8.9238347495,-15.0085441133
O,0,3.5272702267,8.9603995991,-13.2511762984
N,0,3.0762279918,9.1081179397,-15.9636725245
C,0,6.3122394389,9.1396753552,-14.0412339557
C,0,7.5006799362,8.3810850714,-14.1465666876
H,0,7.5727077222,7.6576489769,-14.9579879569
C,0,8.5056644189,8.5660094133,-13.2278363261
H,0,9.4323905546,7.9927293242,-13.3046070354

C,0,8.3461435961,9.4765193439,-12.1470253394
C,0,9.3452574643,9.6114199074,-11.1443254208
H,0,10.2660307563,9.0317135294,-11.2450932687
C,0,9.1549598616,10.4395127807,-10.0600442205
H,0,9.9275655185,10.5312528198,-9.2937064876
C,0,7.9438075834,11.161323317,-9.9325637203
H,0,7.7828451133,11.7982398278,-9.0597710247
C,0,6.9620798902,11.0646054738,-10.8965623129
H,0,6.0314714179,11.6159511577,-10.7727386903
C,0,7.1346912722,10.2392766547,-12.0431420065
C,0,6.1203384204,10.0994181509,-13.0563849367
C,0,4.8855499123,10.925564206,-13.0208714068
C,0,4.9364609132,12.3630481458,-12.9933845778
C,0,6.148872208,13.0842274656,-13.1795708415
H,0,7.0741610361,12.5333726244,-13.3424892218
C,0,6.16346398,14.4627684442,-13.1779775743
H,0,7.1053763984,14.993255106,-13.3341992764
C,0,4.9689362259,15.1983546383,-12.9823951346
H,0,4.9972200712,16.2900615123,-12.9743268208
C,0,3.7738861775,14.5330265625,-12.8198603262
H,0,2.842027324,15.088639444,-12.6894988729
C,0,3.7209748161,13.1117792703,-12.8387270689
C,0,2.4822483325,12.4196723587,-12.7532411553
H,0,1.5613692988,12.9929942331,-12.6253300976
C,0,2.4357079174,11.0517939763,-12.8888178689
H,0,1.4903817733,10.510670306,-12.8903231256
C,0,3.6363016858,10.3219740822,-13.0468305166
C,0,1.8236193984,8.6574299668,-16.4881439707
C,0,0.676692594,8.716118192,-15.6911584875
H,0,0.7584873386,9.1380352791,-14.6921628678
C,0,-0.5405645261,8.2181684372,-16.1553645584
H,0,-1.4245038047,8.2547876953,-15.5187899759
C,0,-0.6177941961,7.6784617522,-17.4435641795
H,0,-1.5700282365,7.2954140838,-17.8171454143
C,0,0.518078781,7.6418122497,-18.2498945307
H,0,0.4546944074,7.2290801828,-19.2594031825
C,0,1.7622815101,8.1271201657,-17.7991854893
C,0,2.9361443442,7.9919996748,-18.658538098
H,0,2.8670038263,7.2005892729,-19.4086779624
C,0,4.0526829087,8.7573928792,-18.6812075575
H,0,4.7999700038,8.5253803607,-19.4459304345
C,0,4.2827479662,9.9808952216,-17.9231444788
C,0,5.0370591745,11.0183537931,-18.5055418557
H,0,5.5238802712,10.8379259601,-19.467001529
C,0,5.1441803046,12.2645403855,-17.8929700335
H,0,5.7372140171,13.0530542448,-18.3612671233
C,0,4.4715712907,12.5072342368,-16.6912320221
H,0,4.5281571468,13.4844680327,-16.2073189988
C,0,3.7423445743,11.4831130822,-16.0869320013
H,0,3.2234390396,11.6600916339,-15.1512826487
C,0,3.6900966025,10.214527394,-16.6641840352
P,0,1.9515167529,5.9832019305,-13.1219208125
O,0,1.2130174381,7.4297177566,-12.966441221
O,0,0.6711226566,4.9365669529,-13.2644482538
N,0,2.4331156398,5.5704168478,-11.5318453456
C,0,0.3149268569,7.6807408856,-11.9343112389
C,0,0.7028642493,8.6027856857,-10.940289491
H,0,1.676194156,9.0807095681,-11.0197869404

C,0,-0.1501504613,8.8536878563,-9.8915403617
H,0,0.1295884254,9.5719694126,-9.1175579617
C,0,-1.379344548,8.1498533704,-9.7690403604
C,0,-2.2272690854,8.334874668,-8.6424127847
H,0,-1.9258293487,9.0511129584,-7.8742150997
C,0,-3.3966021697,7.6187515732,-8.5112373111
H,0,-4.037105077,7.7651656252,-7.6389376007
C,0,-3.7620044209,6.6778469271,-9.5041902691
H,0,-4.677496442,6.0942178064,-9.3847448522
C,0,-2.9711484958,6.4874523097,-10.6176513425
H,0,-3.2638663911,5.7551664699,-11.3695136748
C,0,-1.7668514354,7.223065794,-10.795026702
C,0,-0.9170667494,7.0541239495,-11.9452557605
C,0,-1.3598747875,6.2847185683,-13.1356037615
C,0,-2.6065449303,6.6301024769,-13.7783328809
C,0,-3.3317901992,7.8090068404,-13.4443395529
H,0,-2.9443423765,8.475013555,-12.6754659135
C,0,-4.5038706446,8.1348149492,-14.0946475262
H,0,-5.0312439971,9.0528781152,-13.8266096882
C,0,-5.0276633326,7.2964257482,-15.1074663077
H,0,-5.9624011837,7.5616636726,-15.6057224965
C,0,-4.3442646163,6.1563793743,-15.4685046478
H,0,-4.725834925,5.5068071434,-16.2601896085
C,0,-3.1206997945,5.8083094438,-14.8343018957
C,0,-2.3760673416,4.6714026121,-15.2412898737
H,0,-2.7888440479,4.0145985304,-16.0102010971
C,0,-1.139149983,4.4209459439,-14.702756384
H,0,-0.5549799748,3.5617190741,-15.0237982544
C,0,-0.6074481479,5.256169524,-13.6910537533
C,0,3.3632589633,6.4739229581,-10.8850053273
C,0,3.0054313032,7.0580640034,-9.6691689718
H,0,2.0325041892,6.8182253156,-9.2418589534
C,0,3.8831051543,7.9357681156,-9.0309958927
H,0,3.5989537735,8.3955148284,-8.0819361958
C,0,5.1234035668,8.2219575653,-9.6124446453
H,0,5.816253327,8.9135108079,-9.1295204253
C,0,5.4898792118,7.6126403486,-10.8105580745
H,0,6.4681992064,7.8113858208,-11.2449927514
C,0,4.6241239672,6.720426153,-11.4622067991
C,0,5.0776465668,6.0337288947,-12.6829427114
H,0,6.0085139508,6.4196696329,-13.0993588087
C,0,4.800629684,4.6669667596,-12.9601948784
H,0,5.5432059943,4.1523484355,-13.5733258502
C,0,4.0324048927,3.7786574358,-12.0617354075
C,0,4.4902788751,2.4735058148,-11.8166540869
H,0,5.4088473325,2.1368797271,-12.2980940455
C,0,3.7991131486,1.617407567,-10.9618932483
H,0,4.182380823,0.6112366796,-10.7822961725
C,0,2.6165958339,2.0373775741,-10.3440180665
H,0,2.0673009908,1.361126677,-9.6856053645
C,0,2.1491223135,3.3339480995,-10.5618205251
H,0,1.2412122877,3.704470755,-10.0836255157
C,0,2.8644080719,4.1989488481,-11.3895417782
H,0,0.9615945285,1.5094949964,-13.9022437638
C,0,3.2612551529,2.8064225261,-18.7706552904
C,0,2.1118908276,3.6432212544,-19.0053261531
C,0,2.1400882252,4.5943567433,-20.0616453609
C,0,3.2904325089,4.7160963856,-20.8068224351

C,0,4.4330376433,3.904707754,-20.5306878564
C,0,4.4400897033,2.9510721305,-19.5300588986
C,0,1.7417362525,2.3345699944,-17.1623543512
C,0,1.141575171,3.3011940693,-18.0538353167
H,0,1.2667844194,5.2154273966,-20.2647422247
H,0,3.3438399134,5.4417002306,-21.6203884344
H,0,5.3304943683,4.0419545966,-21.1380145825
H,0,5.3191294209,2.3348592323,-19.3362655659
H,0,3.639044253,1.3558504244,-17.2973005853
H,0,1.1329168967,1.5373682344,-16.7310025604
N,0,2.964426954,1.9488226349,-17.7691802858
C,0,-0.2272836696,3.8629685661,-17.9365058787
H,0,-0.9120137916,3.1634443033,-17.4387036839
H,0,-0.2225922472,4.7997082044,-17.3543906009
H,0,-0.6353709688,4.1057326287,-18.9284736013

TS-2-*exo*-*Si*-(*ap*)
Opt @ B3LYP-D3(BJ)/SDD/def2-SVP [Gaussian16]
SCF Done: E(RB3LYP) = -5024.49508256 a.u.
Zero-point correction = 1.254528 Hartree/Particle
Thermal correction to Gibbs Free Energy = 1.145566 Hartree/Particle
Imaginary Frequency = -431.8883 cm^{-1}
SP @ RI-B3LYP-D3(BJ)/def2-TZVPP in DCM (SMD) [ORCA 4.1]
FINAL SINGLE POINT ENERGY = -5026.524852806513 a.u.

Ir,0,3.8160627674,5.9372055755,-14.4998832198
Cl,0,5.9188851271,5.8564194958,-15.7939379639
C,0,2.8272540401,5.5847028313,-16.3578597086
H,0,3.3773742412,6.0884283889,-17.1533131752
H,0,1.7514299726,5.7762347049,-16.3472752351
C,0,3.3056369014,4.3009771211,-15.9199574226
H,0,4.2817698362,3.9851924127,-16.2938638443
C,0,2.3991952937,3.2338541742,-15.5262927938
H,0,1.4466584101,3.5952565513,-15.1524389024
C,0,2.8813784321,2.0366703084,-14.8038166621
C,0,4.1050485617,1.4218311245,-15.1197669317
H,0,4.7745826317,1.8781533001,-15.8523187885
C,0,4.4621858998,0.2147723179,-14.5200466495
H,0,5.4083018419,-0.2631873164,-14.7834694048
C,0,3.6096143908,-0.3859719927,-13.5864706497
H,0,3.8878246204,-1.3357803606,-13.1245525269
C,0,2.4153889297,0.2440340193,-13.2297583133
H,0,1.764273375,-0.2013644904,-12.4754576748
C,0,2.0529552349,1.4466909763,-13.835293833
P,0,3.9847004225,8.275237401,-14.6933096316
O,0,5.4570829161,8.9516165828,-14.9415196213
O,0,3.5923315185,8.9593450598,-13.2296104345
N,0,3.210809514,9.1076932112,-15.9581667076
C,0,6.3953870373,9.178912457,-13.9532735332
C,0,7.5968658191,8.4386759439,-14.0378142438
H,0,7.695834475,7.7191517572,-14.8500925163
C,0,8.5817213755,8.6381871591,-13.1005148599
H,0,9.5188968087,8.0802253991,-13.1610921425
C,0,8.387552346,9.5447208961,-12.0219219039
C,0,9.3665782276,9.6949369917,-11.0017340345
H,0,10.2984914836,9.1307912792,-11.0870061721
C,0,9.143705372,10.5185669483,-9.9202505973

H,0,9.9013099095,10.6224066482,-9.1406127317
C,0,7.918539816,11.2196977264,-9.8131659093
H,0,7.7316989632,11.8526686777,-8.942665609
C,0,6.9554647655,11.1077034893,-10.7941846028
H,0,6.0136849988,11.6430776772,-10.6858806474
C,0,7.1619198846,10.287247255,-11.9386112742
C,0,6.1686493608,10.133218776,-12.9706808963
C,0,4.9209134436,10.9407492126,-12.95748642
C,0,4.9500961177,12.3789490636,-12.9236150988
C,0,6.1550492576,13.1183657056,-13.0849226946
H,0,7.0911272796,12.5816948335,-13.2325305799
C,0,6.1494239275,14.4969775172,-13.0791014825
H,0,7.0862189715,15.0415857807,-13.2162708946
C,0,4.9408538168,15.2144369524,-12.9037291163
H,0,4.9529927478,16.3064003144,-12.8919080914
C,0,3.7528118264,14.5312278642,-12.7658785225
H,0,2.810622104,15.072777329,-12.6516384851
C,0,3.7210255292,13.1094359251,-12.790175718
C,0,2.4908626747,12.3994476897,-12.7303243714
H,0,1.5597717875,12.9590467024,-12.6163514965
C,0,2.4667126774,11.0316543922,-12.8722136833
H,0,1.5300750247,10.4755453863,-12.8917530783
C,0,3.6806452788,10.3207217399,-13.0121788232
C,0,1.9750016299,8.6495624245,-16.5132168186
C,0,0.8127106104,8.6851666932,-15.737396807
H,0,0.8714775379,9.0929132741,-14.7306508475
C,0,-0.3902489209,8.1822104562,-16.2314072338
H,0,-1.2869186031,8.2042115378,-15.6131766513
C,0,-0.4372821073,7.6546350438,-17.5257385815
H,0,-1.3791753634,7.2643197139,-17.9165104138
C,0,0.7138100689,7.6381392438,-18.3107819667
H,0,0.6775303901,7.2398173702,-19.329001161
C,0,1.9438145952,8.1337211159,-17.8317682609
C,0,3.1332522023,8.0209340438,-18.6729236027
H,0,3.0780533682,7.2497076346,-19.4482397763
C,0,4.244549449,8.7949324155,-18.6639646365
H,0,5.0051697449,8.5830942148,-19.4210789559
C,0,4.4499862336,10.0093337467,-17.8844165427
C,0,5.2070236443,11.0592770359,-18.4406673627
H,0,5.716335579,10.8923813988,-19.3927963411
C,0,5.2883021758,12.3007982128,-17.8147770487
H,0,5.8840723818,13.0989489359,-18.2627513891
C,0,4.5861192054,12.5265636838,-16.6267593923
H,0,4.6216889314,13.500109941,-16.1335329089
C,0,3.8537372624,11.4900748014,-16.047807131
H,0,3.3116148476,11.6538557727,-15.1228881002
C,0,3.8280471653,10.2261084521,-16.6366516262
P,0,2.0562669127,5.9521442512,-13.1212747299
O,0,1.2967147028,7.3874669297,-12.9709520875
O,0,0.7874216407,4.8905298516,-13.275360466
N,0,2.5352733216,5.535059755,-11.5313913254
C,0,0.3532636578,7.6160012043,-11.9742669659
C,0,0.690886166,8.5299595065,-10.954497522
H,0,1.6613430763,9.0195834988,-10.9898530553
C,0,-0.2078359637,8.7614041903,-9.9399850328
H,0,0.0319819598,9.473152765,-9.1467900691
C,0,-1.4369181786,8.0490022211,-9.8802121856
C,0,-2.3348958211,8.2160402314,-8.7902132231

H,0,-2.0716472222,8.9241090354,-8.0007076839
C,0,-3.5052997882,7.4930753598,-8.7199341341
H,0,-4.1846268724,7.6258101166,-7.8752546355
C,0,-3.8215352079,6.5630346055,-9.7395991453
H,0,-4.7386093609,5.9740435117,-9.6681707244
C,0,-2.9808749188,6.3898746317,-10.8188910015
H,0,-3.2357762047,5.6656588807,-11.5920357144
C,0,-1.7736732709,7.1331155167,-10.93354801
C,0,-0.8727343084,6.9811973081,-12.0466121155
C,0,-1.2547052939,6.2172878251,-13.2617304762
C,0,-2.4705997495,6.5562516807,-13.9635466008
C,0,-3.2250450185,7.7243564834,-13.6577312343
H,0,-2.8878823073,8.3852798611,-12.8611230384
C,0,-4.3612761895,8.0472557505,-14.3705375546
H,0,-4.9118315047,8.9574512337,-14.1230476264
C,0,-4.8181746555,7.2164545067,-15.421475588
H,0,-5.724524457,7.4807077493,-15.9702492225
C,0,-4.1059925206,6.0848520798,-15.7529898903
H,0,-4.4341834872,5.4391861035,-16.5713256409
C,0,-2.9184537032,5.7394279592,-15.0527501089
C,0,-2.1470030756,4.609482386,-15.4265411702
H,0,-2.5090447685,3.9514171567,-16.2155468464
C,0,-0.9392468923,4.3624963062,-14.8252934489
H,0,-0.3508604856,3.5012315609,-15.1281792822
C,0,-0.4677644938,5.1968706748,-13.7825287931
C,0,3.4419717049,6.4577173446,-10.8775330034
C,0,3.059208032,7.0436048452,-9.6702545035
H,0,2.0822432121,6.7955075904,-9.2569659928
C,0,3.919334833,7.9307145801,-9.0212416974
H,0,3.6166022282,8.391235827,-8.0783249497
C,0,5.1673302645,8.2231893612,-9.5826170116
H,0,5.8476950003,8.9199224248,-9.0896834435
C,0,5.5567823973,7.6152106544,-10.7740915785
H,0,6.5399066497,7.8215496195,-11.1937437436
C,0,4.7080052094,6.7162534452,-11.4387943283
C,0,5.1815594404,6.0451142569,-12.6611253684
H,0,6.1119662301,6.4466887326,-13.063465191
C,0,4.9224261442,4.6786060546,-12.9626618707
H,0,5.675749016,4.1808713817,-13.5768444695
C,0,4.1706830993,3.772845921,-12.0693932935
C,0,4.6645279589,2.4811214739,-11.8225567464
H,0,5.5688734114,2.1523761402,-12.3377331512
C,0,4.0290682998,1.6322828511,-10.9188853578
H,0,4.4437857545,0.6405680861,-10.7289241963
C,0,2.8583771067,2.0378077833,-10.2694931837
H,0,2.3536663476,1.3663609045,-9.5715787787
C,0,2.3399213417,3.3101650804,-10.5130197847
H,0,1.4352123973,3.6651628109,-10.0172017693
C,0,3.0043687816,4.175010966,-11.3828058502
H,0,1.1205673966,1.9415842928,-13.5557642193
C,0,-0.0326016052,3.4115376636,-18.2099286951
C,0,-0.3909351033,2.1319971686,-17.6626134844
C,0,-1.7247647787,1.6608055106,-17.793148358
C,0,-2.6428516866,2.4528754495,-18.4513409566
C,0,-2.2681360807,3.7232932971,-18.9782655915
C,0,-0.9828174723,4.2230762337,-18.8589455014
C,0,1.8349552225,2.5385009377,-17.2516377073
C,0,0.7779062832,1.5635240368,-17.1162044531

H,0,-2.0085505185,0.6870360068,-17.3893827999
H,0,-3.6714111104,2.1100516502,-18.5766548617
H,0,-3.023369077,4.3209273265,-19.4938467358
H,0,-0.7079438173,5.1958235641,-19.2643564334
H,0,1.7854462038,4.4737867461,-18.1734052473
H,0,2.8351822004,2.2032719927,-17.534020253
N,0,1.2909996673,3.6057742736,-18.0020473577
C,0,0.909266781,0.1994778799,-16.5424545943
H,0,1.955471003,-0.0960955455,-16.402177833
H,0,0.4159790859,0.1402730077,-15.5578036969
H,0,0.4132058123,-0.5361013674,-17.1957137251

TS-3-*endo*-*Si*-(-*sc*) [TS-3 in main text]
Opt @ B3LYP-D3(BJ)/SDD/def2-SVP [Gaussian16]
SCF Done: E(RB3LYP) = -5024.49942565 a.u.
Zero-point correction = 1.255640 Hartree/Particle
Thermal correction to Gibbs Free Energy = 1.146056 Hartree/Particle
Imaginary Frequency = -146.1871 cm^{-1}
SP @ RI-B3LYP-D3(BJ)/def2-TZVPP in DCM (SMD) [ORCA 4.1]
FINAL SINGLE POINT ENERGY = -5026.538705059045 a.u.

Ir,0,2.9509560893,7.1804931829,-17.0741591115
Cl,0,4.8741512825,6.3462629569,-18.4292433368
C,0,1.6906053745,5.7237251431,-18.0056612393
H,0,2.1051863266,5.4900989615,-18.9809956807
H,0,0.6273243579,5.9683732838,-18.0226470542
C,0,2.240754895,5.0822917639,-16.8443399337
H,0,1.6057740971,5.0230466225,-15.9599500011
C,0,3.3077767737,4.0522245409,-16.9012447997
C,0,3.9264369085,3.6490571781,-15.5987609995
C,0,3.157261365,3.3530478364,-14.4628610153
H,0,2.0793182993,3.5291549887,-14.4730344924
C,0,3.7501875209,2.8381409121,-13.3086344011
H,0,3.1345949827,2.6197932975,-12.4333848014
C,0,5.1303252097,2.6269704646,-13.2657860314
H,0,5.5958886052,2.2200106834,-12.3653859016
C,0,5.9167019207,2.9692120003,-14.3710352347
H,0,7.0000411234,2.8355053692,-14.3355599215
C,0,5.3190966262,3.4773177472,-15.5241687097
P,0,2.8185815932,8.9067445874,-18.659908579
O,0,4.163526956,9.3585698529,-19.4891507689
O,0,2.4931431631,10.3224797994,-17.8450895714
N,0,1.8266516173,8.7930974487,-20.0334678016
C,0,5.1484175286,10.1913574024,-19.0036961637
C,0,6.4241114213,9.6134604432,-18.8085252362
H,0,6.5368204648,8.5495274154,-19.017575972
C,0,7.4612688128,10.3960428234,-18.3616223968
H,0,8.4545198905,9.9645725918,-18.2176598803
C,0,7.2543955863,11.7681058515,-18.0491283372
C,0,8.3020109789,12.5651596321,-17.511525692
H,0,9.2886407599,12.1124853766,-17.3858788682
C,0,8.0801516718,13.8731398891,-17.1407259906
H,0,8.8917162331,14.4741440772,-16.725023237
C,0,6.7878608773,14.4327193337,-17.2844511726
H,0,6.605179048,15.4615778677,-16.9659738558
C,0,5.7544568979,13.6902637476,-17.8166523994
H,0,4.7642645762,14.1341233404,-17.9030797411

C,0,5.9531169478,12.3449309754,-18.2367436656
C,0,4.8910801345,11.5375088144,-18.7809520948
C,0,3.5465840037,12.1169272195,-19.0430639915
C,0,3.3681210499,13.2722370578,-19.8814475058
C,0,4.4249998913,13.8098673933,-20.6677967796
H,0,5.4058437744,13.3377976454,-20.6308554503
C,0,4.2186000352,14.8984577207,-21.4881364522
H,0,5.0433477607,15.2848131413,-22.0911358488
C,0,2.9464389325,15.516653787,-21.5623562478
H,0,2.7995910281,16.3840454598,-22.2095120779
C,0,1.8954148073,15.010187974,-20.8299666469
H,0,0.9037022941,15.4646115222,-20.8944492647
C,0,2.0691936151,13.8743788014,-19.9916710402
C,0,0.9713868113,13.2971368951,-19.2963769849
H,0,-0.0112395934,13.7683847638,-19.3711323644
C,0,1.1311863179,12.1289883084,-18.5883225782
H,0,0.2929178038,11.6319499767,-18.1002794677
C,0,2.4108912788,11.5354198802,-18.495847091
C,0,0.6586996836,7.9677064316,-20.0502334919
C,0,-0.4877578051,8.3720899956,-19.3605340856
H,0,-0.4720406597,9.3274398477,-18.8380218457
C,0,-1.6174966899,7.555806824,-19.3258928186
H,0,-2.506477606,7.8763761196,-18.7843960789
C,0,-1.6017115081,6.3242868983,-19.9902182229
H,0,-2.4838764732,5.6814074065,-19.9608871099
C,0,-0.4655408696,5.9254300666,-20.6899418909
H,0,-0.4550804375,4.9644343629,-21.2109716291
C,0,0.6862691796,6.7359062487,-20.7460087245
C,0,1.870612089,6.2527646565,-21.4524686917
H,0,1.9343985608,5.1649085243,-21.5563930817
C,0,2.8321825557,6.9831047292,-22.065640147
H,0,3.6077835211,6.4339440431,-22.6076226629
C,0,2.8420818986,8.4309393782,-22.2422520062
C,0,3.3950999015,8.9807965281,-23.4151290637
H,0,3.8935084525,8.3151877274,-24.1240675705
C,0,3.2916161347,10.3422734999,-23.6917213767
H,0,3.7308745351,10.7470741899,-24.6060752237
C,0,2.6080962683,11.1825032531,-22.8070009351
H,0,2.5062365802,12.2491378185,-23.0172738158
C,0,2.0762117304,10.6599924357,-21.6277897009
H,0,1.5530181108,11.3038979011,-20.9276879021
C,0,2.2334299351,9.3090513424,-21.3204311664
P,0,1.3746252363,7.9787436034,-15.7442390063
O,0,0.3722025456,9.1069629521,-16.3673186634
O,0,0.3130763966,6.9253883317,-15.0314653977
N,0,2.0386964617,8.6683339196,-14.3318468372
C,0,-0.5892895983,9.7216081054,-15.5687837552
C,0,-0.403802575,11.0881478341,-15.2715614199
H,0,0.4651170125,11.5985236368,-15.6837448713
C,0,-1.315238775,11.728405981,-14.4654461435
H,0,-1.1936625041,12.7888036372,-14.232756329
C,0,-2.4050820899,11.017048099,-13.8924022658
C,0,-3.3088433551,11.6495868432,-12.9951015838
H,0,-3.1635734201,12.7085162991,-12.7680197553
C,0,-4.3380306808,10.9437679275,-12.4118780332
H,0,-5.0229336176,11.4391362692,-11.7204048483
C,0,-4.4990876333,9.5665919058,-12.6983856756
H,0,-5.3014146293,9.0043692118,-12.2154230795

C,0,-3.6497867222,8.9270456785,-13.5765486156
H,0,-3.782718862,7.8650892991,-13.7810503966
C,0,-2.5893359188,9.6296208802,-14.213076637
C,0,-1.6853708064,8.9943864007,-15.1357544528
C,0,-1.9182559236,7.6171926597,-15.6451066494
C,0,-3.1553395576,7.2746891809,-16.3015921102
C,0,-4.1449528202,8.2481910264,-16.6168058702
H,0,-3.9771590676,9.2879517965,-16.3417123062
C,0,-5.2994774006,7.9011696261,-17.2870994926
H,0,-6.0357683056,8.6711363563,-17.5275730243
C,0,-5.5367717064,6.5600246406,-17.6724057553
H,0,-6.4584497078,6.2984510282,-18.1963271965
C,0,-4.5948953707,5.5938763688,-17.3946152102
H,0,-4.7571396456,4.5571180618,-17.6999806513
C,0,-3.3857125307,5.9234636007,-16.724215755
C,0,-2.3799575921,4.9462288972,-16.4996124591
H,0,-2.57065591,3.9133361733,-16.800396051
C,0,-1.1747303997,5.3007044576,-15.9470789493
H,0,-0.3802704183,4.5717041723,-15.7946918706
C,0,-0.9371877056,6.6391826182,-15.5552315815
C,0,2.8932873244,9.8200332934,-14.542058587
C,0,2.5449122882,11.0340321354,-13.9486608876
H,0,1.6385972505,11.0763123439,-13.3447977583
C,0,3.3508234781,12.1588770439,-14.1328096412
H,0,3.075797264,13.1090626502,-13.669833042
C,0,4.5121044203,12.0588145938,-14.906337285
H,0,5.1508239248,12.9299271536,-15.062073662
C,0,4.8651750541,10.8403399237,-15.4824435691
H,0,5.7789426677,10.7618650621,-16.0694020517
C,0,4.0674229859,9.6982072698,-15.3125726739
C,0,4.491609826,8.4229877508,-15.9207052745
H,0,5.3355100175,8.5131110067,-16.6054776293
C,0,4.349532298,7.1561347353,-15.2853912451
H,0,5.0930006925,6.4061743444,-15.5604934346
C,0,3.7792287412,6.9672237999,-13.932973183
C,0,4.3813552633,6.0575951204,-13.0488726446
H,0,5.2530458952,5.4948235827,-13.38426628
C,0,3.8832563725,5.867411913,-11.7607348246
H,0,4.3759925968,5.1573509987,-11.093053699
C,0,2.7555177994,6.5714824074,-11.3270913117
H,0,2.3585923623,6.4156694954,-10.3216900393
C,0,2.1411154411,7.4831578584,-12.1877157936
H,0,1.2607293794,8.0525338225,-11.8862822224
C,0,2.6593020607,7.6900102014,-13.4652301408
H,0,5.9313413987,3.7406501991,-16.388120682
H,0,4.079982547,4.3349385407,-17.6254086397
C,0,3.6154115788,0.7187806942,-16.5709339256
C,0,3.9443003151,1.6089627849,-17.6073049765
C,0,5.2070467866,1.5418065295,-18.1902160346
C,0,6.1085333517,0.5886276829,-17.7033773865
C,0,5.7642536399,-0.2743103139,-16.648940103
C,0,4.4993213989,-0.2245844139,-16.0577580479
C,0,1.7921449445,2.0024183997,-16.9047283408
C,0,2.8055958629,2.5584727453,-17.7840546757
H,0,5.4937528437,2.2252095055,-18.9917868181
H,0,7.1039673706,0.5162493502,-18.1460534125
H,0,6.495852233,-0.9988649075,-16.2866203519
H,0,4.2265958529,-0.8894953174,-15.2366368363

H,0,1.8065108577,0.560040802,-15.4153787652
H,0,0.7810714206,2.3766039808,-16.7450015105
N,0,2.2867365856,1.0096470964,-16.1911017411
C,0,2.4135571311,2.9305499881,-19.2081162266
H,0,1.4551744347,3.4614865525,-19.2384446522
H,0,3.1782818974,3.57894414,-19.657231658
H,0,2.3280760557,2.0170220859,-19.8147981513

TS-3-*endo-Si*-(+*sc*)
Opt @ B3LYP-D3(BJ)/SDD/def2-SVP [Gaussian16]
SCF Done: E(RB3LYP) = -5024.50658650 a.u.
Zero-point correction = 1.255904 Hartree/Particle
Thermal correction to Gibbs Free Energy = 1.147607 Hartree/Particle
Imaginary Frequency = -333.9370 cm^{-1}
SP @ RI-B3LYP-D3(BJ)/def2-TZVPP in DCM (SMD) [ORCA 4.1]
FINAL SINGLE POINT ENERGY = -5026.536858286455 a.u.

Ir,0,2.9871724248,7.2025896149,-17.0553788296
Cl,0,4.8910241556,6.3763941099,-18.4430972835
C,0,1.7626536495,5.7275091388,-17.9704118718
H,0,2.2058070694,5.5027122041,-18.936613704
H,0,0.6945486941,5.9449669505,-18.0073714899
C,0,2.292541177,5.0914865394,-16.7966814535
H,0,1.6265197171,5.0240743493,-15.9378785151
C,0,3.3857462595,4.1010482342,-16.7987992871
C,0,3.874056436,3.6219141,-15.4800822492
C,0,3.0102533314,3.3763244238,-14.3984834177
H,0,1.9456888789,3.5891397452,-14.4892840319
C,0,3.5012835544,2.8608645664,-13.1998396526
H,0,2.8164693579,2.6789847585,-12.3691202218
C,0,4.866110202,2.5952156567,-13.0534187833
H,0,5.2481447523,2.1898712289,-12.1138268968
C,0,5.7432546337,2.8707508026,-14.1068805136
H,0,6.8139378471,2.6884447153,-13.9920862746
C,0,5.2495057209,3.3783086181,-15.3092033598
P,0,2.8292952974,8.8997912149,-18.65636904
O,0,4.1540527108,9.3742909123,-19.5036939329
O,0,2.4720503225,10.3176138512,-17.8665187956
N,0,1.8397217831,8.7199108265,-20.0326166692
C,0,5.1278001629,10.2282364264,-19.0289519523
C,0,6.4136224173,9.6739239297,-18.8340176837
H,0,6.5449683555,8.6102704152,-19.0338402647
C,0,7.4386562661,10.479628467,-18.4000376909
H,0,8.4401304134,10.0675171816,-18.2566492842
C,0,7.2083669148,11.8507858232,-18.0999115242
C,0,8.2441646676,12.6712644312,-17.5748231989
H,0,9.239271662,12.2374943057,-17.4497371741
C,0,8.0006044582,13.9784291981,-17.2149019117
H,0,8.8032526372,14.5975643374,-16.8086871288
C,0,6.6978540285,14.5135660581,-17.3571779035
H,0,6.4984268173,15.541798512,-17.0469041712
C,0,5.6752280351,13.7480273868,-17.8773936108
H,0,4.6768135971,14.1732961644,-17.9625011843
C,0,5.8960760499,12.4028260732,-18.2862370892
C,0,4.846444178,11.5711519317,-18.8181621186
C,0,3.4901336537,12.1234028935,-19.0787948206
C,0,3.2856565637,13.2692857472,-19.924156171

C,0,4.3269947764,13.8166973548,-20.7242092949
H,0,5.3158342704,13.3612285558,-20.6910439745
C,0,4.0956246614,14.8937939201,-21.5530467677
H,0,4.90859206,15.2878100436,-22.166949762
C,0,2.8130567233,15.4908230607,-21.6213837291
H,0,2.6465950277,16.3496919002,-22.275093928
C,0,1.7770738433,14.9754666127,-20.8737841342
H,0,0.7781120393,15.4145376068,-20.9322904038
C,0,1.9765276474,13.85037994,-20.0267654406
C,0,0.8947471695,13.2636594316,-19.3143750051
H,0,-0.0949480436,13.7211911922,-19.3805396703
C,0,1.0785274997,12.1020127518,-18.6013858154
H,0,0.2526530492,11.5950916093,-18.102466486
C,0,2.3676040642,11.5271528419,-18.521124882
C,0,0.7305739545,7.8202400301,-20.0560445476
C,0,-0.4239105942,8.1185012926,-19.3252332387
H,0,-0.4551696123,9.0485969515,-18.7590819976
C,0,-1.4976426741,7.2290098284,-19.2936029393
H,0,-2.3861159043,7.467566456,-18.7104170319
C,0,-1.4288858553,6.0355626454,-20.0209404247
H,0,-2.2765047769,5.3464950674,-20.0150149942
C,0,-0.286923135,5.7418561803,-20.7644581074
H,0,-0.2523835796,4.8312249954,-21.3737479812
C,0,0.821551731,6.61537311,-20.7962426157
C,0,2.0257753934,6.2186301677,-21.5218215158
H,0,2.1457366113,5.1395219344,-21.6702072258
C,0,2.9481136066,7.0155165458,-22.1120191157
H,0,3.7500156874,6.5231460396,-22.6698496031
C,0,2.8779587982,8.464332766,-22.2495091778
C,0,3.4037912151,9.0709264598,-23.4065292188
H,0,3.9493642228,8.4530208666,-24.1237757253
C,0,3.2137734013,10.4280259163,-23.657682852
H,0,3.6337539477,10.8794279213,-24.5590315997
C,0,2.4682326607,11.2024006582,-22.7637147702
H,0,2.2983874553,12.2640503109,-22.9539154923
C,0,1.9631652522,10.6233251995,-21.5986523994
H,0,1.3964704067,11.2200052128,-20.8904502961
C,0,2.2080949162,9.2810901629,-21.313495237
P,0,1.4135764165,8.0011851143,-15.7191775944
O,0,0.4144263519,9.1315340982,-16.3464459201
O,0,0.3556037973,6.950024874,-15.0066315537
N,0,2.0864890307,8.6958755549,-14.3137213796
C,0,-0.5300464814,9.7649507683,-15.5407835654
C,0,-0.3306636696,11.1328860365,-15.2605481302
H,0,0.5359388717,11.6337003972,-15.6884489351
C,0,-1.2275375952,11.787123969,-14.4491459634
H,0,-1.0958339617,12.8489489067,-14.2288229455
C,0,-2.3142394558,11.0889643413,-13.8543059983
C,0,-3.2016834341,11.7367821239,-12.9516132967
H,0,-3.046160158,12.7968233312,-12.7368988767
C,0,-4.2277887752,11.0438692635,-12.3478678359
H,0,-4.8999075463,11.5507280255,-11.652210202
C,0,-4.402036463,9.6650719774,-12.6183117864
H,0,-5.2013419371,9.1132109618,-12.1187283952
C,0,-3.5690492292,9.0108447373,-13.5012982385
H,0,-3.7116081464,7.9477202385,-13.6925436152
C,0,-2.5122674765,9.6996769217,-14.1583795024
C,0,-1.6244727352,9.0496540185,-15.0867267394

C,0,-1.8774346725,7.6716426922,-15.5829557789
C,0,-3.1341587922,7.3432971595,-16.2087448113
C,0,-4.1062016773,8.3311376781,-16.534067219
H,0,-3.9056473007,9.3750512675,-16.2988309095
C,0,-5.2867422865,7.9904007055,-17.1610695969
H,0,-6.0098660686,8.770058725,-17.4101170124
C,0,-5.5690851056,6.6425003563,-17.4887824412
H,0,-6.5125452491,6.3862079076,-17.9752494136
C,0,-4.6433310057,5.6629892934,-17.2033809195
H,0,-4.8399005,4.6202440563,-17.4655293323
C,0,-3.4068623344,5.9857121601,-16.5807871007
C,0,-2.413173724,4.9964365868,-16.3605189041
H,0,-2.6333830564,3.959991836,-16.6157777567
C,0,-1.1785703755,5.3363600318,-15.8652771293
H,0,-0.4000066649,4.5858767295,-15.7354327693
C,0,-0.9099535114,6.6776906664,-15.5028220337
C,0,2.9328831912,9.8517914704,-14.532457992
C,0,2.5839614816,11.0639620467,-13.9355394502
H,0,1.686008077,11.1002051502,-13.319143011
C,0,3.377526271,12.1951817876,-14.1334058374
H,0,3.1015088222,13.1435577367,-13.667349572
C,0,4.5270325268,12.1043260147,-14.9254537535
H,0,5.1548865961,12.9810324159,-15.0935749962
C,0,4.8831484263,10.8872639998,-15.502594111
H,0,5.7899154381,10.8148978936,-16.1011374968
C,0,4.0992038684,9.7379527587,-15.316270427
C,0,4.5328651458,8.4631929859,-15.9174190723
H,0,5.3684583894,8.5560985152,-16.6119753134
C,0,4.4040200321,7.1995658422,-15.2759813334
H,0,5.1477897277,6.4513684272,-15.5571557607
C,0,3.8381438041,7.0036706082,-13.9229233969
C,0,4.4445206957,6.0908182489,-13.0450215579
H,0,5.3156412024,5.5295780266,-13.3846476699
C,0,3.9497132777,5.8935013597,-11.7568890444
H,0,4.4446428054,5.1804348169,-11.0944615944
C,0,2.8207993087,6.5920435592,-11.3178692163
H,0,2.4258218901,6.4292671444,-10.3128688684
C,0,2.2009302334,7.505164013,-12.172974428
H,0,1.3181606876,8.0686056427,-11.8674302434
C,0,2.7159102176,7.7198825215,-13.4503071947
H,0,5.9337063496,3.5953783108,-16.1322647921
H,0,4.2105933937,4.3847818714,-17.4554912985
C,0,0.591696341,2.4829416029,-18.0401479886
C,0,1.609261776,2.0354313221,-17.1749883028
C,0,1.259942964,1.3130734061,-16.0331326142
C,0,-0.0976191441,1.0868498037,-15.7791410286
C,0,-1.094198871,1.5613452276,-16.648930852
C,0,-0.7632093203,2.2691229761,-17.8074329346
C,0,2.5384849972,3.0691924668,-19.0022600882
C,0,2.9081182555,2.497225453,-17.7268683313
H,0,2.0233142649,0.9304562841,-15.3558012679
H,0,-0.3905365968,0.5211145448,-14.892468775
H,0,-2.1432896604,1.3610648649,-16.4222267947
H,0,-1.5262692978,2.6316032604,-18.4975098498
H,0,0.7269262735,3.6315311423,-19.8511684583
H,0,3.2016846599,3.5210915117,-19.7397806231
N,0,1.2227497933,3.0961746773,-19.138262219
C,0,4.1289184245,1.5986614763,-17.666429912

H,0,5.0205556883,2.1110818907,-18.0557408128
H,0,4.3355406566,1.2937103181,-16.6332244229
H,0,3.9600004574,0.6907486764,-18.2648678735

TS-3-*endo*-*Si*-(*ap*)
Opt @ B3LYP-D3(BJ)/SDD/def2-SVP [Gaussian16]
SCF Done: E(RB3LYP) = -5024.49878185 a.u.
Zero-point correction = 1.255804 Hartree/Particle
Thermal correction to Gibbs Free Energy = 1.146960 Hartree/Particle
Imaginary Frequency = -312.2915 cm^{-1}
SP @ RI-B3LYP-D3(BJ)/def2-TZVPP in DCM (SMD) [ORCA 4.1]
FINAL SINGLE POINT ENERGY = -5026.534291476153 a.u.

Ir,0,2.9617510323,7.1958909993,-17.135436121
Cl,0,4.894325329,6.3878075299,-18.4741492956
C,0,1.7191405449,5.7190931458,-18.0689235878
H,0,2.1552028843,5.4389164458,-19.0267200378
H,0,0.6560588489,5.9621060857,-18.1143679274
C,0,2.2454107973,5.088466512,-16.8883249683
H,0,1.6178506277,5.0477744994,-15.9984274659
C,0,3.3109103227,4.081686771,-16.940356896
C,0,4.0343997361,3.7544551346,-15.6717609404
C,0,3.3666849298,3.4607579258,-14.4740861332
H,0,2.2903800333,3.6163775002,-14.3924708064
C,0,4.0678435145,2.9665123942,-13.371709102
H,0,3.5293110763,2.7311689016,-12.4516176408
C,0,5.4532607929,2.7984117674,-13.4352896829
H,0,6.0005604615,2.4133696448,-12.572147174
C,0,6.1393029715,3.15781161,-14.6011676174
H,0,7.2272250099,3.0680525107,-14.6460058161
C,0,5.4343939257,3.6275633948,-15.7109970392
P,0,2.8360077286,8.9691631789,-18.6615995223
O,0,4.1791023567,9.4236127461,-19.4910166338
O,0,2.5483709115,10.362295362,-17.8001830337
N,0,1.814088729,8.9299519874,-20.0140697335
C,0,5.1758568484,10.2458185666,-19.0129624551
C,0,6.4514032662,9.6569035984,-18.8519299439
H,0,6.5517778708,8.5951443453,-19.077907475
C,0,7.5025442645,10.4259840646,-18.4150201057
H,0,8.495599604,9.9861893778,-18.2972840088
C,0,7.3113209163,11.7949092364,-18.0797552116
C,0,8.3761369506,12.5771627314,-17.5543158705
H,0,9.3621461094,12.1161387457,-17.4574842432
C,0,8.1717376675,13.8808930977,-17.1595631262
H,0,8.9964085054,14.4705392587,-16.7535459033
C,0,6.8801565831,14.4501781888,-17.2653587997
H,0,6.7110395064,15.4750148975,-16.9270524182
C,0,5.8302806217,13.7224601776,-17.7856829016
H,0,4.841726755,14.1742179977,-17.8424565868
C,0,6.0102438433,12.3827285204,-18.2316005134
C,0,4.9307422116,11.5899754222,-18.7649560279
C,0,3.5828408034,12.1814481751,-18.9779961448
C,0,3.3888445662,13.3616115342,-19.7774329373
C,0,4.4233719522,13.9068657269,-20.5875428087
H,0,5.3980097948,13.4207530817,-20.6000658483
C,0,4.2019777314,15.0196530628,-21.3705248207
H,0,5.0088066499,15.411026848,-21.9941524032

C,0,2.9368744459,15.6564175599,-21.3800705338
H,0,2.7782483818,16.5428935776,-21.9979263342
C,0,1.9069456522,15.1444710208,-20.6218355721
H,0,0.9201726966,15.6137628048,-20.63646485
C,0,2.0956297012,13.9836151183,-19.8219813972
C,0,1.0158162585,13.3993032243,-19.1049662509
H,0,0.0372675282,13.884123622,-19.1302200627
C,0,1.1839343906,12.2051170272,-18.4439736469
H,0,0.3546315987,11.6983367816,-17.9519384662
C,0,2.4584873521,11.5946862566,-18.4132161113
C,0,0.7003595111,8.0363509061,-20.0923355015
C,0,-0.486553706,8.3348112718,-19.4172396781
H,0,-0.5418731374,9.2574372302,-18.8407213118
C,0,-1.5679953131,7.4564392547,-19.4710586657
H,0,-2.4909569347,7.6934479514,-18.9438555053
C,0,-1.4622870392,6.2744485323,-20.2131390484
H,0,-2.3099556113,5.5875905562,-20.2624983635
C,0,-0.2827101355,5.9812390443,-20.8929150012
H,0,-0.210905672,5.0651982468,-21.482587103
C,0,0.8264979164,6.8503190166,-20.8516554901
C,0,2.0676401359,6.481716163,-21.5313715119
H,0,2.2340182535,5.4089357481,-21.6447792993
C,0,2.9837457322,7.3034554211,-22.0946847221
H,0,3.820905115,6.8348169605,-22.6203403285
C,0,2.8780790602,8.7516175871,-22.2232120537
C,0,3.3987659422,9.3937127319,-23.3626613584
H,0,3.9574940314,8.8017304923,-24.0916653577
C,0,3.1882592517,10.7535674013,-23.5816758952
H,0,3.6033333748,11.2328173645,-24.4710134839
C,0,2.4302850036,11.4966057886,-22.671279044
H,0,2.2471951243,12.5602230774,-22.8365868446
C,0,1.9309546857,10.882549217,-21.5220179714
H,0,1.3548832017,11.4526296606,-20.7994040874
C,0,2.1922664158,9.5364306514,-21.2722797967
P,0,1.3859772886,7.9911170703,-15.7900990279
O,0,0.3849103121,9.1231826105,-16.4088478772
O,0,0.3298761849,6.9461029314,-15.0600939425
N,0,2.0585426946,8.6863832032,-14.384009814
C,0,-0.5968611659,9.717602167,-15.6174854516
C,0,-0.4268250104,11.0785363783,-15.2867422548
H,0,0.4515631306,11.6019242488,-15.6593830257
C,0,-1.3661876576,11.6999813213,-14.4982070744
H,0,-1.2560343151,12.7561091084,-14.2415919005
C,0,-2.4716919506,10.9748122284,-13.9753078902
C,0,-3.4082233088,11.5886608551,-13.0989275402
H,0,-3.2768791931,12.6447957067,-12.8512777466
C,0,-4.451547057,10.8681815776,-12.5605896265
H,0,-5.1619790489,11.3489464253,-11.8847432999
C,0,-4.5937297254,9.4944562445,-12.8722507637
H,0,-5.4072724024,8.9199123076,-12.4238241715
C,0,-3.7120554645,8.8730707925,-13.7313735205
H,0,-3.8313410362,7.8133266724,-13.9539749351
C,0,-2.6364579967,9.5915735641,-14.3233550482
C,0,-1.6983477535,8.9761610227,-15.2257745726
C,0,-1.8953575868,7.5951921834,-15.7386606394
C,0,-3.1070314892,7.2273281854,-16.4265370537
C,0,-4.0934590063,8.1858524985,-16.7925908813
H,0,-3.9433445375,9.2319565573,-16.5305166703

C,0,-5.2208195921,7.8154462016,-17.4959203248
H,0,-5.9553898063,8.5734347634,-17.7764119307
C,0,-5.4322760561,6.4648293009,-17.8633236848
H,0,-6.3328362098,6.184771966,-18.4137857
C,0,-4.4935685394,5.5120472093,-17.5326230859
H,0,-4.6376109284,4.467734991,-17.820704451
C,0,-3.3115334595,5.8650883774,-16.8267700248
C,0,-2.3101148503,4.9001351715,-16.536995381
H,0,-2.4842527676,3.8558621528,-16.8066154468
C,0,-1.1260296676,5.2781423285,-15.9543625261
H,0,-0.3415919413,4.5544961548,-15.7441964122
C,0,-0.9079751767,6.629557182,-15.6007759693
C,0,2.900242789,9.848033783,-14.5868590286
C,0,2.5414934135,11.0540254239,-13.983470894
H,0,1.6330415764,11.0846938863,-13.38217295
C,0,3.3417650057,12.185178911,-14.1521591128
H,0,3.0585423634,13.1289281802,-13.6810658598
C,0,4.5092517118,12.0998702242,-14.9182402235
H,0,5.144832537,12.9758107549,-15.0588097975
C,0,4.8722621009,10.8901180417,-15.5059217931
H,0,5.790764218,10.8225434447,-16.0868368345
C,0,4.0786340028,9.7427029295,-15.3529800134
C,0,4.5164409746,8.4762682185,-15.9663708859
H,0,5.3401025675,8.5737429672,-16.6742179007
C,0,4.394586159,7.2151938649,-15.3326468683
H,0,5.1302219291,6.4644994865,-15.6243401765
C,0,3.8319616661,7.023854135,-13.9787811446
C,0,4.4682204784,6.1471830607,-13.0857213935
H,0,5.3568683103,5.6098388606,-13.4180250918
C,0,3.9836466982,5.9613229818,-11.7919686272
H,0,4.5025571427,5.2788925703,-11.1153873221
C,0,2.8343040517,6.6335734199,-11.3640674666
H,0,2.4462613885,6.477868951,-10.3552386702
C,0,2.1881485964,7.5149569368,-12.2328620577
H,0,1.2934323373,8.0625226601,-11.9334727297
C,0,2.6954801978,7.722010263,-13.5147487729
H,0,5.9633412182,3.9016928919,-16.6270976545
H,0,4.007681804,4.2668156611,-17.7601459367
C,0,3.7012512845,1.9197582073,-19.5318029102
C,0,2.5300952657,2.5353002696,-19.0472278675
C,0,1.5956232252,3.0206724326,-19.9607065838
C,0,1.8774285102,2.8986678056,-21.3259859285
C,0,3.0778262572,2.3301813546,-21.7819497905
C,0,4.0200904531,1.8242707293,-20.8828415829
C,0,3.6857754429,1.5987582349,-17.2998458529
C,0,2.5508790673,2.4611665669,-17.5593997278
H,0,0.6654587471,3.4797283194,-19.6250445089
H,0,1.1484837709,3.25006452,-22.0580798077
H,0,3.2698999361,2.2637690056,-22.8544015165
H,0,4.9423097892,1.3538795425,-21.2285727778
H,0,5.2649444898,0.9001882365,-18.4536362254
H,0,4.0308569498,1.2216803124,-16.3394945005
N,0,4.3715862701,1.3836964206,-18.4124998021
C,0,1.2421335238,2.2367499335,-16.8310528074
H,0,1.3830185376,2.1918798016,-15.7436505109
H,0,0.5478845789,3.0551855464,-17.0630102107
H,0,0.7776069534,1.2941996207,-17.1577648383

TS-3-*endo*-*Re*-(-*sc*)
Opt @ B3LYP-D3(BJ)/SDD/def2-SVP [Gaussian16]
SCF Done: E(RB3LYP) = -5024.50321399 a.u.
Zero-point correction = 1.255836 Hartree/Particle
Thermal correction to Gibbs Free Energy = 1.147163 Hartree/Particle
Imaginary Frequency = -320.3163 cm^{-1}
SP @ RI-B3LYP-D3(BJ)/def2-TZVPP in DCM (SMD) [ORCA 4.1]
FINAL SINGLE POINT ENERGY = -5026.538642190456 a.u.

Ir,0,2.795832405,5.514339408,-14.238709857
Cl,0,5.0229530384,5.6106379405,-15.3583846297
C,0,1.8705267202,5.2713784608,-16.1529403126
H,0,2.5247228234,5.7141977133,-16.8976398951
H,0,0.8316906998,5.5984968828,-16.2190277469
C,0,2.1397612513,3.9351932968,-15.7007752047
H,0,1.3065028146,3.3717943036,-15.2806453614
C,0,3.2576165936,3.110455502,-16.1837028437
C,0,3.5378485769,1.8392563921,-15.4522300377
C,0,2.5214774478,0.9482192541,-15.08022975
H,0,1.4759294433,1.215573406,-15.2360339768
C,0,2.8337953292,-0.2949205172,-14.5234085901
H,0,2.0285261842,-0.9760032051,-14.2406083235
C,0,4.1655543484,-0.6599373392,-14.3172211455
H,0,4.4082433323,-1.6314735809,-13.8808326232
C,0,5.1885199842,0.2380797415,-14.6469814001
H,0,6.2317889041,-0.0261526664,-14.4592027818
C,0,4.8749510976,1.4760582717,-15.2091936798
P,0,2.9564005768,7.8498881571,-14.1723723348
O,0,4.4351602763,8.5641486116,-14.1680329725
O,0,2.3890708661,8.3601096932,-12.6942261127
N,0,2.3186009169,8.8255071511,-15.4064797913
C,0,5.2406023577,8.7176927548,-13.0599606547
C,0,6.4657758709,8.0116497976,-13.0685294311
H,0,6.6766611235,7.3710746229,-13.9248770697
C,0,7.3340614574,8.1481662576,-12.0123950311
H,0,8.2890730537,7.617713865,-12.0125415164
C,0,6.9934762037,8.9534203642,-10.8905667828
C,0,7.8498767416,9.0365611755,-9.7585147655
H,0,8.8032075573,8.5031810406,-9.7864474559
C,0,7.4842836431,9.7578079689,-8.643301028
H,0,8.1486962039,9.8109102668,-7.7782174812
C,0,6.2324394661,10.4180715674,-8.61500715
H,0,5.9306976686,10.967880671,-7.7205821615
C,0,5.386048274,10.3690366374,-9.7029222914
H,0,4.4204756191,10.8692954053,-9.6542747831
C,0,5.7423261765,9.6573641531,-10.8829092767
C,0,4.8739799166,9.5735819139,-12.0297019783
C,0,3.6006092524,10.3398520305,-12.0821605973
C,0,3.5652675808,11.7671794506,-11.9053412908
C,0,4.7479354412,12.5573035634,-11.8695318807
H,0,5.7155578171,12.0680201879,-11.9721534436
C,0,4.6841013202,13.9274731712,-11.731238372
H,0,5.6061649839,14.5128820339,-11.7187390756
C,0,3.4350392396,14.5845195727,-11.6121978325
H,0,3.3998261707,15.6696324013,-11.493814086
C,0,2.2690119659,13.8528815192,-11.6632216016
H,0,1.2981977211,14.3497475293,-11.594169254

C,0,2.2993133506,12.4404865265,-11.828274436
C,0,1.0998993667,11.6890669887,-11.964399878
H,0,0.139496884,12.2041706696,-11.8903673
C,0,1.1480411324,10.3435840122,-12.2452723503
H,0,0.2446812333,9.759234385,-12.4185753498
C,0,2.3990399717,9.6908521971,-12.3304431237
C,0,1.2315105302,8.3908070787,-16.2285039167
C,0,-0.0643736776,8.3544261783,-15.7057365693
H,0,-0.2228028934,8.6818574918,-14.6791573164
C,0,-1.1260558102,7.8863106322,-16.4788057354
H,0,-2.1326011602,7.8593365661,-16.0639238472
C,0,-0.8901356987,7.4525321265,-17.7882323694
H,0,-1.719411819,7.0815866588,-18.3941976286
C,0,0.3977516487,7.5008899172,-18.3161218947
H,0,0.5807603244,7.1636657275,-19.3397459916
C,0,1.486228451,7.9774669789,-17.5577926439
C,0,2.8271693409,7.9587430751,-18.1381363983
H,0,2.9690748724,7.2376202423,-18.9492872339
C,0,3.8691638526,8.7772663636,-17.8588721013
H,0,4.778439234,8.6502995133,-18.4537386133
C,0,3.8457208714,9.9397092836,-16.978517161
C,0,4.6309358176,11.0646974681,-17.2972113309
H,0,5.3184036652,11.0035477373,-18.1443497911
C,0,4.5221325035,12.2490093583,-16.571696945
H,0,5.1431409622,13.1079600184,-16.8351065272
C,0,3.6029338853,12.3402589194,-15.5216604194
H,0,3.4952309014,13.2666301055,-14.9537858257
C,0,2.8362624162,11.2275244682,-15.1744815171
H,0,2.127454992,11.2824657838,-14.3541632929
C,0,2.9898725587,10.0230683157,-15.8597765104
P,0,0.921578996,5.3994220897,-13.0594930914
O,0,0.0635392661,6.7794747548,-12.9048846429
O,0,-0.2455748689,4.3045558332,-13.475515908
N,0,1.219112391,4.8525548188,-11.4697524807
C,0,-1.0740086386,6.825164458,-12.1017838209
C,0,-0.9990886058,7.5875323104,-10.9171683094
H,0,-0.0705961605,8.1076812251,-10.6885038228
C,0,-2.0924397111,7.6419601134,-10.0851605109
H,0,-2.0564959811,8.2355866757,-9.1688122573
C,0,-3.2693666795,6.9007958943,-10.3811139019
C,0,-4.3770456448,6.887772642,-9.4895243321
H,0,-4.3173881505,7.4813842984,-8.5740624507
C,0,-5.4972030351,6.1340557814,-9.7628712665
H,0,-6.3397744335,6.1279765116,-9.0681362635
C,0,-5.5499449587,5.3519323131,-10.9419243346
H,0,-6.4296008714,4.7365402405,-11.1433140479
C,0,-4.4996217962,5.3551146392,-11.8353944948
H,0,-4.5524749672,4.7422561348,-12.7347018977
C,0,-3.336362503,6.137810884,-11.5956343557
C,0,-2.2222217598,6.1659496832,-12.5066325989
C,0,-2.2894770399,5.5393721566,-13.8526986689
C,0,-3.3248196094,5.9079509041,-14.7859745089
C,0,-4.2338976597,6.9735593576,-14.5324578651
H,0,-4.1643693665,7.5223318502,-13.5948692042
C,0,-5.1827295087,7.3390363435,-15.4646487253
H,0,-5.8588056071,8.1693128665,-15.2493289779
C,0,-5.2847966142,6.6544240737,-16.6992909374
H,0,-6.045047751,6.9501788232,-17.4251991919

C,0,-4.413790633,5.6253285585,-16.9826733833
H,0,-4.4707130306,5.0977204472,-17.9381361163
C,0,-3.4118189081,5.2387951166,-16.051812189
C,0,-2.4665379603,4.226409062,-16.3657346597
H,0,-2.554959751,3.7008904916,-17.3193264098
C,0,-1.4391516495,3.9375533326,-15.5027279155
H,0,-0.6946381377,3.1803622724,-15.7369880022
C,0,-1.3341986919,4.6237799057,-14.2720876424
C,0,2.052330425,5.7092636795,-10.6518848347
C,0,1.5293784868,6.2262975716,-9.4659759124
H,0,0.5070539446,5.9668818831,-9.1916435139
C,0,2.3117922126,7.0552928917,-8.660181846
H,0,1.9005828993,7.4609474126,-7.7332551812
C,0,3.6227313409,7.3574254671,-9.0439255256
H,0,4.2449801335,8.0073165613,-8.4263639769
C,0,4.1466290363,6.8250909545,-10.2200159335
H,0,5.1734448187,7.0468916025,-10.5065639613
C,0,3.3764417467,5.9900713821,-11.0443195936
C,0,3.9804336341,5.4211679469,-12.2624192615
H,0,4.9510574182,5.8443604482,-12.5230844816
C,0,3.7463004165,4.0964525217,-12.7203341549
H,0,4.5597886672,3.645763972,-13.2911457861
C,0,2.8767557069,3.116989505,-12.032873237
C,0,3.2801055889,1.7745884057,-11.9450616341
H,0,4.2254634626,1.4769080445,-12.3990643093
C,0,2.4947442348,0.8279502269,-11.2892099169
H,0,2.8363465298,-0.2077558183,-11.232201924
C,0,1.273144341,1.1963139691,-10.7167519859
H,0,0.6520888053,0.4530311292,-10.2123853172
C,0,0.8515496602,2.5250882315,-10.7909984956
H,0,-0.095644859,2.8510702911,-10.3590655225
C,0,1.6526257358,3.4735266654,-11.4249522964
H,0,5.6709497108,2.1809613842,-15.4645120612
C,0,2.0282205482,0.5407681041,-18.0953236273
C,0,1.7079783976,1.9114991366,-18.063242961
C,0,0.3678845357,2.2972821383,-18.0291422228
C,0,-0.6087201757,1.2962201842,-18.0067997806
C,0,-0.2627552335,-0.066842011,-18.0187811544
C,0,1.0728970988,-0.4713230106,-18.068501225
C,0,3.9823290992,1.6553800161,-18.1641258432
C,0,2.9747413667,2.6817248408,-17.9998850937
H,0,0.0912984744,3.3522443597,-18.0158438649
H,0,-1.664326867,1.572831676,-17.9792702865
H,0,-1.0510743896,-0.8215974043,-17.9970395378
H,0,1.3489405906,-1.5270699045,-18.0809484938
H,0,3.9549416095,-0.4201851824,-18.1174468799
H,0,5.0630005183,1.7870200575,-18.2043217055
N,0,3.4327943411,0.4511404054,-18.1611727087
C,0,3.1223172562,3.9736520192,-18.7873059644
H,0,3.1914790278,3.7553138344,-19.8635673825
H,0,2.2463729982,4.6128045166,-18.6310992182
H,0,4.0134137923,4.5378760164,-18.4776485119
H,0,4.1644252805,3.6993540278,-16.3354082972

TS-3-*endo*-*Re*-(+*sc*)
Opt @ B3LYP-D3(BJ)/SDD/def2-SVP [Gaussian16]
SCF Done: E(RB3LYP) = -5024.49928243 a.u.

Zero-point correction = 1.255387 Hartree/Particle
Thermal correction to Gibbs Free Energy = 1.145627 Hartree/Particle
Imaginary Frequency = -312.6449 cm^{-1}
SP @ RI-B3LYP-D3(BJ)/def2-TZVPP in DCM (SMD) [ORCA 4.1]
FINAL SINGLE POINT ENERGY = -5026.536864326293 a.u.

Ir,0,2.8040385877,5.6157284887,-14.2791205419
Cl,0,5.0376174024,5.644420276,-15.3696223952
C,0,1.9167180544,5.3532345915,-16.2141892998
H,0,2.58246536,5.7409009964,-16.9855180541
H,0,0.8863622,5.7021642137,-16.3030541086
C,0,2.1675063386,4.0323185825,-15.6949000138
H,0,1.3301099638,3.4967032492,-15.2476843293
C,0,3.2432147846,3.1563673358,-16.1919700286
C,0,3.5807704241,1.9349414503,-15.4100600335
C,0,2.6023318159,1.0352493022,-14.9560211739
H,0,1.5427674335,1.2810491075,-15.0543247706
C,0,2.9639917185,-0.176561866,-14.3698625112
H,0,2.1894776902,-0.8671385578,-14.0306370785
C,0,4.314527983,-0.4969915439,-14.1964824014
H,0,4.5982331859,-1.4484914185,-13.7415790078
C,0,5.2976863721,0.4163790273,-14.5868093313
H,0,6.3536743886,0.1860714039,-14.4296221523
C,0,4.9328800121,1.6202406876,-15.1938998501
P,0,3.006894699,7.9532638345,-14.1116435928
O,0,4.5025838261,8.6314180901,-14.1157092492
O,0,2.4767737939,8.410826503,-12.5987424903
N,0,2.3474293866,9.0181182406,-15.2549005998
C,0,5.336156665,8.716293723,-13.0205977307
C,0,6.5383292681,7.9744445898,-13.0821468418
H,0,6.7121612136,7.3607352987,-13.9659288547
C,0,7.4287732661,8.0400879789,-12.0379090561
H,0,8.3658572234,7.4799743344,-12.0776371318
C,0,7.135213803,8.8102501704,-10.8788153856
C,0,8.0165081525,8.8233493873,-9.7629772492
H,0,8.952116135,8.2629956854,-9.8319291097
C,0,7.6962221161,9.5113222004,-8.6134360639
H,0,8.3792249753,9.5106009154,-7.7613112515
C,0,6.4663409782,10.2074892217,-8.5332641195
H,0,6.2000639437,10.7312817614,-7.6123415648
C,0,5.5972269127,10.226984654,-9.6041437625
H,0,4.6495490573,10.7551835689,-9.5160759601
C,0,5.9073186654,9.5515624683,-10.8181152232
C,0,5.0138507091,9.5377982924,-11.9487388897
C,0,3.7582121158,10.3345163201,-11.9407120088
C,0,3.7650031693,11.7541112347,-11.7063309685
C,0,4.9691495693,12.5112673681,-11.6692169361
H,0,5.9203334678,12.001697583,-11.8167830557
C,0,4.945945524,13.8756448043,-11.4730661168
H,0,5.8836156009,14.4357138779,-11.4603922899
C,0,3.7185181171,14.5595456147,-11.2946807304
H,0,3.7157370844,15.6392880339,-11.1307589308
C,0,2.5322352695,13.8613536748,-11.3460268193
H,0,1.5771717875,14.3800019232,-11.2320251594
C,0,2.5203621431,12.4567767992,-11.5697017194
C,0,1.2986893988,11.7427519425,-11.7071916661
H,0,0.3542401491,12.2788125752,-11.5895331513
C,0,1.3049387608,10.4089940695,-12.0423970475

H,0,0.3818866789,9.8566268421,-12.2157927622
C,0,2.5341581588,9.7254336937,-12.1837636196
C,0,1.1956878281,8.6627038753,-16.0225536731
C,0,-0.0729070486,8.7096862872,-15.4390953778
H,0,-0.1584001944,9.0383749619,-14.405162383
C,0,-1.1986659048,8.3276485337,-16.1654815222
H,0,-2.1852055168,8.3621748871,-15.705611121
C,0,-1.0554686176,7.9044371611,-17.4918973003
H,0,-1.9371744672,7.6055800528,-18.0629374414
C,0,0.2062552934,7.8793615497,-18.0809578573
H,0,0.316677236,7.5692759933,-19.1207011164
C,0,1.3600835312,8.2613218958,-17.3667397557
C,0,2.6690834972,8.1951471008,-18.0110686546
H,0,2.7421217133,7.4756427175,-18.8268811993
C,0,3.7566822307,8.9668408269,-17.7763121239
H,0,4.6284788612,8.808503383,-18.4183937505
C,0,3.8319076279,10.1117794035,-16.8760995598
C,0,4.6447050624,11.2117373421,-17.212035042
H,0,5.2787671785,11.1466580495,-18.0996341837
C,0,4.6297912846,12.3776138104,-16.4495394675
H,0,5.270422971,13.2176615408,-16.7266821432
C,0,3.7799239913,12.4756898871,-15.342869023
H,0,3.7467401843,13.3885285616,-14.7445848229
C,0,2.9870803458,11.3866254578,-14.9801356464
H,0,2.3305917797,11.4468700563,-14.1179436234
C,0,3.0468352753,10.1983261863,-15.7072149652
P,0,0.9079314272,5.4944956901,-13.1454695085
O,0,0.0199226677,6.8579977339,-13.0160041206
O,0,-0.234288271,4.3777903091,-13.5965627722
N,0,1.1588555958,4.9493183195,-11.5489187563
C,0,-1.156565098,6.8804924193,-12.2727903727
C,0,-1.1476771529,7.6327690314,-11.0792624684
H,0,-0.234786071,8.1562779841,-10.8005072565
C,0,-2.2794834157,7.6707171132,-10.3000316644
H,0,-2.2931684192,8.2571573023,-9.3785018862
C,0,-3.4323454888,6.9187620934,-10.6563431073
C,0,-4.5791306572,6.886857028,-9.8162553153
H,0,-4.569462196,7.4780946325,-8.897414442
C,0,-5.6741576958,6.1171543576,-10.1415340659
H,0,-6.5472490301,6.0961507862,-9.4859516015
C,0,-5.6595923871,5.3363015854,-11.3222492736
H,0,-6.518086924,4.7056364832,-11.5636900364
C,0,-4.5699762307,5.3584584251,-12.1672136795
H,0,-4.57313465,4.7437323096,-13.0665589463
C,0,-3.4318247986,6.160631194,-11.8758049386
C,0,-2.2771232345,6.2100112056,-12.7357970573
C,0,-2.2757049802,5.5735167295,-14.0797377707
C,0,-3.2850061914,5.8994061643,-15.056418994
C,0,-4.2310036523,6.9433794842,-14.8535036663
H,0,-4.2024079439,7.5146959321,-13.9271582808
C,0,-5.164713748,7.2590558568,-15.817943961
H,0,-5.8700500586,8.0736706034,-15.6403262048
C,0,-5.2156699431,6.5414057465,-17.0371249454
H,0,-5.9664608977,6.7956283878,-17.7881498878
C,0,-4.310007697,5.5303967,-17.2716458532
H,0,-4.3320032895,4.974461369,-18.2125129343
C,0,-3.3219905455,5.1964165318,-16.3059638103
C,0,-2.3543062125,4.1874726246,-16.5584076881

```
H,0,-2.4300202322,3.6149782152,-17.4881916728
C,0,-1.3477870732,3.9435113496,-15.6574155491
H,0,-0.5844574746,3.1883534915,-15.8356705731
C,0,-1.2894651873,4.665690069,-14.441713235
C,0,1.9758140137,5.8013151834,-10.7077722457
C,0,1.420507809,6.3255368773,-9.5398866516
H,0,0.3847286567,6.0834463164,-9.3021999587
C,0,2.1902691535,7.1339273805,-8.7017822346
H,0,1.7541803833,7.5450174189,-7.7887503171
C,0,3.522585077,7.4032205273,-9.0325177352
H,0,4.1379611228,8.0309083953,-8.3857168604
C,0,4.0768258765,6.8670101315,-10.192686969
H,0,5.1184129766,7.0668215473,-10.4391578371
C,0,3.3175564179,6.0599365992,-11.0538101734
C,0,3.9491029988,5.501773746,-12.2635169825
H,0,4.9263413458,5.9236471125,-12.5004773251
C,0,3.7154910506,4.1838136274,-12.7334379471
H,0,4.5329211957,3.7286044576,-13.2943348611
C,0,2.8366225154,3.2129760134,-12.0445507908
C,0,3.2609718458,1.8835578945,-11.8935940866
H,0,4.2255258903,1.5883645899,-12.3069865585
C,0,2.4734045705,0.9474373418,-11.2253068008
H,0,2.8320600594,-0.0784318902,-11.1172658072
C,0,1.2280719227,1.3135016977,-10.7048620206
H,0,0.6053091174,0.5778095323,-10.1914993963
C,0,0.7882850574,2.6320284306,-10.8358447368
H,0,-0.1727522185,2.9584520817,-10.4359225108
C,0,1.593686073,3.5713099366,-11.478175186
H,0,5.6992312443,2.3310535879,-15.5107720932
C,0,1.5360831792,3.9853421081,-19.1113283871
C,0,2.8856661405,3.6851627512,-18.8536912197
C,0,3.8830675792,4.442412024,-19.46802956
C,0,3.4943497612,5.4604789058,-20.3434148973
C,0,2.1376841394,5.7350383511,-20.5913198923
C,0,1.1257014466,5.0043963477,-19.9647969799
C,0,1.5449697222,2.1297587213,-17.8328722275
C,0,2.9327571384,2.5086051608,-17.9479019055
H,0,4.9391036889,4.2418267219,-19.2804313789
H,0,4.2582386455,6.0568679284,-20.8458982676
H,0,1.870194645,6.5373682186,-21.2815055096
H,0,0.0709968808,5.2133937828,-20.1490930688
H,0,-0.2471148542,3.0426334933,-18.3656383196
H,0,1.1309292901,1.2909278569,-17.2761088535
N,0,0.7675444972,3.023048189,-18.424963364
C,0,3.9924720393,1.4530239009,-18.2160587779
H,0,3.9568137986,1.1557471179,-19.2749229706
H,0,4.9940439048,1.8535266817,-18.0068772577
H,0,3.8484501984,0.5619137352,-17.5939122709
H,0,4.1351085172,3.7259140845,-16.4688825649
----------------------------------------------------------------------
```

TS-3-*endo*-*Re*-(*ap*)

Opt @ B3LYP-D3(BJ)/SDD/def2-SVP [Gaussian16]
SCF Done: E(RB3LYP) = -5024.50444279 a.u.
Zero-point correction = 1.255412 Hartree/Particle
Thermal correction to Gibbs Free Energy = 1.145403 Hartree/Particle
Imaginary Frequency = -336.2212 cm^{-1}
SP @ RI-B3LYP-D3(BJ)/def2-TZVPP in DCM (SMD) [ORCA 4.1]

FINAL SINGLE POINT ENERGY = -5026.535910399967 a.u.

Ir,0,2.8371924538,5.7684164942,-14.3201184738
Cl,0,5.0299126335,5.9645798256,-15.52126198
C,0,1.888916333,5.5058684946,-16.210083539
H,0,2.4801391371,5.9919794379,-16.980165765
H,0,0.8327269583,5.77710828,-16.2499591746
C,0,2.2289078477,4.1858516524,-15.7493869053
H,0,1.4013804874,3.6128913701,-15.3365658058
C,0,3.3473775619,3.3318936608,-16.208426229
C,0,3.3700289734,1.9329023128,-15.7046403303
C,0,2.2037114513,1.1827496881,-15.4764293971
H,0,1.2213443402,1.6305098033,-15.6283402938
C,0,2.2814625252,-0.1378774724,-15.0337239582
H,0,1.3643149627,-0.7022695202,-14.853197563
C,0,3.5260293791,-0.7325552154,-14.8068479886
H,0,3.5840940908,-1.7659168795,-14.457636412
C,0,4.6946754207,0.0034722925,-15.0220674127
H,0,5.6714313393,-0.4513110478,-14.843755241
C,0,4.6141476627,1.3205987333,-15.4709611031
P,0,2.9393016099,8.0967351125,-14.159015063
O,0,4.394740561,8.8598311981,-14.1457634214
O,0,2.3657713731,8.5370481894,-12.6602502394
N,0,2.2605216429,9.089005975,-15.3584264489
C,0,5.2074412727,8.9742970118,-13.0374574183
C,0,6.4439000022,8.2902111272,-13.0855373944
H,0,6.6604409889,7.6934452406,-13.9717184447
C,0,7.3157455794,8.3917048724,-12.0282032837
H,0,8.2792402521,7.8776185159,-12.0570631608
C,0,6.9679203053,9.1377480284,-10.8682461876
C,0,7.8290728242,9.1827487188,-9.7376786598
H,0,8.7913088894,8.6680242352,-9.7948305062
C,0,7.4570470377,9.84445974,-8.588187929
H,0,8.1251192078,9.8684198888,-7.724646509
C,0,6.1943498361,10.4811121132,-8.5229065823
H,0,5.8887074925,10.9835040875,-7.602322341
C,0,5.3427247335,10.4684382961,-9.6077606551
H,0,4.3693729456,10.9500818056,-9.5321005227
C,0,5.7046608213,9.8184113433,-10.8209841121
C,0,4.8313654848,9.7725386984,-11.9657493083
C,0,3.5428396189,10.5150732462,-11.9774893319
C,0,3.481498767,11.9333121671,-11.7443618531
C,0,4.6493279833,12.7435113942,-11.6782635364
H,0,5.6259461444,12.276969979,-11.8001774367
C,0,4.5599025291,14.1057312633,-11.4855381387
H,0,5.4708450874,14.7073782531,-11.4496542215
C,0,3.2988962475,14.7339582888,-11.3397327396
H,0,3.2435567386,15.812623216,-11.1781565978
C,0,2.1467131458,13.9831594058,-11.418708907
H,0,1.1669200635,14.458525724,-11.3286202394
C,0,2.2031686179,12.5791528753,-11.6391493009
C,0,1.0176164327,11.8104329987,-11.7987662753
H,0,0.0480081778,12.3036959711,-11.7003242718
C,0,1.0897429959,10.476699782,-12.1266720498
H,0,0.1965184362,9.8793917676,-12.3100403161
C,0,2.3525313652,9.8524021095,-12.2439506277
C,0,1.1340233314,8.6627293983,-16.1303287635
C,0,-0.1296731896,8.604346308,-15.5364241954

H,0,-0.2316970595,8.9107178176,-14.4968487964
C,0,-1.2304382581,8.1422784703,-16.2559495055
H,0,-2.2111162321,8.0968640692,-15.7843937575
C,0,-1.0677659784,7.7358345859,-17.5847812825
H,0,-1.9268385242,7.3665409806,-18.1479578936
C,0,0.1865388938,7.8087480277,-18.1847897749
H,0,0.3113172319,7.4954047955,-19.2245692408
C,0,1.3125886623,8.2818846226,-17.4813303328
C,0,2.6113349154,8.3124136112,-18.1492790107
H,0,2.6986816091,7.6451289102,-19.0143212005
C,0,3.6579205189,9.1388617979,-17.9081658557
H,0,4.5203204323,9.0608911094,-18.577342727
C,0,3.6854677711,10.2638651648,-16.980301534
C,0,4.446317641,11.4040787895,-17.305937749
H,0,5.0784410504,11.3784448965,-18.196965759
C,0,4.3819422537,12.560097158,-16.5315047119
H,0,4.9831914517,13.4308393862,-16.8017401299
C,0,3.5308605323,12.6081165296,-15.4227236875
H,0,3.4572365937,13.5122348502,-14.8148875514
C,0,2.7881611384,11.4808646506,-15.0708474227
H,0,2.1317102349,11.5028956029,-14.2066359188
C,0,2.8995707564,10.3026521245,-15.8084759953
P,0,0.9797996344,5.5316191827,-13.1402678784
O,0,0.0666049478,6.8621675675,-12.9077419108
O,0,-0.1329617656,4.4176272976,-13.6355740381
N,0,1.3100525682,4.8995473712,-11.5906163913
C,0,-1.0691258393,6.8071393435,-12.1003553882
C,0,-1.0231330017,7.5138558489,-10.8804307014
H,0,-0.1230591159,8.0763727538,-10.6369531744
C,0,-2.1061846496,7.462192868,-10.034883505
H,0,-2.0934826002,8.011540722,-9.0907447639
C,0,-3.2395184329,6.6649073613,-10.3541116404
C,0,-4.3300677809,6.5375923075,-9.4504187732
H,0,-4.2927919476,7.0867377675,-8.5064683125
C,0,-5.4052823848,5.7295507953,-9.747801637
H,0,-6.2346050947,5.6352868603,-9.0435641355
C,0,-5.4279287787,5.0058878056,-10.9645775825
H,0,-6.2703065499,4.3467322052,-11.1857755497
C,0,-4.3945692435,5.1206683969,-11.8703080977
H,0,-4.4235424694,4.5518748928,-12.7991876569
C,0,-3.2785469088,5.9619851626,-11.6056082089
C,0,-2.1835469886,6.1051537899,-12.5293720701
C,0,-2.2428795835,5.5581152147,-13.911049629
C,0,-3.3189846845,5.9203441319,-14.8001487292
C,0,-4.2906070714,6.9041306903,-14.4613122371
H,0,-4.2365038207,7.3967366949,-13.4921304883
C,0,-5.2844549848,7.2613251451,-15.348678922
H,0,-6.009471103,8.0278442596,-15.066436313
C,0,-5.3712770626,6.649597948,-16.6220962427
H,0,-6.168437746,6.9360290549,-17.3112304566
C,0,-4.4393073182,5.7038195304,-16.9889460854
H,0,-4.4836432922,5.2340879589,-17.9746974687
C,0,-3.3901233675,5.3300422191,-16.1056636827
C,0,-2.3842004553,4.4120767125,-16.5086533452
H,0,-2.4522532836,3.9491792117,-17.4956561005
C,0,-1.3207484242,4.1359893112,-15.6859791686
H,0,-0.5267366855,3.4594567417,-15.9972832682
C,0,-1.2428922345,4.7328298679,-14.4060834433

C,0,2.1176654688,5.7413656441,-10.7318209899
C,0,1.5806806283,6.1778712519,-9.5199944272
H,0,0.5709913224,5.8639616741,-9.2560077759
C,0,2.3321793686,6.9986574052,-8.6771723728
H,0,1.9093655006,7.3412890726,-7.7302702199
C,0,3.6270268703,7.3739808379,-9.0506559393
H,0,4.2251462557,8.0189220004,-8.4045248563
C,0,4.1662888502,6.9211451847,-10.2528662984
H,0,5.1812242941,7.2000016928,-10.5317589606
C,0,3.4273377408,6.0958979633,-11.1148464118
C,0,4.0473763732,5.6159560853,-12.3634655586
H,0,5.0031304908,6.084850481,-12.5998489593
C,0,3.8550648498,4.31125323,-12.8943020488
H,0,4.681371444,3.9228392278,-13.4934614819
C,0,3.0226465477,3.2599262615,-12.2695296188
C,0,3.465198833,1.9265547048,-12.2810291084
H,0,4.4207325106,1.6899366445,-12.7488256767
C,0,2.7041133072,0.9099349693,-11.7072193202
H,0,3.0740202226,-0.1169130539,-11.730669988
C,0,1.4711704071,1.1994762115,-11.1137556225
H,0,0.8685509448,0.4021361403,-10.6737836593
C,0,1.0142129208,2.5180219395,-11.0835363867
H,0,0.0571687043,2.7827338973,-10.6318256177
C,0,1.7891911876,3.535119889,-11.6391364175
H,0,5.5284771216,1.8901483084,-15.6526009188
C,0,5.5388202025,2.8296793711,-18.6753127284
C,0,4.3136904385,2.1701344506,-18.4569017271
C,0,4.2753104776,0.7770968806,-18.4960441779
C,0,5.4651014249,0.0857164312,-18.7447324787
C,0,6.6796306357,0.7651762308,-18.9374209236
C,0,6.7382514863,2.1613958273,-18.9058973492
C,0,3.9897130742,4.4397522981,-18.3939951196
C,0,3.2934700816,3.197017222,-18.1251415338
H,0,3.3452058716,0.2360871147,-18.316682393
H,0,5.4533031069,-1.0053285243,-18.7807140713
H,0,7.5924476746,0.1953037551,-19.1212360219
H,0,7.6766281399,2.696276921,-19.0629780005
H,0,5.9764435891,4.943566457,-18.6657553388
H,0,3.6159697628,5.4571129721,-18.3250976293
N,0,5.2714007314,4.2118520721,-18.6284638687
C,0,1.8454366293,2.9949342325,-18.5107798933
H,0,1.7533026283,2.8404238493,-19.5964033163
H,0,1.4441646916,2.1024798591,-18.0093894333
H,0,1.2309005104,3.8581707649,-18.224683461
H,0,4.3276678205,3.7984730615,-16.1085900123

TS-3-*exo*-*Re*-(+*sc*)
Opt @ B3LYP-D3(BJ)/SDD/def2-SVP [Gaussian16]
SCF Done: E(RB3LYP) = -5024.48858722 a.u.
Zero-point correction = 1.255975 Hartree/Particle
Thermal correction to Gibbs Free Energy = 1.147658 Hartree/Particle
Imaginary Frequency = -118.2755 cm^{-1}
SP @ RI-B3LYP-D3(BJ)/def2-TZVPP in DCM (SMD) [ORCA 4.1]
FINAL SINGLE POINT ENERGY = -5026.528672621346 a.u.

Ir,0,3.5480282984,5.8909842425,-14.6255195336
Cl,0,5.6008147234,5.7468365575,-16.0107843143

C,0,2.4449385922,5.6143090344,-16.4408907277
H,0,2.961921782,6.1312591378,-17.2484722568
H,0,1.3793931924,5.8362116837,-16.3680718304
C,0,2.9252400098,4.3221083038,-16.0524520156
H,0,3.8804724864,4.0302425756,-16.492985402
C,0,2.0315271871,3.1875547026,-15.727905379
H,0,1.1162847357,3.5216654411,-15.2366141082
C,0,2.6078280737,2.0127008432,-14.9957265984
C,0,3.8976902208,1.5286150681,-15.2644523171
H,0,4.5697720581,2.0981829328,-15.9101579369
C,0,4.3344512284,0.3206858156,-14.7151767628
H,0,5.3413776566,-0.0426042882,-14.9346368724
C,0,3.489741627,-0.4162048508,-13.8810674553
H,0,3.827083349,-1.3654625503,-13.458941067
C,0,2.227858784,0.0889780725,-13.5561481293
H,0,1.5790706484,-0.4557736501,-12.8678330609
C,0,1.7972172802,1.2962338915,-14.1019325188
P,0,3.7784597571,8.2425044875,-14.8072309943
O,0,5.2635297874,8.8750512978,-15.1159995702
O,0,3.4832355667,8.9225418139,-13.314077237
N,0,2.977226754,9.1324980089,-16.0170276728
C,0,6.2536463619,9.0381067103,-14.167547902
C,0,7.4163002872,8.247554005,-14.3175826268
H,0,7.4477265013,7.5409406686,-15.1462889473
C,0,8.4473705482,8.3814838516,-13.4191823878
H,0,9.3544254192,7.7829459299,-13.5306932928
C,0,8.3403745067,9.2711451776,-12.3147936458
C,0,9.3661650219,9.3542313603,-11.3336536723
H,0,10.2666071727,8.7501161453,-11.4691856235
C,0,9.2261849387,10.1630246699,-10.2273230127
H,0,10.0189698904,10.2148549976,-9.4779982726
C,0,8.0404073774,10.9169884042,-10.0550827678
H,0,7.9189496553,11.5387121743,-9.1650872668
C,0,7.0339303783,10.8704664699,-10.9970161645
H,0,6.1231784586,11.4458023156,-10.8391869672
C,0,7.1548168308,10.0662252761,-12.165023237
C,0,6.1137135783,9.9796015511,-13.1565853578
C,0,4.9049513222,10.8404618122,-13.0731096902
C,0,4.9984913359,12.2748305367,-13.0163980762
C,0,6.2268870671,12.9647335128,-13.2163500338
H,0,7.1313453156,12.3907656421,-13.4129437991
C,0,6.2821052917,14.3419120655,-13.1861965213
H,0,7.2353429322,14.848194111,-13.3537944558
C,0,5.1145695658,15.1072657833,-12.9467090775
H,0,5.1749936605,16.1973039076,-12.916562341
C,0,3.9046723243,14.4732219721,-12.7696078706
H,0,2.9927635411,15.0525074559,-12.6053240787
C,0,3.8094816577,13.0548431488,-12.8168878573
C,0,2.5535401285,12.3968423557,-12.7140916741
H,0,1.6534130634,12.993534463,-12.5501559173
C,0,2.4640904078,11.0339697372,-12.8755197006
H,0,1.5041752277,10.5187996002,-12.8608971929
C,0,3.6382901599,10.2734408585,-13.0814374381
C,0,1.6942642184,8.7318337487,-16.5081791539
C,0,0.5760783138,8.8204572915,-15.6742781696
H,0,0.7029949005,9.2325265563,-14.6755394287
C,0,-0.6699606576,8.3638122485,-16.1024237806
H,0,-1.5319122713,8.4289490347,-15.4395044442

C,0,-0.8038690888,7.8233478497,-17.3854320185
H,0,-1.7768355378,7.461125858,-17.7244428709
C,0,0.3024285285,7.7583425671,-18.2297092133
H,0,0.1970345953,7.3411873706,-19.2346018518
C,0,1.5713116952,8.2160578916,-17.8210441577
C,0,2.7073019337,8.0684430378,-18.7277180569
H,0,2.5810143501,7.3041399532,-19.5013035654
C,0,3.8419444383,8.8062580851,-18.7789482562
H,0,4.549205225,8.5766923516,-19.5814643228
C,0,4.1382783443,10.0045792305,-18.0030889964
C,0,4.9039540133,11.0295825147,-18.5934593103
H,0,5.3462033021,10.8542337277,-19.5771454088
C,0,5.0784178719,12.2574473872,-17.9600056319
H,0,5.6792266478,13.03569127,-18.4355664011
C,0,4.463484123,12.4956501021,-16.7268049595
H,0,4.5733252078,13.4591979962,-16.224857005
C,0,3.723110541,11.4837734203,-16.1158136564
H,0,3.2479996372,11.6572490929,-15.15646975
C,0,3.6032160723,10.2306627659,-16.7169329764
P,0,1.8485884896,5.9535010039,-13.1940494602
O,0,1.1466197971,7.4141580283,-12.9884394265
O,0,0.5283760863,4.9512239924,-13.3383090601
N,0,2.3511955178,5.4888803776,-11.6260135926
C,0,0.2658823466,7.6594637154,-11.942032872
C,0,0.6881883299,8.5440317085,-10.9276907126
H,0,1.6716916037,9.0011867353,-11.008611185
C,0,-0.1451799265,8.7863201612,-9.8612694735
H,0,0.1619756987,9.4753005908,-9.0711885207
C,0,-1.3910825935,8.1113927744,-9.7432664634
C,0,-2.2204820438,8.2846251328,-8.6011013317
H,0,-1.8903458875,8.9682440995,-7.8151668176
C,0,-3.4079285194,7.5974334997,-8.4773257112
H,0,-4.0338614528,7.7345634629,-7.5930028652
C,0,-3.811439643,6.6984993784,-9.4941822771
H,0,-4.7419821072,6.1377024847,-9.3815409181
C,0,-3.0388042257,6.5207055888,-10.6224210376
H,0,-3.3601746379,5.8211812896,-11.393718979
C,0,-1.816250359,7.2274859451,-10.7917397067
C,0,-0.985139973,7.0693946986,-11.9563498753
C,0,-1.4597707362,6.3547895878,-13.1683965345
C,0,-2.6960328196,6.759128729,-13.7987868328
C,0,-3.4001708506,7.9320716176,-13.4045867165
H,0,-3.0070823697,8.5446015964,-12.5955002237
C,0,-4.5576100889,8.3212214847,-14.0464812581
H,0,-5.0678832621,9.233559114,-13.7301638338
C,0,-5.0866302003,7.5566987406,-15.1131416299
H,0,-6.0083919324,7.8723762849,-15.6061227527
C,0,-4.4232553376,6.4250203291,-15.5334079152
H,0,-4.8077639776,5.8333248714,-16.3679202116
C,0,-3.2163739395,6.0120422571,-14.9069924122
C,0,-2.4909893629,4.8856609171,-15.3757445818
H,0,-2.9024795721,4.2887177196,-16.1935749031
C,0,-1.2718408875,4.5738281274,-14.8305169921
H,0,-0.701510227,3.7217625249,-15.1848267875
C,0,-0.7316010806,5.3341979869,-13.7664807378
C,0,3.3233216155,6.3599132435,-10.9928862382
C,0,3.0159389219,6.9312517281,-9.7573929585
H,0,2.0495068991,6.7086531141,-9.3064399639

C,0,3.934720866,7.7735251611,-9.1292986867
H,0,3.6904201048,8.2236601499,-8.1646246077
C,0,5.1656376066,8.0351561842,-9.7411589688
H,0,5.8911497085,8.698578209,-9.2667343915
C,0,5.4806718269,7.4377942021,-10.9599614031
H,0,6.450840895,7.6192430457,-11.4196520115
C,0,4.5726720877,6.5831009757,-11.6041731913
C,0,4.9677322183,5.9183015196,-12.859399686
H,0,5.9079521271,6.2801174722,-13.2766139559
C,0,4.6427512521,4.5598422086,-13.1644086104
H,0,5.3652512994,4.0359794368,-13.7934865354
C,0,3.8979694913,3.6751103491,-12.2409019363
C,0,4.3562569911,2.3681336933,-12.0096953226
H,0,5.2461776255,2.0199110551,-12.5358068504
C,0,3.7012035056,1.5235257787,-11.1156297863
H,0,4.0863394664,0.5161491322,-10.9462762486
C,0,2.5501864334,1.9541283936,-10.4481721089
H,0,2.0288988386,1.2869490015,-9.7583469263
C,0,2.0802707289,3.2516643302,-10.6558271082
H,0,1.1994189456,3.6329791682,-10.1369665865
C,0,2.764360598,4.1072977353,-11.5190293815
H,0,0.8134985478,1.6890920721,-13.8408969976
C,0,1.3735763607,0.2251464385,-17.2497102152
C,0,0.5616665656,1.3235398557,-16.9148118088
C,0,-0.6692874361,1.0860347475,-16.3075864229
C,0,-1.0297009233,-0.2368101211,-16.0250995513
C,0,-0.1804794522,-1.3108270767,-16.3365333189
C,0,1.0480273224,-1.0959676752,-16.9662525768
C,0,2.4814353922,2.0555391976,-17.9554862531
C,0,1.3107599814,2.5769639388,-17.2524923073
H,0,-1.3412855945,1.9036852322,-16.0453071448
H,0,-1.9912637303,-0.4391218634,-15.5492208924
H,0,-0.4874143508,-2.3293705806,-16.0923072533
H,0,1.7129612228,-1.9234878813,-17.2187087023
H,0,3.3075145086,0.1762607944,-18.1963207526
H,0,3.290410874,2.6330135105,-18.4030772936
N,0,2.5214073298,0.7450832897,-17.8885163567
C,0,0.5409859327,3.6946706753,-17.9530175368
H,0,-0.0141301031,3.2803096485,-18.8074930449
H,0,-0.1771081313,4.1671662015,-17.2721545746
H,0,1.2151596433,4.4771571587,-18.3187359065

TS-3-*exo*-*Re*-(-*sc*)
Opt @ B3LYP-D3(BJ)/SDD/def2-SVP [Gaussian16]
SCF Done: E(RB3LYP) = -5024.49445703 a.u.
Zero-point correction = 1.255881 Hartree/Particle
Thermal correction to Gibbs Free Energy = 1.147870 Hartree/Particle
Imaginary Frequency = -305.0013 cm^{-1}
SP @ RI-B3LYP-D3(BJ)/def2-TZVPP in DCM (SMD) [ORCA 4.1]
FINAL SINGLE POINT ENERGY = -5026.524052225290 a.u.

Ir,0,3.5805004385,5.8482965036,-14.5874048193
Cl,0,5.6129471364,5.709477926,-15.9907539392
C,0,2.4660748996,5.5969120204,-16.382943693
H,0,2.9896360649,6.0939795644,-17.1970753115
H,0,1.4088163869,5.8523788595,-16.2925560264
C,0,2.9141039889,4.2886917085,-16.0135595568

H,0,3.8381524536,3.9651924655,-16.4918793951
C,0,2.0060308428,3.1948178809,-15.6311607302
H,0,1.1575346056,3.5331725435,-15.0393327736
C,0,2.5984961559,1.9615412381,-15.048679591
C,0,3.8839365182,1.5121040323,-15.3998751609
H,0,4.513601481,2.1134475274,-16.0548074953
C,0,4.3660964641,0.2975843397,-14.9108299683
H,0,5.3656282742,-0.0401344346,-15.1940200264
C,0,3.5783145345,-0.4834023302,-14.0594055411
H,0,3.9562024808,-1.4371027186,-13.6842627858
C,0,2.3188721292,-0.0236581371,-13.66590267
H,0,1.7144329216,-0.6063815475,-12.9682620532
C,0,1.8387549551,1.1911637352,-14.1514076974
P,0,3.8154145878,8.1914094596,-14.7810607431
O,0,5.2961472372,8.8363009777,-15.0796921123
O,0,3.4941873278,8.8892023905,-13.3052328914
N,0,3.0319634223,9.0612291617,-16.0291278559
C,0,6.2770008284,9.013240191,-14.1234540164
C,0,7.4433233778,8.2255742233,-14.2555749609
H,0,7.4826415758,7.507153578,-15.0741357455
C,0,8.4686801244,8.3795183665,-13.3536831642
H,0,9.3798668923,7.7848190706,-13.450690526
C,0,8.3496209171,9.2850780811,-12.2633960563
C,0,9.3703418253,9.3890742514,-11.2789507277
H,0,10.2756518662,8.7896689121,-11.4020536273
C,0,9.2195459063,10.2119002031,-10.1844658715
H,0,10.008600946,10.2798297714,-9.4325429756
C,0,8.027324789,10.9588620937,-10.0274928282
H,0,7.8970559548,11.5911751313,-9.1462753059
C,0,7.02513216,10.8916700697,-10.9727533351
H,0,6.1091132448,11.4612558628,-10.825295929
C,0,7.1572809454,10.0732339276,-12.1295195021
C,0,6.1224926318,9.9662985269,-13.1260316577
C,0,4.9049085086,10.816769197,-13.0587675832
C,0,4.9844422312,12.2522803493,-13.0087735492
C,0,6.2057069648,12.9520013928,-13.2169867955
H,0,7.1147269966,12.3849512934,-13.4128251679
C,0,6.248083668,14.3298271528,-13.1970418661
H,0,7.1958393342,14.8438854298,-13.371782164
C,0,5.0743633051,15.085874897,-12.9583259177
H,0,5.1247431819,16.1765925387,-12.9357942795
C,0,3.8712010301,14.441786184,-12.7709534425
H,0,2.9548602563,15.0137719978,-12.6057692841
C,0,3.7890796212,13.0222986319,-12.8087500861
C,0,2.5395364827,12.3534068483,-12.6972508859
H,0,1.6349538604,12.9422683854,-12.5294216598
C,0,2.4617170576,10.9898914364,-12.8594215918
H,0,1.5066836306,10.4654886862,-12.8463077076
C,0,3.6424695585,10.2406066584,-13.0696308253
C,0,1.815430753,8.6087637657,-16.6233051256
C,0,0.6406664658,8.5698566603,-15.8646724148
H,0,0.6782367449,8.911631105,-14.8317629194
C,0,-0.5454819011,8.0704212007,-16.404247084
H,0,-1.4478030725,8.0322458378,-15.7945401682
C,0,-0.5717017138,7.6403493067,-17.7357516222
H,0,-1.5064215883,7.2836867879,-18.1753849666
C,0,0.5926426174,7.693861233,-18.5030245618
H,0,0.5663821333,7.3984593881,-19.5580950395

C,0,1.8134304669,8.159390298,-17.9685553968
C,0,3.0258412871,8.0643548374,-18.7781967211
H,0,2.9934406737,7.3102941512,-19.5713274365
C,0,4.1407798005,8.8296221101,-18.7129457672
H,0,4.9265996953,8.6309444581,-19.4474885826
C,0,4.3196594516,10.022782236,-17.8969535686
C,0,5.0896120756,11.0882084178,-18.4018705895
H,0,5.6338360368,10.9464414118,-19.3386558168
C,0,5.1393624675,12.3154143401,-17.7445915704
H,0,5.7463422463,13.1274124431,-18.1505436061
C,0,4.3917418289,12.5082872888,-16.5793207255
H,0,4.4039989424,13.4696902491,-16.0618926834
C,0,3.6465766036,11.4534294386,-16.0503363853
H,0,3.0742494343,11.5923039137,-15.1391785626
C,0,3.6524710297,10.2030393265,-16.6666753169
P,0,1.8885566754,5.9141123896,-13.1405113586
O,0,1.1872298156,7.3794151595,-12.9715319841
O,0,0.5606995328,4.907332064,-13.2349620353
N,0,2.4042753245,5.4645732391,-11.5743667716
C,0,0.2877661859,7.6556088797,-11.9492875311
C,0,0.7050620096,8.536764946,-10.9299771759
H,0,1.7061116284,8.9587638346,-10.9793199281
C,0,-0.1597038043,8.8232503539,-9.9004209647
H,0,0.1415204204,9.5110300111,-9.1070159494
C,0,-1.436249885,8.2011318407,-9.8263252748
C,0,-2.3040490123,8.4297368581,-8.7233222102
H,0,-1.9797125986,9.1166098613,-7.9378604805
C,0,-3.5210597937,7.7905229489,-8.6351761892
H,0,-4.1768862862,7.9698031026,-7.7806108678
C,0,-3.9152826444,6.8844133752,-9.6488184117
H,0,-4.8690455697,6.3590722358,-9.5629978961
C,0,-3.1045055598,6.6532217278,-10.740236009
H,0,-3.420919592,5.9454807494,-11.5055752537
C,0,-1.8514255227,7.312345896,-10.8750721052
C,0,-0.9768485695,7.1006706244,-12.0002301971
C,0,-1.4160337994,6.3333083672,-13.1933865118
C,0,-2.6303163121,6.7066646637,-13.8800483647
C,0,-3.3019699957,7.9324645775,-13.6112149668
H,0,-2.8970985134,8.6076680527,-12.8590124997
C,0,-4.4407451526,8.2884648324,-14.3036675537
H,0,-4.9273745919,9.2419246181,-14.0872488071
C,0,-4.9833230392,7.4340611564,-15.2930366594
H,0,-5.8920696302,7.72393091,-15.824525963
C,0,-4.3534660095,6.2445795909,-15.5874656708
H,0,-4.7536736151,5.5797649533,-16.3570236282
C,0,-3.1642010842,5.8630360032,-14.9095503397
C,0,-2.4773368549,4.6630036786,-15.2333157632
H,0,-2.9202618078,3.9727230814,-15.9559429802
C,0,-1.2638775124,4.381655912,-14.6552958596
H,0,-0.7346609617,3.4608595861,-14.8870859959
C,0,-0.696607389,5.2522319828,-13.6908283525
C,0,3.3876561485,6.3340760726,-10.9597679637
C,0,3.0874225366,6.9354810142,-9.7368227087
H,0,2.1185193986,6.7334207751,-9.2809514473
C,0,4.0166492989,7.7799792042,-9.127087742
H,0,3.7792557774,8.252593447,-8.1715079332
C,0,5.249772302,8.0140320055,-9.7454247895
H,0,5.983594504,8.6779667231,-9.2849599511

C,0,5.554955956,7.3908945916,-10.953863771
H,0,6.5252759466,7.5546407989,-11.4199738257
C,0,4.6363602616,6.5341799189,-11.5802279175
C,0,5.0157022118,5.8546644906,-12.832500959
H,0,5.9558986708,6.2049823329,-13.2595497383
C,0,4.6703183981,4.5024087336,-13.1396848076
H,0,5.3791666538,3.9706294431,-13.7775576828
C,0,3.9101543896,3.6233274588,-12.2239185446
C,0,4.3245100904,2.2947797739,-12.0364413639
H,0,5.1877055021,1.9268603613,-12.593038641
C,0,3.6555409489,1.4510618312,-11.1516987825
H,0,4.005287708,0.4257343209,-11.0176411544
C,0,2.5346414815,1.9059242394,-10.4500936392
H,0,2.0026004622,1.2404118477,-9.7669829298
C,0,2.1043282931,3.2227952789,-10.6199652181
H,0,1.2420737959,3.6181866591,-10.0808893443
C,0,2.8002178338,4.0751376911,-11.4766826984
H,0,0.8627730251,1.5557504236,-13.825179788
C,0,2.1567189434,3.640596082,-19.0047557441
C,0,2.1737646166,2.466595316,-18.2280739632
C,0,3.1360885791,1.4932922093,-18.4912004138
C,0,4.0743644907,1.7439065884,-19.500064528
C,0,4.0569507811,2.9394466171,-20.2370014431
C,0,3.0835357217,3.9150440547,-20.0046943102
C,0,0.4196428263,3.8067157175,-17.5834517343
C,0,1.1153424375,2.5929883144,-17.186149459
H,0,3.1640282147,0.5624119157,-17.9250133748
H,0,4.8372121111,0.9945498083,-19.7204111091
H,0,4.8093480393,3.1064341128,-21.0098938671
H,0,3.0550580714,4.8412179639,-20.5808738987
H,0,0.8486529376,5.3610019894,-18.8659065857
H,0,-0.4444171333,4.2710373487,-17.1090694582
N,0,1.053863495,4.4041726656,-18.5706444757
C,0,0.2755888195,1.3791827209,-16.8299181957
H,0,-0.3077691165,1.054330919,-17.7046618408
H,0,0.9156788764,0.5458681962,-16.5161811743
H,0,-0.423618311,1.5902994873,-16.0084190775

TS-3-*exo*-*Re*-(*ap*)

Opt @ B3LYP-D3(BJ)/SDD/def2-SVP [Gaussian16]
SCF Done: E(RB3LYP) = -5024.48738825 a.u.
Zero-point correction = 1.255526 Hartree/Particle
Thermal correction to Gibbs Free Energy = 1.146989 Hartree/Particle
Imaginary Frequency = -365.9160 cm^{-1}
SP @ RI-B3LYP-D3(BJ)/def2-TZVPP in DCM (SMD) [ORCA 4.1]
FINAL SINGLE POINT ENERGY = -5026.524453042643 a.u.

Ir,0,3.534318667,5.9621690119,-14.665665678
Cl,0,5.6004311336,5.8143451411,-16.0258696934
C,0,2.4318357566,5.6662820216,-16.4860219926
H,0,2.9419551983,6.189434146,-17.294871935
H,0,1.3596411606,5.8641285771,-16.4215327814
C,0,2.9297095686,4.3747875846,-16.1085514059
H,0,3.9060411727,4.1090604782,-16.5121194829
C,0,2.0904801549,3.2331327635,-15.7619569523
H,0,1.1148707037,3.5054115103,-15.3584798734
C,0,2.6867262895,2.0819984498,-15.0203530733

C,0,3.9959834548,1.6371648278,-15.2553998305
H,0,4.676769776,2.2341895549,-15.8638548269
C,0,4.4363148901,0.4225369058,-14.7210729274
H,0,5.4530342773,0.0798250807,-14.9262929918
C,0,3.5849473128,-0.3487987283,-13.9267967977
H,0,3.9293070427,-1.3003061297,-13.5159545807
C,0,2.3024802783,0.1242136156,-13.627495676
H,0,1.650047905,-0.4454041045,-12.9624443685
C,0,1.8607737793,1.330887915,-14.167294393
P,0,3.8176501678,8.2917012688,-14.800039486
O,0,5.3157789955,8.8978825446,-15.0845384674
O,0,3.5204615859,8.9459113235,-13.2986455641
N,0,3.0421114722,9.2141822743,-15.9965309843
C,0,6.30132502,9.0399901776,-14.1276540707
C,0,7.4576194508,8.2408535497,-14.2823825567
H,0,7.4891377218,7.548156283,-15.1226896117
C,0,8.4827197119,8.349882778,-13.3739088697
H,0,9.3848740928,7.744636439,-13.4886174225
C,0,8.375420443,9.2221320656,-12.2557060055
C,0,9.3934495458,9.2777249513,-11.2645656465
H,0,10.2888825589,8.6668665242,-11.4027958195
C,0,9.2517554017,10.0685108023,-10.1455244599
H,0,10.0382894294,10.0991123262,-9.3885156189
C,0,8.0721042558,10.8313344547,-9.9703187952
H,0,7.9489132858,11.4382958136,-9.0704318781
C,0,7.0738907719,10.812324449,-10.9220088352
H,0,6.167710195,11.3942778187,-10.7620305172
C,0,7.1973834513,10.0276725091,-12.1030184137
C,0,6.1634957316,9.9683148143,-13.1043938103
C,0,4.964567848,10.8420229322,-13.0167575768
C,0,5.076173072,12.274133713,-12.9366466579
C,0,6.3149418527,12.950986469,-13.1158570983
H,0,7.2136928784,12.368716629,-13.3141369114
C,0,6.3874687977,14.32672847,-13.0637541808
H,0,7.3484572031,14.8231933792,-13.2156249789
C,0,5.2278517191,15.1032623025,-12.8219503603
H,0,5.3019991404,16.1918114298,-12.7742941781
C,0,4.0084334304,14.4823286837,-12.6648446533
H,0,3.1026752479,15.0707761786,-12.4992627989
C,0,3.895549879,13.0662164407,-12.7351112663
C,0,2.630394122,12.4230388111,-12.653302407
H,0,1.7366703391,13.028666958,-12.4872440663
C,0,2.5246945618,11.0640297718,-12.8362971329
H,0,1.558346657,10.5610233391,-12.8359514849
C,0,3.6908196203,10.2920287039,-13.0440404971
C,0,1.749860029,8.856337477,-16.4942907005
C,0,0.6325186791,8.9641108436,-15.6617472355
H,0,0.7706740487,9.3436144279,-14.6520133251
C,0,-0.628488209,8.5697257457,-16.1072836983
H,0,-1.4898218298,8.6444115527,-15.4440812961
C,0,-0.7778073038,8.0811588479,-17.4086752532
H,0,-1.7631501263,7.7706660079,-17.7620796913
C,0,0.3299622158,7.994755301,-18.2483366341
H,0,0.2150015597,7.6254008268,-19.2704355946
C,0,1.6162719945,8.3763258746,-17.8189537023
C,0,2.7520715369,8.2000806264,-18.7215233749
H,0,2.6054350689,7.4502978204,-19.5059215306
C,0,3.9112810913,8.8994219795,-18.7565626681

H,0,4.6173556553,8.6551593242,-19.5556556161
C,0,4.2365469625,10.0800807132,-17.9650805521
C,0,5.0319088131,11.0918712328,-18.5385004038
H,0,5.47696074,10.9166414416,-19.5209042599
C,0,5.2313449342,12.3080306832,-17.8898214311
H,0,5.8545672228,13.076450154,-18.3522980219
C,0,4.6128861001,12.5482202809,-16.6586454543
H,0,4.742085755,13.5036140083,-16.1458801445
C,0,3.844001406,11.5481320867,-16.0636479017
H,0,3.366366707,11.7223948903,-15.1056070698
C,0,3.6986176052,10.3053989419,-16.6802883
P,0,1.8214748696,6.0358651253,-13.250354367
O,0,1.1487593491,7.5041855015,-12.9973329857
O,0,0.4912199833,5.0692235136,-13.4594447004
N,0,2.3018910334,5.5271545815,-11.6873418453
C,0,0.2695543641,7.7204282708,-11.9415924427
C,0,0.6913006359,8.5771491701,-10.9037863069
H,0,1.6705790501,9.0447618948,-10.9759563537
C,0,-0.1364965673,8.7789601859,-9.824208703
H,0,0.1707684328,9.4456155613,-9.015251519
C,0,-1.3743108062,8.0876277208,-9.7173383251
C,0,-2.1963573205,8.2132520549,-8.5635503488
H,0,-1.8654493752,8.8701512554,-7.755450159
C,0,-3.3784331642,7.513967304,-8.4570204617
H,0,-3.9985906722,7.6143206856,-7.5637248206
C,0,-3.7853840927,6.6516836286,-9.5041770352
H,0,-4.7128051546,6.0830540724,-9.406008538
C,0,-3.020196256,6.5206146326,-10.6436826265
H,0,-3.3430618602,5.8511815849,-11.4409550604
C,0,-1.8017411929,7.238618496,-10.7932606034
C,0,-0.9801453424,7.1277580047,-11.9687620019
C,0,-1.4700765801,6.4915261261,-13.2176399847
C,0,-2.6988263604,6.9633358808,-13.8132037792
C,0,-3.3945813137,8.1082798743,-13.3298570505
H,0,-2.9988419546,8.6554917967,-12.476754746
C,0,-4.549914875,8.5526718894,-13.9386049579
H,0,-5.0540819465,9.4411982845,-13.5523679629
C,0,-5.084873207,7.8758615679,-15.0605437538
H,0,-6.0054607904,8.2340244401,-15.52602154
C,0,-4.426514919,6.7782183059,-15.568757556
H,0,-4.8120756965,6.2564224555,-16.4483264304
C,0,-3.2207312125,6.3118508518,-14.9778121974
C,0,-2.4920009641,5.2390003489,-15.5501596176
H,0,-2.8923799431,4.7335081576,-16.4273520103
C,0,-1.2721217917,4.8707977209,-15.041671637
H,0,-0.6928106699,4.0822660252,-15.514112661
C,0,-0.7514741301,5.5122916007,-13.8932293544
C,0,3.265285286,6.3788575995,-11.0143185636
C,0,2.9436440915,6.9068286306,-9.7631938604
H,0,1.9731319711,6.6673201418,-9.3305995713
C,0,3.8533404056,7.7288364528,-9.0965103192
H,0,3.5966500528,8.145078821,-8.1199307959
C,0,5.0902632023,8.0152117338,-9.6849775248
H,0,5.8085545161,8.6638566834,-9.180008899
C,0,5.4221761173,7.4576237097,-10.9177795521
H,0,6.3991624922,7.6519815563,-11.3568021719
C,0,4.5243756445,6.6206458995,-11.598762498
C,0,4.9485733549,5.9748593992,-12.852847807

H,0,5.8830927902,6.3557176466,-13.2655186304
C,0,4.6371116455,4.6248747751,-13.1722574995
H,0,5.3580968439,4.1131855725,-13.8130153089
C,0,3.8740148349,3.7291907798,-12.2764097842
C,0,4.3448348961,2.4291944371,-12.031206756
H,0,5.2582866546,2.0957745755,-12.5250205186
C,0,3.6726814732,1.5742434525,-11.160355034
H,0,4.0682938415,0.5735257363,-10.9768008866
C,0,2.4915880323,1.9860879545,-10.5344103032
H,0,1.9566086348,1.3095511837,-9.8644575736
C,0,2.0109266261,3.2776278666,-10.7537971143
H,0,1.1099123546,3.6463299147,-10.2611238634
C,0,2.7129894062,4.1457521726,-11.5899822625
H,0,0.8575774173,1.6987674102,-13.9374719035
C,0,-0.7495366114,2.3995756643,-17.7624794385
C,0,0.3802980031,3.2192649262,-17.964267358
C,0,0.2148805946,4.4428738952,-18.6132050441
C,0,-1.0687170221,4.80611897,-19.0348276413
C,0,-2.1765535215,3.9724188683,-18.8179234139
C,0,-2.0359969762,2.7442961781,-18.1655717283
C,0,1.0342046927,1.207271972,-17.0720551617
C,0,1.5800732675,2.4951078188,-17.4527684495
H,0,1.064541665,5.0987731234,-18.7976702549
H,0,-1.2130891642,5.755285429,-19.5520840805
H,0,-3.1623860318,4.2839015315,-19.1684208896
H,0,-2.8894858619,2.0835288821,-18.0030109519
H,0,-0.88327366,0.4228242095,-16.9132563471
H,0,1.5762825648,0.3422008965,-16.6958486842
N,0,-0.2854608446,1.1973024803,-17.1898101848
C,0,2.824345804,2.4897534636,-18.3276075022
H,0,2.5994443187,2.0269657637,-19.3003006435
H,0,3.1655705354,3.5170799825,-18.5087920779
H,0,3.6423172673,1.9273141793,-17.8587885003

TS-3-*exo*-*Si*-(+*sc*)
Opt @ B3LYP-D3(BJ)/SDD/def2-SVP [Gaussian16]
SCF Done: E(RB3LYP) = -5024.49044559 a.u.
Zero-point correction = 1.255677 Hartree/Particle
Thermal correction to Gibbs Free Energy = 1.146337 Hartree/Particle
Imaginary Frequency = -302.7485 cm^{-1}
SP @ RI-B3LYP-D3(BJ)/def2-TZVPP in DCM (SMD) [ORCA 4.1]
FINAL SINGLE POINT ENERGY = -5026.527487232105 a.u.

Ir,0,3.5560431915,5.8238634218,-14.5617544476
Cl,0,5.6188099288,5.6386735385,-15.9178395095
C,0,2.4861377266,5.5445340129,-16.3927735012
H,0,3.0268202556,6.0377586842,-17.1997395196
H,0,1.4253167238,5.7903801281,-16.3390828052
C,0,2.9240989623,4.2411836475,-15.9915428529
H,0,3.876450839,3.9028722608,-16.3995978579
C,0,1.9987760319,3.1735230677,-15.6105023245
H,0,1.0901773058,3.5584727829,-15.1519996687
C,0,2.5211685476,1.9881847702,-14.8668018861
C,0,3.7693954332,1.419266926,-15.1558853506
H,0,4.4433344594,1.909947114,-15.8588278784
C,0,4.1483927516,0.2082737114,-14.5696144558
H,0,5.1193127928,-0.228096712,-14.8146473527

C,0,3.2899458564,-0.4445254519,-13.6830870674
H,0,3.5857248963,-1.3936072431,-13.2302746959
C,0,2.0649259073,0.1428114743,-13.3471829448
H,0,1.4101771822,-0.3338285548,-12.6149127478
C,0,1.6894930344,1.3516892791,-13.9302314366
P,0,3.8017915448,8.161488306,-14.7904309457
O,0,5.2934323139,8.7763937482,-15.0939200917
O,0,3.490856807,8.8710964484,-13.3155643281
N,0,3.0193682046,9.0281924966,-16.028674697
C,0,6.2742462165,8.9561023578,-14.1382667693
C,0,7.4354060846,8.1587336451,-14.2599684556
H,0,7.4725802889,7.4343569937,-15.0729394403
C,0,8.4583276135,8.3096908876,-13.354935253
H,0,9.3646425128,7.7064370389,-13.4446961341
C,0,8.3436218612,9.2241516628,-12.2717447215
C,0,9.3608954756,9.3265559458,-11.2835718568
H,0,10.2608829933,8.7173681917,-11.3975362581
C,0,9.2134030208,10.1604437954,-10.1970450844
H,0,9.99972643,10.2272083117,-9.4421382682
C,0,8.0282151168,10.921085775,-10.0523349843
H,0,7.9006657037,11.5630193772,-9.1776809802
C,0,7.0299064349,10.8559166565,-11.001829801
H,0,6.119493151,11.4370857891,-10.8648138207
C,0,7.1589961971,10.025476502,-12.1504557504
C,0,6.1269897547,9.9198270289,-13.1496894105
C,0,4.9210801294,10.786984774,-13.0982809969
C,0,5.0214489163,12.2219552105,-13.0725931547
C,0,6.2553759106,12.9010496672,-13.2754716066
H,0,7.1589145343,12.3185252268,-13.4497743338
C,0,6.3172785578,14.2782771759,-13.2763195042
H,0,7.2747403018,14.7759027872,-13.445683852
C,0,5.1512999778,15.0546668479,-13.0666880719
H,0,5.2170335085,16.1447871327,-13.0609574978
C,0,3.9363410618,14.4308415265,-12.8880857653
H,0,3.025684821,15.0181611439,-12.7471757027
C,0,3.834462856,13.0122282529,-12.9040168184
C,0,2.5739954627,12.363068152,-12.8010704961
H,0,1.675209329,12.9677634614,-12.6609245086
C,0,2.4790745532,10.9974621576,-12.9336551547
H,0,1.5161325324,10.4881205043,-12.9190128309
C,0,3.6514197042,10.2266396268,-13.1090457828
C,0,1.7345353409,8.6322611506,-16.519170397
C,0,0.6149601804,8.7400214324,-15.6894481877
H,0,0.7420969894,9.1661272297,-14.6967951416
C,0,-0.6327209249,8.2829403621,-16.1129854979
H,0,-1.4955395233,8.36099789,-15.4523745518
C,0,-0.7672963692,7.7261000952,-17.3889022899
H,0,-1.7413392277,7.3640792196,-17.7250303952
C,0,0.3401567173,7.6440999158,-18.2303094404
H,0,0.2342977108,7.2132054697,-19.2292603191
C,0,1.6112842933,8.0986550938,-17.8251882669
C,0,2.7484807508,7.9240276791,-18.7255407571
H,0,2.6153946881,7.1492345101,-19.4874724361
C,0,3.893098261,8.6457706672,-18.7826395815
H,0,4.6009674392,8.3948591976,-19.5781874105
C,0,4.1999988743,9.8527662946,-18.0245902363
C,0,4.97904668,10.8595100807,-18.6288143249
H,0,5.4246754136,10.6622396028,-19.6067837845

C,0,5.161578271,12.0970273177,-18.0168374716
H,0,5.7724840185,12.8607220502,-18.5029690618
C,0,4.541101734,12.3638175979,-16.7922958273
H,0,4.6562228602,13.3356443183,-16.3078197941
C,0,3.7878217216,11.3701903506,-16.1673902206
H,0,3.3078906568,11.5659230735,-15.2149013763
C,0,3.660523103,10.1071309854,-16.7456440235
P,0,1.8392417957,5.9166509467,-13.1442262031
O,0,1.1399602292,7.381535725,-12.9793812685
O,0,0.5201276024,4.9070146187,-13.2652422036
N,0,2.3328416482,5.4851593242,-11.5630250511
C,0,0.2518593092,7.6577733189,-11.9469366687
C,0,0.6698996613,8.5663753905,-10.9524384195
H,0,1.6572135617,9.0148238324,-11.0350911886
C,0,-0.1729433182,8.8419412711,-9.9017598016
H,0,0.1299958728,9.5500864291,-9.1271576672
C,0,-1.4243562749,8.1780678264,-9.7793449175
C,0,-2.2647561336,8.3886349664,-8.6516289146
H,0,-1.9388252509,9.092546709,-7.8820350856
C,0,-3.4575715064,7.7121272147,-8.5211358023
H,0,-4.092135558,7.8780988559,-7.6479930024
C,0,-3.8552966596,6.7866894199,-9.5161417076
H,0,-4.790173313,6.234400951,-9.3976154495
C,0,-3.0718187695,6.5722374772,-10.630489642
H,0,-3.3895326714,5.8522802257,-11.3841848543
C,0,-1.8434652603,7.2671302383,-10.8070952074
C,0,-1.0004511647,7.0720412999,-11.9580137494
C,0,-1.4666907325,6.3177645689,-13.1495174042
C,0,-2.6984258856,6.7039092008,-13.7986816294
C,0,-3.3899105707,7.901611843,-13.4605366599
H,0,-2.9899373418,8.5473221959,-12.6809641038
C,0,-4.5427554643,8.2720215109,-14.1213511423
H,0,-5.0432969016,9.2039680015,-13.8498442997
C,0,-5.0800862836,7.4624019164,-15.1500994025
H,0,-5.9984274559,7.7639243188,-15.6580853246
C,0,-4.4301100287,6.3037729101,-15.5140590984
H,0,-4.8224410176,5.675534451,-16.3175972225
C,0,-3.2276117363,5.9086854786,-14.8677583585
C,0,-2.5194617383,4.7474010444,-15.2726372032
H,0,-2.9446653933,4.1079833332,-16.0498632077
C,0,-1.300993987,4.4499366091,-14.7165819581
H,0,-0.7503686373,3.5655893903,-15.024963724
C,0,-0.7442972629,5.2664489421,-13.7011577123
C,0,3.3024741707,6.3656339102,-10.9407661378
C,0,2.9843006093,6.9707946883,-9.724244138
H,0,2.0111547939,6.7663353993,-9.2792020872
C,0,3.9014457607,7.822784959,-9.1069972572
H,0,3.6488544162,8.2990620857,-8.1571410833
C,0,5.1414554037,8.0606230504,-9.7102164987
H,0,5.8658153653,8.7307623364,-9.243642812
C,0,5.4666100684,7.431218866,-10.9099596881
H,0,6.4434145325,7.5945937835,-11.3623438572
C,0,4.5603064236,6.5660838009,-11.5428281848
C,0,4.9648517949,5.8700590565,-12.7768653486
H,0,5.9071746739,6.2208936711,-13.1985806013
C,0,4.6321254701,4.5114702744,-13.0614023515
H,0,5.3500406829,3.9728287544,-13.6834263971
C,0,3.8703382059,3.6480424223,-12.1332284666

C,0,4.3127415591,2.3386815399,-11.8837476258
H,0,5.1962700582,1.9705400376,-12.4074764025
C,0,3.6505733942,1.5183931386,-10.9726986288
H,0,4.0244031433,0.5098016765,-10.7861264583
C,0,2.50647173,1.9745034008,-10.3099269236
H,0,1.9804386637,1.3261734405,-9.6058628613
C,0,2.0472765448,3.271434669,-10.5431717369
H,0,1.16923627,3.6697922045,-10.032452774
C,0,2.7385794094,4.1041397658,-11.4230186236
H,0,0.7394519544,1.8165129176,-13.6544854722
C,0,2.4161077517,0.5671119112,-17.7986008495
C,0,2.44126122,1.9618565972,-17.9774218786
C,0,3.4933557703,2.5380049942,-18.6897447441
C,0,4.4938341158,1.697170392,-19.1889028594
C,0,4.4495639732,0.3061608837,-18.9880067498
C,0,3.3991852106,-0.2889441408,-18.285836344
C,0,0.573913569,1.3703289917,-16.7808066189
C,0,1.2930283001,2.5477180006,-17.2314030057
H,0,3.5455766795,3.6161151228,-18.8454613322
H,0,5.3279475917,2.1292778888,-19.7450469132
H,0,5.2481681318,-0.3203721755,-19.3897571734
H,0,3.3606519782,-1.3670880649,-18.1215478573
H,0,1.015957022,-0.6557629153,-16.712214004
H,0,-0.3557011047,1.3313523734,-16.2142556467
N,0,1.2518618275,0.270085253,-17.0604387927
C,0,0.5003219748,3.6728586402,-17.8783633819
H,0,-0.2364414006,4.1126696201,-17.1939927099
H,0,1.1742127764,4.4742110447,-18.2004354612
H,0,-0.0250582872,3.2955190896,-18.7686150406

TS-3-*exo*-*Si*-(-*sc*)
Opt @ B3LYP-D3(BJ)/SDD/def2-SVP [Gaussian16]
SCF Done: E(RB3LYP) = -5024.48735990 a.u.
Zero-point correction = 1.255480 Hartree/Particle
Thermal correction to Gibbs Free Energy = 1.146642 Hartree/Particle
Imaginary Frequency = -268.0799 cm^{-1}
SP @ RI-B3LYP-D3(BJ)/def2-TZVPP in DCM (SMD) [ORCA 4.1]
FINAL SINGLE POINT ENERGY = -5026.525047942666 a.u.

Ir,0,3.5246199258,5.9291956831,-14.6007611374
Cl,0,5.5907305266,5.7865903842,-15.9741632805
C,0,2.4240610181,5.6104058422,-16.4188108439
H,0,2.9182252585,6.1381585158,-17.2343735836
H,0,1.3462204322,5.7833810584,-16.3629853802
C,0,2.9473972511,4.3352876826,-16.0159273054
H,0,3.9177496114,4.0717279654,-16.4396256506
C,0,2.1058602269,3.1831149625,-15.6609167348
H,0,1.1342986488,3.4840731238,-15.2673258785
C,0,2.6913101482,2.0654655688,-14.858655008
C,0,4.0106757917,1.6218425932,-15.0537746975
H,0,4.6906422956,2.1965959216,-15.6867894251
C,0,4.4626910633,0.4489350535,-14.4497490245
H,0,5.4867705102,0.1097383047,-14.6222596796
C,0,3.6119279246,-0.2867255615,-13.6173829121
H,0,3.9634642715,-1.2101432131,-13.1523067751
C,0,2.3269012609,0.1885761822,-13.3528277088
H,0,1.673009767,-0.3504180014,-12.6647196329

C,0,1.873145612,1.3583015032,-13.9658182112
P,0,3.7866180283,8.2809435146,-14.7337121173
O,0,5.2819328187,8.8779018677,-15.0594364404
O,0,3.5213351114,8.9557434253,-13.2309177912
N,0,2.9859657078,9.2117119895,-15.9084816427
C,0,6.2925893338,9.0172009415,-14.1285595529
C,0,7.4410608324,8.2113260516,-14.3054459715
H,0,7.4494271296,7.5134344595,-15.141546455
C,0,8.4869877304,8.3181183925,-13.4207401989
H,0,9.3823742887,7.7063345617,-13.5528584078
C,0,8.4115269367,9.1968357166,-12.3051359908
C,0,9.4526248143,9.2522970273,-11.3382397098
H,0,10.3401561957,8.6337228009,-11.4923744405
C,0,9.3428145842,10.0528247532,-10.2225854989
H,0,10.1467771647,10.0832513177,-9.4841006849
C,0,8.1734617428,10.8263525069,-10.0264653547
H,0,8.0759468142,11.4416601284,-9.1291060201
C,0,7.1533119185,10.8073410767,-10.9545524504
H,0,6.2556485088,11.3979301894,-10.7788698379
C,0,7.2431771466,10.0117269348,-12.1313140739
C,0,6.1860790949,9.9519934098,-13.1078755054
C,0,4.998352469,10.8376798388,-13.0018289198
C,0,5.1314160661,12.2693554564,-12.94169633
C,0,6.3754520463,12.9267952089,-13.1552133032
H,0,7.2617450292,12.330084864,-13.3658471859
C,0,6.4684479835,14.3018743437,-13.1202121898
H,0,7.4330350961,14.7826328173,-13.2977303949
C,0,5.3254454251,15.0977960463,-12.8628304806
H,0,5.416040036,16.1856104266,-12.8287205927
C,0,4.1009692083,14.4961143805,-12.6741390289
H,0,3.206997681,15.0990237958,-12.4969339889
C,0,3.9670522704,13.0810730983,-12.7260384628
C,0,2.6957346487,12.4562082343,-12.6114433973
H,0,1.8134221425,13.0753338316,-12.4345227765
C,0,2.5684549007,11.0967060812,-12.7743368618
H,0,1.5945889539,10.6104331117,-12.7429066001
C,0,3.7174209177,10.3028207076,-12.996545959
C,0,1.6481992411,8.9063023578,-16.3120069172
C,0,0.5916349536,9.1091797677,-15.4202831544
H,0,0.8129289674,9.5176711898,-14.438091138
C,0,-0.7144416094,8.7768162902,-15.7744719478
H,0,-1.5286153246,8.9252466737,-15.0658889983
C,0,-0.9731620779,8.2587782346,-17.0471178805
H,0,-1.9954001823,8.0002544584,-17.3319990019
C,0,0.0734056392,8.0880220873,-17.9497846616
H,0,-0.1293614899,7.7112432458,-18.9517548
C,0,1.4043016669,8.4024514311,-17.6103505568
C,0,2.4675170469,8.1683041905,-18.5848389493
H,0,2.2387601481,7.4081464984,-19.3363257519
C,0,3.6435426841,8.827565437,-18.7147013874
H,0,4.2819078788,8.5485027768,-19.5585701469
C,0,4.0682192643,10.006238756,-17.9679658865
C,0,4.8552626373,10.9808750019,-18.6133189514
H,0,5.2184193895,10.7781565863,-19.6238327153
C,0,5.1481368555,12.195626336,-17.9987469218
H,0,5.7625247764,12.9347598288,-18.5174097778
C,0,4.6335921812,12.4727526818,-16.7279037643
H,0,4.8356369997,13.4284930495,-16.239828215

C,0,3.8744094632,11.5099143471,-16.0637653387
H,0,3.4761945032,11.7132599564,-15.0759223771
C,0,3.6364624253,10.2662846116,-16.649943077
P,0,1.8107249874,5.9878855868,-13.1918378949
O,0,1.0990974482,7.4460475992,-13.0118055456
O,0,0.5080596972,4.968225101,-13.3451394805
N,0,2.298879102,5.5567339748,-11.6109941367
C,0,0.1933943024,7.6945999782,-11.9878981523
C,0,0.5853700063,8.5962243957,-10.976465494
H,0,1.5665154624,9.0603733915,-11.041774029
C,0,-0.2705989098,8.8422636417,-9.9290057513
H,0,0.0135004149,9.5446256439,-9.1420730246
C,0,-1.509299947,8.1522587969,-9.8249158505
C,0,-2.3610766867,8.3284063285,-8.6997883414
H,0,-2.0550866109,9.0276462011,-7.9178389173
C,0,-3.5396097309,7.6243213841,-8.5868181071
H,0,-4.182919308,7.763546182,-7.7154001175
C,0,-3.9106964067,6.7046754935,-9.5974422537
H,0,-4.833445385,6.1297244083,-9.4925113876
C,0,-3.1161972556,6.5240026955,-10.7099034753
H,0,-3.4130109867,5.808034861,-11.4757820571
C,0,-1.9024897093,7.2483584566,-10.8688380897
C,0,-1.0494048167,7.0882813306,-12.0178404028
C,0,-1.4972740213,6.3507569506,-13.2264913713
C,0,-2.7342967172,6.7264786071,-13.8710504237
C,0,-3.4585609859,7.8963248279,-13.5039965459
H,0,-3.0769405551,8.533096618,-12.7080274137
C,0,-4.621263451,8.2517131309,-14.1551384309
H,0,-5.1469783583,9.1624461239,-13.860220504
C,0,-5.1378847024,7.4526468009,-15.2030281299
H,0,-6.0655868581,7.7403616555,-15.7020035505
C,0,-4.4570793611,6.3212353954,-15.5944197984
H,0,-4.8343050018,5.7002373425,-16.4108517825
C,0,-3.2429362273,5.9439721479,-14.9584994316
C,0,-2.5042542723,4.8131390949,-15.3921102892
H,0,-2.9183249233,4.1816068491,-16.1821091953
C,0,-1.2781385566,4.5313615712,-14.8444012759
H,0,-0.6989967839,3.6772402479,-15.1849334449
C,0,-0.7522209125,5.3254423062,-13.7971200317
C,0,3.2536661108,6.445969975,-10.975025027
C,0,2.9204036465,7.0318427962,-9.7531239003
H,0,1.9445024979,6.8148626368,-9.3204330572
C,0,3.8275533895,7.8773809934,-9.1128912136
H,0,3.5625711562,8.3387079721,-8.1590408275
C,0,5.0752831245,8.1239922327,-9.6961500115
H,0,5.7946747304,8.7851306496,-9.2093522467
C,0,5.4156000104,7.5130874697,-10.9010687313
H,0,6.39859946,7.6823337634,-11.3370417231
C,0,4.517135873,6.6600837316,-11.5602991198
C,0,4.9386518677,5.9846095811,-12.8010234334
H,0,5.8746910245,6.3551964634,-13.2198014215
C,0,4.6281472848,4.6245818197,-13.0878115803
H,0,5.3524885981,4.0956363931,-13.7103432391
C,0,3.8881710006,3.7560390649,-12.1438442548
C,0,4.38745825,2.4806068124,-11.837355336
H,0,5.30061026,2.1393713108,-12.326309096
C,0,3.7455752495,1.6595713472,-10.9120652726
H,0,4.1630008788,0.6769005964,-10.6839322196

C,0,2.5670361179,2.081032586,-10.2887217241
H,0,2.0554083373,1.4305475902,-9.576161744
C,0,2.0594304759,3.3508373539,-10.566460015
H,0,1.1605885872,3.7292206501,-10.0771900553
C,0,2.7317747538,4.1861031049,-11.458283365
H,0,0.8660588018,1.7246775136,-13.7575804008
C,0,1.9791313492,3.7066752609,-19.0850973715
C,0,0.9703320229,3.4279927157,-18.1451243728
C,0,-0.2490520467,4.0920289223,-18.2526284643
C,0,-0.4183293269,5.0130188956,-19.2908101463
C,0,0.612633574,5.2852977087,-20.2038978862
C,0,1.845345943,4.634271322,-20.1121014755
C,0,2.7368834726,1.9935542259,-17.8339951999
C,0,1.4763925805,2.3732412653,-17.2186337505
H,0,-1.0599685016,3.9016382344,-17.5529577258
H,0,-1.3736271903,5.5305052399,-19.3928806897
H,0,0.4488079114,6.0112941836,-21.0025161535
H,0,2.6494200797,4.8306419793,-20.8233333716
H,0,3.931958933,2.8207157548,-19.3114173411
H,0,3.4247702801,1.2182378152,-17.5016282394
N,0,3.0428679114,2.817456043,-18.8159893208
C,0,0.5076776405,1.27424576,-16.8051529146
H,0,1.0154193147,0.4708946245,-16.2586786514
H,0,-0.2815750609,1.6771872715,-16.1546752969
H,0,0.0242482879,0.8453205763,-17.6952696811

TS-3-*exo*-*Si*-(ap)
Opt @ B3LYP-D3(BJ)/SDD/def2-SVP [Gaussian16]
SCF Done: E(RB3LYP) = -5024.49078817 a.u.
Zero-point correction = 1.255560 Hartree/Particle
Thermal correction to Gibbs Free Energy = 1.147112 Hartree/Particle
Imaginary Frequency = -357.6052 cm^{-1}
SP @ RI-B3LYP-D3(BJ)/def2-TZVPP in DCM (SMD) [ORCA 4.1]
FINAL SINGLE POINT ENERGY = -5026.524390306597 a.u.

Ir,0,3.6166833186,5.8682247269,-14.5519891791
Cl,0,5.6972856623,5.7140821355,-15.8776761844
C,0,2.6133457222,5.5618297863,-16.4089990317
H,0,3.1827623391,6.0407861283,-17.204932522
H,0,1.5496787777,5.8079993909,-16.3884309515
C,0,3.0208972918,4.2638755511,-15.9641026011
H,0,3.9792189512,3.9012225482,-16.3360380696
C,0,2.0363632491,3.2266378981,-15.6375378154
H,0,1.123213367,3.6307405189,-15.2053403335
C,0,2.4655882149,1.9912691715,-14.939978141
C,0,3.7208154868,1.3985870706,-15.1700327332
H,0,4.4572319638,1.9023701502,-15.7969807078
C,0,4.037238135,0.1664163538,-14.5972018415
H,0,5.0105922497,-0.2880515251,-14.79481141
C,0,3.118167768,-0.4796190488,-13.7644133329
H,0,3.3683875596,-1.4445708948,-13.3178927505
C,0,1.8926103488,0.1284061099,-13.481163705
H,0,1.1849635978,-0.3513682117,-12.8024110706
C,0,1.5686937032,1.3497050784,-14.0688053295
P,0,3.8344266893,8.2080235577,-14.7735861957
O,0,5.3115086074,8.8629761407,-15.0615952595
O,0,3.4845499831,8.9213635359,-13.3114759367

N,0,3.0461267819,9.0417370289,-16.0381266457
C,0,6.2759652573,9.0542646298,-14.0897400299
C,0,7.4497392538,8.2736630821,-14.1940000198
H,0,7.5112829235,7.5514575331,-15.0074666433
C,0,8.4551357205,8.4379228925,-13.2716277507
H,0,9.3714813028,7.8480879902,-13.3472835976
C,0,8.3086084534,9.3480133512,-12.1885656862
C,0,9.3072495122,9.4623088794,-11.1828423791
H,0,10.2177484408,8.8664778127,-11.2828051831
C,0,9.1290574937,10.2910206252,-10.096922068
H,0,9.9013344123,10.3669747396,-9.3285219573
C,0,7.9308147054,11.0343057873,-9.9706226353
H,0,7.7792854859,11.6720076094,-9.0967108282
C,0,6.9497533385,10.9572412948,-10.93704907
H,0,6.0287695766,11.524760221,-10.8140552704
C,0,7.1103623718,10.131573264,-12.0850143731
C,0,6.0972311342,10.0131121522,-13.1018621121
C,0,4.8767250172,10.8608694179,-13.0688458284
C,0,4.9509356531,12.2971226357,-13.037870397
C,0,6.1754195544,12.9983577904,-13.2209326999
H,0,7.0915370136,12.4322035116,-13.3834231607
C,0,6.2127358764,14.3764625423,-13.2172721503
H,0,7.1634162803,14.8916360034,-13.3715921611
C,0,5.0302361544,15.1313142249,-13.0218680603
H,0,5.0765404574,16.222399807,-13.0121628525
C,0,3.8242114564,14.4855918989,-12.8610065533
H,0,2.9016584492,15.0564194547,-12.7300000765
C,0,3.747749549,13.0654358529,-12.8823055871
C,0,2.4977534647,12.3937641154,-12.797129714
H,0,1.5866875621,12.9820050718,-12.6664997749
C,0,2.4287206213,11.0272910548,-12.9372360668
H,0,1.4747616548,10.5010795162,-12.9419928156
C,0,3.617480062,10.2789735489,-13.0997378065
C,0,1.8111898673,8.5753194188,-16.5863315318
C,0,0.6596475595,8.5707895599,-15.7923131268
H,0,0.7245822562,8.9672854073,-14.7814965576
C,0,-0.5375784931,8.0359430947,-16.2687577564
H,0,-1.4211555417,8.0220735948,-15.6303128798
C,0,-0.5947659189,7.5310972716,-17.5728798395
H,0,-1.5381924831,7.1407930887,-17.964534813
C,0,0.5429761538,7.5565422538,-18.3784266414
H,0,0.4979652583,7.1708359218,-19.4008681693
C,0,1.7702105395,8.0705034175,-17.9101560607
C,0,2.9517376203,7.9664051155,-18.7629207436
H,0,2.8918395265,7.1962223351,-19.5384312957
C,0,4.0630246579,8.7398292307,-18.7569552556
H,0,4.8186294972,8.5309384538,-19.5198945469
C,0,4.2750145499,9.9490280317,-17.972068281
C,0,5.0322866953,10.9986872156,-18.528167334
H,0,5.5361965209,10.8334979686,-19.4834655726
C,0,5.1211151998,12.2375194059,-17.8980132674
H,0,5.7176403059,13.0354282678,-18.3453759199
C,0,4.4257448864,12.4598307151,-16.7057490566
H,0,4.4676127843,13.4306944795,-16.2077480515
C,0,3.6928571265,11.4230153647,-16.1275174267
H,0,3.1584714214,11.5853061016,-15.1979598657
C,0,3.659339334,10.161068132,-16.7201593233
P,0,1.8886993528,5.9467431985,-13.1422842734

O,0,1.1853352409,7.4106517887,-12.9779328557
O,0,0.5735410704,4.9339223757,-13.2675730188
N,0,2.3726575045,5.5088191328,-11.5592399921
C,0,0.3197776478,7.6960842843,-11.9297232425
C,0,0.7580846859,8.6079422521,-10.9473652947
H,0,1.7468007297,9.0493392097,-11.0456747186
C,0,-0.0686122652,8.8947858315,-9.8870901313
H,0,0.249584765,9.6056964836,-9.1212389786
C,0,-1.3211557651,8.2377996277,-9.7417417972
C,0,-2.1428460575,8.4611108319,-8.6027612325
H,0,-1.8024755345,9.1709116363,-7.8449481483
C,0,-3.3349051416,7.7888166371,-8.4465713937
H,0,-3.9548256663,7.964196863,-7.5648219879
C,0,-3.7497602576,6.8543873482,-9.4258205565
H,0,-4.6827738055,6.303871478,-9.2863350388
C,0,-2.9849631791,6.6277899876,-10.550808577
H,0,-3.316342333,5.8991997789,-11.2899503821
C,0,-1.7588093198,7.3189721427,-10.7548308623
C,0,-0.932608742,7.1126813411,-11.9168986493
C,0,-1.4178639045,6.3396409715,-13.0881666562
C,0,-2.6742402197,6.7041107733,-13.7015531849
C,0,-3.3440000874,7.9207974123,-13.3888237
H,0,-2.8997178502,8.6033266591,-12.6662872041
C,0,-4.5327316882,8.2592634166,-14.0012446924
H,0,-5.017002613,9.2059001868,-13.7522108768
C,0,-5.1299579028,7.3954118591,-14.9499899771
H,0,-6.0784224063,7.6699271209,-15.4162310165
C,0,-4.5013903886,6.2175823527,-15.2895260569
H,0,-4.943136396,5.5470177862,-16.0312592254
C,0,-3.261173018,5.8564435057,-14.6970931806
C,0,-2.573215595,4.6735152816,-15.073599173
H,0,-3.0564334907,3.9765164959,-15.7613984793
C,0,-1.3185224136,4.4090769463,-14.5841129183
H,0,-0.7878263232,3.504610138,-14.872709458
C,0,-0.7122219563,5.2747687956,-13.6398761121
C,0,3.3362892546,6.3818501192,-10.9199894762
C,0,3.011233795,6.9703338918,-9.6970448464
H,0,2.038242333,6.7548555274,-9.2567839441
C,0,3.9209796461,7.8219737193,-9.0684263
H,0,3.663364615,8.2850791756,-8.1134312492
C,0,5.1591564222,8.0782479706,-9.6679592089
H,0,5.8764328292,8.7506405926,-9.1937627686
C,0,5.4917564682,7.4641885757,-10.873762037
H,0,6.4676643316,7.6408924274,-11.3233235981
C,0,4.5939653494,6.5968694592,-11.5157860782
C,0,5.0070300753,5.910223794,-12.751741484
H,0,5.9521274383,6.2652000358,-13.1637750729
C,0,4.6811948286,4.5528059812,-13.0519645279
H,0,5.4084417297,4.0237989031,-13.6712349641
C,0,3.8973725684,3.6715840218,-12.160299067
C,0,4.3035268687,2.3406138939,-11.9680785019
H,0,5.1816762048,1.9734516001,-12.5014423215
C,0,3.6128359869,1.4975383844,-11.1000025548
H,0,3.95763517,0.4716637974,-10.9584698653
C,0,2.4781916385,1.9546146096,-10.4218875344
H,0,1.9305332062,1.2891664123,-9.7511711647
C,0,2.051881668,3.2709055787,-10.6022866422
H,0,1.1762613347,3.6664166887,-10.0853069412

```
C,0,2.7680748407,4.122395536,-11.4429663266
H,0,0.6057221841,1.815907888,-13.8539739924
C,0,-0.9312212936,2.1971514762,-17.2057472777
C,0,0.3301375691,1.5886219343,-17.052484081
C,0,0.3952036396,0.2494029285,-16.6663761341
C,0,-0.8020207146,-0.433068182,-16.4273095869
C,0,-2.0477787963,0.2007401027,-16.5700233422
C,0,-2.1348120937,1.5370416441,-16.9696127103
C,0,0.6002148078,3.7493724104,-17.7735044219
C,0,1.3771664888,2.6060004639,-17.335234861
H,0,1.3549832748,-0.2541066881,-16.5479192512
H,0,-0.7691947181,-1.4813854776,-16.1242838503
H,0,-2.9637505329,-0.3611567773,-16.3779993686
H,0,-3.1008094883,2.0256215036,-17.106654493
H,0,-1.4150329017,4.2243176986,-17.768038708
H,0,0.9618733309,4.7152116862,-18.1195241594
N,0,-0.6961049553,3.5153264479,-17.6466826096
C,0,2.5910327815,2.2082732548,-18.1560714213
H,0,3.2674358725,3.0594848754,-18.3108668069
H,0,3.1457158551,1.4057415568,-17.6525711533
H,0,2.2737721099,1.8313162676,-19.1398478787
-------------------------------------------------------------------
```

References

1. J. Y. Hamilton, D. Sarlah, E. M. Carreira, *Org. Synth*. **2015**, *92*, 1-12.

2.M. Lafrance, M. Roggen, E. M. Carreira, *Angew. Chem., Int. Ed*. **2012**, *51*, 3470-3473.

3.P. M. Killoran, S. B. Rossington, J. A. Wilkinson, J. A. Hadfield, *Tetrahedron Lett*. **2016**, *57*, 3954-3957.

4. A. W. J. Logan, J. S. Parker, M. S. Hallside, J. W. Burton, *Org. Lett*. **2012**, *14*, 2940-2943.

5. M. Saravanan, B. Satyanarayana, P. P. Reddy, *Synth. Commun*. **2013**, 2050-2056.

6. C. Morrill, G. L. Beutner, R. H. Grubbs, *J. Org. Chem*. **2006**, *71*, 7813-7825.

7. Y.-M. Su, Y. Hou, F. Yin, Y.-M. Xu, Y. Li, X. Zheng, X.-S. Wang, *Org. Lett*. **2014**, *16*, 2958-2961.

8. P.A. Patel, N. Kvaratskhelia, Y. Mansour, J. Antwi, L. Feng, P. Koneru, M. J. Kobe, N. Jena, G. Shu, M. S. Mohamed, C. Li, J. J. Kessl, J. R. Fuchs, *Bioorg. Med. Chem. Lett*. **2016**, *26*, 4748-4752.

9. G. Angelovski, M. D. Keränen, P. Linnepe, S. Grudzielanek, P. A. Eilbracht, *Adv. Synth. Catal*. **2006**, *348*, 1193-1199.

10. M. Kimura, M. Futamata, R. Mukai, Y. Tamaru, *J. Am. Chem. Soc*. **2005**, *127*, 4592-4593.

11. C. A. Kuttruff, H. Zipse, D. Trauner, *Angew. Chem., Int. Ed*. **2011**, *50*, 1402-1405.

12. I. Choi, H. Chung, J. W. Park, Y. K. Chung, *Org. Lett*. **2016**, *18*, 5508-5511.

13. C. Seck, M. D. Mbaye, S. Gaillard, J.-L. Renaud, *Adv. Synth. Catal*. **2018**, *360*, 4640-4645.

14. R. Hirschmann, K. C. Nicolaou, S. Pietranico, E. M. Leahy, J. Salvino, B. Arison, M. A. Cichy, P. G. Spoors, W. C. Shakespreare, P, A. Sprengeler, P. Hamley, A. B. Smith III, T. Reisine, K. Raynor, L. Maechler, C. Donaldson, W. Vale, R. M. Freidinger, M. R. Cascieri, C. D. Strader, *J. Am. Chem. Soc*. **1993**, *115*, 12550-12568.

15. D. L. Priebbenow, L. C. Henderson, F. M. Pfeffer, S. G. Stewart, *J. Org. Chem*. **2010**, *75*, 1787-1790.

16. R. B. Bedford, N. Fey, M. F. Haddow, R. F. Sankey, *Chem. Commun*. **2011**, *47*, 3649-3651.

17. L. Cooper, J. M. Alonso, L. Eagling, H. Newson, S. Herath, C. Thomson, A. Lister, C. Howsham, B. Cox, M. P. Muñoz, *Chem. Eur. J*. **2018**, *24*, 6105-6114.

18. W.-B. Liu, H. He, L.-X. Dai, S.-L. You, *Synthesis*, **2009**, *12*, 2076-2082.

19. Gaussian 16, Revision A.03, M. J. Frisch, G. W. Trucks, H. B. Schlegel, G. E. Scuseria, M. A. Robb, J. R. Cheeseman, G. Scalmani, V. Barone, G. A. Petersson, H. Nakatsuji, X. Li, M. Caricato, A. V. Marenich, J. Bloino, B. G. Janesko, R. Gomperts, B. Mennucci, H. P. Hratchian, J. V. Ortiz, A. F. Izmaylov, J. L. Sonnenberg, D. Williams-Young, F. Ding, F. Lipparini, F. Egidi, J. Goings, B. Peng, A. Petrone, T. Henderson, D. Rana-singhe, V. G. Zakrzewski, J. Gao, N. Rega, G. Zheng, W. Liang, M. Hada, M. Ehara, K. Toyota, R. Fukuda, J. Hasegawa, M. Ishida, T. Nakajima, Y. Honda, O. Kitao, H. Nakai, T. Vreven, K. Throssell, J. A. Jr. Montgomery, J. E. Peralta, F. Ogliaro, M. J. Bearpark, J. J. Heyd, E. N. Brothers, K. N. Kudin, V. N. Staroverov, T. A. Keith, R. Kobayashi, J. Normand, K. Raghavachari, A. P. Rendell, J. C. Burant, S. S. Iyengar, J. Tomasi, M. Cossi, J. M. Millam, M. Klene, C. Adamo, R. Cammi, J. W. Ochterski, R. L. Martin, K. Morokuma, O. Farkas, J. B. Foresman, D. J. Fox, Gaussian, Inc., Wallingford CT, 2016.

20. (a) F. Neese, *Wiley Interdiscip. Rev.: Comput. Mol. Sci*. **2012**, *2*, 73. (b) F. Neese, *Wiley Interdiscip. Rev.: Comput. Mol. Sci.* **2017**, *8*, e1327.

21. (a) A. D. Becke, *J. Chem. Phys*. **1993**, *98*, 5648-5652. (b) C. Lee, W. Yang, R. G. Parr, *Phys. Rev. B: Condens. Matter Mater. Phys*. **1988**, *37*, 785-789. (c) P. J. Stephens, F. J. Devlin, C. F. Chabalowski, M. J. Frisch, *J. Phys. Chem*. **1994**, *98*, 11623-11627.

22. (a) S. Grimme, S. Ehrlich, L. Goerigk, *J. Comput. Chem*. **2011**, *32*, 1456-1465. (b) S. Grimme, J. Antony, S. Ehrlich, H. A. Krieg, *J. Chem. Phys*. **2010**, *132*, 154104-154122.

23. (a) F. Weigend, R. Ahlrichs, *Phys. Chem. Chem. Phys*. **2005**, *7*, 3297-3305. (b) F. Weigend, *Phys. Chem. Chem. Phys*. **2006**, *8*, 1057-1065.

24. D. Andrae, U. Haeussermann, M. Dolg, H. Stoll, *Theor. Chim. Acta*. **1990**, *77*, 123-141.

25. Marenich, A. V.; Cramer, C. J.; Truhlar, D. G. *Universal Solvation Model Based on Solute Electron Density and on a Continuum Model of the Solvent Defined by the Bulk Dielectric Constant and Atomic Surface Tensions. J. Phys. Chem*. *B*, **2009**, *113*, 6378-6396.
26. C. Y. Legault, CYLView, 1.0b; Université de Sherbrooke: Montreal, Québec, Canada, 2009; http://www.cylview.org.
27. W. Humphrey, A. Dalke, K. Schulten, *J. Mol. Graphics* **1996**, *14*, 33.
28. B. Bhaskararao, R. B. Sunoj, *J. Am. Chem. Soc*. **2015**, *137*, 15712-15722.
29. S. L. Rössler, S. Krautwald, E. M. Carreira, *J. Am. Chem. Soc*. **2017**, *139*, 3603-3606.
